# Proteomics for optimizing therapy in acute myeloid leukemia: venetoclax plus hypomethylating agents versus conventional chemotherapy

**DOI:** 10.1038/s41375-024-02208-8

**Published:** 2024-03-26

**Authors:** Eduardo Sabino de Camargo Magalhães, Stefan Edward Hubner, Brandon Douglas Brown, Yihua Qiu, Steven Mitchell Kornblau

**Affiliations:** 1 Department of Ageing Biology/ERIBA, University of Groningen, University Medical Center Groningen, Groningen, 9713 AV the Netherlands; 2 John Sealy School of Medicine, The University of Texas Medical Branch at Galveston, Galveston, TX 77555 USA; 3 Division of Pediatrics, The University of Texas MD Anderson Cancer Center, Houston, TX 77030-4009 USA; 4 Department of Leukemia, The University of Texas MD Anderson Cancer Center, Houston, TX 77030-4009 USA

**Keywords:** Acute myeloid leukaemia, Combination drug therapy, Chemotherapy, Translational research

## Abstract

The use of Hypomethylating agents combined with Venetoclax (VH) for the treatment of Acute Myeloid Leukemia (AML) has greatly improved outcomes in recent years. However not all patients benefit from the VH regimen and a way to rationally select between VH and Conventional Chemotherapy (CC) for individual AML patients is needed. Here, we developed a proteomic-based triaging strategy using Reverse-phase Protein Arrays (RPPA) to optimize therapy selection. We evaluated the expression of 411 proteins in 810 newly diagnosed adult AML patients, identifying 109 prognostic proteins, that divided into five patient expression profiles, which are useful for optimizing therapy selection. Furthermore, using machine learning algorithms, we determined a set of 14 proteins, among those 109, that were able to accurately recommend therapy, making it feasible for clinical application. Next, we identified a group of patients who did not benefit from either VH or CC and proposed target-based approaches to improve outcomes. Finally, we calculated that the clinical use of our proteomic strategy would have led to a change in therapy for 30% of patients, resulting in a 43% improvement in OS, resulting in around 2600 more cures from AML per year in the United States.

## Introduction

Acute Myeloid Leukemia (AML) is characterized by the uncontrolled clonal expansion of hematopoietic precursors. Although the majority of patients achieve remission, most ultimately relapse. Despite recent innovation in therapy [1], AML remains a fatal diagnosis for the majority, especially the elderly population [2, 3]. The identification of recurrent chromosomal abnormalities and common somatic mutations has improved the understanding of leukemogenesis, leading to revision in both diagnostic and prognostic categorization of AML [4–7]. However, most of these mutations lack therapies that can directly target them [8].

Since the 1970s, anthracycline combined with cytosine arabinoside (AraC), hereafter referred to as conventional chemotherapy (CC), has been the standard of care in AML induction therapy [9]. Despite being the backbone of AML treatment, it has been challenged with more target-based therapies [10, 11]. Increasing evidence has demonstrated that some patients with newly diagnosed AML benefit from the combination of venetoclax (VEN) and hypomethylating agents (HMA), such as Azacytidine or Decitabine, hereafter referred to as VH [12, 13]. Moreover, achieving long-term remission is still challenging in AML [14], and the VH combination has proven advantageous for use in patients with relapse [15]. However, it has been reported that specific groups of patients may not benefit from VH [16]. Moreover, despite the improved molecular classification of AML and the resulting improvement in prognostication for outcome, these schemas do not predict which of the available regimens individual patients will respond best to, especially older patients [17, 18]. Most patients are selected for CC or VH treatments based on clinical characteristics such as age, performance status, or occasionally cytogenetics and/or individual mutations, rather than on characteristics of the underlying pathophysiology of the leukemic blasts that cause differential responses to different therapeutic options [19]. Therefore, incorrect therapy triaging reduces the effectiveness and cure fraction achieved.

The ability to recognize which patients are more likely to respond to one regimen versus another is crucial for maximizing outcomes with existing therapies. Previous studies from our group using reverse-phase protein array (RPPA)-based proteomics have demonstrated that leukemia (AML, ALL, CML, and CLL) is characterized by a limited number of recurrent proteomic signatures, which are prognostic for outcome [20–28]. RPPA is a high-throughput microarray that can quantitatively measure the levels of hundreds of proteins in more than 1000 samples in a single array, using very little biological material [29, 30]. We investigated whether this technique could be leveraged to identify proteomic signatures associated with a superior response to CC vs. VH therapies in AML.

In the present study, we identified specific protein profiles associated with an improved response to CC or VH therapy using machine learning algorithms to develop a Protein Classifier based on the expression of a limited set of proteins that could be utilized clinically to recommend either VH, CC, or neither. Revised triaging based on these calculated predictions was estimated to increase the 5-year cure rate by 43%. Furthermore, we identified potentially targetable signaling hubs for a group of patients who did not benefit from either VH or CC.

## Materials and methods

### Study design, ethics statement, and patient population

The use of AML samples in the present study was approved by the MD Anderson Cancer Center (MDACC) Investigational Review Board (IRB), according to previously approved protocols (LAB01-473, Lab05-0654). Informed consent was obtained for sample use in compliance with the Declaration of Helsinki. PB and BM samples were collected from 810 adult patients (>17 years old) with newly diagnosed AML admitted to the MDACC between April 2012 and June 2020. Patients were included in the analysis if they received VH combination therapy (*N* = 85) or Conventional Chemotherapy (CC) (*N* = 369), predominantly anthracycline and cytosine arabinoside. Patients who were not treated at the MDACC (*N* = 115), or did not receive VH nor CC (*N* = 241) were excluded.

### Sample collection and processing

Immediately after harvesting, the samples were cooled to 4° C and processed within two hours. Fresh samples were layered on a Ficoll gradient, washed with PBS, and then counted. When T and B cells represented more than 5% of the post-Ficoll cells, CD3 and CD19 positive cells were removed by Magnetic Activated Cell Sorting (MACS) using the Miltenyi AutoMACS Magnetic Cell Sorter. Sample concentrations were normalized to 1 × 10^4^ cells/mL, and whole-cell lysates were prepared as previously described [31].

### Reverse-phase protein arrays (RPPA)

RPPA was performed in the MDACC RPPA Core Facility as described previously [20, 21, 23, 31, 32]. Briefly, whole-cell lysates were subjected to five serial 2× dilutions (1:1, 1:2, 1:4, 1:8, and 1:16) and printed onto nitrocellulose-coated glass slides. To determine protein expression levels, slides were probed with 411 validated primary antibodies (322 total and 89 post-translational modified (PTM)), together with secondary antibodies conjugated to an infrared molecule. The primary antibodies used were validated, as previously described [33]. Stained slides were quantitated with Microvigene (Version 3.4, Vigene Tech), and expression was normalized to normal bone marrow (NBM)-derived CD34+ cells. More specifically, the mean expression of NBM was normalized to zero and the values of each AML sample are expressed in Log2-fold-change (LFC) values compared to NBM. The antibodies used are listed in Supplementary Table S1.

### Computational analysis

Data analysis was performed using R v4.3.2 (“Eye Holes”) and Python3. To identify the proteins that significantly affected patient prognosis, the expression level of a single protein was split into quantiles: median split, tertiles, quartiles, quintiles, and sextiles, resulting in the formation of five groups. Overall survival (OS) was compared between quantiles in each case. This was repeated for each of the 411 proteins, resulting in the generation of a *p*-value table (Supplementary Table S2). Prognostic proteins were defined using two significance cutoffs: *p* < 0.05 and *p* < 0.01. Next, patients underwent unbiased hierarchical clustering according to their protein expression using the progeny clustering algorithm [34]. The protein set that showed clusters with clearly distinct protein expression profiles and most significant cluster separation in Kaplan–Meier (KM) plots for OS and complete remission duration (CRD), was chosen for further analysis and named protein selector set (PS). Three protein selector sets (PS1, PS2, and PS3) were developed to cover different population subsets. In order to create a stricter contrast between VH and CC for outcome analyses, patients who received HMA + VEN and AraC were removed from the VH group after the generation of PS1, leaving a total of 79. Similarly, the CC population was filtered for AraC-treated patients only, reducing the number of patients in this group to 340. The list of selected proteins for the PS1, PS2, and PS3, along with their respective *p*-values generated from the initial assessment can be found in Supplementary Table S3. Protein networks were made with Cytoscape v3.10.1 (ref. [35]), the StringApp [36], and the R package Rcy3(ref. [37]). Pathway enrichment analysis was performed using the Enrichr webtool. To assess the significancy of each biological process, a combination of adjusted *p*-values and odds-ratio, entitled ‘combined score’ was used. Ontologies were filtered using an adjusted *p*-value cutoff <0.01, and the combination of lowest adjusted *p*-value and highest odds-ratio (i.e., highest combined score) were considered the most significant. Further details of the methodology can be found elsewhere [38–40].

For Machine learning analysis, datasets were separated into developmental (dev) and test sets using an 80/20 split. Dev sets were further separated into training and validation sets using a 75/25 split. Model weights were initialized using replicable random states. Random forest machine learning algorithms were used in Python3 from the sklearn.ensemble package (scikit-learn) with specific importation of the RandomForestClassifier function. Hyperparameter tuning involved the application of two individually assembled Python functions: holdout_grid_search and random_forest_grid_search. Grid search was performed to optimize hyper-parameters, including the number of trees in the random forest and their maximum depth. 150 hyperparameter search-spaces were evaluated based on the unique n_estimators, max_depth, and min_samples_leaf hyperparameter combinations. Shapley Additive Explanations (SHAP) values were calculated to explain the model predictions by quantifying the additive importance of each feature. SHAP functions were imported from the shap library. For each of the 3 protein classifier models, all available proteins served as inputs into the aforementioned random forest algorithm, and the output was a SHAP-based hierarchy of the most predictive proteins. Few proteins (defined as 6 or less proteins) were tested from the top 6 proteins in each model to train the final version of each random forest model. The combination of proteins that generated the highest C-index for each model were isolated and reported. C-index calculation was used to evaluate model accuracy, using the formula: ((#concordant pairs + 0.5*#ties)/(#permissible pairs)).

### Statistical analysis

LogRank tests with *p*-values adjusted by the Benjamini–Hochberg (BH) method were used to compare outcomes. Pearson’s correlation coefficient was used to measure the linear the correlation between proteins. Fisher’s exact test, Wilcoxon or Kruskal–Wallis tests were used to compare measured variables. Univariate (UV) and multivariate (MV) models were build using Cox proportional-hazards (CoxPH). Wilcoxon tests adjusted by the False Discovery Rate (FDR), with the cutoff *p* < 0.05, and mean Log2-fold change values, with a threshold of 0.5, were used for differential expression analysis. Statistical significance was defined as a *p*-value < 0.05, and significance symbols were determined as ****p < 0.0001, ***p < 0.001, **p < 0.01, *p < 0.05, and ns not significant.

## Results

### Protein selector sets (PS) identify patient groups with distinct clinical outcomes

**Fig. 1:**
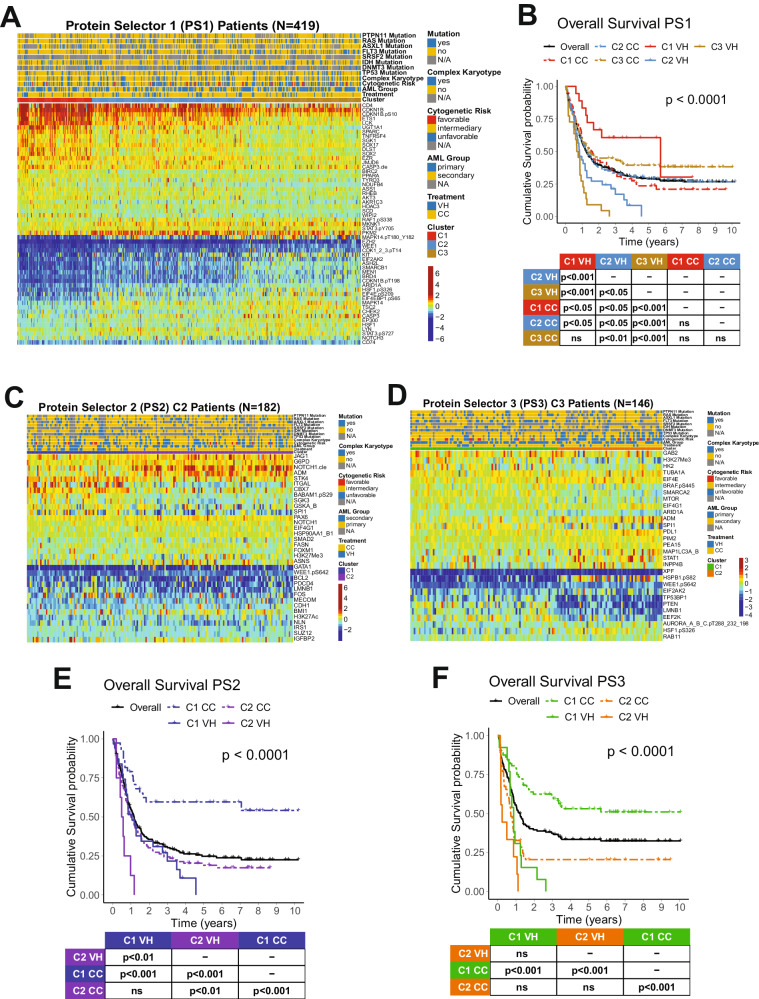
Protein expression and clinical outcomes of patients clustered with PS1. **A** Heatmap depicting the protein expression of PS1 patients (*N* = 419). **B** Kaplan–Meier plots of Overall Survival PS1 patients (*N* = 419) separated by cluster and treatment modality (VH = solid line, CC = dashed line; PS1-C1 = red, PS1-C2 = light blue, PS1-C3 = yellow). **C** Heatmap depicting the protein expression of PS2 patients (*N* = 182) and **D** PS3 patients (*N* = 146). **E** Kaplan–Meier plots of Overall Survival from PS2 patients (*N* = 182) and **F** PS3 patients (*N* = 146) separated by cluster and treatment modality (VH = solid line, CC = dashed line; PS2-C1 = blue, PS2-C2 = purple, PS3-C1 = orange, PS3-C2 = green). Annotations above the heatmaps, starting closest to the heatmap, show the clusters, VH vs. CC treatment modality (second from bottom), and then other annotations for several previously recognized prognostic features including AML group, cytogenetic risk, and presence of complex karyotype and mutations. Colors for the annotations have the value shown in the legends along the right side. Protein expression ranging from above normal (red) to normal (yellow-green-aqua) to below normal (dark blue) as shown in the color legend.

We developed an algorithm to identify the most therapeutically discriminating proteins and generated Protein Selector Sets (see “Materials and Methods” section). The first one, entitled PS1, was comprised of 55 proteins, which identified three clusters (C1, C2, and C3) with unique expression signatures. Protein levels across the clusters are shown in Fig. 1A. Although the protein signature of each cluster was the same in both patients with VH and CC, their overall survival (OS) varied greatly between treatments. As shown in Fig. 1B, patients in C1 (red) treated with VH (solid line) had diametrically different and superior responses compared to those treated with CC (dashed line), with a Median OS (MS) of 68.5 months (mo.) in the VH group versus (vs.) MS of 19.4 mo. in the CC population. The opposite was true for C3 (yellow), where CC patients had a MS of 16.8 mo. and the VH population displayed a very poor MS of 8.7 mo. However, PS1 did not identify an optimal therapy for patients in cluster C2 (light blue). Therefore, to identify the preferred therapy for PS1-C2 patients (*N* = 182), we generated PS2, using the same strategy described previously. As shown in Fig. 1C, PS2 separated the population into two clusters with distinct expression profiles. In Fig. 1E, cluster PS2-C1 (blue color) treated with CC (dashed line) had a markedly better OS (>120 mo.), compared to C1-VH (solid blue), which has a MS of 12.7 mo. The same was true for cluster PS2-C2 (purple color), where CC (dashed line) had a MS 12.2 mo., and VH (solid line) had a MS of 6.4 mo. Moreover, as shown in Fig. 1B, the best PS1-C3 curve (dashed yellow, CC-treated) has an OS comparable to the worst PS1-C1 group (dashed red, CC-treated). Therefore, we generated a PS3 for PS1-C3 patients (*N* = 146) in an attempt to identify a group with better OS. Within PS3, two clusters with contrasting protein expression levels were defined, and separated by treatment (Fig. 1D). As shown in Fig. 1F, patients in cluster PS3-C1 (green color) had a very good prognosis when treated with CC (dashed line), with MS > 120 mo., and a very poor outcome when treated with VH (solid line), having a MS of 10.4 mo. In contrast, OS of patients in PS3-C2 (orange color) were similarly poor for both therapies.

**Fig. 2:**
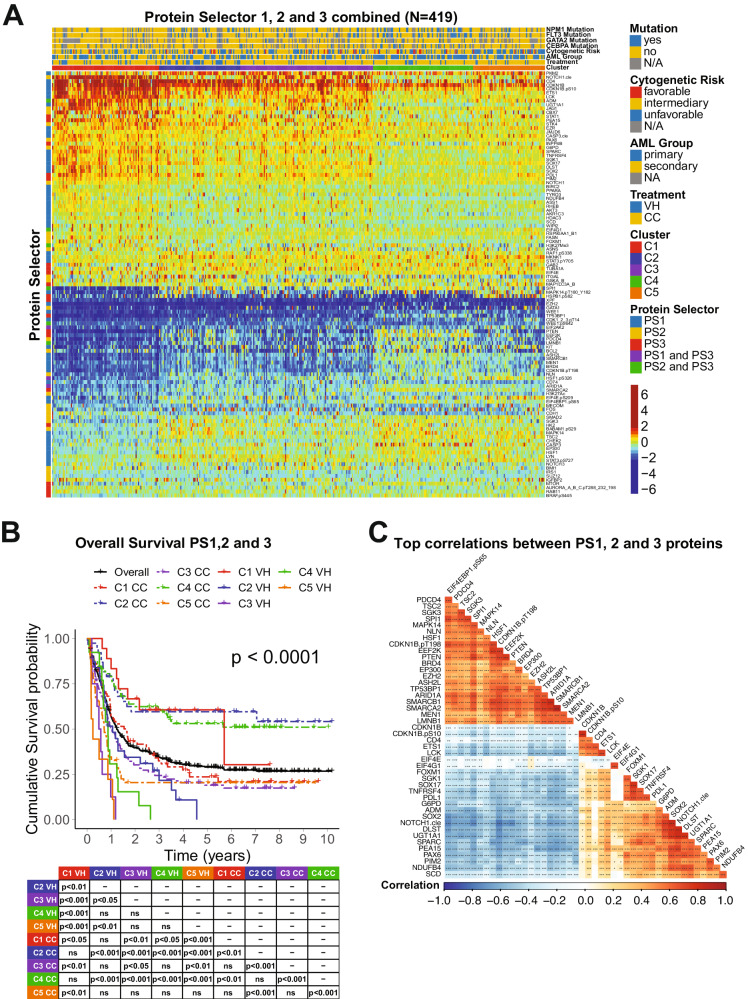
Integrated analysis of protein expression and clinical outcomes of patients clustered with PS1, PS2, and PS3. **A** Heatmap depicting the protein expression of all patients (*N* = 419). Annotations above the heatmap, starting closest to the heatmap, show the cluster membership and treatment modality, and then other previously recognized prognostic features (AML group, cytogenetic risk, and presence of complex karyotype and mutations). Legends are as described in Fig. 1. **B** Kaplan–Meier plots of Overall Survival and **C** Top correlations between all the PS proteins (*N* = 45). Squares represent the correlation between each protein are colored according to the degree of the linear correlation, which varies between (−1, 1) and follows a ‘blue’ (−1), ‘white’ (0), and ‘red’ (1) gradient, as shown in the color legend. Significant correlations are highlighted according to the following: ***p < 0.001, **p < 0.01, *p < 0.05, and blank = not significant.

The combination of the PS sets led to the generation of five clusters separated by the expression levels of 109 proteins as shown in Fig. 2A. C1 derived from PS1, C2 and C3 from PS2 (former PS2-C1 and PS2-C2), and C4 and C5 from PS3 (former PS3-C1 and PS3-C2). In Fig. 2B, the OS was better for C1 patients (red) treated with VH (solid) compared to CC (dashed) (MS = 68.5 mo. vs. 19.4 mo.). In contrast, both C2-CC (dashed blue) and C4-CC (dashed green) displayed MS > 120 mo., outperforming both C2-VH (solid blue), with a MS of 12.7 mo., and C4-VH (solid green), which has a MS of 10.4 mo. Moreover, although C3-CC (purple dashed) do better than C3-VH (purple solid) (MS of 12.2 mo. vs. 6.4 mo.), their OS are worse than the C2-CC and C4-CC populations. Finally, our PS system could not determine which treatment patients in cluster C5 (orange) should receive. Considering their poor outcomes in both VH (MS = 2.9 mo.) and CC (MS = 8.6 mo), it seems that this population might benefit from another treatment regimen (e.g., target-based therapies). Analysis of CRD for all PS sets showed a similar outcome pattern (Supplementary Fig. S1). Comparison of VH vs. CC for each cluster separately is shown in Supplementary Fig. S2.

To better assess the biological meaning of the PS analyses, we evaluated the correlation of the expression levels of the 109 prognostic proteins between each other. In Fig. 2C, the top most correlated proteins, defined as having a correlation coefficient > 0.60, are shown. Among the biological processes related to those proteins, the most common were ribosomal and transcriptional activity (10 proteins), histone modifiers (8 proteins), cell cycle and DNA damage response (7 proteins), cell metabolism (6 proteins). For an expanded view of these protein relationships, the complete correlation plot, together with protein networks of the PS proteins divided by functional group are shown in Supplementary Fig. S3. The correlation coefficients for all proteins, along with *p*-values of each comparison are shown in Supplementary Table S4. The stratification of all 109 proteins by biological process with their respective Protein Selector Set is shown in Supplementary Table S5.

### Clusters associations with demographic, clinical, and molecular features

We examined how the clusters differed considering demographic (age, gender, race), clinical (AML group and laboratory parameters), and molecular features (cytogenetics and mutation profiles), as shown in Table 1. There were significant differences in age distribution, as well as the frequency of many clinical variables (primary vs. secondary AML, white blood cell count, percentage of blasts and platelets number), cytogenetics (by risk group, simple vs. complex karyotype, or for specific events, such as −5/5q-, −7/7q- and inv16), and for several individual mutations (ASXL1, CEBPA, DNMT3A, EZH2, FLT3 [individually for ITD and D835, and in combination], NPM1, and TP53). An expanded table with all variables assessed is shown in Supplementary Table S6.

**Table 1:** Significant demographic, clinical, and molecular characteristics.

Variable	Overall	Patient cluster					
	*N* = 419^*a*^	C1, *N* = 91^*a*^	C2, *N* = 69^*a*^	C3, *N* = 113^*a*^	C4, *N* = 85^*a*^	C5, *N* = 61^*a*^	*p*-value^*b*^
**Age (years)**	58.1 (15.0)	61.1 (13.5)	60.7 (16.4)	58.3 (13.7)	53.8 (15.8)	56.5 (15.3)	**0.004**
**White Blood Cell count (K/uL)**	22.7 (41.9)	3.8 (7.0)	25.3 (34.8)	18.4 (40.4)	44.1 (57.2)	26.7 (43.8)	**<0.001**
**Blasts (%)**	32.0 (31.1)	10.4 (20.0)	26.7 (28.7)	26.8 (25.7)	58.7 (28.2)	41.5 (31.1)	**<0.001**
**Platelets (K/uL)**	69.5 (99.2)	69.9 (118.5)	101.6 (153.0)	66.8 (69.8)	40.9 (40.3)	77.4 (86.4)	**<0.001**
**Secondary AML**	46%	53%	51%	54%	27%	43%	**0.001**
**Unfavorable Cytogenetics**	37%	33%	38%	35%	24%	63%	**<0.001**
**Complex Karyotype**	28%	24%	26%	29%	16%	47%	**0.003**
**−5/5q-**	14%	10%	12%	12%	7.6%	32%	**0.001**
**−7/7q-**	15%	18%	12%	12%	2.5%	36%	**<0.001**
**Inv16**	5.4%	1.3%	14%	1.9%	10%	1.7%	**<0.001**
**ASLX1 Mutation**	18%	28%	27%	18%	10%	5.7%	**0.010**
**CEBPA Mutation**	13%	12%	3.8%	11%	26%	6.1%	**0.003**
**DNMT3 Mutation**	25%	19%	41%	19%	30%	18%	**0.016**
**EZH2 Mutation**	4.1%	6.0%	0%	7.0%	0%	7.1%	**0.034**
**FLT3 Mutation**	22%	5.2%	23%	19%	43%	19%	**<0.001**
**FLT3 D835 Mutation**	5.8%	0%	7.1%	2.1%	17%	3.7%	**<0.001**
**FLT3 ITD Mutation**	18%	5.2%	18%	16%	30%	19%	**0.001**
**NPM1 Mutation**	19%	5.5%	27%	13%	38%	15%	**<0.001**
**TP53 Mutation**	18%	19%	18%	15%	9.2%	36%	**0.005**

^a^Mean (SD), %;

^b^Kruskal–Wallis rank sum test; Fisher’s Exact Test for Count Data with simulated *p*-value (based on 10000 replicates).

Bold values indicate statistical significance *p* < 0.05.

Since many of these features with unbalanced distributions among the clusters are known to be prognostic, we wondered whether the cluster prognostic impact was just a reflection of these imbalances or if the clusters were independently predictive. Here, we generated KM plots to verify whether cluster membership is prognostic for OS and CRD when the population is filtered for specific variables (e.g., males only, secondary AML only, etc.). KM plots with *p*-values are shown in Supplementary Figs. S4 and S5. The prognostic impact of the five clusters was sustained for almost all the variables, including gender, all three age groups, all races, both primary and secondary AML, and major cytogenetic groupings (whether divided into three prognostic groups or for complex karyotypes). Since most individual cytogenetic and mutation events occur at a low frequency when the five clusters are subdivided by treatment modality (ten groups in total), the small sample sizes often preclude reaching statistical thresholds. However, similar trends (C1, C2, and C4, better than C3 and C5) were maintained for the majority, with exceptions noted for FLT3, IDH1, IDH2, JAK2, MLL, PTPN11, and TP53 mutations.

Next, we measured the prognostic value of the clusters and other variables using univariate (UV) and multivariate (MV) Cox proportional-hazards models (CoxPH) for both OS and CRD. In both analyses, clusters were condensed into three groups to avoid a large number of levels in a single variable, which might negatively influence the CoxPH models. Therefore, clusters with good prognosis (C1-VH, C2-CC, and C4-CC) were joined and renamed Group1; the ones with intermediate OS and CRD (C1-CC, C2-VH, C3-CC) were compacted into Group2; and finally, the remaining clusters, with poor prognosis, (C3-VH, C4-VH, C5-VH and C5-CC) were merged into Group3. As demonstrated in Table 2, all cluster groups were predictive of survival and remission in both the UV and MV models, reinforcing their prognostic value. Moreover, a few demographic (age, white race, and Asian race), clinical (secondary AML, blasts, Hbg, and serum B2M), cytogenetic (complex karyotype, −5/5q-, −7/7q-, t(8;21), Inv16, and Del12), and mutational (ASLX1, CEBPA, FLT3 [individually for ITD and D835, and in combination], IDH2, JAK2, MLL, NPM1, PTPN11, and TP53 mutations) features were also prognostic in the UV model for OS. However, only clusters, secondary AML, complex karyotype, Inv16, and IDH2 and PTPN11 mutations remained significant in the MV analysis. Regarding CRD, in the UV analysis clusters remained highly significant along with other characteristics (age, black race, AML group, complex karyotype, −5/5q-, Inv16, and FLT3, RUNX1, and TP53 mutations), with only clusters, black race, and complex karyotype, which remained significant in the MV model. Taken together, these findings corroborate the independent prognostic value of the PS protein signatures. An expanded table containing all variables evaluates in the UV model for both OS and CRD is shown in Supplementary Table S7.

**Table 2:** Significant univariate and multivariate Cox proportional-hazards of overall survival (OS) and complete remission duration (CRD).

Variable	Univariate OS (*N* = 419)			Multivariate OS (*N* = 419)			Univariate CRD (*N* = 274)			Multivariate CRD (*N* = 274)		
	HR^a^	95% CI^a^	*p*-value	HR^a^	95% CI^a^	*p*-value	HR^a^	95% CI^a^	*p*-value	HR^a^	95% CI^a^	*p*-value
**Cluster membership**												
Group1	1.00	—		1.00	—		1.00	—		1.00	—	
Group2	2.49	1.85, 3.37	**<0.001**	1.72	1.10, 2.68	**0.017**	2.22	1.44, 3.42	**<0.001**	1.90	1.16, 3.11	**0.011**
Group3	3.99	2.81, 5.68	**<0.001**	3.05	1.77, 5.26	**<0.001**	3.87	2.26, 6.62	**<0.001**	4.16	2.18, 7.96	**<0.001**
**Age (years)**	1.04	1.03, 1.05	**<0.001**	1.02	1.00, 1.03	**0.010**	1.04	1.02, 1.05	**<0.001**	1.03	1.01, 1.04	**0.003**
**White (race)**	1.49	1.11, 2.01	**0.008**	0.79	0.50, 1.23	0.29	1.09	0.72, 1.66	0.69			
**Asian (race)**	0.42	0.20, 0.90	**0.025**	0.48	0.19, 1.18	0.11	1.03	0.45, 2.34	0.95			
**Black (race)**	1.03	0.62, 1.70	0.91				1.88	1.01, 3.50	**0.046**	2.92	1.46, 5.85	**0.002**
**AML group**	2.84	2.24, 3.60	**<0.001**	1.48	1.00, 2.18	**0.049**	2.24	1.55, 3.23	**<0.001**	1.44	0.91, 2.27	0.12
**Blasts (%)**	1.00	0.99, 1.00	**0.014**				1.00	0.99, 1.01	0.82			
**Hemoglobin (g/dL)**	0.92	0.85, 0.98	**0.017**				0.93	0.83, 1.04	0.22			
**Serum B2M (ug/mL)**	1.13	1.05, 1.21	**0.001**				1.07	0.95, 1.21	0.26			
**Complex Karyotype**	2.51	1.95, 3.22	**<0.001**	1.66	1.03, 2.66	**0.037**	2.29	1.53, 3.44	**<0.001**	2.67	1.50, 4.78	**<0.001**
**−5/5q-**	2.55	1.86, 3.49	**<0.001**	1.19	0.54, 2.63	0.67	2.37	1.39, 4.04	**0.001**	0.71	0.28, 1.83	0.48
**−7/7q-**	2.04	1.50, 2.78	**<0.001**	0.88	0.55, 1.40	0.58	1.36	0.71, 2.60	0.35			
**t(8;21)**	0.45	0.23, 0.87	**0.017**	0.36	0.13, 1.02	0.055	0.82	0.40, 1.69	0.59			
**Inv16**	0.16	0.06, 0.44	**<0.001**	0.22	0.05, 0.95	**0.042**	0.29	0.11, 0.80	**0.016**	0.41	0.12, 1.33	0.14
**Del12**	2.44	1.29, 4.59	**0.006**	0.65	0.26, 1.62	0.35	2.01	0.64, 6.34	0.23			
**ASLX1 Mutation**	1.43	1.01, 2.02	**0.042**	1.34	0.87, 2.06	0.18	1.30	0.74, 2.28	0.37			
**CEBPA Mutation**	0.56	0.35, 0.87	**0.011**	0.84	0.48, 1.47	0.54	0.60	0.31, 1.15	0.12			
**FLT3 Mutation**	0.59	0.42, 0.83	**0.002**	1.31	0.82, 2.09	0.26	0.60	0.36, 0.98	**0.041**	0.94	0.54, 1.65	0.84
**FLT3 D835 Mutation**	0.51	0.27, 0.97	**0.039**				0.61	0.27, 1.39	0.24			
**FLT3 ITD Mutation**	0.62	0.43, 0.90	**0.012**				0.65	0.37, 1.12	0.12			
**IDH2 Mutation**	0.61	0.41, 0.90	**0.014**	0.52	0.30, 0.90	**0.019**	0.73	0.41, 1.28	0.27			
**JAK2 Mutation**	1.90	1.03, 3.48	**0.038**	1.01	0.49, 2.07	0.97	1.26	0.40, 3.99	0.69			
**MLL Mutation**	1.95	1.16, 3.30	**0.012**				2.30	0.90, 5.91	0.083			
**NPM1 Mutation**	0.54	0.37, 0.78	**0.001**	0.65	0.37, 1.14	0.13	0.61	0.37, 1.01	0.053			
**PTPN11 Mutation**	1.82	1.20, 2.75	**0.004**	2.02	1.21, 3.36	**0.007**	1.74	0.90, 3.36	0.10			
**RUNX1 Mutation**	1.21	0.83, 1.75	0.32				2.18	1.24, 3.84	**0.007**			
**TP53 Mutation**	3.40	2.54, 4.57	**<0.001**	1.42	0.71, 2.84	0.32	2.82	1.74, 4.57	**<0.001**	0.87	0.40, 1.93	0.74

^a^*HR* hazard ratio.

*CI* confidence interval.

Bold values indicate statistical significance *p* < 0.05.

### Development of a protein classifier (PC) for treatment recommendation

**Fig. 3:**
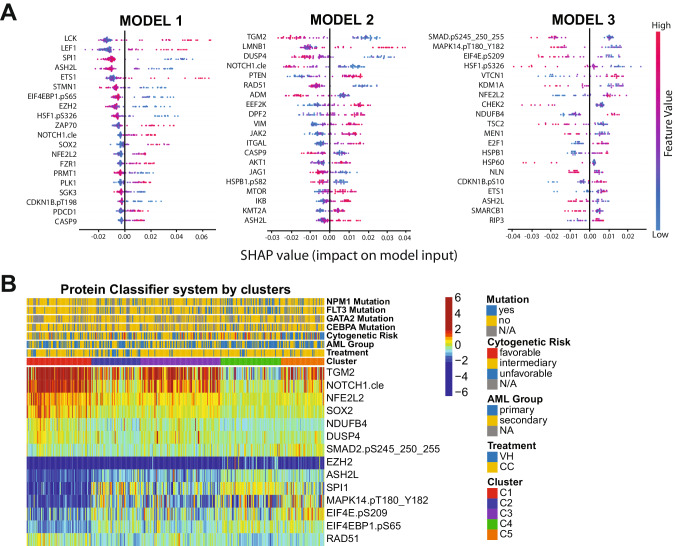
Development of protein Classifier (PC) models using random forest machine learning approach. **A** Top predictive proteins for each of the three models developed (y-axis), for all test-set patients, according to each protein’s calculated SHAP value (x-axis). Color legend indicates the value of a given prognostic protein’s expression relative to other expression values of that protein among test-set patients. (pink = high predictive value; blue = low predictive value). **B** Heatmap showing the expression levels of the proteins selected for the protein classifier by cluster and treatment modality. Annotations and legends are as described in Figs. 1–3.

Although the PS system can efficiently separate patients who should receive VH from those who would do better with CC, it is not feasible to measure more than 100 different proteins in the clinical setting. The number of proteins required to be assessed is excessive and poses a major cost-benefit challenge for the application of the method. Instead, the identification of a few proteins that can be measured using a Clinical Laboratory Improvement Amendments (CLIA)-certified test to accurately assign an individual patient to a specific protein expression profile is practical. Therefore, we designed a classification algorithm using the random forest machine learning technique entitled Protein Classifier (PC). The system can identify the most predictive proteins for treatment recommendation, based on previously developed cluster memberships and protein expression data. In other words, we recommended VH treatment for patients belonging to cluster C1 (*N* = 91); CC therapy for patients in clusters C2, C3, and C4 (*N* = 267); and neither VH nor CC for the C5 patient population (*N* = 61). The system was developed with the goal of defining clusters using three different models sequentially:(1) Define C1 patients (*N* = 91); (2) Distinguish C2 and C4 groups (*N* = 154) from the C3 and C5 populations (*N* = 174); and (3) Separate C3 (*N* = 113) from C5 (*N* = 61) patients. In Fig. 3A, the top predictive proteins are visualized together with their respective SHAP values. The first step of the PC system identified the six most predictive proteins for C1: SPI1, ASH2L, EIF4EBP1.pS65, EZH2, NFE2L2 and SOX2 (C-index: 0.951). Thus, according to our previous OS and CRD analyses, patients with this protein signature should receive VH therapy. In the second step of the PC system, TGM2, NOTCH1.cle, DUSP4, and RAD51 were the best proteins to differentiate C2 + C4 from C3 + C5 (C-index: 0.903). Of note, distinguishing C3 from C2 and C4 is necessary, because although both patient groups should receive CC, the OS and CRD for C3 is much lower, so this patient group may benefit from additional therapy (e.g., CC and stem cell transplant in first remission), whereas C2 and C4 seem to do well with CC alone. Finally, SMAD2.pS245_250_255, MAPK14.pT180_Y182, EIF4E.pS209, and NDUFB4 were identified as the best proteins to segregate C3 and C5, defining the last step of our system (C-index:0.923). The expression of all proteins in the PC system by cluster is shown in Fig. 3B. Importantly, the C-index, a measure of individual patient discriminatory power, of all models in our PC system is above 0.90, demonstrating that it robustly predicts optimal therapy choice (a C-index higher than 0.7 is considered predictive, while a measure of 1 would indicate perfection). Moreover, by considering all three models working together, we predicted that 87.3% of patients would receive the correct therapy, and only a small fraction of 5.5% would be misassigned. The proportion of patients in the C5 group who could be assigned to either CC or VH, instead of being defined as ‘undetermined’, was 7.1%. Overall sensitivity, specificity, and accuracy were 84.2%, 79.6%, and 82.8%, respectively. The predictive calculations for the PC model are presented in Supplementary Table S8. Therefore, the development of a kit that determines the expression of the aforementioned 14 proteins would be useful and financially feasible for triaging patients and guiding the recommendation for VH or CC.

### Patients with the worst outcomes have a unique and targetable protein signature

**Fig. 4:**
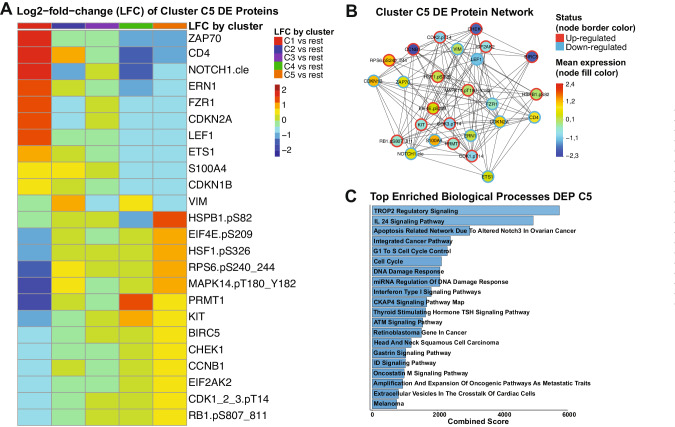
Differential expression (DE) analysis of cluster C5 patients. **A** Heatmap demonstrating the mean Log2-fold change (LFC) values of every assessed comparison, regarding the 24 proteins that are differentially expressed in cluster C5 patients. Heatmap annotation refer to the comparison as established by the legend on the right. The mean LFC ranges according to the values and color legend on the right **B** Protein network of DE proteins of Cluster C5. Network nodes are colored according to the mean expression value, ranging from above normal (red) to normal (yellow-green-aqua) to below normal (dark blue) as shown in the color legend (right). Node borders are colored according to the Differential Expression status of protein (up-regulated or down-regulated), following the colors shown in the legend (right). **C** Top twenty enriched biological processes related to the differentially expressed proteins of cluster C5. Y-axis shows the name of each ontology and x-axis shows the combined scores of each process. Bargraphs are colored according to a blue gradient, where darker blue corresponds to lower values and lighter blue to higher values.

Since our PS system was unable to recommend either VH or CC for cluster C5 patients, we decided to determine the most associated signaling pathways within this population. We identified 24 proteins among the 411 in our database which in combination form a unique expression profile in C5 patients, compared to all the other clusters. In Fig. 4A, the Log2-fold-change (LFC) values of each each cluster against all the others is shown for each differentially expressed (DE) protein of cluster C5. Proteins from ZAP70 until VIM have lower LFC values and, thus, were considered down-regulated in C5, whereas the proteins from HSPB1.pS82 to RB1.pS807_811 were classified as up-regulated since their LFC values are higher in C5 compared to the others. A table with FDR-adjusted *p*-values and LFC values comparing each cluster against all the others is shown in Supplementary Table S9. To better visualize connections of the C5 DE proteins with each other, we generated a protein network, annotating the mean expression values of each one compared to normal bone marrow (node fill color), and whether the protein is up- or down-regulated (node border). Importantly, although a few proteins are up-regulated compared to the other clusters, their mean expression is below the levels of normal bone marrow (e.g., CHEK1, BIRC5, CCNB1). A table with all the DE proteins and their directionality (up- or down-regulated), stratified by cluster is in Supplementary Table S10. Volcano plots highlighting the directionality of DE proteins for every cluster are shown in Supplementary Fig. S6.

To gain insights about the biological meaning of our data, we performed pathway enrichment analysis of the 24 DE proteins. As shown in Fig. 4C, processes with the highest combined scores (i.e., lowest *p*-value and highest odds-ratio) were most significantly correlated to these proteins. Most of those were related to cell cycle regulation and the DNA damage response (DDR), but specific pathways were also enriched (e.g., TROP2, IL-24, and CKAP4 signaling). The complete table with all the processes and their combined scores, along with adjusted *p*-values and odds ratios can be found in Supplementary Table S11. Altogether, even though we were unable to recommend a specific treatment for C5 patients, our DE analysis revealed potential druggable signaling pathways that could be useful for developing target-based therapies.

## Discussion

Proteomic profiling studies, developed previously by our group using the RPPA methodology, identified proteomic signatures that create a novel proteomic-based categorization system that was prognostic in leukemia [20–24]. In this study, we applied a similar proteomic-based strategy to a large cohort of AML patients and identified unique and recurrent protein signatures that could be useful for recommending either HMA + VEN or Conventional Chemotherapy treatments. We identified five protein signatures: one (22% of cases) that should optimally receive VH, three (63%) in which CC is superior, and the last one (15% of cases) for which neither VH nor CC was preferable (especially after removing favorable cytogenetics patients that are known to do well with CC). However, for this group, the PS system and differential expression (DE) analysis identified major signaling hubs connected to the protein profile of those patients, providing insights for possible target-based therapies in the remaining 15% (61/419) of patients. A therapy triaging system, optimized by the evaluation of protein expression, would have reassigned 30% of cases (125/419), with great impact on the five-year survival and remission rates. Considering the adequate treatment for cluster C1 as VH and the best treatment for clusters C2, C3, and C4 as CC, if the patients were triaged by the PS system, the overall five-year survival rate would be predicted to increase from 30% (126 patients) to 43% (181 patients), a 43% increase in survival. The proportion in remission at the five-year timepoint jumps from 52 to 63%, an increase of 21%. Considering the US annual incidence of 20,000 newly diagnosed AML cases, our proteomic triaging system using proteomics-optimized therapy selection could result in 2600 more cures using existing therapies (full calculations are in Supplementary Table S12). Of note, centralized proteomic assessment as part of a clinical trial or for routine testing is feasible, since protein levels, including phosphorylation, have been shown by us to remain stable for up to 72 h if the samples are refrigerated, even if they are shipped across long distances [41].

Furthermore, most demographic, clinical, and molecular characteristics were not exclusively associated with a single protein signature, although some showed biased distribution among the five clusters. However, cluster membership by treatment was an independent prognostic factor for OS, and to a lesser extent, for CRD, in both univariate and univariate models. Therefore, proteomic analysis provides new prognostic information regarding responses that are not available for known prognostic factors. Since most of the assessed molecular and cytogenetic features were equally common in all protein signatures, it seems that several distinct associations of independent molecular events may lead to a similar proteomic signature, and a similar corresponding pathophysiology, which is being captured by our PS system.

Interestingly, the PS system was also able to identify recurrent biological processes relevant to patient prognosis. Since the three selector sets (PS1, PS2 and PS3) were sequentially derived from patient subsets of a larger population, it is not surprising that most proteins (*N* = 100) were unique to a single selector set, while only three (ARID1A, EIF2AK2 and HSF1.pS326) were common to PS1 and PS2, and just six showed overlap between PS2 and PS3 (H3K27Me3, WEE1.pS642, EIF4G1, SP1, ADM and LMNB1). However, while the proteins in each selector set tended to be unique, the cellular functions involved were recurrent in all of them. Among the 15 functionally related groups of proteins defined by us, 10 showed substantial convergence between the PS sets: histone modifiers, cell cycle and DDR, ribosomal and transcriptional activity, cell metabolism, proliferative pathways, cell adhesion and cytoskeleton regulation, apoptosis, signaling regulation, heatshock proteins, and cell differentiation (see Supplementary Table S4). Importantly, considering the distinct expression pattern of all five protein signatures, it seems that each cluster has its own biases regarding those functional groups. This suggests that these biological processes are not only are related to prognosis but also might represent a therapeutic opportunity worth exploring to improve patient response.

Finally, our PS system identified a particular patient population for whom neither VH nor CC was recommended as the main therapy. By exploring the protein expression profiles of those patients, we identified a small number of differentially expressed proteins that were up- or down-regulated in comparison to the other clusters. We also correlated those proteins with ontologies related to cell cycle and DDR and other more specific pathways. Furthermore, two proteins caught our attention: RPS6.pS240_244, which is up-regulated in C5 and has higher expression levels compared to normal bone marrow (NBM), and FZR1, which is down-regulated has low expression compared to NBM. RPS6 composes part of the 40 S unit of the ribosome and is a downstream target of several proliferative pathways, such as PI3K/AKT/mTORC1 and MAPK/ERK axis, both of which converge to activate S6K, responsible for the phosphorylation of RPS6 at S240/S244(refs. [42, 43]). Phospho-RSP6 increases translation of specific mRNAs, ultimately inducing cell growth, and its overexpression has been observed in many cancer types, including AML [43–45]. In contrast, loss of FZR1, a cell cycle and DDR regulator, increases the sensitivity to genotoxic agents in B-cell acute leukemia and also contributes to the selection therapy-resistant subclones [46]. Interestingly, phosphorylation of FZR1 by ERK facilitates melanomagenesis, and loss of FZR1 cooperates with AKT to transform primary melanocytes [47]. Therefore, high RPS6.pS240_244 and low FZR1 might actually be directly correlated to PI3K/AKT/mTORC1 and/or MAPK/ERK activation in C5 patients, and inhibition of those pathways with FDA-approved drugs (e.g., sirolimus, capivasertib, sorafenib) could potentially improve outcomes.

In summary, we developed a proteomic-based triaging system to recommend either VH or CC for patients with AML. We predict that by applying our proteomic approach both overall survival and complete remission duration of AML patients will experience a significant increase, resulting in 2 600 more cures per year in the USA using existing therapies. Moreover, we identified potential therapeutic targets to improve the therapy of patients who would not be predicted to benefit from either VH or CC treatment regimens.

### Supplementary information

Supplementary files

## Data Availability

Patient datasets and code scripts are freely available at https://github.com/escmagalhaes/23-LEU-1445 and will be transferred to http://www.leukemiaatlas.org upon publication.
