# Supplementary material for: Proteomics for optimizing therapy in acute myeloid leukemia: venetoclax plus hypomethylating agents versus conventional chemotherapy
Source: Leukemia. 2024 Mar 26;38(5):1046–56. doi: 10.1038/s41375-024-02208-8 (PMC11073970; doi:10.1038/s41375-024-02208-8)
Supplement: Supplementary file 1 — Supplementary files [file 41375_2024_2208_MOESM1_ESM.pdf]

A

Remission Duration PS1

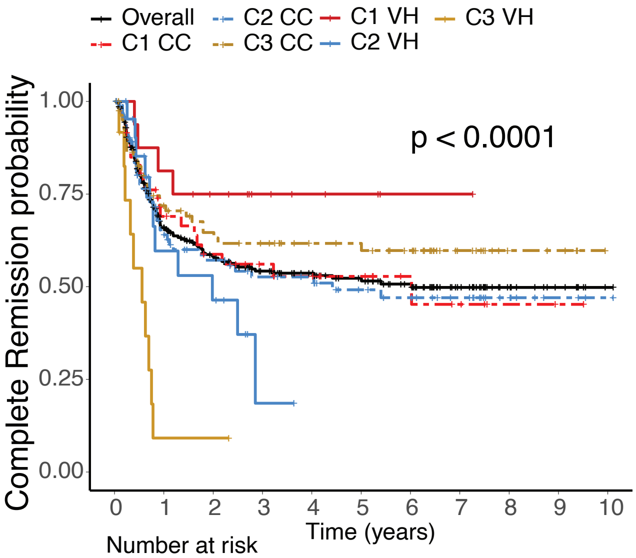

Number at risk

Time (years)	0	1	2	3	4	5	6	7	8	9	10
Overall	274	153	124	98	83	70	56	35	15	8	1
C1 CC	47	28	22	17	14	11	7	5	2	1	0
C2 CC	94	50	40	34	32	24	21	16	8	3	1
C3 CC	82	52	44	40	33	32	27	13	5	4	0
C1 VH	16	13	10	6	4	3	1	1	0	0	0
C2 VH	23	9	7	1	0	0	0	0	0	0	0
C3 VH	12	1	1	0	0	0	0	0	0	0	0

	C1 VH	C2 VH	C3 VH	C1 CC	C2 CC
C2 VH	ns	–	–	–	–
C3 VH	p<0.001	p<0.01	–	–	–
C1 CC	ns	ns	p<0.001	–	–
C2 CC	ns	ns	p<0.001	ns	–
C3 CC	ns	ns	p<0.001	ns	ns

B

Remission Duration PS2

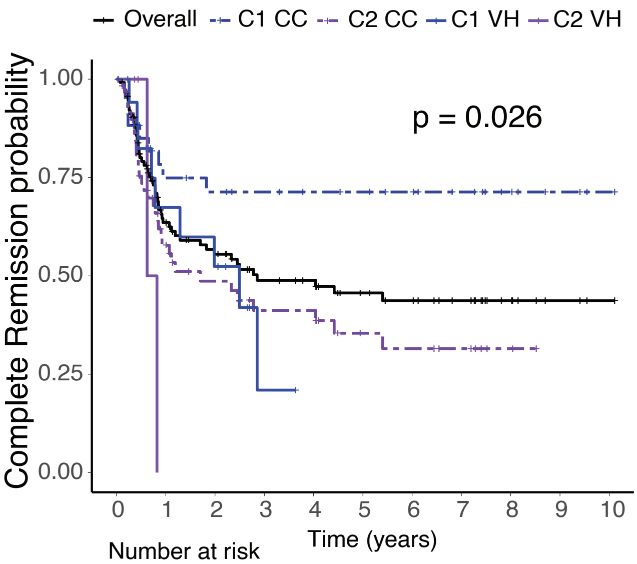

Number at risk

Time (years)	0	1	2	3	4	5	6	7	8	9	10
Overall	117	59	47	35	32	24	21	16	8	3	1
C1 CC	34	22	20	18	16	15	13	10	6	3	1
C2 CC	60	28	20	16	16	9	8	6	2	0	0
C1 VH	19	9	7	1	0	0	0	0	0	0	0
C2 VH	4	0	0	0	0	0	0	0	0	0	0

	C1 VH	C2 VH	C1 CC
C2 VH	ns	–	–
C1 CC	ns	ns	–
C2 CC	ns	ns	p<0.05

C

Remission Duration PS3

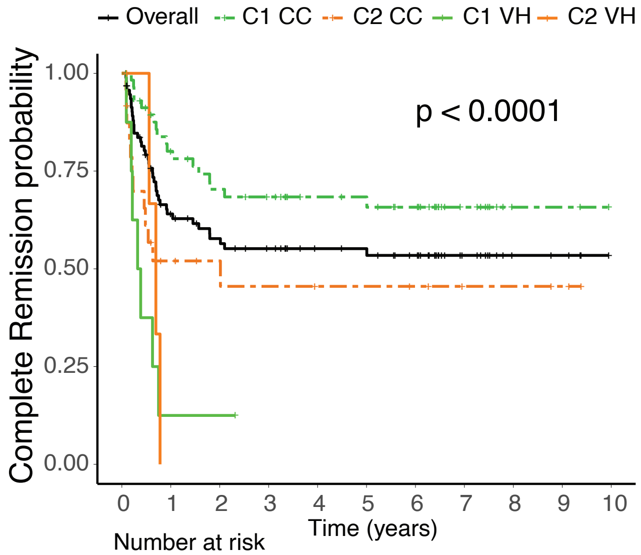

Number at risk

Time (years)	0	1	2	3	4	5	6	7	8	9	10
Overall	94	53	45	40	33	32	27	13	5	4	0
C1 CC	58	42	36	33	27	26	22	10	2	2	0
C2 CC	24	10	8	7	6	6	5	3	3	2	0
C1 VH	8	1	1	0	0	0	0	0	0	0	0
C2 VH	4	0	0	0	0	0	0	0	0	0	0

	C1 VH	C2 VH	C1 CC
C2 VH	ns	–	–
C1 CC	p<0.001	p<0.01	–
C2 CC	ns	ns	p<0.05

D

Remission Duration PS1,2 and 3

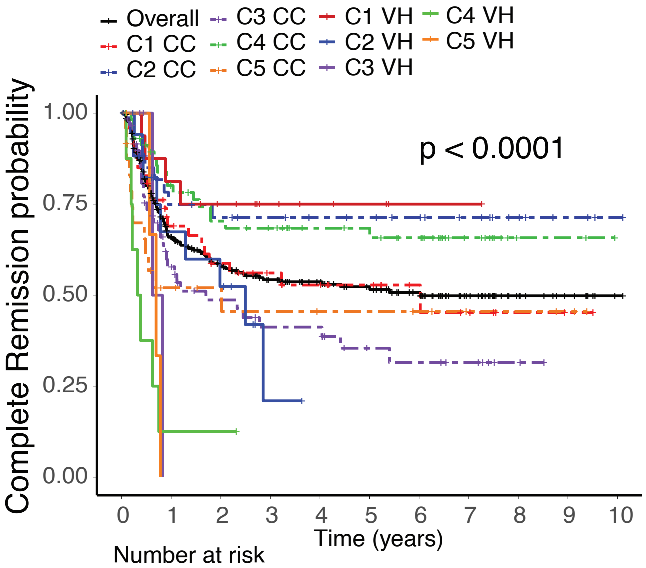

Number at risk

Time (years)	0	1	2	3	4	5	6	7	8	9	10
Overall	274	153	124	98	83	70	56	35	15	8	1
C1 CC	47	28	22	17	14	11	7	5	2	1	0
C2 CC	34	22	20	18	16	15	13	10	6	3	1
C3 CC	60	28	20	16	16	9	8	6	2	0	0
C4 CC	58	42	36	33	27	26	22	10	2	2	0
C5 CC	24	10	8	7	6	6	5	3	3	2	0
C1 VH	16	13	10	6	4	3	1	1	0	0	0
C2 VH	19	9	7	1	0	0	0	0	0	0	0
C3 VH	4	0	0	0	0	0	0	0	0	0	0
C4 VH	8	1	1	0	0	0	0	0	0	0	0
C5 VH	4	0	0	0	0	0	0	0	0	0	0

	C1 VH	C2 VH	C3 VH	C4 VH	C5 VH	C1 CC	C2 CC	C3 CC	C4 CC
C2 VH	ns	–	–	–	–	–	–	–	–
C3 VH	ns	ns	–	–	–	–	–	–	–
C4 VH	p<0.01	p<0.05	ns	–	–	–	–	–	–
C5 VH	p<0.05	ns	ns	ns	–	–	–	–	–
C1 CC	ns	ns	ns	p<0.01	ns	–	–	–	–
C2 CC	ns	ns	ns	p<0.01	p<0.05	ns	–	–	–
C3 CC	ns	ns	ns	p<0.01	ns	ns	p<0.05	–	–
C4 CC	ns	ns	ns	p<0.001	p<0.01	ns	ns	p<0.01	–
C5 CC	ns	ns	ns	ns	ns	ns	ns	ns	p<0.05

Supplementary Figure S1. Complete remission duration of PS sets by cluster

(A) Complete Remission Duration of PS1 (N=419), **(B)** PS2 (N=182), **(C)** PS3 (N=146), and **(D)** integrated PS1, 2 and 3 (N=419), separated by cluster and treatment modality (VH=solid line, CC=dashed line; PS1-C1=red, PS1-C2=light blue, PS1-C3=yellow, PS2-C1=blue, PS2-C2=purple, PS3-C1=orange, PS3-C2=green, C1=red, C2=blue, C3=purple, C4=green and C5=orange).

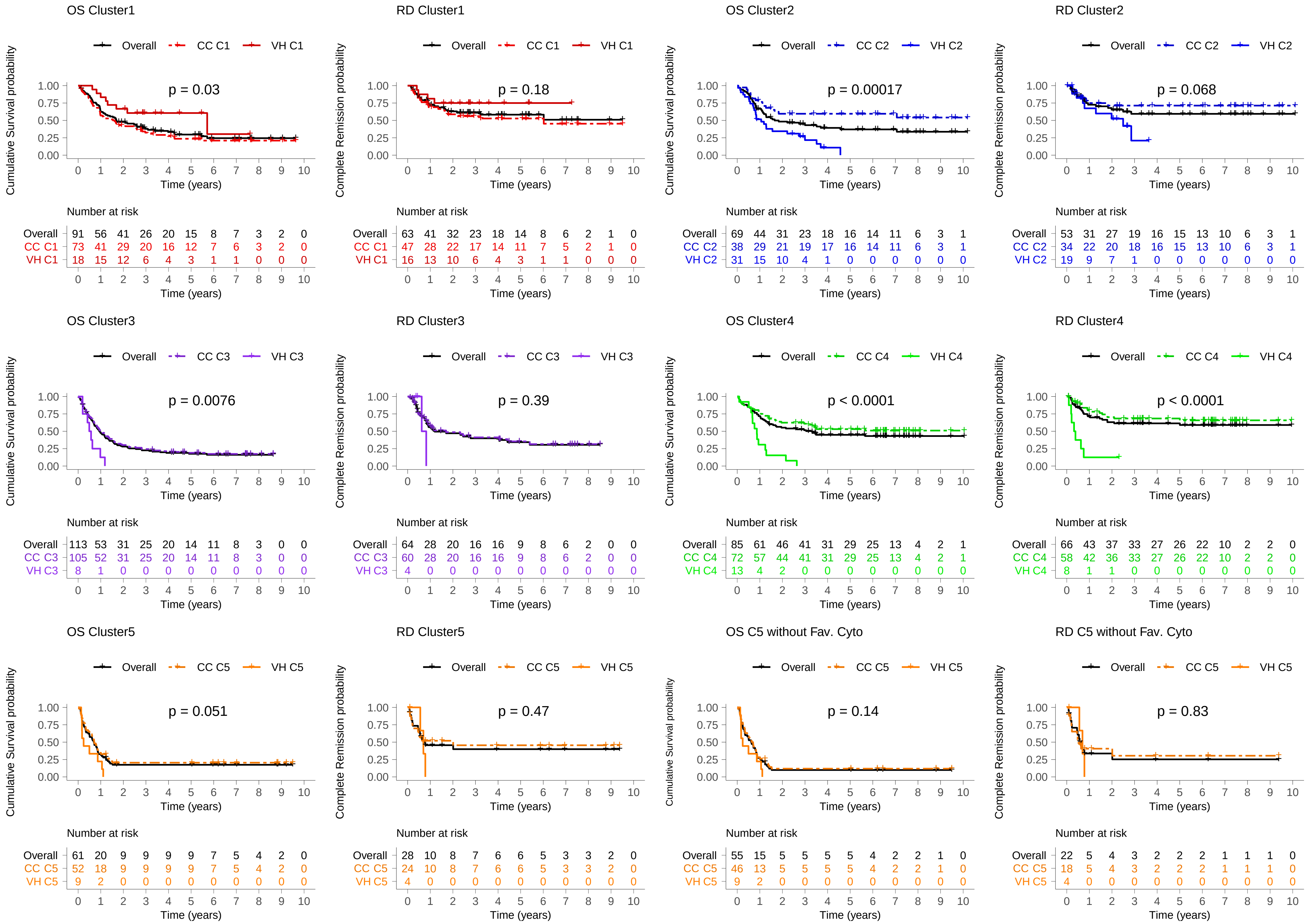
OS Cluster1
Overall CC C1 VH C1
p = 0.03
Cumulative Survival probability
Time (years)
Number at risk
Overall 91 56 41 26 20 15 8 7 3 2 0
CC C1 73 41 29 20 16 12 7 6 3 2 0
VH C1 18 15 12 6 4 3 1 1 0 0 0
RD Cluster1
Overall CC C1 VH C1
p = 0.18
Complete Remission probability
Time (years)
Number at risk
Overall 63 41 32 23 18 14 8 6 2 1 0
CC C1 47 28 22 17 14 11 7 5 2 1 0
VH C1 16 13 10 6 4 3 1 1 0 0 0
OS Cluster2
Overall CC C2 VH C2
p = 0.00017
Cumulative Survival probability
Time (years)
Number at risk
Overall 69 44 31 23 18 16 14 11 6 3 1
CC C2 38 29 21 19 17 16 14 11 6 3 1
VH C2 31 15 10 4 1 0 0 0 0 0 0
RD Cluster2
Overall CC C2 VH C2
p = 0.068
Complete Remission probability
Time (years)
Number at risk
Overall 53 31 27 19 16 15 13 10 6 3 1
CC C2 34 22 20 18 16 15 13 10 6 3 1
VH C2 19 9 7 1 0 0 0 0 0 0 0
OS Cluster3
Overall CC C3 VH C3
p = 0.0076
Cumulative Survival probability
Time (years)
Number at risk
Overall 113 53 31 25 20 14 11 8 3 0 0
CC C3 105 52 31 25 20 14 11 8 3 0 0
VH C3 8 1 0 0 0 0 0 0 0 0 0
RD Cluster3
Overall CC C3 VH C3
p = 0.39
Complete Remission probability
Time (years)
Number at risk
Overall 64 28 20 16 16 9 8 6 2 0 0
CC C3 60 28 20 16 16 9 8 6 2 0 0
VH C3 4 0 0 0 0 0 0 0 0 0 0
OS Cluster4
Overall CC C4 VH C4
p < 0.0001
Cumulative Survival probability
Time (years)
Number at risk
Overall 85 61 46 41 31 29 25 13 4 2 1
CC C4 72 57 44 41 31 29 25 13 4 2 1
VH C4 13 4 2 0 0 0 0 0 0 0 0
RD Cluster4
Overall CC C4 VH C4
p < 0.0001
Complete Remission probability
Time (years)
Number at risk
Overall 66 43 37 33 27 26 22 10 2 2 0
CC C4 58 42 36 33 27 26 22 10 2 2 0
VH C4 8 1 1 0 0 0 0 0 0 0 0
OS Cluster5
Overall CC C5 VH C5
p = 0.051
Cumulative Survival probability
Time (years)
Number at risk
Overall 61 20 9 9 9 9 7 5 4 2 0
CC C5 52 18 9 9 9 9 7 5 4 2 0
VH C5 9 2 0 0 0 0 0 0 0 0 0
RD Cluster5
Overall CC C5 VH C5
p = 0.47
Complete Remission probability
Time (years)
Number at risk
Overall 28 10 8 7 6 6 5 3 3 2 0
CC C5 24 10 8 7 6 6 5 3 3 2 0
VH C5 4 0 0 0 0 0 0 0 0 0 0
OS C5 without Fav. Cyto
Overall CC C5 VH C5
p = 0.14
Cumulative Survival probability
Time (years)
Number at risk
Overall 55 15 5 5 5 5 4 2 2 1 0
CC C5 46 13 5 5 5 5 4 2 2 1 0
VH C5 9 2 0 0 0 0 0 0 0 0 0
RD C5 without Fav. Cyto
Overall CC C5 VH C5
p = 0.83
Complete Remission probability
Time (years)
Number at risk
Overall 22 5 4 3 2 2 2 1 1 1 0
CC C5 18 5 4 3 2 2 2 1 1 1 0
VH C5 4 0 0 0 0 0 0 0 0 0 0

Supplementary Figure S2. Overall survival and complete remission duration by cluster

Kaplan-Meier plots of overall survival and complete remission duration separated by each individual cluster (VH=solid line, CC=dashed line; C1=red, C2=blue, C3=purple, C4=green and C5=orange). LogRank tests were used to compare the groups. For C5 (orange), two extra plots are shown without the inclusion of five favorable cytogenetic cases.

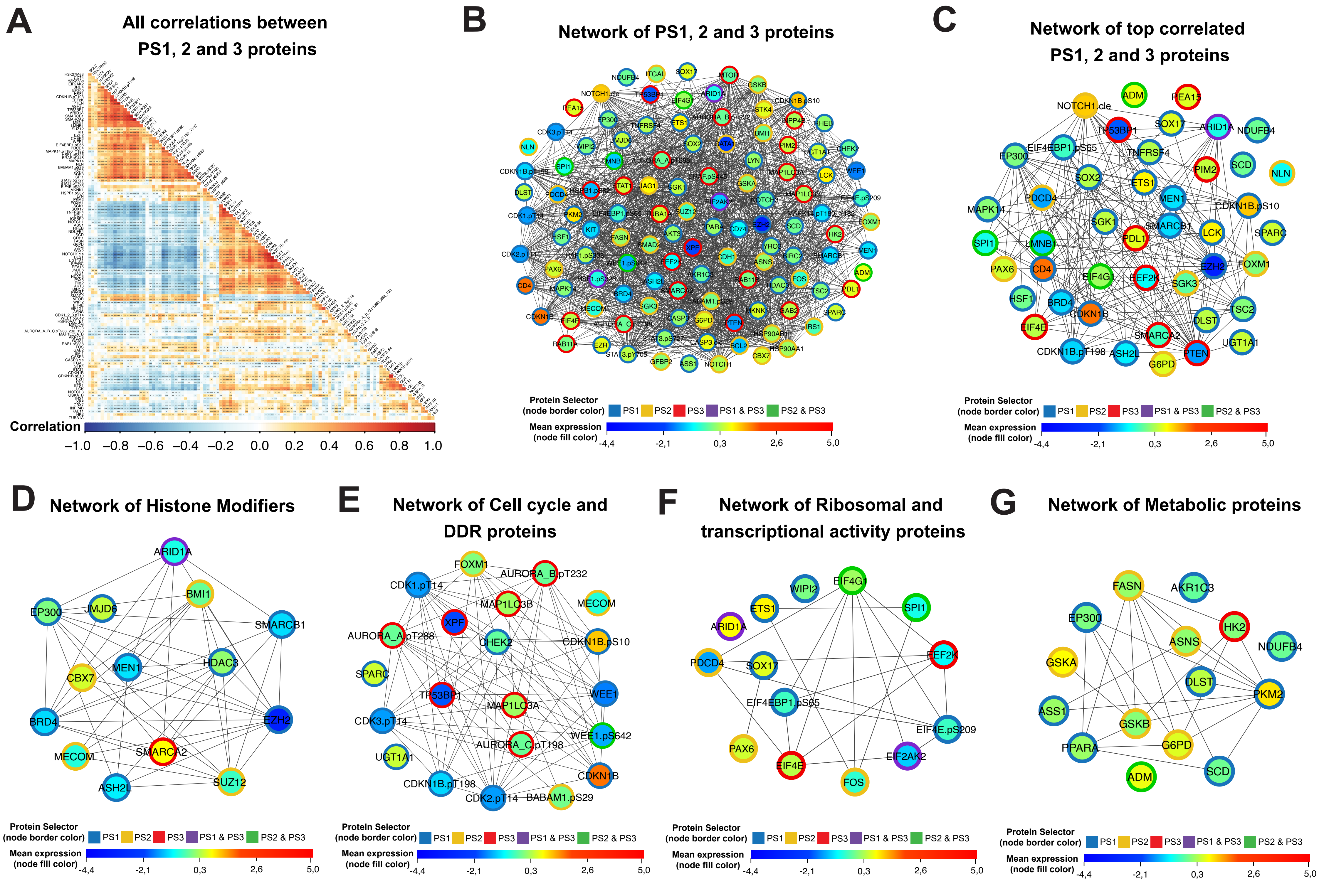
A
All correlations between
PS1, 2 and 3 proteins
Correlation
−1.0 −0.8 −0.6 −0.4 −0.2 0.0 0.2 0.4 0.6 0.8 1.0
B
Network of PS1, 2 and 3 proteins
Protein Selector
(node border color)
PS1 PS2 PS3 PS1 & PS3 PS2 & PS3
Mean expression
(node fill color)
-4,4 -2,1 0,3 2,6 5,0
C
Network of top correlated
PS1, 2 and 3 proteins
NOTCH1.cle
ADM
PEA15
TP53BP1
SOX17
ARID1A
NDUFB4
EP300
EIF4EBP1.pS65
TNFRSF4
SCD
NLN
PIM2
SOX2
ETS1
PDCD4
MEN1
CDKN1B.pS10
MAPK14
SGK1
SMARCB1
SPARC
SPI1
LMNB1
PDL1
LCK
PAX6
CD4
EIF4G1
EEF2K
SGK3
EZH2
FOXM1
HSF1
BRD4
CDKN1B
DLST
TSC2
EIF4E
SMARCA2
UGT1A1
CDKN1B.pT198
ASH2L
PTEN
G6PD
Protein Selector
(node border color)
PS1 PS2 PS3 PS1 & PS3 PS2 & PS3
Mean expression
(node fill color)
-4,4 -2,1 0,3 2,6 5,0
D
Network of Histone Modifiers
ARID1A
BMI1
EP300
JMJD6
SMARCB1
MEN1
HDAC3
CBX7
BRD4
EZH2
MECOM
SMARCA2
ASH2L
SUZ12
Protein Selector
(node border color)
PS1 PS2 PS3 PS1 & PS3 PS2 & PS3
Mean expression
(node fill color)
-4,4 -2,1 0,3 2,6 5,0
E
Network of Cell cycle and
DDR proteins
FOXM1
AURORA_B.pT232
CDK1.pT14
MAP1LC3B
MECOM
XPF
AURORA_A.pT288
CHEK2
CDKN1B.pS10
SPARC
WEE1
TP53BP1
MAP1LC3A
CDK3.pT14
WEE1.pS642
AURORA_C.pT198
UGT1A1
CDKN1B
CDKN1B.pT198
CDK2.pT14
BABAM1.pS29
Protein Selector
(node border color)
PS1 PS2 PS3 PS1 & PS3 PS2 & PS3
Mean expression
(node fill color)
-4,4 -2,1 0,3 2,6 5,0
F
Network of Ribosomal and
transcriptional activity proteins
EIF4G1
WIPI2
ETS1
SPI1
ARID1A
EEF2K
PDCD4
SOX17
EIF4EBP1.pS65
EIF4E.pS209
PAX6
EIF4E
EIF2AK2
FOS
Protein Selector
(node border color)
PS1 PS2 PS3 PS1 & PS3 PS2 & PS3
Mean expression
(node fill color)
-4,4 -2,1 0,3 2,6 5,0
G
Network of Metabolic proteins
FASN
AKR1C3
EP300
HK2
ASNS
NDUFB4
GSKA
DLST
PKM2
ASS1
GSKB
PPARA
G6PD
ADM
SCD
Protein Selector
(node border color)
PS1 PS2 PS3 PS1 & PS3 PS2 & PS3
Mean expression
(node fill color)
-4,4 -2,1 0,3 2,6 5,0

Supplementary Figure S3. Expanded view of protein-protein connections within PS selector sets

(A) Expanded view of correlation plot between all the 109 PS proteins. Squares represent the correlation between each protein are colored according to the degree of the linear correlation, which varies between (-1, 1) and follows a 'blue' (-1), 'white' (0) and 'red' (1) gradient, as shown in the color legend. Significant correlations are highlighted according to the following: ***$p < 0.001$, **$p < 0.01$, *$p < 0.05$, and blank = not significant. Protein networks of **(B)** all PS proteins, **(C)** the top correlated PS proteins, **(D)** Histone modifiers, **(E)** Cell cycle and DDR proteins, **(F)** Ribosomal and transcriptional activity regulation proteins and **(G)** Metabolic proteins. Network nodes are colored according to the mean expression value, ranging from above normal (red) to normal (yellow-green-aqua) to below normal (dark blue) as shown in the color legend (bottom of each panel). Node borders are colored according to the PS of which the protein belong, following the colors shown in the legend (bottom of each panel).

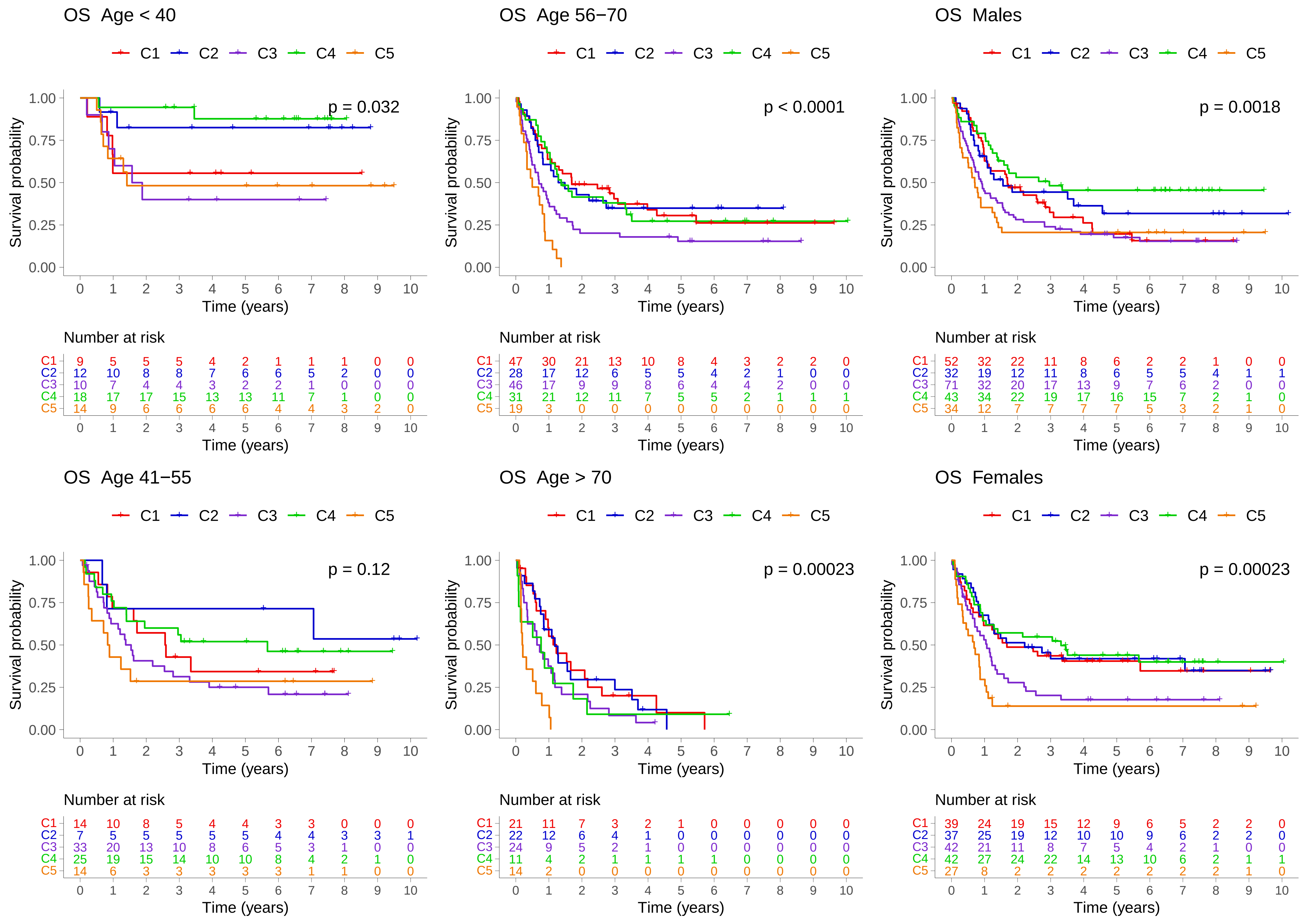
OS Age < 40
C1 C2 C3 C4 C5
p = 0.032
Survival probability
Time (years)
Number at risk
C1 9 5 5 5 4 2 1 1 1 0 0
C2 12 10 8 8 7 6 6 5 2 0 0
C3 10 7 4 4 3 2 2 1 0 0 0
C4 18 17 17 15 13 13 11 7 1 0 0
C5 14 9 6 6 6 6 4 4 3 2 0
OS Age 56−70
C1 C2 C3 C4 C5
p < 0.0001
Survival probability
Time (years)
Number at risk
C1 47 30 21 13 10 8 4 3 2 2 0
C2 28 17 12 6 5 5 4 2 1 0 0
C3 46 17 9 9 8 6 4 4 2 0 0
C4 31 21 12 11 7 5 5 2 1 1 1
C5 19 3 0 0 0 0 0 0 0 0 0
OS Males
C1 C2 C3 C4 C5
p = 0.0018
Survival probability
Time (years)
Number at risk
C1 52 32 22 11 8 6 2 2 1 0 0
C2 32 19 12 11 8 6 5 5 4 1 1
C3 71 32 20 17 13 9 7 6 2 0 0
C4 43 34 22 19 17 16 15 7 2 1 0
C5 34 12 7 7 7 7 5 3 2 1 0
OS Age 41−55
C1 C2 C3 C4 C5
p = 0.12
Survival probability
Time (years)
Number at risk
C1 14 10 8 5 4 4 3 3 0 0 0
C2 7 5 5 5 5 5 4 4 3 3 1
C3 33 20 13 10 8 6 5 3 1 0 0
C4 25 19 15 14 10 10 8 4 2 1 0
C5 14 6 3 3 3 3 3 1 1 0 0
OS Age > 70
C1 C2 C3 C4 C5
p = 0.00023
Survival probability
Time (years)
Number at risk
C1 21 11 7 3 2 1 0 0 0 0 0
C2 22 12 6 4 1 0 0 0 0 0 0
C3 24 9 5 2 1 0 0 0 0 0 0
C4 11 4 2 1 1 1 1 0 0 0 0
C5 14 2 0 0 0 0 0 0 0 0 0
OS Females
C1 C2 C3 C4 C5
p = 0.00023
Survival probability
Time (years)
Number at risk
C1 39 24 19 15 12 9 6 5 2 2 0
C2 37 25 19 12 10 10 9 6 2 2 0
C3 42 21 11 8 7 5 4 2 1 0 0
C4 42 27 24 22 14 13 10 6 2 1 1
C5 27 8 2 2 2 2 2 2 2 1 0

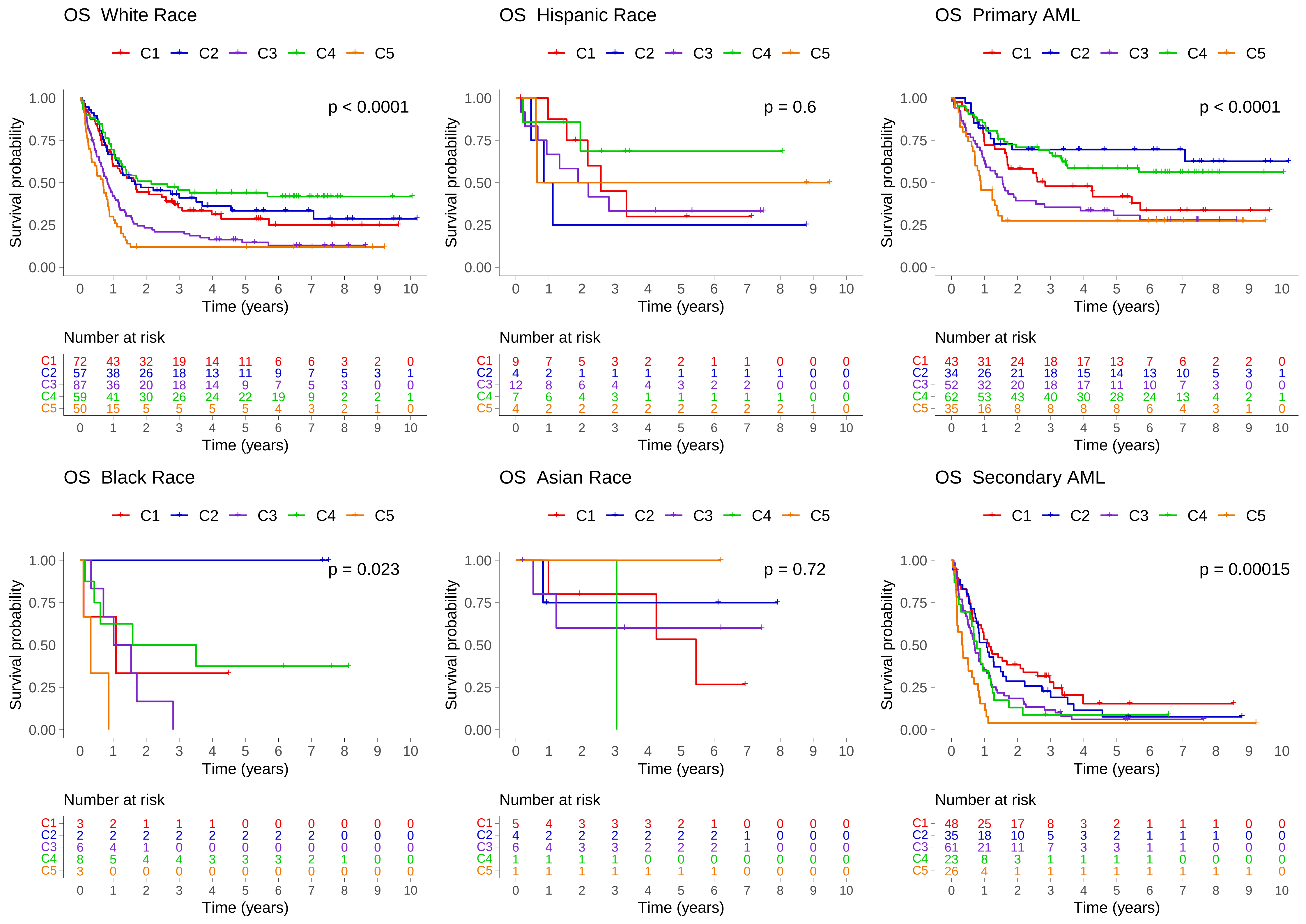

OS White Race
C1 C2 C3 C4 C5
p < 0.0001
Survival probability
Time (years)
Number at risk
C1 72 43 32 19 14 11 6 6 3 2 0
C2 57 38 26 18 13 11 9 7 5 3 1
C3 87 36 20 18 14 9 7 5 3 0 0
C4 59 41 30 26 24 22 19 9 2 2 1
C5 50 15 5 5 5 5 4 3 2 1 0
OS Hispanic Race
p = 0.6
Number at risk
C1 9 7 5 3 2 2 1 1 0 0 0
C2 4 2 1 1 1 1 1 1 1 0 0
C3 12 8 6 4 4 3 2 2 0 0 0
C4 7 6 4 3 1 1 1 1 1 0 0
C5 4 2 2 2 2 2 2 2 2 1 0
OS Primary AML
p < 0.0001
Number at risk
C1 43 31 24 18 17 13 7 6 2 2 0
C2 34 26 21 18 15 14 13 10 5 3 1
C3 52 32 20 18 17 11 10 7 3 0 0
C4 62 53 43 40 30 28 24 13 4 2 1
C5 35 16 8 8 8 8 6 4 3 1 0
OS Black Race
p = 0.023
Number at risk
C1 3 2 1 1 1 0 0 0 0 0 0
C2 2 2 2 2 2 2 2 2 0 0 0
C3 6 4 1 0 0 0 0 0 0 0 0
C4 8 5 4 4 3 3 3 2 1 0 0
C5 3 0 0 0 0 0 0 0 0 0 0
OS Asian Race
p = 0.72
Number at risk
C1 5 4 3 3 3 2 1 0 0 0 0
C2 4 2 2 2 2 2 2 1 0 0 0
C3 6 4 3 3 2 2 2 1 0 0 0
C4 1 1 1 1 0 0 0 0 0 0 0
C5 1 1 1 1 1 1 1 0 0 0 0
OS Secondary AML
p = 0.00015
Number at risk
C1 48 25 17 8 3 2 1 1 1 0 0
C2 35 18 10 5 3 2 1 1 1 0 0
C3 61 21 11 7 3 3 1 1 0 0 0
C4 23 8 3 1 1 1 1 0 0 0 0
C5 26 4 1 1 1 1 1 1 1 1 0

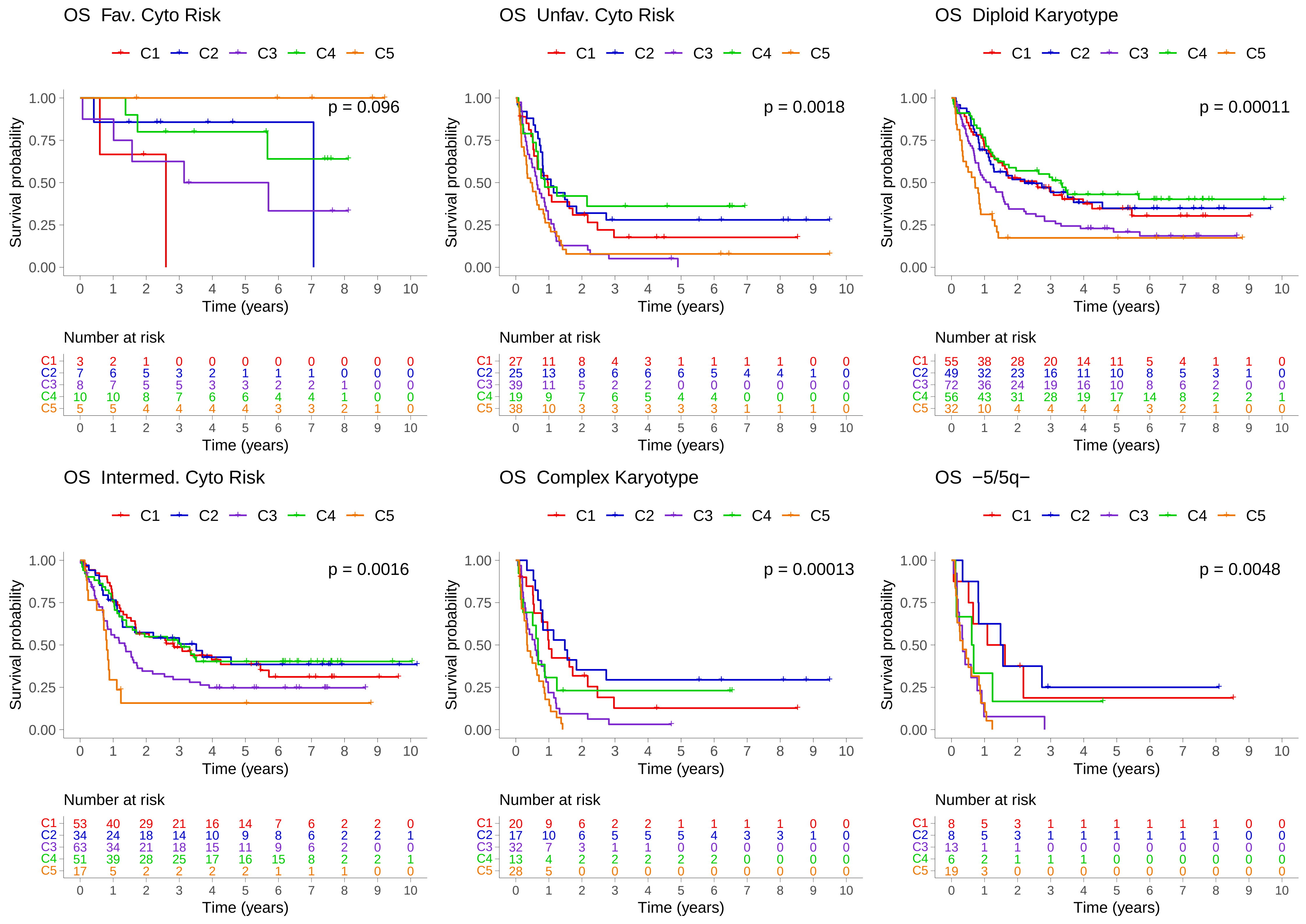
OS Fav. Cyto Risk
C1 C2 C3 C4 C5
p = 0.096
Survival probability
Time (years)
Number at risk
C1 3 2 1 0 0 0 0 0 0 0 0
C2 7 6 5 3 2 1 1 1 0 0 0
C3 8 7 5 5 3 3 2 2 1 0 0
C4 10 10 8 7 6 6 4 4 1 0 0
C5 5 5 4 4 4 4 3 3 2 1 0
OS Unfav. Cyto Risk
p = 0.0018
C1 27 11 8 4 3 1 1 1 1 0 0
C2 25 13 8 6 6 6 5 4 4 1 0
C3 39 11 5 2 2 0 0 0 0 0 0
C4 19 9 7 6 5 4 4 0 0 0 0
C5 38 10 3 3 3 3 3 1 1 1 0
OS Diploid Karyotype
p = 0.00011
C1 55 38 28 20 14 11 5 4 1 1 0
C2 49 32 23 16 11 10 8 5 3 1 0
C3 72 36 24 19 16 10 8 6 2 0 0
C4 56 43 31 28 19 17 14 8 2 2 1
C5 32 10 4 4 4 4 3 2 1 0 0
OS Intermed. Cyto Risk
p = 0.0016
C1 53 40 29 21 16 14 7 6 2 2 0
C2 34 24 18 14 10 9 8 6 2 2 1
C3 63 34 21 18 15 11 9 6 2 0 0
C4 51 39 28 25 17 16 15 8 2 2 1
C5 17 5 2 2 2 2 1 1 1 0 0
OS Complex Karyotype
p = 0.00013
C1 20 9 6 2 2 1 1 1 1 0 0
C2 17 10 6 5 5 5 4 3 3 1 0
C3 32 7 3 1 1 0 0 0 0 0 0
C4 13 4 2 2 2 2 2 0 0 0 0
C5 28 5 0 0 0 0 0 0 0 0 0
OS −5/5q−
p = 0.0048
C1 8 5 3 1 1 1 1 1 1 0 0
C2 8 5 3 1 1 1 1 1 1 0 0
C3 13 1 1 0 0 0 0 0 0 0 0
C4 6 2 1 1 1 0 0 0 0 0 0
C5 19 3 0 0 0 0 0 0 0 0 0

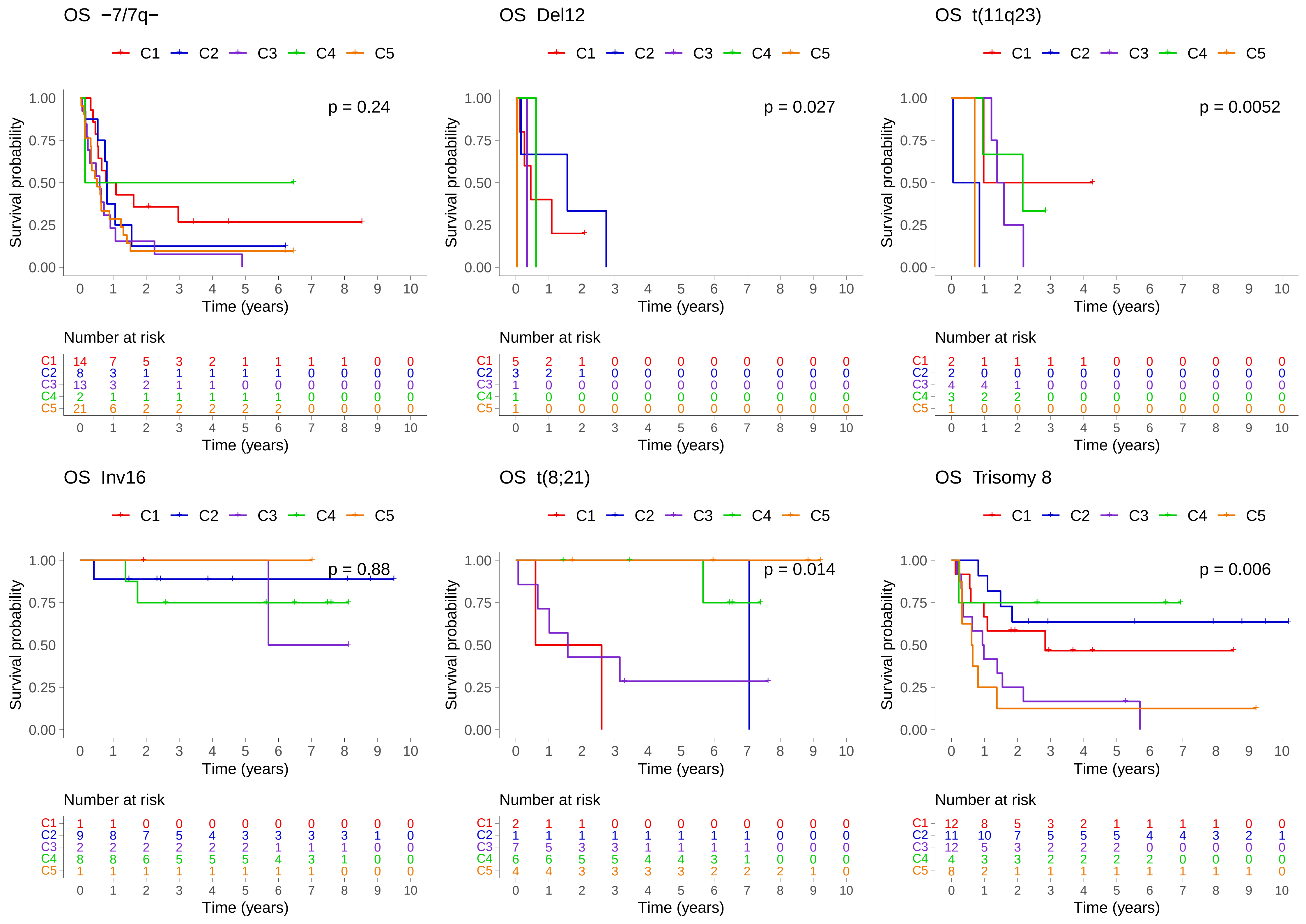
OS −7/7q−
C1 C2 C3 C4 C5
p = 0.24
Survival probability
Time (years)
Number at risk
C1 14 7 5 3 2 1 1 1 1 0 0
C2 8 3 1 1 1 1 1 0 0 0 0
C3 13 3 2 1 1 0 0 0 0 0 0
C4 2 1 1 1 1 1 1 0 0 0 0
C5 21 6 2 2 2 2 2 0 0 0 0
OS Del12
p = 0.027
Number at risk
C1 5 2 1 0 0 0 0 0 0 0 0
C2 3 2 1 0 0 0 0 0 0 0 0
C3 1 0 0 0 0 0 0 0 0 0 0
C4 1 0 0 0 0 0 0 0 0 0 0
C5 1 0 0 0 0 0 0 0 0 0 0
OS t(11q23)
p = 0.0052
Number at risk
C1 2 1 1 1 1 0 0 0 0 0 0
C2 2 0 0 0 0 0 0 0 0 0 0
C3 4 4 1 0 0 0 0 0 0 0 0
C4 3 2 2 0 0 0 0 0 0 0 0
C5 1 0 0 0 0 0 0 0 0 0 0
OS Inv16
p = 0.88
Number at risk
C1 1 1 0 0 0 0 0 0 0 0 0
C2 9 8 7 5 4 3 3 3 3 1 0
C3 2 2 2 2 2 2 1 1 1 0 0
C4 8 8 6 5 5 5 4 3 1 0 0
C5 1 1 1 1 1 1 1 1 0 0 0
OS t(8;21)
p = 0.014
Number at risk
C1 2 1 1 0 0 0 0 0 0 0 0
C2 1 1 1 1 1 1 1 1 0 0 0
C3 7 5 3 3 1 1 1 1 0 0 0
C4 6 6 5 5 4 4 3 1 0 0 0
C5 4 4 3 3 3 3 2 2 2 1 0
OS Trisomy 8
p = 0.006
Number at risk
C1 12 8 5 3 2 1 1 1 1 0 0
C2 11 10 7 5 5 5 4 4 3 2 1
C3 12 5 3 2 2 2 0 0 0 0 0
C4 4 3 3 2 2 2 2 0 0 0 0
C5 8 2 1 1 1 1 1 1 1 1 0

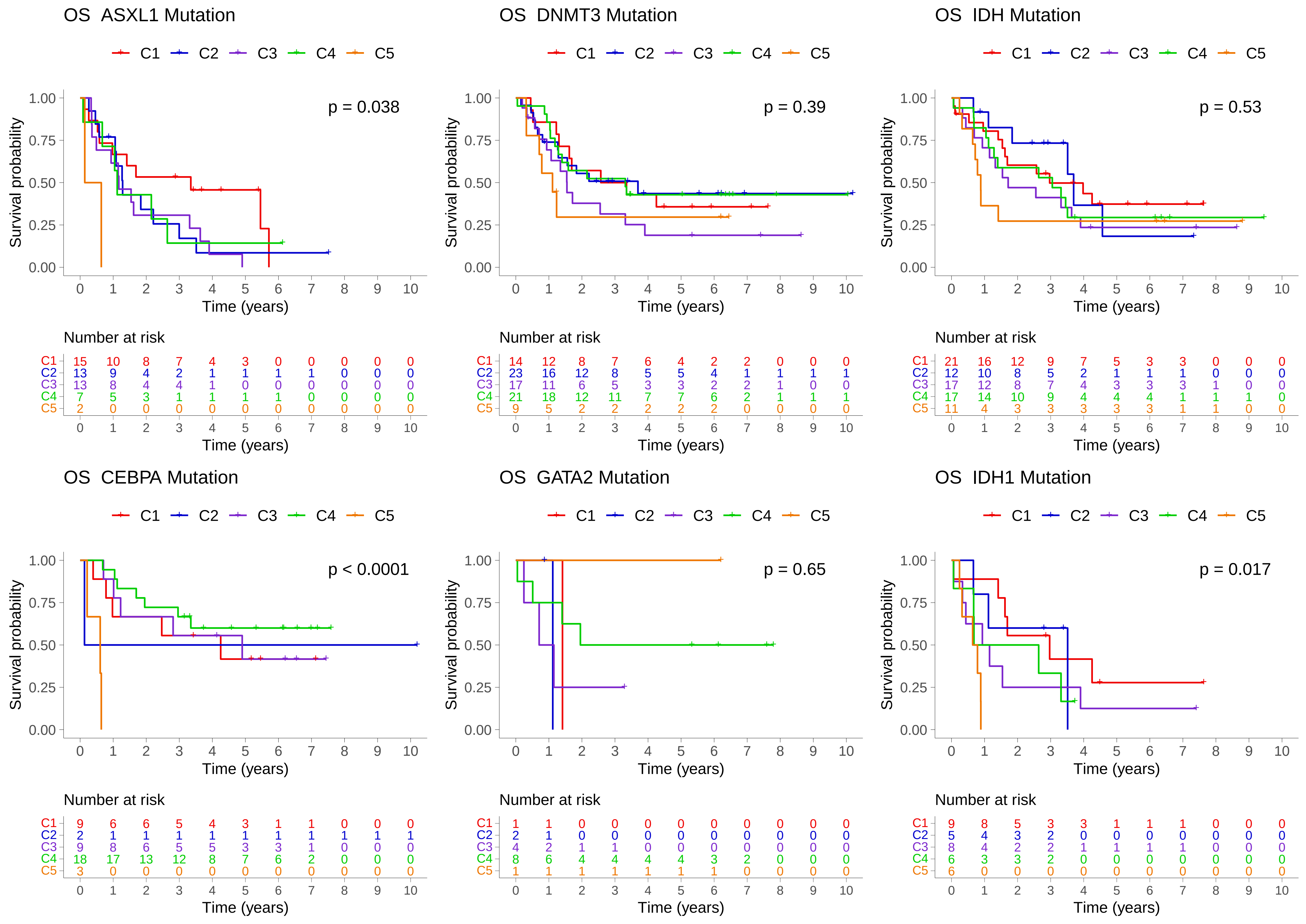
OS ASXL1 Mutation
C1 C2 C3 C4 C5
p = 0.038
Survival probability
Time (years)
Number at risk
C1 15 10 8 7 4 3 0 0 0 0 0
C2 13 9 4 2 1 1 1 1 0 0 0
C3 13 8 4 4 1 0 0 0 0 0 0
C4 7 5 3 1 1 1 1 0 0 0 0
C5 2 0 0 0 0 0 0 0 0 0 0
OS DNMT3 Mutation
C1 C2 C3 C4 C5
p = 0.39
Survival probability
Time (years)
Number at risk
C1 14 12 8 7 6 4 2 2 0 0 0
C2 23 16 12 8 5 5 4 1 1 1 1
C3 17 11 6 5 3 3 2 2 1 0 0
C4 21 18 12 11 7 7 6 2 1 1 1
C5 9 5 2 2 2 2 2 0 0 0 0
OS IDH Mutation
C1 C2 C3 C4 C5
p = 0.53
Survival probability
Time (years)
Number at risk
C1 21 16 12 9 7 5 3 3 0 0 0
C2 12 10 8 5 2 1 1 1 0 0 0
C3 17 12 8 7 4 3 3 3 1 0 0
C4 17 14 10 9 4 4 4 1 1 1 0
C5 11 4 3 3 3 3 3 1 1 0 0
OS CEBPA Mutation
C1 C2 C3 C4 C5
p < 0.0001
Survival probability
Time (years)
Number at risk
C1 9 6 6 5 4 3 1 1 0 0 0
C2 2 1 1 1 1 1 1 1 1 1 1
C3 9 8 6 5 5 3 3 1 0 0 0
C4 18 17 13 12 8 7 6 2 0 0 0
C5 3 0 0 0 0 0 0 0 0 0 0
OS GATA2 Mutation
C1 C2 C3 C4 C5
p = 0.65
Survival probability
Time (years)
Number at risk
C1 1 1 0 0 0 0 0 0 0 0 0
C2 2 1 0 0 0 0 0 0 0 0 0
C3 4 2 1 1 0 0 0 0 0 0 0
C4 8 6 4 4 4 4 3 2 0 0 0
C5 1 1 1 1 1 1 1 0 0 0 0
OS IDH1 Mutation
C1 C2 C3 C4 C5
p = 0.017
Survival probability
Time (years)
Number at risk
C1 9 8 5 3 3 1 1 1 0 0 0
C2 5 4 3 2 0 0 0 0 0 0 0
C3 8 4 2 2 1 1 1 1 0 0 0
C4 6 3 3 2 0 0 0 0 0 0 0
C5 6 0 0 0 0 0 0 0 0 0 0

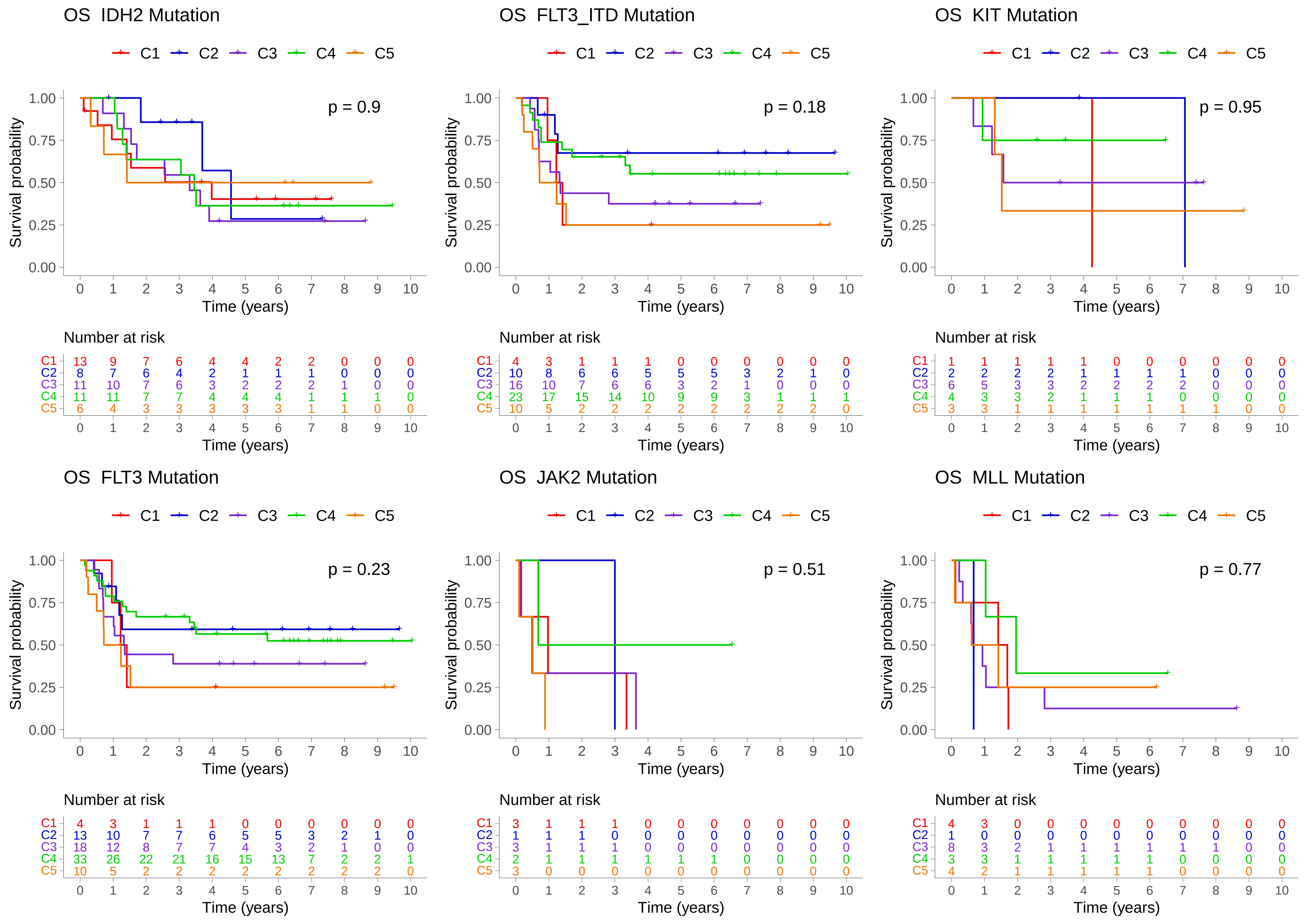
OS IDH2 Mutation
C1 C2 C3 C4 C5
p = 0.9
Survival probability
Time (years)
Number at risk
C1 13 9 7 6 4 4 2 2 0 0 0
C2 8 7 6 4 2 1 1 1 0 0 0
C3 11 10 7 6 3 2 2 2 1 0 0
C4 11 11 7 7 4 4 4 1 1 1 0
C5 6 4 3 3 3 3 3 1 1 0 0
OS FLT3_ITD Mutation
C1 C2 C3 C4 C5
p = 0.18
Survival probability
Time (years)
Number at risk
C1 4 3 1 1 1 0 0 0 0 0 0
C2 10 8 6 6 5 5 5 3 2 1 0
C3 16 10 7 6 6 3 2 1 0 0 0
C4 23 17 15 14 10 9 9 3 1 1 1
C5 10 5 2 2 2 2 2 2 2 2 0
OS KIT Mutation
C1 C2 C3 C4 C5
p = 0.95
Survival probability
Time (years)
Number at risk
C1 1 1 1 1 1 0 0 0 0 0 0
C2 2 2 2 2 1 1 1 1 0 0 0
C3 6 5 3 3 2 2 2 2 0 0 0
C4 4 3 3 2 1 1 1 0 0 0 0
C5 3 3 1 1 1 1 1 1 1 0 0
OS FLT3 Mutation
C1 C2 C3 C4 C5
p = 0.23
Survival probability
Time (years)
Number at risk
C1 4 3 1 1 1 0 0 0 0 0 0
C2 13 10 7 7 6 5 5 3 2 1 0
C3 18 12 8 7 7 4 3 2 1 0 0
C4 33 26 22 21 16 15 13 7 2 2 1
C5 10 5 2 2 2 2 2 2 2 2 0
OS JAK2 Mutation
C1 C2 C3 C4 C5
p = 0.51
Survival probability
Time (years)
Number at risk
C1 3 1 1 1 0 0 0 0 0 0 0
C2 1 1 1 0 0 0 0 0 0 0 0
C3 3 1 1 1 0 0 0 0 0 0 0
C4 2 1 1 1 1 1 1 0 0 0 0
C5 3 0 0 0 0 0 0 0 0 0 0
OS MLL Mutation
C1 C2 C3 C4 C5
p = 0.77
Survival probability
Time (years)
Number at risk
C1 4 3 0 0 0 0 0 0 0 0 0
C2 1 0 0 0 0 0 0 0 0 0 0
C3 8 3 2 1 1 1 1 1 0 0 0
C4 3 3 1 1 1 1 1 0 0 0 0
C5 4 2 1 1 1 1 1 0 0 0 0

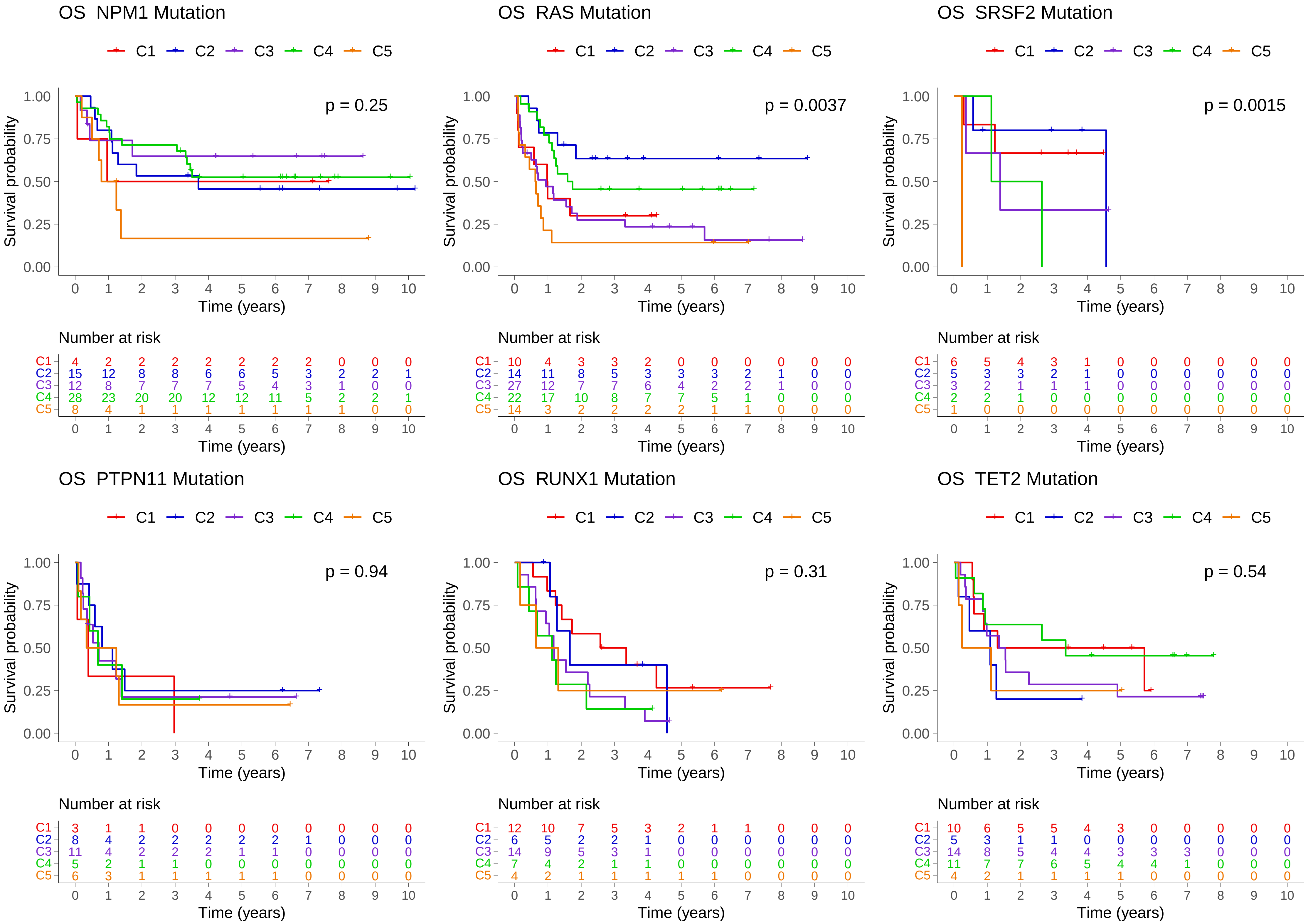
OS NPM1 Mutation
C1 C2 C3 C4 C5
p = 0.25
Survival probability
Time (years)
Number at risk
C1 4 2 2 2 2 2 2 2 0 0 0
C2 15 12 8 8 6 6 5 3 2 2 1
C3 12 8 7 7 7 5 4 3 1 0 0
C4 28 23 20 20 12 12 11 5 2 2 1
C5 8 4 1 1 1 1 1 1 1 0 0
OS RAS Mutation
p = 0.0037
Number at risk
C1 10 4 3 3 2 0 0 0 0 0 0
C2 14 11 8 5 3 3 3 2 1 0 0
C3 27 12 7 7 6 4 2 2 1 0 0
C4 22 17 10 8 7 7 5 1 0 0 0
C5 14 3 2 2 2 2 1 1 0 0 0
OS SRSF2 Mutation
p = 0.0015
Number at risk
C1 6 5 4 3 1 0 0 0 0 0 0
C2 5 3 3 2 1 0 0 0 0 0 0
C3 3 2 1 1 1 0 0 0 0 0 0
C4 2 2 1 0 0 0 0 0 0 0 0
C5 1 0 0 0 0 0 0 0 0 0 0
OS PTPN11 Mutation
p = 0.94
Number at risk
C1 3 1 1 0 0 0 0 0 0 0 0
C2 8 4 2 2 2 2 2 1 0 0 0
C3 11 4 2 2 2 1 1 0 0 0 0
C4 5 2 1 1 0 0 0 0 0 0 0
C5 6 3 1 1 1 1 1 0 0 0 0
OS RUNX1 Mutation
p = 0.31
Number at risk
C1 12 10 7 5 3 2 1 1 0 0 0
C2 6 5 2 2 1 0 0 0 0 0 0
C3 14 9 5 3 1 0 0 0 0 0 0
C4 7 4 2 1 1 0 0 0 0 0 0
C5 4 2 1 1 1 1 1 0 0 0 0
OS TET2 Mutation
p = 0.54
Number at risk
C1 10 6 5 5 4 3 0 0 0 0 0
C2 5 3 1 1 0 0 0 0 0 0 0
C3 14 8 5 4 4 3 3 3 0 0 0
C4 11 7 7 6 5 4 4 1 0 0 0
C5 4 2 1 1 1 1 0 0 0 0 0

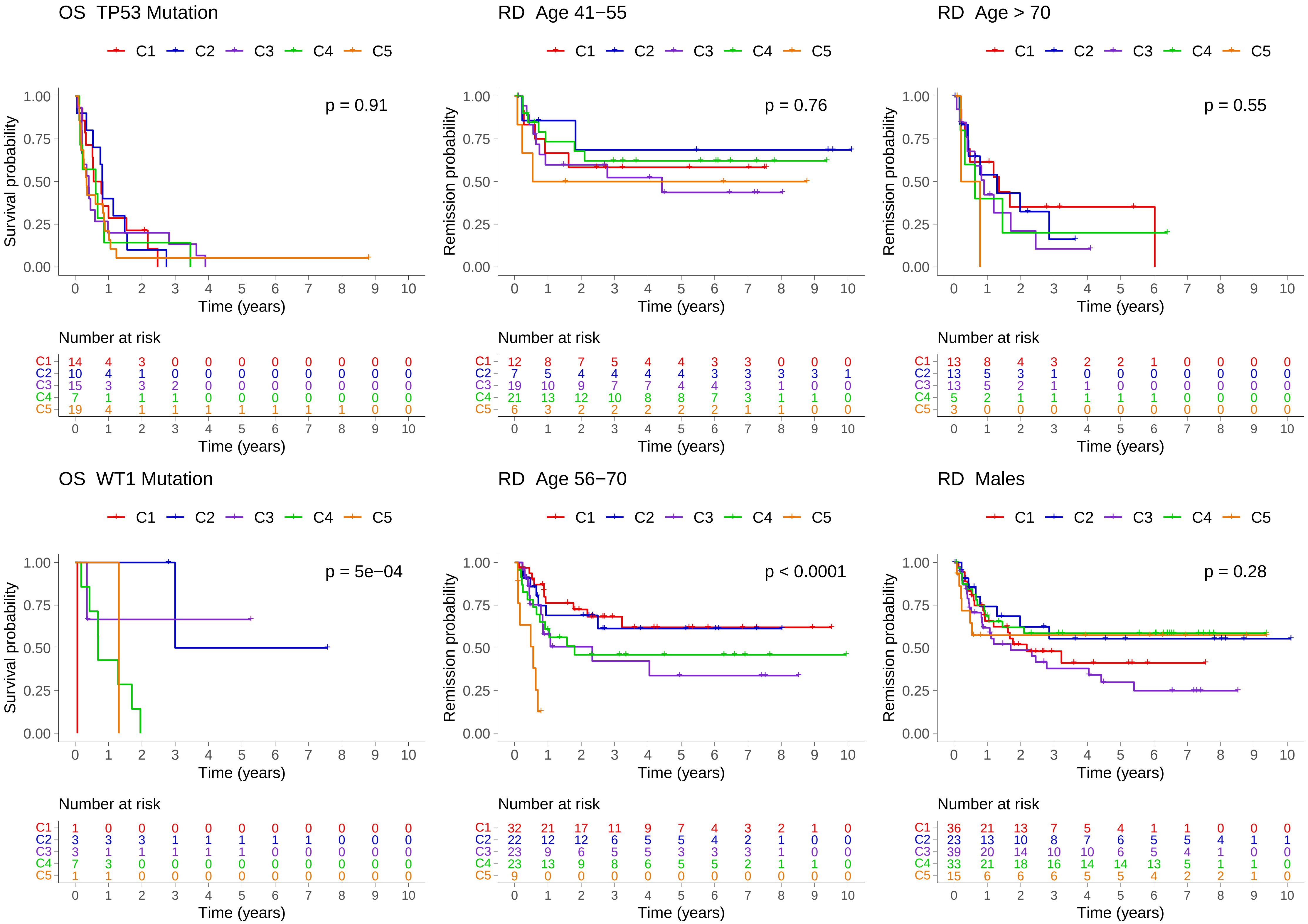

OS TP53 Mutation
C1 C2 C3 C4 C5
p = 0.91
Survival probability
1.00 0.75 0.50 0.25 0.00
Time (years)
0 1 2 3 4 5 6 7 8 9 10
Number at risk
C1 14 4 3 0 0 0 0 0 0 0 0
C2 10 4 1 0 0 0 0 0 0 0 0
C3 15 3 3 2 0 0 0 0 0 0 0
C4 7 1 1 1 0 0 0 0 0 0 0
C5 19 4 1 1 1 1 1 1 1 0 0
RD Age 41−55
p = 0.76
Remission probability
Number at risk
C1 12 8 7 5 4 4 3 3 0 0 0
C2 7 5 4 4 4 4 3 3 3 3 1
C3 19 10 9 7 7 4 4 3 1 0 0
C4 21 13 12 10 8 8 7 3 1 1 0
C5 6 3 2 2 2 2 2 1 1 0 0
RD Age > 70
p = 0.55
Number at risk
C1 13 8 4 3 2 2 1 0 0 0 0
C2 13 5 3 1 0 0 0 0 0 0 0
C3 13 5 2 1 1 0 0 0 0 0 0
C4 5 2 1 1 1 1 1 0 0 0 0
C5 3 0 0 0 0 0 0 0 0 0 0
OS WT1 Mutation
p = 5e−04
Number at risk
C1 1 0 0 0 0 0 0 0 0 0 0
C2 3 3 3 1 1 1 1 1 0 0 0
C3 3 1 1 1 1 1 0 0 0 0 0
C4 7 3 0 0 0 0 0 0 0 0 0
C5 1 1 0 0 0 0 0 0 0 0 0
RD Age 56−70
p < 0.0001
Number at risk
C1 32 21 17 11 9 7 4 3 2 1 0
C2 22 12 12 6 5 5 4 2 1 0 0
C3 23 9 6 5 5 3 3 3 1 0 0
C4 23 13 9 8 6 5 5 2 1 1 0
C5 9 0 0 0 0 0 0 0 0 0 0
RD Males
p = 0.28
Number at risk
C1 36 21 13 7 5 4 1 1 0 0 0
C2 23 13 10 8 7 6 5 5 4 1 1
C3 39 20 14 10 10 6 5 4 1 0 0
C4 33 21 18 16 14 14 13 5 1 1 0
C5 15 6 6 6 5 5 4 2 2 1 0

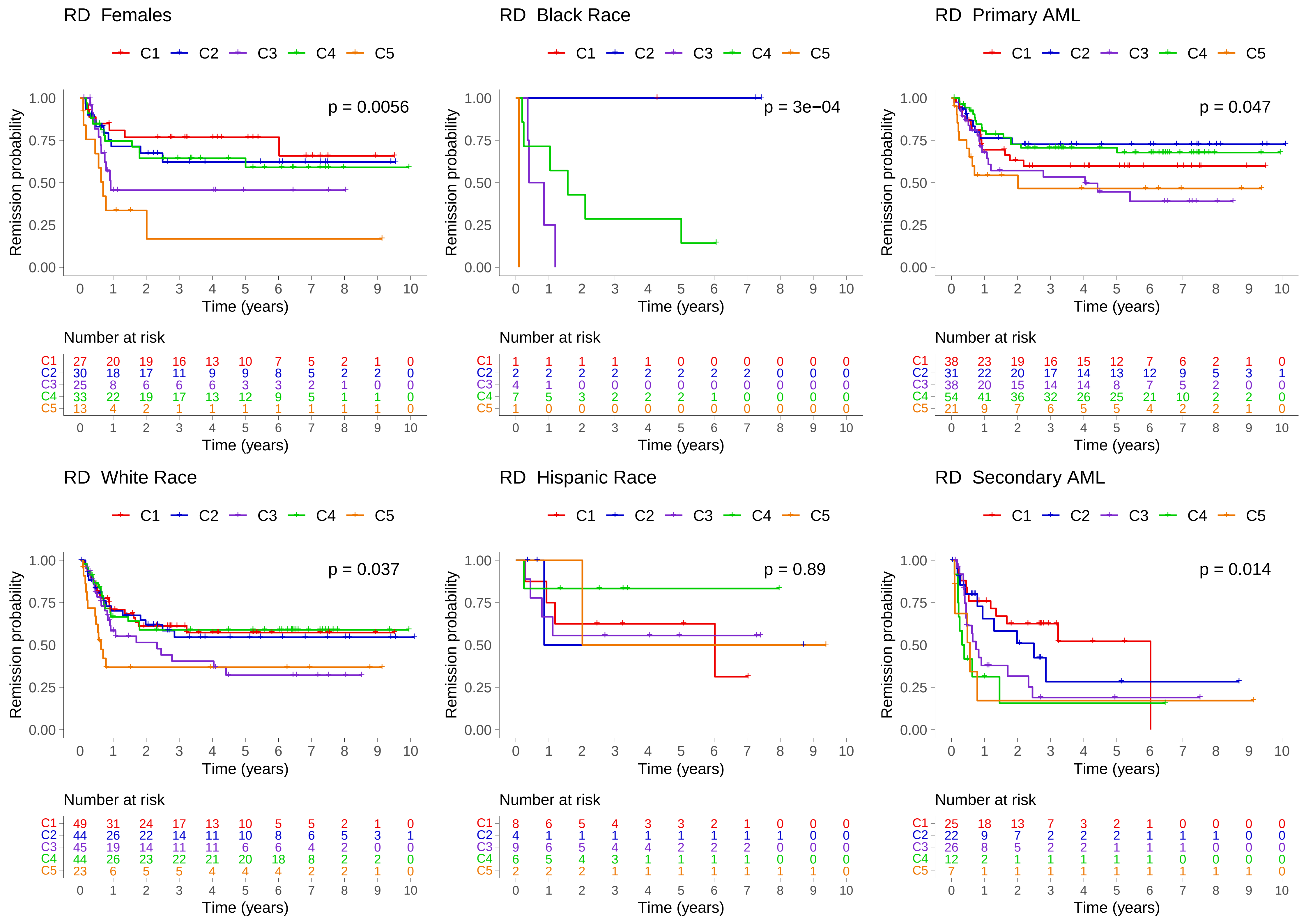
RD Females
C1 C2 C3 C4 C5
p = 0.0056
Remission probability
Time (years)
Number at risk
C1 27 20 19 16 13 10 7 5 2 1 0
C2 30 18 17 11 9 9 8 5 2 2 0
C3 25 8 6 6 6 3 3 2 1 0 0
C4 33 22 19 17 13 12 9 5 1 1 0
C5 13 4 2 1 1 1 1 1 1 1 0
RD Black Race
C1 C2 C3 C4 C5
p = 3e−04
Remission probability
Time (years)
Number at risk
C1 1 1 1 1 1 0 0 0 0 0 0
C2 2 2 2 2 2 2 2 2 0 0 0
C3 4 1 0 0 0 0 0 0 0 0 0
C4 7 5 3 2 2 2 1 0 0 0 0
C5 1 0 0 0 0 0 0 0 0 0 0
RD Primary AML
C1 C2 C3 C4 C5
p = 0.047
Remission probability
Time (years)
Number at risk
C1 38 23 19 16 15 12 7 6 2 1 0
C2 31 22 20 17 14 13 12 9 5 3 1
C3 38 20 15 14 14 8 7 5 2 0 0
C4 54 41 36 32 26 25 21 10 2 2 0
C5 21 9 7 6 5 5 4 2 2 1 0
RD White Race
C1 C2 C3 C4 C5
p = 0.037
Remission probability
Time (years)
Number at risk
C1 49 31 24 17 13 10 5 5 2 1 0
C2 44 26 22 14 11 10 8 6 5 3 1
C3 45 19 14 11 11 6 6 4 2 0 0
C4 44 26 23 22 21 20 18 8 2 2 0
C5 23 6 5 5 4 4 4 2 2 1 0
RD Hispanic Race
C1 C2 C3 C4 C5
p = 0.89
Remission probability
Time (years)
Number at risk
C1 8 6 5 4 3 3 2 1 0 0 0
C2 4 1 1 1 1 1 1 1 1 0 0
C3 9 6 5 4 4 2 2 2 0 0 0
C4 6 5 4 3 1 1 1 1 0 0 0
C5 2 2 2 1 1 1 1 1 1 1 0
RD Secondary AML
C1 C2 C3 C4 C5
p = 0.014
Remission probability
Time (years)
Number at risk
C1 25 18 13 7 3 2 1 0 0 0 0
C2 22 9 7 2 2 2 1 1 1 0 0
C3 26 8 5 2 2 1 1 1 0 0 0
C4 12 2 1 1 1 1 1 0 0 0 0
C5 7 1 1 1 1 1 1 1 1 1 0

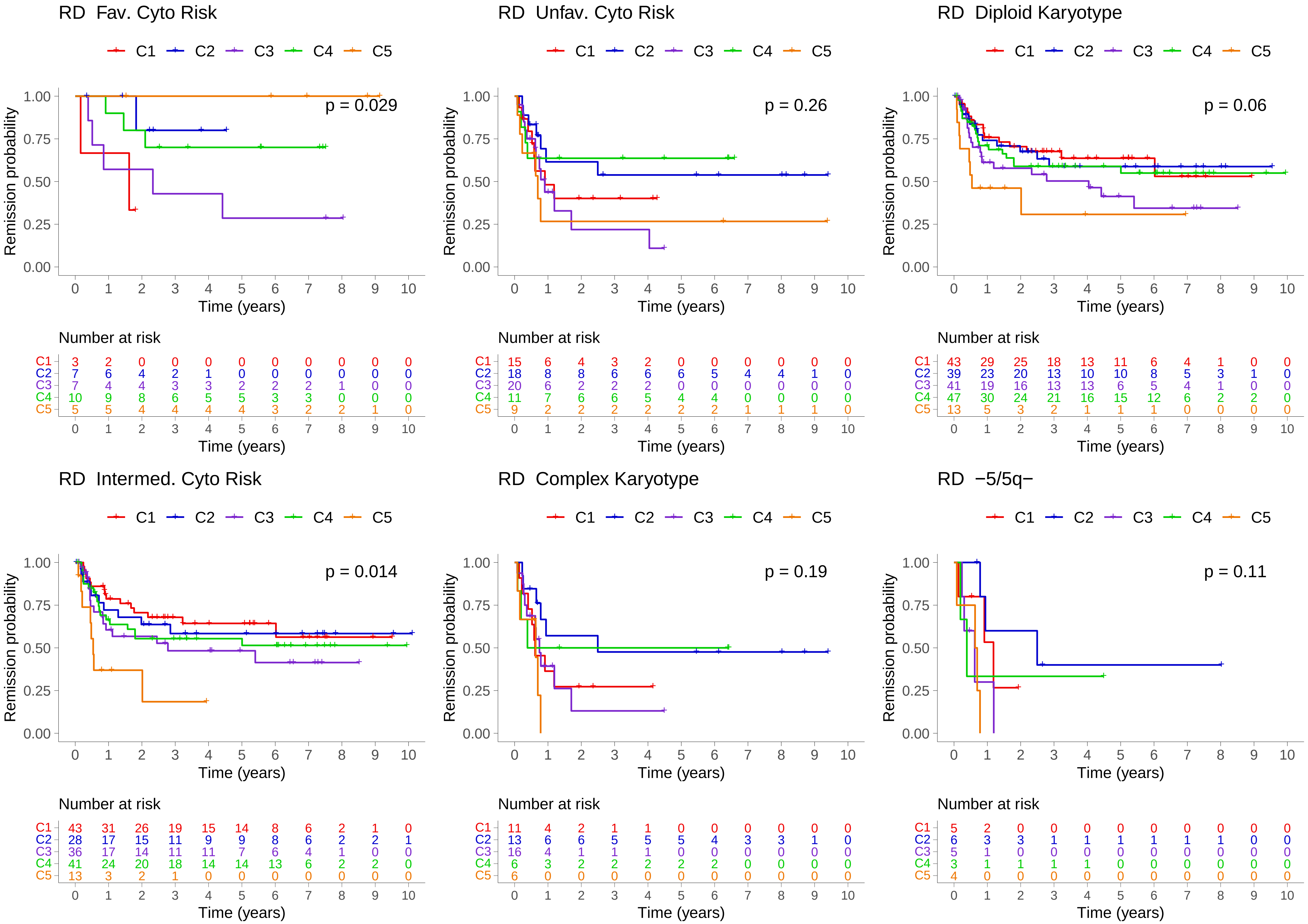
RD Fav. Cyto Risk
C1 C2 C3 C4 C5
p = 0.029
Remission probability
Time (years)
Number at risk
C1 3 2 0 0 0 0 0 0 0 0 0
C2 7 6 4 2 1 0 0 0 0 0 0
C3 7 4 4 3 3 2 2 2 1 0 0
C4 10 9 8 6 5 5 3 3 0 0 0
C5 5 5 4 4 4 4 3 2 2 1 0
RD Unfav. Cyto Risk
C1 C2 C3 C4 C5
p = 0.26
Remission probability
Time (years)
Number at risk
C1 15 6 4 3 2 0 0 0 0 0 0
C2 18 8 8 6 6 6 5 4 4 1 0
C3 20 6 2 2 2 0 0 0 0 0 0
C4 11 7 6 6 5 4 4 0 0 0 0
C5 9 2 2 2 2 2 2 1 1 1 0
RD Diploid Karyotype
C1 C2 C3 C4 C5
p = 0.06
Remission probability
Time (years)
Number at risk
C1 43 29 25 18 13 11 6 4 1 0 0
C2 39 23 20 13 10 10 8 5 3 1 0
C3 41 19 16 13 13 6 5 4 1 0 0
C4 47 30 24 21 16 15 12 6 2 2 0
C5 13 5 3 2 1 1 1 0 0 0 0
RD Intermed. Cyto Risk
C1 C2 C3 C4 C5
p = 0.014
Remission probability
Time (years)
Number at risk
C1 43 31 26 19 15 14 8 6 2 1 0
C2 28 17 15 11 9 9 8 6 2 2 1
C3 36 17 14 11 11 7 6 4 1 0 0
C4 41 24 20 18 14 14 13 6 2 2 0
C5 13 3 2 1 0 0 0 0 0 0 0
RD Complex Karyotype
C1 C2 C3 C4 C5
p = 0.19
Remission probability
Time (years)
Number at risk
C1 11 4 2 1 1 0 0 0 0 0 0
C2 13 6 6 5 5 5 4 3 3 1 0
C3 16 4 1 1 1 0 0 0 0 0 0
C4 6 3 2 2 2 2 2 0 0 0 0
C5 6 0 0 0 0 0 0 0 0 0 0
RD −5/5q−
C1 C2 C3 C4 C5
p = 0.11
Remission probability
Time (years)
Number at risk
C1 5 2 0 0 0 0 0 0 0 0 0
C2 6 3 3 1 1 1 1 1 1 0 0
C3 5 1 0 0 0 0 0 0 0 0 0
C4 3 1 1 1 1 0 0 0 0 0 0
C5 4 0 0 0 0 0 0 0 0 0 0

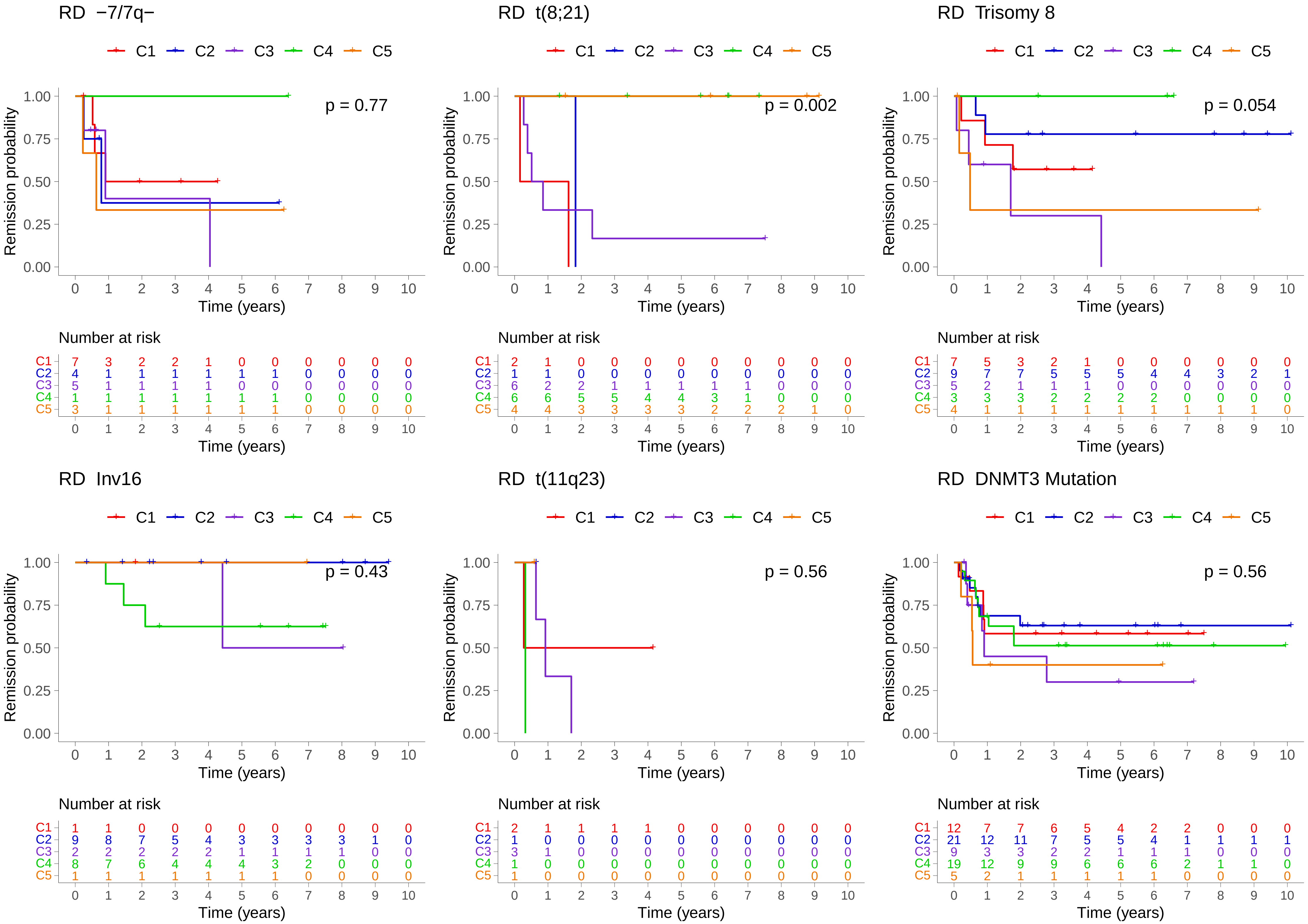
RD −7/7q−
C1 C2 C3 C4 C5
p = 0.77
Remission probability
Time (years)
Number at risk
C1 7 3 2 2 1 0 0 0 0 0 0
C2 4 1 1 1 1 1 1 0 0 0 0
C3 5 1 1 1 1 0 0 0 0 0 0
C4 1 1 1 1 1 1 1 0 0 0 0
C5 3 1 1 1 1 1 1 0 0 0 0
RD t(8;21)
p = 0.002
C1 2 1 0 0 0 0 0 0 0 0 0
C2 1 1 0 0 0 0 0 0 0 0 0
C3 6 2 2 1 1 1 1 1 0 0 0
C4 6 6 5 5 4 4 3 1 0 0 0
C5 4 4 3 3 3 3 2 2 2 1 0
RD Trisomy 8
p = 0.054
C1 7 5 3 2 1 0 0 0 0 0 0
C2 9 7 7 5 5 5 4 4 3 2 1
C3 5 2 1 1 1 0 0 0 0 0 0
C4 3 3 3 2 2 2 2 0 0 0 0
C5 4 1 1 1 1 1 1 1 1 1 0
RD Inv16
p = 0.43
C1 1 1 0 0 0 0 0 0 0 0 0
C2 9 8 7 5 4 3 3 3 3 1 0
C3 2 2 2 2 2 1 1 1 1 0 0
C4 8 7 6 4 4 4 3 2 0 0 0
C5 1 1 1 1 1 1 1 0 0 0 0
RD t(11q23)
p = 0.56
C1 2 1 1 1 1 0 0 0 0 0 0
C2 1 0 0 0 0 0 0 0 0 0 0
C3 3 1 0 0 0 0 0 0 0 0 0
C4 1 0 0 0 0 0 0 0 0 0 0
C5 1 0 0 0 0 0 0 0 0 0 0
RD DNMT3 Mutation
p = 0.56
C1 12 7 7 6 5 4 2 2 0 0 0
C2 21 12 11 7 5 5 4 1 1 1 1
C3 9 3 3 2 2 1 1 1 0 0 0
C4 19 12 9 9 6 6 6 2 1 1 0
C5 5 2 1 1 1 1 1 0 0 0 0

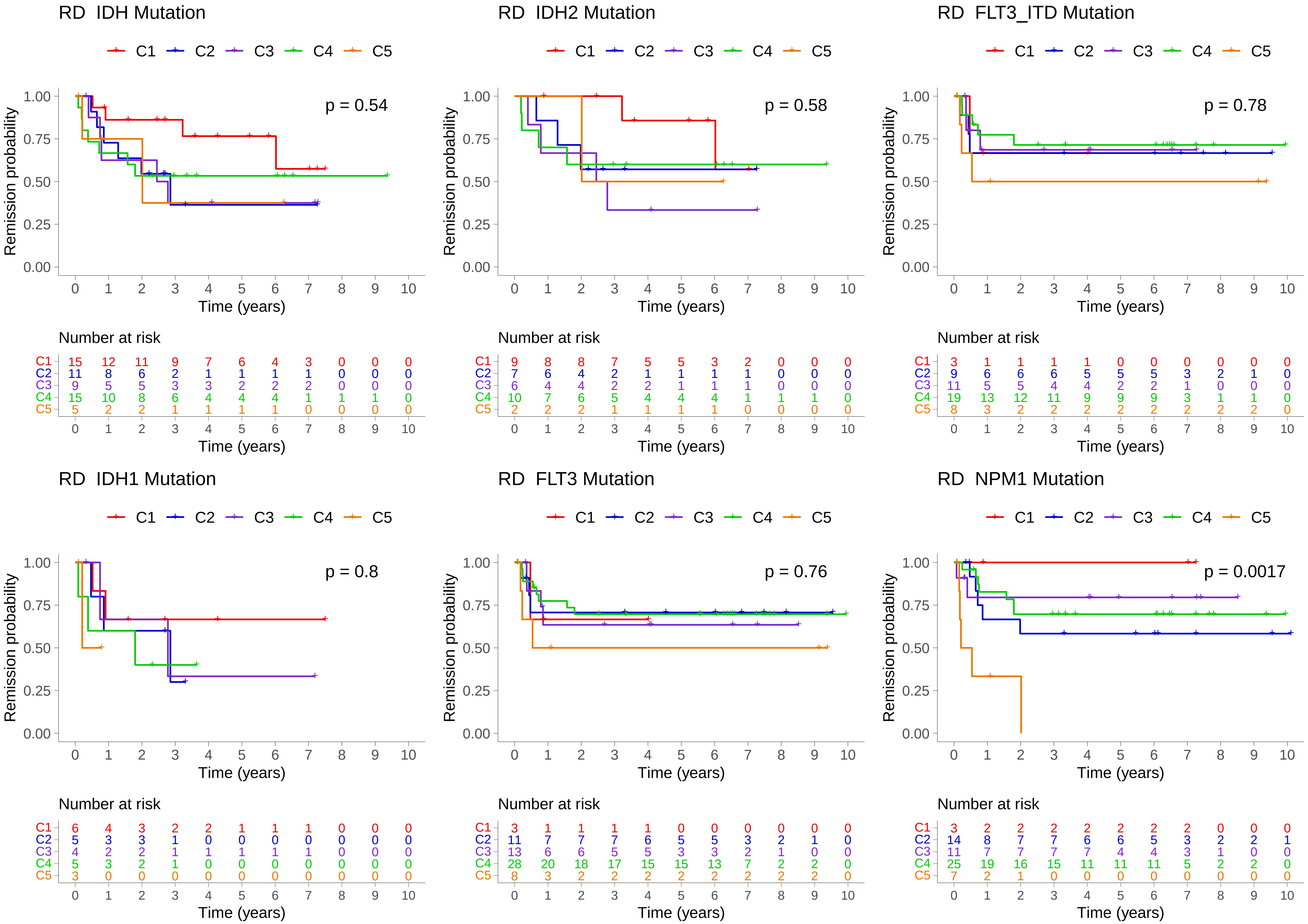
RD IDH Mutation
C1 C2 C3 C4 C5
p = 0.54
Remission probability
1.00 0.75 0.50 0.25 0.00
Time (years)
0 1 2 3 4 5 6 7 8 9 10
Number at risk
C1 15 12 11 9 7 6 4 3 0 0 0
C2 11 8 6 2 1 1 1 1 0 0 0
C3 9 5 5 3 3 2 2 2 0 0 0
C4 15 10 8 6 4 4 4 1 1 1 0
C5 5 2 2 1 1 1 1 0 0 0 0
Time (years)
RD IDH2 Mutation
C1 C2 C3 C4 C5
p = 0.58
Remission probability
Time (years)
Number at risk
C1 9 8 8 7 5 5 3 2 0 0 0
C2 7 6 4 2 1 1 1 1 0 0 0
C3 6 4 4 2 2 1 1 1 0 0 0
C4 10 7 6 5 4 4 4 1 1 1 0
C5 2 2 2 1 1 1 1 0 0 0 0
Time (years)
RD FLT3_ITD Mutation
C1 C2 C3 C4 C5
p = 0.78
Remission probability
Time (years)
Number at risk
C1 3 1 1 1 1 0 0 0 0 0 0
C2 9 6 6 6 5 5 5 3 2 1 0
C3 11 5 5 4 4 2 2 1 0 0 0
C4 19 13 12 11 9 9 9 3 1 1 0
C5 8 3 2 2 2 2 2 2 2 2 0
Time (years)
RD IDH1 Mutation
C1 C2 C3 C4 C5
p = 0.8
Remission probability
Time (years)
Number at risk
C1 6 4 3 2 2 1 1 1 0 0 0
C2 5 3 3 1 0 0 0 0 0 0 0
C3 4 2 2 1 1 1 1 1 0 0 0
C4 5 3 2 1 0 0 0 0 0 0 0
C5 3 0 0 0 0 0 0 0 0 0 0
Time (years)
RD FLT3 Mutation
C1 C2 C3 C4 C5
p = 0.76
Remission probability
Time (years)
Number at risk
C1 3 1 1 1 1 0 0 0 0 0 0
C2 11 7 7 7 6 5 5 3 2 1 0
C3 13 6 6 5 5 3 3 2 1 0 0
C4 28 20 18 17 15 15 13 7 2 2 0
C5 8 3 2 2 2 2 2 2 2 2 0
Time (years)
RD NPM1 Mutation
C1 C2 C3 C4 C5
p = 0.0017
Remission probability
Time (years)
Number at risk
C1 3 2 2 2 2 2 2 2 0 0 0
C2 14 8 7 7 6 6 5 3 2 2 1
C3 11 7 7 7 7 4 4 3 1 0 0
C4 25 19 16 15 11 11 11 5 2 2 0
C5 7 2 1 0 0 0 0 0 0 0 0
Time (years)

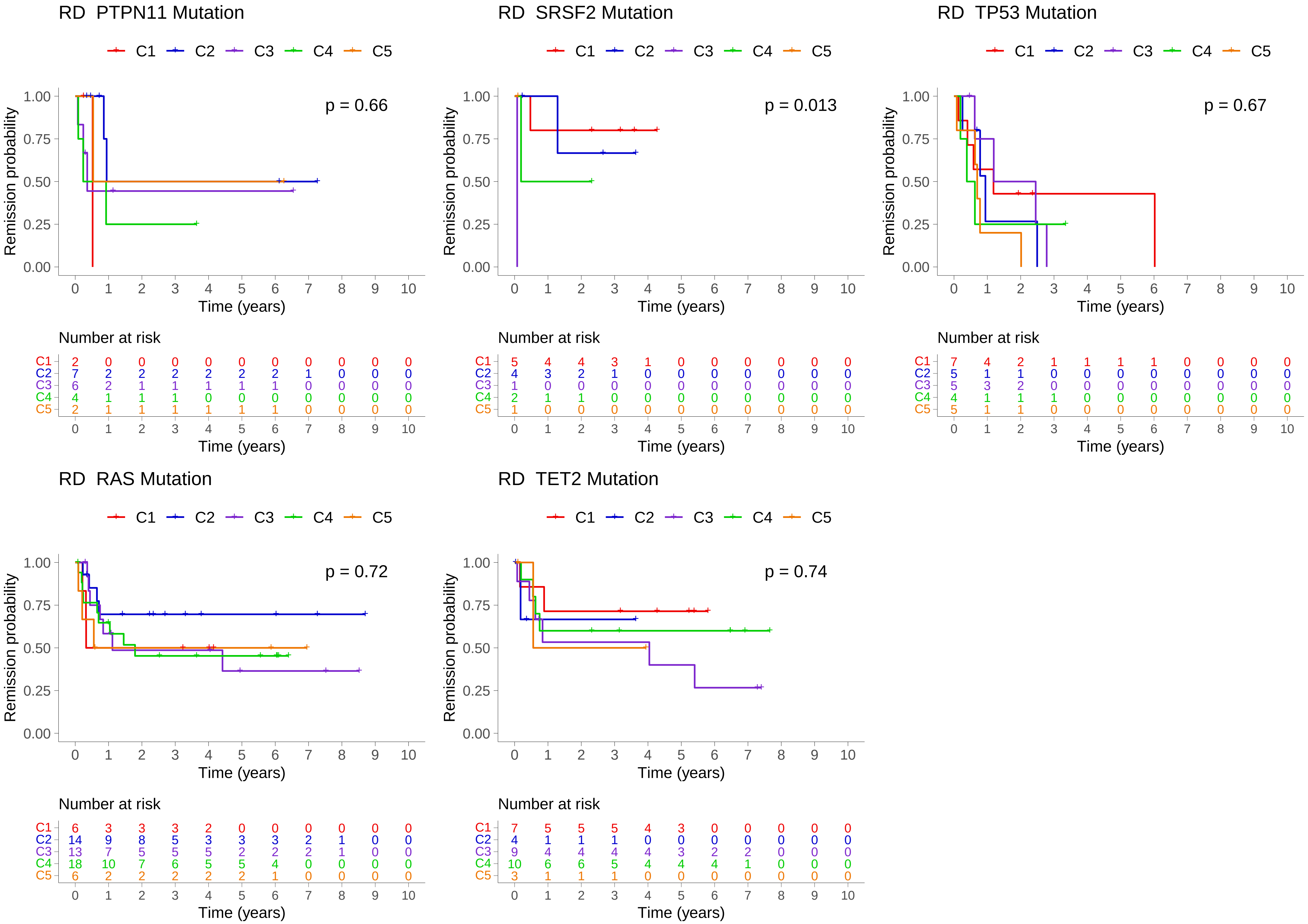

RD PTPN11 Mutation
C1 C2 C3 C4 C5
p = 0.66
Remission probability
1.00 0.75 0.50 0.25 0.00
Time (years)
0 1 2 3 4 5 6 7 8 9 10
Number at risk
C1 2 0 0 0 0 0 0 0 0 0 0
C2 7 2 2 2 2 2 2 1 0 0 0
C3 6 2 1 1 1 1 1 0 0 0 0
C4 4 1 1 1 0 0 0 0 0 0 0
C5 2 1 1 1 1 1 1 0 0 0 0
RD SRSF2 Mutation
C1 C2 C3 C4 C5
p = 0.013
Remission probability
Time (years)
Number at risk
C1 5 4 4 3 1 0 0 0 0 0 0
C2 4 3 2 1 0 0 0 0 0 0 0
C3 1 0 0 0 0 0 0 0 0 0 0
C4 2 1 1 0 0 0 0 0 0 0 0
C5 1 0 0 0 0 0 0 0 0 0 0
RD TP53 Mutation
C1 C2 C3 C4 C5
p = 0.67
Remission probability
Time (years)
Number at risk
C1 7 4 2 1 1 1 1 0 0 0 0
C2 5 1 1 0 0 0 0 0 0 0 0
C3 5 3 2 0 0 0 0 0 0 0 0
C4 4 1 1 1 0 0 0 0 0 0 0
C5 5 1 1 0 0 0 0 0 0 0 0
RD RAS Mutation
C1 C2 C3 C4 C5
p = 0.72
Remission probability
Time (years)
Number at risk
C1 6 3 3 3 2 0 0 0 0 0 0
C2 14 9 8 5 3 3 3 2 1 0 0
C3 13 7 5 5 5 2 2 2 1 0 0
C4 18 10 7 6 5 5 4 0 0 0 0
C5 6 2 2 2 2 2 1 0 0 0 0
RD TET2 Mutation
C1 C2 C3 C4 C5
p = 0.74
Remission probability
Time (years)
Number at risk
C1 7 5 5 5 4 3 0 0 0 0 0
C2 4 1 1 1 0 0 0 0 0 0 0
C3 9 4 4 4 4 3 2 2 0 0 0
C4 10 6 6 5 4 4 4 1 0 0 0
C5 3 1 1 1 0 0 0 0 0 0 0

Supplementary Figure S4. Overall survival and complete remission duration filtered by five clusters categorical variables

Kaplan-Meier plots of overall survival and complete remission duration by five clusters (C1=red, C2=blue, C3=purple, C4=green and C5=orange) were filtered by several categorical variables: presence of mutations in ASLX1, CEBPA, GATA2, DNMT3A, IDH (both total and IDH1 and IDH2), FLT3 (total and by FLT3 ITD), JAK2, RUNX1, MLL, KIT, NPM1, PTPN11, RAS, RUNX1, SRSF2, TET2, TP53, and WT1, several age groups, gender, race and ethnicity, primary vs. secondary AML, and cytogenetics stratified by risk groups (favorable, intermediary and unfavorable), complex and diploid karyotypes, and for several individual events (Inv16, Del12, t(8;21), t(11q23), -7/7q-, -5/5q-, and Trisomy8). LogRank tests were used to compare the groups.

OS Age < 70

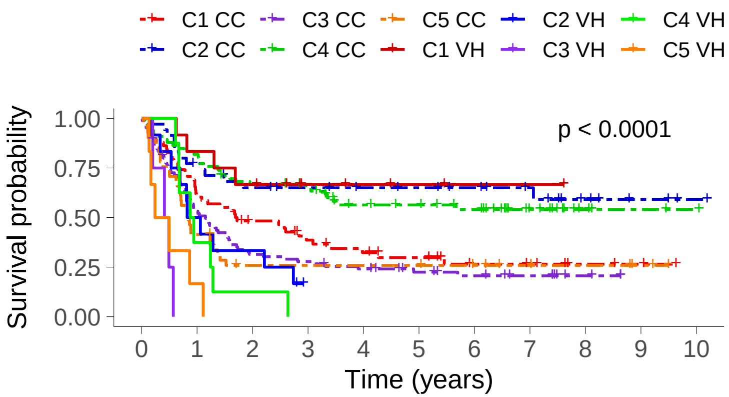

Number at risk

	0	1	2	3	4	5	6	7	8	9	10
C1 CC	58	35	26	19	15	12	7	6	3	2	0
C2 CC	35	26	21	19	17	16	14	11	6	3	1
C3 CC	85	44	26	23	19	14	11	8	3	0	0
C4 CC	66	54	43	40	30	28	24	13	4	2	1
C5 CC	41	17	9	9	9	9	7	5	4	2	0
C1 VH	12	10	8	4	3	2	1	1	0	0	0
C2 VH	12	6	4	0	0	0	0	0	0	0	0
C3 VH	4	0	0	0	0	0	0	0	0	0	0
C4 VH	8	3	1	0	0	0	0	0	0	0	0
C5 VH	6	1	0	0	0	0	0	0	0	0	0

Time (years)

OS Males

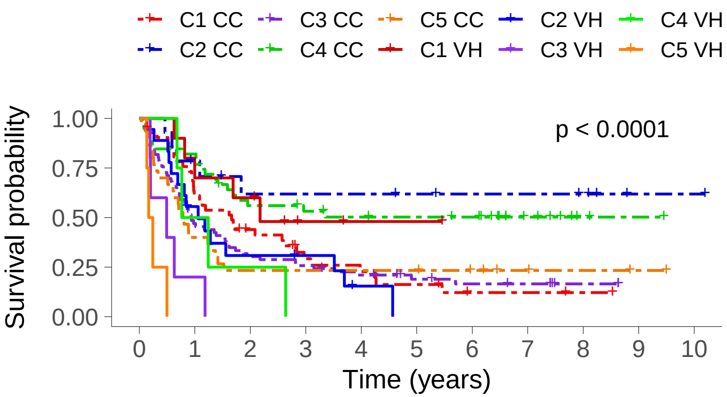

Number at risk

	0	1	2	3	4	5	6	7	8	9	10
C1 CC	42	25	16	9	7	5	2	2	1	0	0
C2 CC	14	10	7	7	7	6	5	5	4	1	1
C3 CC	66	31	20	17	13	9	7	6	2	0	0
C4 CC	39	32	21	19	17	16	15	7	2	1	0
C5 CC	30	12	7	7	7	7	5	3	2	1	0
C1 VH	10	7	6	2	1	1	0	0	0	0	0
C2 VH	18	9	5	4	1	0	0	0	0	0	0
C3 VH	5	1	0	0	0	0	0	0	0	0	0
C4 VH	4	2	1	0	0	0	0	0	0	0	0
C5 VH	4	0	0	0	0	0	0	0	0	0	0

Time (years)

OS White Race

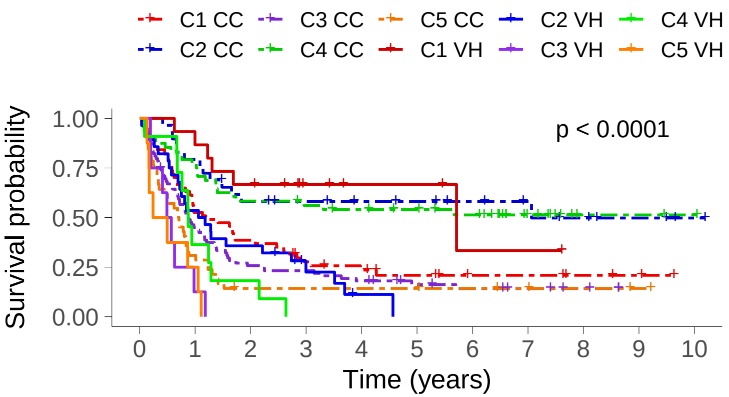

Number at risk

	0	1	2	3	4	5	6	7	8	9	10
C1 CC	57	30	22	14	11	8	5	5	3	2	0
C2 CC	29	23	16	14	12	11	9	7	5	3	1
C3 CC	79	35	20	18	14	9	7	5	3	0	0
C4 CC	48	37	28	26	24	22	19	9	2	2	1
C5 CC	42	13	5	5	5	5	4	3	2	1	0
C1 VH	15	13	10	5	3	3	1	1	0	0	0
C2 VH	28	15	10	4	1	0	0	0	0	0	0
C3 VH	8	1	0	0	0	0	0	0	0	0	0
C4 VH	11	4	2	0	0	0	0	0	0	0	0
C5 VH	8	2	0	0	0	0	0	0	0	0	0

Time (years)

OS Age > 70

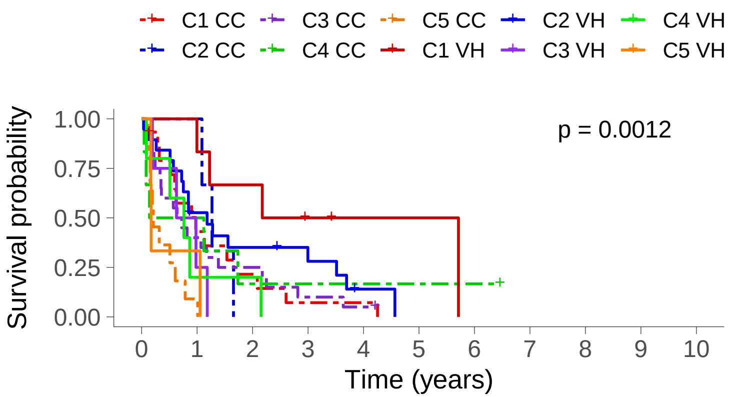

Number at risk

	0	1	2	3	4	5	6	7	8	9	10
C1 CC	15	6	3	1	1	0	0	0	0	0	0
C2 CC	3	3	0	0	0	0	0	0	0	0	0
C3 CC	20	8	5	2	1	0	0	0	0	0	0
C4 CC	6	3	1	1	1	1	1	0	0	0	0
C5 CC	11	1	0	0	0	0	0	0	0	0	0
C1 VH	6	5	4	2	1	1	0	0	0	0	0
C2 VH	19	9	6	4	1	0	0	0	0	0	0
C3 VH	4	1	0	0	0	0	0	0	0	0	0
C4 VH	5	1	1	0	0	0	0	0	0	0	0
C5 VH	3	1	0	0	0	0	0	0	0	0	0

Time (years)

OS Females

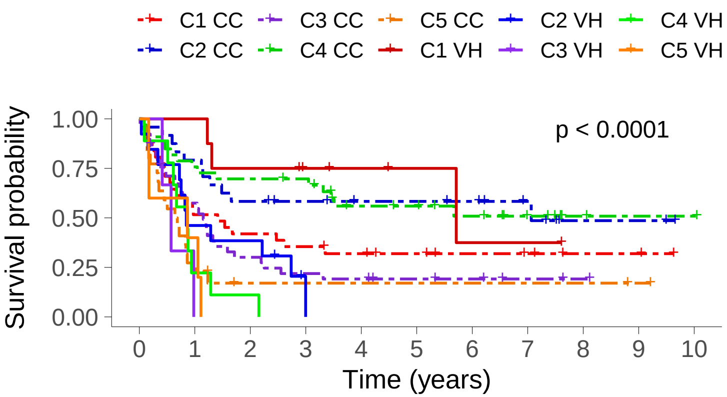

Number at risk

	0	1	2	3	4	5	6	7	8	9	10
C1 CC	31	16	13	11	9	7	5	4	2	2	0
C2 CC	24	19	14	12	10	10	9	6	2	2	0
C3 CC	39	21	11	8	7	5	4	2	1	0	0
C4 CC	33	25	23	22	14	13	10	6	2	1	1
C5 CC	22	6	2	2	2	2	2	2	2	1	0
C1 VH	8	8	6	4	3	2	1	1	0	0	0
C2 VH	13	6	5	0	0	0	0	0	0	0	0
C3 VH	3	0	0	0	0	0	0	0	0	0	0
C4 VH	9	2	1	0	0	0	0	0	0	0	0
C5 VH	5	2	0	0	0	0	0	0	0	0	0

Time (years)

OS Primary AML

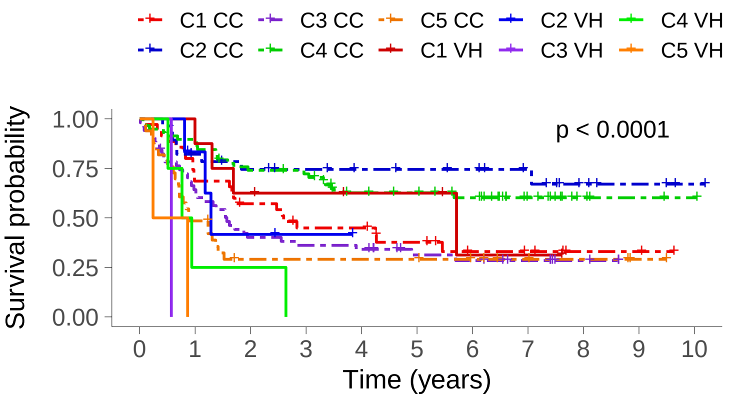

Number at risk

	0	1	2	3	4	5	6	7	8	9	10
C1 CC	35	24	19	14	14	10	6	5	2	2	0
C2 CC	28	22	19	17	15	14	13	10	5	3	1
C3 CC	51	32	20	18	17	11	10	7	3	0	0
C4 CC	58	52	42	40	30	28	24	13	4	2	1
C5 CC	33	16	8	8	8	8	6	4	3	1	0
C1 VH	8	7	5	4	3	3	1	1	0	0	0
C2 VH	6	4	2	1	0	0	0	0	0	0	0
C3 VH	1	0	0	0	0	0	0	0	0	0	0
C4 VH	4	1	1	0	0	0	0	0	0	0	0
C5 VH	2	0	0	0	0	0	0	0	0	0	0

Time (years)

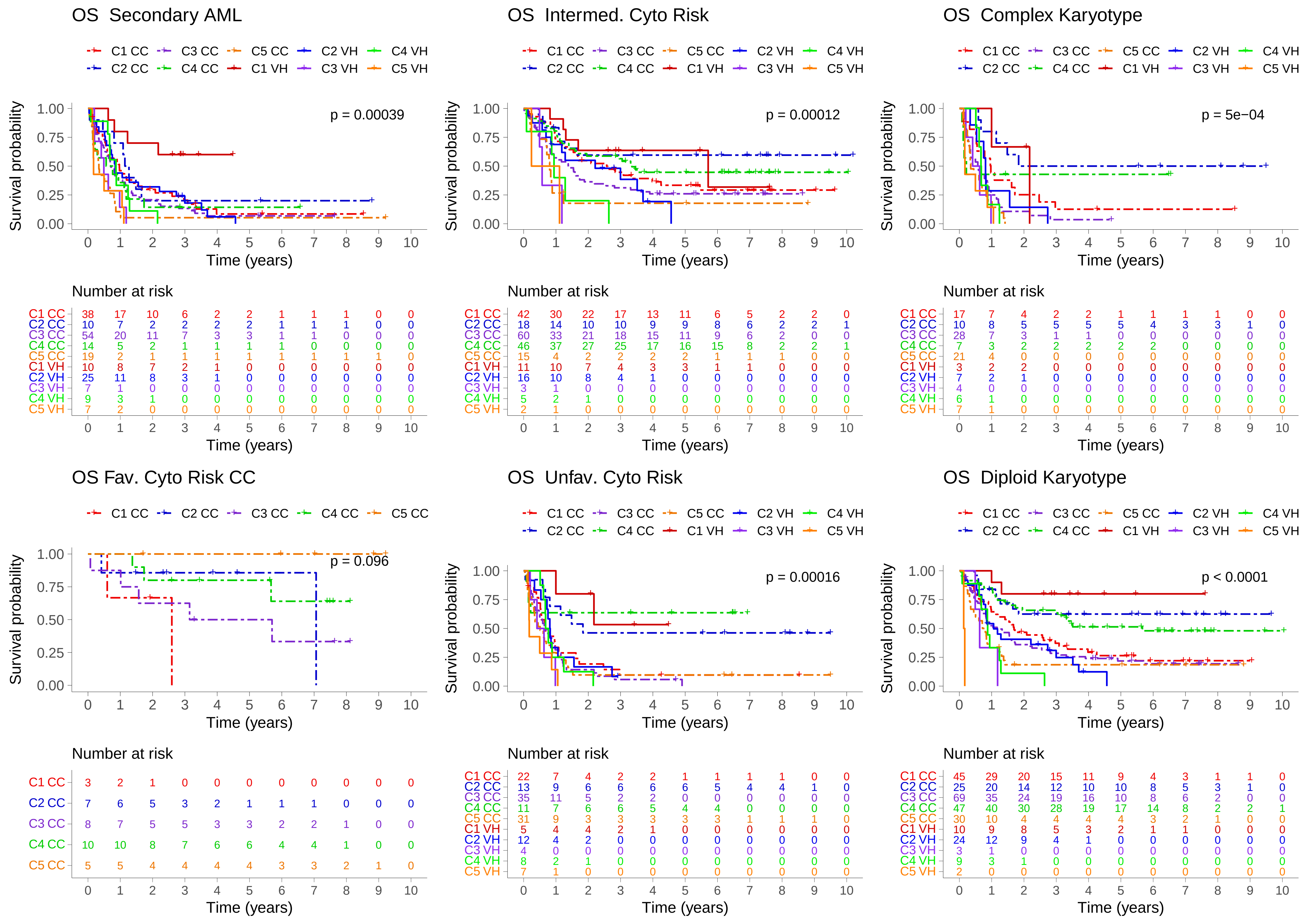
OS Secondary AML
C1 CC C3 CC C5 CC C2 VH C4 VH
C2 CC C4 CC C1 VH C3 VH C5 VH
p = 0.00039
Survival probability
1.00 0.75 0.50 0.25 0.00
0 1 2 3 4 5 6 7 8 9 10
Time (years)
Number at risk
C1 CC 38 17 10 6 2 2 1 1 1 0 0
C2 CC 10 7 2 2 2 2 1 1 1 0 0
C3 CC 54 20 11 7 3 3 1 1 0 0 0
C4 CC 14 5 2 1 1 1 1 0 0 0 0
C5 CC 19 2 1 1 1 1 1 1 1 1 0
C1 VH 10 8 7 2 1 0 0 0 0 0 0
C2 VH 25 11 8 3 1 0 0 0 0 0 0
C3 VH 7 1 0 0 0 0 0 0 0 0 0
C4 VH 9 3 1 0 0 0 0 0 0 0 0
C5 VH 7 2 0 0 0 0 0 0 0 0 0
Time (years)
OS Intermed. Cyto Risk
C1 CC C3 CC C5 CC C2 VH C4 VH
C2 CC C4 CC C1 VH C3 VH C5 VH
p = 0.00012
Survival probability
Time (years)
Number at risk
C1 CC 42 30 22 17 13 11 6 5 2 2 0
C2 CC 18 14 10 10 9 9 8 6 2 2 1
C3 CC 60 33 21 18 15 11 9 6 2 0 0
C4 CC 46 37 27 25 17 16 15 8 2 2 1
C5 CC 15 4 2 2 2 2 1 1 1 0 0
C1 VH 11 10 7 4 3 3 1 1 0 0 0
C2 VH 16 10 8 4 1 0 0 0 0 0 0
C3 VH 3 1 0 0 0 0 0 0 0 0 0
C4 VH 5 2 1 0 0 0 0 0 0 0 0
C5 VH 2 1 0 0 0 0 0 0 0 0 0
Time (years)
OS Complex Karyotype
C1 CC C3 CC C5 CC C2 VH C4 VH
C2 CC C4 CC C1 VH C3 VH C5 VH
p = 5e−04
Survival probability
Time (years)
Number at risk
C1 CC 17 7 4 2 2 1 1 1 1 0 0
C2 CC 10 8 5 5 5 5 4 3 3 1 0
C3 CC 28 7 3 1 1 0 0 0 0 0 0
C4 CC 7 3 2 2 2 2 2 0 0 0 0
C5 CC 21 4 0 0 0 0 0 0 0 0 0
C1 VH 3 2 2 0 0 0 0 0 0 0 0
C2 VH 7 2 1 0 0 0 0 0 0 0 0
C3 VH 4 0 0 0 0 0 0 0 0 0 0
C4 VH 6 1 0 0 0 0 0 0 0 0 0
C5 VH 7 1 0 0 0 0 0 0 0 0 0
Time (years)
OS Fav. Cyto Risk CC
C1 CC C2 CC C3 CC C4 CC C5 CC
p = 0.096
Survival probability
Time (years)
Number at risk
C1 CC 3 2 1 0 0 0 0 0 0 0 0
C2 CC 7 6 5 3 2 1 1 1 0 0 0
C3 CC 8 7 5 5 3 3 2 2 1 0 0
C4 CC 10 10 8 7 6 6 4 4 1 0 0
C5 CC 5 5 4 4 4 4 3 3 2 1 0
Time (years)
OS Unfav. Cyto Risk
C1 CC C3 CC C5 CC C2 VH C4 VH
C2 CC C4 CC C1 VH C3 VH C5 VH
p = 0.00016
Survival probability
Time (years)
Number at risk
C1 CC 22 7 4 2 2 1 1 1 1 0 0
C2 CC 13 9 6 6 6 6 5 4 4 1 0
C3 CC 35 11 5 2 2 0 0 0 0 0 0
C4 CC 11 7 6 6 5 4 4 0 0 0 0
C5 CC 31 9 3 3 3 3 3 1 1 1 0
C1 VH 5 4 4 2 1 0 0 0 0 0 0
C2 VH 12 4 2 0 0 0 0 0 0 0 0
C3 VH 4 0 0 0 0 0 0 0 0 0 0
C4 VH 8 2 1 0 0 0 0 0 0 0 0
C5 VH 7 1 0 0 0 0 0 0 0 0 0
Time (years)
OS Diploid Karyotype
C1 CC C3 CC C5 CC C2 VH C4 VH
C2 CC C4 CC C1 VH C3 VH C5 VH
p < 0.0001
Survival probability
Time (years)
Number at risk
C1 CC 45 29 20 15 11 9 4 3 1 1 0
C2 CC 25 20 14 12 10 10 8 5 3 1 0
C3 CC 69 35 24 19 16 10 8 6 2 0 0
C4 CC 47 40 30 28 19 17 14 8 2 2 1
C5 CC 30 10 4 4 4 4 3 2 1 0 0
C1 VH 10 9 8 5 3 2 1 1 0 0 0
C2 VH 24 12 9 4 1 0 0 0 0 0 0
C3 VH 3 1 0 0 0 0 0 0 0 0 0
C4 VH 9 3 1 0 0 0 0 0 0 0 0
C5 VH 2 0 0 0 0 0 0 0 0 0 0
Time (years)

OS −5/5q−

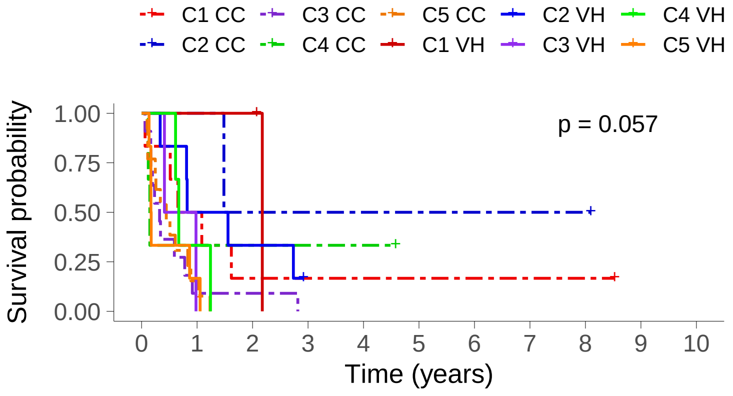

Number at risk

	0	1	2	3	4	5	6	7	8	9	10
C1 CC	6	3	1	1	1	1	1	1	1	0	0
C2 CC	2	2	1	1	1	1	1	1	1	0	0
C3 CC	11	1	1	0	0	0	0	0	0	0	0
C4 CC	3	1	1	1	1	0	0	0	0	0	0
C5 CC	13	2	0	0	0	0	0	0	0	0	0
C1 VH	2	2	2	0	0	0	0	0	0	0	0
C2 VH	6	3	2	0	0	0	0	0	0	0	0
C3 VH	2	0	0	0	0	0	0	0	0	0	0
C4 VH	3	1	0	0	0	0	0	0	0	0	0
C5 VH	6	1	0	0	0	0	0	0	0	0	0

Time (years)

OS FLT3 Mutation

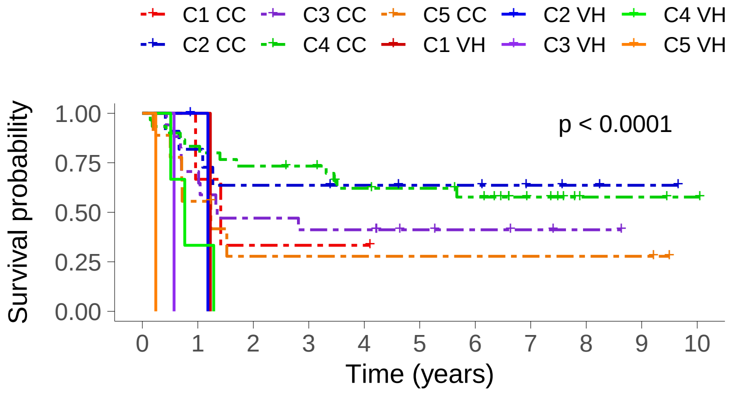

Number at risk

	0	1	2	3	4	5	6	7	8	9	10
C1 CC	3	2	1	1	1	0	0	0	0	0	0
C2 CC	11	9	7	7	6	5	5	3	2	1	0
C3 CC	17	12	8	7	7	4	3	2	1	0	0
C4 CC	30	25	22	21	16	15	13	7	2	2	1
C5 CC	9	5	2	2	2	2	2	2	2	2	0
C1 VH	1	1	0	0	0	0	0	0	0	0	0
C2 VH	2	1	0	0	0	0	0	0	0	0	0
C3 VH	1	0	0	0	0	0	0	0	0	0	0
C4 VH	3	1	0	0	0	0	0	0	0	0	0
C5 VH	1	0	0	0	0	0	0	0	0	0	0

Time (years)

OS RUNX1 Mutation

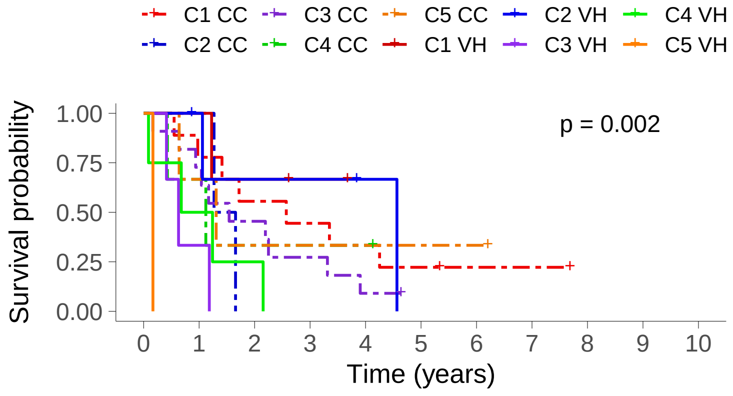

Number at risk

	0	1	2	3	4	5	6	7	8	9	10
C1 CC	9	7	5	4	3	2	1	1	0	0	0
C2 CC	2	2	0	0	0	0	0	0	0	0	0
C3 CC	11	8	5	3	1	0	0	0	0	0	0
C4 CC	3	2	1	1	1	0	0	0	0	0	0
C5 CC	3	2	1	1	1	1	1	0	0	0	0
C1 VH	3	3	2	1	0	0	0	0	0	0	0
C2 VH	4	3	2	2	1	0	0	0	0	0	0
C3 VH	3	1	0	0	0	0	0	0	0	0	0
C4 VH	4	2	1	0	0	0	0	0	0	0	0
C5 VH	1	0	0	0	0	0	0	0	0	0	0

Time (years)

OS DNMT3 Mutation

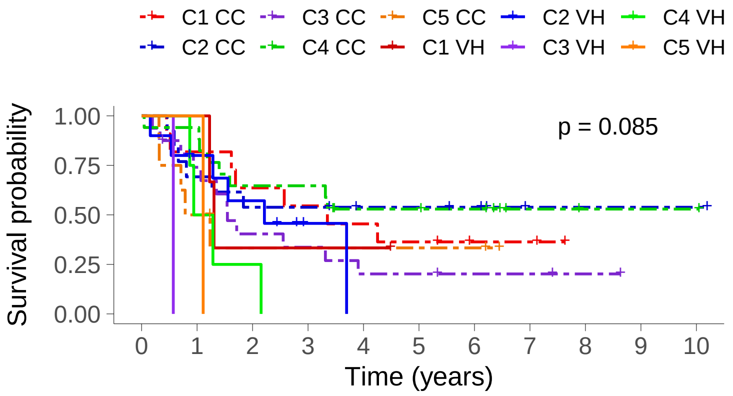

Number at risk

	0	1	2	3	4	5	6	7	8	9	10
C1 CC	11	9	7	6	5	4	2	2	0	0	0
C2 CC	13	9	7	7	5	5	4	1	1	1	1
C3 CC	16	11	6	5	3	3	2	2	1	0	0
C4 CC	17	16	11	11	7	7	6	2	1	1	1
C5 CC	8	4	2	2	2	2	2	0	0	0	0
C1 VH	3	3	1	1	1	0	0	0	0	0	0
C2 VH	10	7	5	1	0	0	0	0	0	0	0
C3 VH	1	0	0	0	0	0	0	0	0	0	0
C4 VH	4	2	1	0	0	0	0	0	0	0	0
C5 VH	1	1	0	0	0	0	0	0	0	0	0

Time (years)

OS FLT3_ITD Mutation

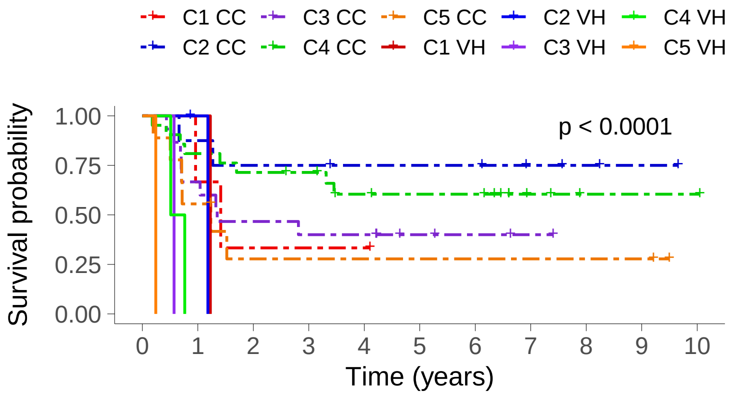

Number at risk

	0	1	2	3	4	5	6	7	8	9	10
C1 CC	3	2	1	1	1	0	0	0	0	0	0
C2 CC	8	7	6	6	5	5	5	3	2	1	0
C3 CC	15	10	7	6	6	3	2	1	0	0	0
C4 CC	21	17	15	14	10	9	9	3	1	1	1
C5 CC	9	5	2	2	2	2	2	2	2	2	0
C1 VH	1	1	0	0	0	0	0	0	0	0	0
C2 VH	2	1	0	0	0	0	0	0	0	0	0
C3 VH	1	0	0	0	0	0	0	0	0	0	0
C4 VH	2	0	0	0	0	0	0	0	0	0	0
C5 VH	1	0	0	0	0	0	0	0	0	0	0

Time (years)

OS TET2 Mutation

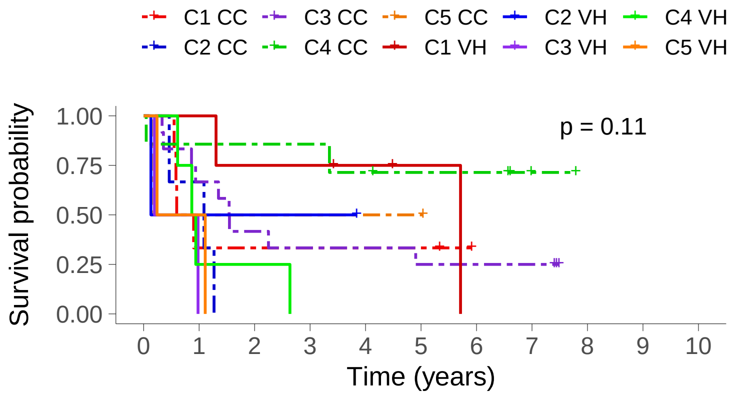

Number at risk

	0	1	2	3	4	5	6	7	8	9	10
C1 CC	6	2	2	2	2	2	0	0	0	0	0
C2 CC	3	2	0	0	0	0	0	0	0	0	0
C3 CC	12	8	5	4	4	3	3	3	0	0	0
C4 CC	7	6	6	6	5	4	4	1	0	0	0
C5 CC	2	1	1	1	1	1	0	0	0	0	0
C1 VH	4	4	3	3	2	1	0	0	0	0	0
C2 VH	2	1	1	1	0	0	0	0	0	0	0
C3 VH	2	0	0	0	0	0	0	0	0	0	0
C4 VH	4	1	1	0	0	0	0	0	0	0	0
C5 VH	2	1	0	0	0	0	0	0	0	0	0

Time (years)

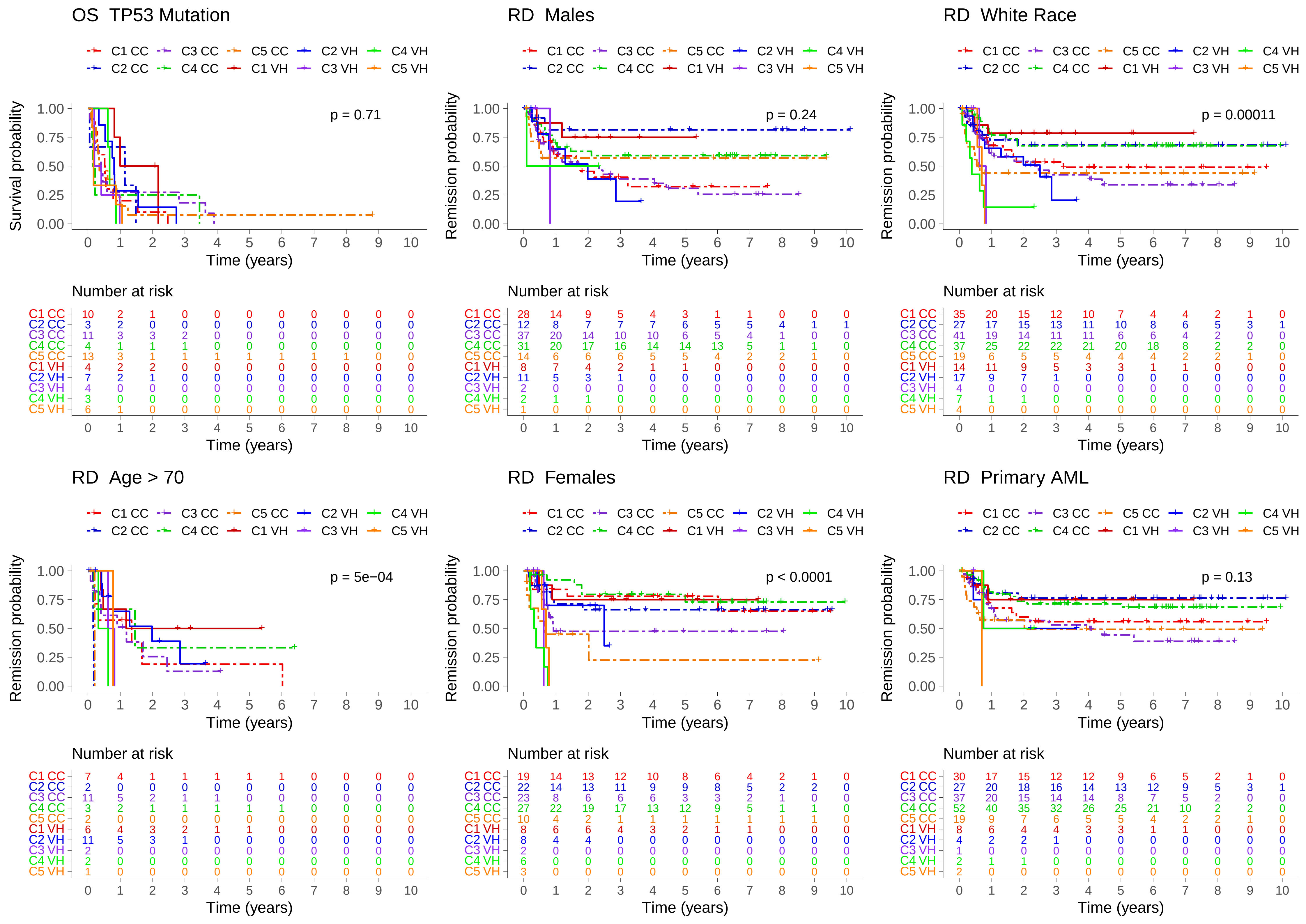
OS TP53 Mutation
C1 CC C3 CC C5 CC C2 VH C4 VH
C2 CC C4 CC C1 VH C3 VH C5 VH
p = 0.71
Survival probability
1.00 0.75 0.50 0.25 0.00
0 1 2 3 4 5 6 7 8 9 10
Time (years)
Number at risk
C1 CC 10 2 1 0 0 0 0 0 0 0 0
C2 CC 3 2 0 0 0 0 0 0 0 0 0
C3 CC 11 3 3 2 0 0 0 0 0 0 0
C4 CC 4 1 1 1 0 0 0 0 0 0 0
C5 CC 13 3 1 1 1 1 1 1 1 0 0
C1 VH 4 2 2 0 0 0 0 0 0 0 0
C2 VH 7 2 1 0 0 0 0 0 0 0 0
C3 VH 4 0 0 0 0 0 0 0 0 0 0
C4 VH 3 0 0 0 0 0 0 0 0 0 0
C5 VH 6 1 0 0 0 0 0 0 0 0 0
Time (years)
RD Males
C1 CC C3 CC C5 CC C2 VH C4 VH
C2 CC C4 CC C1 VH C3 VH C5 VH
p = 0.24
Remission probability
1.00 0.75 0.50 0.25 0.00
0 1 2 3 4 5 6 7 8 9 10
Time (years)
Number at risk
C1 CC 28 14 9 5 4 3 1 1 0 0 0
C2 CC 12 8 7 7 7 6 5 5 4 1 1
C3 CC 37 20 14 10 10 6 5 4 1 0 0
C4 CC 31 20 17 16 14 14 13 5 1 1 0
C5 CC 14 6 6 6 5 5 4 2 2 1 0
C1 VH 8 7 4 2 1 1 0 0 0 0 0
C2 VH 11 5 3 1 0 0 0 0 0 0 0
C3 VH 2 0 0 0 0 0 0 0 0 0 0
C4 VH 2 1 1 0 0 0 0 0 0 0 0
C5 VH 1 0 0 0 0 0 0 0 0 0 0
Time (years)
RD White Race
C1 CC C3 CC C5 CC C2 VH C4 VH
C2 CC C4 CC C1 VH C3 VH C5 VH
p = 0.00011
Remission probability
1.00 0.75 0.50 0.25 0.00
0 1 2 3 4 5 6 7 8 9 10
Time (years)
Number at risk
C1 CC 35 20 15 12 10 7 4 4 2 1 0
C2 CC 27 17 15 13 11 10 8 6 5 3 1
C3 CC 41 19 14 11 11 6 6 4 2 0 0
C4 CC 37 25 22 22 21 20 18 8 2 2 0
C5 CC 19 6 5 5 4 4 4 2 2 1 0
C1 VH 14 11 9 5 3 3 1 1 0 0 0
C2 VH 17 9 7 1 0 0 0 0 0 0 0
C3 VH 4 0 0 0 0 0 0 0 0 0 0
C4 VH 7 1 1 0 0 0 0 0 0 0 0
C5 VH 4 0 0 0 0 0 0 0 0 0 0
Time (years)
RD Age > 70
C1 CC C3 CC C5 CC C2 VH C4 VH
C2 CC C4 CC C1 VH C3 VH C5 VH
p = 5e−04
Remission probability
1.00 0.75 0.50 0.25 0.00
0 1 2 3 4 5 6 7 8 9 10
Time (years)
Number at risk
C1 CC 7 4 1 1 1 1 1 0 0 0 0
C2 CC 2 0 0 0 0 0 0 0 0 0 0
C3 CC 11 5 2 1 1 0 0 0 0 0 0
C4 CC 3 2 1 1 1 1 1 0 0 0 0
C5 CC 2 0 0 0 0 0 0 0 0 0 0
C1 VH 6 4 3 2 1 1 0 0 0 0 0
C2 VH 11 5 3 1 0 0 0 0 0 0 0
C3 VH 2 0 0 0 0 0 0 0 0 0 0
C4 VH 2 0 0 0 0 0 0 0 0 0 0
C5 VH 1 0 0 0 0 0 0 0 0 0 0
Time (years)
RD Females
C1 CC C3 CC C5 CC C2 VH C4 VH
C2 CC C4 CC C1 VH C3 VH C5 VH
p < 0.0001
Remission probability
1.00 0.75 0.50 0.25 0.00
0 1 2 3 4 5 6 7 8 9 10
Time (years)
Number at risk
C1 CC 19 14 13 12 10 8 6 4 2 1 0
C2 CC 22 14 13 11 9 9 8 5 2 2 0
C3 CC 23 8 6 6 6 3 3 2 1 0 0
C4 CC 27 22 19 17 13 12 9 5 1 1 0
C5 CC 10 4 2 1 1 1 1 1 1 1 0
C1 VH 8 6 6 4 3 2 1 1 0 0 0
C2 VH 8 4 4 0 0 0 0 0 0 0 0
C3 VH 2 0 0 0 0 0 0 0 0 0 0
C4 VH 6 0 0 0 0 0 0 0 0 0 0
C5 VH 3 0 0 0 0 0 0 0 0 0 0
Time (years)
RD Primary AML
C1 CC C3 CC C5 CC C2 VH C4 VH
C2 CC C4 CC C1 VH C3 VH C5 VH
p = 0.13
Remission probability
1.00 0.75 0.50 0.25 0.00
0 1 2 3 4 5 6 7 8 9 10
Time (years)
Number at risk
C1 CC 30 17 15 12 12 9 6 5 2 1 0
C2 CC 27 20 18 16 14 13 12 9 5 3 1
C3 CC 37 20 15 14 14 8 7 5 2 0 0
C4 CC 52 40 35 32 26 25 21 10 2 2 0
C5 CC 19 9 7 6 5 5 4 2 2 1 0
C1 VH 8 6 4 4 3 3 1 1 0 0 0
C2 VH 4 2 2 1 0 0 0 0 0 0 0
C3 VH 1 0 0 0 0 0 0 0 0 0 0
C4 VH 2 1 1 0 0 0 0 0 0 0 0
C5 VH 2 0 0 0 0 0 0 0 0 0 0
Time (years)

RD Secondary AML

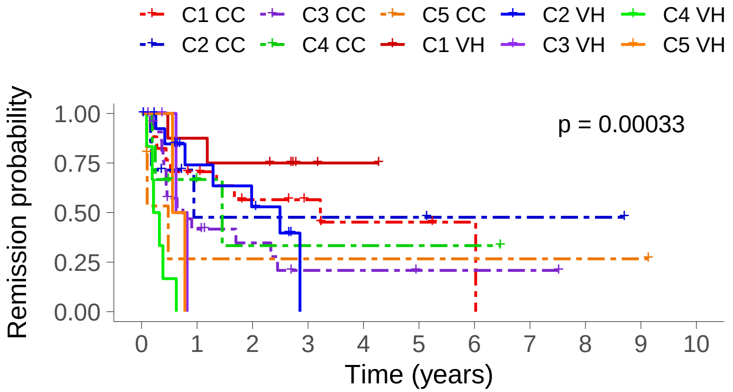

Number at risk

	0	1	2	3	4	5	6	7	8	9	10
C1 CC	17	11	7	5	2	2	1	0	0	0	0
C2 CC	7	2	2	2	2	2	1	1	1	0	0
C3 CC	23	8	5	2	2	1	1	1	0	0	0
C4 CC	6	2	1	1	1	1	1	0	0	0	0
C5 CC	5	1	1	1	1	1	1	1	1	1	0
C1 VH	8	7	6	2	1	0	0	0	0	0	0
C2 VH	15	7	5	0	0	0	0	0	0	0	0
C3 VH	3	0	0	0	0	0	0	0	0	0	0
C4 VH	6	0	0	0	0	0	0	0	0	0	0
C5 VH	2	0	0	0	0	0	0	0	0	0	0

Time (years)

RD Intermed. Cyto Risk

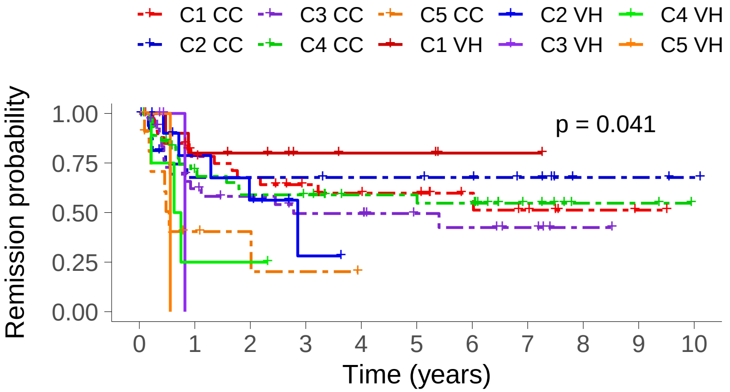

Number at risk

	0	1	2	3	4	5	6	7	8	9	10
C1 CC	33	23	19	15	12	11	7	5	2	1	0
C2 CC	16	10	10	10	9	9	8	6	2	2	1
C3 CC	33	17	14	11	11	7	6	4	1	0	0
C4 CC	37	23	19	18	14	14	13	6	2	2	0
C5 CC	11	3	2	1	0	0	0	0	0	0	0
C1 VH	10	8	7	4	3	3	1	1	0	0	0
C2 VH	12	7	5	1	0	0	0	0	0	0	0
C3 VH	3	0	0	0	0	0	0	0	0	0	0
C4 VH	4	1	1	0	0	0	0	0	0	0	0
C5 VH	2	0	0	0	0	0	0	0	0	0	0

Time (years)

RD Complex Karyotype

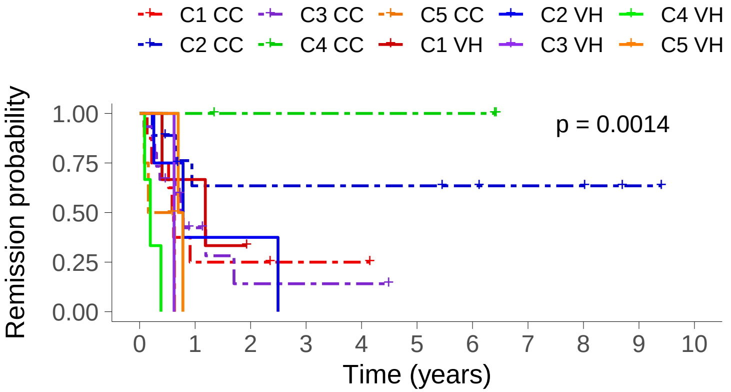

Number at risk

	0	1	2	3	4	5	6	7	8	9	10
C1 CC	8	2	2	1	1	0	0	0	0	0	0
C2 CC	9	5	5	5	5	5	4	3	3	1	0
C3 CC	15	4	1	1	1	0	0	0	0	0	0
C4 CC	3	3	2	2	2	2	2	0	0	0	0
C5 CC	4	0	0	0	0	0	0	0	0	0	0
C1 VH	3	2	0	0	0	0	0	0	0	0	0
C2 VH	4	1	1	0	0	0	0	0	0	0	0
C3 VH	1	0	0	0	0	0	0	0	0	0	0
C4 VH	3	0	0	0	0	0	0	0	0	0	0
C5 VH	2	0	0	0	0	0	0	0	0	0	0

Time (years)

RD Fav. Cyto Risk CC

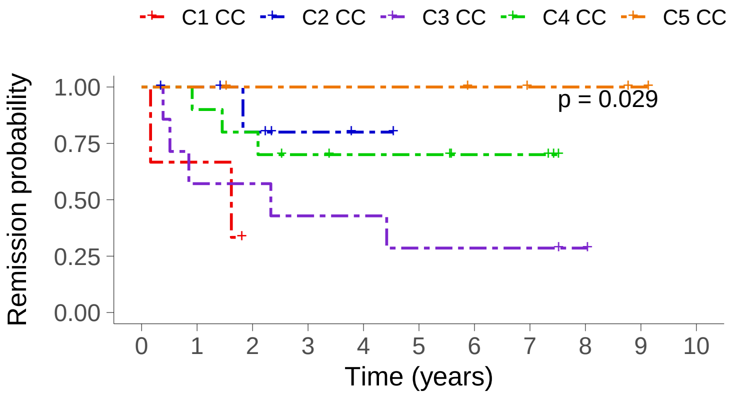

Number at risk

	0	1	2	3	4	5	6	7	8	9	10
C1 CC	3	2	0	0	0	0	0	0	0	0	0
C2 CC	7	6	4	2	1	0	0	0	0	0	0
C3 CC	7	4	4	3	3	2	2	2	1	0	0
C4 CC	10	9	8	6	5	5	3	3	0	0	0
C5 CC	5	5	4	4	4	4	3	2	2	1	0

Time (years)

RD Unfav. Cyto Risk

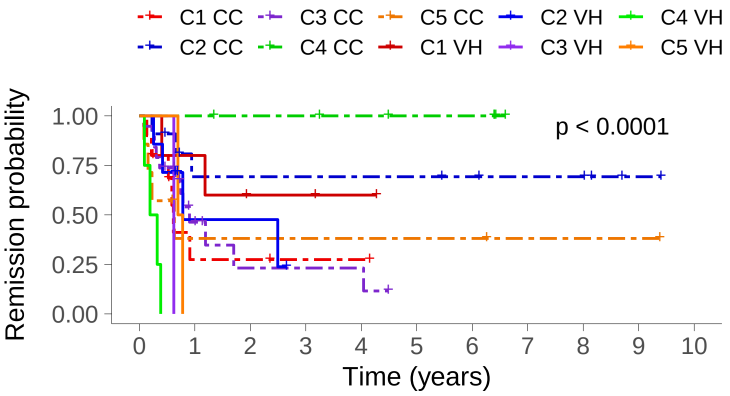

Number at risk

	0	1	2	3	4	5	6	7	8	9	10
C1 CC	10	2	2	1	1	0	0	0	0	0	0
C2 CC	11	6	6	6	6	6	5	4	4	1	0
C3 CC	19	6	2	2	2	0	0	0	0	0	0
C4 CC	7	7	6	6	5	4	4	0	0	0	0
C5 CC	7	2	2	2	2	2	2	1	1	1	0
C1 VH	5	4	2	2	1	0	0	0	0	0	0
C2 VH	7	2	2	0	0	0	0	0	0	0	0
C3 VH	1	0	0	0	0	0	0	0	0	0	0
C4 VH	4	0	0	0	0	0	0	0	0	0	0
C5 VH	2	0	0	0	0	0	0	0	0	0	0

Time (years)

RD −5/5q−

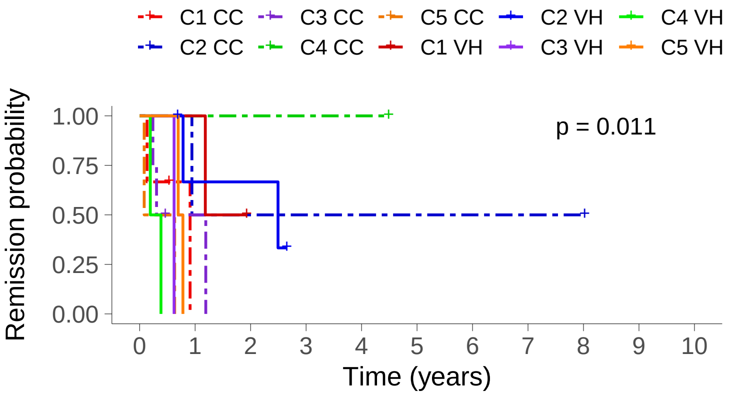

Number at risk

	0	1	2	3	4	5	6	7	8	9	10
C1 CC	3	0	0	0	0	0	0	0	0	0	0
C2 CC	2	1	1	1	1	1	1	1	1	0	0
C3 CC	4	1	0	0	0	0	0	0	0	0	0
C4 CC	1	1	1	1	1	0	0	0	0	0	0
C5 CC	2	0	0	0	0	0	0	0	0	0	0
C1 VH	2	2	0	0	0	0	0	0	0	0	0
C2 VH	4	2	2	0	0	0	0	0	0	0	0
C3 VH	1	0	0	0	0	0	0	0	0	0	0
C4 VH	2	0	0	0	0	0	0	0	0	0	0
C5 VH	2	0	0	0	0	0	0	0	0	0	0

Time (years)

RD DNMT3 Mutation

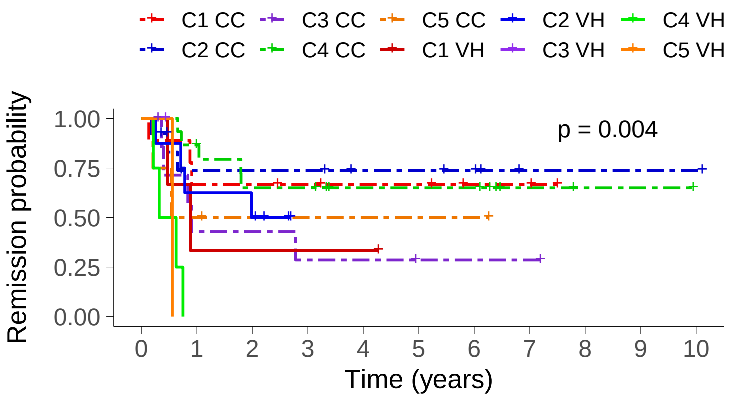

Number at risk

	0	1	2	3	4	5	6	7	8	9	10
C1 CC	9	6	6	5	4	4	2	2	0	0	0
C2 CC	13	7	7	7	5	5	4	1	1	1	1
C3 CC	8	3	3	2	2	1	1	1	0	0	0
C4 CC	15	12	9	9	6	6	6	2	1	1	0
C5 CC	4	2	1	1	1	1	1	0	0	0	0
C1 VH	3	1	1	1	1	0	0	0	0	0	0
C2 VH	8	5	4	0	0	0	0	0	0	0	0
C3 VH	1	0	0	0	0	0	0	0	0	0	0
C4 VH	4	0	0	0	0	0	0	0	0	0	0
C5 VH	1	0	0	0	0	0	0	0	0	0	0

Time (years)

RD TET2 Mutation

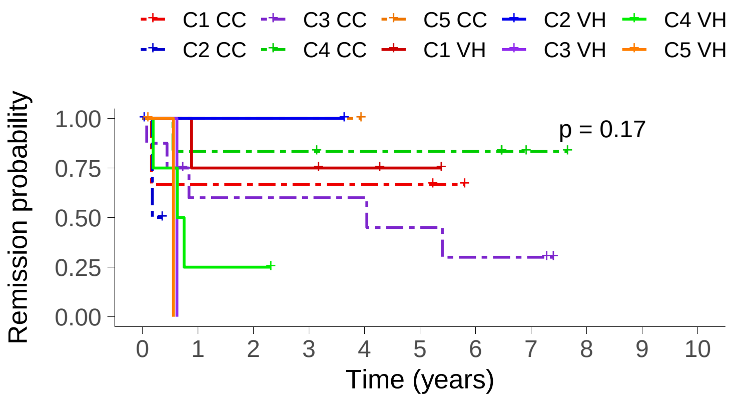

Number at risk

	0	1	2	3	4	5	6	7	8	9	10
C1 CC	3	2	2	2	2	2	0	0	0	0	0
C2 CC	2	0	0	0	0	0	0	0	0	0	0
C3 CC	8	4	4	4	4	3	2	2	0	0	0
C4 CC	6	5	5	5	4	4	4	1	0	0	0
C5 CC	1	1	1	1	0	0	0	0	0	0	0
C1 VH	4	3	3	3	2	1	0	0	0	0	0
C2 VH	2	1	1	1	0	0	0	0	0	0	0
C3 VH	1	0	0	0	0	0	0	0	0	0	0
C4 VH	4	1	1	0	0	0	0	0	0	0	0
C5 VH	2	0	0	0	0	0	0	0	0	0	0

Time (years)

RD FLT3 Mutation

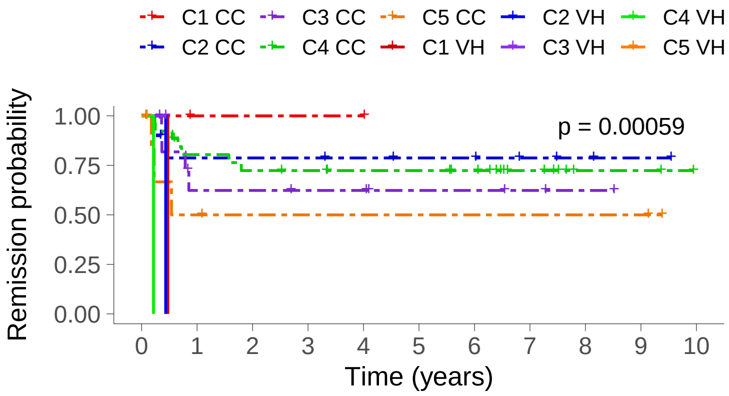

Number at risk

	0	1	2	3	4	5	6	7	8	9	10
C1 CC	2	1	1	1	1	0	0	0	0	0	0
C2 CC	10	7	7	7	6	5	5	3	2	1	0
C3 CC	12	6	6	5	5	3	3	2	1	0	0
C4 CC	27	20	18	17	15	15	13	7	2	2	0
C5 CC	7	3	2	2	2	2	2	2	2	2	0
C1 VH	1	0	0	0	0	0	0	0	0	0	0
C2 VH	1	0	0	0	0	0	0	0	0	0	0
C3 VH	1	0	0	0	0	0	0	0	0	0	0
C4 VH	1	0	0	0	0	0	0	0	0	0	0
C5 VH	1	0	0	0	0	0	0	0	0	0	0

Time (years)

RD TP53 Mutation

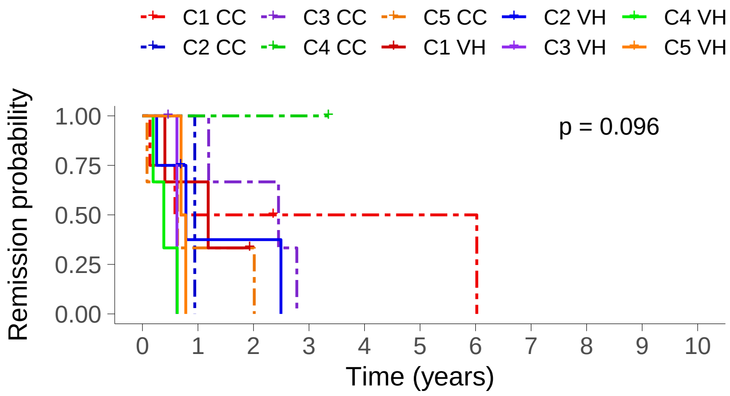

Number at risk

	0	1	2	3	4	5	6	7	8	9	10
C1 CC	4	2	2	1	1	1	1	0	0	0	0
C2 CC	1	0	0	0	0	0	0	0	0	0	0
C3 CC	4	3	2	0	0	0	0	0	0	0	0
C4 CC	1	1	1	1	0	0	0	0	0	0	0
C5 CC	3	1	1	0	0	0	0	0	0	0	0
C1 VH	3	2	0	0	0	0	0	0	0	0	0
C2 VH	4	1	1	0	0	0	0	0	0	0	0
C3 VH	1	0	0	0	0	0	0	0	0	0	0
C4 VH	3	0	0	0	0	0	0	0	0	0	0
C5 VH	2	0	0	0	0	0	0	0	0	0	0

Time (years)

Supplementary Figure S5. Overall survival and complete remission duration by ten clusters filtered by categorical variables

Kaplan-Meier plots of overall survival and complete remission duration by cluster and treatment modality (VH=solid line, CC=dashed line; C1=red, C2=blue, C3=purple, C4=green and C5=orange) were filtered by several categorical variables: presence of DNMT3A, FLT3 [total and by FLT3 ITD], RUNX1, TET2, TP53 mutations, age <70 years, age >70 years, males, females, white race, primary and secondary AML, unfavorable and intermediary/favorable cytogenetic risks, -5/5q-, and complex and diploid karyotypes. LogRank tests were used to compare the groups.

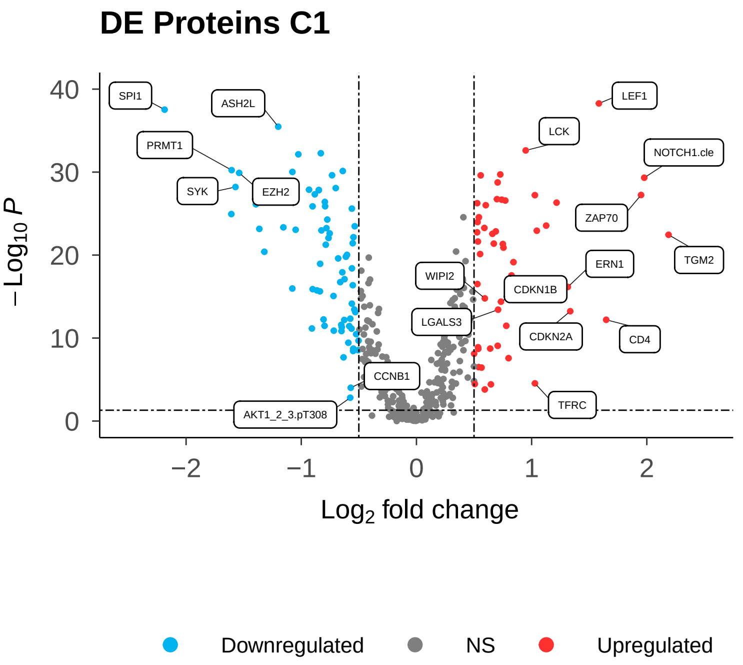
DE Proteins C1
−Log10 P
Log2 fold change
SPI1
ASH2L
PRMT1
SYK
EZH2
LEF1
LCK
NOTCH1.cle
ZAP70
TGM2
ERN1
WIPI2
CDKN1B
LGALS3
CDKN2A
CD4
CCNB1
TFRC
AKT1_2_3.pT308
Downregulated
NS
Upregulated

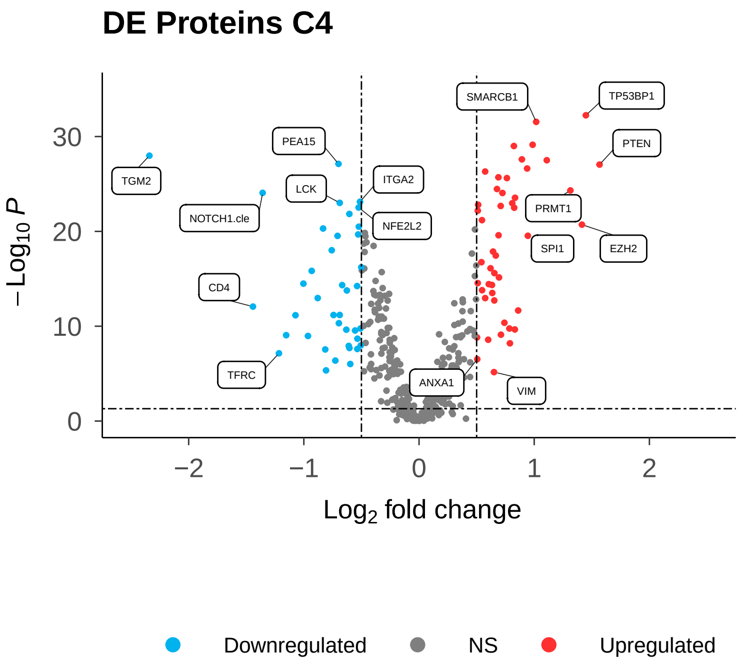
DE Proteins C4
−Log10 P
Log2 fold change
TGM2
PEA15
LCK
NOTCH1.cle
ITGA2
NFE2L2
SMARCB1
TP53BP1
PTEN
PRMT1
SPI1
EZH2
CD4
TFRC
ANXA1
VIM
Downregulated
NS
Upregulated

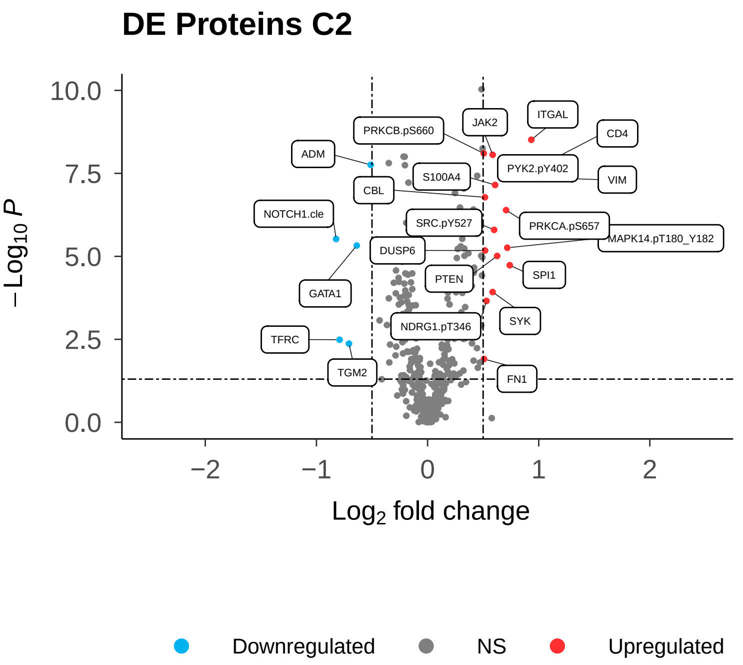
DE Proteins C2
−Log10 P
Log2 fold change
PRKCB.pS660
JAK2
ITGAL
CD4
ADM
S100A4
PYK2.pY402
VIM
CBL
NOTCH1.cle
SRC.pY527
PRKCA.pS657
MAPK14.pT180_Y182
DUSP6
SPI1
GATA1
PTEN
SYK
NDRG1.pT346
TFRC
TGM2
FN1
Downregulated
NS
Upregulated

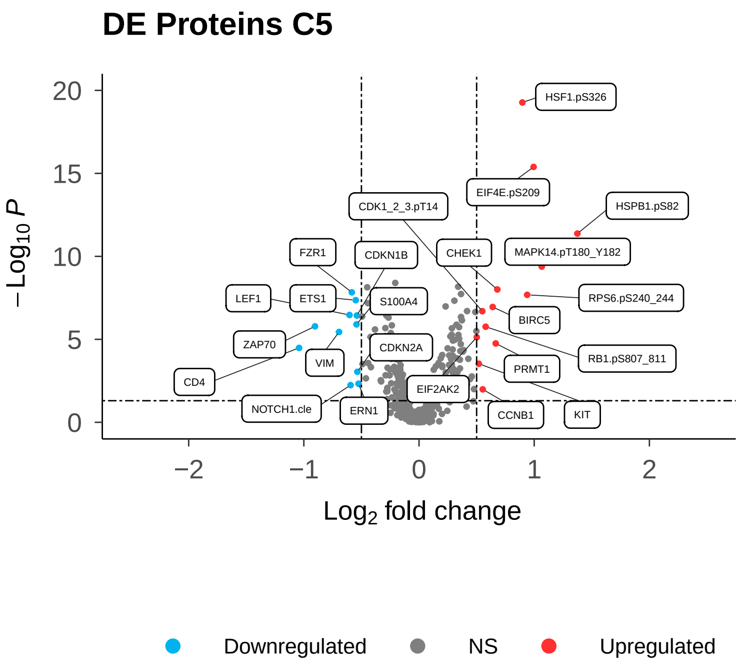
DE Proteins C5
−Log10 P
Log2 fold change
HSF1.pS326
EIF4E.pS209
CDK1_2_3.pT14
HSPB1.pS82
FZR1
CDKN1B
CHEK1
MAPK14.pT180_Y182
LEF1
ETS1
S100A4
RPS6.pS240_244
BIRC5
ZAP70
CDKN2A
VIM
RB1.pS807_811
CD4
PRMT1
EIF2AK2
NOTCH1.cle
ERN1
CCNB1
KIT
Downregulated
NS
Upregulated

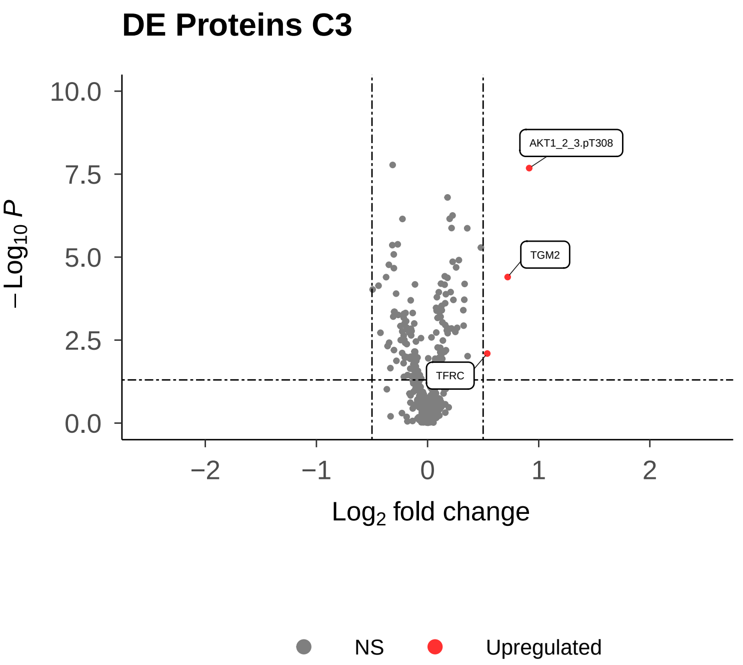
DE Proteins C3
−Log10 P
Log2 fold change
AKT1_2_3.pT308
TGM2
TFRC
NS
Upregulated

Supplementary Figure S6. Up- and down-regulated proteins of each cluster

Volcano plots demonstrating the up- and down-regulated proteins of each cluster. Each point represents an individual protein, the y-axis shows the -Log10(adjusted p-value) of the comparison of a given cluster vs. all the others, and the x-axis shows the mean Log2 fold change (LFC) value for each cluster (highlighted in the panel title). A p-value < 0.05 cutoff and a LFC threshold of 0.5 was used to determine differentially expressed proteins. Points are colored according to the following color code: red=up-regulated, blue=downregulated, grey=non-significant, as shown in the figure legend.

Supplementary Table S1. Antibodies used in the Reverse-Phase Protein Arrays

Antibody name	HUGO	Modified HUGO	R2 (RPPA vs WB)	Host	company	catalog#	lot#	1st Ab dilution	2nd Ab dilution	RRID
4EBP1	EIF4EBP1	EIF4EBP1	>0.7	Rabbit	Cell Signaling	9452	9	1000	10000	AB_331692
4EBP1 (P-S65)	EIF4EBP1-phospho Ser65	EIF4EBP1 p65	>0.7	Rabbit	Cell Signaling	9456	2	400	10000	AB_823413
14-3-3-ε	YWHAE	YWHAE	0.5-0.7	mouse	Santa Cruz	sc-23957	A1205	100	10000	AB_626619
53BP1	53BP1	TP53BP1	>0.7	Rabbit	Cell Signaling	4937	3	300	10000	AB_10694558
Abl-c	ABL1	ABL1	>0.7	Rabbit	cell signaling	2862	16	100	10000	AB_2257757
c-Abl (phospho Y412)	Abl_pY412	Abl_pY412	0.5-0.7	Rabbit	Cell Signaling	2865	3	100	10000	AB_331381
Acetyl CoA Carboxylase 1	ACACA	ACACA	0.5-0.7	Rabbit	abcam	ab45174	GR79853-13	5000	10000	AB_867475
Acetyl CoA Carboxylase (P-S79)	ACACA/ACACB	ACACA/B	>0.7	Rabbit	Cell Signaling	3661	4	4000	10000	AB_330337
ADAR1	ADAR	ADAR	>0.7	mouse	abcam	ab88574	GR191241-1	200	10000	AB_2222658
adrenomedullin	ADM	ADM	0.5-0.7	Rabbit	ThermoFisher	PA5-103107	WL3457592	2000	10000	AB_2852477
AIF	AIFM1	AIFM1	0.5-0.7	mouse	santa cruz	sc-13116	C1306	500	10000	AB_626654
AKR1C3 (Aldo-Keto Reductase Family 1, Member C3)	ARR1C3	ARR1C3	0.5-0.7	mouse	marina-Dr.wang			1000	10000	N/A
AKT	AKT1/AKT2/AKT3	AKT1/2/3	>0.7	Rabbit	cell signaling	9272	22	400	10000	AB_329827
AKT1	AKT1	AKT1	>0.7	mouse	cell signaling	2967	11	1000	10000	AB_331160
AKT2	AKT2	AKT2	>0.7	Rabbit	cell signaling	2962	2	100	10000	AB_329872
AKT3	AKT3	AKT3	>0.7	Rabbit	cell signaling	4059	2	75	10000	AB_2225351
AKT-P308(Thr)	AKT1/AKT2/AKT3-phospho Thr308	AKT1/2/3 p308	0.5-0.7	Rabbit	cell signaling	9275	25	200	10000	AB_329828
AKT-P473(Ser)	AKT1/AKT2/AKT3-phospho ser473	AKT1/2/3 p473	0.5-0.7	Rabbit	cell signaling	9271	12	100	10000	AB_329825
Ambra1 (phospho S52)	Ambra1_phosohoS52	Ambra1_pS52	0.5-0.7	Rabbit	Millipore	ABC80	3440802	500	10000	AB_2750901
AMPKα	PRKAA1/PRKAA2	PRKAA1/2	0.5-0.7	Rabbit	cell signaling	2532	19	500	10000	AB_330331
AMPKα P(Thr172)	PRKAA1/PRKAA2-phospho Thr172	PRKAA1/2 p172	0.5-0.7	Rabbit	cell signaling	2535	21	1000	10000	AB_331250
AMPKα2-phospho Ser345	PRKAA2-phospho Ser345	PRKAA2 p345	>0.7	Rabbit	abcam	ab129081	Y111002CS	1000	10000	AB_11155357
Androgen Receptor	AR	AR	0.5-0.7	Rabbit	cell signaling	5153	? from Michele, Montreal	200	10000	AB_10691711
Annexin I (Lipocortin-1, Calpactin II)	ANXA1	ANXA1	>0.7	mouse	BD Biosciences	610066	6273584	10000	10000	AB_397477
Annexin VII	ANAX7	ANAX7	>0.7	mouse	BD Biosciences	610668	6302833	250	10000	AB_397995
ARC	ARC	ARC	0.5-0.7	Rabbit	Novus	NBP2-41753	2081-1304	2000	10000	AB_2938523
ARID1A	ARID1A	ARID1A	0.5-0.7	Rabbit	Sigma	HPA005456	k106722	4000	10000	AB_1078205
Ash2L	ASH2L	ASH2L	>0.7	Rabbit	Cell Signaling	5019	? from Jill Butler	5000	10000	AB_1950350
ASNS	ASNS	ASNS	0.5-0.7	Rabbit	Sigma	HPA029318	? from Gautam Borthakur	800	10000	AB_10602389
ASS1	ASS1	ASS1	0.5-0.7	mouse	Palars	uncommercial,	91215	300	10000	N/A
ATF3	ATF3	ATF3	>0.7	Rabbit	abcam	ab87213	GR1422-2	300	10000	AB_1951498
Atg3	ATG3	ATG3	>0.7	Rabbit	cell signaling	3415	2	800	10000	AB_2059244
Atg7	ATG7	ATG7	>0.7	Rabbit	Cell Signaling	8558	1	300	10000	AB_10831194
Atg4B	Atg4B	ATG4B	0.5-0.7	Rabbit	cell signaling	13507	1	200	10000	AB_2750642
Aurora A	AURKA	AURKA	>0.7	Rabbit	cell signaling	14475	2	400	10000	AB_2665504
Aurora A/B/C (phospho_T288_T232_T198)	Aurora ABC_p_T288_T232_T198	AURKA, B, C	0.5-0.7	Rabbit	cell signaling	2914	9	500	10000	AB_2061631
Axl	AXL	AXL	>0.7	Rabbit	cell signaling	8661	4	300	10000	AB_11217435
B7-H3	CD276	CD276	0.5-0.7	Rabbit	cell signaling	14058	1	50	10000	AB_2750877
B7-H4	VTCN1	VTCN1	0.5-0.7	Rabbit	cell signaling	14572	1	50	10000	AB_2750878
Baf47/SMARCB1	SMARCB1	SMARCB1	>0.7	Rabbit	cell signaling	8745	1	500	10000	AB_10950321
bak	BAK1	BAK1	0.5-0.7	Rabbit	abcam	ab32371	GR106730-6	150	10000	AB_725624
BAP1	BAP1	BAP1	0.5-0.7	mouse	santa Cruz	sc-28383	F2821	250	10000	AB_626723
bax	BAX	BAX	>0.7	Rabbit	cell signaling	2772	10	300	10000	AB_10695870
bcl2	BCL2	BCL2	>0.7	mouse	DAKO	M0887	6140	1000	10000	AB_2064429
bcl2-pser70	bcl2-phospho ser70	bcl2-p70	0.5-0.7	Rabbit	cell signaling	2827	13	250	10000	AB_659950
Bcl2A1	BCL2A1	BCL2A1	>0.7	Rabbit	abnova	PAB8528	40201	2000	10000	AB_1672563
bcl-XL	BCL2L1	BCL2L1	>0.7	Rabbit	cell signaling	2762S	7	400	10000	AB_10694844
Beclin-1	BECN1	BECN1	>0.7	Rabbit	cell signaling	3738	3	400	10000	AB_490837
bid	BID	BID	0.5-0.7	Rabbit	cell signaling	2002	3	500	10000	AB_10692485
bim	BCL2L11	BCL2L11	>0.7	Rabbit	abcam	ab32158	GR66791-12	100	10000	AB_725697
bip/GRP78	GRP78	GRP78	>0.7	mouse	BD biosciences	610978	4357837	500	10000	AB_398291
Bmi-1	BMI1	BMI1	0.5-0.7	Rabbit	cell signaling	2830	4	50	10000	AB_2065421
BRD4	BRD4	BRD4	0.5-0.7	Rabbit	epitomics	5716-1	yl101204CS	1000	10000	AB_11145462
Brg1	SMARCA4	SMARCA4	0.5-0.7	Rabbit	cell signaling	49360	3	500	10000	AB_2728743
BRM	SMARCA2	SMARCA2	>0.7	Rabbit	cell signaling	6889	2	300	10000	AB_10831818
BTK	BTK	BTK	0.5-0.7	Rabbit	Abclonal	A19002	4000000485	300	10000	AB_2862494
C23 (nucleolin	NCL	NCL	>0.7	mouse	santa Cruz	sc8031	D2908	300	10000	AB_670271
Cadherin-E	CDH1	CDH1	>0.7	Rabbit	Cell Signaling	3195	13	50	10000	AB_2291471
caspase 3	CASP3	CASP3	0.5-0.7	Rabbit	cell signaling	9662	10	2000	10000	AB_331439
caspase 3(active)	CASP3 cleaved	CASP3 cl	0.5-0.7	Rabbit	Abcam	ab32042	GR297363-2	1000	10000	AB_725947
caspase 7 cleaved Asp198	CASP7 cleaved Asp198	CASP7 cl198	0.5-0.7	Rabbit	cell signaling	9491	9	75	10000	AB_2068144
caspase 9	CASP9	CASP9	0.5-0.7	mouse	BD Sciences	551246	80518	100	10000	AB_394118
phospho-catenin-beta Ser33/37/Thr41	CTNNB1-phospho Ser33/37/Thr41	CTNNB1 p33/37/41	>0.7	Rabbit	cell signaling	9561	12	500	10000	AB_331729
phospho-catenin-beta/T41/S45	CTNNB1-phospho Thr41/Ser45	CTNNB1 p41/45	>0.7	Rabbit	cell signaling	9565	2	150	10000	AB_331731
Caveolin-1	CAV1	CAV1	>0.7	Rabbit	cell signaling	3238	3	75	10000	AB_2072166
Cbl-c	CBL	CBL	0.5-0.7	mouse	BD biosciences	610441	66598	250	10000	AB_397815
Cbx7	CBX7	CBX7	0.5-0.7	Rabbit	Abcam	ab21873	GR3210651-2	2000	10000	AB_726005
CD4	CD4	CD4	>0.7	Rabbit	Abcam	ab133616	GR303505-17	500	10000	AB_2750883
CD11a	ITGAL	ITGAL	>0.7	mouse	BD biosciences	610826	3130886	500	10000	AB_398145
CD29	ITGB1	ITGB1	>0.7	mouse	BD Sciences	610467	7008831	25	10000	AB_2128060

CD31/PECAM	PECAM1	PECAM1	>0.7	mouse	Dako	M0823	66899	50	10000	AB_2114471
CD44	CD44	CD44	0.5-0.7	mouse	Cell Signaling	3570	10	100	10000	AB_2076465
CD49b	ITGA2	ITGA2	>0.7	mouse	BD biosciences	611016	5329899	100	10000	AB_398329
CD70	CD70	CD70	>0.7	goat	R & D systems	AF2738	VIQ032104A	500	10000	AB_2229046
CD74	CD74	CD74	0.5-0.7	mouse	santa Cruz	sc-6262	? from Samaniego	150	10000	AB_627176
CD86	CD86	CD86	>0.7	Rabbit	Abcam	ab053004	YH0809040	1000	10000	AB_869050
CD134/OX40	TNFRSF4	TNFRSF4	0.5-0.7	Rabbit	Abcam	ab76000	GR3193490-1	50	10000	AB_1310050
cdc2/cdk1	CDK1	CDK1	0.5-0.7	mouse	calbiochem	cc01	D30015	75	10000	AB_564423
CDC25C	CDC25C	CDC25C	>0.7	Rabbit	cell signaling	4688	3	75	10000	AB_560956
cdk2	CDK2	CDK2	>0.7	mouse	santa Cruz	sc6248	D1607	25	10000	AB_627238
CDK1/2/3 (phospho T14)	CDK1/2/3_phosphoT14	CDK1/2/3_pT14	0.5-0.7	Rabbit	Abcam	ab32384	GR235756-25	1000	10000	AB_726756
CDK9	CDK9	CDK9	>0.7	Rabbit	cell signaling	2316	9	2000	10000	AB_2291505
CDT1	CDT1	CDT1	>0.7	Rabbit	cell signaling	8064	4	100	10000	AB_10896851
CDX2	CDX2	CDX2	0.5-0.7	Rabbit	cell signaling	12306S	1	25	10000	AB_2797879
ChK1	CHEK1	CHEK1	0.5-0.7	mouse	cell Signaling	2360	3	250	10000	AB_2080320
ChK1-pS345	CHEK1 phospho Ser345	CHEK1 pS345	0.5-0.7	Rabbit	cell Signaling	2348	15	250	10000	AB_331212
ChK2	CHEK2	CHEK2	>0.7	mouse	cell Signaling	3440	4	150	10000	AB_2229490
ChK2-pT68	CHEK2 phospho Thr68	CHEK2 pT68	0.5-0.7, hard to do in Leu	Rabbit	cell Signaling	2197	2	500	10000	AB_2080501
cIAP-1/HIAP	BIRC2	BIRC2	0.5-0.7	Rabbit	upstate	07-759	30622	800	10000	AB_492650
CK2α	CSNK2A1	CSNK2A1	0.5-0.7	Rabbit	Cell Signaling	2656	3	25	10000	AB_2236816
CLpP	CLPP	CLPP	>0.7	Rabbit	Abcam	ab124822	Y10531055	1000	10000	AB_10975619
cox-2	PTGS2	PTGS2	0.5-0.7	Rabbit	cell signaling	4842	6	100	10000	AB_2084968
CREB	CREB1	CREB1	>0.7	Rabbit	Epitomics	1496-1	C08024	2000	10000	AB_562092
CREB-p(ser133)	CREB2 phospho Ser133	CREB1 p133	0.5-0.7	Rabbit	Epitomics	1113-1	YE092401	2000	10000	AB_351503
CRM1	XPO1	XPO1	>0.7	Rabbit	ThermoFisher	703238	2072635	2000	10000	AB_2784570
CSE1L/XPO2	CSE1L	CSE1L	>0.7	Rabbit	proteintech	22219-1-AP	20876	2000	10000	AB_10950892
CSK	CSK	CSK	0.5-0.7	Rabbit	cell signaling	4980	2	150	10000	AB_2276592
CTCF	CTCF	CTCF	0.5-0.7	Rabbit	cell signaling	3418	5	1000	10000	AB_2086791
CXCR5	CXCR5	CXCR5	0.5-0.7	Rabbit	Abcam	ab133706	GR3261998-3	500	10000	AB_2938524
cyclin B1	CCNB1	CCNB1	>0.7	Rabbit	Epitomics	1495-1	yc053103	1000	10000	AB_562272
cyclin D1(M-20)	CCND1	CCND1	>0.7	Rabbit	santa Cruz	sc-718	H2007	500	10000	AB_2070436
cyclin D3	CCND3	CCND3	0.5-0.7	mouse	Cell Signaling	2936	2	500	10000	AB_2070801
DAP Kinase 1 (phospho S308)	DAPK1_pS308	DAPK1_pS308	Special_Request	Mouse	GeneTex	GTX10524	822002946	5000	10000	AB_381070
DAP5/NAT1	EIF4G2	EIF4G2	>0.7	mouse	BD Biosciences	610743	39405	500	10000	AB_398066
DDB-1	DDB-1	DDB1	>0.7	Rabbit	cell signaling	6998	1	400	10000	AB_10829458
DLST	DLST	DLST	>0.7	rabbit	cell signaling	11954	? from Hui Feng	50	10000	AB_2732907
DLX1	DLX1	DLX1	0.5-0.7	mouse	abnova	H00001745_M01	11055-2H3	4000	10000	AB_828811
DNMT1 (D63A6)	DNMT1	DNMT1	>0.7	Rabbit	cell signaling	5032	1	500	10000	AB_10548197
DPF2	DPF2	DPF2	>0.7	Rabbit	cell signaling	71642	1	500	10000	AB_2938525
DRP1 (D8H5)	DRP1	DNM1L	>0.7	Rabbit	cell signaling	5391	1	1000	10000	AB_11178938
DUSP4/MKP2	DUSP4	DUSP4	>0.7	rabbit	cell signaling	5149	1	50	10000	AB_2750867
DUSP6/MKP3	DUSP6	DUSP6	>0.7	rabbit	Abcam	ab76310	GR76113-4	2000	10000	AB_1523517
DVl3	DVL3	DVL3	>0.7	Rabbit	cell signaling	3218	2	400	10000	AB_10694060
E2F-1	E2F1	E2F1	>0.7	mouse	santa Cruz	sc-251	G1017	50	10000	AB_627476
eEF2	EEF2	EEF2	0.5-0.7	Rabbit	cell signaling	2332	7	150	10000	AB_10693546
eEF2K	EEF2K	EEF2K	>0.7	Rabbit	cell signaling	3692	3	150	10000	AB_10694413
EGFR	EGFR	EGFR	>0.7	Rabbit	cell signaling	2232	16	75	10000	AB_331707
eIF2α	EIF2S1	EIF2S1	0.5-0.7	Rabbit	cell signaling	9722	13	4000	10000	AB_2230924
phospho-eIF2-α ser51	EIF2S1-phospho Ser51	EIF2S1 p51	>0.7	Rabbit	cell signaling	9721	16	500	10000	AB_330951
eIF4E	EIF4E	EIF4E	>0.7	Rabbit	cell signaling	9742	3	800	10000	AB_823488
eIF4E-phospho ser209	EIF4E-phospho ser209	EIF4E-p209	>0.7	Rabbit	abcam	ab76256	GR210598-5	1000	10000	AB_1523534
EIF4G	EIF4G1	EIF4G1	0.5-0.7	Rabbit	cell signaling	2498	3	2000	10000	AB_2096025
ElK-1(phospho-ser383)	ELK1-phospho Ser383	ELK1 p383	0.5-0.7	Rabbit	cell signaling	9181	7	200	10000	AB_2099016
Eph Receptor A2	EPHA2	EPHA2	**>0.7**	Rabbit	cell signaling	6997	1	400	10000	AB_10827743
EphA2 (phospho Ser897)	EphA2_pS897	EPHA2	0.5-0.7	Rabbit	cell signaling	6347	1	400	10000	AB_11220420
EphA2 (phospho Tyr588)	EphA2_pY588	EPHA2	0.5-0.7	Rabbit	cell signaling	12677	1	500	10000	AB_2797989
Epithelial Membrane Antigen(EMA)	MUC1	MUC1	0.5-0.7	mouse	DAKO	M061329-2	20045580	400	10000	AB_2750907
Erg1/2/3	ERG	ERG	>0.7	Rabbit	santa Cruz	sc-353	H2807	1000	10000	AB_675518
erk2	MAPK1	MAPK1	>0.7	Rabbit	santa Cruz	Sc-154	A0605	10000	10000	AB_2141292
erk-p42/44(Thr202/Tyr204	MAPK1/MAPK3-phospho Thr202/Tyr204	MAPK1/3 p202/204	>0.7	Rabbit	cell signaling	9101	30	1000	10000	AB_331646
Erk5	MAPK7	MAPK7	**>0.7**	Rabbit	cell signaling	3552	3	200	10000	AB_2297353
Ets-1	ETS1	ETS1	>0.7	Rabbit	bethyl Lab	A303-501A	1	2000	10000	AB_10951836
EVI1	MECOM	MECOM	>0.7	Rabbit	cell signaling	2593	4	800	10000	AB_2184098
Ezh2	EZH2	EZH2	>0.7	Rabbit	cell signaling	5246	9	1000	10000	AB_10694683
Ezrin	EZR	EZR	0.5-0.7	Rabbit	Abclonal	A19048	4000000392	500	10000	AB_2862541
FAK	PTK2	PTK2	>0.7	Rabbit	Cell Signaling	3285	2	200	10000	AB_2269034
FAK pY397	PTK2 phospho tyr397	PTK2 pY397	>0.7	Rabbit	cell signaling	3283	6	50	10000	AB_2173659
Fatty Acid Synthase	FASN	FASN	>0.7	Rabbit	cell signaling	3180	2	1000	10000	AB_2100796
Fibronectin	FN1	FN1	>0.7	Rabbit	Epitomics	1574-1	C11237=1	10000	10000	AB_562115
Fli-1	Fli1	Fli1	0.5-0.7	Rabbit	Dennis Watson South Carolina	? From Watson	?from Watson	1000	10000	N/A
Fos-C	FOS	FOS	0.5-0.7	Rabbit	cell signaling	2250	2	500	10000	AB_2247211
FoxM1	FOXM1	FOXM1	>0.7	Rabbit	cell signaling	5436	4	75	10000	AB_10692483

FoxO3a	FOXO3	FOXO3	**>0.7**	Rabbit	cell signaling	12829	5	250	10000	AB_2636990
FKHRL1/FoxO3α (P-Ser 318/321)	FOXO3-phospho Ser318/321	FOXO3-p318/321	0.5-0.7	Rabbit	Cell Signaling	9465	1	10000	10000	AB_2106498
FRS2-a (phospho Y196)	FRS2-a_pY196	FRS2-a_pY196	0.5-0.7	Rabbit	Cell Signaling	3864	11	50	10000	AB_2106222
FZR1/CDH1	FZR1	FZR1	>0.7	Rabbit	LS Bio	LS-C159693	54697	300	10000	AB_2938530
G6PD	G6PD	G6PD	>0.7	Rabbit	cell Signaling	8866	3	50	10000	AB_10827744
Gab2	GAB2	GAB2	>0.7	Rabbit	cell signaling	3239	3	800	10000	AB_10698601
pGab2-Tyr452	GAB2-phospho Tyr452	GAB2-p452	0.5-0.7	Rabbit	cell signaling	3882	1	25	10000	AB_2107875
GAPDH	GAPDH	GAPDH	0.5-0.7	mouse	Ambion	AM4300	86081	5000	10000	AB_2536381
GATA-1	GATA1	GATA1	>0.7	Rabbit	cell signaling	3535s	1	200	10000	AB_2108288
Gata3	GATA3	GATA3	>0.7	mouse	BD Biosciences	558686	76189	500	10000	AB_2108590
galectin-3	LGALS3	LGALS3	>0.7	mouse	santa Cruz	sc-32790	? From Peter Ruvolo	75	10000	AB_627657
GCN5L2	KAT2A	KAT2A	>0.7	Rabbit	cell signaling	3305	4	200	10000	AB_2128281
Glutaminase	GLS	GLS	0.5-0.7	Rabbit	abcam	ab156876	GR249636-15	400	10000	AB_2721038
Glycogen Synthase	GYS1	GYS1	>0.7	Rabbit	cell signaling	3886	3	1000	10000	AB_2116392
Glycogen Synthase-pS641	GYS1 phospho Ser641	GYS1 pS641	>0.7	Rabbit	cell signaling	3891	2	400	10000	AB_2116390
GPX4	GPX4	GPX4	>0.7	Rabbit	abcam	ab125066	GR3229900-15	2000	10000	AB_10973901
Grp75 (D13H4)	Grp75	HSPA9	0.5-0.7	Rabbit	cell signaling	3593	2	1000	10000	AB_2120328
GSK3α/β	GSKA/GSKB	GSKA/B	>0.7	mouse	santa Cruz	sc-7291	L1605	500	10000	AB_2279451
GSK3α/β(p-ser21/9)	GSKA/GSKB-phospho Ser21/9	GSKA/B-p21/9	>0.7	Rabbit	cell signaling	9331	20	200	10000	AB_329830
H2AX (phospho S139)	H2AX_pS139	H2AX_pS139	0.5-0.7	Rabbit	Thermo Fisher sci	LFPA0025(PA5-77995)	UD2747769D	1000	10000	AB_2736382
H3 Histone	HIST3H3	HIST3H3	>0.7	Rabbit	active motif	39163	? from Jill Butler	50000	10000	AB_2614978
H3K4Me1	HIST3H3-Me1	HIST3H3-Me1	>0.7	Rabbit	cell signaling	5326	1	400	10000	AB_10695148
H3K4Me2	HIST3H3-Me2	HIST3H3-Me2	>0.7	Rabbit	active motif	39141	? from Jill Butler	8000	10000	AB_2614985
H3K4Me3	HIST3H3-Me3	HIST3H3-Me3	0.5-0.7	Rabbit	active motif	39159	? from Jill Butler	10000	10000	AB_2615077
H3K9Me2	HIST3H3-Me2	HIST3H3-Me2	0.5-0.7	Rabbit	Abcam	ab32521	GR36032-12	2000	10000	AB_732927
H3K27Ac	HIST3H3-Ac	HIST3H3-Ac	>0.7	Rabbit	cell signaling	8173	8	500	10000	AB_10949503
H3K27Me3	HIST3H3-Me3	HIST3H3-Me3	>0.7	Mouse	active motif	61017	17813007	3000	10000	AB_2614987
H3K36Me3	HIST3H3-Me3	HIST3H3-Me3	>0.7	Rabbit	active motif	61011	32412003	5000	10000	AB_2615073
HDAC1	HDAC1	HDAC1	0.5-0.7	Rabbit	Imgenex	IM-337	622601E-01	400	10000	AB_316912
HDAC2	HDAC2	HDAC2	>0.7	Rabbit	Santa Cruz	sc7899	?from Jan Hermen	400	10000	AB_2118563
HDAC3	HDAC3	HDAC3	0.5-0.7	Rabbit	cell signaling	2632	3	50	10000	AB_331545
HDAC6	HDAC6	HDAC6	>0.7	Rabbit	cell signaling	7558	1	10000	10000	AB_10891804
HER2/Erb2	ERBB2	ERBB2	>0.7	mouse	fisher scientific	MS325P0	325P160BH	1000	10000	AB_61443
HER2(p-Tyr1248)	ERBB2-phospho Tyr1248	ERBB2 pY1248	0.5-0.7 (see pEGFR)	Rabbit	R&D System	AF1768	UVM0215051	1000	10000	AB_416537
HES1	HES1	HES1	>0.7	Rabbit	cell signaling	11988	3	150	10000	AB_2728766
HEXIM1	HEXIM1	HEXIM1	>0.7	Rabbit	cell signaling	12604	1	1000	10000	AB_2797969
Hexokinase II	HK2	HK2	>0.7	Rabbit	cell signaling	2867	5	75	10000	AB_2232946
HIF-1α	HIF1A	HIF1A	0.5-0.7	Mouse	BD Biosciences	610959 (150ug)	54033,	25	10000	AB_398272
hnRNPK	HNRNPK	HNRNPK	0.5-0.7	Mouse	Santa Cruz	sc28380	?from Sean Post	2000	10000	AB_627734
HSF1	HSF1	HSF1	>0.7	Rabbit	cell signaling	4356	?from Jo	500	10000	AB_2120258
HSF1-p326 (Ser)	HSF1-phospho Ser326	HSF1-p326	>0.7	Rabbit	Abcam	ab76076	?from Jo	2000	10000	AB_1310328
HSP27	HSPB1	HSPB1	0.5-0.7	Mouse	Cell Signaling	2402	8	75	10000	AB_331761
HSP27 (phospho S82)	HSPB-phospho Ser82	HSPB1-pS82	>0.7	Rabbit	Cell Signaling	2401	14	200	10000	AB_331644
HSP60	HSP60	HSP60	>0.7	Rabbit	Cell Signaling	12165	3	500	10000	AB_2636980
Hsp75/TRAP1	TRAP1	TRAP1	**>0.7**	Mouse	BD Biosciences	612344	7325578	750	10000	AB_399710
HSP90	HSP90AA1/HSP90AB1	HSP90AA1/B1	0.5-0.7	Rabbit	Cell Signaling	4874	2	1000	10000	B_2121214
IDO	IDO1	IDO1	0.5-0.7	Rabbit	cell signaling	86630	5	100	10000	AB_2636818
IGFRβ	IGF1R	IGF1R	0.5-0.7	Rabbit	cell signaling	3027	5	750	10000	AB_2122378
IGF1R (phospho Y1135/Y1136)	IGF1R -phospho Y1135/Y1136)	IGF1R-pY1135/1136	>0.7	Rabbit	cell signaling	3024	15	30	10000	AB_331253
IGFBP-2	IGFBP2	IGFBP2	>0.7	Rabbit	Cell Signaling	3922	1	75	10000	AB_2123207
IKBα	NKB1A	NKB1A	>0.7	mouse	cell signaling	4814	17	500	10000	AB_390781
IL23 Receptor	IL23R	IL23R	>0.7	mouse	origene	TA807494	W002	1500	10000	AB_2628470
INPP4b	INPP4B	INPP4B	>0.7	Rabbit	Cell Signaling	4039	3	100	10000	AB_2126015
IRE1	ERN1	ERN1	0.5-0.7	Rabbit	cell signaling	3294	? From Horton	500	10000	AB_823545
IRS1	IRS1	IRS1	>0.7	Rabbit	Millipore	06-248	3352015	400	10000	AB_2127890
IRS2	IRS2	IRS2	>0.7	Rabbit	cell signaling	4502	5	200	10000	AB_2125774
JAB1	COPS5	COPS5	>0.7	mouse	santa Cruz	sc-13157	10605	400	10000	AB_627835
Jagged1	JAG1	JAG1	>0.7	Rabbit	Abcam	ab109536	GR474428-20	100	10000	AB_10862281
Jak2	Jak2	Jak2	>0.7	Rabbit	cell signaling	3230	10	4000	10000	AB_2128522
JMJD6	JMJD6	JMJD6	0.5-0.7	Rabbit	Abcam	ab50720	802854	1000	10000	AB_873907
Jun-B	JUNB	JUNB	>0.7	Rabbit	cell signaling	3755	1,2	50	10000	AB_2181353
Jun-c(p-ser73	JUN-phospho Ser73	JUN-p73	>0.7	Rabbit	cell signaling	9164	5, 7,11	100	10000	AB_330892
KAP1	TRIM28	TRIM28	**>0.7**	Rabbit	Abcam	ab10484	GR288493-42	5000	10000	AB_297223
Keap1	KEAP1	KEAP1	0.5-0.7	Rabbit	cell signaling	8047	1	75	10000	AB_10860776
c-Kit	KIT	KIT	>0.7	Rabbit	Epitomics	1522-1	YG071302CA	500	10000	AB_562072
Lamin B1	LMNB1	LMNB1	>0.7	Rabbit	Abcam	ab133741	GR181358-46	5000	10000	AB_2616597
LATS1	LATS1	LATS1	>0.7	Rabbit	cell signaling	3477	6	300	10000	AB_2133513
LATS2	LATS2	LATS2	>0.7	Rabbit	cell signaling	5888	1	100	10000	AB_10835233
LC3A/B	MAP1LC3A/B	MAP1LC3A/B	0.5-0.7	Rabbit	cell signaling	4108	3	500	10000	AB_2137703
Lck	LCK	LCK	>0.7	Rabbit	cell signaling	2752	2	1000	10000	AB_2234649
LEF1	LEF1	LEF1	>0.7	Rabbit	cell signaling	2230s	2	2000	10000	AB_823558
LKB1/STK11	STK11	STK11	0.5-0.7	Rabbit	cell signaling	3050	4	250	10000	AB_823559

LRP6-pS1490	LRP6-pS1490	LRP6-pS1490	>0.7	Rabbit	cell signaling	2568	6	200	10000	AB_2139327
LSD1	KDM1A	KDM1A	>0.7	Rabbit	cell signaling	2184	1	3000	10000	AB_2070132
lyn	LYN	LYN	>0.7	Rabbit	cell signaling	2732	5	400	10000	AB_10694080
MCL1	MCL1	MCL1	>0.7	Rabbit	cell signaling	5453	4	100	10000	AB_10694494
MDM2	MDM2	MDM2	0.5-0.7	Rabbit	Santa Cruz	sc-813	H3007	2000	10000	AB_2250633
MDM2-pS166	MDM2 phospho Ser166	MDM2 pS166	>0.7	Rabbit	cell signaling	3521	5	300	10000	AB_2143550
MEF2C	MEF2C	MEF2C	>0.7	Rabbit	cell signaling	5030	Alex kentsis 5/2014	200	10000	AB_10548759
MEK1	MAP2K1	MAP2K1	>0.7	Rabbit	Abcam	ab32576	GR267481-1	20000	10000	AB_776274
MEK(p-ser217/221)	MAP2K1/MAP2K2-phospho ser217/221	MAP2K1/2 pS217/221	0.5-0.7	Rabbit	cell signaling	9121	6	2000	10000	AB_331648
Menin	MEN1	MEN1	>0.7	Rabbit	cell signaling	6891	1	500	10000	AB_10858216
MEP50	WDR77	WDR77	>0.7	Rabbit	cell signaling	2018	1	2000	10000	AB_2215723
MERIT40-pS29	BABAM1-pS29	BABAM1-pS29	>0.7	Rabbit	cell signaling	12110	1	1000	10000	AB_2750884
mesothelin	MSLN	MSLN	>0.7	Rabbit	cell signaling	99966	1	500	10000	AB_2800323
Phospho-sp C-Met(Py1230/1234/1235	MET-phospho Py1230/1234/1235	MET p1230/1234/1235	>0.7	Rabbit	cell signaling	3129	5	100	10000	AB_561173
MLL1	KMT2A	KMT2A	0.5-0.7	Rabbit	cell signaling	14197	1	300	10000	AB_2688010
MLL2	KMT2D	KMT2D	>0.7	Rabbit	cell signaling	63735	1	200	10000	AB_2737357
MNK1	MKNK1	MKNK1	>0.7	Rabbit	cell signaling	2195	5	800	10000	AB_2235175
MSH2	MSH2	MSH2	>0.7	mouse	cell signaling	2850	1	150	10000	AB_2144797
MSH6	MSH6	MSH6	0.5-0.7	Rabbit	Novus	22030002	G2910-369A02	2000	10000	AB_2266534
MSI2	MSI2	MSI2	0.5-0.7	Rabbit	abcam	ab76148	GR3186291-4	200	10000	AB_1523981
MST1	STK4	STK4	>0.7	Rabbit	cell signaling	3682	4	200	10000	AB_2144632
mTor	MTOR	MTOR	>0.7	Rabbit	cell signaling	2983	16	500	10000	AB_2105622
mTor(p-Ser2448	MTOR-phospho Ser2448	MTOR pS2448	0.5-0.7	Rabbit	cell signaling	2971	14	100	10000	AB_330970
Myc-C	MYC	MYC	>0.7	Rabbit	cell signaling	9402	2	500	10000	AB_2151827
myosin heavy chain 11	MYH11	MYH11	>0.7	Rabbit	Novus	21370002	G2910305A02	2000	10000	AB_2147162
Myosin IIa-pS1943	MYH9 phospho Ser1943	MYH9 pS1943	>0.7	Rabbit	cell Signaling	5026	2	250	10000	AB_10576567
NDRG-pT346	NDRG1	NDRG1	>0.7	rabbit	Cell Signaling	3217	2	250	10000	AB_2150174
NDUFB4	NDUFB4	NDUFB4	>0.7	mouse	Abcam	ab110243	GR121071-4	50	10000	AB_10890994
NF-kB p65	RELA	RELA	0.5-0.7	Rabbit	cell signaling	3034	2	800	10000	AB_330561
NF-kappaB p65-pS536	RELA phospho Ser536	RELA pS536	0.5-0.7	rabbit	Cell Signaling	3033	16	300	10000	AB_331284
NF-kB p100	RELA	RELA	0.5-0.7	rabbit	Abcam	ab191594	GR240670-2	500	10000	AB_2893501
NF2	NF2	NF2	0.5-0.7	Rabbit	Santa Cruz	sc-332	H0905	800	10000	AB_2149825
nicastrin	NCSTN	NCSTN	>0.7	Rabbit	Cell Signaling	9447	1	400	10000	AB_10950501
NLN	NLN	NLN	>0.7	mouse	origene	TA504178	F001	2000	10000	AB_11128792
Notch1	NOTCH1	NOTCH1	>0.7	Rabbit	Cell Signaling	3268	2	100	10000	AB_1264224
Notch1-cleaved (Val1744)	NOTCH1 cleaved val1744	NOTCH1 cl1744	0.5-0.7	Rabbit	cell signaling	4147	7	75	10000	AB_2153348
Notch2	NOTCH2	NOTCH2	>0.7	Rabbit	cell signaling	4530	2	400	10000	AB_10860068
Notch3	NOTCH3	NOTCH3	0.5-0.7	Rabbit	Santa Cruz	sc-5593	C3108	500	10000	AB_2151246
NPM	NPM1	NPM1	>0.7	mouse	invitrogen	32-5200	141208	400	10000	AB_2155178
NRF2	NFE2L2	NFE2L2	>0.7	Rabbit	Cell Signaling	12721P	3	30	10000	AB_2715528
Numb	NUMB	NUMB	>0.7	Rabbit	Cell Signaling	2761	2	500	10000	AB_2267391
NUP98	NUP98	NUP98	>0.7	Rabbit	Cell Signaling	2598	5	800	10000	AB_2267700
OMA1	OMA1	OMA1	>0.7	Rabbit	cell signaling	95473	1	500	10000	AB_2800248
Osteopotin (OPN)	SPP1	SPP1	0.5-0.7	mouse	santa cruz	sc-21742	C0909	25	10000	AB_2194997
P16	CDKN2A	CDKN2A	>0.7	Rabbit	Abcam	ab81278	GR106921	2000	10000	AB_1640753
p21	CDKN1A	CDKN1A	>0.7	Rabbit	Santa Cruze	sc-397	H2807	1000	10000	AB_632126
p27	CDKN1B	CDKN1B	>0.7	Rabbit	Abcam	ab32034	GR198714-32	200	10000	AB_2244732
P27-phospho(s10)	CDKN1B-phospho Ser10	CDKN1B p10	>0.7	Rabbit	Epitomic	2187-1	YE081401	500	10000	AB_991784
p27 KIP1-pT198	CDKN1B phospho Thr198	CDKN1B pT198	>0.7	Rabbit	abcam	ab64949	GR42023-18	800	10000	AB_1142099
p38	MAPK14	MAPK14	>0.7	Rabbit	cell signaling	9212	11	1000	10000	AB_330713
p38-p-thr180/tyr182	MAPK14-phospho Thr180/tyr182	MAPK14 p180/182	>0.7	Rabbit	cell signaling	9211	17	200	10000	AB_331641
p53	TP53	TP53	0.5-0.7	Rabbit	cell signaling	9282	3	2000	10000	AB_331476
p70S6K	RPS6KB1	RPS6KB1	>0.7	Rabbit	Cell Signaling	9202	7	500	10000	AB_331676
p70S6K(p-thr389)	RPS6KB1-phospho thr389	RPS6KB1 ph389	>0.7	Rabbit	cell signaling	9205	5	100	10000	AB_330944
p300	EP300	EP300	>0.7	mouse	Abcam	ab3164	GR107373-1	250	10000	AB_303567
PAK1	PAK1	PAK1	>0.7	Rabbit	Cell Signaling	2602	7	800	10000	AB_330222
PAK4	PAK4	PAK4	>0.7	Rabbit	Cell Signaling	3242	2	750	10000	AB_2158622
PARP	PARP1	PARP1	0.5-0.7	Rabbit	cell signaling	9542	5	500	10000	AB_2160739
PARP(cleaved Asp214)	PARP1-cleaved Asp214	PARP1 cl214	>0.7	Rabbit	cell signaling	9541	4	100	10000	AB_331426
PAX6	PAX6	PAX6	>0.7	Rabbit	cell signaling	60433	1	50	10000	AB_2797599
Paxillin	PXN	PXN	0.5-0.7	Rabbit	abcam	ab32084	GR215998-21	800	10000	AB_779033
PCNA	PCNA	PCNA	0.5-0.7	mouse	Abcam	ab29	GR201287-12	400	10000	AB_303394
PD-1	PDCD1	PDCD1	>0.7	mouse	Cell Signaling	43248	1	50	10000	AB_2728836
Pdcd4	PDCD4	PDCD4	0.5-0.7	Rabbit	Rockland	600-401-965	24065	4000	10000	AB_828370
PDGFR-beta	PDGFRB	PDGFRB	>0.7	Rabbit	cell signaling	3169	13	30	10000	AB_2162497
PDHK1	PDHK1	PDHK1	0.5-0.7	Rabbit	cell signaling	3820	2	100	10000	AB_1904078
PDK1	PDK1	PDK1	>0.7	Rabbit	cell signaling	3062	3	200	10000	AB_2236832
PDK1-p241(Ser)	PDK1-phospho ser241	PDK1 p241	>0.7	Rabbit	cell signaling	3061	5	800	10000	AB_2161919
PD-L1	PD-L1	PD-L1	0.5-0.7	Rabbit	cell signaling	13684	7	150	10000	AB_2687655
PEA-15	PEA15	PEA15	>0.7	Rabbit	cell signaling	2780	2	500	10000	AB_2268149
PERK	EIF2AK3	EIF2AK3	>0.7	Rabbit	cell signaling	3192	10	200	10000	AB_2095847
PHLPP	PHLPP	PHLPP1	>0.7	Rabbit	Proteintech	22789-1-AP	58883	3000	10000	AB_2750897

PI3K p85	PI3K-p85	PIK3R1	0.5-0.7	Rabbit	Millipore	ABS1856	3393294	200	10000	AB_2750892
PI3 K p110-alpha	PIK3CA	PIK3CA	0.5-0.7	Rabbit	cell signaling	4255	1	200	10000	B_659888
PI3 K p110-beta	PIK3CB	PIK3CB	0.5-0.7	mouse	Santa Cruz	sc-376412	A1315	75	10000	AB_11150465
PIM2	PIM2	PIM2	>0.7	Rabbit	Cell Signaling	4730	4	75	10000	AB_2163921
PKA RI alpha	PRKAR1A	PRKAR1A	>0.7	Rabbit	Cell Signaling	5675	1	1000	10000	AB_10695452
PKCα	PRKCA	PRKCA	>0.7	mouse	Upstate	05-154	23336	10000	10000	AB_2284233
PKCα-p657(Ser)	PRKCA-phospho ser657	PRKCA-p657	0.5-0.7	Rabbit	Upstate	06-822	21320	10000	10000	AB_310258
PKCβII	PRKCB	PRKCB	0.5-0.7	mouse	santa Cruz	SC-13149	C0906	100	10000	AB_628144
PKCβII-pS660	PRKCB phospho Ser660	PRKCB pS660	>0.7	Rabbit	cell signaling	9371	3	400	10000	AB_2168219
PKM2	PKM2	PKM2	0.5-0.7	Rabbit	Cell Signaling	4053	5	500	10000	AB_1904096
PKR(PRKR)(EIF2AK2)	EIF2AK2	EIF2AK2	0.5-0.7	mouse	Abnova	H00005610-M02	7058_1D11-00Aa6	10000	10000	AB_714699
PLC gamma1 (phospho Ser1248)	PLC-gamma1_pS1248	PLCG1	>0.7	Rabbit	Cell Signaling	8713	15	500	10000	AB_10890863
PLC gamma2 (phospho Y759)	PLCG2-pY759	PLCG2-pY759	0.5-0.7	Rabbit	Cell Signaling	3874	6	50	10000	AB_2163714
PLK1	PLK1	PLK1	>0.7	mouse	Thermo scientific	MA5-171512	SG2424033	100	10000	AB_2538623
PPAR-α	PPARA	PPARA	>0.7	Rabbit	boster Bio	PA1412	0141112101253	200	10000	AB_2938527
PP2A-B55	PPP2R2D/PP2R2A/PPP2R2B/PPP2R2C	PPP2R2α/β/γ/δ	0.5-0.7	goat	santa Cruz	sc-18330	D1404	750	10000	AB_2268761
PRAS40	AKT1S1	AKT1S1	0.5-0.7	mouse	invitrogen	AHO1031	484329A	200	10000	AB_2536321
p-PRAS40 (Thr246	AKT1S1 phospho Thr246	AKT1S1 p246	>0.7	Rabbit	Cell Signaling	2997	12	100	10000	AB_2258110
PREX1	PREX1	PREX1	>0.7	rabbit	Abcam	ab102739	GR35114-16	400	10000	AB_10711640
PRMT1	PRMT1	PRMT1	>0.7	Rabbit	Cell Signaling	79780	1	2000	10000	AB_2799941
PRMT5	PRMT5	PRMT5	>0.7	Rabbit	Cell Signaling	79998	1	800	10000	AB_2799945
PRMT7	PRMT7	PRMT7	0.5-0.7	Rabbit	Cell Signaling	14762	1	2000	10000	AB_2798599
PTEN	PTEN	PTEN	>0.7	Rabbit	cell signaling	9552	2	2000	10000	AB_10694066
PTPN12	PTPN12	PTPN12	>0.7	Rabbit	Abcam	ab76942	GR255405-40	500	10000	AB_1524267
PU.1	SPI1	SPI1	0.5-0.7	Rabbit	cell signaling	2258	1	2000	10000	AB_2186909
puma	BBC3	BBC3	0.5-0.7	Rabbit	Cell Signaling	4976	7	200	10000	AB_2064551
Pyk2 (phospho Y402)	Pyk2_pY402	Pyk2_pY402	0.5-0.7	Rabbit	Cell Signaling	3291	6	500	10000	AB_2300530
Rab11	RAB11	RAB11	0.5-0.7	rabbit	Cell Signaling	3539	3	200	10000	AB_2253210
Rad50	RAD50	RAD50	>0.7	mouse	Millipore	05-525	2883130	2000	10000	AB_309782
Rad51	RAD51	RAD51	>0.7	Rabbit	cell Signaling	8875	2	100	10000	AB_2721109
Raf-A	ARAF	ARAF	>0.7	Rabbit	Cell Signaling	4432	2	200	10000	AB_330813
Raf-B	BRAF	BRAF	0.5-0.7	mouse	Santa cruz	sc5284	E1006	100	10000	AB_626760
Raf-B-pS445	BRAF phospho Ser445	BRAF pS445	>0.7	Rabbit	cell signaling	2696	3	100	10000	AB_390721
Raf-C	RAF1	RAF1	0.5-0.7	Rabbit	Millipore	04-739	2867941	300	10000	AB_1977452
Raf-C-pS338	RAF1 phospho Ser338	RAF1 pS338	>0.7	Rabbit	cell signaling	9427	10	500	10000	AB_2067317
Raptor	RPTOR	RPTOR	>0.7	Rabbit	cell signaling	2280	11	500	10000	AB_561245
Ras-N	NRAS	NRAS	>0.7	mouse	santa Cruz	sc-31	B1517	25	10000	AB_628041
Rb	RB1	RB1	>0.7	mouse	BD PharMingen	554136	52561	500	10000	AB_395259
pRb (P-Ser807/811)	RB1-phospho ser807/811	RB1 p807/811	>0.7	Rabbit	Cell Signaling	9308	9	750	10000	AB_331472
Rheb	RHEB	RHEB	0.5-0.7	mouse	R&D systems	MAB3426	WJ1021612	150	10000	AB_2178785
Rictor-pT1135	RICTOR phospho thr1135	RICTOR pT1135	>0.7	Rabbit	cell signaling	3806	5	2000	10000	AB_10557237
RIP(RIP1)	RIPK1	RIPK1	0.5-0.7	Rabbit	cell signaling	4926	2	100	10000	AB_2224503
RIP3	RIP3	RIP3	0.5-0.7	Rabbit	cell signaling	13526	3	200	10000	AB_2687467
RPA32	RPA32	RPA32	0.5-0.7	rat	cell signaling	2208	3	2000	10000	AB_2238543
RPA32 (Phospho S4/S8)	RPA32-phospho Ser4/8	RPA32-pS4/8	0.5-0.7	Rabbit	Bethyl laboratories	A300-245A	5	2000	10000	AB_210547
RSK	RPSKA1/2/3	RPSKA1/2/3	0.5-0.7	Rabbit	cell signaling	9347	5	400	10000	AB_330803
p90RSK (phospho T573)	p90RSK -phospho Thr573	p90RSK -pT573	0.5-0.7	Rabbit	cell Signaling	9346	4	50	10000	AB_330795
S100A4	S100A4	S100A4	**>0.7**	Rabbit	cell signaling	13018	3	2000	10000	AB_2750896
S6 Ribosomal protein	RPS6	RPS6	>0.7	Rabbit	cell signaling	2217	1	2000	10000	AB_331355
S6 Ribosomal protein(phospho-ser235/236)	RPS6-phospho ser235/236	RPS6 p235/236	>0.7	Rabbit	cell signaling	2211	11	2000	10000	AB_331679
S6 Ribosomal protein(phospho-ser240/244)	RPS6-phospho ser240/244	RPS6 p240/244	>0.7	Rabbit	cell signaling	2215	4	1000	10000	AB_331682
SCD	SCD	SCD	>0.7	mouse	santa Cruz	sc-58420	F1015	100	10000	AB_785599
SDHA	SDHA	SDHA	>0.7	Rabbit	cell signaling	11998	2	250	10000	AB_2750900
Set1A	SETD1A	SETD1A	>0.7	Rabbit	cell signaling	61702	1	400	10000	AB_2799614
Set1B	SETD1B	SETD1B	0.5-0.7	Rabbit	cell signaling	44922	1	400	10000	AB_2799275
SF2/SRSF1	SRSF1	SRSF1	>0.7	mouse	invitrogen	32-4500	RF234849	300	10000	AB_86196
SF3B1	SF3B1	SF3B1	0.5-0.7	Rabbit	cell signaling	14434	1	5000	10000	AB_2798479
SF3B1-pThr313	SF3B1 phospho Thr313	SF3B1 pT313	0.5-0.7	Rabbit	cell signaling	25009	1	500	10000	AB_2798893
SFRP1	SFRP1	SFRP1	>0.7	Rabbit	cell signaling	4690	1	100	10000	AB_2187066
SGK1	SGK1	SGK1	**>0.7**	Rabbit	cell signaling	12103	2	200	10000	AB_2687476
SGK3	SGK3	SGK3	**>0.7**	Rabbit	cell signaling	8156	1	100	10000	AB_10949507
Shc-pY317	SHC1 phospho tyr317	SHC1 pY317	>0.7	Rabbit	cell signaling	2431	6	200	10000	AB_2188169
SHP1	PTPN6	PTPN6	>0.7	Goat	abcam	ab260268	CR166718-7	200	10000	AB_946332
SHP1-phosphor Tyr564	PTPN6-pY564	PTPN6-pY564	0.5-0.7	Rabbit	cell signaling	8849	3	100	10000	AB_11141050
SHP-2	PTPN11	PTPN11	0.5-0.7	Rabbit	abcam	ab32083	GR75257-13	200	10000	AB_777915
SHP-2 (phospho Y542)	PTPN11-phospho Y542	PTPN11-p Y542	0.5-0.7	Rabbit	cell signaling	3751	4	75	10000	AB_330825
SIRT1	SIRT1	SIRT1	0.5-0.7	Rabbit	abcam	ab32441	379020	800	10000	AB_777937
SIRT6	SIRT6	SIRT6	>0.7	Rabbit	cell signaling	2590	1	500	10000	AB_2188926
smad1	SMAD1	SMAD1	>0.7	Rabbit	abcam	ab33902	GR308212-2	100	10000	AB_777975
Smad2	SMAD2	SMAD2	>0.7	Rabbit	cell signaling	5339	2	5000	10000	AB_10626777
phospho Smad2(ser245/250/255)	SMAD2-phospho ser245/250/255	SMAD2-p245/250/255	>0.7	Rabbit	cell signaling	3104	2	500	10000	AB_390732
phospho Smad2(ser465/467)	SMAD2-phospho ser465/467	SMAD2-p465/467	0.5-0.7	Rabbit	cell signaling	3108	8	500	10000	AB_490941

phospho Smad5(Ser463/465)	SMAD5 phospho Ser463/465	SMAD5 p463/465	>0.7	Rabbit	Epitomics	2224-1	YE092901R	500	10000	AB_1267337
Snail	SNAI1	SNAI1	>0.7	Rabbit	cell signaling	3879	14	400	10000	AB_2255011
SOD1	SOD1	SOD1	>0.7	mouse	cell signaling	4266	2	2000	10000	AB_2193898
SOD2	SOD2	SOD2	>0.7	Rabbit	cell signaling	13141	2	20000	10000	AB_2636921
Sox2	SOX2	SOX2	>0.7	Rabbit	cell signaling	2748	2	75	10000	AB_823640
SOX17	Sox17	SOX17	>0.7	Rabbit	Abcam	ab224637	GR3256628-3	500	10000	AB_2801385
SPARC	SPARC	SPARC	0.5-0.7	Rabbit	cell signaling	8725	1	500	10000	AB_10860770
Src	SRC	SRC	>0.7	mouse	Upstate	05-184	25373	1000	10000	AB_2302631
Src(phospho-tyr527)	SRC-phospho tyr527	SRC p527	>0.7	Rabbit	cell signaling	2105	5	400	10000	AB_331034
SSBP2	SSBP2	SSBP2	0.5-0.7	Rabbit	abcam	ab177944	GR162880-1	500	10000	AB_2938528
stat1	STAT1	STAT1	>0.7	Rabbit	cell signaling	9172	9	150	10000	AB_2198300
Stat1 (phospho Tyr701)	Stat1_pY701	STAT1	>0.7	Rabbit	cell signaling	9167	25	250	10000	AB_561284
stat3	STAT3	STAT3	0.5-0.7	Rabbit	cell signaling	4904	7	500	10000	AB_331269
stat3 p705(Tyr)	STAT3-phospho tyr705	STAT3 p705	>0.7	Rabbit	cell signaling	9131	5	400	10000	AB_331586
stat3-p727(Ser)	STAT3-phospho ser727	STAT3 p727	0.5-0.7	Rabbit	cell signaling	9134	5	200	10000	AB_331589
stat5a	STAT5A	STAT5A	>0.7	Rabbit	Epitomics/abcam	ab32043	GR155426-12	4000	10000	AB_778107
Stathmin	STMN1	STMN1	>0.7	Rabbit	Epitomics	1972-1	D120601	10000	10000	AB_991829
survivin	BIRC5	BIRC5	>0.7	Rabbit	abcam	ab76424	GR260077-2	1000	10000	AB_1524459
SUZ12	SUZ12	SUZ12	>0.7	Rabbit	cell signaling	3737	6	200	10000	AB_2196850
Syk	SYK	SYK	>0.7	mouse	Santa cruz	sc-1240	K2014	2000	10000	AB_628308
Tapasin	TAPBP	TAPBP	0.5-0.7	Rabbit	Enzo life science	ADI-CSA-630-D	? from Atrash, Gheath	1000	10000	AB_2039612
TG2	TGM2	TGM2	>0.7	mouse	Abcam	ab2386	GR298415-2	1000	10000	AB_2287299
TIGAR	C12ORF5	C12ORF5	>0.7	Rabbit	abcam	ab137573	GR111880-6	800	10000	AB_2721901
TNK1	TNK1	TNK1	>0.7	Rabbit	abgent	ap7722a	SH070918H	100	10000	AB_2287569
Transferrin Receptor	TFRC	TFRC	>0.7	Rabbit	novus	22500002	266A1	10000	10000	AB_10004660
TRXR1	TXNRD1	TXNRD1	>0.7	Rabbit	Cell Signaling	15140	1	500	10000	AB_2798725
TSC1/Harmartin	TSC1	TSC1	0.5-0.7	Rabbit	cell signaling	4906	2	300	10000	AB_2209790
TSC2/tuberin	TSC2	TSC2	>0.7	Rabbit	Epitomics	1613-1	YC120610	500	10000	AB_562354
TSC2-pT1462	TSC2 phospho Thr1462	TSC2 pT1462	>0.7	Rabbit	cell signaling	3617	4	200	10000	AB_490956
tubulin	TUBA1A	TUBA1A	0.5-0.7	mouse	sigma	T6074	046M4763V	5000	10000	AB_477582
Tyro3	TYRO3	TYRO3	>0.7	Rabbit	Cell Signaling	5585	1	1000	10000	AB_10706782
Ubiquityl Histone H2B	H2BFM	H2BFM	0.5-0.7	mouse	millipore	05-1312	3030533	25	10000	AB_1587119
UGT1A	UGT1A1	UGT1A1	>0.7	mouse	santa Cruz	Sc-271268	I2716	200	10000	AB_10610640
ULK1-pS757	ULK1-pS757	ULK1-pS757	0.5-0.7	Rabbit	cell signaling	6888	3	100	10000	AB_10829226
VASP	VASP	VASP	>0.7	Rabbit	Cell Signaling	3112	2	150	10000	AB_2213542
Vav1	VAV1	VAV1	0.5-0.7	Rabbit	Cell Signaling	2502	2	500	10000	AB_2213556
VCP (p97)	VCP	VCP	>0.7	mouse	Abcam	ab11433	GR29842	400000	10000	AB_298039
VDAC1/porin	VDAC1	VDAC1	>0.7	mouse	abcam	ab14734	GR3296736-17	500	10000	AB_298039
VEGFR2	KDR	KDR	>0.7	Rabbit	Cell Signaling	2479	Lot 4,6(D), 7	500	10000	AB_2212507
VHL	VHL	VHL	0.5-0.7	Rabbit	Novus	NB100-485	091504	250	10000	AB_10001296
Vimentin	VIM	VIM	0.5-0.7	mouse	Dako	M0725	20030820	400	10000	AB_10013485
WDR5	WDR5	WDR5	>0.7	Rabbit	cell signaling	13105	1	1000	10000	AB_2620133
WDR82	WDR82	WDR82	0.5-0.7	Rabbit	cell signaling	99715	1	100	10000	AB_2800319
WIPI2	WIPI2	WIPI2	0.5-0.7	rabbit	cell signaling	8567		1000	10000	AB_11178945
Wee1	Wee1	Wee1	0.5-0.7	Rabbit	cell signaling	4936	3	300	10000	AB_2288509
Wee1 (phospho S642)	Wee1_pS642	Wee1_pS642	0.5-0.7	Rabbit	cell signaling	4910	3	50	10000	AB_2215870
WTAP	WTAP	WTAP	>0.7	Rabbit	UTSA(Sanjay Bansal)	?from Sanjay Bansal	?from Sanjay Bansal	5000	10000	N/A
XPA	XPA	XPA	0.5-0.7	mouse	santa cruz	sc-56813	H0509	150	10000	AB_794177
XPF	XPF	XPF	0.5-0.7	Rabbit	abcam	ab73720	GR123824-4	150	10000	AB_1524576
XRCC1	XRCC1	XRCC1	0.5-0.7	Rabbit	cell signaling	2735	2	200	10000	AB_2218471
pYAP-ser127	YAP1-phospho Ser127	YAP1 p127	0.5-0.7 not exp. in leukemia cells(WB)	Rabbit	cell signaling	4911	1	400	10000	AB_2218913
ZAP-70	ZAP-70	ZAP70	0.5-0.7_core; >0.7_YH	Rabbit	cell signaling	2705	10	400	10000	AB_2273231

Supplementary Table S2. P-values of comparisons between quantiles for each 411 proteins according to the quantile group

Protein Name	Median Split PS1	Tertiles PS1	Quartiles PS1	Quintiles PS1	Sextiles PS1	Median Split PS2	Tertiles PS2	Quartiles PS2	Quintiles PS2	Sextiles PS2	Median Split PS3	Tertiles PS3	Quartiles PS3	Quintiles PS3	Sextiles PS3
ABL1	0,460	0,360	0,720	0,170	0,400	0,054	0,068	0,250	0,460	0,022	0,015	0,006	0,038	0,013	0,026
ABL1.pY412	0,240	0,190	0,040	0,330	0,450	0,100	0,110	0,230	0,043	0,170	0,840	0,490	0,037	0,200	0,290
ACACA	0,150	0,110	0,460	0,350	0,360	0,170	0,330	0,340	0,660	0,630	0,110	0,280	0,077	0,170	0,088
ACACA.pS79	0,830	0,950	0,850	0,930	0,990	0,800	0,640	0,490	0,410	0,550	0,140	0,520	0,310	0,047	0,520
ADAR	0,590	0,890	0,920	0,930	0,640	0,009	0,052	0,067	0,180	0,180	0,770	0,630	0,610	0,380	0,390
ADM	0,540	0,710	0,930	0,810	0,810	0,003	0,004	0,006	0,002	0,019	0,007	0,001	0,000	0,000	0,000
AIFM1	0,260	0,160	0,520	0,530	0,550	0,062	0,014	0,003	0,001	0,006	0,450	0,040	0,240	0,038	0,064
AKR1C3	0,011	0,061	0,073	0,041	0,130	0,580	0,730	0,380	0,650	0,780	0,820	0,250	0,290	0,065	0,210
AKT1	0,420	0,870	0,400	0,830	0,960	0,071	0,610	0,010	0,870	0,070	0,006	0,008	0,007	0,030	0,036
AKT1_2_3	0,880	0,910	0,900	0,560	0,980	0,420	0,110	0,360	0,350	0,470	0,005	0,001	0,004	0,011	0,017
AKT1_2_3.pS473	0,600	0,980	0,260	0,630	0,920	0,620	0,970	0,490	0,490	0,850	0,190	0,420	0,620	0,580	0,850
AKT1_2_3.pT308	0,380	0,089	0,500	0,270	0,170	0,068	0,190	0,029	0,051	0,001	0,440	0,380	0,170	0,140	0,250
AKT1S1	0,078	0,091	0,370	0,430	0,035	0,040	0,071	0,095	0,220	0,076	0,100	0,120	0,130	0,380	0,440
AKT1S1.pT246	0,670	0,870	0,340	0,390	0,710	0,920	0,830	0,810	0,970	0,890	0,160	0,660	0,360	0,370	0,870
AKT2	0,490	0,490	0,780	0,370	0,630	0,760	0,690	0,250	0,180	0,900	0,170	0,310	0,500	0,680	0,710
AKT3	0,003	0,083	0,015	0,033	0,016	0,140	0,230	0,220	0,350	0,530	0,820	0,970	0,940	0,890	0,980
AMBRA1.pS52	0,900	0,790	0,490	0,690	0,830	0,170	0,340	0,130	0,370	0,640	0,580	0,650	0,900	0,810	0,780
ANXA1	0,120	0,220	0,430	0,350	0,075	0,040	0,250	0,200	0,500	0,260	0,005	0,150	0,041	0,220	0,140
ANXA7	0,700	0,910	0,830	0,480	0,110	0,120	0,230	0,180	0,430	0,550	0,700	0,750	0,510	0,081	0,340
AR	0,100	0,011	0,042	0,023	0,021	0,510	0,660	0,820	0,760	0,520	0,800	0,610	0,520	0,500	0,790
ARAF	0,250	0,014	0,039	0,096	0,099	0,012	0,060	0,046	0,062	0,190	0,240	0,015	0,093	0,160	0,130
ARID1A	0,021	0,054	0,076	0,099	0,190	0,190	0,210	0,560	0,720	0,290	0,000	0,001	0,000	0,001	0,002
ASH2L	0,009	0,006	0,020	0,037	0,027	0,014	0,570	0,008	0,750	0,044	0,330	0,500	0,680	0,490	0,820
ASNS	0,550	0,120	0,800	0,390	0,330	0,005	0,005	0,006	0,023	0,029	0,031	0,240	0,080	0,041	0,310
ASS1	0,042	0,052	0,018	0,010	0,100	0,330	0,170	0,027	0,430	0,310	0,530	0,960	0,650	0,450	0,760
ATF3	0,120	0,023	0,002	0,000	0,001	0,140	0,280	0,320	0,410	0,550	0,830	0,230	0,360	0,380	0,470
ATG3	0,990	0,081	0,046	0,085	0,140	0,160	0,750	0,230	0,740	0,480	0,780	0,410	0,830	0,430	0,130
ATG4B	0,140	0,600	0,210	0,330	0,098	0,096	0,130	0,400	0,370	0,530	0,002	0,013	0,019	0,140	0,086
ATG7	0,950	0,870	0,820	0,620	0,580	0,018	0,011	0,033	0,030	0,040	0,660	0,370	0,690	1,000	0,470
AURKA	0,120	0,200	0,390	0,340	0,290	0,430	0,610	0,370	0,020	0,001	0,210	0,042	0,140	0,140	0,074
AURORA_A_B_C.pT288_232_198	0,410	0,052	0,120	0,310	0,240	0,360	0,120	0,140	0,270	0,077	0,600	0,520	0,160	0,002	0,050
AXL	0,450	0,200	0,130	0,300	0,560	0,270	0,230	0,210	0,001	0,110	1,000	0,820	0,470	0,480	0,840
BABAM1.pS29	0,450	0,170	0,270	0,031	0,290	0,000	0,001	0,000	0,000	0,001	0,054	0,360	0,240	0,460	0,490
BAK1	0,820	0,920	0,990	0,900	0,970	0,070	0,200	0,320	0,160	0,420	0,450	0,870	0,079	0,420	0,630
BAP1	0,990	0,900	0,960	0,880	0,990	0,210	0,450	0,100	0,360	0,680	0,099	0,180	0,370	0,030	0,610
BAX	0,600	0,810	0,830	0,940	0,830	0,720	0,980	0,780	0,630	0,780	0,100	0,130	0,240	0,470	0,350
BBC3	0,390	0,200	0,250	0,046	0,460	0,300	0,220	0,530	0,410	0,590	0,400	0,740	0,840	0,870	0,750
BCL2	0,370	0,056	0,370	0,410	0,140	0,000	0,001	0,000	0,000	0,001	0,270	0,340	0,310	0,110	0,480
BCL2.pS70	0,270	0,130	0,028	0,350	0,180	0,420	0,680	0,660	0,360	0,210	0,630	0,380	0,160	0,430	0,700
BCL2A1	0,270	0,093	0,170	0,240	0,230	0,039	0,340	0,090	0,023	0,008	0,730	0,560	0,110	0,170	0,580
BCL2L1	0,760	0,200	0,001	0,037	0,061	0,280	0,160	0,280	0,730	0,380	0,200	0,093	0,096	0,450	0,220
BCL2L11	0,500	0,180	0,098	0,076	0,470	0,680	0,540	0,450	0,520	0,550	0,870	0,890	0,950	0,750	0,970
BECN1	0,640	0,580	0,170	0,510	0,740	0,016	0,039	0,037	0,098	0,031	0,150	0,500	0,500	0,680	0,570
BID	0,110	0,077	0,320	0,190	0,350	0,800	0,560	0,710	0,870	0,340	0,800	0,210	0,470	0,410	0,470
BIRC2	0,006	0,190	0,019	0,170	0,036	0,500	0,110	0,110	0,280	0,120	0,370	0,098	0,180	0,220	0,330
BIRC5	0,076	0,120	0,310	0,052	0,280	0,340	0,058	0,190	0,290	0,170	0,087	0,110	0,160	0,081	0,370
BMI1	0,240	0,400	0,220	0,320	0,510	0,240	0,006	0,035	0,085	0,049	0,690	0,590	0,620	0,640	0,036
BRAF	0,590	0,810	0,740	0,810	0,610	0,100	0,270	0,013	0,015	0,074	0,370	0,810	0,094	0,650	0,890
BRAF.pS445	0,051	0,210	0,170	0,029	0,099	0,570	0,550	0,530	0,330	0,910	0,063	0,013	0,000	0,000	0,000
BRD4	0,024	0,140	0,099	0,025	0,370	0,360	0,570	0,280	0,140	0,071	0,061	0,390	0,087	0,360	0,650
BTK	0,094	0,160	0,300	0,120	0,190	0,350	0,220	0,490	0,130	0,061	0,610	0,440	0,330	0,410	0,630
CASP3	0,018	0,072	0,076	0,120	0,260	0,200	0,300	0,390	0,550	0,440	0,300	0,048	0,064	0,091	0,035
CASP3.cle	0,030	0,120	0,005	0,007	0,001	0,280	0,056	0,490	0,380	0,057	0,470	0,570	0,260	0,390	0,160
CASP7.cle	0,280	0,400	0,320	0,720	0,580	0,280	0,590	0,640	0,630	0,890	0,600	0,590	0,600	0,910	0,770
CASP9	0,190	0,210	0,540	0,420	0,360	0,029	0,087	0,160	0,220	0,300	0,051	0,460	0,150	0,740	0,590
CAV1	0,370	0,820	0,250	0,240	0,290	0,032	0,075	0,078	0,046	0,270	0,670	0,850	0,440	0,590	0,640
CBL	0,370	0,120	0,019	0,280	0,140	0,870	0,190	0,340	0,063	0,260	0,450	0,057	0,320	0,020	0,200
CBX7	0,180	0,044	0,520	0,130	0,130	0,027	0,007	0,000	0,006	0,058	0,240	0,450	0,700	0,640	0,770
CCNB1	0,170	0,180	0,510	0,310	0,300	0,530	0,140	0,033	0,018	0,010	0,072	0,160	0,110	0,360	0,130
CCND1	0,640	0,890	0,740	0,920	0,850	0,043	0,022	0,090	0,005	0,029	0,170	0,430	0,180	0,310	0,660
CCND3	0,520	0,470	0,430	0,550	0,830	0,870	0,770	0,630	0,960	0,750	0,290	0,130	0,130	0,370	0,210
CD276	0,630	0,440	0,700	0,320	0,390	0,610	0,110	0,005	0,051	0,310	0,960	0,260	0,780	0,150	0,430
CD4	0,048	0,030	0,120	0,140	0,093	0,001	0,023	0,002	0,015	0,008	0,290	0,760	0,460	0,510	0,940
CD44	0,200	0,220	0,120	0,012	0,005	0,380	0,130	0,770	0,100	0,400	0,920	0,520	0,990	0,720	0,880
CD70	0,070	0,280	0,330	0,490	0,320	0,990	0,880	0,730	0,650	0,880	0,049	0,990	0,025	0,038	0,005
CD74	0,035	0,100	0,099	0,099	0,078	0,670	0,470	0,530	0,850	0,460	0,006	0,001	0,005	0,013	0,007
CD86	0,550	0,850	0,720	0,680	0,920	0,630	0,940	0,880	0,740	0,240	0,100	0,230	0,420	0,150	0,620
CDC25C	0,280	0,750	0,660	0,140	0,250	0,067	0,011	0,013	0,025	0,012	0,170	0,020	0,130	0,400	0,081
CDH1	0,380	0,470	0,700	0,600	0,880	0,004	0,001	0,005	0,007	0,008	0,600	0,630	0,750	0,250	0,490
CDK1	0,180	0,120	0,064	0,048	0,330	0,800	0,580	0,720	0,310	0,750	0,004	0,120	0,014	0,083	0,024
CDK1_2_3.pT14	0,045	0,010	0,100	0,060	0,044	0,160	0,110	0,083	0,027	0,010	0,190	0,440	0,340	0,560	0,490
CDK2	0,130	0,220	0,470	0,630	0,640	0,950	0,600	0,049	0,670	0,470	0,830	0,540	0,820	0,840	0,560
CDK9	0,096	0,020	0,290	0,180	0,055	0,590	0,650	0,670	0,970	0,490	0,005	0,070	0,007	0,160	0,019
CDKN1A	0,420	0,740	0,870	0,720	0,680	0,940	0,260	0,510	0,110	0,088	0,470	0,370	0,500	0,570	0,760
CDKN1B	0,000	0,000	0,001	0,001	0,001	0,790	0,440	0,410	0,170	0,860	0,810	0,460	0,320	0,800	0,640
CDKN1B.pS10	0,002	0,000	0,002	0,001	0,003	0,900	0,490	0,650	0,860	0,820	0,300	0,190	0,390	0,590	0,680
CDKN1B.pT198	0,043	0,180	0,250	0,360	0,430	0,120	0,370	0,400	0,150	0,230	0,110	0,100	0,470	0,048	0,089
CDKN2A	0,360	0,200	0,800	0,850	0,510	0,160	0,240	0,100	0,110	0,220	0,370	0,740	0,290	0,300	0,160
CDT1	0,320	0,170	0,390	0,170	0,540	0,530	0,730	0,860	0,810	0,980	0,140	0,170	0,250	0,470	0,018
CDX2	0,380	0,650	0,680	0,140	0,810	0,140	0,470	0,350	0,053	0,390	0,880	0,570	0,620	0,690	0,880
CHEK1	0,081	0,210	0,022	0,150	0,410	0,410	0,770	0,850	0,610	0,270	0,120	0,100	0,130	0,200	0,340
CHEK1.pS345	0,350	0,280	0,710	0,460	0,640	0,180	0,370	0,580	0,580	0,650	0,330	0,970	0,450	0,760	0,590
CHEK2	0,046	0,190	0,082	0,250	0,270	0,220	0,540	0,670	0,570	0,120	0,500	0,960	0,890	0,820	0,580
CHEK2.pT68	0,830	0,890	0,970	0,930	0,920	0,046	0,020	0,120	0,019	0,110	0,520	0,570	0,720	0,920	0,950
CLPP	0,340	0,700	0,590	0,520	0,770	0,430	0,200	0,510	0,750	0,360	0,730	0,760	0,780	0,730	0,890
COPS5	0,072	0,006	0,070	0,075	0,029	0,720	0,750	0,960	0,690	0,870	0,310	0,710	0,390	0,088	0,260
CREB1	0,390	0,650	0,670	0,079	0,180	0,910	0,500	0,270	0,350	0,270	0,310	0,810	0,290	0,830	0,110
CREB1.pS133	0,560	0,130	0,340	0,660	0,230	0,038	0,270	0,160	0,390	0,400	0,660	0,930	0,950	0,970	0,940
CSK	0,880	0,026	0,260	0,100	0,160	0,340	0,015	0,120	0,380	0,052	0,690	0,860	0,900	0,540	0,960
CSNK2A1	0,340	0,790	0,810	0,079	0,360	0,330	0,440	0,810	0,550	0,092	0,760	0,520	0,700	0,290	0,060
CTNNB1.pS33_37_T41	0,710	0,800	0,620	0,260	0,740	0,027	0,260	0,019	0,460	0,061	0,120	0,077	0,410	0,190	0,250
CTNNB1.pT41_S45	0,120	0,110	0,180	0,220	0,035	0,160	0,140	0,460	0,120	0,072	0,081	0,120	0,150	0,280	0,260
CXCR5	0,670	0,060	0,900	0,840	0,250	0,610	0,650	0,500	0,450	0,650	0,560	0,500	0,820	0,630	0,430
DAPK1.pS308	0,430	0,140	0,710	0,320	0,031	0,900	0,480	0,073	0,084	0,094	0,540	0,071	0,200	0,088	0,064
DDB1	0,440	0,430	0,520	0,680	0,830	0,160	0,120	0,410	0,080	0,260	0,930	0,910	0,920	0,990	0,840
DLST	0,044	0,075	0,099	0,021	0,270	0,150	0,076	0,180	0,170	0,100	0,290	0,210	0,570	0,720	0,170
DLX1	0,500	0,420	0,820	0,540	0,540	0,320	0,720	0,530	0,680	0,950	0,360	0,610	0,570	0,150	0,600
DNM1L	0,410	0,280	0,620	0,740	0,550	0,062	0,220	0,063	0,230	0,250	0,810	0,310	0,690	0,340	0,091
DNMT1	0,390	0,330	0,830	0,810	0,670	0,110	0,760	0,063	0,260	0,041	0,110	0,730	0,400	0,700	0,220
DUSP4	0,980	0,360	0,680	0,400	0,580	0,027	0,013	0,014	0,006	0,033	0,130	0,066	0,110	0,033	0,099
DUSP6	0,270	0,280	0,510	0,470	0,430	0,880	0,550	0,820	0,450	0,640	0,700	0,630	0,760	0,260	0,590
DVL3	0,170	0,290	0,480	0,500	0,260	0,490	0,420	0,091	0,210	0,750	0,570	0,600	0,330	0,910	0,620
E2F1	0,220	0,031	0,600	0,053	0,160	0,140	0,028	0,074	0,340	0,057	0,110	0,260	0,450	0,530	0,480
EEF2	0,290	0,970	0,550	0,890	0,430	0,022	0,035	0,150	0,200	0,170	0,048	0,010	0,027	0,130	0,016
EEF2K	0,095	0,290	0,390	0,460	0,540	0,860	0,380	0,540	0,420	0,580	0,086	0,001	0,000	0,006	0,005
EGFR	0,920	0,007	0,270	0,340	0,002	0,140	0,150	0,260	0,470	0,540	0,950	0,320	0,960	0,640	0,520
EIF2AK2	0,002	0,012	0,008	0,027	0,049	0,530	0,900	0,190	0,850	0,780	0,095	0,004	0,002	0,007	0,032
EIF2AK3	0,690	0,870	0,700	0,200	0,950	0,270	0,630	0,730	0,940	0,520	0,900	0,890	0,870	0,120	0,950
EIF2S1	0,750	0,620	0,150	0,890	0,920	0,900	0,880	0,600	0,620	0,410	0,760	0,450	0,540	0,290	0,820
EIF2S1.pS51	0,290	0,740	0,034	0,130	0,130	0,140	0,045	0,160	0,110	0,080	0,230	0,180	0,210	0,520	0,580
EIF4E	0,560	0,008	0,180	0,690	0,063	0,079	0,260	0,200	0,280	0,240	0,023	0,040	0,067	0,009	0,020

EIF4E.pS209	0,012	0,094	0,084	0,160	0,250	0,700	0,250	0,490	0,250	0,053	0,041	0,040	0,017	0,063	0,160
EIF4EBP1	0,990	0,900	0,990	0,500	0,490	0,470	0,770	0,760	0,970	0,780	0,940	0,910	0,760	0,800	0,790
EIF4EBP1.pS65	0,016	0,140	0,054	0,220	0,035	0,380	0,460	0,470	0,520	0,058	0,490	0,760	0,028	0,370	0,710
EIF4G1	0,740	0,910	0,750	0,390	0,590	0,001	0,003	0,005	0,018	0,019	0,770	0,340	0,039	0,005	0,085
EIF4G2	0,290	0,330	0,360	0,260	0,710	0,170	0,210	0,010	0,120	0,500	0,430	0,670	0,680	0,950	0,920
ELK1.pS383	0,430	0,330	0,420	0,500	0,160	0,006	0,017	0,044	0,068	0,029	0,570	0,330	0,570	0,490	0,630
EP300	0,019	0,012	0,011	0,043	0,029	0,580	0,950	0,820	0,870	0,830	0,081	0,070	0,120	0,063	0,077
EPHA2	0,064	0,078	0,230	0,390	0,350	0,480	0,140	0,450	0,490	0,280	0,750	0,570	0,460	0,240	0,370
EPHA2.pS897	0,360	0,820	0,470	0,850	0,890	0,940	0,310	0,770	0,910	0,067	0,180	0,260	0,510	0,490	0,790
EPHA2.pY588	0,870	0,072	0,690	0,150	0,160	0,480	0,650	0,900	0,450	0,750	0,068	0,280	0,280	0,420	0,330
ERBB2	0,770	0,047	0,029	0,015	0,120	0,011	0,048	0,030	0,050	0,140	0,130	0,380	0,420	0,480	0,290
ERBB2.pY1248	0,410	0,120	0,190	0,590	0,095	0,093	0,300	0,330	0,310	0,340	0,380	0,560	0,700	0,620	0,870
ERG	0,092	0,170	0,000	0,006	0,014	0,590	0,660	0,510	0,360	0,390	0,500	0,860	0,740	0,600	0,900
ERN1	0,920	0,930	0,980	0,710	0,700	0,850	0,750	0,990	0,890	0,960	0,120	0,320	0,140	0,330	0,450
ESR1	0,580	0,960	0,620	0,910	0,660	0,760	0,690	0,830	0,670	0,850	0,110	0,390	0,460	0,390	0,380
ETS1	0,014	0,010	0,023	0,120	0,088	0,240	0,490	0,320	0,830	0,670	0,460	0,310	0,610	0,980	0,550
EZH2	0,013	0,140	0,060	0,083	0,100	0,250	0,310	0,590	0,290	0,530	0,015	0,010	0,003	0,016	0,004
EZR	0,014	0,190	0,100	0,320	0,250	0,710	0,800	0,440	0,690	0,860	0,660	0,510	0,160	0,580	0,680
FASN	0,620	0,180	0,690	0,210	0,450	0,000	0,000	0,000	0,001	0,000	0,360	0,940	0,810	0,460	0,360
FLI1	0,770	0,800	0,480	0,790	0,700	0,280	0,540	0,670	0,850	0,590	0,460	0,540	0,340	0,038	0,110
FN1	0,086	0,300	0,390	0,057	0,230	0,061	0,110	0,290	0,150	0,350	0,370	0,280	0,004	0,590	0,140
FOS	0,210	0,210	0,350	0,610	0,490	0,063	0,008	0,011	0,004	0,016	0,490	0,250	0,780	0,370	0,470
FOXM1	0,780	0,120	0,250	0,590	0,310	0,001	0,000	0,002	0,001	0,000	0,250	0,190	0,280	0,058	0,490
FOXO3	0,130	0,320	0,500	0,460	0,650	0,004	0,012	0,006	0,013	0,082	0,280	0,360	0,480	0,310	0,082
FOXO3.pS318_321	0,270	0,130	0,290	0,310	0,100	0,560	0,890	0,750	0,780	0,740	0,032	0,230	0,120	0,340	0,470
FRS2A.pY196	0,270	0,370	0,670	0,490	0,530	0,360	0,200	0,290	0,150	0,450	0,800	0,150	0,044	0,045	0,170
FZR1	0,074	0,110	0,037	0,370	0,250	0,071	0,910	0,025	0,440	0,068	0,410	0,790	0,750	0,370	0,520
G6PD	0,370	0,490	0,320	0,530	0,630	0,002	0,002	0,012	0,010	0,016	0,670	0,650	0,680	0,120	0,006
GAB2	0,790	0,530	0,990	0,110	0,002	0,065	0,120	0,150	0,120	0,350	0,013	0,023	0,038	0,003	0,087
GAB2.pY452	0,740	0,830	0,470	0,660	0,870	0,070	0,220	0,040	0,072	0,190	0,550	0,930	0,870	0,780	0,890
GAPDH	0,670	0,230	0,380	0,740	0,680	0,065	0,240	0,046	0,330	0,510	0,700	0,440	0,480	0,910	0,920
GATA1	0,720	0,660	0,870	0,870	0,280	0,046	0,002	0,032	0,000	0,004	0,025	0,043	0,140	0,150	0,240
GATA3	0,270	0,017	0,006	0,120	0,020	0,880	0,610	0,780	0,160	0,900	0,570	0,940	0,940	0,880	0,990
GLS	0,150	0,230	0,080	0,056	0,200	0,026	0,060	0,130	0,120	0,280	0,200	0,180	0,070	0,130	0,460
GPX4	0,480	0,280	0,810	0,730	0,560	0,032	0,023	0,096	0,084	0,063	0,150	0,020	0,120	0,033	0,094
GRP75	0,640	0,500	0,740	0,370	0,520	0,062	0,300	0,140	0,130	0,019	0,570	0,940	0,560	0,460	0,600
GRP78	0,870	0,260	0,068	0,540	0,033	0,120	0,360	0,120	0,440	0,280	0,210	0,350	0,076	0,046	0,430
GSKA_B	0,250	0,670	0,700	0,760	0,700	0,000	0,006	0,002	0,029	0,004	0,870	0,400	0,960	0,940	0,790
GSKA_B.pS21_9	0,770	0,960	0,850	1,000	0,950	0,370	0,860	0,830	0,770	0,470	0,072	0,037	0,190	0,065	0,180
GYS1	0,160	0,069	0,013	0,004	0,170	0,110	0,052	0,170	0,140	0,240	0,720	0,032	0,150	0,170	0,060
GYS1.pS641	0,420	0,270	0,610	0,430	0,270	0,230	0,077	0,290	0,410	0,290	0,630	0,880	0,800	0,720	0,850
H2AX.pS139	0,240	0,600	0,600	0,490	0,570	0,240	0,073	0,390	0,160	0,130	0,790	0,800	0,160	0,840	0,790
H2BFM	0,150	0,017	0,140	0,080	0,062	0,310	0,630	0,480	0,540	0,850	0,480	0,350	0,440	0,580	0,590
H3K27Ac	0,990	0,850	1,000	0,800	0,870	0,003	0,000	0,000	0,000	0,000	0,650	0,160	0,510	0,440	0,290
H3K27Me3	0,900	0,650	0,640	0,720	0,690	0,000	0,000	0,001	0,000	0,000	0,000	0,000	0,000	0,000	0,000
H3K36Me3	0,620	0,720	0,590	0,400	0,570	0,350	0,670	0,570	0,340	0,430	0,110	0,520	0,330	0,490	0,210
H3K4Me1	0,480	0,580	0,650	0,110	0,890	0,210	0,150	0,039	0,095	0,060	0,320	0,480	0,210	0,140	0,690
H3K4Me2	0,820	0,098	0,000	0,003	0,008	0,032	0,130	0,180	0,140	0,360	0,073	0,130	0,200	0,730	0,200
H3K4Me3	0,250	1,000	0,024	0,082	0,048	0,390	0,420	0,018	0,096	0,290	0,170	0,800	0,550	0,530	0,830
H3K9Me2	0,240	0,440	0,600	0,410	0,500	0,810	0,210	0,071	0,130	0,130	0,006	0,017	0,010	0,022	0,072
HDAC1	0,430	0,200	0,340	0,760	0,390	0,940	0,670	0,680	0,960	0,300	0,130	0,220	0,260	0,250	0,550
HDAC2	0,690	0,760	0,630	0,320	0,530	0,110	0,330	0,230	0,530	0,570	0,025	0,044	0,110	0,012	0,120
HDAC3	0,030	0,057	0,097	0,490	0,032	0,400	0,330	0,330	0,025	0,160	0,390	0,530	0,280	0,690	0,790
HDAC6	0,370	0,630	0,690	0,530	0,880	0,013	0,024	0,050	0,210	0,073	0,038	0,095	0,210	0,060	0,170
HES1	0,630	0,440	0,520	0,530	0,710	0,330	0,320	0,450	0,650	0,660	0,036	0,043	0,057	0,019	0,015
HEXIM1	0,550	0,550	0,550	0,520	0,810	0,130	0,350	0,460	0,170	0,160	0,610	0,045	0,082	0,070	0,045
HIF1A	0,150	0,047	0,016	0,081	0,210	0,210	0,034	0,180	0,500	0,029	0,340	0,840	0,480	0,680	0,590
HIST3H3	0,510	0,830	0,150	0,083	0,340	0,880	0,410	0,580	0,410	0,740	0,150	0,230	0,190	0,370	0,280
HK2	0,120	0,076	0,310	0,330	0,330	0,130	0,890	0,390	0,670	0,140	0,024	0,000	0,001	0,001	0,000
HNRNPK	0,430	0,560	0,600	0,800	0,940	0,630	0,450	0,750	0,840	0,550	0,810	0,480	0,390	0,750	0,810
HSF1	0,023	0,170	0,100	0,003	0,130	0,180	0,250	0,260	0,290	0,056	0,270	0,620	0,670	0,910	0,940
HSF1.pS326	0,003	0,011	0,006	0,023	0,003	0,220	0,097	0,190	0,410	0,046	0,010	0,026	0,012	0,009	0,058
HSP60	0,130	0,130	0,350	0,220	0,230	0,013	0,350	0,034	0,180	0,180	0,400	0,026	0,010	0,028	0,003
HSP90AA1_B1	0,740	0,630	0,630	0,730	0,200	0,002	0,000	0,001	0,000	0,001	0,083	0,420	0,380	0,190	0,380
HSPB1	0,720	0,200	0,007	0,013	0,037	0,460	0,380	0,570	0,110	0,650	0,035	0,055	0,077	0,160	0,120
HSPB1.pS82	0,200	0,140	0,380	0,200	0,420	0,150	0,071	0,110	0,043	0,240	0,001	0,008	0,000	0,001	0,010
IDO1	0,270	0,460	0,610	0,210	0,750	0,550	0,640	0,410	0,640	0,560	0,450	0,220	0,830	0,800	0,320
IGF1R	0,330	0,680	0,120	0,460	0,640	0,013	0,033	0,011	0,000	0,005	0,230	0,740	0,610	0,610	0,690
IGF1R.pY1135_1136	0,081	0,340	0,051	0,110	0,018	0,079	0,031	0,140	0,280	0,180	0,250	0,330	0,680	0,470	0,710
IGFBP2	0,590	0,960	0,870	0,670	0,910	0,000	0,000	0,002	0,000	0,000	0,210	0,350	0,660	0,950	0,780
IKB	0,360	0,540	0,700	0,220	0,640	0,110	0,290	0,300	0,220	0,620	0,100	0,440	0,190	0,320	0,600
INPP4B	0,340	0,260	0,430	0,860	0,590	0,066	0,130	0,170	0,200	0,300	0,001	0,001	0,008	0,002	0,010
IRS1	0,220	0,054	0,170	0,130	0,150	0,043	0,006	0,018	0,047	0,042	0,110	0,086	0,280	0,120	0,320
IRS2	0,160	0,088	0,210	0,390	0,310	0,900	0,610	0,910	0,910	0,760	0,620	0,720	0,400	0,740	0,910
ITGA2	0,083	0,075	0,037	0,130	0,280	0,260	0,260	0,510	0,250	0,460	0,900	0,830	0,570	0,830	0,300
ITGAL	0,200	0,650	0,270	0,160	0,260	0,000	0,000	0,000	0,001	0,000	0,240	0,400	0,330	0,350	0,310
ITGB1	0,110	0,095	0,180	0,320	0,320	0,056	0,045	0,120	0,084	0,230	0,064	0,550	0,230	0,140	0,510
JAG1	0,320	0,740	0,460	0,920	0,540	0,003	0,006	0,017	0,021	0,029	0,015	0,009	0,019	0,031	0,059
JAK2	0,640	0,330	0,700	0,880	0,540	0,003	0,023	0,011	0,019	0,012	0,018	0,096	0,038	0,050	0,170
JMJD6	0,007	0,180	0,007	0,210	0,140	0,600	0,750	0,760	0,960	0,940	0,580	0,920	0,700	0,830	0,930
JUN.pS73	0,680	0,870	0,900	0,580	0,790	0,850	0,560	0,360	0,420	0,840	0,780	0,680	0,350	0,750	0,940
JUNB	0,780	0,870	0,700	0,560	0,880	0,150	0,930	0,270	0,720	0,430	0,160	0,150	0,280	0,260	0,510
KAT2A	0,310	0,190	0,510	0,340	0,430	0,250	0,011	0,200	0,190	0,095	0,760	0,350	0,870	0,960	0,500
KDM1A	0,490	0,440	0,750	0,750	0,750	0,220	0,390	0,490	0,470	0,720	0,380	0,190	0,600	0,680	0,310
KDR	0,710	0,980	0,960	0,890	0,720	0,530	0,690	0,450	0,150	0,610	0,280	0,390	0,270	0,140	0,058
KEAP1	0,460	0,990	0,580	0,720	0,110	0,640	0,520	0,900	0,200	0,360	0,046	0,690	0,086	0,028	0,029
KIT	0,040	0,002	0,130	0,006	0,006	0,230	0,410	0,460	0,037	0,810	0,042	0,260	0,130	0,110	0,250
KMT2A	0,580	0,770	0,770	0,970	0,960	0,085	0,023	0,046	0,240	0,100	0,450	0,420	0,870	0,950	0,800
KMT2D	0,072	0,023	0,074	0,093	0,075	0,850	0,700	0,910	0,980	0,770	0,700	0,180	0,350	0,470	0,350
LATS1	0,680	0,950	0,180	0,180	0,760	0,860	0,770	0,970	0,940	0,690	0,110	0,270	0,280	0,290	0,610
LATS2	0,150	0,049	0,560	0,290	0,044	0,290	0,870	0,390	0,760	0,880	0,860	0,270	0,070	0,059	0,030
LCK	0,001	0,053	0,008	0,035	0,020	0,190	0,930	0,470	0,830	0,580	0,320	0,110	0,430	0,400	0,220
LEF1	0,160	0,045	0,056	0,017	0,033	0,540	0,077	0,330	0,810	0,260	0,700	0,770	0,430	0,200	0,930
LGALS3	0,310	0,520	0,750	0,390	0,740	0,800	0,190	0,700	0,100	0,250	0,370	0,840	0,630	0,960	0,790
LMNB1	0,710	0,660	0,830	0,520	0,960	0,000	0,000	0,000	0,000	0,000	0,001	0,000	0,000	0,000	0,000
LRP6.pS1490	0,700	0,950	0,880	0,870	0,920	0,870	0,940	0,980	0,960	0,960	0,730	0,110	0,040	0,820	0,023
LYN	0,023	0,075	0,120	0,053	0,250	0,780	0,800	0,560	0,640	0,970	0,110	0,240	0,470	0,790	0,300
MAP1LC3A_B	0,600	0,400	0,460	0,800	0,700	0,079	0,110	0,340	0,100	0,360	0,280	0,010	0,080	0,008	0,025
MAP2K1	0,510	0,250	0,330	0,720	0,440	0,800	0,550	0,780	0,620	0,870	0,610	0,480	0,140	0,110	0,190
MAP2K1.pS217_221	0,250	0,370	0,590	0,220	0,140	0,240	0,085	0,250	0,510	0,170	0,730	0,860	0,630	0,640	0,980
MAPK1	0,910	0,650	0,770	0,160	0,620	0,990	0,670	0,900	0,790	0,470	0,068	0,220	0,130	0,490	0,620
MAPK1_3.pT202_Y204	0,440	0,150	0,020	0,084	0,180	0,430	0,670	0,720	0,750	0,920	0,630	0,320	0,130	0,420	0,250
MAPK14	0,016	0,028	0,066	0,037	0,100	0,800	0,390	0,950	0,910	0,710	0,990	0,730	0,860	0,520	0,420
MAPK14.pT180_Y182	0,023	0,028	0,110	0,028	0,029	0,610	0,470	0,780	0,380	0,850	0,160	0,056	0,210	0,330	0,220
MAPK7	0,130	0,220	0,200	0,430	0,520	0,280	0,290	0,510	0,650	0,520	0,300	0,580	0,680	0,500	0,610
MCL1	0,840	0,790	0,210	0,540	0,980	0,600	0,820	0,670	0,380	0,540	0,940	0,270	0,200	0,600	0,520
MDM2	0,950	0,440	0,900	0,900	0,180	0,700	0,430	0,077	0,410	0,082	0,580	0,210	0,510	0,540	0,430
MDM2.pS166	0,090	0,050	0,001	0,001	0,002	0,240	0,490	0,690	0,830	0,900	0,520	0,950	0,710	0,990	0,680
MECOM	0,970	0,730	0,950	0,790	0,850	0,083	0,003	0,010	0,025	0,020	0,009	0,180	0,027	0,011	0,000
MEF2C	0,056	0,250	0,260	0,460	0,410	0,007	0,024	0,052	0,013	0,100	0,350	0,220	0,150	0,061	0,210
MEN1	0,048	0,440	0,130	0,400	0,400	0,046	0,270	0,240	0,250	0,260	0,290	0,130	0,660	0,820	0,280
MET.pY1230_1234_1235	0,980	0,680	0,190	0,600	0,610	0,300	0,092	0,400	0,530	0,069	0,050	0,120	0,120	0,310	0,320
MKNK1	0,002	0,083	0,017	0,450	0,060	1,000	0,670	0,810	0,660	0,820	0,770	0,140	0,500	0,330	0,065

MSH2	0,440	0,530	0,580	0,270	0,370	0,020	0,300	0,017	0,340	0,240	0,380	0,290	0,230	0,790	0,340
MSH6	0,270	0,480	0,170	0,190	0,710	0,099	0,027	0,054	0,390	0,150	0,019	0,310	0,076	0,190	0,190
MSI2	0,050	0,580	0,096	0,540	0,430	0,410	0,300	0,055	0,085	0,330	0,870	0,680	0,580	0,920	0,810
MSLN	0,095	0,180	0,270	0,180	0,520	0,730	0,230	0,077	0,001	0,160	0,960	0,084	0,140	0,150	0,090
MTOR	0,600	0,630	0,034	0,470	0,150	0,006	0,013	0,012	0,026	0,013	0,010	0,000	0,000	0,000	0,001
MTOR.pS2448	0,240	0,420	0,670	0,740	0,730	0,460	0,750	0,660	0,640	0,690	0,120	0,042	0,390	0,200	0,180
MUC1	0,360	0,200	0,350	0,470	0,580	0,350	0,440	0,640	0,330	0,800	0,750	0,640	0,800	0,380	0,590
MYC	0,059	0,049	0,150	0,220	0,160	0,500	0,640	0,840	0,490	0,490	0,007	0,180	0,056	0,330	0,029
MYH11	1,000	0,110	0,590	0,140	0,220	0,082	0,530	0,110	0,170	0,039	0,970	0,850	0,670	0,890	0,300
MYH9.pS1943	0,510	0,820	0,810	0,780	0,970	0,890	0,270	0,580	0,160	0,350	0,260	0,088	0,330	0,730	0,460
NCL	0,065	0,210	0,110	0,057	0,190	0,220	0,120	0,110	0,120	0,240	0,210	0,460	0,270	0,200	0,380
NCSTN	0,320	0,110	0,330	0,530	0,330	0,690	0,940	0,920	0,980	0,890	0,150	0,680	0,380	0,460	0,570
NDRG1.pT346	0,150	0,550	0,330	0,600	0,220	0,063	0,330	0,260	0,120	0,390	0,110	0,270	0,340	0,220	0,490
NDUFB4	0,043	0,290	0,170	0,076	0,095	0,040	0,075	0,004	0,088	0,230	0,690	0,270	0,130	0,450	0,540
NF2	0,900	0,400	0,016	0,370	0,110	0,650	0,450	0,370	0,240	0,140	0,690	0,410	0,380	0,770	0,093
NFE2L2	0,180	0,090	0,360	0,220	0,069	0,026	0,052	0,015	0,015	0,170	0,280	0,640	0,570	0,890	0,800
NLN	0,240	0,039	0,260	0,390	0,067	0,072	0,005	0,016	0,083	0,041	0,250	0,890	0,620	0,015	0,008
NOL3	0,280	0,076	0,550	0,033	0,160	0,580	0,460	0,840	0,560	0,820	0,840	0,800	0,860	0,460	0,550
NOTCH1	0,510	0,840	0,530	0,480	0,880	0,000	0,000	0,000	0,000	0,000	0,029	0,001	0,001	0,023	0,007
NOTCH1.cle	0,071	0,170	0,160	0,390	0,480	0,006	0,002	0,007	0,050	0,018	0,430	0,390	0,130	0,110	0,440
NOTCH2	0,550	0,074	0,006	0,035	0,072	0,140	0,200	0,260	0,340	0,310	0,220	0,130	0,500	0,320	0,260
NOTCH3	0,013	0,230	0,094	0,230	0,120	0,008	0,020	0,046	0,037	0,019	0,030	0,260	0,057	0,510	0,300
NPM1	0,610	0,100	0,150	0,280	0,440	0,520	0,780	0,870	0,470	0,800	0,230	0,650	0,440	0,660	0,830
NRAS	0,650	0,280	0,730	0,220	0,120	0,690	0,870	0,440	0,830	0,650	0,180	0,990	0,061	0,120	0,130
NUMB	0,150	0,490	0,110	0,064	0,260	0,130	0,680	0,500	0,350	0,046	0,084	0,120	0,130	0,200	0,086
NUP98	0,170	0,053	0,160	0,035	0,180	0,740	0,380	0,620	0,800	0,410	0,520	0,960	0,830	0,620	0,790
OMA1	0,640	0,110	0,670	0,380	0,270	0,001	0,012	0,005	0,007	0,029	0,450	0,590	0,027	0,077	0,072
P90RSK.pT573	0,680	0,880	0,960	0,830	0,720	0,970	0,580	0,220	0,720	0,800	0,890	0,660	0,940	0,990	0,970
PAK1	0,052	0,390	0,280	0,400	0,470	0,340	0,310	0,730	0,660	0,690	0,100	0,005	0,048	0,055	0,014
PAK4	0,420	0,400	0,420	0,520	0,570	0,011	0,037	0,005	0,034	0,061	0,990	0,350	0,110	0,420	0,400
PARP	0,190	0,120	0,390	0,660	0,470	0,910	0,530	0,990	0,730	0,930	0,500	0,620	0,730	0,480	0,460
PARP.cle214	0,970	0,220	0,120	0,310	0,540	0,380	0,710	0,800	0,940	0,690	0,160	0,120	0,120	0,540	0,280
PAX6	0,160	0,170	0,450	0,510	0,400	0,110	0,004	0,002	0,032	0,006	0,570	0,000	0,049	0,062	0,004
PCNA	0,410	0,670	0,550	0,270	0,460	0,370	0,580	0,820	0,420	0,190	0,680	0,970	0,660	0,270	0,960
PDCD1	0,170	0,250	0,570	0,012	0,190	0,480	0,290	0,660	0,063	0,290	0,300	0,610	0,420	0,920	0,390
PDCD4	0,160	0,015	0,190	0,071	0,035	0,000	0,001	0,000	0,000	0,001	0,200	0,520	0,310	0,580	0,840
PDGFRB	0,130	0,180	0,150	0,190	0,340	0,039	0,086	0,088	0,098	0,290	0,160	0,058	0,054	0,170	0,160
PDHK1	0,350	0,320	0,610	0,000	0,270	0,470	0,330	0,850	0,590	0,660	0,720	0,460	0,140	0,020	0,170
PDK1	0,860	1,000	0,970	0,330	0,870	0,580	0,042	0,500	0,150	0,210	0,041	0,160	0,170	0,370	0,480
PDK1.pS241	0,310	0,370	0,480	0,960	0,630	0,140	0,410	0,370	0,260	0,620	0,006	0,270	0,049	0,160	0,011
PDL1	0,220	0,270	0,650	0,190	0,590	0,130	0,110	0,016	0,031	0,210	0,230	0,003	0,074	0,007	0,043
PEA15	0,720	0,036	0,320	0,014	0,087	0,620	0,820	0,850	0,340	0,320	0,000	0,003	0,002	0,001	0,015
PECAM1	0,240	0,004	0,042	0,001	0,002	0,490	0,890	0,860	0,510	0,320	0,350	0,100	0,030	0,290	0,150
PHLPP	0,440	0,340	0,670	0,210	0,470	0,630	0,130	0,860	0,870	0,130	0,490	0,410	0,820	0,250	0,640
PIK3CA	0,750	0,420	0,056	0,440	0,110	0,850	0,970	0,530	0,110	0,430	0,680	0,940	0,780	0,900	1,000
PIK3CB	0,300	0,270	0,580	0,230	0,650	0,015	0,011	0,004	0,006	0,049	0,120	0,073	0,200	0,460	0,390
PIK3R1	0,380	0,930	0,270	0,550	0,670	0,790	0,150	0,360	0,028	0,037	0,027	0,039	0,065	0,088	0,057
PIM2	0,070	0,077	0,340	0,220	0,210	0,007	0,033	0,015	0,003	0,016	0,005	0,001	0,006	0,000	0,000
PKM2	0,018	0,092	0,083	0,200	0,220	0,690	0,420	0,460	0,270	0,080	0,140	0,021	0,320	0,077	0,150
PLCG1.pS1248	0,170	0,250	0,490	0,730	0,510	0,570	0,840	0,820	0,390	0,130	0,500	0,750	0,910	0,570	0,980
PLCG2.pY759	0,480	0,830	0,870	0,720	0,680	0,880	0,910	0,980	0,800	0,800	0,360	0,390	0,720	0,840	0,510
PLK1	0,150	0,170	0,290	0,460	0,340	0,260	0,180	0,700	0,480	0,580	0,220	0,110	0,500	0,160	0,170
PPARA	0,013	0,004	0,033	0,010	0,041	0,005	0,063	0,044	0,018	0,073	0,047	0,023	0,110	0,098	0,062
PPP2R2A_B_C_D	0,770	0,410	0,160	0,950	0,720	0,100	0,021	0,140	0,420	0,057	0,260	0,180	0,360	0,270	0,560
PREX1	0,170	0,350	0,370	0,200	0,690	0,820	0,740	0,350	0,130	0,230	0,340	0,280	0,710	0,640	0,096
PRKAA1_2	0,480	0,790	0,480	0,130	0,280	0,290	0,310	0,170	0,006	0,005	0,920	0,790	0,910	1,000	0,960
PRKAA1_2.pT172	0,160	0,400	0,580	0,700	0,680	0,750	0,870	0,870	0,790	0,550	0,590	0,760	0,800	0,870	0,880
PRKAA2.pS345	0,100	0,170	0,420	0,220	0,570	0,033	0,550	0,035	0,062	0,051	0,049	0,035	0,180	0,140	0,012
PRKAR1A	0,730	0,630	0,910	0,940	0,950	0,770	0,670	0,590	0,990	0,960	0,220	0,380	0,360	0,180	0,150
PRKCA	0,630	0,520	0,930	0,370	0,260	0,410	0,610	0,450	0,830	0,930	0,120	0,062	0,170	0,083	0,071
PRKCA.pS657	0,650	0,790	0,810	0,720	0,710	0,840	0,270	0,410	0,570	0,200	0,560	0,045	0,290	0,240	0,088
PRKCB	0,067	0,270	0,200	0,270	0,530	0,360	0,600	0,510	0,092	0,450	0,042	0,210	0,160	0,250	0,170
PRKCB.pS660	0,120	0,380	0,380	0,470	0,480	0,120	0,160	0,380	0,500	0,570	0,750	0,520	0,710	0,770	0,830
PRMT1	0,088	0,015	0,200	0,260	0,120	0,870	0,720	0,760	0,930	0,660	0,006	0,026	0,029	0,041	0,008
PRMT5	0,940	0,440	0,220	0,200	0,050	0,250	0,110	0,230	0,480	0,170	0,140	0,760	0,380	0,051	0,690
PRMT7	0,170	0,580	0,200	0,430	0,480	0,510	0,140	0,570	0,510	0,390	0,550	0,650	0,830	0,950	0,720
PTEN	0,520	0,620	0,900	0,810	0,930	0,090	0,062	0,180	0,120	0,210	0,030	0,007	0,008	0,001	0,000
PTGS2	0,930	0,930	0,890	0,610	0,900	0,600	0,630	0,018	0,076	0,110	0,320	0,770	0,790	0,930	0,860
PTK2	0,510	0,700	0,890	0,700	0,620	0,055	0,042	0,140	0,240	0,210	0,021	0,002	0,008	0,026	0,008
PTK2.pY397	0,200	0,071	0,190	0,081	0,220	0,200	0,750	0,430	0,680	0,570	0,620	0,870	0,950	0,930	0,990
PTPN11	0,260	0,190	0,410	0,240	0,490	0,013	0,056	0,048	0,029	0,140	0,700	0,530	0,760	0,630	0,440
PTPN11.pY542	0,300	0,410	0,540	0,130	0,640	0,120	0,160	0,480	0,670	0,210	0,930	0,830	0,600	0,930	0,490
PTPN12	0,480	0,260	0,780	0,590	0,430	0,330	0,890	0,720	0,900	0,750	0,690	0,140	0,160	0,690	0,210
PTPN6	0,880	0,290	0,220	0,340	0,055	0,170	0,130	0,440	0,160	0,450	0,046	0,180	0,230	0,350	0,170
PTPN6.pY564	0,087	0,250	0,011	0,160	0,200	0,490	0,940	0,860	0,700	0,800	0,810	0,920	0,970	0,056	0,650
PXN	0,260	0,490	0,200	0,330	0,075	0,600	0,350	0,054	0,280	0,410	0,130	0,012	0,041	0,190	0,130
PYK2.pY402	0,650	0,360	0,670	0,072	0,490	0,400	0,640	0,580	0,850	0,820	0,290	0,990	0,320	0,088	0,310
RAB11	0,560	0,670	0,750	0,540	0,750	0,610	0,810	0,650	0,880	0,800	0,032	0,002	0,002	0,001	0,002
RAD50	0,720	0,950	0,910	0,560	0,830	0,078	0,024	0,051	0,067	0,110	0,980	0,490	0,230	0,190	0,720
RAD51	0,130	0,290	0,260	0,390	0,300	0,056	0,030	0,051	0,065	0,180	0,850	0,550	0,610	0,700	0,690
RAF1	0,390	0,046	0,400	0,340	0,160	0,680	0,790	0,880	0,900	0,710	0,500	0,580	0,910	0,390	0,780
RAF1.pS338	0,014	0,037	0,082	0,040	0,190	0,670	0,280	0,640	0,920	0,250	0,250	0,470	0,720	0,770	0,760
RB1	0,060	0,045	0,031	0,031	0,130	0,790	0,430	0,990	0,088	0,510	0,860	0,870	0,830	0,920	0,280
RB1.pS807_811	0,180	0,051	0,270	0,053	0,250	0,180	0,049	0,540	0,083	0,059	0,041	0,270	0,096	0,680	0,540
RELA_P100	0,420	0,140	0,630	0,100	0,520	0,830	0,390	0,930	0,840	0,810	0,082	0,043	0,099	0,330	0,230
RELA_P65	0,680	0,560	0,500	0,380	0,750	0,022	0,023	0,044	0,120	0,021	0,190	0,230	0,160	0,630	0,480
RELA_P65.pS536	0,640	0,280	0,290	0,940	0,490	0,620	0,520	0,460	0,810	0,780	0,500	0,540	0,470	0,770	0,750
RHEB	0,035	0,065	0,160	0,066	0,130	0,120	0,570	0,350	0,550	0,720	0,024	0,150	0,100	0,074	0,210
RICTOR.pT1135	0,310	0,024	0,300	0,078	0,050	0,400	0,320	0,041	0,240	0,460	0,100	0,290	0,047	0,075	0,035
RIP3	0,130	0,061	0,250	0,280	0,150	0,660	0,720	0,710	0,610	0,890	0,250	0,049	0,200	0,270	0,190
RIPK1	0,090	0,350	0,350	0,580	0,700	0,190	0,033	0,095	0,390	0,079	0,030	0,400	0,170	0,260	0,230
RPA32	0,160	0,370	0,530	0,770	0,610	0,001	0,029	0,004	0,050	0,021	0,005	0,044	0,044	0,110	0,140
RPA32.pS4_8	0,120	0,300	0,410	0,200	0,740	0,510	0,520	0,180	0,200	0,150	0,830	0,650	0,280	0,039	0,180
RPS6	0,330	0,150	0,200	0,370	0,470	0,240	0,310	0,430	0,490	0,510	0,270	0,071	0,390	0,360	0,026
RPS6.pS235_236	0,069	0,130	0,100	0,200	0,230	0,130	0,150	0,120	0,110	0,470	0,034	0,170	0,050	0,160	0,210
RPS6.pS240_244	0,052	0,059	0,009	0,017	0,190	0,230	0,085	0,530	0,770	0,370	0,024	0,190	0,150	0,410	0,290
RPS6KB1	0,410	0,120	0,210	0,095	0,064	0,900	0,770	0,980	0,510	0,910	0,800	0,440	0,095	0,210	0,160
RPS6KB1.pT389	0,061	0,150	0,290	0,160	0,390	0,120	0,390	0,380	0,360	0,740	0,350	0,550	0,740	0,390	0,380
RPTOR	0,410	0,550	0,840	0,610	0,680	0,410	0,760	0,270	0,800	0,810	0,850	0,980	0,960	0,730	0,990
RSKA1_2_3	0,250	0,190	0,470	0,700	0,470	0,210	0,510	0,500	0,560	0,450	0,036	0,120	0,180	0,140	0,280
S100A4	0,530	0,530	0,720	0,710	0,810	0,081	0,170	0,250	0,190	0,290	0,690	0,700	0,900	0,790	0,990
SCD	0,004	0,033	0,034	0,042	0,097	0,350	0,055	0,560	0,370	0,160	0,800	0,220	0,400	0,650	0,270
SDHA	0,640	0,720	0,750	0,930	0,520	0,200	0,930	0,390	0,790	0,270	0,038	0,050	0,170	0,058	0,026
SETD1A	0,075	0,069	0,220	0,078	0,120	0,600	0,630	0,910	0,800	0,570	0,240	0,780	0,620	0,610	0,890
SETD1B	0,490	0,780	0,820	0,990	0,670	0,210	0,630	0,550	0,640	0,710	0,160	0,250	0,300	0,410	0,670
SF3B1	0,140	0,047	0,270	0,036	0,082	0,100	0,360	0,450	0,290	0,053	0,049	0,140	0,130	0,190	0,230
SF3B1.pT313	0,310	0,820	0,640	0,690	0,730	0,610	0,400	0,520	0,920	0,590	0,190	0,090	0,240	0,340	0,420
SFRP1	0,930	0,510	0,800	0,740	0,230	0,520	0,520	0,710	0,390	0,810	0,740	0,770	0,930	0,910	0,790
SGK1	0,013	0,079	0,100	0,040	0,086	0,550	0,920	0,900	0,980	0,780	0,190	0,230	0,420	0,130	0,190
SGK3	0,470	0,510	0,360	0,078	0,320	0,018	0,006	0,017	0,029	0,060	0,073	0,280	0,170	0,120	0,094
SHC1.pY317	0,170	0,450	0,510	0,200	0,250	0,170	0,270	0,570	0,780	0,490	0,780	0,780	0,990	0,930	0,910
SIRT1	0,370	0,220	0,660	0,740	0,360	0,630	0,150	0,110	0,320	0,130	0,630	0,460	0,910	0,430	0,170
SIRT6	0,066	0,210	0,280	0,060	0,450	0,790	0,400	0,350	0,560	0,740	0,610	0,370	0,620	0,046	0,060

SMAD1	0,690	0,640	0,950	0,460	0,420	0,012	0,075	0,005	0,014	0,076	0,170	0,440	0,590	0,310	0,590
SMAD2	0,570	0,320	0,810	0,230	0,300	0,380	0,002	0,000	0,001	0,001	0,130	0,650	0,250	0,140	0,180
SMAD2.pS245_250_255	0,078	0,540	0,110	0,830	0,420	0,450	0,240	0,240	0,540	0,520	0,041	0,088	0,072	0,045	0,190
SMAD2.pS465_467	0,380	0,240	0,650	0,240	0,390	0,280	0,270	0,540	0,660	0,740	0,620	0,098	0,540	0,510	0,280
SMAD5.pS463_465	0,450	0,680	0,460	0,012	0,190	0,160	0,064	0,170	0,210	0,150	0,630	0,160	0,310	0,140	0,032
SMARCA2	0,140	0,340	0,210	0,097	0,160	0,130	0,035	0,240	0,220	0,120	0,620	0,007	0,014	0,007	0,016
SMARCB1	0,031	0,009	0,039	0,081	0,012	0,250	0,051	0,028	0,190	0,130	0,020	0,051	0,097	0,086	0,028
SNAI1	0,540	0,240	0,860	0,760	0,420	0,320	0,630	0,510	0,260	0,760	0,560	0,940	0,660	0,540	0,300
SOD1	0,980	0,830	0,560	0,630	0,560	0,074	0,011	0,130	0,130	0,100	0,820	0,850	0,054	0,040	0,010
SOD2	0,450	0,920	0,880	0,850	0,180	0,350	0,480	0,160	0,470	0,450	0,098	0,170	0,073	0,250	0,290
SOX17	0,019	0,019	0,041	0,008	0,030	0,520	0,830	0,690	0,450	0,780	0,130	0,500	0,240	0,200	0,440
SOX2	0,005	0,001	0,024	0,029	0,014	0,029	0,013	0,180	0,032	0,055	0,050	0,400	0,055	0,550	0,330
SPARC	0,002	0,088	0,013	0,180	0,017	0,035	0,018	0,030	0,100	0,095	0,017	0,063	0,073	0,200	0,170
SPI1	0,620	0,200	0,031	0,011	0,061	0,010	0,007	0,013	0,031	0,037	0,013	0,036	0,011	0,010	0,055
SPP1	0,790	0,570	0,850	0,960	0,530	0,430	0,260	0,620	0,250	0,610	0,055	0,140	0,140	0,460	0,078
SRC	0,290	0,930	0,660	0,900	0,720	0,100	0,650	0,330	0,710	0,390	0,002	0,067	0,006	0,029	0,060
SRC.pY527	0,150	0,044	0,280	0,790	0,210	0,910	0,860	0,830	0,760	0,990	0,004	0,096	0,019	0,026	0,150
SRSF1	0,620	0,440	0,200	0,350	0,790	0,900	0,930	0,270	0,430	0,560	0,750	0,610	0,960	0,730	0,900
SSBP2	0,097	0,095	0,099	0,190	0,310	0,065	0,150	0,180	0,100	0,099	0,056	0,660	0,120	0,310	0,140
STAT1	0,970	0,840	1,000	0,410	0,990	0,330	0,640	0,570	0,790	0,960	0,010	0,000	0,007	0,002	0,000
STAT1.pY701	0,140	0,083	0,120	0,280	0,220	0,076	0,086	0,360	0,180	0,069	0,053	0,095	0,087	0,220	0,310
STAT3	0,220	0,250	0,390	0,180	0,500	0,041	0,093	0,220	0,330	0,360	0,130	0,350	0,140	0,084	0,230
STAT3.pS727	0,004	0,001	0,009	0,003	0,003	0,610	0,900	0,950	0,850	0,530	0,110	0,360	0,340	0,340	0,520
STAT3.pY705	0,035	0,330	0,200	0,300	0,190	0,910	0,830	0,990	0,920	0,820	0,037	0,008	0,001	0,011	0,024
STAT5A	0,210	0,089	0,150	0,083	0,048	0,034	0,110	0,003	0,041	0,064	0,520	0,560	0,410	0,290	0,430
STK11	0,480	0,470	0,310	0,300	0,540	0,100	0,380	0,170	0,210	0,680	0,012	0,130	0,076	0,170	0,150
STK4	0,240	0,860	0,420	0,730	0,410	0,003	0,009	0,011	0,013	0,019	0,870	0,590	0,940	0,840	0,910
STMN1	0,210	0,360	0,620	0,096	0,740	0,720	0,530	0,660	0,810	0,750	0,046	0,015	0,180	0,054	0,026
SUZ12	0,500	0,730	0,810	0,580	0,970	0,076	0,007	0,200	0,061	0,040	0,100	0,280	0,180	0,540	0,430
SYK	0,180	0,081	0,097	0,057	0,220	0,540	0,610	0,880	0,630	0,760	0,380	0,340	0,450	0,790	0,190
TAPBP	0,130	0,530	0,480	0,840	0,690	0,900	0,740	0,770	0,250	0,300	0,680	0,690	0,730	0,670	0,750
TFRC	0,300	0,230	0,750	0,120	0,035	0,005	0,190	0,028	0,043	0,005	0,350	0,250	0,360	0,430	0,480
TGM2	0,510	0,790	0,700	0,300	0,740	0,051	0,012	0,062	0,085	0,064	0,010	0,030	0,012	0,078	0,078
TIGAR	0,370	0,470	0,450	0,300	0,560	0,240	0,670	0,300	0,860	0,820	0,800	0,680	0,810	0,740	0,920
TNFRSF4	0,018	0,006	0,067	0,000	0,049	0,190	0,400	0,450	0,890	0,200	0,550	0,290	0,710	0,570	0,620
TNK1	0,072	0,008	0,005	0,006	0,040	0,280	0,150	0,570	0,410	0,280	0,440	0,340	0,450	0,660	0,460
TP53	0,550	0,300	0,840	0,920	0,150	0,770	0,780	0,930	0,660	0,580	0,760	0,710	0,630	0,310	0,170
TP53BP1	0,055	0,260	0,280	0,440	0,380	0,002	0,019	0,016	0,033	0,050	0,051	0,005	0,003	0,001	0,020
TRAP1	0,470	0,180	0,280	0,800	0,290	0,097	0,031	0,150	0,078	0,120	0,920	0,570	0,250	0,190	0,320
TRIM28	0,260	0,320	0,490	0,230	0,270	0,120	0,270	0,210	0,170	0,056	0,110	0,011	0,005	0,043	0,029
TSC1	0,420	0,840	0,360	0,093	0,340	0,067	0,220	0,310	0,460	0,440	0,530	0,460	0,430	0,390	0,400
TSC2	0,042	0,012	0,005	0,026	0,053	0,500	0,790	0,860	0,470	0,380	0,570	0,680	0,930	0,430	0,360
TSC2.pT1462	0,510	0,120	0,780	0,075	0,190	0,580	0,620	0,440	0,730	0,850	0,810	0,310	0,002	0,048	0,160
TUBA1A	0,700	0,940	0,940	0,840	0,910	0,910	0,880	0,790	0,860	0,710	0,092	0,140	0,016	0,009	0,240
TXNRD1	0,710	0,580	0,430	0,790	0,750	0,200	0,450	0,270	0,670	0,410	0,073	0,094	0,190	0,300	0,120
TYRO3	0,021	0,008	0,071	0,003	0,027	0,790	0,790	0,180	0,410	0,400	0,670	0,310	0,800	0,670	0,210
UGT1A1	0,048	0,016	0,066	0,250	0,100	0,110	0,420	0,340	0,650	0,160	0,280	0,250	0,390	0,400	0,440
ULK1.pS757	0,220	0,620	0,490	0,810	0,810	0,140	0,190	0,460	0,190	0,220	0,430	0,750	0,690	0,970	0,980
VASP	0,068	0,051	0,300	0,150	0,064	0,008	0,260	0,019	0,320	0,049	0,560	0,240	0,230	0,790	0,470
VAV1	0,550	0,390	0,400	0,720	0,570	0,370	0,260	0,840	0,210	0,640	0,440	0,400	0,330	0,940	0,670
VCP	0,390	0,300	0,270	0,860	0,260	0,020	0,019	0,002	0,007	0,017	0,810	0,830	0,790	0,610	0,660
VDAC1_VDAC3	0,680	0,590	0,980	0,980	0,780	0,700	0,080	0,069	0,360	0,170	0,008	0,160	0,034	0,091	0,140
VHL	0,230	0,530	0,620	0,370	0,620	0,380	0,700	0,230	0,500	0,096	0,420	0,600	0,400	0,620	0,160
VIM	0,670	0,190	0,150	0,720	0,470	0,030	0,032	0,021	0,014	0,029	0,130	0,011	0,240	0,190	0,075
VTCN1	0,097	0,078	0,360	0,200	0,220	0,120	0,045	0,130	0,025	0,091	0,400	0,220	0,540	0,130	0,510
WDR5	0,056	0,200	0,300	0,410	0,480	0,007	0,037	0,028	0,035	0,130	0,130	0,032	0,089	0,220	0,002
WDR77	0,610	0,025	0,240	0,063	0,030	0,710	0,300	0,650	0,820	0,600	0,130	0,550	0,240	0,130	0,210
WDR82	0,530	0,630	0,420	0,450	0,099	0,001	0,025	0,005	0,007	0,015	0,450	0,230	0,770	0,018	0,440
WEE1	0,027	0,240	0,150	0,490	0,150	0,390	0,071	0,730	0,100	0,180	0,590	0,980	0,590	0,240	0,250
WEE1.pS642	0,170	0,220	0,410	0,069	0,350	0,011	0,007	0,033	0,021	0,011	0,000	0,004	0,003	0,008	0,024
WIPI2	0,025	0,055	0,150	0,140	0,120	0,820	0,061	0,310	0,057	0,079	0,540	0,610	0,680	0,950	0,940
WTAP	0,060	0,070	0,190	0,330	0,270	0,730	0,560	0,150	0,810	0,800	0,210	0,063	0,071	0,220	0,290
XPA	0,650	0,620	0,770	0,740	0,870	0,042	0,120	0,140	0,170	0,200	0,350	0,770	0,540	0,280	0,710
XPF	0,200	0,560	0,490	0,640	0,810	0,110	0,014	0,140	0,300	0,130	0,003	0,004	0,019	0,008	0,005
XRCC1	0,770	0,350	0,870	0,290	0,340	0,084	0,900	0,160	0,860	0,200	0,130	0,031	0,140	0,068	0,011
YAP1.pS127	0,330	1,000	0,240	0,910	0,490	0,140	0,420	0,260	0,430	0,720	0,220	0,079	0,082	0,130	0,210
YWHAE	0,051	0,025	0,055	0,044	0,088	0,068	0,240	0,057	0,870	0,440	0,280	0,410	0,620	0,940	0,410
ZAP70	0,290	0,017	0,095	0,170	0,089	0,600	0,430	0,170	0,330	0,490	0,630	0,920	0,360	0,680	0,500

Supplementary Table S3. P-values of selected proteins according to each protein selector set

PS1 proteins	p_value	PS2 proteins	p_value	PS3 proteins	p_value
CDKN1B	0,00017	FASN	0,0001	H3K27Me3	0,0001
LCK	0,00071	FOXM1	0,0001	LMNB1	0,0001
CDKN1B.pS10	0,0016	H3K27Ac	0,0001	STAT1	0,0001
SPARC	0,0019	IGFBP2	0,0001	HK2	0,00018
MKNK1	0,0022	LMNB1	0,0001	MTOR	0,00018
EIF2AK2	0,0024	NOTCH1	0,0001	EEF2K	0,00052
HSF1.pS326	0,0025	H3K27Me3	0,00015	INPP4B	0,00081
AKT3	0,0033	ITGAL	0,00017	PIM2	0,00082
STAT3.pS727	0,0041	HSP90AA1_B1	0,00022	ARID1A	0,00084
SCD	0,0044	CDH1	0,00062	ADM	0,00098
SOX2	0,0049	BABAM1.pS29	0,00074	RAB11	0,0019
BIRC2	0,0061	PDCD4	0,0012	PDL1	0,0026
JMJD6	0,0069	BCL2	0,0014	PEA15	0,003
ASH2L	0,0092	SMAD2	0,0015	EIF2AK2	0,0037
AKR1C3	0,011	G6PD	0,002	WEE1.pS642	0,0037
EIF4E.pS209	0,012	NOTCH1.cle	0,002	XPF	0,0038
EZH2	0,013	GATA1	0,0024	TP53BP1	0,0053
NOTCH3	0,013	EIF4G1	0,0026	PTEN	0,0069
PPARA	0,013	MECOM	0,0031	SMARCA2	0,0074
SGK1	0,013	PAX6	0,0038	HSPB1.pS82	0,0079
ETS1	0,014	ADM	0,0042	MAP1LC3A_B	0,01
EZR	0,014	ASNS	0,0049	BRAF.pS445	0,013
RAF1.pS338	0,014	NLN	0,0052	GAB2	0,023
EIF4EBP1.pS65	0,016	BMI1	0,0061	HSF1.pS326	0,026
MAPK14	0,016	IRS1	0,0061	SPI1	0,036
CASP3	0,018	JAG1	0,0061	EIF4E	0,04
PKM2	0,018	GSKA_B	0,0064	TUBA1A	0,14
TNFRSF4	0,018	SGK3	0,0064	EIF4G1	0,34
EP300	0,019	WEE1.pS642	0,0067	AURORA_A_B_C.pT288_232_198	0,52
SOX17	0,019	SPI1	0,0069		
ARID1A	0,021	CBX7	0,007		
TYRO3	0,021	SUZ12	0,0073		
HSF1	0,023	FOS	0,008		
LYN	0,023	STK4	0,0092		
MAPK14.pT180_Y182	0,023				
BRD4	0,024				
WIPI2	0,025				
WEE1	0,027				
CASP3.cle	0,03				
HDAC3	0,03				
SMARCB1	0,031				
CD74	0,035				
RHEB	0,035				
STAT3.pY705	0,035				
KIT	0,04				
ASS1	0,042				
TSC2	0,042				
CDKN1B.pT198	0,043				
NDUFB4	0,043				
DLST	0,044				
CDK1_2_3.pT14	0,045				
CHEK2	0,046				
CD4	0,048				
MEN1	0,048				
UGT1A1	0,048				

Supplementary Table S4A. Matrix of correlation coefficients for each comparison

AKR1C3	AKT3	ASH2L	ASS1	BIRC2	BRD4	CASP3	CASP3.cle	CD4	CD74	CDK1_2_3.pT14	CDKN1B	CDKN1B.pS10	CDKN1B.pT198	CHEK2	DLST	EIF4E.pS209	EIF4EBP1.pS65	EP300	ETS1	EZH2	EZR	HDAC3	HSF1	JMJD6	KIT	LCK	LYN	MAPK14	MAPK14.pT180_Y182	MEN1	MKNK1	NDUFB4
1	0,384209759	-0,236668486	0,165406011	0,247257246	-0,166593437	0,024743681	0,154227797	-0,055153681	-0,105236695	-0,125549956	0,200700277	0,319007376	-0,345726447	-0,152774095	0,377879587	-0,283234283	-0,326100634	-0,235239309	0,206445353	-0,222052321	0,119587064	0,23881382	-0,343344061	0,067874146	0,015840326	0,215331476	-0,269984467	-0,360256464	-0,409671647	-0,088565268	-0,412476458	0,269840224
0,384209759	1	-0,350671184	0,32873459	0,455893345	-0,264088378	-0,070512329	0,195960112	0,012777064	-0,053919058	-0,093474611	0,036548805	0,184536853	-0,47929091	-0,243124626	0,480773608	-0,310662435	-0,449808173	-0,395605704	0,253271933	-0,329250739	0,086515449	0,333491888	-0,451385374	0,165366175	-0,101449886	0,284317713	-0,000504524	-0,339695973	-0,265529513	-0,283409356	-0,196616045	0,399422176
-0,236668486	-0,350671184	1	-0,298552594	-0,351841762	0,49452603	0,267606559	-0,106145577	-0,311782734	0,152764825	0,209805804	-0,198030496	-0,336887553	0,578759723	0,371340946	-0,47409562	0,221249343	0,518279533	0,460669826	-0,407256143	0,47100155	-0,354427648	-0,169506892	0,571418031	-0,256284222	0,462639742	-0,569564588	-0,027065302	0,383069689	0,330831262	0,564303599	0,037663255	-0,407301425
0,165406011	0,32873459	-0,298552594	1	0,188873431	-0,413523546	-0,093299025	0,277164339	0,017790849	-0,066457499	-0,034651604	0,063844232	0,135987362	-0,451480131	-0,147340577	0,511319538	-0,263877454	-0,384911264	-0,37142818	0,190556084	-0,226731763	0,119551045	0,325687027	-0,367555043	0,166900288	-0,077005072	0,206483645	-0,098556222	-0,338097119	-0,222184494	-0,267089008	-0,117918383	0,555996812
0,247257246	0,455893345	-0,351841762	0,188873431	1	-0,089642266	-0,100207969	0,153031853	0,163085753	0,075639946	-0,165361171	0,236129231	0,329997656	-0,343870739	-0,103213688	0,315224363	-0,346718452	-0,383579814	-0,236394115	0,411334405	-0,258589023	0,198932071	0,324286144	-0,266563154	0,266254133	-0,194160066	0,327145736	-0,219475342	-0,416064263	-0,297133185	-0,121754477	-0,291516982	0,300194767
-0,166593437	-0,264088378	0,49452603	-0,413523546	-0,089642266	1	0,026784452	-0,249505753	-0,190506543	0,108545361	0,300341297	-0,194188578	-0,212026016	0,637568355	0,417745019	-0,557306735	0,264625254	0,457766002	0,604797647	-0,310352194	0,474255146	-0,251767853	-0,214098075	0,596834648	-0,311940315	0,224304983	-0,423508149	-0,08451198	0,186868012	0,140883867	0,576177474	-0,031522953	-0,430398115
0,024743681	-0,070512329	0,267606559	-0,093299025	-0,100207969	0,026784452	1	0,272212169	-0,341097836	0,030640929	-0,007509563	-0,109154898	-0,153644624	0,23403298	0,115181445	-0,14958786	0,26203912	0,201617868	0,078767051	-0,276576649	0,245697628	-0,215612281	-0,050693927	0,109364988	-0,036817404	0,210227574	-0,327674179	0,08464108	0,226766711	0,162073775	0,098315192	0,105840095	-0,204262763
0,154227797	0,195960112	-0,106145577	0,277164339	0,153031853	-0,249505753	0,272212169	1	-0,135186699	0,044741053	-0,11961169	-0,042525617	-0,007253738	-0,268514554	-0,026263451	0,474023429	-0,221404881	-0,322390153	-0,203109355	0,022092681	-0,110806797	-0,050053166	0,372306156	-0,272410683	0,132587661	-0,108552355	0,014202463	-0,180226381	-0,249829329	-0,138961728	-0,181917986	-0,116341237	0,295276774
-0,055153681	0,012777064	-0,311782734	0,017790849	0,163085753	-0,190506543	-0,341097836	-0,135186699	1	0,132581186	-0,184019277	0,534421632	0,528542368	-0,189731937	-0,325633984	0,011306552	-0,110471055	-0,159036604	-0,17022249	0,650599684	-0,327023698	0,454679299	-0,109840436	-0,198920907	0,206846717	-0,412200227	0,561636603	-0,035309152	-0,177149895	-0,05824374	-0,115922556	-0,046807877	0,015160945
-0,105236695	-0,053919058	0,152764825	-0,066457499	0,075639946	0,108545361	0,030640929	0,044741053	0,132581186	1	-0,185207177	0,143055022	0,080027614	0,122383828	0,024871277	-0,039263481	-0,074664252	0,043698748	0,046083754	0,153252557	0,098319459	-0,047396538	-0,005175953	0,153936618	0,139747853	-0,003086909	0,021227944	-0,11623221	0,015812205	0,010050347	0,168453871	-0,078240662	-0,027933836
-0,125549956	-0,093474611	0,209805804	-0,034651604	-0,165361171	0,300341297	-0,007509563	-0,11961169	-0,184019277	-0,185207177	1	-0,310005186	-0,285046406	0,243351422	0,128269737	-0,224186305	0,310582987	0,33943642	0,178430402	-0,366000921	0,386124289	-0,160524685	-0,193260456	0,129292233	-0,220046204	0,099337479	-0,423883736	-0,124322629	0,100549556	0,204054704	0,118525582	0,176844412	-0,151593319
0,200700277	0,036548805	-0,198030496	0,063844232	0,236129231	-0,194188578	-0,109154898	-0,042525617	0,534421632	0,143055022	-0,310005186	1	0,801296154	-0,228351556	-0,361170054	0,111189533	-0,145880606	-0,19684192	-0,1636016	0,567782985	-0,3356885	0,414648492	0,036124328	-0,125094498	0,21120685	-0,337595629	0,447625067	-0,23194103	-0,213303302	-0,183400817	-0,109479253	-0,262409126	0,075914849
0,319007376	0,184536853	-0,336887553	0,135987362	0,329997656	-0,212026016	-0,153644624	-0,007253738	0,528542368	0,080027614	-0,285046406	0,801296154	1	-0,334077093	-0,33455542	0,239146828	-0,252641631	-0,328671853	-0,194798639	0,62814799	-0,368817607	0,409758766	0,153203092	-0,247862956	0,227524881	-0,35410441	0,53966574	-0,22602776	-0,309038608	-0,286869341	-0,12721402	-0,300602431	0,192871436
-0,345726447	-0,47929091	0,578759723	-0,451480131	-0,343870739	0,637568355	0,23403298	-0,268514554	-0,189731937	0,122383828	0,243351422	-0,228351556	-0,334077093	1	0,419472734	-0,719953797	0,326732783	0,689664273	0,531213526	-0,392474577	0,506163947	-0,178488832	-0,4201645	0,717605095	-0,284210845	0,223985758	-0,515721741	0,049328872	0,554385646	0,393488091	0,59441176	0,125514756	-0,630710856
-0,152774095	-0,243124626	0,371340946	-0,147340577	-0,103213688	0,417745019	0,115181445	-0,026263451	-0,325633984	0,024871277	0,128269737	-0,361170054	-0,33455542	0,419472734	1	-0,16278396	0,065924694	0,197981564	0,34771972	-0,358673168	0,428460423	-0,414593287	0,003631303	0,537631312	-0,192263876	0,390759041	-0,417334552	-0,058096042	0,141625387	0,056584818	0,424564566	0,13260111	-0,11732823
0,377879587	0,480773608	-0,47409562	0,511319538	0,315224363	-0,557306735	-0,14958786	0,474023429	0,011306552	-0,039263481	-0,224186305	0,111189533	0,239146828	-0,719953797	-0,16278396	1	-0,438826946	-0,665442162	-0,50185752	0,247200079	-0,317210479	0,091670232	0,590808082	-0,512421392	0,31175328	-0,142351773	0,394711998	-0,152948316	-0,51931887	-0,442070291	-0,478178875	-0,19564718	0,677443262
-0,283234283	-0,310662435	0,221249343	-0,263877454	-0,346718452	0,264625254	0,26203912	-0,221404881	-0,110471055	-0,074664252	0,310582987	-0,145880606	-0,252641631	0,326732783	0,065924694	-0,438826946	1	0,518967554	0,226531478	-0,37590166	0,370705594	-0,106593005	-0,446796843	0,157351065	-0,182587041	0,112626885	-0,373938876	0,237432613	0,32105836	0,484320927	0,094425696	0,503980594	-0,37802943
-0,326100634	-0,449808173	0,518279533	-0,384911264	-0,383579814	0,457766002	0,201617868	-0,322390153	-0,159036604	0,043698748	0,33943642	-0,19684192	-0,328671853	0,689664273	0,197981564	-0,665442162	0,518967554	1	0,450387721	-0,375895762	0,429934538	-0,13698136	-0,518221506	0,484063576	-0,282018451	0,170735895	-0,501890692	0,0897978	0,549153114	0,554433157	0,40952262	0,229015057	-0,566371782
-0,235239309	-0,395605704	0,460669826	-0,37142818	-0,236394115	0,604797647	0,078767051	-0,203109355	-0,17022249	0,046083754	0,178430402	-0,1636016	-0,194798639	0,531213526	0,34771972	-0,50185752	0,226531478	0,450387721	1	-0,299472533	0,33298197	-0,129038025	-0,241225951	0,540771375	-0,270921633	0,148911223	-0,348984846	0,052099102	0,309248164	0,224165169	0,471276304	0,152536227	-0,417071314
0,206445353	0,253271933	-0,407256143	0,190556084	0,411334405	-0,310352194	-0,276576649	0,022092681	0,650599684	0,153252557	-0,366000921	0,567782985	0,62814799	-0,392474577	-0,358673168	0,247200079	-0,37590166	-0,375895762	-0,299472533	1	-0,528683997	0,535882247	0,118268735	-0,330465766	0,325786062	-0,35528132	0,718309674	-0,179978797	-0,353826907	-0,232682832	-0,134973835	-0,316171419	0,230783266
-0,222052321	-0,329250739	0,47100155	-0,226731763	-0,258589023	0,474255146	0,245697628	-0,110806797	-0,327023698	0,098319459	0,386124289	-0,3356885	-0,368817607	0,506163947	0,428460423	-0,317210479	0,370705594	0,429934538	0,33298197	-0,528683997	1	-0,289158106	-0,130703091	0,443627739	-0,253086786	0,341878771	-0,584024436	-0,08780985	0,259254085	0,145436378	0,341480404	0,221212581	-0,25535368
0,119587064	0,086515449	-0,354427648	0,119551045	0,198932071	-0,251767853	-0,215612281	-0,050053166	0,454679299	-0,047396538	-0,160524685	0,414648492	0,409758766	-0,178488832	-0,414593287	0,091670232	-0,106593005	-0,13698136	-0,129038025	0,535882247	-0,289158106	1	-0,04795887	-0,286768118	0,165402081	-0,377258962	0,428530047	-0,05647749	-0,082326464	-0,120548539	-0,122039969	-0,083808045	0,061491632
0,23881382	0,333491888	-0,169506892	0,325687027	0,324286144	-0,214098075	-0,050693927	0,372306156	-0,109840436	-0,005175953	-0,193260456	0,036124328	0,153203092	-0,4201645	0,003631303	0,590808082	-0,446796843	-0,518221506	-0,241225951	0,118268735	-0,130703091	-0,04795887	1	-0,229812475	0,101931394	0,019377555	0,189841602	-0,216341618	-0,431485331	-0,431705678	-0,168993556	-0,276822856	0,53836766
-0,343344061	-0,451385374	0,571418031	-0,367555043	-0,266563154	0,596834648	0,109364988	-0,272410683	-0,198920907	0,153936618	0,129292233	-0,125094498	-0,247862956	0,717605095	0,537631312	-0,512421392	0,157351065	0,484063576	0,540771375	-0,330465766	0,443627739	-0,286768118	-0,229812475	1	-0,264709308	0,228695152	-0,442300437	-0,004385073	0,384906175	0,248375564	0,514096945	0,069336813	-0,417552776
0,067874146	0,165366175	-0,256284222	0,166900288	0,266254133	-0,311940315	-0,036817404	0,132587661	0,206846717	0,139747853	-0,220046204	0,21120685	0,227524881	-0,284210845	-0,192263876	0,31175328	-0,182587041	-0,282018451	-0,270921633	0,325786062	-0,253086786	0,165402081	0,101931394	-0,264709308	1	-0,176461565	0,519100279	0,033000733	-0,30756454	-0,10054631	-0,289979728	-0,12431782	0,272210276
0,015840326	-0,101449886	0,462639742	-0,077005072	-0,194160066	0,224304983	0,210227574	-0,108552355	-0,412200227	-0,003086909	0,099337479	-0,337595629	-0,35410441	0,223985758	0,390759041	-0,142351773	0,112626885	0,170735895	0,148911223	-0,35528132	0,341878771	-0,377258962	0,019377555	0,228695152	-0,176461565	1	-0,397515911	-0,05357288	0,230710247	0,10207157	0,308525865	-0,069904939	-0,122399337
0,215331476	0,284317713	-0,569564588	0,206483645	0,327145736	-0,423508149	-0,327674179	0,014202463	0,561636603	0,021227944	-0,423883736	0,447625067	0,53966574	-0,515721741	-0,417334552	0,394711998	-0,373938876	-0,501890692	-0,348984846	0,718309674	-0,584024436	0,428530047	0,189841602	-0,442300437	0,519100279	-0,397515911	1	0,073115316	-0,40977392	-0,317803985	-0,386505307	-0,255128231	0,31718809
-0,269984467	-0,000504524	-0,027065302	-0,098556222	-0,219475342	-0,08451198	0,08464108	-0,180226381	-0,035309152	-0,11623221	-0,124322629	-0,23194103	-0,22602776	0,049328872	-0,058096042	-0,152948316	0,237432613	0,0897978	0,052099102	-0,179978797	-0,08780985	-0,05647749	-0,216341618	-0,004385073	0,033000733	-0,05357288	0,073115316	1	0,336480395	0,308402551	-0,165034221	0,335000295	-0,125761581
-0,360256464	-0,339695973	0,383069689	-0,338097119	-0,416064263	0,186868012	0,226766711	-0,249829329	-0,177149895	0,015812205	0,100549556	-0,213303302	-0,309038608	0,554385646	0,141625387	-0,51931887	0,32105836	0,549153114	0,309248164	-0,353826907	0,259254085	-0,082326464	-0,431485331	0,384906175	-0,30756454	0,230710247	-0,40977392	0,336480395	1	0,535980216	0,232875855	0,29420869	-0,501733061
-0,409671647	-0,265529513	0,330831262	-0,222184494	-0,297133185	0,140883867	0,162073775	-0,138961728	-0,05824374	0,010050347	0,204054704	-0,183400817	-0,286869341	0,393488091	0,056584818	-0,442070291	0,484320927	0,554433157	0,224165169	-0,232682832	0,145436378	-0,120548539	-0,431705678	0,248375564	-0,10054631	0,10207157	-0,317803985	0,308402551	0,535980216	1	0,179794509	0,335701548	-0,391195295
-0,088565268	-0,283409356	0,564303599	-0,267089008	-0,121754477	0,576177474	0,098315192	-0,181917986	-0,115922556	0,168453871	0,118525582	-0,109479253	-0,12721402	0,59441176	0,424564566	-0,478178875	0,094425696	0,40952262	0,471276304	-0,134973835	0,341480404	-0,122039969	-0,168993556	0,514096945	-0,289979728	0,308525865	-0,386505307	-0,165034221	0,232875855	0,179794509	1	-0,046545241	-0,378687472
-0,412476458	-0,196616045	0,037663255	-0,117918383	-0,291516982	-0,031522953	0,105840095	-0,116341237	-0,046807877	-0,078240662	0,176844412	-0,262409126	-0,300602431	0,125514756	0,13260111	-0,19564718	0,503980594	0,229015057	0,152536227	-0,316171419	0,221212581	-0,083808045	-0,276822856	0,069336813	-0,12431782	-0,069904939	-0,255128231	0,335000295	0,29420869	0,335701548	-0,046545241	1	-0,209905362
0,269840224	0,399422176	-0,407301425	0,555996812	0,300194767	-0,430398115	-0,204262763	0,295276774	0,015160945	-0,027933836	-0,151593319	0,075914849	0,192871436	-0,630710856	-0,11732823	0,677443262	-0,37802943	-0,566371782	-0,417071314	0,230783266	-0,25535368	0,061491632	0,53836766	-0,417552776	0,272210276	-0,122399337	0,31718809	-0,125761581	-0,501733061	-0,391195295	-0,378687472	-0,209905362	1
0,089932183	0,057495027	0,077843044	0,057930799	0,048852318	-0,015338736	-0,024011948	0,103139031	0,056719141	0,124917574	0,057185796	0,059789087	0,034901714	-0,06487893	-0,056273368	0,142945364	-0,176938186	-0,083385941	-0,109138986	0,008268741	-0,053012156	-0,130729442	0,178344716	-0,071451138	0,057258313	-0,057505943	0,026396375	-0,125252863	-0,14613476	-0,087488528	0,141600615	-0,162666668	0,223410147
-0,314144699	-0,123946914	0,116893178	-0,170310403	-0,384976054	-0,10191091	-0,078090545	-0,242961708	0,052416938	-0,079425737	0,086981533	-0,157159112	-0,198107205	0,146184664	-0,230674172	-0,297279172	0,230850031	0,382482055	0,16752019	-0,123263874	-0,055789036	0,05526928	-0,364718275	-0,025363708	-0,112190485	-0,014858579	-0,131858395	0,383687633	0,516073289	0,42854148	-0,041758393	0,320369889	-0,295431056
0,270090169	0,44183975	-0,288846525	0,308950194	0,525200649	-0,34406074	-0,114900948	0,364787596	0,117024048	0,206392025	-0,338175057	0,210598759	0,307480806	-0,475911361	-0,261359063	0,515507719	-0,491999184	-0,491078777	-0,292745883	0,444498369	-0,361866078	0,16934863	0,467684336	-0,330861619	0,353346592	-0,211084346	0,415394501	-0,168404756	-0,391479653	-0,325593361	-0,242510581	-0,325251119	0,443302195
-0,350605946	-0,086941348	0,023697402	0,06786387	-0,121109259	-0,101640919	-0,087692437	0,042773641	0,024051285	-0,038175527	0,20318109	-0,155748951	-0,204130808	0,043061802	-0,026930528	-0,087376214	0,077524241	0,270758727	0,082931895	-0,129911618	0,01004834	-0,065041342	-0,12102712	0,037343587	0,01794215	-0,044963271	-0,089975858	0,065844698	0,162841477	0,255266947	-0,167273015	0,188954758	-0,024272289
0,282858146	0,329123177	-0,336546002	0,406898333	0,202374437	-0,349493772	-0,140487993	0,226581474	0,079352234	-0,198737817	0,018047402	0,129481225	0,179518704	-0,384936443	-0,257862986	0,396872338	-0,257218404	-0,261247382	-0,370303679	0,242899041	-0,303497926	0,295924506	0,161116671	-0,402964002	0,052279066	-0,199311502	0,234481865	-0,135727778	-0,216180186	-0,185294058	-0,284901658	-0,198437531	0,454095418
0,256977814	0,323273767	-0,281925579	0,465298385	0,162678963	-0,363697635	-0,126973002	0,228330522	-0,018377545	0,019964343	-0,006620896	0,065232569	0,159813522	-0,44462499	-0,163442009	0,508341699	-0,309230625	-0,334165727	-0,326151845	0,167773073	-0,225969982	0,147389654	0,361170483	-0,357489938	0,126065128	-0,073954259	0,196999526	-0,163304848	-0,286972816	-0,178718072	-0,225070468	-0,187241308	0,621616905
0,330533106	0,514068744	-0,563831722	0,324095262	0,496654387	-0,364766384	-0,198506928	0,161218619	0,224259251	-0,205359644	-0,092546901	0,109482899	0,267684947	-0,468881618	-0,365858071	0,322769433	-0,270170297	-0,386861593	-0,485595097	0,393688996	-0,444846728	0,293462801	0,12434266	-0,494769202	0,262465942	-0,295895458	0,43906005	-0,032725922	-0,314142962	-0,167199279	-0,42319967	-0,203946469	0,3586681
-0,226731049	-0,455037455	0,695539156	-0,414200404	-0,298437986	0,646631774	0,326947138	-0,144271617	-0,356852635	0,177689814	0,167415542	-0,258083609	-0,372104012	0,715437701	0,532927557	-0,539923814	0,221959642	0,537796699	0,573879806	-0,458374623	0,559338493	-0,379122038	-0,16217269	0,714652068	-0,270572863	0,469415898	-0,609343698	-0,099610168	0,398625598	0,278703392	0,603777008	-0,022940651	-0,452198693
0,293060733	0,472423724	-0,488550994	0,363031791	0,369470481	-0,421998429	-0,164737838	0,200221518	0,14703026	-0,198625308	-0,026270218	0,047301604	0,171595092	-0,467927711	-0,328774991	0,379260705	-0,173772776	-0,34231709	-0,543586886	0,280248232	-0,383752874	0,293303684	0,125541508	-0,531396993	0,269051512	-0,252916079	0,356157315	-0,021941617	-0,244921384	-0,114398609	-0,498974751	-0,10272089	0,37556952
0,402909534	0,444578208	-0,533627853	0,487698038	0,375956838	-0,489804502	-0,138939606	0,423110247	-0,002701258	-0,125684316	-0,23673139	0,042674194	0,200959496	-0,654803799	-0,13501578	0,761022113	-0,476968325	-0,700255026	-0,521459802	0,215471453	-0,321163213	0,058434596	0,556881906	-0,463000323	0,229463488	-0,193985682	0,349839592	-0,179042395	-0,578597689	-0,526521595	-0,442742482	-0,199633927	0,646059604
0,29038265	0,480492258	-0,525219226	0,378446729	0,240297281	-0,604179218	-0,081315418	0,267033266	0,116618441	-0,140007879	-0,246888914	0,106456938	0,213824636	-0,669636845	-0,361384576	0,565664114	-0,226212423	-0,572211521	-0,635298059	0,334847428	-0,473715158	0,164064636	0,333571593	-0,649370395	0,359049697	-0,214172783	0,474940728	0,124645502	-0,347595046	-0,25800815	-0,523276739	-0,089584835	0,483594039
-0,071361981	-0,155372188	0,252202976	-0,229218052	-0,282260199	0,226451595	0,165448255	-0,166681879	-0,303551672	-0,034493798	0,013779256	-0,271053419	-0,283417738	0,242310881	0,104891273	-0,315359647	0,27791477	0,370102156	0,389170213	-0,288570562	0,042923526	-0,304511242	-0,212037116	0,191278824	-0,158520648	0,238025586	-0,190451262	0,292715292	0,300326577	0,316058614	0,16706581	0,089669554	-0,298574993
0,037813559	0,037223032	-0,023460285	-0,003037229	-0,047557368	-0,025512953	-0,028034858	-0,013079415	-0,101963483	-0,101856216	-0,143617784	-0,099889958	-0,054875702	-0,076112349	0,044539175	0,025327252	-0,004742293	-0,017219976	0,049341053	0,012849187	-0,067873831	-0,079618292	0,056343232	-0,031600267	-0,037349621	0,083013935	0,056424947	0,165063424	0,066351819	-0,021632758	-0,010408774	0,071035711	0,005483642
0,450668643	0,540496291	-0,518152946	0,357158547	0,467362658	-0,367259438	-0,157618223	0,191533895	0,159043655	-0,208283075	-0,137108561	0,136199737	0,330214396	-0,499286235	-0,329923363	0,402935288	-0,364717935	-0,435966568	-0,457445676	0,346267558	-0,436549808	0,250441338	0,224618286	-0,529647258	0,19772694	-0,279881967	0,438114954	-0,090998936	-0,372161387	-0,35145323	-0,333161848	-0,261557304	0,464972935
-0,559920723	-0,453752009	0,456573782	-0,311906967	-0,406994261	0,365401289	0,165321927	-0,263902058	-0,136889218	0,004150179	0,291803254	-0,236739349	-0,372011065	0,619975044	0,279318528	-0,593899515	0,433463992	0,528606345	0,394561499	-0,477653778	0,413630955	-0,21711726	-0,423934158	0,463487743	-0,347286982	0,200974644	-0,530992144	0,191922007	0,559256	0,446660126	0,366078104	0,362071374	-0,496891833
0,214209688	0,16468791	-0,19499782	0,147097134	0,046034584	-0,172621309	-0,161344041	0,101130167	-0,00624124	-0,169193905	0,073136263	-0,083896873	-0,018352223	-0,213185309	0,03353853	0,267057479	-0,162787015	-0,162920163	-0,302551865	-0,022642997	-0,113353544	-0,046996847	0,134958956	-0,201662068	0,097861577	-0,106710027	0,097566274	-0,104984976	-0,271052469	-0,168241466	-0,060338664	-0,043786697	0,261646488
0,475814941	0,458528527	-0,492650712	0,501217778	0,252528166	-0,546346154	-0,21759504	0,379472317	0,05034992	-0,151750286	-0,183581525	0,096215042	0,244754228	-0,707473222	-0,265564508	0,791368715	-0,459840823	-0,619545965	-0,494949509	0,254508068	-0,405678462	0,120310021	0,508707958	-0,544636206	0,206380515	-0,196488591	0,413824451	-0,189260441	-0,538564671	-0,464594442	-0,492280123	-0,248944967	0,666692203
-0,213038866	-0,25233572	0,420726515	-0,13682525	-0,176957911	0,359889477	0,075574588	-0,132184118	-0,367411267	0,066586714	0,413358212	-0,4275531	-0,406841658	0,395911824	0,511159123	-0,232308831	0,184406558	0,349798466	0,262501873	-0,302405607	0,500030461	-0,227781036	-0,05102987	0,412971727	-0,227038591	0,391783725	-0,491913016	-0,188104251	0,187180797	0,101310722	0,370436202	0,141635699	-0,191325591
0,293507439	0,114803963	-0,3282247	0,073873999	0,143381159	-0,195757964	-0,08021654	0,083637003	0,073220046	-0,02102446	-0,231949844	0,394201158	0,345369142	-0,303331874	-0,124479957	0,335435824	-0,089394369	-0,352471879	-0,28759688	0,133862645	-0,213394837	0,103651772	0,150638233	-0,13811574	0,136021423	-0,202617381	0,258357444	-0,14828333	-0,320134034	-0,38615981	-0,289327614	-0,133228528	0,23504704
0,072800948	0,099927987	-0,059403537	0,182511503	0,073938658	-0,192796846	-0,103519952	-0,007004197	0,14724456	-0,064472268	0,32924193	0,155479196	0,139641532	-0,210959258	-0,235813147	0,124626882	0,054103918	-0,03672366	-0,243238223	0,122768309	-0,067399413	0,071821404	0,015112436	-0,231811076	0,087908124	-0,146331704	0,097606045	-0,157213788	-0,135656448	-0,047155993	-0,187636037	0,007270021	0,141875365
-0,374982327	-0,360112009	0,308543014	-0,31782318	-0,345389327	0,409876609	0,062889012	-0,221335936	-0,091853413	0,073230501	0,177160073	-0,268001856	-0,382260313	0,566905718	0,278900937	-0,557818396	0,371005524	0,540661838	0,359888759	-0,460792759	0,338934723	-0,229713917	-0,40560421	0,439557231	-0,24266447	0,092633587	-0,41775445	0,1994545	0,464040047	0,420439768	0,227443715	0,249925869	-0,42068864
0,114057345	-0,157766414	0,19256036	-0,179303705	0,073577894	0,46017345	-0,053562048	-0,104900837	0,075321397	0,175398842	-0,113903393	0,066470341	0,057982903	0,248720035	0,275289218	-0,183612444	-0,18029816	0,007462597	0,231195369	0,122842544	0,128543531	-0,089100278	0,044531224	0,256307121	-0,08948142	0,236103292	0,002914902	-0,292204308	-0,101785938	-0,24002756	0,410320397	-0,374575343	-0,13383294
0,078270819	0,095390527	0,040092789	0,11132562	0,081486531	-0,096817552	-0,033208773	0,140227212	-0,10242774	-0,004951955	-0,044114953	-0,040382509	-0,004343299	-0,082829803	-0,019247056	0,192997797	-0,182558478	-0,067764052	0,048454564	-0,024780039	-0,032311499	0,010434061	0,207847335	-0,067748691	0,06983441	0,060544684	0,016300565	-0,171414814	-0,12083262	-0,111264837	-0,057552167	-0,097328334	0,179106903
0,176603971	0,18595977	-0,353092557	0,068094114	0,253031386	-0,129382637	-0,208615112	-0,07998598	0,389241504	-0,08647813	-0,201207124	0,243072828	0,335999222	-0,192746919	-0,24198763	-0,037831936	-0,151033919	-0,158276713	-0,220894834	0,491196291	-0,433622554	0,286809615	-0,143483213	-0,273553801	0,200964883	-0,311048138	0,459164289	0,031477937	-0,206565266	-0,141301243	-0,021431924	-0,223049509	0,056401598
0,257485151	0,244733765	-0,356845026	0,356119807	0,224758745	-0,306889626	-0,102171136	0,104014491	0,076241829	-0,184373224	0,169442433	0,223964409	0,255777367	-0,444939656	-0,299758977	0,377318989	-0,095191935	-0,237947391	-0,327993721	0,229073824	-0,179095728	0,180607606	0,185911796	-0,359462722	0,103626868	-0,165222588	0,205407472	-0,230017033	-0,346669136	-0,220878642	-0,426202813	-0,105292769	0,330916343
0,289372003	0,22629155	-0,43249385	0,296078388	0,074739475	-0,371408337	-0,084569045	0,143224869	-0,09339572	-0,315423289	0,004947282	0,044897423	0,118545853	-0,413259358	-0,150528499	0,495212727	-0,146177817	-0,383658923	-0,346487614	0,032882817	-0,1756901	0,061713399	0,304184204	-0,282180443	0,107262304	-0,140407231	0,234990843	-0,092588128	-0,403334599	-0,315744373	-0,434292397	-0,020793562	0,439160128
-0,078600192	-0,057946891	0,101240694	-0,116566847	-0,12097519	0,100117496	0,021428211	-0,074007338	0,000252023	0,041878035	0,133145469	-0,076753648	-0,148620129	0,156496886	0,109611548	-0,135810947	0,121863682	0,209877822	0,199766425	-0,246434786	0,177279462	-0,072819752	-0,1260649	0,141251498	-0,172946117	0,066198739	-0,207031817	0,080169163	0,191062148	0,093913947	0,072256708	0,210054558	-0,133554332
0,221098417	0,359814061	-0,367657569	0,310292535	0,464328975	-0,300476075	-0,264028062	0,154387883	0,140589761	-0,142133638	0,04539752	0,060439594	0,180681084	-0,479216754	-0,12915458	0,395689958	-0,284652387	-0,317151625	-0,394060497	0,379986051	-0,347978127	0,165020043	0,244523768	-0,383209815	0,258256925	-0,169140758	0,357822393	-0,140832914	-0,376236187	-0,198952361	-0,303845521	-0,18476444	0,387500454
0,183872784	0,322933986	-0,483172023	0,241813628	0,08789486	-0,510769826	-0,094153889	0,236800915	-0,004623843	-0,115553027	-0,179453445	0,035558187	0,121499513	-0,573725249	-0,297147043	0,529685224	-0,17318453	-0,505104001	-0,357636859	0,058094782	-0,345634299	0,086315	0,288864209	-0,487743393	0,137075872	-0,25113393	0,240771377	0,125663758	-0,223630618	-0,249248136	-0,553565847	0,101092196	0,345995376
0,274672499	0,077165691	0,031048481	0,068084462	0,030467039	-0,045494504	0,225427259	0,070285188	-0,209344909	-0,094825522	0,266514295	0,087644883	0,100016028	-0,189466656	0,074803827	0,166515344	0,170465619	-0,03572443	-0,035922636	-0,101553609	0,183365935	-0,105228036	0,153505778	-0,112337468	-0,051320796	0,142264924	-0,110246788	-0,172518202	-0,184791017	-0,101241548	-0,160583905	-0,101342683	0,093659816
0,231733784	0,076518545	-0,13848052	-0,032971724	0,004384722	-0,035250569	0,059312983	-0,142287316	0,101351259	-0,035399646	0,023210255	0,169437213	0,19034681	-0,07755897	-0,167207566	-0,042382866	0,197255953	-0,001137303	-0,111308548	-0,047028858	-0,100111961	-0,02461619	-0,225800962	-0,171717547	0,000494629	-0,119686957	0,046752107	0,087987162	-0,142073332	-0,058999477	0,123531288	0,111864218	-0,035240225
-0,151481043	-0,176748314	0,40514189	-0,250898328	-0,105274987	0,272234995	0,03400358	-0,051412352	0,011735494	0,285623712	0,015161241	0,068320011	-0,050926198	0,294576309	0,03661108	-0,270336936	-0,026150211	0,308593361	0,45158211	0,047865516	0,073071847	0,053886168	-0,088695572	0,240883162	-0,076929419	0,141915517	-0,206865304	-0,08385666	0,242009441	0,237924686	0,398451663	-0,050149312	-0,310772665
-0,052402846	-0,11640312	0,003017234	0,179061361	-0,094380579	-0,008654694	0,129918138	0,101166863	-0,264230256	-0,193591766	0,10905204	-0,142807126	-0,147788722	-0,015407598	0,20085919	0,155761325	0,157404949	-0,110715151	0,024028658	-0,20281558	0,239322462	0,020881325	0,207420048	0,031129985	-0,038506962	0,1579498	-0,161108167	-0,006708798	-0,079796489	-0,097564026	-0,027140039	0,189935539	0,152562022
0,083526235	0,294630174	-0,334235785	0,223693768	0,218123974	-0,303501516	-0,051982222	0,069181716	0,020661066	-0,218737002	-0,035636507	-0,129543399	0,024132654	-0,339422642	-0,194643991	0,185580686	-0,069131546	-0,237332725	-0,297489643	0,213067291	-0,309734032	0,054578777	0,057680735	-0,363100119	0,150201139	-0,047327724	0,243294589	0,174547321	-0,14467142	-0,062932983	-0,255417249	0,028658699	0,214182789
0,234287976	0,014782111	0,055654319	-0,051284398	0,098371798	0,168839437	0,070616239	0,0413407	-0,086851682	0,082917699	0,066939051	-0,080580721	0,013724612	0,168620839	0,027775212	-0,180160898	-0,148150555	0,136197633	0,015749224	-0,072970261	-0,037607654	-0,068057937	-0,031200837	0,021509012	0,049230723	-0,06657308	-0,058028613	-0,133291173	-0,088197931	-0,124370819	0,194027734	-0,242924186	-0,02745045
-0,29919571	-0,189956653	-0,080495834	-0,148341541	-0,052324493	0,109278619	-0,2512223	-0,216573272	0,502837624	0,176888184	-0,116701276	0,219621224	0,168423662	0,140886869	-0,0962174	-0,212471682	0,068293969	0,111571343	0,142517234	0,162197503	0,002740977	0,25487578	-0,261074105	0,09569945	-0,022949492	-0,29526158	0,198482934	0,254076462	0,194315287	0,161035503	0,032516116	0,184858794	-0,17326747
0,131465233	0,20361194	-0,275777292	0,204090811	0,160579327	-0,326800665	-0,125043243	0,243422807	0,02803565	0,151933622	-0,104745125	0,048602091	0,095691582	-0,437401769	-0,1364852	0,449638809	-0,194748397	-0,337361056	-0,389364503	0,117620976	-0,161784228	-0,028914497	0,331354869	-0,341226783	0,401197929	-0,187522517	0,269369413	-0,043607202	-0,387887527	-0,231354407	-0,447899699	-0,052800616	0,423282217
0,106386073	0,020284041	-0,046886966	0,000155348	-0,035124865	0,065916263	-0,06335919	-0,020808001	-0,099357774	-0,11298931	0,090155009	-0,249255075	-0,145509212	-0,063892479	-0,011958952	-0,006132455	0,089659904	-0,046666887	-0,05494824	-0,085221676	0,052915804	-0,086287892	0,060336468	-0,024897883	-0,02002018	0,056435087	-0,049872437	0,022995864	-0,091303117	0,019947495	-0,065841797	0,059627781	0,012298274
-0,230988156	-0,342974672	0,48056484	-0,234653693	-0,403949943	0,281315399	0,226909436	-0,14473598	-0,350646842	-0,051303353	0,277228595	-0,36939038	-0,454807969	0,582437162	0,35632917	-0,420741642	0,302632849	0,551146246	0,322096828	-0,545208838	0,451105492	-0,278685066	-0,322619249	0,504539729	-0,288393449	0,348009237	-0,596822349	0,097408133	0,564178798	0,389894591	0,311135945	0,232088964	-0,348380393
0,163844845	0,270670348	-0,349858636	0,225446706	0,281738203	-0,10093429	-0,138803275	0,171178202	-0,154624725	-0,272996615	-0,101514936	-0,17599403	0,00325519	-0,279518269	0,066869855	0,313887101	-0,285989196	-0,366266599	-0,130921747	0,093678963	-0,266724661	-0,10454534	0,325470121	-0,218144244	-0,0557277	-0,013785231	0,203531577	0,117726002	-0,186789914	-0,195127172	-0,153106568	-0,094843383	0,304048411
0,462790488	0,460361893	-0,592773641	0,443464922	0,306428029	-0,599413849	-0,193229337	0,369111107	0,086112814	-0,216477015	-0,273397578	0,165167539	0,327387292	-0,792221678	-0,257604785	0,813421776	-0,397760327	-0,746415824	-0,53886205	0,289346189	-0,41988282	0,142702192	0,536004534	-0,589924205	0,2103399	-0,209285282	0,46019338	-0,137630892	-0,557697152	-0,502842376	-0,529189583	-0,159945594	0,629201095
0,231472113	0,441381659	-0,409599596	0,452310791	0,413269282	-0,462545197	-0,16298478	0,359068199	0,034018493	-0,080579595	-0,096898034	0,054512328	0,125214809	-0,629953717	-0,221671963	0,660008989	-0,42698461	-0,574658736	-0,523581278	0,260556111	-0,320760648	0,075014234	0,509110925	-0,490458778	0,296023389	-0,18220226	0,359547288	-0,144522371	-0,519339922	-0,386741184	-0,438643187	-0,196627739	0,615128143
-0,31957981	-0,447849097	0,52978445	-0,409260382	-0,305403568	0,390825209	0,209723232	-0,213597397	-0,08815147	0,109561443	0,114611763	-0,108643631	-0,250706325	0,619966394	0,265601255	-0,573328504	0,408614501	0,651459824	0,444447147	-0,267545959	0,366878765	-0,159643079	-0,400424065	0,503835644	-0,238662903	0,27199373	-0,424671613	0,03745851	0,466535735	0,52192132	0,350310611	0,135971137	-0,542054461
-0,462065257	-0,359549835	0,439547161	-0,346753023	-0,360510832	0,348532617	-0,001989127	-0,287699684	-0,042200699	0,091849961	0,15500102	-0,260888907	-0,365145437	0,570552623	0,249717854	-0,585912401	0,289882212	0,557812686	0,374406884	-0,412519742	0,338109372	-0,248922185	-0,463718367	0,46018713	-0,250365483	0,227860282	-0,420986424	0,269735494	0,58678919	0,443717287	0,334454983	0,277973315	-0,491834838
-0,036160701	-0,220844089	0,190402011	-0,230761254	-0,358005638	0,198179908	0,281608298	-0,130546262	-0,347007066	-0,087194732	0,099283472	-0,231890188	-0,290816969	0,328898868	0,085381096	-0,254839772	0,373952476	0,243456227	0,206055871	-0,501146737	0,266328233	-0,277356485	-0,24306017	0,189615564	-0,184639008	0,208041278	-0,394640686	0,219318771	0,256237696	0,114209242	0,124582615	0,161796835	-0,282685918
-0,021794101	-0,046904399	-0,084951421	-0,189874467	0,050288874	0,039972648	-0,136610078	-0,13392865	0,378507412	0,233547172	-0,284075867	0,307526617	0,272010802	-0,033612507	-0,133056829	-0,102727317	-0,138766167	-0,059498527	0,133557842	0,333307368	-0,257887322	0,097106568	-0,14625005	0,014849409	0,331712549	-0,29313589	0,466392087	0,141924757	-0,036283588	-0,09366181	-0,1090026	-0,144740821	-0,080292417
-0,023957436	0,017709857	0,112379808	0,005207522	0,074662456	0,325270642	-0,007825106	0,01883427	-0,02915865	0,167880499	0,297525285	-0,188677544	-0,113067998	0,18090472	0,211617824	-0,151935376	0,104226265	0,07730384	-0,081104345	-0,210243267	0,352231177	-0,180832972	0,021420958	0,126405037	-0,176946927	0,149222562	-0,292755917	-0,105153493	0,010330267	-0,027118913	0,2405496	0,013910193	-0,029121196
0,316426546	0,18075109	-0,244425268	0,294991422	0,084054241	-0,292923977	-0,113502685	0,136298426	-0,012895487	-0,220035172	0,27013304	0,03033439	0,147843255	-0,331499142	-0,161424323	0,331419344	-0,085845529	-0,143319759	-0,2957144	0,033593916	-0,102050953	0,054036498	0,264786431	-0,34883431	-0,02238425	0,005581853	0,051390047	-0,262738722	-0,291204887	-0,133795149	-0,09674602	-0,029109977	0,290741338
-0,473623808	-0,339212647	0,35130593	-0,217720868	-0,316987564	0,185707972	0,221548695	-0,065754708	-0,13811652	0,09623655	0,166190666	-0,132566215	-0,245937777	0,466931693	0,265461699	-0,396370519	0,286183592	0,417736912	0,204809157	-0,308461795	0,390576275	-0,268309567	-0,274957048	0,402227012	-0,251576657	0,217489666	-0,451861644	0,138641606	0,556008365	0,355745604	0,233576471	0,258394468	-0,340305695
-0,351288206	-0,388531233	0,560164487	-0,34099576	-0,28226166	0,545598909	0,158551908	-0,31769103	-0,233431067	0,178848347	0,18326331	-0,249211245	-0,358591552	0,742086853	0,525447248	-0,56063696	0,239550194	0,521159829	0,396148453	-0,368000177	0,569536706	-0,354985849	-0,333755674	0,750749769	-0,196293505	0,335505591	-0,462702805	0,064272297	0,412170324	0,296220534	0,478686295	0,185128655	-0,452545379
0,071366356	-0,003642211	-0,112831512	0,085874264	-0,06438701	-0,105585841	0,205244041	0,095951798	-0,257077295	-0,220080139	0,042316737	-0,060109981	-0,090960856	-0,154883842	0,136162482	0,192469707	0,368074396	-0,154899362	-0,184172267	-0,249503564	0,107409544	-0,202194041	0,068488264	-0,00380306	-0,05850942	0,064965085	-0,147247889	0,07228533	-0,150639148	-0,123085228	-0,190295241	0,284944213	0,098372117
-0,167730711	-0,032406062	0,123567249	0,036901403	-0,200970891	0,0096684	0,218718586	0,1866684	-0,2852433	0,002009546	0,037444874	-0,402605902	-0,375119095	0,110349336	0,212182095	0,078929691	0,03196447	0,087477184	0,15005131	-0,4484461	0,237434676	-0,281093758	0,072502562	0,139870836	-0,129691446	0,170583214	-0,350663469	0,18145936	0,200858749	0,099858109	0,030232785	0,348450519	0,028505404
-0,109684265	-0,013558127	0,17348625	-0,099984812	-0,159329526	-0,028501557	0,267767582	-0,079205351	-0,174430596	0,027085969	0,066208293	-0,204130319	-0,275890851	0,10324326	-0,004001652	-0,159343401	0,148503608	0,183436886	0,070711563	-0,223644657	0,198380137	-0,108218297	-0,170546444	-0,000399606	0,077675974	0,145767479	-0,112102326	0,253478725	0,23084778	0,175100266	-0,073260542	0,1309046	-0,120043852
-0,162650317	0,029815238	-0,101853606	0,044378282	-0,169833116	-0,11642015	-0,009417304	-0,193789254	0,048221345	-0,30709623	0,165340448	-0,105081458	-0,046662433	-0,052126875	-0,144638225	-0,118799222	0,374019067	0,11718652	-0,063520318	-0,044767006	-0,144244178	0,107719407	-0,232933419	-0,104112587	-0,054214339	-0,072975919	0,030765007	0,364068842	0,099902575	0,327148792	-0,07092707	0,302774621	-0,114542104
0,204389205	0,280200742	-0,295218621	0,029156	0,107796909	-0,17823367	-0,109360563	-0,101936741	0,236983066	-0,079968649	-0,219737555	0,15431341	0,248684441	-0,305929992	-0,281529623	0,055419911	-0,083519805	-0,290048213	-0,254043322	0,391569663	-0,41725268	0,158502537	0,025276712	-0,38744821	0,14223114	-0,15397942	0,464547382	0,175158656	-0,206243806	-0,135915974	-0,199670976	-0,197774136	0,064241467
0,139314825	0,011626575	-0,151164884	0,070596591	-0,116505651	-0,189306192	-0,038023066	-0,043279095	-0,09949099	-0,286865562	0,282766412	-0,012757814	0,039621373	-0,133910828	-0,095701153	0,098943473	0,181555777	-0,006524589	-0,157074902	-0,217060085	0,051795862	0,031201177	-0,020557026	-0,200604129	-0,167878243	-0,103862494	-0,157846845	-0,066219752	-0,002209849	-0,018785022	-0,121176083	0,309943971	0,077725892
-0,098647929	-0,360292803	0,354945078	-0,303716059	-0,29711645	0,238859518	0,305644043	-0,177124698	-0,215651551	0,012308058	0,009088992	0,053842943	-0,177264882	0,41185186	0,171136488	-0,350287604	0,397716217	0,352899449	0,079043316	-0,365353504	0,330614145	-0,145417289	-0,284974671	0,333292515	-0,193182235	0,262214639	-0,399898438	0,064573217	0,300857871	0,116043576	0,255779051	0,163404124	-0,309383291
0,241530823	0,531357774	-0,459521921	0,411711565	0,409347986	-0,419592324	-0,166009156	0,204728961	0,155968092	-0,20544066	-0,070836956	0,138682438	0,257168743	-0,570789013	-0,378878846	0,441433418	-0,255660706	-0,44467055	-0,496340543	0,376608409	-0,455533012	0,194961047	0,270682966	-0,538895084	0,310777256	-0,183640673	0,434385832	0,006447613	-0,370173265	-0,205181904	-0,463770457	-0,206037403	0,501731343
0,236351238	0,343730906	-0,651888358	0,342746383	0,252110632	-0,540725303	-0,210737426	0,184036135	0,206503034	-0,226663774	-0,246471756	0,143002871	0,268741859	-0,660672969	-0,327944041	0,582343398	-0,209874414	-0,593791957	-0,443223176	0,24131968	-0,379272602	0,234554154	0,307168042	-0,540642137	0,27655039	-0,371396829	0,504374194	0,174177453	-0,467674445	-0,37741662	-0,598778663	0,035945818	0,488751837
0,17465512	0,372942269	-0,407246332	0,352848019	0,384051654	-0,274377923	-0,189540042	0,200912102	0,076784662	-0,181346552	-0,129076379	0,098891168	0,200729439	-0,514918219	-0,154760555	0,466118253	-0,343204381	-0,508754537	-0,415662404	0,300922905	-0,32803273	0,043203209	0,53845817	-0,388456452	0,120534242	-0,176468351	0,37616678	-0,125375359	-0,422374411	-0,342472808	-0,338500284	-0,171997711	0,532227752
-0,400523478	-0,36101595	0,517484782	-0,337005406	-0,343718301	0,466909842	0,282758523	-0,190159716	-0,236105197	0,263880845	0,11017667	-0,246219604	-0,396160869	0,727568793	0,313768032	-0,560019641	0,217514498	0,579941589	0,390795764	-0,395225506	0,451331912	-0,28271435	-0,377383073	0,626942911	-0,161482017	0,179369524	-0,455732896	0,210584413	0,55647572	0,37541053	0,358166923	0,146240238	-0,428270237
0,08321979	0,164759608	-0,198717783	-0,033143973	0,009767984	-0,136242637	0,051448397	-0,051941901	0,063873527	-0,113194373	-0,124843312	-0,016888528	0,030967506	-0,092296986	-0,257844814	-0,089203048	0,06810936	0,003760416	-0,184888515	0,112966984	-0,331641491	0,117210955	-0,176086995	-0,280568298	0,186377675	-0,211165091	0,232383527	0,295409977	0,074589893	0,052400787	-0,220278193	-0,032261113	-0,027854433
-0,239351037	-0,405526119	0,696230299	-0,333733825	-0,246383592	0,616611193	0,280970286	-0,075940437	-0,365418543	0,152950319	0,124915779	-0,295661349	-0,402657074	0,683745625	0,514577434	-0,493179347	0,152087181	0,441876819	0,559864119	-0,463839971	0,531714842	-0,370039595	-0,122746172	0,643584861	-0,261595665	0,47880633	-0,586596295	-0,054423617	0,349081656	0,208541148	0,620049067	-0,013337826	-0,418092577
-0,015408077	-0,058205537	-0,118597604	-0,028863025	0,061224214	-0,140460551	-0,13521816	-0,078468337	0,398212908	0,007970645	-0,156758353	0,267887959	0,220106105	-0,10001665	-0,247886342	-0,022177223	-0,003844262	-0,04127265	-0,129027589	0,432491419	-0,173441892	0,384675762	-0,092579391	-0,123677133	0,124440807	-0,261824505	0,366297725	-0,016316811	-0,06382381	0,045794603	-0,119338873	-0,056914057	-0,049135871
-0,125352444	-0,360111462	0,622985791	-0,298691368	-0,219554838	0,623690479	0,290250986	-0,138770452	-0,360749081	0,142788634	0,167287917	-0,285506533	-0,318310489	0,655609162	0,521065665	-0,485352852	0,163050055	0,433757068	0,372167616	-0,462159171	0,617165092	-0,425555501	-0,120328668	0,593921458	-0,301917417	0,43362041	-0,56728875	-0,138127203	0,269891791	0,122160624	0,631059198	-0,048498078	-0,322085596
-0,068732988	0,01167114	-0,051823151	-0,02258013	-0,130236769	0,007607	0,104754257	-0,191650259	0,019179323	0,013968436	0,014462812	-0,075742644	-0,061948281	0,02811659	-0,084267564	-0,217596867	0,184727737	0,0710797	0,070205073	-0,009664759	-0,073283208	0,054486114	-0,227352537	-0,137051078	0,013697362	-0,049738645	0,04367842	0,23047096	0,084158586	0,065619017	-0,041150748	0,086641022	-0,087748155
0,324848822	0,341737587	-0,25704935	0,110996793	0,352500657	-0,120412364	-0,162787372	0,093977415	0,111344641	0,002912546	-0,132626027	0,125194281	0,263635223	-0,147106433	-0,123314833	0,144162647	-0,314147405	-0,15368966	-0,263316236	0,233352174	-0,205060259	0,216598623	0,121677306	-0,231490199	0,059349748	-0,126949676	0,154369285	-0,113041871	-0,132758029	-0,153882045	0,032812092	-0,275909653	0,164774394
-0,25801886	-0,453358559	0,662045169	-0,371669513	-0,306653427	0,52953927	0,270712131	-0,167239744	-0,20305101	0,266661593	0,181311247	-0,24028793	-0,355350206	0,664327176	0,409832021	-0,539585495	0,25929572	0,580365873	0,517198483	-0,368620328	0,54395673	-0,206996674	-0,256587734	0,569324343	-0,302091755	0,394122334	-0,525543097	-0,059887353	0,448482814	0,355621821	0,591795245	0,085009746	-0,52092029
-0,038420273	-0,288547162	0,384203524	-0,370407888	-0,21746898	0,524046988	0,107074003	-0,234163072	-0,215409432	0,019391081	0,070660613	-0,113903454	-0,153772181	0,437165885	0,270366751	-0,443352242	0,209946688	0,400303173	0,439738843	-0,210949848	0,282145826	-0,133352854	-0,221215725	0,395179033	-0,258851847	0,204478548	-0,27747794	-0,007544255	0,228243364	0,187102728	0,439029931	0,014280113	-0,366115598
-0,265746567	-0,247756964	0,421227906	-0,140821943	-0,332353501	0,281114519	0,303429615	-0,055706304	-0,33989507	-0,0405659	0,296179403	-0,318607798	-0,434569721	0,381816992	0,210289364	-0,417151418	0,524261407	0,505708371	0,297352582	-0,423481671	0,260599818	-0,311665338	-0,28955643	0,253394774	-0,23731774	0,341748245	-0,500975688	0,185568566	0,383513672	0,593497301	0,236324686	0,218920787	-0,31139572
0,413455474	0,358480204	-0,37772196	0,353035516	0,185917333	-0,45314014	-0,055504685	0,323975332	0,01630227	-0,064453846	-0,256253625	0,21056543	0,33150226	-0,623342442	-0,139509895	0,705312783	-0,253874161	-0,544624509	-0,436126435	0,279786496	-0,334285838	0,168064761	0,467289191	-0,460828531	0,166005647	-0,087453061	0,33866042	-0,155572257	-0,434355232	-0,435930736	-0,349948507	-0,17253646	0,485459649
0,013176392	-0,008667443	-0,187375342	0,156050916	-0,043833804	-0,253123011	0,037054001	0,060091091	-0,056385162	-0,167852308	0,102080668	0,105631432	0,020921269	-0,165513475	0,021525495	0,305836675	0,226880158	-0,149186066	-0,238432233	-0,132996569	0,113521957	0,011343017	0,036930954	-0,035558846	-0,004702883	-0,109822546	-0,054456411	-0,013000041	-0,145554989	-0,134462691	-0,298267031	0,320489122	0,138775932
0,162304847	0,006125073	0,156876154	-0,002340138	0,175777509	0,055493843	0,101464255	0,173196017	0,195801554	0,245645854	-0,310406305	0,315606441	0,295130892	0,070929993	0,060327978	0,077475362	-0,310791093	-0,115882056	0,076800926	0,317071861	0,089567366	0,10088068	0,218289525	0,090048229	0,152869064	0,055186022	0,163446486	-0,299999949	-0,153122064	-0,226818252	0,250271527	-0,379314916	0,05985967
-0,096531087	-0,34748261	0,523991111	-0,333253169	-0,220820315	0,45075495	0,178594547	-0,194034558	-0,032572808	0,217260278	0,067984076	-0,080228605	-0,13094105	0,599353057	0,218008386	-0,51792037	0,090562334	0,4863978	0,448169465	-0,267838071	0,407198424	-0,14224089	-0,298717355	0,449499441	-0,218000396	0,202083624	-0,398010254	-0,021218016	0,327750494	0,234123958	0,616560712	0,007668765	-0,418646716
-0,5190034	-0,423404198	0,615351795	-0,366675834	-0,453530757	0,4538476	0,188180038	-0,238975218	-0,249125682	0,160800714	0,272455016	-0,369123473	-0,516136218	0,664932559	0,338828777	-0,573024278	0,353157077	0,703365843	0,535569246	-0,555587849	0,514600374	-0,297484101	-0,410971384	0,576266278	-0,293873277	0,324887996	-0,622319191	0,233559605	0,655977102	0,520447488	0,395534279	0,332170808	-0,511593223
-0,100788992	0,093287052	0,014963377	0,1666038	-0,010338397	-0,015738203	-0,089968884	0,02475901	-0,138747832	-0,265746616	0,352723664	-0,329428095	-0,255873923	-0,056867924	0,010822465	-0,055926192	0,141092427	0,086231844	-0,07937481	-0,020259954	-0,059027993	0,034913144	-0,070544915	-0,182220624	-0,015366317	0,085131004	-0,077203067	0,028051666	-0,026897989	0,198902911	-0,050872664	0,086350473	0,01547215

Supplementary Table S4A. Matrix of correlation coefficients for each comparison

NOTCH3	PKM2	PPARA	RAF1.pS338	RHEB	SCD	SGK1	SMARCB1	SOX17	SOX2	SPARC	STAT3.pS727	STAT3.pY705	TNFRSF4	TSC2	TYRO3	UGT1A1	WEE1	WIPI2	ASNS	BABAM1.pS29	BCL2	BMI1	CBX7	CDH1	FASN	FOS	FOXM1	G6PD	GATA1	GSKA_B	H3K27Ac	HSP90AA1_B1	IGFBP2
0,089932183	-0,314144699	0,270090169	-0,350605946	0,282858146	0,256977814	0,330533106	-0,226731049	0,293060733	0,402909534	0,29038265	-0,071361981	0,037813559	0,450668643	-0,559920723	0,214209688	0,475814941	-0,213038866	0,293507439	0,072800948	-0,374982327	0,114057345	0,078270819	0,176603971	0,257485151	0,289372003	-0,078600192	0,221098417	0,183872784	0,274672499	0,231733784	-0,151481043	-0,052402846	0,083526235
0,057495027	-0,123946914	0,44183975	-0,086941348	0,329123177	0,323273767	0,514068744	-0,455037455	0,472423724	0,444578208	0,480492258	-0,155372188	0,037223032	0,540496291	-0,453752009	0,16468791	0,458528527	-0,25233572	0,114803963	0,099927987	-0,360112009	-0,157766414	0,095390527	0,18595977	0,244733765	0,22629155	-0,057946891	0,359814061	0,322933986	0,077165691	0,076518545	-0,176748314	-0,11640312	0,294630174
0,077843044	0,116893178	-0,288846525	0,023697402	-0,336546002	-0,281925579	-0,563831722	0,695539156	-0,488550994	-0,533627853	-0,525219226	0,252202976	-0,023460285	-0,518152946	0,456573782	-0,19499782	-0,492650712	0,420726515	-0,3282247	-0,059403537	0,308543014	0,19256036	0,040092789	-0,353092557	-0,356845026	-0,43249385	0,101240694	-0,367657569	-0,483172023	0,031048481	-0,13848052	0,40514189	0,003017234	-0,334235785
0,057930799	-0,170310403	0,308950194	0,06786387	0,406898333	0,465298385	0,324095262	-0,414200404	0,363031791	0,487698038	0,378446729	-0,229218052	-0,003037229	0,357158547	-0,311906967	0,147097134	0,501217778	-0,13682525	0,073873999	0,182511503	-0,31782318	-0,179303705	0,11132562	0,068094114	0,356119807	0,296078388	-0,116566847	0,310292535	0,241813628	0,068084462	-0,032971724	-0,250898328	0,179061361	0,223693768
0,048852318	-0,384976054	0,525200649	-0,121109259	0,202374437	0,162678963	0,496654387	-0,298437986	0,369470481	0,375956838	0,240297281	-0,282260199	-0,047557368	0,467362658	-0,406994261	0,046034584	0,252528166	-0,176957911	0,143381159	0,073938658	-0,345389327	0,073577894	0,081486531	0,253031386	0,224758745	0,074739475	-0,12097519	0,464328975	0,08789486	0,030467039	0,004384722	-0,105274987	-0,094380579	0,218123974
-0,015338736	-0,10191091	-0,34406074	-0,101640919	-0,349493772	-0,363697635	-0,364766384	0,646631774	-0,421998429	-0,489804502	-0,604179218	0,226451595	-0,025512953	-0,367259438	0,365401289	-0,172621309	-0,546346154	0,359889477	-0,195757964	-0,192796846	0,409876609	0,46017345	-0,096817552	-0,129382637	-0,306889626	-0,371408337	0,100117496	-0,300476075	-0,510769826	-0,045494504	-0,035250569	0,272234995	-0,008654694	-0,303501516
-0,024011948	-0,078090545	-0,114900948	-0,087692437	-0,140487993	-0,126973002	-0,198506928	0,326947138	-0,164737838	-0,138939606	-0,081315418	0,165448255	-0,028034858	-0,157618223	0,165321927	-0,161344041	-0,21759504	0,075574588	-0,08021654	-0,103519952	0,062889012	-0,053562048	-0,033208773	-0,208615112	-0,102171136	-0,084569045	0,021428211	-0,264028062	-0,094153889	0,225427259	0,059312983	0,03400358	0,129918138	-0,051982222
0,103139031	-0,242961708	0,364787596	0,042773641	0,226581474	0,228330522	0,161218619	-0,144271617	0,200221518	0,423110247	0,267033266	-0,166681879	-0,013079415	0,191533895	-0,263902058	0,101130167	0,379472317	-0,132184118	0,083637003	-0,007004197	-0,221335936	-0,104900837	0,140227212	-0,07998598	0,104014491	0,143224869	-0,074007338	0,154387883	0,236800915	0,070285188	-0,142287316	-0,051412352	0,101166863	0,069181716
0,056719141	0,052416938	0,117024048	0,024051285	0,079352234	-0,018377545	0,224259251	-0,356852635	0,14703026	-0,002701258	0,116618441	-0,303551672	-0,101963483	0,159043655	-0,136889218	-0,00624124	0,05034992	-0,367411267	0,073220046	0,14724456	-0,091853413	0,075321397	-0,10242774	0,389241504	0,076241829	-0,09339572	0,000252023	0,140589761	-0,004623843	-0,209344909	0,101351259	0,011735494	-0,264230256	0,020661066
0,124917574	-0,079425737	0,206392025	-0,038175527	-0,198737817	0,019964343	-0,205359644	0,177689814	-0,198625308	-0,125684316	-0,140007879	-0,034493798	-0,101856216	-0,208283075	0,004150179	-0,169193905	-0,151750286	0,066586714	-0,02102446	-0,064472268	0,073230501	0,175398842	-0,004951955	-0,08647813	-0,184373224	-0,315423289	0,041878035	-0,142133638	-0,115553027	-0,094825522	-0,035399646	0,285623712	-0,193591766	-0,218737002
0,057185796	0,086981533	-0,338175057	0,20318109	0,018047402	-0,006620896	-0,092546901	0,167415542	-0,026270218	-0,23673139	-0,246888914	0,013779256	-0,143617784	-0,137108561	0,291803254	0,073136263	-0,183581525	0,413358212	-0,231949844	0,32924193	0,177160073	-0,113903393	-0,044114953	-0,201207124	0,169442433	0,004947282	0,133145469	0,04539572	-0,179453445	0,266514295	0,023210255	0,015161241	0,10905204	-0,035636507
0,059789087	-0,157159112	0,210598759	-0,155748951	0,129481225	0,065232569	0,109482899	-0,258083609	0,047301604	0,042674194	0,106456938	-0,271053419	-0,099889958	0,136199737	-0,236739349	-0,083896873	0,096215042	-0,4275531	0,394201158	0,155479196	-0,268001856	0,066470341	-0,040382509	0,243072828	0,223964409	0,044897423	-0,076753648	0,060439594	0,035558187	0,087644883	0,169437213	0,068320011	-0,142807126	-0,129543399
0,034901714	-0,198107205	0,307480806	-0,204130808	0,179518704	0,159813522	0,267684947	-0,372104012	0,171595092	0,200959496	0,213824636	-0,283417738	-0,054875702	0,330214396	-0,372011065	-0,018352223	0,244754228	-0,406841658	0,345369142	0,139641532	-0,382260313	0,057982903	-0,004343299	0,335999222	0,255777367	0,118545853	-0,148620129	0,180681084	0,121499513	0,100016028	0,19034681	-0,050926198	-0,147788722	0,024132654
-0,06487893	0,146184664	-0,475911361	0,043061802	-0,384936443	-0,44462499	-0,468881618	0,715437701	-0,467927711	-0,654803799	-0,669636845	0,242310881	-0,076112349	-0,499286235	0,619975044	-0,213185309	-0,707473222	0,395911824	-0,303331874	-0,210959258	0,566905718	0,248720035	-0,082829803	-0,192746919	-0,444939656	-0,413259358	0,156496886	-0,479216764	-0,573725249	-0,189466656	-0,07755897	0,294576309	-0,015407598	-0,339422642
-0,056273368	-0,230674172	-0,261359063	-0,026930528	-0,257862986	-0,163442009	-0,365858071	0,532927557	-0,328774991	-0,13501578	-0,361384576	0,104891273	0,044539175	-0,329923363	0,279318528	0,03353853	-0,265564508	0,511159123	-0,124479957	-0,235813147	0,278900937	0,275289218	-0,019247056	-0,24198763	-0,299758977	-0,150528499	0,109611548	-0,12915458	-0,297147043	0,074803827	-0,167207566	0,03661108	0,20085919	-0,194643991
0,142945364	-0,297279172	0,515507719	-0,087376214	0,396872338	0,508341699	0,322769433	-0,539923814	0,379260705	0,761022113	0,565664114	-0,315359647	0,025327252	0,402935288	-0,593899515	0,267057479	0,791368715	-0,232308831	0,335435824	0,124626882	-0,557818396	-0,183612444	0,192997797	-0,037831936	0,377318989	0,495212727	-0,135810947	0,395689958	0,529685224	0,166515344	-0,042382866	-0,270336936	0,155761325	0,185580686
-0,176938186	0,230850031	-0,491999184	0,077524241	-0,257218404	-0,309230625	-0,270170297	0,221959642	-0,173772776	-0,476968325	-0,226212423	0,27791477	-0,004742293	-0,364717935	0,433463992	-0,162787015	-0,459840823	0,184406558	-0,089394369	0,054103918	0,371005524	-0,18029816	-0,182558478	-0,151033919	-0,095191935	-0,146177817	0,121863682	-0,284652387	-0,17318453	0,170465619	0,197255953	-0,026150211	0,157404949	-0,069131546
-0,083385941	0,382482055	-0,491078777	0,270758727	-0,261247382	-0,334165727	-0,386861593	0,537796699	-0,34231709	-0,700255026	-0,572211521	0,370102156	-0,017219976	-0,435966568	0,528606345	-0,162920163	-0,619545965	0,349798466	-0,352471879	-0,03672366	0,540661838	0,007462597	-0,067764052	-0,158276713	-0,237947391	-0,383658923	0,209877822	-0,317151625	-0,505104001	-0,03572443	-0,001137303	0,308593361	-0,110715151	-0,237332725
-0,109138986	0,16752019	-0,292745883	0,082931895	-0,370303679	-0,326151845	-0,485595097	0,573879806	-0,543586886	-0,521459802	-0,635298059	0,389170213	0,049341053	-0,457445676	0,394561499	-0,302551865	-0,494949509	0,262501873	-0,28759688	-0,243238223	0,359888759	0,231195369	0,048454564	-0,220894834	-0,327993721	-0,346487614	0,199766425	-0,394060497	-0,357636859	-0,035922636	-0,111308548	0,45158211	0,024028658	-0,297489643
0,008268741	-0,123263874	0,444498369	-0,129911618	0,242899041	0,167773073	0,393688996	-0,458374623	0,280248232	0,215471453	0,334847428	-0,288570562	0,012849187	0,346267558	-0,477653778	-0,022642997	0,254508068	-0,302405607	0,133862645	0,122768309	-0,460792759	0,122842544	-0,024780039	0,491196291	0,229073824	0,032882817	-0,246434786	0,379986051	0,058094782	-0,101553609	-0,047028858	0,047865516	-0,20281558	0,213067291
-0,053012156	-0,055789036	-0,361866078	0,01004834	-0,303497926	-0,225969982	-0,444846728	0,559338493	-0,383752874	-0,321163213	-0,473715158	0,042923526	-0,067873831	-0,436549808	0,413630955	-0,113353544	-0,405678462	0,500030461	-0,213394837	-0,067399413	0,338934723	0,128543531	-0,032311499	-0,433622554	-0,179095728	-0,1756901	0,177279462	-0,347978127	-0,345634299	0,183365935	-0,100111961	0,073071847	0,239322462	-0,309734032
-0,130729442	0,05526928	0,16934863	-0,065041342	0,295924506	0,147389654	0,293462801	-0,379122038	0,293303684	0,058434596	0,164064636	-0,304511242	-0,079618292	0,250441338	-0,21711726	-0,046996847	0,120310021	-0,227781036	0,103651772	0,071821404	-0,229713917	-0,089100278	0,010434061	0,286809615	0,180607606	0,061713399	-0,072819752	0,165020043	0,086315	-0,105228036	-0,02461619	0,053886168	0,020881325	0,054578777
0,178344716	-0,364718275	0,467684336	-0,12102712	0,161116671	0,361170483	0,12434266	-0,16217269	0,125541508	0,556881906	0,333571593	-0,212037116	0,056343232	0,224618286	-0,423934158	0,134958956	0,508707958	-0,05102987	0,150638233	0,015112436	-0,40560421	0,044531224	0,207847335	-0,143483213	0,185911796	0,304184204	-0,1260649	0,244523768	0,288864209	0,153505778	-0,225800962	-0,088695572	0,207420048	0,057680735
-0,071451138	-0,025363708	-0,330861619	0,037343587	-0,402964002	-0,357489938	-0,494769202	0,714652068	-0,531396993	-0,463000323	-0,649370395	0,191278824	-0,031600267	-0,529647258	0,463487743	-0,201662068	-0,544636206	0,412971727	-0,13811574	-0,231811076	0,439557231	0,256307121	-0,067748691	-0,273553801	-0,359462722	-0,282180443	0,141251498	-0,383210895	-0,487743393	-0,112337468	-0,171717547	0,240883162	0,031129985	-0,363100119
0,057258313	-0,112190485	0,353346592	0,01794215	0,052279066	0,126065128	0,262465942	-0,270572863	0,269051512	0,229463488	0,359049697	-0,158520648	-0,037349621	0,19772694	-0,347286982	0,097861577	0,206380515	-0,227038591	0,136021423	0,087908124	-0,24266447	-0,08948142	0,06983441	0,200964883	0,103626868	0,107262304	-0,172946117	0,258256925	0,137075872	-0,051320796	0,000494629	-0,076929419	-0,038506962	0,150201139
-0,057505943	-0,014858579	-0,211084346	-0,044963271	-0,199311502	-0,073954259	-0,295895458	0,469415898	-0,252916079	-0,193985682	-0,214172783	0,238025586	0,083013935	-0,279881967	0,200974644	-0,106710027	-0,196488591	0,391783725	-0,202617381	-0,146331704	0,092633587	0,236103292	0,060544684	-0,311048138	-0,165222588	-0,140407231	0,066198739	-0,169140758	-0,25113393	0,142264924	-0,119686957	0,141915517	0,1579498	-0,047327724
0,026396375	-0,131858395	0,415394501	-0,089975858	0,234481865	0,196999526	0,43906005	-0,609343698	0,356157315	0,349839592	0,474940728	-0,190451262	0,056424947	0,438114954	-0,530992144	0,097566274	0,413824451	-0,491913016	0,258357444	0,097606045	-0,41775445	0,002914902	0,016300565	0,459164289	0,205407472	0,234990843	-0,207031817	0,357822393	0,240771377	-0,110246788	0,046752107	-0,206865304	-0,161108167	0,243294589
-0,125252863	0,383687633	-0,168404756	0,065844698	-0,135727778	-0,163304848	-0,032725922	-0,099610168	-0,021941617	-0,179042395	0,124645502	0,292715292	0,165063424	-0,090998936	0,191922007	-0,104984976	-0,189260441	-0,188104251	-0,14828333	-0,157213788	0,1994545	-0,292204308	-0,171414814	0,031477937	-0,230017033	-0,092588128	0,080169163	-0,140832914	0,125663758	-0,172518202	0,087987162	-0,08385666	-0,006708798	0,174547321
-0,14613476	0,516073289	-0,391479653	0,162841477	-0,216180186	-0,286972816	-0,314142962	0,398625598	-0,244921384	-0,578597689	-0,347595046	0,300326577	0,066351819	-0,372161387	0,559256	-0,271052469	-0,538564671	0,187180797	-0,320134034	-0,135656448	0,464040047	-0,101785938	-0,12083262	-0,206565266	-0,346669136	-0,403334599	0,191062148	-0,376236187	-0,223630618	-0,184791017	-0,142073332	0,242009441	-0,079796489	-0,14467142
-0,087488528	0,42854148	-0,325593361	0,255266947	-0,185294058	-0,178718072	-0,167199279	0,278703392	-0,114398609	-0,526521595	-0,25800815	0,316058614	-0,021632758	-0,35145323	0,446660126	-0,168241466	-0,464594442	0,101310722	-0,38615981	-0,047155993	0,420439768	-0,24002756	-0,111264837	-0,141301243	-0,220878642	-0,315744373	0,093913947	-0,198952361	-0,249248136	-0,101241548	-0,058999477	0,237924686	-0,097564026	-0,062932983
0,141600615	-0,041758393	-0,242510581	-0,167273015	-0,284901658	-0,225070468	-0,42319967	0,603777008	-0,498974751	-0,442742482	-0,523276739	0,16706581	-0,010408774	-0,333161848	0,366078104	-0,060338664	-0,492280123	0,370436202	-0,289327614	-0,187636037	0,227443715	0,410320397	-0,057552167	-0,021431924	-0,426202813	-0,434292397	0,072256708	-0,303845521	-0,553565847	-0,160583905	0,123531288	0,398451663	-0,027140039	-0,255417249
-0,162666668	0,320369889	-0,325251119	0,188954758	-0,198437531	-0,187241308	-0,203946469	-0,022940651	-0,10272089	-0,199633927	-0,089584835	0,089669554	0,071035711	-0,261557304	0,362071374	-0,043786697	-0,248944967	0,141635699	-0,133228528	0,007270021	0,249925869	-0,374575343	-0,097328334	-0,223049509	-0,105292769	-0,020793562	0,210054558	-0,18476444	0,101092196	-0,101342683	0,111864218	-0,050149312	0,189935539	0,028658699
0,223410147	-0,295431056	0,443302195	-0,024272289	0,454095418	0,621616905	0,3586681	-0,452198693	0,37556952	0,646059604	0,483594039	-0,298574993	0,005483642	0,464972935	-0,496891833	0,261646488	0,666692203	-0,191325591	0,23504704	0,141875365	-0,42068864	-0,13383294	0,179106903	0,056401598	0,330916343	0,439160128	-0,133554332	0,387500454	0,345995376	0,093659816	-0,035240225	-0,310772665	0,152562022	0,214182789
1	-0,141122422	0,166562237	0,002875688	0,088651357	0,176182057	-0,059436056	-0,040698185	-0,033616547	0,121515513	0,09897482	-0,043536106	-0,072734645	0,114243933	-0,088789802	0,221665255	0,151003709	-0,078196392	0,056829752	0,189809421	-0,049582152	0,131121978	0,035926323	0,042779252	-0,025779699	-0,026329642	0,087117186	0,057465673	-0,038850251	-0,056021347	0,20554133	0,022298922	-0,201572368	-0,093831659
-0,141122422	1	-0,250027049	0,287544972	-0,07881711	-0,126190846	-0,158929553	-0,027668024	-0,067376458	-0,407075546	-0,093327586	0,258610741	0,044434373	-0,20731536	0,350011572	-0,134265546	-0,24508909	-0,018174057	-0,340538142	-0,00346621	0,255446811	-0,271374886	-0,019453737	-0,036980084	-0,128120691	-0,240457005	0,161700125	-0,22508525	0,06062516	-0,207054843	-0,039689932	0,331069339	-0,153607406	0,036624806
0,166562237	-0,250027049	1	0,013505676	0,265664502	0,273110609	0,362364135	-0,401140487	0,353194793	0,494106575	0,417001608	-0,23247232	0,005522835	0,391917214	-0,578145782	0,075826455	0,478428388	-0,242119897	0,177937745	0,105285201	-0,45979973	-0,04523993	0,138702351	0,133175179	0,221638331	0,171976005	-0,180364161	0,390781902	0,303514909	-0,001839512	-0,148708792	-0,024609127	-0,133715514	0,151160885
0,002875688	0,287544972	0,013505676	1	-0,000108339	0,02955935	0,024479027	-0,03467813	0,047813408	-0,138123591	-0,104191903	0,081620305	-0,041088772	-0,054612939	0,187348173	-0,026219013	-0,089064492	0,087040254	-0,285392754	0,108121813	0,167319076	-0,257176961	0,040242367	-0,110063015	0,045845731	-0,09499181	0,113057235	0,103559994	-0,000297108	-0,096620325	-0,265710637	0,069401515	-0,035113024	0,083098639
0,088651357	-0,07881711	0,265664502	-0,000108339	1	0,581306729	0,463334132	-0,421829455	0,545328056	0,33580583	0,383103771	-0,226535342	-0,036301042	0,518880763	-0,331053027	0,143037906	0,576266216	-0,233653271	0,11843042	0,229326246	-0,245771556	-0,165097961	0,073578977	0,162132824	0,390567119	0,334023207	-0,065880484	0,299783022	0,185353599	0,026617135	-0,002934143	-0,276890754	-0,043226384	0,140307081
0,176182057	-0,126190846	0,273110609	0,02955935	0,581306729	1	0,314026706	-0,321696292	0,401933987	0,383766782	0,413320727	-0,218475464	-0,00495516	0,344323036	-0,320298773	0,194097572	0,552040412	-0,148549851	0,117945816	0,159980178	-0,307544292	-0,076980347	0,121587627	0,033099943	0,257258151	0,351749126	-0,052249906	0,173296752	0,189307189	0,055171509	0,012831731	-0,18056946	0,014271042	0,104593664
-0,059436056	-0,158929553	0,362364135	0,024479027	0,463334132	0,314026706	1	-0,607318418	0,785730305	0,424317107	0,544433526	-0,269639699	-0,072350654	0,721573368	-0,445214424	0,129556229	0,437892843	-0,393036398	0,135507876	0,127647288	-0,330660385	-0,204421751	0,024791774	0,454086708	0,320771177	0,241539047	-0,124209532	0,632382246	0,181847248	-0,049940709	0,042515194	-0,410159253	-0,163916281	0,473878594
-0,040698185	-0,027668024	-0,401140487	-0,03467813	-0,421829455	-0,321696292	-0,607318418	1	-0,574390463	-0,557182038	-0,659306885	0,274997134	-0,005548422	-0,595355543	0,52149049	-0,237245819	-0,593411732	0,501244602	-0,316308199	-0,292067909	0,457266789	0,385895463	-0,0468423	-0,371371873	-0,41993888	-0,398818777	0,107632585	-0,488896423	-0,553101908	0,026272368	-0,218860032	0,433235851	0,075178942	-0,435698323
-0,033616547	-0,067376458	0,353194793	0,047813408	0,545328056	0,401933987	0,785730305	-0,574390463	1	0,402461721	0,639627047	-0,284690922	-0,040928106	0,637891873	-0,378088784	0,201117579	0,510926881	-0,29880474	0,089154323	0,236407198	-0,281121577	-0,299723459	0,072303405	0,274266673	0,347798805	0,229451392	-0,12516549	0,549094268	0,251313261	0,016448323	-0,062751547	-0,387986648	-0,082067181	0,390788199
0,121515513	-0,407075546	0,494106575	-0,138123591	0,33580583	0,383766782	0,424317107	-0,557182038	0,402461721	1	0,565202702	-0,29447206	0,036093962	0,563058851	-0,604605058	0,383644772	0,768279291	-0,242593676	0,416057465	0,094498333	-0,480776064	-0,143233973	0,131696038	0,104785799	0,372941393	0,555244986	-0,142941405	0,416532564	0,554596023	0,113629861	0,070897384	-0,46634691	0,173505232	0,242335711
0,09897482	-0,093327586	0,417001608	-0,104191903	0,383103771	0,413320727	0,544433526	-0,659306885	0,639627047	0,565202702	1	-0,236263591	0,104245194	0,545979218	-0,456440001	0,239709268	0,596526886	-0,405878852	0,235531789	0,172016932	-0,438704587	-0,258353198	0,059760455	0,269837932	0,276182753	0,390600923	-0,189477162	0,410778134	0,436250424	0,022400323	0,076205099	-0,411174786	-0,030658717	0,443330821
-0,043536106	0,258610741	-0,23247232	0,081620305	-0,226535342	-0,218475464	-0,269639699	0,274997134	-0,284690922	-0,29447206	-0,236263591	1	0,458567122	-0,251865341	0,187628557	-0,105388788	-0,223493408	0,092496887	-0,171261927	-0,171656989	0,309774728	-0,023637597	0,003601055	-0,053624019	-0,156670222	-0,113364682	0,091200698	-0,284356394	-0,076660986	0,050240578	0,068278309	0,122677478	-0,087887449	-0,003583388
-0,072734645	0,044434373	0,005522835	-0,041088772	-0,036301042	-0,00495516	-0,072350654	-0,005548422	-0,040928106	0,036093962	0,104245194	0,458567122	1	0,006217449	-0,022794302	0,001054238	0,058105679	0,032933025	0,001937893	-0,139435421	-0,005967009	-0,021557337	0,065040465	0,024270509	-0,036078855	0,077508855	-0,026838888	-0,06489378	0,094253995	-0,029800169	0,001199142	-0,063080987	0,015995003	0,063576926
0,114243933	-0,20731536	0,391917214	-0,054612939	0,518880763	0,344323036	0,721573368	-0,595355543	0,637891873	0,563058851	0,545979218	-0,251865341	0,006217449	1	-0,534707844	0,283508119	0,557313847	-0,365533581	0,210214645	0,139406603	-0,329112613	-0,115819236	0,055423055	0,471148946	0,32542284	0,336067117	-0,092738115	0,545907484	0,23317591	-0,018620357	0,176012855	-0,43456421	-0,136088514	0,417482801
-0,088789802	0,350011572	-0,578145782	0,187348173	-0,331053027	-0,320298773	-0,445214424	0,52149049	-0,378088784	-0,604605058	-0,456440001	0,187628557	-0,022794302	-0,534707844	1	-0,202318656	-0,649826077	0,291227457	-0,345809801	-0,100204958	0,550659467	0,002358793	-0,160136906	-0,24567064	-0,403372943	-0,408877385	0,225493069	-0,4504258	-0,343845511	-0,149433413	-0,03020536	0,215338049	0,088550755	-0,202978464
0,221665255	-0,134265546	0,075826455	-0,026219013	0,143037906	0,194097572	0,129556229	-0,237245819	0,201117579	0,383644772	0,239709268	-0,105388788	0,001054238	0,283508119	-0,202318656	1	0,32154046	0,026063387	0,174996654	0,130997395	-0,133902013	-0,066262021	0,04581537	0,158312832	0,101496794	0,304838009	-0,014308575	0,24019742	0,205375748	-0,032153765	0,26435241	-0,292074404	0,021553548	0,151403684
0,151003709	-0,24508909	0,478428388	-0,089064492	0,576266216	0,552040412	0,437892843	-0,593411732	0,510926881	0,768279291	0,596526886	-0,223493408	0,058105679	0,557313847	-0,649826077	0,32154046	1	-0,314078273	0,326011629	0,183168368	-0,515540288	-0,175599218	0,246474558	0,094270223	0,463085523	0,567765616	-0,110105077	0,36402045	0,489947429	0,115662093	0,042585184	-0,378430731	0,00822324	0,220235039
-0,078196392	-0,018174057	-0,242119897	0,087040254	-0,233653271	-0,148549851	-0,393036398	0,501244602	-0,29880474	-0,242593676	-0,405878852	0,092496887	0,032933025	-0,365533581	0,291227457	0,026063387	-0,314078273	1	-0,218113975	0,023843458	0,173905138	0,155802988	0,006500529	-0,371354851	-0,118775471	-0,150556157	0,045104199	-0,036151571	-0,330203589	0,110738293	-0,252631085	0,230800019	0,241383677	-0,147490448
0,056829752	-0,340538142	0,177937745	-0,285392754	0,11843042	0,117945816	0,135507876	-0,316308199	0,089154323	0,416057465	0,235531789	-0,171261927	0,001937893	0,210214645	-0,345809801	0,174996654	0,326011629	-0,218113975	1	0,129712002	-0,265968654	0,021521951	-0,015245089	-0,007696421	0,217467644	0,415960763	-0,110330926	0,064715012	0,326450036	0,160979384	0,389278233	-0,323582172	0,008259737	-0,120168187
0,189809421	-0,00346621	0,105285201	0,108121813	0,229326246	0,159980178	0,127647288	-0,292067909	0,236407198	0,094498333	0,172016932	-0,171656989	-0,139435421	0,139406603	-0,100204958	0,130997395	0,183168368	0,023843458	0,129712002	1	-0,228416687	-0,222611113	-0,0185013	0,003441244	0,380469554	0,149650229	-0,026387938	0,249849944	0,122542476	0,225867731	0,103451644	-0,091387059	0,012384464	0,09377121
-0,049582152	0,255446811	-0,45979973	0,167319076	-0,245771556	-0,307544292	-0,330660385	0,457266789	-0,281121577	-0,480776064	-0,438704587	0,309774728	-0,005967009	-0,329112613	0,550659467	-0,133902013	-0,515540288	0,173905138	-0,265968654	-0,228416687	1	0,107829176	-0,165880982	-0,099089782	-0,38591613	-0,319556432	0,323157333	-0,408191449	-0,316479459	-0,238076683	-0,0070529	0,039901778	-0,114417476	-0,298127252
0,131121978	-0,271374886	-0,04523993	-0,257176961	-0,165097961	-0,076980347	-0,204421751	0,385895463	-0,299723459	-0,143233973	-0,258353198	-0,023637597	-0,021557337	-0,115819236	0,002358793	-0,066262021	-0,175599218	0,155802988	0,021521951	-0,222611113	0,107829176	1	-0,038684654	0,096637711	-0,255001557	-0,256859992	-0,005987762	-0,186410054	-0,405845832	-0,188370135	0,050288781	0,177689383	-0,15968382	-0,250416562
0,035926323	-0,019453737	0,138702351	0,040242367	0,073578977	0,121587627	0,024791774	-0,0468423	0,072303405	0,131696038	0,059760455	0,003601055	0,065040465	0,055423055	-0,160136906	0,04581537	0,246474558	0,006500529	-0,015245089	-0,0185013	-0,165880982	-0,038684654	1	-0,077833934	0,048951708	0,040567903	-0,042006538	0,118737744	0,034425547	0,013705885	-0,158302634	0,053436409	0,00234345	0,069771819
0,042779252	-0,036980084	0,133175179	-0,110063015	0,162132824	0,033099943	0,454086708	-0,371371873	0,274266673	0,104785799	0,269837932	-0,053624019	0,024270509	0,471148946	-0,24567064	0,158312832	0,094270223	-0,371354851	-0,007696421	0,003441244	-0,099089782	0,096637711	-0,077833934	1	0,075305594	-0,038465082	-0,13143841	0,305485871	-0,066651102	-0,19110506	0,276594199	-0,253501487	-0,272571179	0,374041526
-0,025779699	-0,128120691	0,221638331	0,045845731	0,390567119	0,257258151	0,320771177	-0,41993888	0,347798805	0,372941393	0,276182753	-0,156670222	-0,036078855	0,32542284	-0,403372943	0,101496794	0,463085523	-0,118775471	0,217467644	0,380469554	-0,38591613	-0,255001557	0,048951708	0,075305594	1	0,460445796	-0,09638971	0,334952976	0,305314325	0,379391309	-0,009753966	-0,256635744	0,139788789	0,199953289
-0,026329642	-0,240457005	0,171976005	-0,09499181	0,334023207	0,351749126	0,241539047	-0,398818777	0,229451392	0,555244986	0,390600923	-0,113364682	0,077508855	0,336067117	-0,408877385	0,304838009	0,567765616	-0,150556157	0,415960763	0,149650229	-0,319556432	-0,256859992	0,040567903	-0,038465082	0,460445796	1	-0,06740285	0,157175482	0,43667942	0,1866785	0,128613775	-0,501672205	0,239147477	0,184517108
0,087117186	0,161700125	-0,180364161	0,113057235	-0,065880484	-0,052249906	-0,124209532	0,107632585	-0,12516549	-0,142941405	-0,189477162	0,091200698	-0,026838888	-0,092738115	0,225493069	-0,014308575	-0,110105077	0,045104199	-0,110330926	-0,026387938	0,323157333	-0,005987762	-0,042006538	-0,13143841	-0,09638971	-0,06740285	1	-0,172048643	-0,094090821	-0,047652561	0,099325624	0,012546757	-0,023545263	-0,132149003
0,057465673	-0,22508525	0,390781902	0,103559994	0,299783022	0,173296752	0,632382246	-0,488896423	0,549094268	0,416532564	0,410778134	-0,284356394	-0,06489378	0,545907484	-0,4504258	0,24019742	0,36402045	-0,036151571	0,064715012	0,249849944	-0,408191449	-0,186410054	0,118737744	0,305485871	0,334952976	0,157175482	-0,172048643	1	0,119225787	0,120545432	-0,138039471	-0,225383011	-0,007992572	0,463944748
-0,038850251	0,06062516	0,303514909	-0,000297108	0,185353599	0,189307189	0,181847248	-0,553101908	0,251313261	0,554596023	0,436250424	-0,076660986	0,094253995	0,23317591	-0,343845511	0,205375748	0,489947429	-0,330203589	0,326450036	0,122542476	-0,316479459	-0,405845832	0,034425547	-0,066651102	0,305314325	0,43667942	-0,094090821	0,119225787	1	0,187909741	0,093457561	-0,240364886	0,081994882	0,196360056
-0,056021347	-0,207054843	-0,001839512	-0,096620325	0,026617135	0,055171509	-0,049940709	0,026272368	0,016448323	0,113629861	0,022400323	0,050240578	-0,029800169	-0,018620357	-0,149433413	-0,032153765	0,115662093	0,110738293	0,160979384	0,225867731	-0,238076683	-0,188370135	0,013705885	-0,19110506	0,379391309	0,1866785	-0,047652561	0,120545432	0,187909741	1	-0,035743361	-0,080290767	0,247886193	0,031646058
0,20554133	-0,039689932	-0,148708792	-0,265710637	-0,002934143	0,012831731	0,042515194	-0,218860032	-0,062751547	0,070897384	0,076205099	0,068278309	0,001199142	0,176012855	-0,03020536	0,26435241	0,042585184	-0,252631085	0,389278233	0,103451644	-0,0070529	0,050288781	-0,158302634	0,276594199	-0,009753966	0,128613775	0,099325624	-0,138039471	0,093457561	-0,035743361	1	-0,20371707	-0,16703656	0,037633362
0,022298922	0,331069339	-0,024609127	0,069401515	-0,276890754	-0,18056946	-0,410159253	0,433235851	-0,387986648	-0,46634691	-0,411174786	0,122677478	-0,063080987	-0,43456421	0,215338049	-0,292074404	-0,378430731	0,230800019	-0,323582172	-0,091387059	0,039901778	0,177689383	0,053436409	-0,253501487	-0,256635744	-0,501672205	0,012546757	-0,225383011	-0,240364886	-0,080290767	-0,20371707	1	-0,121172824	-0,290457639
-0,201572368	-0,153607406	-0,133715514	-0,035113024	-0,043226384	0,014271042	-0,163916281	0,075178942	-0,082067181	0,173505232	-0,030658717	-0,087887449	0,015995003	-0,136088514	0,088550755	0,021553548	0,00822324	0,241383677	0,008259737	0,012384464	-0,114417476	-0,15968382	0,00234345	-0,272571179	0,139788789	0,239147477	-0,023545263	-0,007992572	0,081994882	0,247886193	-0,16703656	-0,121172824	1	0,024429891
-0,093831659	0,036624806	0,151160885	0,083098639	0,140307081	0,104593664	0,473878594	-0,435698323	0,390788199	0,242335711	0,443330821	-0,003583388	0,063576926	0,417482801	-0,202978464	0,151403684	0,220235039	-0,147490448	-0,120168187	0,09377121	-0,298127252	-0,250416562	0,069771819	0,374041526	0,199953289	0,184517108	-0,132149003	0,463944748	0,196360056	0,031646058	0,037633362	-0,290457639	0,024429891	1
0,29078008	-0,143991601	0,029322611	-0,059979158	0,050015258	0,071116881	0,131652099	0,087559744	0,073642481	0,002177501	0,038913014	0,066987723	-0,02259369	0,285693963	-0,059446805	0,194483294	0,008478809	-0,062962769	-0,008383796	-0,073412427	0,1786232	0,272070827	0,019944047	0,293277488	-0,121890814	-0,089064986	0,0012522	0,016339552	-0,210571544	-0,176493654	0,320833889	-0,09648605	-0,258894944	-0,008887026
-0,011811123	0,209993015	-0,138340896	0,091904292	-0,157111672	-0,184616803	-0,074607776	-0,061107485	-0,140609822	-0,224312863	-0,194772867	-0,168463889	-0,146291821	-0,11037342	0,221464418	-0,128998869	-0,249612858	-0,227120841	-0,079045826	-0,141659171	0,267815397	0,116777562	-0,228029694	0,150789848	-0,237738368	-0,309959251	0,207604062	-0,146400899	-0,107568096	-0,336263675	0,109574429	0,130862061	-0,223193216	-0,166407088
0,141269779	-0,131406741	0,269509924	-0,003233863	0,145282154	0,239217654	0,221443909	-0,358231418	0,344896124	0,435431306	0,415756246	-0,183843249	-0,054858356	0,215947686	-0,377035266	0,184298015	0,432989521	-0,179651946	0,224788344	0,193829678	-0,262231729	-0,194270376	0,054502025	0,002541011	0,205770516	0,221147335	-0,078568807	0,278453831	0,335425675	0,130871565	-0,005279688	-0,231263729	0,034754902	0,100221455
-0,092714163	0,063202413	-0,046367303	-0,049904305	0,001486096	0,019144363	0,083830053	-0,015723041	0,085533778	0,070087179	0,067306081	0,088118362	-0,000562624	0,068001401	-0,017136307	0,068419871	0,066015193	0,02377181	-0,062806105	0,035533833	-0,005999319	-0,040134062	0,015309248	0,011633918	0,016262752	0,132072031	-0,089776756	0,024048383	0,096585236	0,053833125	-0,043495118	-0,154761749	0,147397762	0,208478664
-0,092631314	0,269693069	-0,419896423	0,177241206	-0,219326622	-0,203751158	-0,340877614	0,557938811	-0,259047152	-0,457384809	-0,41877159	0,213876438	-0,053918058	-0,389784622	0,502973312	-0,123816371	-0,404998168	0,333744552	-0,271012281	-0,172880884	0,478649136	-0,033610119	0,001168103	-0,385010169	-0,276446644	-0,177922786	0,228323082	-0,341710752	-0,381552605	-0,095604653	-0,129507137	0,161804736	0,028917291	-0,22755683
-0,07957665	-0,160291843	0,156197354	-0,033958405	0,215746508	0,123740938	0,341578913	-0,278015824	0,231198393	0,418275472	0,252037849	0,007898786	0,131502298	0,376968113	-0,257404281	0,156181545	0,300112005	-0,083472476	0,001074504	-0,013721002	-0,27265187	-0,059653047	0,015564223	0,165189744	0,161266351	0,267024779	-0,113415833	0,397653733	0,280498295	-0,025208699	-0,071378295	-0,270311017	0,151969199	0,373938974
0,045118291	-0,279086811	0,433442897	-0,186022211	0,384789422	0,410538939	0,405913127	-0,642468867	0,448035512	0,823919199	0,635834094	-0,242352225	0,111913372	0,521807131	-0,647124472	0,364155084	0,818190592	-0,308249738	0,408590363	0,146738959	-0,549770589	-0,208416167	0,118170093	0,108639136	0,395712026	0,573312703	-0,156118369	0,392635016	0,619881242	0,181806613	0,083702832	-0,408841063	0,139456934	0,258274889
0,206384265	-0,32564207	0,538581789	-0,007857562	0,401149111	0,395504841	0,464988356	-0,536054134	0,504244548	0,666761397	0,587349668	-0,348389967	-0,042320175	0,492726523	-0,534773	0,336814935	0,633772941	-0,202010095	0,210488262	0,309776215	-0,49519286	-0,218168919	0,179228342	0,096786951	0,428767983	0,422187801	-0,166534917	0,58393282	0,361306217	0,101040027	-0,122079217	-0,350510963	0,067354765	0,309588839
-0,127264769	0,204739141	-0,397046163	0,198707491	-0,307423022	-0,33429826	-0,407015551	0,610029537	-0,373829106	-0,638474114	-0,538070999	0,303980087	-0,054937581	-0,453053413	0,491601648	-0,331961037	-0,595174483	0,268762586	-0,356966224	-0,200703617	0,513370794	0,130114253	-0,081446887	-0,201418028	-0,340032596	-0,419785889	0,175726742	-0,374906987	-0,506079473	-0,008934715	-0,196612242	0,292390632	-0,101888862	-0,29831919
-0,081611514	0,451040231	-0,437086848	0,219819664	-0,37865476	-0,355097463	-0,339979333	0,483522456	-0,331871583	-0,597709403	-0,457902002	0,233206929	-0,022413285	-0,455623876	0,687159562	-0,201981216	-0,609836925	0,195995751	-0,360672216	-0,186888841	0,594638927	0,097654222	-0,110676997	-0,150174787	-0,483740884	-0,493662396	0,212649187	-0,3655094	-0,385017195	-0,289922937	-0,031381607	0,297927183	-0,181391689	-0,233843702
-0,074856766	0,202772326	-0,442588661	-0,173000571	-0,240493076	-0,172874514	-0,312634737	0,340822017	-0,293370108	-0,222032361	-0,180925716	0,244661632	-0,025094327	-0,302681941	0,384295429	-0,033399976	-0,268662121	0,102662569	0,062855266	-0,173254158	0,226380644	0,032559562	-0,053137352	-0,281289997	-0,194785877	0,01503406	0,027229857	-0,476046043	-0,040951523	0,055184098	0,232772293	0,058373932	0,096189735	-0,101547479
0,141313306	0,080685236	0,145261174	-0,058426747	-0,088663283	-0,184340192	0,004447429	-0,093061642	-0,062231181	-0,159190626	0,033495613	0,104612202	0,050525093	0,017917789	-0,054320017	-0,167201591	-0,071672154	-0,336315521	0,075958668	-0,054077507	0,091499838	0,235655176	-0,095627838	0,318912985	-0,166860692	-0,187098824	-0,061362643	-0,061657758	0,007898871	-0,162863759	0,110953267	0,069219383	-0,352887864	-0,076521493
0,107599784	-0,20021979	-0,089078338	0,013402625	-0,052464149	-0,018969948	-0,001741078	0,194058327	0,037659206	-0,071190872	-0,096267346	-0,193376679	-0,129909416	-0,01128548	0,186528351	0,046701167	-0,176246795	0,150386146	-0,092783385	0,057371469	0,149464718	0,187273337	-0,14594755	-0,049073783	-0,126138881	-0,191113814	0,120395517	-0,061251102	-0,16871781	-0,038146347	0,070286011	-0,102682838	-0,006599583	-0,034545919
0,140921752	-0,073024834	0,11150926	0,031810399	0,324319951	0,378834873	0,202346102	-0,313253763	0,244799372	0,37696798	0,247630887	-0,079071016	0,007131777	0,333708895	-0,274613278	0,40348462	0,460678011	-0,024046029	0,080756046	0,309002339	-0,236966472	-0,21007996	0,072427621	0,091945197	0,409187069	0,38461025	0,066066115	0,276160699	0,21687091	0,270162621	0,213467187	-0,215925533	0,083675258	0,15752119
-0,126221667	0,252841837	-0,325322787	0,213439443	-0,291071374	-0,252911136	-0,430983365	0,395223795	-0,371733112	-0,438854092	-0,39027931	0,167541933	0,020723423	-0,426172318	0,542425672	-0,177819136	-0,535315338	0,316266411	-0,190893032	-0,090480164	0,414013969	-0,042395909	-0,094804685	-0,252016687	-0,249470093	-0,299944192	0,091811825	-0,349544981	-0,145245396	-0,043702795	-0,109849258	0,13615313	0,074942685	-0,1731092
-0,073632413	0,021806841	-0,393644163	0,05627485	-0,40464898	-0,379299216	-0,472066544	0,651751536	-0,467058925	-0,521175146	-0,568176984	0,144604761	-0,075158015	-0,488821925	0,520488732	-0,206604261	-0,61202819	0,4401433	-0,208437495	-0,207161557	0,485559092	0,211751028	-0,167835593	-0,250365134	-0,389920207	-0,304616699	0,065014214	-0,372011617	-0,51511274	-0,133054147	-0,117242266	0,174374791	0,025443221	-0,273763542
-0,118878587	-0,204230647	-0,11739512	-0,147758963	-0,078491513	-0,093528138	-0,065969291	-0,09676553	-0,026184428	0,304228972	0,091861737	0,008768503	0,068473376	-0,061248255	-0,036304977	0,109284428	0,118621735	0,108983668	0,454133055	0,108800952	-0,115262297	-0,208337867	-0,125820921	-0,228199904	0,190895267	0,414180993	-0,079766762	-0,044285014	0,297646419	0,284171509	0,239666545	-0,393669895	0,431717098	0,032337188
-0,026837192	0,102993103	-0,111167524	0,164001796	-0,140892232	-0,050980701	-0,172247292	0,143870051	-0,110507639	0,136599497	-0,091594897	0,120665521	0,058580099	-0,13290967	0,186149987	0,060003158	0,035267027	0,147391649	-0,124447127	-0,181401944	0,215774217	-0,139373111	0,065865179	-0,314766757	-0,148470064	0,017093951	0,315538327	-0,157968278	0,106333772	-0,21310335	-0,001970807	-0,003435107	0,127656666	-0,040812601
0,016331327	0,155030539	-0,140584813	0,096148652	-0,057263166	-0,070814399	-0,097768906	0,071202849	-0,075094343	-0,237508386	-0,003866202	0,113612366	-0,017339355	-0,131117672	0,103316173	-0,226101221	-0,156078383	-0,020525226	-0,161008117	-0,06156472	0,131419934	-0,060691274	-0,005568897	-0,072611606	-0,038445447	-0,083893616	0,083887792	-0,17179272	-0,037021051	-0,036753475	-0,009746269	0,006957285	-0,111104347	0,028800345
-0,168385766	0,28513505	-0,207665983	0,047218981	0,013289922	-0,068172189	0,094338394	-0,236388692	0,088788245	-0,072953941	0,167058834	0,177424926	0,119246769	0,031421007	0,089767979	0,035361063	-0,063697617	-0,010558186	-0,048508779	0,128879658	0,006907816	-0,3422906	-0,066368823	0,127198479	0,144047835	0,137579817	0,007842406	0,026714273	0,031498765	-0,004628715	0,168017908	-0,171759944	0,132574348	0,255951161
-0,102761587	0,031434996	0,095659459	-0,196219809	0,156835902	0,099978746	0,327831093	-0,355742713	0,278154573	0,131450962	0,403928753	0,046552049	0,076278313	0,290104113	-0,197325377	0,005833264	0,220710335	-0,337589307	0,058907847	-0,034673062	-0,193331423	0,014050123	-0,067562072	0,415649923	0,064985373	0,101347075	-0,19889581	0,126184884	0,104911617	-0,05295409	0,11233019	-0,194446415	-0,165877024	0,296400569
-0,027737384	0,088500763	-0,180099544	-0,06324467	0,131993306	0,117952718	0,026067756	-0,202003876	0,106957392	0,137130111	0,076527252	-0,081345453	-0,047856946	0,132210413	0,012856578	0,255999823	0,14121587	0,003874223	0,100385137	0,217413879	-0,032416156	-0,3202019	-0,012937889	-0,0448132	0,239633874	0,279188346	0,099234567	0,00733114	0,24592379	0,259402804	0,261838802	-0,219012222	0,259212135	0,093957867
-0,019115818	0,008086134	-0,440890163	-0,197961774	-0,272540224	-0,30491793	-0,373199297	0,385667239	-0,322744019	-0,291602589	-0,299791166	0,047361518	-0,058000883	-0,330421813	0,380513043	-0,066681614	-0,404725259	0,165545499	0,247088153	-0,124959718	0,276675554	0,124175616	-0,134292262	-0,243580203	-0,200652285	-0,073539509	0,052011559	-0,387876465	-0,203677922	0,03837779	0,251529861	0,003742605	0,160036146	-0,293712573
0,04353504	-0,113332001	0,475199063	0,067192111	0,491640248	0,302225891	0,681791045	-0,601032105	0,643009644	0,459790621	0,587019208	-0,204929826	0,035952249	0,650348682	-0,486035136	0,177013574	0,514593036	-0,310472345	0,068856914	0,190758589	-0,43878949	-0,264460386	0,10424105	0,350303338	0,416506599	0,338361985	-0,188381568	0,615620265	0,253812795	0,053741668	-0,020691641	-0,375246974	-0,01066654	0,489920204
-0,036745098	-0,139210435	0,287068456	-0,116888453	0,279089326	0,248937422	0,432783711	-0,679170077	0,400413753	0,697749721	0,575417875	-0,194731488	0,098912766	0,477534357	-0,455309824	0,265987486	0,626317729	-0,426807589	0,386750997	0,121545204	-0,334617427	-0,284567076	0,000735566	0,238718208	0,323848327	0,537965742	-0,063428893	0,322456411	0,564717181	0,053231724	0,179089589	-0,531679433	0,16410967	0,301886674
0,210914557	-0,333066704	0,428967652	-0,01327284	0,364297533	0,397557347	0,402066189	-0,4701861	0,396175744	0,562616519	0,485507224	-0,172886755	0,157203879	0,447092853	-0,439274935	0,275207222	0,50028241	-0,168147429	0,189026724	0,210969662	-0,413401441	-0,108241275	0,120763678	0,130761287	0,318672333	0,403164713	-0,158756015	0,452765316	0,304650801	0,135978387	-0,127193016	-0,39074695	0,110953066	0,292728508
0,009328661	0,138761371	-0,360001634	0,128738104	-0,333589086	-0,326321109	-0,414472148	0,613123723	-0,415944311	-0,548151187	-0,532502177	0,167522287	-0,087775079	-0,419675884	0,50137016	-0,262858332	-0,572280814	0,275119677	-0,223172243	-0,228356011	0,509505418	0,156066717	-0,124246701	-0,217623764	-0,365381774	-0,346176914	0,142457142	-0,436580242	-0,441018049	-0,227672112	-0,056872198	0,228523209	-0,116881768	-0,303435328
0,02365498	0,245755687	0,01862337	-0,072054858	0,288020757	0,074071533	0,31858347	-0,269788274	0,336972133	-0,02170861	0,39868673	0,126090398	0,152380286	0,270305032	-0,054808409	0,004023828	0,057083572	-0,323346311	-0,008014317	0,016215973	0,06516691	-0,112212374	-0,026522424	0,379510017	-0,008714675	-0,016782276	-0,065717365	0,064374293	0,087527817	-0,111721231	0,130761024	-0,228138914	-0,231018825	0,229677071
-0,023292841	-0,02297147	-0,349300201	-0,026315158	-0,426307036	-0,343439559	-0,557845835	0,90663242	-0,533516042	-0,49777685	-0,614167078	0,231237916	0,005725869	-0,562079341	0,532149682	-0,234998795	-0,564516862	0,444198454	-0,373231184	-0,32903843	0,437354215	0,39129291	-0,038065228	-0,33007192	-0,484986787	-0,441311974	0,108670154	-0,46295452	-0,532900681	-0,095506649	-0,213855844	0,419672671	0,109210946	-0,402400758
-0,090983154	0,011089943	0,02074237	-0,096297088	0,137510634	0,01121946	0,182393909	-0,219003575	0,196552802	-0,056651122	0,132035679	-0,067874399	0,031167095	0,036588985	-0,107784478	-0,104622193	0,052481936	-0,188692074	-0,007410202	0,116319063	-0,131321775	-0,080730228	-0,065725652	0,180949566	0,063912473	-0,005567578	-0,089150826	0,096756117	-0,039851079	-0,011899155	-0,097018535	0,020486015	-0,107034004	0,03641389
0,064396417	-0,158113618	-0,361748568	-0,135877502	-0,320932635	-0,245175917	-0,455768103	0,756642221	-0,449864196	-0,398104737	-0,531373822	0,140242326	-0,080306941	-0,368693761	0,452145847	-0,103991956	-0,491348485	0,380422423	-0,246888535	-0,194640508	0,368554118	0,415814608	-0,025699601	-0,235946715	-0,41335272	-0,327922503	0,055989942	-0,41235004	-0,512749472	-0,022026265	-0,058201837	0,131750133	0,076833866	-0,301829516
-0,032400281	0,196212905	-0,155223585	-0,031727462	-0,000468712	0,028622839	0,065671288	-0,05862919	0,091355419	-0,217614921	0,147935539	0,153348154	0,053373202	0,010290985	0,136757154	-0,096605925	-0,133391103	-0,04882795	-0,055562409	-0,041091601	0,088598981	-0,004261401	-0,058293642	0,200069908	-0,082196893	-0,114975426	-0,028327126	-0,113330975	-0,037458363	-0,071873689	0,155290977	-0,007674019	-0,127929348	0,115859679
0,111842779	-0,100779151	0,290530107	-0,064681448	0,188735412	0,161615852	0,420292998	-0,245655686	0,343537325	0,295586417	0,207188633	-0,192355172	-0,010139311	0,523466875	-0,26118147	0,185494018	0,181346398	-0,184168681	0,050371374	-0,070958978	-0,131612513	0,030911509	0,050807898	0,324952757	-0,018396578	-0,040787745	0,000152706	0,303521256	0,077247212	-0,111113346	0,182697375	-0,076102599	-0,149100672	0,154040881
-0,018913537	0,12026465	-0,41870961	0,036204977	-0,384288005	-0,320916047	-0,577196863	0,825789025	-0,515794117	-0,598690371	-0,617629076	0,191070038	-0,079489795	-0,590257996	0,541810954	-0,263894996	-0,595499283	0,451910205	-0,431631766	-0,226131704	0,419404163	0,273218828	-0,048344178	-0,337496309	-0,410328589	-0,507908994	0,166512517	-0,459062456	-0,524851373	-0,016547397	-0,229841153	0,523425661	0,003634959	-0,391827845
-0,128736567	0,042441511	-0,369315792	-0,171234262	-0,214907731	-0,215585982	-0,320247845	0,428740197	-0,410258346	-0,41298615	-0,418201012	0,356999104	0,090757116	-0,328784655	0,251171855	-0,176941085	-0,371507937	0,177093345	-0,114622555	-0,186861938	0,262990464	0,243883973	-0,076589099	-0,083807178	-0,262526652	-0,16391646	0,057906164	-0,342432584	-0,340007159	-0,008918545	0,070379321	0,20894165	-0,067981761	-0,209599729
-0,130597566	0,230142184	-0,386581229	0,146066972	-0,159964283	-0,195435239	-0,313971948	0,444455371	-0,238414115	-0,461589719	-0,306435023	0,466957567	0,102387391	-0,377220542	0,435979463	-0,165618147	-0,407963522	0,277736053	-0,397377222	-0,050376864	0,409331591	-0,06326346	-0,041353009	-0,250592999	-0,171252143	-0,236095852	0,092625273	-0,258620318	-0,280398038	0,059541825	-0,128672506	0,190142709	0,129705257	-0,032496653
0,031138396	-0,239366069	0,363972549	-0,229233146	0,29204928	0,347890223	0,198266808	-0,457352704	0,273734977	0,633694765	0,5173996	-0,169682717	0,105850184	0,314778393	-0,555748074	0,205316924	0,654747803	-0,168989776	0,418673591	0,138298482	-0,55713608	-0,152335959	0,119539337	0,022831678	0,414106875	0,454417083	-0,12223077	0,24630578	0,523244021	0,310337442	0,052234265	-0,22054014	0,209061767	0,162238918
-0,096698862	-0,172227233	-0,110648442	-0,054826719	0,091506289	-0,001170846	-0,045968407	-0,213919632	0,006842809	0,293644909	0,073645936	-0,194416132	-0,046685172	-0,012857537	-0,05986744	0,106217162	0,182225239	0,053631708	0,509727366	0,255659643	-0,158485094	-0,32622788	-0,066168421	-0,265108547	0,335014861	0,483287181	0,050077823	0,031403619	0,317194915	0,257843147	0,193081509	-0,328930099	0,378510371	-0,022443566
0,155818086	-0,260969332	0,263763376	-0,179936669	-0,063550299	-0,030362986	-0,058033753	0,180580525	-0,134891788	-0,01734625	-0,097730474	-0,146432075	-0,119895826	-0,033954068	-0,13697925	-0,129278184	0,00803725	-0,080142548	-0,046849866	-0,193966758	-0,113358619	0,373010732	0,141877117	0,094609322	-0,205932393	-0,261121062	-0,074705831	-0,042222159	-0,228503077	-0,046166479	-0,152039349	0,199636131	-0,118611797	-0,209222928
0,163464064	0,137346503	-0,309340847	-0,052621623	-0,333383668	-0,247775883	-0,435360843	0,607651089	-0,468291957	-0,495773387	-0,550886631	0,119135063	-0,113572994	-0,322846005	0,4163851	-0,120063072	-0,499283601	0,154356244	-0,280470588	-0,220853954	0,437119602	0,403358238	-0,06768203	-0,088384912	-0,48570358	-0,473463473	0,251116351	-0,47502066	-0,468393312	-0,246525083	0,209849216	0,416515726	-0,204795666	-0,377697998
-0,057148161	0,436236971	-0,463718063	0,286968219	-0,370654533	-0,312581528	-0,554830833	0,6503188	-0,469230871	-0,674523315	-0,607841148	0,272689435	-0,046605195	-0,603064214	0,676474075	-0,256914476	-0,640451689	0,387351237	-0,440368898	-0,174465829	0,589937363	0,037811816	-0,039561761	-0,387015028	-0,417718027	-0,487813859	0,309182859	-0,484273376	-0,387054288	-0,183376957	-0,14893634	0,4405936	-0,067960358	-0,317827981
-0,056978189	0,121586927	-0,077511036	0,127436703	0,215816815	0,112306417	0,276197013	-0,133901644	0,348066889	-0,015900109	0,185148489	0,03080223	0,027456095	0,174767421	0,076104959	0,141106233	0,099094172	0,330878907	-0,261384443	0,165491398	-0,009429244	-0,176650745	0,065291786	0,103543792	0,096241646	-0,025668674	-0,103272686	0,351658756	-0,114517939	-0,014406026	-0,137561872	-0,110964553	0,103818692	0,291486835

Supplementary Table S4A. Matrix of correlation coefficients for each comparison

IRS1	ITGAL	JAG1	MECOM	NLN	NOTCH1	NOTCH1.cle	PAX6	PDCD4	SGK3	SMAD2	STK4	SUZ12	AURORA_A_B_C.pT288_232_198	BRAF.pS445	EEF2K	EIF4E	GAB2	HK2	HSPB1.pS82	INPP4B	MAP1LC3A_B	MTOR	PDL1	PEA15	PIM2	PTEN	RAB11	SMARCA2	STAT1	TP53BP1	TUBA1A	XPF
0,234287976	-0,29919571	0,131465233	0,106386073	-0,230988156	0,163844845	0,462790488	0,231472113	-0,31957981	-0,462065257	-0,036160701	-0,021794101	-0,023957436	0,316426546	-0,473623808	-0,351288206	0,071366356	-0,167730711	-0,109684265	-0,162650317	0,204389205	0,139314825	-0,098647929	0,241530823	0,236351238	0,17465512	-0,400523478	0,08321979	-0,239351037	-0,015408077	-0,125352444	-0,068732988	0,324848822
0,014782111	-0,189956653	0,20361194	0,020284041	-0,342974672	0,270670348	0,460361893	0,441381659	-0,447849097	-0,359549835	-0,220844089	-0,046904399	0,017709857	0,18075109	-0,339212647	-0,388531233	-0,003642211	-0,032406062	-0,013558127	0,029815238	0,280200742	0,011626575	-0,360292803	0,531357774	0,343730906	0,372942269	-0,36101595	0,164759608	-0,405526119	-0,058205537	-0,360111462	0,01167114	0,341737587
0,055654319	-0,080495834	-0,275777292	-0,046886966	0,48056484	-0,349858636	-0,592773641	-0,409599596	0,52978445	0,439547161	0,190402011	-0,084951421	0,112379808	-0,244425268	0,35130593	0,560164487	-0,112831512	0,123567249	0,17348625	-0,101853606	-0,295218621	-0,15116488	0,354945078	-0,459521921	-0,651888358	-0,407246332	0,517484782	-0,198717783	0,696230299	-0,118597604	0,622985791	-0,051823151	-0,25704935
-0,051284398	-0,148341541	0,204090811	0,000155348	-0,234653693	0,225446706	0,443464922	0,452310791	-0,409260382	-0,346753023	-0,230761254	-0,189874467	0,005207522	0,294991422	-0,217720868	-0,34099576	0,085874264	0,036901403	-0,099984812	0,044378282	0,029156	0,070596591	-0,303716059	0,411711565	0,342746383	0,352848019	-0,337005406	-0,033143973	-0,333733825	-0,028863025	-0,298691368	-0,02258013	0,110996793
0,098371798	-0,052324493	0,160579327	-0,035124865	-0,403949943	0,281738203	0,306428029	0,413269282	-0,305403568	-0,360510832	-0,358005638	0,050288874	0,074662456	0,084054241	-0,316987564	-0,28226166	-0,06438701	-0,200970891	-0,159329526	-0,169833116	0,107796909	-0,11650565	-0,29711645	0,409347986	0,252110632	0,384051654	-0,343718301	0,009767984	-0,246383592	0,061224214	-0,219554838	-0,130236769	0,352500657
0,168839437	0,109278619	-0,326800665	0,065916263	0,281315399	-0,10093429	-0,599413849	-0,462545197	0,390825209	0,348532617	0,198179908	0,039972648	0,325270642	-0,292923977	0,185707972	0,545598909	-0,105585841	0,0096684	-0,028501557	-0,11642015	-0,17823367	-0,18930619	0,238859518	-0,419592324	-0,540725303	-0,274377923	0,466909842	-0,136242637	0,616611193	-0,140460551	0,623690479	0,007607	-0,120412364
0,070616239	-0,2512223	-0,125043243	-0,06335919	0,226909436	-0,138803275	-0,193229337	-0,16298478	0,209723232	-0,001989127	0,281608298	-0,136610078	-0,007825106	-0,113502685	0,221548695	0,158551908	0,205244041	0,218718586	0,267767582	-0,009417304	-0,109360563	-0,03802307	0,305644043	-0,166009156	-0,210737426	-0,189540042	0,282758523	0,051448397	0,280970286	-0,13521816	0,290250986	0,104754257	-0,162787372
0,0413407	-0,216573272	0,243422807	-0,020808001	-0,14473598	0,171178202	0,369111107	0,359068199	-0,213597397	-0,287699684	-0,130546262	-0,13392865	0,01883427	0,136298426	-0,065754708	-0,31769103	0,095951798	0,1866684	-0,079205351	-0,193789254	-0,101936741	-0,04327909	-0,177124698	0,204728961	0,184036135	0,200912102	-0,190159716	-0,051941901	-0,075940437	-0,078468337	-0,138770452	-0,191650259	0,093977415
-0,086851682	0,502837624	0,02803565	-0,099357774	-0,350646842	-0,154624725	0,086112814	0,034018493	-0,08815147	-0,042200699	-0,347007066	0,378507412	-0,02915865	-0,012895487	-0,13811652	-0,233431067	-0,257077295	-0,2852433	-0,174430596	0,048221345	0,236983066	-0,09949099	-0,215651551	0,155968092	0,206503034	0,076784662	-0,236105197	0,063873527	-0,365418543	0,398212908	-0,360749081	0,019179323	0,111344641
0,082917699	0,176888184	0,151933622	-0,11298931	-0,051303353	-0,272996615	-0,216477015	-0,080579595	0,109561443	0,091849961	-0,087194732	0,233547172	0,167880499	-0,220035172	0,09623655	0,178848347	-0,220080139	0,002009546	0,027085969	-0,30709623	-0,079968649	-0,28686556	0,012308058	-0,20544066	-0,226663774	-0,181346552	0,263880845	-0,113194373	0,152950319	0,007970645	0,142788634	0,013968436	0,002912546
0,066939051	-0,116701276	-0,104745125	0,090155009	0,277228595	-0,101514936	-0,273397578	-0,096898034	0,114611763	0,15500102	0,099283472	-0,284075867	0,297525285	0,27013304	0,166190666	0,18326331	0,042316737	0,037444874	0,066208293	0,165340448	-0,219737555	0,282766412	0,009088992	-0,070836956	-0,246471756	-0,129076379	0,11017667	-0,124843312	0,124915779	-0,156758353	0,167287917	0,014462812	-0,132626027
-0,080580721	0,219621224	0,048602091	-0,249255075	-0,36939038	-0,17599403	0,165167539	0,054512328	-0,108643631	-0,260888907	-0,231890188	0,307526617	-0,188677544	0,03033439	-0,132566215	-0,249211245	-0,060109981	-0,402605902	-0,204130319	-0,105081458	0,15431341	-0,01275781	0,053842943	0,138682438	0,143002871	0,098891168	-0,246219604	-0,016888528	-0,295661349	0,267887959	-0,285506533	-0,075742644	0,125194281
0,013724612	0,168423662	0,095691582	-0,145509212	-0,454807969	0,00325519	0,327387292	0,125214809	-0,250706325	-0,365145437	-0,290816969	0,272010802	-0,113067998	0,147843255	-0,245937777	-0,358591552	-0,090960856	-0,375119095	-0,275890851	-0,046662433	0,248684441	0,039621373	-0,177264882	0,257168743	0,268741859	0,200729439	-0,396160869	0,030967506	-0,402657074	0,220106105	-0,318310489	-0,061948281	0,263635223
0,168620839	0,140886869	-0,437401769	-0,063892479	0,582437162	-0,279518269	-0,792221678	-0,629953717	0,619966394	0,570552623	0,328898868	-0,033612507	0,18090472	-0,331499142	0,466931693	0,742086853	-0,154883842	0,110349336	0,10324326	-0,052126875	-0,305929992	-0,13391083	0,41185186	-0,570789013	-0,660672969	-0,514918219	0,727568793	-0,092296986	0,683745625	-0,10001665	0,655609162	0,02811659	-0,147106433
0,027775212	-0,0962174	-0,1364852	-0,011958952	0,35632917	0,066869855	-0,257604785	-0,221671963	0,265601255	0,249717854	0,085381096	-0,133056829	0,211617824	-0,161424323	0,265461699	0,525447248	0,136162482	0,212182095	-0,004001652	-0,144638225	-0,281529623	-0,09570115	0,171136488	-0,378878846	-0,327944041	-0,154760555	0,313768032	-0,257844814	0,514577434	-0,247886342	0,521065665	-0,084267564	-0,123314833
-0,180160898	-0,212471682	0,449638809	-0,006132455	-0,420741642	0,313887101	0,813421776	0,660008989	-0,573328504	-0,585912401	-0,254839772	-0,102727317	-0,151935376	0,331419344	-0,396370519	-0,56063696	0,192469707	0,078929691	-0,159343401	-0,118799222	0,055419911	0,098943473	-0,350287604	0,441433418	0,582343398	0,466118253	-0,560019641	-0,089203048	-0,493179347	-0,022177223	-0,485352852	-0,217596867	0,144162647
-0,148150555	0,068293969	-0,194748397	0,089659904	0,302632849	-0,285989196	-0,397760327	-0,42698461	0,408614501	0,289882212	0,373952476	-0,138766167	0,104226265	-0,085845529	0,286183592	0,239550194	0,368074396	0,03196447	0,148503608	0,374019067	-0,083519805	0,181555777	0,397716217	-0,255660706	-0,209874414	-0,343204381	0,217514498	0,06810936	0,152087181	-0,003844262	0,163050055	0,184727737	-0,314147405
0,136197633	0,111571343	-0,337361056	-0,046666887	0,551146246	-0,366266599	-0,746415824	-0,574658736	0,651459824	0,557812686	0,243456227	-0,059498527	0,07730384	-0,143319759	0,417736912	0,521159829	-0,154899362	0,087477184	0,183436886	0,11718652	-0,290048213	-0,00652459	0,352899449	-0,44467055	-0,593791957	-0,508754537	0,579941589	0,003760416	0,441876819	-0,04127265	0,433757068	0,0710797	-0,15368966
0,015749224	0,142517234	-0,389364503	-0,05494824	0,322096828	-0,130921747	-0,53886205	-0,523581278	0,444447147	0,374406884	0,206055871	0,133557842	-0,081104345	-0,2957144	0,204809157	0,396148453	-0,184172267	0,15005131	0,070711563	-0,063520318	-0,254043322	-0,1570749	0,079043316	-0,496340543	-0,443223176	-0,415662404	0,390795764	-0,184888515	0,559864119	-0,129027589	0,372167616	0,070205073	-0,263316236
-0,072970261	0,162197503	0,117620976	-0,085221676	-0,545208838	0,093678963	0,289346189	0,260556111	-0,267545959	-0,412519742	-0,501146737	0,333307368	-0,210243267	0,033593916	-0,308461795	-0,368000177	-0,249503564	-0,4484461	-0,223644657	-0,044767006	0,391569663	-0,21706009	-0,365353504	0,376608409	0,24131968	0,300922905	-0,395225506	0,112966984	-0,463839971	0,432491419	-0,462159171	-0,009664759	0,233352174
-0,037607654	0,002740977	-0,161784228	0,052915804	0,451105492	-0,266724661	-0,41988282	-0,320760648	0,366878765	0,338109372	0,266328233	-0,257887322	0,352231177	-0,102050953	0,390576275	0,569536706	0,107409544	0,237434676	0,198380137	-0,144244178	-0,41725268	0,051795862	0,330614145	-0,455533012	-0,379272602	-0,32803273	0,451331912	-0,331641491	0,531714842	-0,173441892	0,617165092	-0,073283208	-0,205060259
-0,068057937	0,25487578	-0,028914497	-0,086287892	-0,278685066	-0,10454534	0,142702192	0,075014234	-0,159643079	-0,248922185	-0,277356485	0,097106568	-0,180832972	0,054036498	-0,268309567	-0,354985849	-0,202194041	-0,281093758	-0,108218297	0,107719407	0,158502537	0,031201177	-0,145417289	0,194961047	0,234554154	0,043203209	-0,28271435	0,117210955	-0,370039595	0,384675762	-0,425555501	0,054486114	0,216598623
-0,031200837	-0,261074105	0,331354869	0,060336468	-0,322619249	0,325470121	0,536004534	0,509110925	-0,400424065	-0,463718367	-0,24306017	-0,14625005	0,021420958	0,264786431	-0,274957048	-0,333755674	0,068488264	0,072502562	-0,170546444	-0,232933419	0,025276712	-0,02055703	-0,284974671	0,270682966	0,307168042	0,53845817	-0,377383073	-0,176086995	-0,122746172	-0,092579391	-0,120328668	-0,227352537	0,121677306
0,021509012	0,09569945	-0,341226783	-0,024897883	0,504539729	-0,218144244	-0,589924205	-0,490458778	0,503835644	0,46018713	0,189615564	0,014849409	0,126405037	-0,34883431	0,402227012	0,750749769	-0,00380306	0,139870836	-0,000399606	-0,104112587	-0,38744821	-0,20060413	0,333292515	-0,538895084	-0,540642137	-0,388456452	0,626942911	-0,280568298	0,643584861	-0,123677133	0,593921458	-0,137051078	-0,231490199
0,049230723	-0,022949492	0,401197929	-0,02002018	-0,288393449	-0,0557277	0,2103399	0,296023389	-0,238662903	-0,250365483	-0,184639008	0,331712549	-0,176946927	-0,02238425	-0,251576657	-0,196293505	-0,05850942	-0,129691446	0,077675974	-0,054214339	0,14223114	-0,16787824	-0,193182235	0,310777256	0,27655039	0,120534242	-0,161482017	0,186377675	-0,261595665	0,124440807	-0,301917417	0,013697362	0,059349748
-0,06657308	-0,29526158	-0,187522517	0,056435087	0,348009237	-0,013785231	-0,209285282	-0,18220226	0,27199373	0,227860282	0,208041278	-0,29313589	0,149222562	0,005581853	0,217489666	0,335505591	0,064965085	0,170583214	0,145767479	-0,072975919	-0,15397942	-0,10386249	0,262214639	-0,183640673	-0,371396829	-0,176468351	0,179369524	-0,211165091	0,47880633	-0,261824505	0,43362041	-0,049738645	-0,126949676
-0,058028613	0,198482934	0,269369413	-0,049872437	-0,596822349	0,203531577	0,46019338	0,359547288	-0,424671613	-0,420986424	-0,394640686	0,466392087	-0,292755917	0,051390047	-0,451861644	-0,462702805	-0,147247889	-0,350663469	-0,112102326	0,030765007	0,464547382	-0,15784684	-0,399898438	0,434385832	0,504374194	0,37616678	-0,455732896	0,232383527	-0,586596295	0,366297725	-0,56728875	0,04367842	0,154369285
-0,133291173	0,254076462	-0,043607202	0,022995864	0,097408133	0,117726002	-0,137630892	-0,144522371	0,03745851	0,269735494	0,219318771	0,141924757	-0,105153493	-0,262738722	0,138641606	0,064272297	0,07228533	0,18145936	0,253478725	0,364068842	0,175158656	-0,06621975	0,064573217	0,006447613	0,174177453	-0,125375359	0,210584413	0,295409977	-0,054423617	-0,016316811	-0,138127203	0,23047096	-0,113041871
-0,088197931	0,194315287	-0,387887527	-0,091303117	0,564178798	-0,186789914	-0,557697152	-0,519339922	0,466535735	0,58678919	0,256237696	-0,036283588	0,010330267	-0,291204887	0,556008365	0,412170324	-0,150639148	0,200858749	0,23084778	0,099902575	-0,206243806	-0,00220985	0,300857871	-0,370173265	-0,467674445	-0,422374411	0,55647572	0,074589893	0,349081656	-0,06382381	0,269891791	0,084158586	-0,132758029
-0,124370819	0,161035503	-0,231354407	0,019947495	0,389894591	-0,195127172	-0,502842376	-0,386741184	0,52192132	0,443717287	0,114209242	-0,09366181	-0,027118913	-0,133795149	0,355745604	0,296220534	-0,123085228	0,099858109	0,175100266	0,327148792	-0,135915974	-0,01878502	0,116043576	-0,205181904	-0,37741662	-0,342472808	0,37541053	0,052400787	0,208541148	0,045794603	0,122160624	0,065619017	-0,153882045
0,194027734	0,032516116	-0,447899699	-0,065841797	0,311135945	-0,153106568	-0,529189583	-0,438643187	0,350310611	0,334454983	0,124582615	-0,1090026	0,2405496	-0,09674602	0,233576471	0,478686295	-0,190295241	0,030232785	-0,073260542	-0,07092707	-0,199670976	-0,12117608	0,255779051	-0,463770457	-0,598778663	-0,338500284	0,358166923	-0,220278193	0,620049067	-0,119338873	0,631059198	-0,041150748	0,032812092
-0,242924186	0,184858794	-0,052800616	0,059627781	0,232088964	-0,094843383	-0,159945594	-0,196627739	0,135971137	0,277973315	0,161796835	-0,144740821	0,013910193	-0,029109977	0,258394468	0,185128655	0,284944213	0,348450519	0,1309046	0,302774621	-0,197774136	0,309943971	0,163404124	-0,206037403	0,035945818	-0,171997711	0,146240238	-0,032261113	-0,013337826	-0,056914057	-0,048498078	0,086641022	-0,275909653
-0,02745045	-0,17326747	0,423282217	0,012298274	-0,348380393	0,304048411	0,629201095	0,615128143	-0,542054461	-0,491834838	-0,282685918	-0,080292417	-0,029121196	0,290741338	-0,340305695	-0,452545379	0,098372117	0,028505404	-0,120043852	-0,114542104	0,064241467	0,077725892	-0,309383291	0,501731343	0,488751837	0,532227752	-0,428270237	-0,027854433	-0,418092577	-0,049135871	-0,322085596	-0,087748155	0,164774394
0,29078008	-0,011811123	0,141269779	-0,092714163	-0,092631314	-0,07957665	0,045118291	0,206384265	-0,127264769	-0,081611514	-0,074856766	0,141313306	0,107599784	0,140921752	-0,126221667	-0,073632413	-0,118878587	-0,026837192	0,016331327	-0,168385766	-0,102761587	-0,02773738	-0,019115818	0,04353504	-0,036745098	0,210914557	0,009328661	0,02365498	-0,023292841	-0,090983154	0,064396417	-0,032400281	0,111842779
-0,143991601	0,209993015	-0,131406741	0,063202413	0,269693069	-0,160291843	-0,279086811	-0,32564207	0,204739141	0,451040231	0,202772326	0,080685236	-0,20021979	-0,073024834	0,252841837	0,021806841	-0,204230647	0,102993103	0,155030539	0,28513505	0,031434996	0,088500763	0,008086134	-0,113332001	-0,139210435	-0,333066704	0,138761371	0,245755687	-0,02297147	0,011089943	-0,158113618	0,196212905	-0,100779151
0,029322611	-0,138340896	0,269509924	-0,046367303	-0,419896423	0,156197354	0,433442897	0,538581789	-0,397046163	-0,437086848	-0,442588661	0,145261174	-0,089078338	0,11150926	-0,325322787	-0,393644163	-0,11739512	-0,111167524	-0,140584813	-0,207665983	0,095659459	-0,18009954	-0,440890163	0,475199063	0,287068456	0,428967652	-0,360001634	0,01862337	-0,349300201	0,02074237	-0,361748568	-0,155223585	0,290530107
-0,059979158	0,091904292	-0,003233863	-0,049904305	0,177241206	-0,033958405	-0,186022211	-0,007857562	0,198707491	0,219819664	-0,173000571	-0,058426747	0,013402625	0,031810399	0,213439443	0,05627485	-0,147758963	0,164001796	0,096148652	0,047218981	-0,196219809	-0,06324467	-0,197961774	0,067192111	-0,116888453	-0,01327284	0,128738104	-0,072054858	-0,026315158	-0,096297088	-0,135877502	-0,031727462	-0,064681448
0,050015258	-0,157111672	0,145282154	0,001486096	-0,219326622	0,215746508	0,384789422	0,401149111	-0,307423022	-0,37865476	-0,240493076	-0,088663283	-0,052464149	0,324319951	-0,291071374	-0,40464898	-0,078491513	-0,140892232	-0,057263166	0,013289922	0,156835902	0,131993306	-0,272540224	0,491640248	0,279089326	0,364297533	-0,333589086	0,288020757	-0,426307036	0,137510634	-0,320932635	-0,000468712	0,188735412
0,071116881	-0,184616803	0,239217654	0,019144363	-0,203751158	0,123740938	0,410538939	0,395504841	-0,33429826	-0,355097463	-0,172874514	-0,184340192	-0,018969948	0,378834873	-0,252911136	-0,379299216	-0,093528138	-0,050980701	-0,070814399	-0,068172189	0,099978746	0,117952718	-0,30491793	0,302225891	0,248937422	0,397557347	-0,326321109	0,074071533	-0,343439559	0,01121946	-0,245175917	0,028622839	0,161615852
0,131652099	-0,074607776	0,221443909	0,083830053	-0,340877614	0,341578913	0,405913127	0,464988356	-0,407015551	-0,339979333	-0,312634737	0,004447429	-0,001741078	0,202346102	-0,430983365	-0,472066544	-0,065969291	-0,172247292	-0,097768906	0,094338394	0,327831093	0,026067756	-0,373199297	0,681791045	0,432783711	0,402066189	-0,414472148	0,31858347	-0,557845835	0,182393909	-0,455768103	0,065671288	0,420292998
0,087559744	-0,061107485	-0,358231418	-0,015723041	0,557938811	-0,278015824	-0,642468867	-0,536054134	0,610029537	0,483522456	0,340822017	-0,093061642	0,194058327	-0,313253763	0,395223795	0,651751536	-0,09676553	0,143870051	0,071202849	-0,236388692	-0,355742713	-0,20200388	0,385667239	-0,601032105	-0,679170077	-0,4701861	0,613123723	-0,269788274	0,90663242	-0,219003575	0,756642221	-0,05862919	-0,245655686
0,073642481	-0,140609822	0,344896124	0,085533778	-0,259047152	0,231198393	0,448035512	0,504244548	-0,373829106	-0,331871583	-0,293370108	-0,062231181	0,037659206	0,244799372	-0,371733112	-0,467058925	-0,026184428	-0,110507639	-0,075094343	0,088788245	0,278154573	0,106957392	-0,322744019	0,643009644	0,400413753	0,396175744	-0,415944311	0,336972133	-0,533516042	0,196552802	-0,449864196	0,091355419	0,343537325
0,002177501	-0,224312863	0,435431306	0,070087179	-0,457384809	0,418275472	0,823919199	0,666761397	-0,638474114	-0,597709403	-0,222032361	-0,159190626	-0,071190872	0,37696798	-0,438854092	-0,521175146	0,304228972	0,136599497	-0,237508386	-0,072953941	0,131450962	0,137130111	-0,291602589	0,459790621	0,697749721	0,562616519	-0,548151187	-0,02170861	-0,49777685	-0,056651122	-0,398104737	-0,217614921	0,295586417
0,038913014	-0,194772867	0,415756246	0,067306081	-0,41877159	0,252037849	0,635834094	0,587349668	-0,538070999	-0,457902002	-0,180925716	0,033495613	-0,096267346	0,247630887	-0,39027931	-0,568176984	0,091861737	-0,091594897	-0,003866202	0,167058834	0,403928753	0,076527252	-0,299791166	0,587019208	0,575417875	0,485507224	-0,532502177	0,39868673	-0,614167078	0,132035679	-0,531373822	0,147935539	0,207188633
0,066987723	-0,168463889	-0,183843249	0,088118362	0,213876438	0,007898786	-0,242352225	-0,348389967	0,303980087	0,233206929	0,244661632	0,104612202	-0,193376679	-0,079071016	0,167541933	0,144604761	0,008768503	0,120665521	0,113612366	0,177424926	0,046552049	-0,08134545	0,047361518	-0,204929826	-0,194731488	-0,172886755	0,167522287	0,126090398	0,231237916	-0,067874399	0,140242326	0,153348154	-0,192355172
-0,02259369	-0,146291821	-0,054858356	-0,000562624	-0,053918058	0,131502298	0,111913372	-0,042320175	-0,054937581	-0,022413285	-0,025094327	0,050525093	-0,129909416	0,007131777	0,020723423	-0,075158015	0,068473376	0,058580099	-0,017339355	0,119246769	0,076278313	-0,04785695	-0,058000883	0,035952249	0,098912766	0,157203879	-0,087775079	0,152380286	0,005725869	0,031167095	-0,080306941	0,053373202	-0,010139311
0,285693963	-0,11037342	0,215947686	0,068001401	-0,389784622	0,376968113	0,521807131	0,492726523	-0,453053413	-0,455623876	-0,302681941	0,017917789	-0,01128548	0,333708895	-0,426172318	-0,488821925	-0,061248255	-0,13290967	-0,131117672	0,031421007	0,290104113	0,132210413	-0,330421813	0,650348682	0,477534357	0,447092853	-0,419675884	0,270305032	-0,562079341	0,036588985	-0,368693761	0,010290985	0,523466875
-0,059446805	0,221464418	-0,377035266	-0,017136307	0,502973312	-0,257404281	-0,647124472	-0,534773	0,491601648	0,687159562	0,384295429	-0,054320017	0,186528351	-0,274613278	0,542425672	0,520488732	-0,036304977	0,186149987	0,103316173	0,089767979	-0,197325377	0,012856578	0,380513043	-0,486035136	-0,455309824	-0,439274935	0,50137016	-0,054808409	0,532149682	-0,107784478	0,452145847	0,136757154	-0,26118147
0,194483294	-0,128998869	0,184298015	0,068419871	-0,123816371	0,156181545	0,364155084	0,336814935	-0,331961037	-0,201981216	-0,033399976	-0,167201591	0,046701167	0,40348462	-0,177819136	-0,206604261	0,109284428	0,060003158	-0,226101221	0,035361063	0,005833264	0,255999823	-0,066681614	0,177013574	0,265987486	0,275207222	-0,262858332	0,004023828	-0,234998795	-0,104622193	-0,103991956	-0,096605925	0,185494018
0,008478809	-0,249612858	0,432989521	0,066015193	-0,404998168	0,300112005	0,818190592	0,633772941	-0,595174483	-0,609836925	-0,268662121	-0,071672154	-0,176246795	0,460678011	-0,535315338	-0,61202819	0,118621735	0,035267027	-0,156078383	-0,063697617	0,220710335	0,14121587	-0,404725259	0,514593036	0,626317729	0,50028241	-0,572280814	0,057083572	-0,564516862	0,052481936	-0,491348485	-0,133391103	0,181346398
-0,062962769	-0,227120841	-0,179651946	0,02377181	0,333744552	-0,083472476	-0,308249738	-0,202010095	0,268762586	0,195995751	0,102662569	-0,336315521	0,150386146	-0,024046029	0,316266411	0,4401433	0,108983668	0,147391649	-0,020525226	-0,010558186	-0,337589307	0,003874223	0,165545499	-0,310472345	-0,426807589	-0,168147429	0,275119677	-0,323346311	0,444198454	-0,188692074	0,380422423	-0,04882795	-0,184168681
-0,008383796	-0,079045826	0,224788344	-0,062806105	-0,271012281	0,001074504	0,408590363	0,210488262	-0,356966224	-0,360672216	0,062855266	0,075958668	-0,092783385	0,080756046	-0,190893032	-0,208437495	0,454133055	-0,124447127	-0,161008117	-0,048508779	0,058907847	0,100385137	0,247088153	0,068856914	0,386750997	0,189026724	-0,223172243	-0,008014317	-0,373231184	-0,007410202	-0,246888535	-0,055562409	0,050371374
-0,073412427	-0,141659171	0,193829678	0,035533833	-0,172880884	-0,013721002	0,146738959	0,309776215	-0,200703617	-0,186888841	-0,173254158	-0,054077507	0,057371469	0,309002339	-0,090480164	-0,207161557	0,108800952	-0,181401944	-0,06156472	0,128879658	-0,034673062	0,217413879	-0,124959718	0,190758589	0,121545204	0,210969662	-0,228356011	0,016215973	-0,32903843	0,116319063	-0,194640508	-0,041091601	-0,070958978
0,1786232	0,267815397	-0,262231729	-0,005999319	0,478649136	-0,27265187	-0,549770589	-0,49519286	0,513370794	0,594638927	0,226380644	0,091499838	0,149464718	-0,236966472	0,414013969	0,485559092	-0,115262297	0,215774217	0,131419934	0,006907816	-0,193331423	-0,03241616	0,276675554	-0,43878949	-0,334617427	-0,413401441	0,509505418	0,06516691	0,437354215	-0,131321775	0,368554118	0,088598981	-0,131612513
0,272070827	0,116777562	-0,194270376	-0,040134062	-0,033610119	-0,059653047	-0,208416167	-0,218168919	0,130114253	0,097654222	0,032559562	0,235655176	0,187273337	-0,21007996	-0,042395909	0,211751028	-0,208337867	-0,139373111	-0,060691274	-0,3422906	0,014050123	-0,3202019	0,124175616	-0,264460386	-0,284567076	-0,108241275	0,156066717	-0,112212374	0,39129291	-0,080730228	0,415814608	-0,004261401	0,030911509
0,019944047	-0,228029694	0,054502025	0,015309248	0,001168103	0,015564223	0,118170093	0,179228342	-0,081446887	-0,110676997	-0,053137352	-0,095627838	-0,14594755	0,072427621	-0,094804685	-0,167835593	-0,125820921	0,065865179	-0,005568897	-0,066368823	-0,067562072	-0,01293789	-0,134292262	0,10424105	0,000735566	0,120763678	-0,124246701	-0,026522424	-0,038065228	-0,065725652	-0,025699601	-0,058293642	0,050807898
0,293277488	0,150789848	0,002541011	0,011633918	-0,385010169	0,165189744	0,108639136	0,096786951	-0,201418028	-0,150174787	-0,281289997	0,318912985	-0,049073783	0,091945197	-0,252016687	-0,250365134	-0,228199904	-0,314766757	-0,072611606	0,127198479	0,415649923	-0,0448132	-0,243580203	0,350303338	0,238718208	0,130761287	-0,217623764	0,379510017	-0,33007192	0,180949566	-0,235946715	0,200069908	0,324952757
-0,121890814	-0,237738368	0,205770516	0,016262752	-0,276446644	0,161266351	0,395712026	0,428767983	-0,340032596	-0,483740884	-0,194785877	-0,166860692	-0,126138881	0,409187069	-0,249470093	-0,389920207	0,190895267	-0,148470064	-0,038445447	0,144047835	0,064985373	0,239633874	-0,200652285	0,416506599	0,323848327	0,318672333	-0,365381774	-0,008714675	-0,484986787	0,063912473	-0,41335272	-0,082196893	-0,018396578
-0,089064986	-0,309959251	0,221147335	0,132072031	-0,177922786	0,267024779	0,573312703	0,422187801	-0,419785889	-0,493662396	0,01503406	-0,187098824	-0,191113814	0,38461025	-0,299944192	-0,304616699	0,414180993	0,017093951	-0,083893616	0,137579817	0,101347075	0,279188346	-0,073539509	0,338361985	0,537965742	0,403164713	-0,346176914	-0,016782276	-0,441311974	-0,005567578	-0,327922503	-0,114975426	-0,040787745
0,0012522	0,207604062	-0,078568807	-0,089776756	0,228323082	-0,113415833	-0,156118369	-0,166534917	0,175726742	0,212649187	0,027229857	-0,061362643	0,120395517	0,066066115	0,091811825	0,065014214	-0,079766762	0,315538327	0,083887792	0,007842406	-0,19889581	0,099234567	0,052011559	-0,188381568	-0,063428893	-0,158756015	0,142457142	-0,065717365	0,108670154	-0,089150826	0,055989942	-0,028327126	0,000152706
0,016339552	-0,146400899	0,278453831	0,024048383	-0,341710752	0,397653733	0,392635016	0,58393282	-0,374906987	-0,3655094	-0,476046043	-0,061657758	-0,061251102	0,276160699	-0,349544981	-0,372011617	-0,044285014	-0,157968278	-0,17179272	0,026714273	0,126184884	0,00733114	-0,387876465	0,615620265	0,322456411	0,452765316	-0,436580242	0,064374293	-0,46295452	0,096756117	-0,41235004	-0,113330975	0,303521256
-0,210571544	-0,107568096	0,335425675	0,096585236	-0,381552605	0,280498295	0,619881242	0,361306217	-0,506079473	-0,385017195	-0,040951523	0,007898871	-0,16871781	0,21687091	-0,145245396	-0,51511274	0,297646419	0,106333772	-0,037021051	0,031498765	0,104911617	0,24592379	-0,203677922	0,253812795	0,564717181	0,304650801	-0,441018049	0,087527817	-0,532900681	-0,039851079	-0,512749472	-0,037458363	0,077247212
-0,176493654	-0,336263675	0,130871565	0,053833125	-0,095604653	-0,025208699	0,181806613	0,101040027	-0,008934715	-0,289922937	0,055184098	-0,162863759	-0,038146347	0,270162621	-0,043702795	-0,133054147	0,284171509	-0,21310335	-0,036753475	-0,004628715	-0,05295409	0,259402804	0,03837779	0,053741668	0,053231724	0,135978387	-0,227672112	-0,111721231	-0,095506649	-0,011899155	-0,022026265	-0,071873689	-0,111113346
0,320833889	0,109574429	-0,005279688	-0,043495118	-0,129507137	-0,071378295	0,083702832	-0,122079217	-0,196612242	-0,031381607	0,232772293	0,110953267	0,070286011	0,213467187	-0,109849258	-0,117242266	0,239666545	-0,001970807	-0,009746269	0,168017908	0,11233019	0,261838802	0,251529861	-0,020691641	0,179089589	-0,127193016	-0,056872198	0,130761024	-0,213855844	-0,097018535	-0,058201837	0,155290977	0,182697375
-0,09648605	0,130862061	-0,231263729	-0,154761749	0,161804736	-0,270311017	-0,408841063	-0,350510963	0,292390632	0,297927183	0,058373932	0,069219383	-0,102682838	-0,215925533	0,13615313	0,174374791	-0,393669895	-0,003435107	0,006957285	-0,171759944	-0,194446415	-0,21901222	0,003742605	-0,375246974	-0,531679433	-0,39074695	0,228523209	-0,228138914	0,419672671	0,020486015	0,131750133	-0,007674019	-0,076102599
-0,258894944	-0,223193216	0,034754902	0,147397762	0,028917291	0,151969199	0,139456934	0,067354765	-0,101888862	-0,181391689	0,096189735	-0,352887864	-0,006599583	0,083675258	0,074942685	0,025443221	0,431717098	0,127656666	-0,111104347	0,132574348	-0,165877024	0,259212135	0,160036146	-0,01066654	0,16410967	0,110953066	-0,116881768	-0,231018825	0,109210946	-0,107034004	0,076833866	-0,127929348	-0,149100672
-0,008887026	-0,166407088	0,100221455	0,208478664	-0,22755683	0,373938974	0,258274889	0,309588839	-0,29831919	-0,233843702	-0,101547479	-0,076521493	-0,034545919	0,15752119	-0,1731092	-0,273763542	0,032337188	-0,040812601	0,028800345	0,255951161	0,296400569	0,093957867	-0,293712573	0,489920204	0,301886674	0,292728508	-0,303435328	0,229677071	-0,402400758	0,03641389	-0,301829516	0,115859679	0,154040881
1	-0,088803716	0,094943235	0,051500029	0,029210954	-0,090059848	-0,117388059	-0,031212109	0,021466734	0,004373765	0,022007256	0,156274819	0,178932355	0,10885026	-0,159406557	0,049193268	-0,247790949	-0,05129075	0,032044927	-0,161163947	0,03336468	-0,02174214	0,00681381	0,01256827	-0,123516043	-0,04488084	0,128040527	0,255634161	0,07124554	-0,125671648	0,265811026	0,145779066	0,33853419
-0,088803716	1	-0,074984376	-0,197439433	-0,043938318	-0,154580381	-0,223124564	-0,241325619	0,163645332	0,373256595	-0,123306604	0,339511418	0,0953704	-0,252215858	0,079158557	0,08421234	-0,275981774	0,059263387	0,006575309	-0,034758921	-0,004830385	-0,10387531	-0,0026725	-0,189397048	0,054455512	-0,200503518	0,204107117	-0,040765148	-0,037923756	0,172665504	-0,109504264	0,043121972	0,045394115
0,094943235	-0,074984376	1	0,13050002	-0,292769505	0,099965351	0,420551305	0,465990262	-0,364713679	-0,303108152	-0,194314213	0,172382922	0,019033765	0,134684717	-0,347315532	-0,279704046	0,10437956	0,001720782	0,007401162	-0,188578895	0,113240811	0,064465918	-0,185040013	0,272200428	0,396459171	0,29647062	-0,259213334	0,127163029	-0,347429993	0,027460264	-0,299928384	-0,030227022	0,071964089
0,051500029	-0,197439433	0,13050002	1	0,03700496	0,109456651	0,062871685	0,028967667	-0,045920786	-0,065088414	0,209394851	-0,039458069	0,133832806	0,074255054	-0,148829551	0,010941616	0,137786532	-0,123179312	-0,107148561	0,095797959	0,202377024	0,150515494	-0,064903469	0,078153429	0,098492832	0,074116726	-0,150200337	0,097899987	-0,033993111	-0,000758447	0,095366454	0,061649553	-0,07149815
0,029210954	-0,043938318	-0,292769505	0,03700496	1	-0,255574669	-0,518090405	-0,419087609	0,513370634	0,537418518	0,395983741	-0,243513872	0,100822607	-0,151826296	0,409793175	0,545234271	0,026858056	0,299748027	0,162515591	0,023835204	-0,309772934	0,028191704	0,391851113	-0,414345088	-0,449261162	-0,463382884	0,518491413	-0,122564944	0,53346984	-0,152257522	0,47061955	-0,045147648	-0,171759059
-0,090059848	-0,154580381	0,099965351	0,109456651	-0,255574669	1	0,400805176	0,340531462	-0,382961932	-0,302222972	-0,157884686	-0,15546932	-0,034765852	0,212872012	-0,136751847	-0,247835747	0,154440874	0,105166437	-0,222605572	0,052576604	0,126440783	0,031179603	-0,321915072	0,350084556	0,341439594	0,457072984	-0,274716855	0,03514521	-0,199527897	-0,104359347	-0,172657385	-0,072125935	0,123130352
-0,117388059	-0,223124564	0,420551305	0,062871685	-0,518090405	0,400805176	1	0,630622867	-0,651082887	-0,658734091	-0,256141548	-0,108552846	-0,154240691	0,450652407	-0,46531935	-0,690196335	0,277815831	0,007306187	-0,27370112	0,001368599	0,189606046	0,233036465	-0,345589877	0,473879259	0,731761459	0,555027607	-0,733004484	0,00992466	-0,614969772	0,02166839	-0,545316643	-0,182825536	0,209512191
-0,031212109	-0,241325619	0,465990262	0,028967667	-0,419087609	0,340531462	0,630622867	1	-0,571874162	-0,515122421	-0,35232448	-0,099049964	-0,011194062	0,343659697	-0,415633135	-0,488746408	0,09495799	-0,01280761	-0,09783874	-0,086507106	0,112253528	0,016611402	-0,35908824	0,549442238	0,49762633	0,657180845	-0,497003035	0,053566537	-0,468988426	0,042418578	-0,402217114	-0,14993204	0,147785575
0,021466734	0,163645332	-0,364713679	-0,045920786	0,513370634	-0,382961932	-0,651082887	-0,571874162	1	0,519369596	0,222752497	-0,023551989	0,011990656	-0,270483728	0,431618997	0,539535093	-0,208608096	0,007625236	0,137993907	-0,01372606	-0,261494971	-0,13347252	0,295023386	-0,466074452	-0,572170554	-0,473312883	0,496759232	-0,108320349	0,52627486	0,009222484	0,477962694	-0,004084362	-0,18122401
0,004373765	0,373256595	-0,303108152	-0,065088414	0,537418518	-0,302222972	-0,658734091	-0,515122421	0,519369596	1	0,300959944	0,105932826	0,134331771	-0,349422537	0,464199121	0,510763081	-0,205849426	0,214592239	0,194531652	-0,022079552	-0,214763744	-0,07845442	0,320630881	-0,451631964	-0,474364296	-0,491726558	0,539576705	-0,029775036	0,506248696	-0,097865063	0,385800832	0,076114023	-0,130328699
0,022007256	-0,123306604	-0,194314213	0,209394851	0,395983741	-0,157884686	-0,256141548	-0,35232448	0,222752497	0,300959944	1	-0,129963533	0,006619973	-0,143461901	0,19002543	0,270100976	0,29269278	0,125778714	0,116377942	0,135461207	-0,041502224	0,11404506	0,505524644	-0,390675105	-0,152364728	-0,33920722	0,280800764	0,081962141	0,323686487	-0,207385873	0,34542828	0,162076862	-0,253974292
0,156274819	0,339511418	0,172382922	-0,039458069	-0,243513872	-0,15546932	-0,108552846	-0,099049964	-0,023551989	0,105932826	-0,129963533	1	-0,149189963	-0,395465898	-0,128141112	-0,00637979	-0,277695115	-0,275831741	0,157068382	-0,191077013	0,273661061	-0,28970462	-0,110874599	0,024908652	0,060013542	-0,081962835	0,091466767	0,324252279	-0,099562351	0,194641353	-0,127370079	0,216570796	-0,022698833
0,178932355	0,0953704	0,019033765	0,133832806	0,100822607	-0,034765852	-0,154240691	-0,011194062	0,011990656	0,134331771	0,006619973	-0,149189963	1	0,025337652	0,122287722	0,235227027	-0,021974039	0,049207769	0,050122138	-0,137268518	-0,153997375	-0,00268339	0,080313663	-0,071702875	-0,207135396	-0,01679864	0,172614778	-0,123548273	0,198134464	-0,166001542	0,404178661	-0,042096252	0,12091835
0,10885026	-0,252215858	0,134684717	0,074255054	-0,151826296	0,212872012	0,450652407	0,343659697	-0,270483728	-0,349422537	-0,143461901	-0,395465898	0,025337652	1	-0,205326922	-0,392843286	0,097329618	-0,016856889	-0,25505787	0,139392677	0,007515866	0,464715976	-0,225212853	0,246124316	0,254873954	0,323715225	-0,480897039	-0,075116029	-0,33652276	-0,081750481	-0,212417098	-0,149350895	0,226223318
-0,159406557	0,079158557	-0,347315532	-0,148829551	0,409793175	-0,136751847	-0,46531935	-0,415633135	0,431618997	0,464199121	0,19002543	-0,128141112	0,122287722	-0,205326922	1	0,477827514	0,049600203	0,178727945	0,079644704	0,05404086	-0,341099149	0,021988807	0,323166633	-0,359484484	-0,421123883	-0,346937629	0,473721866	-0,204102563	0,35878645	-0,218019282	0,362841266	-0,01074557	-0,202708451
0,049193268	0,08421234	-0,279704046	0,010941616	0,545234271	-0,247835747	-0,690196335	-0,488746408	0,539535093	0,510763081	0,270100976	-0,00637979	0,235227027	-0,392843286	0,477827514	1	-0,011456251	0,120730786	0,248144384	-0,107280345	-0,28181772	-0,1987116	0,405050286	-0,507220395	-0,576069369	-0,438134769	0,754964743	-0,186374588	0,645552872	-0,165959951	0,672089902	-0,01437677	-0,230240656
-0,247790949	-0,275981774	0,10437956	0,137786532	0,026858056	0,154440874	0,277815831	0,09495799	-0,208608096	-0,205849426	0,29269278	-0,277695115	-0,021974039	0,097329618	0,049600203	-0,011456251	1	0,170297699	-0,126225554	0,215676877	-0,06845784	0,235729897	0,404615347	-0,019020363	0,329583805	0,104603734	-0,142140583	-0,13885274	-0,091760152	-0,197511267	-0,056665073	-0,135786423	-0,225679814
-0,05129075	0,059263387	0,001720782	-0,123179312	0,299748027	0,105166437	0,007306187	-0,01280761	0,007625236	0,214592239	0,125778714	-0,275831741	0,049207769	-0,016856889	0,178727945	0,120730786	0,170297699	1	0,093370181	-0,009955874	-0,361110884	-0,04618573	0,072663023	-0,165818908	0,082047682	-0,179334696	0,243473161	-0,204027498	0,224918837	-0,298499387	0,091079699	-0,082814774	-0,077729269
0,032044927	0,006575309	0,007401162	-0,107148561	0,162515591	-0,222605572	-0,27370112	-0,09783874	0,137993907	0,194531652	0,116377942	0,157068382	0,050122138	-0,25505787	0,079644704	0,248144384	-0,126225554	0,093370181	1	-0,030729122	-0,00230014	-0,16039038	0,089732292	-0,05183149	-0,164460306	-0,225711802	0,331624243	0,198549715	0,054253105	-0,014775187	0,114488094	0,248576949	-0,166810132
-0,161163947	-0,034758921	-0,188578895	0,095797959	0,023835204	0,052576604	0,001368599	-0,086507106	-0,01372606	-0,022079552	0,135461207	-0,191077013	-0,137268518	0,139392677	0,05404086	-0,107280345	0,215676877	-0,009955874	-0,030729122	1	0,198156392	0,225389473	-0,025921054	0,134087116	0,188106354	-0,008677515	-0,141510769	0,204939884	-0,247908957	0,064457162	-0,226974843	0,196158095	-0,104867678
0,03336468	-0,004830385	0,113240811	0,202377024	-0,309772934	0,126440783	0,189606046	0,112253528	-0,261494971	-0,214763744	-0,041502224	0,273661061	-0,153997375	0,007515866	-0,341099149	-0,28181772	-0,06845784	-0,361110884	-0,00230014	0,198156392	1	-0,08894863	-0,207646786	0,316524183	0,292767465	0,231345418	-0,299226722	0,438269723	-0,326850215	0,274832599	-0,29742531	0,249861924	0,056288638
-0,021742136	-0,103875312	0,064465918	0,150515494	0,028191704	0,031179603	0,233036465	0,016611402	-0,133472522	-0,078454419	0,11404506	-0,289704621	-0,002683394	0,464715976	0,021988807	-0,198711595	0,235729897	-0,046185727	-0,160390379	0,225389473	-0,08894863	1	0,073968664	0,05073982	0,188541373	0,131019957	-0,279872654	0,003970049	-0,235550594	-0,081883209	-0,065967116	-0,018966949	0,086745826
0,00681381	-0,0026725	-0,185040013	-0,064903469	0,391851113	-0,321915072	-0,345589877	-0,35908824	0,295023386	0,320630881	0,505524644	-0,110874599	0,080313663	-0,225212853	0,323166633	0,405050286	0,404615347	0,072663023	0,089732292	-0,025921054	-0,207646786	0,073968664	1	-0,41818816	-0,252133496	-0,361583846	0,429699908	-0,063665361	0,361855678	-0,150504738	0,398661418	-0,04174272	-0,156203993
0,01256827	-0,189397048	0,272200428	0,078153429	-0,414345088	0,350084556	0,473879259	0,549442238	-0,466074452	-0,451631964	-0,390675105	0,024908652	-0,071702875	0,246124316	-0,359484484	-0,507220395	-0,019020363	-0,165818908	-0,05183149	0,134087116	0,316524183	0,05073982	-0,41818816	1	0,443390366	0,491307495	-0,449174754	0,24085439	-0,552580567	0,094562604	-0,482575531	0,011041621	0,282878291
-0,123516043	0,054455512	0,396459171	0,098492832	-0,449261162	0,341439594	0,731761459	0,49762633	-0,572170554	-0,474364296	-0,152364728	0,060013542	-0,207135396	0,254873954	-0,421123883	-0,576069369	0,329583805	0,082047682	-0,164460306	0,188106354	0,292767465	0,188541373	-0,252133496	0,443390366	1	0,450449197	-0,579858649	0,176079475	-0,621935562	0,100021718	-0,584574767	-0,028341511	0,118800903
-0,04488084	-0,200503518	0,29647062	0,074116726	-0,463382884	0,457072984	0,555027607	0,657180845	-0,473312883	-0,491726558	-0,33920722	-0,081962835	-0,01679864	0,323715225	-0,346937629	-0,438134769	0,104603734	-0,179334696	-0,225711802	-0,008677515	0,231345418	0,131019957	-0,361583846	0,491307495	0,450449197	1	-0,497891223	0,152710909	-0,457683164	0,162366566	-0,323373349	-0,096007024	0,145397156
0,128040527	0,204107117	-0,259213334	-0,150200337	0,518491413	-0,274716855	-0,733004484	-0,497003035	0,496759232	0,539576705	0,280800764	0,091466767	0,172614778	-0,480897039	0,473721866	0,754964743	-0,142140583	0,243473161	0,331624243	-0,141510769	-0,299226722	-0,27987265	0,429699908	-0,449174754	-0,579858649	-0,497891223	1	-0,085033811	0,580602463	-0,157510832	0,537418484	0,08518019	-0,194513526
0,255634161	-0,040765148	0,127163029	0,097899987	-0,122564944	0,03514521	0,00992466	0,053566537	-0,108320349	-0,029775036	0,081962141	0,324252279	-0,123548273	-0,075116029	-0,204102563	-0,186374588	-0,13885274	-0,204027498	0,198549715	0,204939884	0,438269723	0,003970049	-0,063665361	0,24085439	0,176079475	0,152710909	-0,085033811	1	-0,273923314	0,13736649	-0,187961028	0,41108681	0,079935949
0,07124554	-0,037923756	-0,347429993	-0,033993111	0,53346984	-0,199527897	-0,614969772	-0,468988426	0,52627486	0,506248696	0,323686487	-0,099562351	0,198134464	-0,33652276	0,35878645	0,645552872	-0,091760152	0,224918837	0,054253105	-0,247908957	-0,326850215	-0,23555059	0,361855678	-0,552580567	-0,621935562	-0,457683164	0,580602463	-0,273923314	1	-0,267160989	0,741682543	-0,065485181	-0,203553158
-0,125671648	0,172665504	0,027460264	-0,000758447	-0,152257522	-0,104359347	0,02166839	0,042418578	0,009222484	-0,097865063	-0,207385873	0,194641353	-0,166001542	-0,081750481	-0,218019282	-0,165959951	-0,197511267	-0,298499387	-0,014775187	0,064457162	0,274832599	-0,08188321	-0,150504738	0,094562604	0,100021718	0,162366566	-0,157510832	0,13736649	-0,267160989	1	-0,25525948	0,04574878	-0,029341336
0,265811026	-0,109504264	-0,299928384	0,095366454	0,47061955	-0,172657385	-0,545316643	-0,402217114	0,477962694	0,385800832	0,34542828	-0,127370079	0,404178661	-0,212417098	0,362841266	0,672089902	-0,056665073	0,091079699	0,114488094	-0,226974843	-0,29742531	-0,06596712	0,398661418	-0,482575531	-0,584574767	-0,323373349	0,537418484	-0,187961028	0,741682543	-0,25525948	1	-0,050020026	-0,085761118
0,145779066	0,043121972	-0,030227022	0,061649553	-0,045147648	-0,072125935	-0,182825536	-0,14993204	-0,004084362	0,076114023	0,162076862	0,216570796	-0,042096252	-0,149350895	-0,01074557	-0,01437677	-0,135786423	-0,082814774	0,248576949	0,196158095	0,249861924	-0,01896695	-0,04174272	0,011041621	-0,028341511	-0,096007024	0,08518019	0,41108681	-0,065485181	0,04574878	-0,050020026	1	-0,061751924
0,33853419	0,045394115	0,071964089	-0,07149815	-0,171759059	0,123130352	0,209512191	0,147785575	-0,18122401	-0,130328699	-0,253974292	-0,022698833	0,12091835	0,226223318	-0,202708451	-0,230240656	-0,225679814	-0,077729269	-0,166810132	-0,104867678	0,056288638	0,086745826	-0,156203993	0,282878291	0,118800903	0,145397156	-0,194513526	0,079935949	-0,203553158	-0,029341336	-0,085761118	-0,061751924	1
0,030586834	0,094488697	-0,353846791	-0,033690849	0,510989485	-0,354389188	-0,662421907	-0,547178089	0,637432156	0,532217082	0,262880512	-0,097633219	0,218177807	-0,297964864	0,377332576	0,570758944	-0,240649464	0,131101934	0,149913899	-0,175791315	-0,345571776	-0,20223173	0,30278575	-0,605199374	-0,675428443	-0,543059252	0,549582886	-0,242384901	0,761705042	-0,085169474	0,633356658	-0,032042224	-0,219900625
0,098521228	-0,017830193	-0,277716174	0,109282892	0,30511136	-0,081266243	-0,400119874	-0,453839422	0,323714872	0,247692825	0,245909379	0,041692506	0,019261011	-0,151755985	0,090417348	0,339247793	-0,094665574	-0,023899372	0,052756059	-0,008259967	0,009646945	-0,03313113	0,220862338	-0,383477481	-0,365158902	-0,208140072	0,290792339	0,047358277	0,357851304	0,312940384	0,41574081	0,066180196	-0,140415395
-0,047507525	-0,172486259	-0,288890283	0,092428575	0,430194449	-0,095576821	-0,467310128	-0,343010168	0,507964092	0,343382634	0,306805428	-0,213459193	0,013059976	-0,100316997	0,359048094	0,285487563	0,041581711	0,107441583	0,156597946	0,269564365	-0,124017806	-0,01455394	0,166711918	-0,205237388	-0,433735188	-0,276102388	0,270735297	0,011640298	0,401719572	-0,09029279	0,330472677	0,099927035	-0,283216548
-0,118092291	-0,259005965	0,365214103	0,033887428	-0,396147899	0,219990168	0,746221257	0,427546809	-0,511769886	-0,628016066	-0,192643059	-0,13710979	-0,184114653	0,339440766	-0,37287986	-0,562076768	0,309534676	-0,047770972	-0,20811087	0,054971734	0,165873612	0,174007782	-0,199682573	0,344513045	0,526261518	0,381514206	-0,591943652	0,021198569	-0,470726515	0,026500537	-0,429414407	-0,143036069	0,112482587
-0,381591286	-0,091216396	0,130486682	-0,023679421	0,011977168	0,045094037	0,282520721	0,186680041	-0,200323588	-0,190914071	0,137999707	-0,264644862	-0,05986086	0,131179814	0,05945399	-0,012265101	0,686813526	0,149365198	-0,057402302	0,147637393	-0,208954944	0,338570273	0,28463579	-0,000764973	0,343481231	0,099980745	-0,092587215	-0,205057147	-0,229567408	-0,039910591	-0,215197558	-0,181244293	-0,206726468
0,07765996	0,064534229	-0,017974549	-0,191086176	-0,126261157	-0,142791409	-0,025975495	-0,023350818	0,114171626	-0,070590014	-0,196651432	0,235938159	-0,036221559	-0,118781401	0,029783007	0,052864625	-0,335523814	-0,099769247	-0,058753255	-0,401116677	-0,073778706	-0,28108783	-0,063242849	-0,086297856	-0,169126115	-0,10878349	-0,004941117	-0,194970563	0,229988264	0,036026585	0,217916813	-0,122084017	0,145478981
0,36499779	0,25507051	-0,364332354	-0,08100762	0,398742084	-0,3684037	-0,576768951	-0,554906841	0,48216933	0,502880343	0,231849253	0,073569389	0,211097878	-0,188768026	0,271437018	0,460333902	-0,331296998	0,166519246	0,12432525	-0,217044772	-0,288316942	-0,09744179	0,274738877	-0,525558998	-0,527796109	-0,550376304	0,512132882	-0,17393691	0,610319462	-0,157378436	0,612340989	0,015159193	0,078299133
-0,034091178	0,194125877	-0,338313676	-0,080175377	0,614786814	-0,352762598	-0,743433899	-0,577496336	0,591624705	0,718612224	0,31043671	-0,072547361	0,169421782	-0,342827125	0,539556613	0,62718629	-0,188028958	0,349227275	0,3000276	-0,025812949	-0,422713566	-0,12886724	0,324993979	-0,548326134	-0,63816949	-0,585847128	0,730279437	-0,161601262	0,632207522	-0,199646455	0,480463775	0,050840594	-0,277689232
0,062924807	-0,241605522	0,042218863	0,15480279	0,00975163	0,133496545	-0,036402551	0,14727544	-0,062013126	-0,022197671	-0,108936211	-0,199638046	-0,020336372	0,160052411	-0,091620608	-0,087382115	-0,007975003	-0,023347344	-0,021530313	0,276772826	0,192063916	0,029905113	-0,207339492	0,268783172	0,015883062	0,146929513	-0,161101169	0,217923553	-0,07880671	0,090424292	-0,12061215	0,186633378	0,025409025

Supplementary Table S4A. Matrix of correlation coefficients for each comparison

ARID1A	EIF2AK2	HSF1.pS326	ADM	EIF4G1	H3K27Me3	LMNB1	SPI1	WEE1.pS642
-0,25801886	-0,038420273	-0,265746567	0,413455474	0,013176392	0,162304847	-0,096531087	-0,5190034	-0,100788992
-0,453358559	-0,288547162	-0,247756964	0,358480204	-0,008667443	0,006125073	-0,34748261	-0,423404198	0,093287052
0,662045169	0,384203524	0,421227906	-0,37772196	-0,187375342	0,156876154	0,523991111	0,615351795	0,014963377
-0,371669513	-0,370407888	-0,140821943	0,353035516	0,156050916	-0,002340138	-0,333253169	-0,366675834	0,1666038
-0,306653427	-0,21746898	-0,332353501	0,185917333	-0,043833804	0,175777509	-0,220820315	-0,453530757	-0,010338397
0,52953927	0,524046988	0,281114519	-0,45314014	-0,253123011	0,055493843	0,45075495	0,4538476	-0,015738203
0,270712131	0,107074003	0,303429615	-0,055504685	0,037054001	0,101464255	0,178594547	0,188180038	-0,089968884
-0,167239744	-0,234163072	-0,055706304	0,323975332	0,060091091	0,173196017	-0,194034558	-0,238975218	0,02475901
-0,20305101	-0,215409432	-0,33989507	0,01630227	-0,056385162	0,195801554	-0,032572808	-0,249125682	-0,138747832
0,266661593	0,019391081	-0,0405659	-0,064453846	-0,167852308	0,245645854	0,217260278	0,160800714	-0,265746616
0,181311247	0,070660613	0,296179403	-0,256253625	0,102080668	-0,310406305	0,067984076	0,272455016	0,352723664
-0,24028793	-0,113903454	-0,318607798	0,21056543	0,105631432	0,315606441	-0,080228605	-0,369123473	-0,329428095
-0,355350206	-0,153772181	-0,434569721	0,33150226	0,020921269	0,295130892	-0,13094105	-0,516136218	-0,255873923
0,664327176	0,437165885	0,381816992	-0,623342442	-0,165513475	0,070929993	0,599353057	0,664932559	-0,056867924
0,409832021	0,270366751	0,210289364	-0,139509895	0,021525495	0,060327978	0,218008386	0,338828777	0,010822465
-0,539585495	-0,443352242	-0,417151418	0,705312783	0,305836675	0,077475362	-0,51792037	-0,573024278	-0,055926192
0,25929572	0,209946688	0,524261407	-0,253874161	0,226880158	-0,310791093	0,090562334	0,353157077	0,141092427
0,580365873	0,400303173	0,505708371	-0,544624509	-0,149186066	-0,115882056	0,4863978	0,703365843	0,086231844
0,517198483	0,439738843	0,297352582	-0,436126435	-0,238432233	0,076800926	0,448169465	0,535569246	-0,07937481
-0,368620328	-0,210949848	-0,423481671	0,279786496	-0,132996569	0,317071861	-0,267838071	-0,555587849	-0,020259954
0,54395673	0,282145826	0,260599818	-0,334285838	0,113521957	0,089567366	0,407198424	0,514600374	-0,059027993
-0,206996674	-0,133352854	-0,311665338	0,168064761	0,011343017	0,10088068	-0,14224089	-0,297484101	0,034913144
-0,256587734	-0,221215725	-0,28955643	0,467289191	0,036930954	0,218289525	-0,298717355	-0,410971384	-0,070544915
0,569324343	0,395179033	0,253394774	-0,460828531	-0,035558846	0,090048229	0,449499441	0,576266278	-0,182220624
-0,302091755	-0,258851847	-0,23731774	0,166005647	-0,004702883	0,152869064	-0,218000396	-0,293873277	-0,015366317
0,394122334	0,204478548	0,341748245	-0,087453061	-0,109822546	0,055186022	0,202083624	0,324887996	0,085131004
-0,525543097	-0,27747794	-0,500975688	0,33866042	-0,054456411	0,163446486	-0,398010254	-0,622319191	-0,077203067
-0,059887353	-0,007544255	0,185568566	-0,155572257	-0,013000041	-0,299999949	-0,021218016	0,233559605	0,028051666
0,448482814	0,228243364	0,383513672	-0,434355232	-0,145554989	-0,153122064	0,327750494	0,655977102	-0,026897989
0,355621821	0,187102728	0,593497301	-0,435930736	-0,134462691	-0,226818252	0,234123958	0,520447488	0,198902911
0,591795245	0,439029931	0,236324686	-0,349948507	-0,298267031	0,250271527	0,616560712	0,395534279	-0,050872664
0,085009746	0,014280113	0,218920787	-0,17253646	0,320489122	-0,379314916	0,007668765	0,332170808	0,086350473
-0,52092029	-0,366115598	-0,31139572	0,485459649	0,138775932	0,05985967	-0,418646716	-0,511593223	0,01547215
-0,018913537	-0,128736567	-0,130597566	0,031138396	-0,096698862	0,155818086	0,163464064	-0,057148161	-0,056978189
0,12026465	0,042441511	0,230142184	-0,239366069	-0,172227233	-0,260969332	0,137346503	0,436236971	0,121586927
-0,41870961	-0,369315792	-0,386581229	0,363972549	-0,110648442	0,263763376	-0,309340847	-0,463718063	-0,077511036
0,036204977	-0,171234262	0,146066972	-0,229233146	-0,054826719	-0,179936669	-0,052621623	0,286968219	0,127436703
-0,384288005	-0,214907731	-0,159964283	0,29204928	0,091506289	-0,063550299	-0,333383668	-0,370654533	0,215816815
-0,320916047	-0,215585982	-0,195435239	0,347890223	-0,001170846	-0,030362986	-0,247775883	-0,312581528	0,112306417
-0,577196863	-0,320247845	-0,313971948	0,198266808	-0,045968407	-0,058033753	-0,435360843	-0,554830833	0,276197013
0,825789025	0,428740197	0,444455371	-0,457352704	-0,213919632	0,180580525	0,607651089	0,6503188	-0,133901644
-0,515794117	-0,410258346	-0,238414115	0,273734977	0,006842809	-0,134891788	-0,468291957	-0,469230871	0,348066889
-0,598690371	-0,41298615	-0,461589719	0,633694765	0,293644909	-0,01734625	-0,495773387	-0,674523315	-0,015900109
-0,617629076	-0,418201012	-0,306435023	0,5173996	0,073645936	-0,097730474	-0,550886631	-0,607841148	0,185148489
0,191070038	0,356999104	0,466957567	-0,169682717	-0,194416132	-0,146432075	0,119135063	0,272689435	0,03080223
-0,079489795	0,090757116	0,102387391	0,105850184	-0,046685172	-0,119895826	-0,113572994	-0,046605195	0,027456095
-0,590257996	-0,328784655	-0,377220542	0,314778393	-0,012857537	-0,033954068	-0,322846005	-0,603064214	0,174767421
0,541810954	0,251171855	0,435979463	-0,555748074	-0,05986744	-0,13697925	0,4163851	0,676474075	0,076104959
-0,263894996	-0,176941085	-0,165618147	0,205316924	0,106217162	-0,129278184	-0,120063072	-0,256914476	0,141106233
-0,595499283	-0,371507937	-0,407963522	0,654747803	0,182225239	0,00803725	-0,499283601	-0,640451689	0,099094172
0,451910205	0,177093345	0,277736053	-0,168989776	0,053631708	-0,080142548	0,154356244	0,387351237	0,330878907
-0,431631766	-0,114622555	-0,397377222	0,418673591	0,509727366	-0,046849866	-0,280470588	-0,440368898	-0,261384443
-0,226131704	-0,186861938	-0,050376864	0,138298482	0,255659643	-0,193966758	-0,220853954	-0,174465829	0,165491398
0,419404163	0,262990464	0,409331591	-0,55713608	-0,158485094	-0,113358619	0,437119602	0,589937363	-0,009429244
0,273218828	0,243883973	-0,06326346	-0,152335959	-0,32622788	0,373010732	0,403358238	0,037811816	-0,176650745
-0,048344178	-0,076589099	-0,041353009	0,119539337	-0,066168421	0,141877117	-0,06768203	-0,039561761	0,065291786
-0,337496309	-0,083807178	-0,250592999	0,022831678	-0,265108547	0,094609322	-0,088384912	-0,387015028	0,103543792
-0,410328589	-0,262526652	-0,171252143	0,414106875	0,335014861	-0,205932393	-0,48570358	-0,417718027	0,096241646
-0,507908994	-0,16391646	-0,236095852	0,454417083	0,483287181	-0,261121062	-0,473463473	-0,487813859	-0,025668674
0,166512517	0,057906164	0,092625273	-0,12223077	0,050077823	-0,074705831	0,251116351	0,309182859	-0,103272686
-0,459062456	-0,342432584	-0,258620318	0,24630578	0,031403619	-0,042222159	-0,47502066	-0,484273376	0,351658756
-0,524851373	-0,340007159	-0,280398038	0,523244021	0,317194915	-0,228503077	-0,468393312	-0,387054288	-0,114517939
-0,016547397	-0,008918545	0,059541825	0,310337442	0,257843147	-0,046166479	-0,246525083	-0,183376957	-0,014406026
-0,229841153	0,070379321	-0,128672506	0,052234265	0,193081509	-0,152039349	0,209849216	-0,14893634	-0,137561872
0,523425661	0,20894165	0,190142709	-0,22054014	-0,328930099	0,199636131	0,416515726	0,4405936	-0,110964553
0,003634959	-0,067981761	0,129705257	0,209061767	0,378510371	-0,118611797	-0,204795666	-0,067960358	0,103818692
-0,391827845	-0,209599729	-0,032496653	0,162238918	-0,022443566	-0,209222928	-0,377697998	-0,317827981	0,291486835
0,030586834	0,098521228	-0,047507525	-0,118092291	-0,381591286	0,07765996	0,36499779	-0,034091178	0,062924807
0,094488697	-0,017830193	-0,172486259	-0,259005965	-0,091216396	0,064534229	0,25507051	0,194125877	-0,241605522
-0,353846791	-0,277716174	-0,288890283	0,365214103	0,130486682	-0,017974549	-0,364332354	-0,338313676	0,042218863
-0,033690849	0,109282892	0,092428575	0,033887428	-0,023679421	-0,191086176	-0,08100762	-0,080175377	0,15480279
0,510989485	0,30511136	0,430194449	-0,396147899	0,011977168	-0,126261157	0,398742084	0,614786814	0,00975163
-0,354389188	-0,081266243	-0,095576821	0,219990168	0,045094037	-0,142791409	-0,3684037	-0,352762598	0,133496545
-0,662421907	-0,400119874	-0,467310128	0,746221257	0,282520721	-0,025975495	-0,576768951	-0,743433899	-0,036402551
-0,547178089	-0,453839422	-0,343010168	0,427546809	0,186680041	-0,023350818	-0,554906841	-0,577496336	0,14727544
0,637432156	0,323714872	0,507964092	-0,511769886	-0,200323588	0,114171626	0,48216933	0,591624705	-0,062013126
0,532217082	0,247692825	0,343382634	-0,628016066	-0,190914071	-0,070590014	0,502880343	0,718612224	-0,022197671
0,262880512	0,245909379	0,306805428	-0,192643059	0,137999707	-0,196651432	0,231849253	0,31043671	-0,108936211
-0,097633219	0,041692506	-0,213459193	-0,13710979	-0,264644862	0,235938159	0,073569389	-0,072547361	-0,199638046
0,218177807	0,019261011	0,013059976	-0,184114653	-0,05986086	-0,036221559	0,211097878	0,169421782	-0,020336372
-0,297964864	-0,151755985	-0,100316997	0,339440766	0,131179814	-0,118781401	-0,188768026	-0,342827125	0,160052411
0,377332576	0,090417348	0,359048094	-0,37287986	0,05945399	0,029783007	0,271437018	0,539556613	-0,091620608
0,570758944	0,339247793	0,285487563	-0,562076768	-0,012265101	0,052864625	0,460333902	0,62718629	-0,087382115
-0,240649464	-0,094665574	0,041581711	0,309534676	0,686813526	-0,335523814	-0,331296998	-0,188028958	-0,007975003
0,131101934	-0,023899372	0,107441583	-0,047770972	0,149365198	-0,099769247	0,166519246	0,349227275	-0,023347344
0,149913899	0,052756059	0,156597946	-0,20811087	-0,057402302	-0,058753255	0,12432525	0,3000276	-0,021530313
-0,175791315	-0,008259967	0,269564365	0,054971734	0,147637393	-0,401116677	-0,217044772	-0,025812949	0,276772826
-0,345571776	0,009646945	-0,124017806	0,165873612	-0,208954944	-0,073778706	-0,288316942	-0,422713566	0,192063916
-0,202231733	-0,033131128	-0,014553943	0,174007782	0,338570273	-0,281087827	-0,097441792	-0,128867244	0,029905113
0,30278575	0,220862338	0,166711918	-0,199682573	0,28463579	-0,063242849	0,274738877	0,324993979	-0,207339492
-0,605199374	-0,383477481	-0,205237388	0,344513045	-0,000764973	-0,086297856	-0,525558998	-0,548326134	0,268783172
-0,675428443	-0,365158902	-0,433735188	0,526261518	0,343481231	-0,169126115	-0,527796109	-0,63816949	0,015883062
-0,543059252	-0,208140072	-0,276102388	0,381514206	0,099980745	-0,10878349	-0,550376304	-0,585847128	0,146929513
0,549582886	0,290792339	0,270735297	-0,591943652	-0,092587215	-0,004941117	0,512132882	0,730279437	-0,161101169
-0,242384901	0,047358277	0,011640298	0,021198569	-0,205057147	-0,194970563	-0,17393691	-0,161601262	0,217923553
0,761705042	0,357851304	0,401719572	-0,470726515	-0,229567408	0,229988264	0,610319462	0,632207522	-0,07880671
-0,085169474	0,312940384	-0,09029279	0,026500537	-0,039910591	0,036026585	-0,157378436	-0,199646455	0,090424292
0,633356658	0,41574081	0,330472677	-0,429414407	-0,215197558	0,217916813	0,612340989	0,480463775	-0,12061215
-0,032042224	0,066180196	0,099927035	-0,143036069	-0,181244293	-0,122084017	0,015159193	0,050840594	0,186633378
-0,219900625	-0,140415395	-0,283216548	0,112482587	-0,206726468	0,145478981	0,078299133	-0,277689232	0,025409025
1	0,385931142	0,41249683	-0,439360247	-0,251383845	0,164389246	0,629380105	0,697415438	-0,060222059
0,385931142	1	0,258943673	-0,243401751	-0,195330134	-0,05227755	0,342306386	0,306327012	-0,063693264
0,41249683	0,258943673	1	-0,352615802	-0,141411569	-0,19593534	0,184600598	0,435541993	0,273620927
-0,439360247	-0,243401751	-0,352615802	1	0,297587045	-0,004444529	-0,506411059	-0,612626425	-0,04184439
-0,251383845	-0,195330134	-0,141411569	0,297587045	1	-0,299927939	-0,341936546	-0,153438444	-0,098582483
0,164389246	-0,05227755	-0,19593534	-0,004444529	-0,299927939	1	0,289155267	-0,099135966	-0,255000846
0,629380105	0,342306386	0,184600598	-0,506411059	-0,341936546	0,289155267	1	0,570454745	-0,259234854
0,697415438	0,306327012	0,435541993	-0,612626425	-0,153438444	-0,099135966	0,570454745	1	-0,030953161
-0,060222059	-0,063693264	0,273620927	-0,04184439	-0,098582483	-0,255000846	-0,259234854	-0,030953161	1

Supplementary Table S4B. Matrix of p-values for each comparison

AKR1C3	AKT3	ASH2L	ASS1	BIRC2	BRD4	CASP3	CASP3.cle	CD4	CD74	CDK1_2_3.pT14	CDKN1B	CDKN1B.pS10	CDKN1B.pT198	CHEK2	DLST	EIF4E.pS209	EIF4EBP1.pS65	EP300	ETS1	EZH2	EZR	HDAC3	HSF1	JMJD6	KIT	LCK	LYN	MAPK14	MAPK14.pT180_Y182	MEN1	MKNK1	NDUFB4	NOTCH3	PKM2
0	3,45611E-16	9,59381E-07	0,00067607	2,96096E-07	0,000617404	0,613520366	0,001542859	0,259973997	0,031264521	0,010098085	3,50133E-05	2,29528E-11	3,30271E-13	0,001710967	1,13924E-15	3,5995E-09	7,74837E-12	1,1197E-06	2,05183E-05	4,45085E-06	0,014310673	7,59365E-07	4,90269E-13	0,165503289	0,746471822	8,71466E-06	1,97094E-08	2,75724E-14	2,17921E-18	0,070136172	1,2138E-18	2,00677E-08	0,065901275	4,75403E-11
3,45611E-16	0	1,43911E-13	5,13953E-12	6,78451E-23	4,08073E-08	0,149631748	5,38076E-05	0,794270425	0,270809783	0,05589553	0,455576971	0,000145419	1,87874E-25	4,71522E-07	1,27368E-25	7,94348E-11	2,91766E-22	3,77795E-17	1,48202E-07	4,74006E-12	0,076901443	2,42399E-12	2,00472E-22	0,000678125	0,037914129	3,11971E-09	0,991784722	8,91917E-13	3,42129E-08	3,51739E-09	5,07321E-05	1,7656E-17	0,240255006	0,01110588
9,59381E-07	1,43911E-13	0	4,49647E-10	1,17965E-13	3,15829E-27	2,64888E-08	0,029825852	6,73911E-11	0,001712091	1,49059E-05	4,46558E-05	1,40632E-12	7,77061E-39	3,80165E-15	7,22613E-25	4,82823E-06	3,53904E-30	2,1148E-23	3,59139E-18	1,59443E-24	7,58138E-14	0,000492868	1,09254E-37	1,0409E-07	1,30062E-23	2,10724E-37	0,580633874	4,29257E-16	3,69633E-12	1,32955E-36	0,441942396	3,55805E-18	0,111595565	0,016674506
0,00067607	5,13953E-12	4,49647E-10	0	0,000100386	9,74215E-19	0,056359917	7,93244E-09	0,716522395	0,174530771	0,479321577	0,192136195	0,005299536	1,95991E-22	0,002498737	2,73863E-29	4,187E-08	3,02332E-16	3,74169E-15	8,67457E-05	2,7533E-06	0,014340227	8,26134E-12	7,54436E-15	0,000603031	0,115514426	2,04443E-05	0,043770714	1,15653E-12	4,3915E-06	2,82385E-08	0,015737408	2,28069E-35	0,23670402	0,00046288
2,96096E-07	6,78451E-23	1,17965E-13	0,000100386	0	0,066781483	0,040340886	0,001679986	0,000805874	0,122128483	0,000678383	1,0171E-06	4,21519E-12	4,49399E-13	0,034680992	4,04897E-11	2,79898E-13	3,89623E-16	9,88344E-07	1,54142E-18	7,91919E-08	4,11489E-05	1,02573E-11	3,01305E-08	3,12988E-08	6,3177E-05	6,58681E-12	5,77313E-06	5,69567E-19	5,48096E-10	0,012627982	1,1871E-09	3,57112E-10	0,318478019	2,98615E-16
0,000617404	4,08073E-08	3,15829E-27	9,74215E-19	0,066781483	0	0,584568177	2,2907E-07	8,71211E-05	0,026296904	3,4982E-10	6,30172E-05	1,20345E-05	3,50599E-49	3,9833E-19	1,46459E-35	3,82185E-08	4,30515E-23	3,76029E-43	8,31252E-11	6,93569E-25	1,765E-07	9,83588E-06	8,64671E-42	6,58467E-11	3,5366E-06	1,15095E-19	0,084016666	0,000119275	0,003856942	1,98396E-38	0,519905524	2,52759E-20	0,754237698	0,037045318
0,613520366	0,149631748	2,64888E-08	0,056359917	0,040340886	0,584568177	0	1,49025E-08	7,09346E-13	0,531657806	0,878193351	0,025460038	0,001608388	1,27473E-06	0,018348145	0,002139646	5,23365E-08	3,21811E-05	0,107396345	8,55445E-09	3,53278E-07	8,47697E-06	0,300554702	0,025176965	0,452269425	1,43146E-05	6,06607E-12	0,083542706	2,74333E-06	0,000869406	0,044291158	0,030302939	2,51815E-05	0,624052779	0,110458319
0,001542859	5,38076E-05	0,029825852	7,93244E-09	0,001679986	2,2907E-07	1,49025E-08	0	0,005576953	0,360954343	0,014290498	0,385246518	0,882312238	2,36693E-08	0,591899192	7,36147E-25	4,75283E-06	1,37233E-11	2,80352E-05	0,652040027	0,023306542	0,306713328	3,18756E-15	1,45341E-08	0,006569709	0,026287168	0,771920813	0,000208464	2,20719E-07	0,004374441	0,000181164	0,017198952	7,08936E-10	0,034812927	4,80169E-07
0,259973997	0,794270425	6,73911E-11	0,716522395	0,000805874	8,71211E-05	7,09346E-13	0,005576953	0	0,006572369	0,000151911	2,55354E-32	1,5869E-31	9,31926E-05	8,32948E-12	0,817504895	0,023731006	0,001088912	0,000466077	8,67181E-52	6,71314E-12	9,09763E-23	0,024546355	4,11907E-05	1,97553E-05	1,28613E-18	3,34048E-36	0,471016613	0,000268227	0,234176856	0,017606338	0,339175816	0,756996368	0,24666974	0,284403901
0,031264521	0,270809783	0,001712091	0,174530771	0,122128483	0,026296904	0,531657806	0,360954343	0,006572369	0	0,000137397	0,003339531	0,101869878	0,012173263	0,611691812	0,422777675	0,127033799	0,372261556	0,34670851	0,001653868	0,0442819	0,333129964	0,9158733	0,001575265	0,004155652	0,949767433	0,664814334	0,017304236	0,746906535	0,837481441	0,000534904	0,109773004	0,568545811	0,010485529	0,104479771
0,010098085	0,05589553	1,49059E-05	0,479321577	0,000678383	3,4982E-10	0,878193351	0,014290498	0,000151911	0,000137397	0	8,7451E-11	2,83256E-09	4,59734E-07	0,008572728	3,57992E-06	8,03644E-11	9,30434E-13	0,000241633	9,96962E-15	2,39709E-16	0,000975668	6,8417E-05	0,008054655	5,45133E-06	0,042119024	1,06061E-19	0,010862007	0,039660673	2,56751E-05	0,015204434	0,000274963	0,001859691	0,242797433	0,075318374
3,50133E-05	0,455576971	4,46558E-05	0,192136195	1,0171E-06	6,30172E-05	0,025460038	0,385246518	2,55354E-32	0,003339531	8,7451E-11	0	4,4952E-95	2,32598E-06	2,34858E-14	0,02283069	0,002760608	4,9712E-05	0,000775168	3,94671E-37	1,70573E-12	7,68575E-19	0,460831553	0,010375826	1,30266E-05	1,25433E-12	4,88913E-22	1,59355E-06	1,06297E-05	0,000160025	0,025024148	5,0045E-08	0,120773385	0,22197671	0,001249056
2,29528E-11	0,000145419	1,40632E-12	0,005299536	4,21519E-12	1,20345E-05	0,001608388	0,882312238	1,5869E-31	0,101869878	2,83256E-09	4,4952E-95	0	2,20795E-12	2,04545E-12	7,32154E-07	1,59484E-07	5,19027E-12	5,96897E-05	2,25071E-47	6,00888E-15	2,14014E-18	0,001659689	2,76384E-07	2,53545E-06	8,01393E-14	4,85508E-33	2,96156E-06	1,00689E-10	2,22199E-09	0,009138578	3,37183E-10	7,08076E-05	0,476153291	4,43467E-05
3,30271E-13	1,87874E-25	7,77061E-39	1,95991E-22	4,49399E-13	3,50599E-49	1,27473E-06	2,36693E-08	9,31926E-05	0,012173263	4,59734E-07	2,32598E-06	2,20795E-12	0	2,75226E-19	3,85468E-68	7,0239E-12	1,97311E-60	6,95028E-32	7,00033E-17	1,20987E-28	0,00024048	2,37216E-19	1,65929E-67	3,16413E-09	3,65427E-06	7,54822E-30	0,313774446	3,92181E-35	5,73754E-17	2,2062E-41	0,010119313	7,35778E-48	0,185015974	0,002704095
0,001710967	4,71522E-07	3,80165E-15	0,002498737	0,034680992	3,9833E-19	0,018348145	0,591899192	8,32948E-12	0,611691812	0,008572728	2,34858E-14	2,04545E-12	2,75226E-19	0	0,000824356	0,178019187	4,4854E-05	2,36701E-13	3,63654E-14	3,88494E-20	7,77586E-19	0,940923569	9,27652E-33	7,46992E-05	9,78756E-17	4,34761E-19	0,235367213	0,003672591	0,247792302	9,14307E-20	0,006564189	0,016271039	0,250408845	1,82237E-06
1,13924E-15	1,27368E-25	7,22613E-25	2,73863E-29	4,04897E-11	1,46459E-35	0,002139646	7,36147E-25	0,817504895	0,422777675	3,57992E-06	0,02283069	7,32154E-07	3,85468E-68	0,000824356	0	3,77177E-21	6,4323E-55	4,10503E-28	2,98025E-07	3,00856E-11	0,060822128	8,75692E-41	1,98705E-29	6,76836E-11	0,003499779	4,50818E-17	0,00168997	2,59667E-30	1,78832E-21	2,51151E-25	5,53361E-05	1,38987E-57	0,003364072	5,37075E-10
3,5995E-09	7,94348E-11	4,82823E-06	4,187E-08	2,79898E-13	3,82185E-08	5,23365E-08	4,75283E-06	0,023731006	0,127033799	8,03644E-11	0,002760608	1,59484E-07	7,0239E-12	0,178019187	3,77177E-21	0	2,88351E-30	2,81107E-06	1,64505E-15	4,26774E-15	0,0291387	5,94107E-22	0,001231741	0,000171321	0,021117969	2,36291E-15	8,82946E-07	1,68165E-11	4,98695E-26	0,053434976	2,25259E-28	1,10786E-15	0,000272879	1,78882E-06
7,74837E-12	2,91766E-22	3,53904E-30	3,02332E-16	3,89623E-16	4,30515E-23	3,21811E-05	1,37233E-11	0,001088912	0,372261556	9,30434E-13	4,9712E-05	5,19027E-12	1,97311E-60	4,4854E-05	6,4323E-55	2,88351E-30	0	2,54241E-22	1,64685E-15	2,80206E-20	0,004972443	3,60063E-30	5,33997E-26	4,22323E-09	0,000447696	4,06685E-28	0,06630808	2,23503E-34	3,85978E-35	2,24769E-18	2,16993E-06	6,47031E-37	0,088243199	4,79836E-16
1,1197E-06	3,77795E-17	2,1148E-23	3,74169E-15	9,88344E-07	3,76029E-43	0,107396345	2,80352E-05	0,000466077	0,34670851	0,000241633	0,000775168	5,96897E-05	6,95028E-32	2,36701E-13	4,10503E-28	2,81107E-06	2,54241E-22	0	3,95268E-10	2,62898E-12	0,008180778	5,82281E-07	3,4086E-33	1,75279E-08	0,002242463	1,91357E-13	0,287338286	9,76627E-11	3,58769E-06	1,48671E-24	0,00174002	4,59831E-19	0,025481589	0,000574936
2,05183E-05	1,48202E-07	3,59139E-18	8,67457E-05	1,54142E-18	8,31252E-11	8,55445E-09	0,652040027	8,67181E-52	0,001653868	9,96962E-15	3,94671E-37	2,25071E-47	7,00033E-17	3,63654E-14	2,98025E-07	1,64505E-15	1,64685E-15	3,95268E-10	0	1,51921E-31	1,6129E-32	0,015427919	3,91565E-12	8,13558E-12	6,546E-14	1,07258E-67	0,000212769	8,40448E-14	1,47263E-06	0,005652871	3,51526E-11	1,80149E-06	0,865990013	0,011561672
4,45085E-06	4,74006E-12	1,59443E-24	2,7533E-06	7,91919E-08	6,93569E-25	3,53278E-07	0,023306542	6,71314E-12	0,0442819	2,39709E-16	1,70573E-12	6,00888E-15	1,20987E-28	3,88494E-20	3,00856E-11	4,26774E-15	2,80206E-20	2,62898E-12	1,51921E-31	0	1,63413E-09	0,007385693	1,24616E-21	1,51434E-07	6,24067E-13	1,11993E-39	0,072570439	7,31489E-08	0,002845108	6,66234E-13	4,84622E-06	1,1615E-07	0,278963154	0,254515863
0,014310673	0,076901443	7,58138E-14	0,014340227	4,11489E-05	1,765E-07	8,47697E-06	0,306713328	9,09763E-23	0,333129964	0,000975668	7,68575E-19	2,14014E-18	0,00024048	7,77586E-19	0,060822128	0,0291387	0,004972443	0,008180778	1,6129E-32	1,63413E-09	0	0,327419669	2,25225E-09	0,000676272	1,27879E-15	3,82559E-20	0,248691817	0,092374257	0,013541318	0,012419884	0,086639269	0,209068463	0,007373686	0,258974961
7,59365E-07	2,42399E-12	0,000492868	8,26134E-12	1,02573E-11	9,83588E-06	0,300554702	3,18756E-15	0,024546355	0,9158733	6,8417E-05	0,460831553	0,001659689	2,37216E-19	0,940923569	8,75692E-41	5,94107E-22	3,60063E-30	5,82281E-07	0,015427919	0,007385693	0,327419669	0	1,99563E-06	0,037007111	0,692473297	9,23094E-05	7,88813E-06	1,98351E-20	1,88822E-20	0,000512961	8,28832E-09	7,34229E-33	0,000243333	1,25342E-14
4,90269E-13	2,00472E-22	1,09254E-37	7,54436E-15	3,01305E-08	8,64671E-42	0,025176965	1,45341E-08	4,11907E-05	0,001575265	0,008054655	0,010375826	2,76384E-07	1,65929E-67	9,27652E-33	1,98705E-29	0,001231741	5,33997E-26	3,4086E-33	3,91565E-12	1,24616E-21	2,25225E-09	1,99563E-06	0	3,78278E-08	2,24388E-06	1,69557E-21	0,92869053	3,02626E-16	2,60693E-07	1,21722E-29	0,156554657	4,15002E-19	0,144272016	0,604657523
0,165503289	0,000678125	1,0409E-07	0,000603031	3,12988E-08	6,58467E-11	0,452269425	0,006569709	1,97553E-05	0,004155652	5,45133E-06	1,30266E-05	2,53545E-06	3,16413E-09	7,46992E-05	6,76836E-11	0,000171321	4,22323E-09	1,75279E-08	8,13558E-12	1,51434E-07	0,000676272	0,037007111	3,78278E-08	0	0,000283629	2,77164E-30	0,500518812	1,24713E-10	0,039667089	1,46247E-09	0,010865098	1,49061E-08	0,242199529	0,021625779
0,746471822	0,037914129	1,30062E-23	0,115514426	6,3177E-05	3,5366E-06	1,43146E-05	0,026287168	1,28613E-18	0,949767433	0,042119024	1,25433E-12	8,01393E-14	3,65427E-06	9,78756E-17	0,003499779	0,021117969	0,000447696	0,002242463	6,546E-14	6,24067E-13	1,27879E-15	0,692473297	2,24388E-06	0,000283629	0	2,58486E-17	0,273902631	1,81544E-06	0,036746547	1,08484E-10	0,153179185	0,012162242	0,240165599	0,761695033
8,71466E-06	3,11971E-09	2,10724E-37	2,04443E-05	6,58681E-12	1,15095E-19	6,06607E-12	0,771920813	3,34048E-36	0,664814334	1,06061E-19	4,88913E-22	4,85508E-33	7,54822E-30	4,34761E-19	4,50818E-17	2,36291E-15	4,06685E-28	1,91357E-13	1,07258E-67	1,11993E-39	3,82559E-20	9,23094E-05	1,69557E-21	2,77164E-30	2,58486E-17	0	0,135133133	2,13341E-18	2,75187E-11	2,22811E-16	1,19268E-07	3,01868E-11	0,590024814	0,006875365
1,97094E-08	0,991784722	0,580633874	0,043770714	5,77313E-06	0,084016666	0,083542706	0,000208464	0,471016613	0,017304236	0,010862007	1,59355E-06	2,96156E-06	0,313774446	0,235367213	0,00168997	8,82946E-07	0,06630808	0,287338286	0,000212769	0,072570439	0,248691817	7,88813E-06	0,92869053	0,500518812	0,273902631	0,135133133	0	1,50172E-12	1,10445E-10	0,000695474	1,90483E-12	0,009971294	0,010278496	3,81718E-16
2,75724E-14	8,91917E-13	4,29257E-16	1,15653E-12	5,69567E-19	0,000119275	2,74333E-06	2,20719E-07	0,000268227	0,746906535	0,039660673	1,06297E-05	1,00689E-10	3,92181E-35	0,003672591	2,59667E-30	1,68165E-11	2,23503E-34	9,76627E-11	8,40448E-14	7,31489E-08	0,092374257	1,98351E-20	3,02626E-16	1,24713E-10	1,81544E-06	2,13341E-18	1,50172E-12	0	1,56383E-32	1,44263E-06	8,21372E-10	4,25147E-28	0,002713298	6,8045E-30
2,17921E-18	3,42129E-08	3,69633E-12	4,3915E-06	5,48096E-10	0,003856942	0,000869406	0,004374441	0,234176856	0,837481441	2,56751E-05	0,000160025	2,22199E-09	5,73754E-17	0,247792302	1,78832E-21	4,98695E-26	3,85978E-35	3,58769E-06	1,47263E-06	0,002845108	0,013541318	1,88822E-20	2,60693E-07	0,039667089	0,036746547	2,75187E-11	1,10445E-10	1,56383E-32	0	0,000216027	1,70216E-12	8,98961E-17	0,073626555	3,81593E-20
0,070136172	3,51739E-09	1,32955E-36	2,82385E-08	0,012627982	1,98396E-38	0,044291158	0,000181164	0,017606338	0,000534904	0,015204434	0,025024148	0,009138578	2,2062E-41	9,14307E-20	2,51151E-25	0,053434976	2,24769E-18	1,48671E-24	0,005652871	6,66234E-13	0,012419884	0,000512961	1,21722E-29	1,46247E-09	1,08484E-10	2,22811E-16	0,000695474	1,44263E-06	0,000216027	0	0,341895708	9,79779E-16	0,003678618	0,393885943
1,2138E-18	5,07321E-05	0,441942396	0,015737408	1,1871E-09	0,519905524	0,030302939	0,017198952	0,339175816	0,109773004	0,000274963	5,0045E-08	3,37183E-10	0,010119313	0,006564189	5,53361E-05	2,25259E-28	2,16993E-06	0,00174002	3,51526E-11	4,84622E-06	0,086639269	8,28832E-09	0,156554657	0,010865098	0,153179185	1,19268E-07	1,90483E-12	8,21372E-10	1,70216E-12	0,341895708	0	1,47642E-05	0,000831645	1,86724E-11
2,00677E-08	1,7656E-17	3,55805E-18	2,28069E-35	3,57112E-10	2,52759E-20	2,51815E-05	7,08936E-10	0,756996368	0,568545811	0,001859691	0,120773385	7,08076E-05	7,35778E-48	0,016271039	1,38987E-57	1,10786E-15	6,47031E-37	4,59831E-19	1,80149E-06	1,1615E-07	0,209068463	7,34229E-33	4,15002E-19	1,49061E-08	0,012162242	3,01868E-11	0,009971294	4,25147E-28	8,98961E-17	9,79779E-16	1,47642E-05	0	3,87594E-06	6,93985E-10
0,065901275	0,240255006	0,111595565	0,23670402	0,318478019	0,754237698	0,624052779	0,034812927	0,24666974	0,010485529	0,242797433	0,22197671	0,476153291	0,185015974	0,250408845	0,003364072	0,000272879	0,088243199	0,025481589	0,865990013	0,278963154	0,007373686	0,000243333	0,144272016	0,242199529	0,240165599	0,590024814	0,010278496	0,002713298	0,073626555	0,003678618	0,000831645	3,87594E-06	0	0,00379674
4,75403E-11	0,01110588	0,016674506	0,00046288	2,98615E-16	0,037045318	0,110458319	4,80169E-07	0,284403901	0,104479771	0,075318374	0,001249056	4,43467E-05	0,002704095	1,82237E-06	5,37075E-10	1,78882E-06	4,79836E-16	0,000574936	0,011561672	0,254515863	0,258974961	1,25342E-14	0,604657523	0,021625779	0,761695033	0,006875365	3,81718E-16	6,8045E-30	3,81593E-20	0,393885943	1,86724E-11	6,93985E-10	0,00379674	0
1,94508E-08	1,88625E-21	1,70422E-09	1,01993E-10	4,41206E-31	4,35489E-13	0,018635935	1,23804E-14	0,016552226	2,06218E-05	1,14201E-12	1,38128E-05	1,26234E-10	4,52451E-25	5,68153E-08	8,03977E-30	6,3087E-27	8,10537E-27	1,00386E-09	1,01749E-21	2,07767E-14	0,000498984	3,69172E-24	3,67866E-12	9,12497E-14	1,31815E-05	6,56429E-19	0,000536944	8,50427E-17	8,38204E-12	5,04916E-07	8,83784E-12	1,3441E-21	0,000618883	2,15761E-07
1,4551E-13	0,075453808	0,628604084	0,165567486	0,013109653	0,03755205	0,072954908	0,382478479	0,623484585	0,435753997	2,78491E-05	0,001383291	2,54935E-05	0,379277803	0,582520324	0,073998644	0,113073976	1,78896E-08	0,089995047	0,007754587	0,837513439	0,183916237	0,01317212	0,445829337	0,714216623	0,358571712	0,065769505	0,178547376	0,000820804	1,1734E-07	0,000585989	9,96828E-05	0,620296558	0,953200515	2,02989E-09
3,78223E-09	4,83587E-12	1,48593E-12	3,86592E-18	3,00104E-05	1,75621E-13	0,003958754	2,79654E-06	0,104802095	4,18817E-05	0,712614141	0,007962016	0,00022099	3,00882E-16	8,63387E-08	2,93823E-17	9,32018E-08	5,75854E-08	4,59105E-15	4,83536E-07	2,2367E-10	6,48196E-10	0,00093372	8,63898E-18	0,285674294	3,97523E-05	1,21478E-06	0,005388086	8,01501E-06	0,000136389	2,88748E-09	4,30389E-05	1,04718E-22	0,069863048	0,107172456
9,58959E-08	1,19853E-11	4,27496E-09	6,71489E-24	0,000830878	1,50275E-14	0,009272328	2,3311E-06	0,707595881	0,683655703	0,892514652	0,182627582	0,001028359	9,87875E-22	0,00078455	6,47947E-29	9,79125E-11	2,17692E-12	7,68707E-12	0,000563828	2,9793E-06	0,002490342	2,3484E-14	4,46777E-14	0,009791898	0,130698333	4,90118E-05	0,000792697	2,19146E-09	0,000236008	3,26903E-06	0,000115524	3,71053E-46	0,000290115	0,009718441
3,87431E-12	1,22733E-29	1,56588E-36	1,05633E-11	1,75552E-27	1,24272E-14	4,27688E-05	0,000926666	3,55324E-06	2,27249E-05	0,058385479	0,025019285	2,62331E-08	2,72951E-24	1,02276E-14	1,2949E-11	1,92569E-08	2,08071E-16	3,5514E-26	5,51525E-17	9,38054E-22	9,09977E-10	0,010849139	2,95395E-27	4,97018E-08	6,50808E-10	3,57576E-21	0,504094869	4,75525E-11	0,000589325	1,23078E-19	2,59354E-05	3,63976E-14	0,22472301	0,001097508
2,7535E-06	8,34438E-23	7,50203E-62	8,44787E-19	4,56911E-10	5,56321E-51	6,7936E-12	0,003077954	4,98988E-14	0,000256697	0,000579592	8,41036E-08	3,30753E-15	6,29816E-67	4,07609E-32	4,47085E-33	4,49293E-06	8,80233E-33	4,53651E-38	3,71131E-23	7,33972E-36	9,0329E-16	0,000862998	1,01826E-66	1,8311E-08	2,38449E-24	6,03268E-44	0,041555031	2,07109E-17	6,50401E-09	5,64793E-43	0,639611394	1,65074E-22	0,406015053	0,572232564
9,61522E-10	1,10933E-24	1,60686E-26	1,69098E-14	5,33972E-15	1,59701E-19	0,000711311	3,65831E-05	0,002552424	4,23118E-05	0,591803708	0,334100275	0,000418436	3,47226E-24	5,10711E-12	8,80139E-16	0,000352054	5,80685E-13	1,37658E-33	5,32226E-09	3,77011E-16	9,30046E-10	0,010103176	6,56536E-32	2,21409E-08	1,54473E-07	5,62777E-14	0,654264433	3,85559E-07	0,019161179	9,21096E-28	0,035559787	1,74931E-15	0,492554295	0,168634006
8,73503E-18	9,98715E-22	3,27471E-32	2,02166E-26	1,62833E-15	1,14526E-26	0,004380745	1,25493E-19	0,956036259	0,010017422	9,52856E-07	0,383586886	3,41902E-05	1,17365E-52	0,005637839	2,18279E-80	3,44071E-25	5,13146E-63	1,3676E-30	8,59539E-06	1,65501E-11	0,232644978	1,69119E-35	1,18948E-23	2,0702E-06	6,41622E-05	1,65658E-13	0,000229813	8,24338E-39	2,94908E-31	1,53052E-21	3,86008E-05	7,25664E-51	0,012804523	3,72747E-18
1,38482E-09	1,37137E-25	4,38719E-31	1,02485E-15	6,4517E-07	4,81221E-43	0,096459394	2,84335E-08	0,016933781	0,004085491	3,08738E-07	0,029346218	1,01024E-05	7,77942E-56	2,26159E-14	8,28316E-37	2,90552E-06	8,23671E-38	9,6864E-49	1,95205E-12	7,96815E-25	0,00074853	2,3934E-12	1,54697E-51	3,40534E-14	9,76423E-06	5,81324E-25	0,010656235	2,41702E-13	8,48617E-08	7,90839E-31	0,066956987	6,04878E-26	0,042879084	0,056284162
0,144774628	0,001421334	1,67818E-07	2,12423E-06	4,0914E-09	2,83444E-06	0,000673897	0,000613229	2,21963E-10	0,481326483	0,778537763	1,72405E-08	3,51351E-09	5,16258E-07	0,031826641	3,96816E-11	7,20162E-09	4,76209E-15	1,33267E-16	1,76873E-09	0,380811615	1,93534E-10	1,20216E-05	8,144E-05	0,001130923	8,27694E-07	8,75417E-05	1,00806E-09	3,50546E-10	3,57504E-11	0,000595407	0,066698227	4,4824E-10	0,37404546	7,89872E-08
0,440121501	0,44730023	0,632044378	0,950574817	0,331490295	0,602532734	0,567147624	0,789514927	0,036947325	0,037147499	0,003216123	0,040982988	0,262387228	0,119807095	0,363127437	0,605177039	0,922898834	0,725245402	0,313654817	0,793135339	0,165505254	0,103639111	0,249820234	0,518881772	0,445755785	0,089676465	0,24913301	0,000693931	0,175218612	0,658821864	0,831770375	0,146625351	0,9108925	0,137183244	0,364258784
2,37806E-22	3,72264E-33	3,67478E-30	4,73221E-14	4,00291E-24	7,95607E-15	0,001208009	7,96416E-05	0,001088348	1,72394E-05	0,004931925	0,005228067	4,07382E-12	8,44386E-28	4,26474E-12	8,68948E-18	1,25349E-14	7,23604E-21	4,6544E-23	3,01786E-13	6,33903E-21	2,05718E-07	3,42465E-06	1,12883E-31	4,58988E-05	5,58202E-09	4,43842E-21	0,062744191	3,27304E-15	1,26023E-13	2,55481E-12	5,54729E-08	7,28316E-24	0,019325483	1,88985E-05
6,01614E-36	1,13737E-22	5,75289E-23	6,61706E-11	3,79036E-18	1,10971E-14	0,000680414	4,17447E-08	0,00500198	0,932500982	1,14171E-09	9,52034E-07	3,36417E-15	7,42856E-46	6,00578E-09	2,68664E-41	1,27312E-20	1,55596E-31	4,64411E-17	2,87941E-25	9,52446E-19	7,30474E-06	1,04903E-19	1,05401E-23	2,54505E-13	3,41426E-05	7,44457E-32	7,69767E-05	7,54921E-36	6,13498E-22	9,83291E-15	2,00379E-14	1,64377E-27	0,069425643	1,60911E-13
9,72902E-06	0,000714011	5,86396E-05	0,002540766	0,347223815	0,000385823	0,000918055	0,038526735	0,898643927	0,00050503	0,135021013	0,086304741	0,707980342	1,07526E-05	0,493559557	2,83486E-08	0,000824167	0,000815967	2,55899E-10	0,643962849	0,020295356	0,337227399	0,005658212	3,20503E-05	0,045284743	0,028961235	0,045941574	0,031673308	1,72426E-08	0,000543777	0,217749239	0,371299089	5,4879E-08	4,62911E-06	0,005912217
4,63874E-25	3,57443E-23	5,28081E-27	4,91469E-28	1,61601E-07	5,61397E-34	6,9661E-06	8,45929E-16	0,303850813	0,001839261	0,000157613	0,049049926	3,92873E-07	7,62422E-65	3,40663E-08	3,73506E-91	2,59242E-23	8,90012E-46	2,81092E-27	1,28269E-07	4,96619E-18	0,0137287	5,83092E-29	9,79699E-34	2,06442E-05	5,13164E-05	9,1443E-19	9,70809E-05	6,89634E-33	8,00394E-24	5,84325E-27	2,44269E-07	3,4398E-55	0,001938303	3,78353E-07
1,09071E-05	1,65253E-07	2,10182E-19	0,005022579	0,000272442	2,94035E-14	0,122452391	0,006737306	7,74193E-15	0,173692478	1,00869E-18	4,74662E-20	3,91125E-18	3,55562E-17	2,8693E-29	1,53245E-06	0,000147028	1,66813E-13	4,94859E-08	2,61268E-10	6,85733E-28	2,46869E-06	0,29735884	1,09401E-18	2,66698E-06	8,01376E-17	6,45866E-27	0,000107271	0,000116124	0,038179755	4,48188E-15	0,003670084	8,11074E-05	0,109974753	0,710687463
9,04423E-10	0,018736354	5,56635E-12	0,131117675	0,003267499	5,47904E-05	0,101061397	0,087286354	0,134573267	0,66783463	1,59206E-06	4,98614E-17	3,50505E-13	2,29026E-10	0,010761304	1,77635E-12	0,067541746	1,05957E-13	2,01581E-09	0,006064464	1,05353E-05	0,033915353	0,001988546	0,004621466	0,005288015	2,93433E-05	8,14071E-08	0,002341887	1,9353E-11	2,38082E-16	1,59719E-09	0,006311218	1,14314E-06	0,245748015	7,77374E-13
0,136824462	0,040905751	0,224977195	0,000172407	0,130779765	7,12749E-05	0,034144205	0,886333001	0,002515238	0,187790839	4,74662E-12	0,001410432	0,004184652	1,33414E-05	1,05246E-06	0,010668007	0,269168001	0,453422254	4,65582E-07	0,011902638	0,168488638	0,142198927	0,757749578	1,61569E-06	0,072249913	0,002677146	0,045852649	0,001244101	0,005412651	0,335592061	0,000111678	0,88204997	0,003612266	9,25678E-05	0,943604843
1,94973E-15	2,82794E-14	1,08214E-10	2,74394E-11	3,4933E-13	2,08837E-18	0,198885855	4,78611E-06	0,060306164	0,134517472	0,000268005	2,52235E-08	5,00405E-16	5,36806E-37	6,33979E-09	1,23127E-35	4,04112E-15	3,53038E-33	2,94072E-14	2,05179E-23	1,00955E-12	2,01642E-06	5,04231E-18	3,19061E-21	4,9634E-07	0,058148994	3,97529E-19	3,92376E-05	9,18844E-24	2,23573E-19	2,55696E-06	2,18285E-07	2,11905E-19	0,311293209	1,14885E-07
0,019525317	0,001195027	7,27756E-05	0,000224932	0,132673752	2,38919E-23	0,273999795	0,031810961	0,123713505	0,000309037	0,01969155	0,174447326	0,23628192	2,50635E-07	1,00866E-08	0,000157204	0,000207231	0,878949275	1,72466E-06	0,011851003	0,008431171	0,068452863	0,363213192	1,03809E-07	0,067273968	1,01996E-06	0,952563076	1,08095E-09	0,037279145	6,64625E-07	1,90426E-18	2,10168E-15	0,006075827	0,007196874	1,65586E-08
0,109635732	0,051031638	0,413039559	0,022663533	0,09575806	0,047642846	0,497820576	0,004027145	0,03609148	0,919501403	0,367720464	0,409668983	0,929368064	0,090392765	0,694439998	7,00226E-05	0,000171731	0,166192054	0,322438887	0,612999089	0,509512951	0,831367794	1,79686E-05	0,166288325	0,153595204	0,216179447	0,739368625	0,000424424	0,013321082	0,022738062	0,23978727	0,046476616	0,000228599	0,46329415	0,691326145
0,000280376	0,000128887	9,53014E-14	0,164133541	1,52414E-07	0,008010222	1,67028E-05	0,102048734	1,31439E-16	0,077029359	3,3421E-05	4,74255E-07	1,62264E-12	7,15893E-05	5,35137E-07	0,439899162	0,001934203	0,0011513	5,00439E-06	7,85047E-27	1,22853E-20	2,2398E-09	0,00324525	1,25779E-08	3,41732E-05	7,50689E-11	3,05984E-23	0,520502074	2,02875E-05	0,003752172	0,661792122	4,0213E-06	0,24932924	0,382416001	0,45027277
9,03001E-08	3,93777E-07	4,99646E-14	5,66436E-14	3,37557E-06	1,37495E-10	0,036562416	0,033292371	0,119176881	0,000147443	0,000495351	3,66227E-06	1,10501E-07	9,17919E-22	3,79683E-10	1,26459E-15	0,051518807	8,34786E-07	5,77092E-12	2,15661E-06	0,000228809	0,000201995	0,000129414	3,16829E-14	0,033958489	0,000685579	2,26231E-05	1,95313E-06	2,82214E-13	5,01258E-06	6,38811E-20	0,031174076	3,64702E-12	0,598743483	0,008650674
1,58765E-09	2,88182E-06	1,58286E-20	6,34528E-10	0,126650261	3,75524E-15	0,0838069	0,00330184	0,056103785	3,93069E-11	0,919577111	0,35927671	0,015186917	1,02987E-18	0,002003862	2,61432E-27	0,002705356	3,83807E-16	2,90902E-13	0,502051587	0,00030187	0,207427811	2,028E-10	4,13444E-09	0,028136162	0,003979821	1,15008E-06	0,05827291	8,01245E-18	3,74684E-11	1,05651E-20	0,671268329	3,49471E-21	0,590965488	6,33908E-07
0,108145279	0,236573601	0,038314015	0,016982863	0,013211747	0,040522691	0,661847078	0,130421578	0,995896198	0,392531064	0,006344197	0,116711026	0,002288067	0,001310527	0,024848217	0,005359572	0,012548021	1,48033E-05	3,81369E-05	3,25039E-07	0,000265417	0,136722837	0,009792031	0,003764522	0,000376004	0,176218536	1,94126E-05	0,101263661	8,29977E-05	0,054747479	0,139790934	1,45544E-05	0,006183335	0,074862635	0,00089401
4,90249E-06	2,97942E-14	7,40651E-15	8,38538E-11	8,55103E-24	3,43241E-10	4,11085E-08	0,001525304	0,003932352	0,003550884	0,353963311	0,216979173	0,00020077	1,91552E-25	0,008122733	3,71543E-17	2,98449E-09	3,03525E-11	5,12627E-17	7,68215E-16	2,26658E-13	0,000696224	4,03175E-07	4,17906E-16	8,23871E-08	0,000507123	4,21705E-14	0,003869912	1,54621E-15	4,1073E-05	2,12851E-10	0,000142647	1,84001E-16	0,240495543	3,26405E-06
0,000153797	1,26266E-11	6,76468E-26	5,45572E-07	0,072293108	3,21257E-29	0,054128793	9,45694E-07	0,924818859	0,017972874	0,000222179	0,467892027	0,012816421	4,79493E-38	5,4704E-10	1,11567E-31	0,000368946	1,63693E-28	4,35522E-14	0,235377386	3,35377E-13	0,077590535	1,70017E-09	1,99716E-26	0,004942309	1,89926E-07	6,12291E-07	0,010029726	3,78956E-06	2,35936E-07	5,16158E-35	0,038600045	3,15799E-13	0,427679616	0,215568459
1,09116E-08	0,114755038	0,526210871	0,164193468	0,533990489	0,352915875	3,15102E-06	0,150950973	1,55785E-05	0,052427842	3,03123E-08	0,073111096	0,040727413	9,53619E-05	0,126322866	0,000621113	0,000457287	0,4658126	0,463340079	0,03771717	0,000160494	0,031278508	0,001624362	0,021453574	0,294609591	0,003520046	0,024018245	0,000388989	0,000142327	0,038312376	0,000971395	0,038118611	0,055409148	0,252540128	1,93703E-05
1,629E-06	0,117838707	0,004513448	0,500895667	0,928696224	0,471753299	0,225686086	0,00351481	0,03810222	0,469879878	0,635680631	0,000495552	8,83418E-05	0,112912185	0,000588949	0,386845183	4,78923E-05	0,981482386	0,022684445	0,336898052	0,040533835	0,615349818	3,0318E-06	0,000414414	0,991945851	0,014228991	0,339752218	0,071992965	0,003565131	0,2281526	0,011381292	0,022012324	0,471883446	2,23406E-05	0,417753461
0,001874432	0,000277115	5,54276E-18	1,95162E-07	0,031202733	1,48597E-08	0,48758354	0,293747915	0,810710004	2,62343E-09	0,756991769	0,162735752	0,298342661	7,80845E-10	0,454808906	1,88598E-08	0,593498139	1,07425E-10	1,91278E-22	0,328363246	0,135366035	0,271102602	0,06972311	6,04776E-07	0,115873467	0,003602661	1,97206E-05	0,086456054	5,33842E-07	8,36855E-07	2,14441E-17	0,305783954	7,81625E-11	0,649008115	3,55997E-12
0,284533576	0,017139443	0,950899791	0,000229455	0,05354963	0,859798056	0,007751482	0,038455999	4,01071E-08	6,64413E-05	0,025599625	0,003395243	0,002423021	0,753170056	3,45065E-05	0,001382057	0,00122692	0,023421749	0,623811393	2,88095E-05	7,18183E-07	0,669962384	1,87119E-05	0,525124982	0,431776834	0,00117914	0,00093431	0,891096433	0,102865913	0,045946605	0,579588994	9,15592E-05	0,001736848	3,23162E-05	0,001612656
0,087707479	7,75073E-10	2,15269E-12	3,76515E-06	6,60866E-06	2,23556E-10	0,288422465	0,157485842	0,673241777	6,21627E-06	0,466911733	0,007931748	0,622309996	9,32523E-13	6,05172E-05	0,00013311	0,157787952	8,92595E-07	5,21564E-10	1,08769E-05	9,0983E-11	0,264981914	0,238737174	1,67064E-14	0,002050197	0,333833127	4,62661E-07	0,000330912	0,002996146	0,198571409	1,15285E-07	0,558549989	9,75467E-06	0,054961006	0,454639715
1,24034E-06	0,762884712	0,255666484	0,294952622	0,044168467	0,000519141	0,14903117	0,398638169	0,075756729	0,090050267	0,171421904	0,099517314	0,779393321	0,000528024	0,570744524	0,000209595	0,00236342	0,00522877	0,747880435	0,135911531	0,442617072	0,164358231	0,524181939	0,660651388	0,314739429	0,173780793	0,235912059	0,006286446	0,071311413	0,010831073	6,39233E-05	4,82182E-07	0,575258674	1,31216E-09	0,003136453
4,1092E-10	9,13914E-05	0,099875536	0,002332503	0,285255302	0,025293007	1,87997E-07	7,7094E-06	3,11332E-28	0,000273989	0,016855239	5,68921E-06	0,000536158	0,003856179	0,049044353	1,15254E-05	0,162896435	0,022364393	0,003461461	0,000861397	0,955390491	1,22856E-07	5,88001E-08	0,050281456	0,639482337	7,10425E-10	4,2862E-05	1,34916E-07	6,2312E-05	0,000939371	0,506834031	0,000141513	0,000366519	0,809513418	1,46406E-05
0,007045377	2,6756E-05	9,4768E-09	2,55888E-05	0,000971724	6,95016E-12	0,010407502	4,56082E-07	0,567136661	0,001815658	0,03206706	0,320966077	0,050300448	5,22231E-21	0,005133368	3,03729E-22	5,99573E-05	1,30281E-12	1,28342E-16	0,016004383	0,000888417	0,555042976	3,4029E-12	6,94519E-13	1,23514E-17	0,000112771	2,12815E-08	0,37326501	1,70771E-16	1,69586E-06	4,58265E-22	0,280888656	1,2089E-19	0,00375998	0,007070989
0,029454795	0,678869576	0,338359477	0,9974704	0,473336186	0,178074801	0,195542266	0,671053401	0,042076827	0,020704137	0,065231253	2,35748E-07	0,002831094	0,19179979	0,807175769	0,900401319	0,066727659	0,340634201	0,261756022	0,0814378	0,279839065	0,077684107	0,217766018	0,611310824	0,682818866	0,24904783	0,308465351	0,638805567	0,061867144	0,683908292	0,178566549	0,223228554	0,801816554	0,057929887	0,196652566
1,76289E-06	5,21081E-13	1,34548E-25	1,19256E-06	7,06944E-18	4,63046E-09	2,70299E-06	0,002983123	1,44505E-13	0,294773954	7,86715E-09	5,41771E-15	8,8196E-23	2,01577E-39	5,46305E-14	2,09498E-19	2,52972E-10	1,15601E-34	1,43528E-11	8,13326E-34	2,14308E-22	6,51945E-09	1,32504E-11	1,92193E-28	1,81137E-09	2,25477E-13	8,68809E-42	0,046296597	1,38838E-36	1,15797E-16	7,41081E-11	1,5687E-06	2,11847E-13	0,058155184	2,04397E-08
0,000761067	1,80888E-08	1,65126E-13	3,14471E-06	4,38121E-09	0,038906175	0,00441978	0,000432404	0,001499668	1,34972E-08	0,037790503	0,000294557	0,947032946	5,85211E-09	0,171866069	4,93917E-11	2,49887E-09	9,50671E-15	0,007286585	0,055359067	2,95367E-08	0,032398223	8,54346E-12	6,59532E-06	0,255039289	0,778444236	2,69567E-05	0,015909653	0,000120075	5,79671E-05	0,001671102	0,052383224	2,06773E-10	0,103820457	0,000992639
1,25296E-23	2,28111E-23	4,13745E-41	1,29423E-21	1,46963E-10	3,16224E-42	6,86054E-05	5,69846E-15	0,078290665	7,7832E-06	1,28294E-08	0,000688458	6,34357E-12	1,75378E-91	8,90272E-08	3,5668E-100	2,46191E-17	9,22711E-76	6,27346E-33	1,59319E-09	2,52026E-19	0,003419078	1,55188E-32	1,22476E-40	1,41609E-05	1,56676E-05	2,37752E-23	0,004768647	1,28299E-35	3,10914E-28	1,30007E-31	0,001018379	1,42345E-47	0,356915432	6,18895E-09
1,67484E-06	2,09677E-21	2,21206E-18	1,60705E-22	1,02772E-18	1,33142E-23	0,000812015	3,39436E-14	0,487392549	0,099522057	0,047457477	0,265564989	0,010301809	1,02488E-47	4,62596E-06	9,43444E-54	5,37974E-20	3,42982E-38	7,21236E-31	6,25807E-08	1,75958E-11	0,125256982	5,19135E-29	9,59177E-27	6,3938E-10	0,000176919	3,12178E-14	0,003026411	2,58041E-30	2,12942E-16	3,93373E-21	5,06788E-05	5,63034E-45	2,06369E-05	8,31906E-12
2,10491E-11	4,63766E-22	1,08199E-31	2,37337E-18	1,70299E-10	9,6622E-17	1,50243E-05	1,03291E-05	0,071461197	0,024914722	0,018936751	0,026160403	1,99532E-07	7,4557E-46	3,3913E-08	5,52695E-38	2,71313E-18	5,77469E-52	1,02971E-21	2,66881E-08	8,51884E-15	0,001041371	1,44363E-17	2,34712E-28	7,72013E-07	1,53184E-08	8,93188E-20	0,444429718	4,92671E-24	1,19035E-30	1,52973E-13	0,005305032	2,25728E-33	0,009110635	2,40852E-05
1,4992E-23	3,12039E-14	3,19799E-21	2,78286E-13	2,63694E-14	2,06493E-13	0,967618915	1,98822E-09	0,388891116	0,060315854	0,00145975	6,01257E-08	1,16154E-14	1,48547E-37	2,23563E-07	5,54318E-40	1,48189E-09	1,23366E-35	2,16793E-15	1,20284E-18	1,15423E-12	2,44907E-07	9,95337E-24	2,38117E-23	2,07523E-07	2,44837E-06	1,98729E-19	2,03317E-08	3,99238E-40	1,22048E-21	2,07857E-12	7,14743E-09	6,59776E-27	0,095248374	2,17667E-22
0,460379971	5,0301E-06	8,79181E-05	1,80568E-06	4,08481E-14	4,40557E-05	4,45638E-09	0,007457518	2,66712E-13	0,074603122	0,042231488	1,60218E-06	1,30561E-09	5,00895E-12	0,0808674	1,23377E-07	2,35701E-15	4,54382E-07	2,12854E-05	5,01381E-28	3,10147E-08	7,73875E-09	4,74925E-07	9,41384E-05	0,000144169	1,76405E-05	4,5721E-17	5,8645E-06	1,04663E-07	0,019362501	0,010696041	0,000887581	3,86888E-09	0,126054024	2,89252E-05
0,656439553	0,338179705	0,082412192	9,20463E-05	0,304438219	0,414442048	0,005092429	0,006039284	1,0133E-15	1,34281E-06	3,22111E-09	1,25399E-10	1,52855E-08	0,49260632	0,006379562	0,035548205	0,00443046	0,224235286	0,00618197	2,4963E-12	8,60893E-08	0,046979983	0,002692081	0,761837678	3,21567E-12	9,51676E-10	5,10737E-24	0,003600454	0,458856135	0,05540393	0,025666955	0,002982149	0,100738129	0,003749183	0,099077662
0,624840518	0,71775776	0,02140419	0,915362107	0,127042971	8,8112E-12	0,873117514	0,700674395	0,551705652	0,000559169	5,1898E-10	0,000102099	0,020615224	0,000197084	1,25195E-05	0,001815434	0,032933173	0,11410502	0,097330142	1,4293E-05	1,10395E-13	0,00019826	0,661954463	0,009594417	0,000272685	0,002194607	1,00248E-09	0,031399138	0,833020538	0,579884266	6,27466E-07	0,776488809	0,55221698	0,027641986	3,65889E-05
3,38364E-11	0,000199609	4,07657E-07	7,37417E-10	0,085714641	9,79693E-10	0,020130009	0,005195151	0,792406897	5,45738E-06	1,93468E-08	0,535773397	0,002413951	3,32613E-12	0,000912583	3,36838E-12	0,079224077	0,003280951	6,67322E-10	0,492845792	0,036784772	0,269765987	3,74728E-08	1,96271E-13	0,647755418	0,909303406	0,293957682	4,80855E-08	1,23859E-09	0,00609031	0,047808111	0,552370187	1,31908E-09	0,003847324	0,135618279
8,15723E-25	9,64943E-13	1,29217E-13	6,87944E-06	3,11088E-11	0,000131678	4,68411E-06	0,179142936	0,004621232	0,048999105	0,000636753	0,006578521	3,43829E-07	4,46073E-24	3,44986E-08	3,24634E-17	2,43497E-09	3,99019E-19	2,39279E-05	1,09498E-10	1,01422E-16	2,42792E-08	1,05231E-08	1,00314E-17	1,8045E-07	7,03948E-06	1,78925E-22	0,004466477	2,27182E-35	6,04243E-14	1,33861E-06	8,1049E-08	8,07466E-13	0,009700508	1,55814E-07
1,29606E-13	1,5081E-16	5,5349E-36	7,21303E-13	4,09061E-09	7,16336E-34	0,001128336	2,79902E-11	1,35959E-06	0,000233501	0,000161883	2,36935E-07	3,68865E-14	1,88621E-74	4,09297E-31	4,70809E-36	7,00447E-07	1,49656E-30	3,39261E-17	6,96352E-15	2,1281E-37	6,88764E-14	2,32417E-12	4,22721E-77	5,22231E-05	1,75657E-12	1,28047E-23	0,189166521	1,29421E-18	6,22151E-10	2,20023E-25	0,000138315	1,5193E-22	0,132386179	0,656251584
0,144749939	0,940746443	0,020883443	0,079123299	0,188376462	0,030704989	2,29727E-05	0,049675515	9,47729E-08	5,43274E-06	0,387587129	0,219501263	0,062854704	0,001472075	0,005240541	7,33588E-05	6,87103E-15	0,001470437	0,000149964	2,29128E-07	0,027919631	3,0515E-05	0,161700478	0,938134777	0,232046352	0,184431996	0,002514664	0,139633704	0,001988419	0,011683578	8,87393E-05	2,87123E-09	0,044167777	0,014901916	2,52571E-05
0,000565674	0,508273987	0,011357227	0,451237848	3,41544E-05	0,843576669	6,22773E-06	0,000121328	2,75948E-09	0,967286701	0,444595658	9,29004E-18	1,90112E-15	0,023886532	1,18538E-05	0,10667029	0,514073195	0,073664067	0,002071729	4,02813E-22	8,82748E-07	4,76656E-09	0,138444813	0,004122334	0,007860089	0,000453091	1,44099E-13	0,000188214	3,45079E-05	0,041047767	0,537141064	2,09364E-13	0,560656812	0,58382842	0,035072045
0,024751971	0,782001582	0,00036019	0,04079057	0,001065705	0,560709728	2,59662E-08	0,105448552	0,00033402	0,58034484	0,176156005	2,54946E-05	9,34013E-09	0,03462885	0,934911208	0,001064618	0,002306558	0,000159541	0,148481835	3,78412E-06	4,32635E-05	0,026755634	0,000454399	0,993493094	0,112368427	0,002781907	0,02172964	1,44672E-07	1,78925E-06	0,000316549	0,13435726	0,007294314	0,013940495	0,738894637	0,00145666
0,000832665	0,542779788	0,037152381	0,364865193	0,000480482	0,017123097	0,847588846	6,52893E-05	0,324776126	1,33452E-10	0,000679455	0,031516086	0,340680342	0,287081064	0,003002863	0,014969466	2,32832E-15	0,016401512	0,19440593	0,360675564	0,003083642	0,027468621	1,4338E-06	0,033125573	0,268190581	0,135881105	0,529996494	1,4069E-14	0,040957349	6,58368E-12	0,147245586	2,47926E-10	0,019009841	0,000537734	2,79943E-09
2,4886E-05	5,35527E-09	7,14652E-10	0,551741823	0,027356798	0,000245552	0,025182898	0,036997143	9,27175E-07	0,102123257	5,62313E-06	0,001533447	2,51656E-07	1,5789E-10	4,5025E-09	0,257677147	0,087731973	1,44898E-09	1,3544E-07	8,35608E-17	4,42411E-19	0,001132424	0,605897611	1,85862E-16	0,003527958	0,001570463	8,09833E-24	0,000315066	2,09119E-05	0,005323757	3,84706E-05	4,57034E-05	0,189379263	0,035486503	0,521071463
0,004274914	0,81243407	0,001916518	0,149144593	0,017041241	9,6697E-05	0,437590544	0,37687517	0,041800734	2,22311E-09	3,82815E-09	0,794573449	0,418558805	0,006046073	0,050277348	0,042945322	0,000186711	0,894068872	0,001256723	7,3463E-06	0,290156817	0,524177427	0,674792968	3,53234E-05	0,000559267	0,033552233	0,001188035	0,17608103	0,964028082	0,701419553	0,013059027	8,82365E-11	0,112137067	0,571269466	0,070341392
0,043574051	2,73973E-14	6,93614E-14	2,1682E-10	5,49373E-10	7,55574E-07	1,64517E-10	0,000268777	8,44423E-06	0,801662167	0,852840759	0,27148776	0,000265732	1,38342E-18	0,000433825	1,5357E-13	2,48366E-17	9,84988E-14	0,10616535	1,11922E-14	3,82512E-12	0,002848791	2,85965E-09	2,50221E-12	6,88915E-05	5,12371E-08	1,60459E-17	0,187099175	3,25251E-10	0,017487725	1,10479E-07	0,000786793	9,57585E-11	0,696419907	0,868922529
5,62951E-07	6,64583E-32	2,80324E-23	1,42461E-18	2,33063E-18	2,68251E-19	0,000645655	2,41081E-05	0,001361596	2,25528E-05	0,147761564	0,004454641	9,37518E-08	1,36602E-37	9,45342E-16	2,07187E-21	1,12029E-07	9,7743E-22	1,91482E-27	1,44308E-15	7,40266E-23	5,88322E-05	1,80602E-08	6,2078E-33	7,81099E-11	0,000156831	1,03449E-20	0,895311414	4,70104E-15	2,31069E-05	9,82538E-24	2,13225E-05	4,25353E-28	0,374057171	0,020319337
9,92945E-07	4,59913E-13	4,71345E-52	5,41061E-13	1,69626E-07	3,45931E-33	1,36296E-05	0,000151695	2,0407E-05	2,77278E-06	3,23682E-07	0,003351182	2,30102E-08	6,81528E-54	5,81587E-12	2,08677E-39	1,48081E-05	2,79998E-41	1,36899E-21	5,76271E-07	8,7818E-16	1,20539E-06	1,32075E-10	3,55273E-33	8,58332E-09	3,76313E-15	2,01448E-28	0,000340856	3,70093E-24	1,24183E-15	4,0546E-42	0,463051366	1,52214E-26	0,453158479	0,004304123
0,000328065	2,83722E-15	3,59866E-18	9,93679E-14	3,56175E-16	1,13284E-08	9,47571E-05	3,43393E-05	0,116562899	0,000189988	0,008161637	0,043056033	3,49198E-05	9,5634E-30	0,001485145	5,47006E-24	5,01705E-13	5,75318E-29	6,20223E-19	3,22279E-10	5,73586E-12	0,377713177	7,13398E-33	1,53006E-16	0,013552486	0,000283473	1,56623E-15	0,010203766	1,47214E-19	5,65995E-13	1,08334E-12	0,000405347	5,07021E-32	1,3399E-05	2,59378E-12
1,41503E-17	2,41308E-14	4,48123E-30	1,37983E-12	4,60873E-13	4,48527E-24	3,83212E-09	8,9792E-05	1,01975E-06	4,18527E-08	0,024108666	3,3305E-07	3,38427E-17	3,05595E-70	5,02711E-11	5,81605E-36	7,02212E-06	5,0458E-39	9,71779E-17	4,07318E-17	2,03045E-22	3,85444E-09	1,24961E-15	3,7938E-47	0,000908668	0,000223718	7,0532E-23	1,38318E-05	1,94018E-35	1,80149E-15	3,97178E-14	0,002693881	4,05175E-20	0,849006185	0,004431842
0,088881055	0,000710136	4,1958E-05	0,498660197	0,84198656	0,005213735	0,293409134	0,288797114	0,191931883	0,020473123	0,010531881	0,73032639	0,527290838	0,059071727	8,65253E-08	0,068133334	0,164038924	0,938827102	0,000141157	0,020729424	3,25205E-12	0,01637895	0,000292353	5,10489E-09	0,000124377	1,30792E-05	1,52032E-06	6,9601E-10	0,127413819	0,284552525	5,3255E-06	0,510173751	0,569645928	0,629218996	3,50971E-07
7,15934E-07	5,12361E-18	5,08003E-62	2,33228E-12	3,26928E-07	3,04097E-45	4,84405E-09	0,120647849	1,1063E-14	0,00168973	0,010486647	6,72237E-10	9,19413E-18	4,91824E-59	1,0571E-29	4,56992E-27	0,001796103	1,87015E-21	6,13368E-36	9,65709E-24	5,94778E-32	4,81645E-15	0,011918074	2,27545E-50	5,52167E-08	2,13235E-25	4,29166E-40	0,266344774	1,88261E-13	1,68209E-05	7,20032E-46	0,785456716	3,69845E-19	0,634478594	0,639161537
0,753162629	0,234484338	0,015142279	0,555747803	0,21105927	0,003965901	0,005565811	0,108740009	2,24922E-17	0,870778108	0,001285931	2,5582E-08	5,41857E-06	0,040726156	2,75648E-07	0,650796492	0,937465895	0,399415639	0,008185993	1,58373E-20	0,000361465	3,16232E-16	0,058296752	0,011283969	0,010786286	5,37118E-08	9,45388E-15	0,739118294	0,192278726	0,349745824	0,014515406	0,245047111	0,315673846	0,062789973	0,820941441
0,010217709	2,82821E-14	2,07356E-46	4,41001E-10	5,72725E-06	1,53508E-46	1,40974E-09	0,004429225	2,52893E-14	0,003399432	0,000585318	2,66463E-09	2,54981E-11	7,97195E-53	1,53947E-30	3,78859E-26	0,00080804	1,1919E-20	3,26931E-15	1,4648E-23	2,41396E-45	7,36212E-20	0,013713968	2,66408E-41	2,79998E-10	1,22912E-20	4,69398E-37	0,004618036	1,99389E-08	0,012332851	6,31533E-48	0,322004015	1,43775E-11	0,188311783	0,001165113
0,160203518	0,81172854	0,289902421	0,64488347	0,007601093	0,876625422	0,032051992	7,88336E-05	0,695461582	0,775577877	0,767858355	0,121620867	0,205700312	0,566017619	0,084919914	6,96484E-06	0,000143091	0,146374781	0,15141838	0,843634819	0,134236477	0,26579525	2,58134E-06	0,004950198	0,77982007	0,309766599	0,372484234	1,86188E-06	0,085325164	0,180043738	0,400810637	0,076472293	0,072772253	0,508349674	5,26021E-05
9,40462E-12	6,38704E-13	9,50871E-08	0,02306926	1,05438E-13	0,01364802	0,000824145	0,054583259	0,022640253	0,952601376	0,006553972	0,010314405	4,31234E-08	0,002539149	0,011527104	0,0031006	4,75212E-11	0,001603238	4,48295E-08	1,3711E-06	2,33719E-05	7,69008E-06	0,012684758	1,67163E-06	0,225398088	0,009285364	0,001527334	0,020644709	0,006500082	0,001581408	0,502972112	9,31769E-09	0,00070934	0,022037931	0,039208942
8,47537E-08	1,25013E-22	3,47108E-54	3,58059E-15	1,42263E-10	1,1671E-31	1,79943E-08	0,000587492	2,81874E-05	2,97672E-08	0,000190546	6,45835E-07	6,46877E-14	1,1213E-54	2,10781E-18	4,98108E-33	7,27858E-08	4,31939E-39	4,8782E-30	6,2268E-15	1,22124E-33	1,94772E-05	1,00424E-07	2,29382E-37	2,73164E-10	5,0642E-17	3,9752E-31	0,221216558	3,99334E-22	6,17285E-14	6,01305E-41	0,082201112	1,60812E-30	0,699475626	0,013764601
0,432815062	1,7743E-09	3,46022E-16	4,50499E-15	7,05397E-06	6,26345E-31	0,028415177	1,25707E-06	8,64804E-06	0,692269574	0,148775261	0,019691484	0,001593841	5,51046E-21	1,87895E-08	1,32856E-21	1,47058E-05	1,47919E-17	3,06037E-21	1,33535E-05	4,15326E-09	0,006262141	4,84468E-06	4,11078E-17	7,67484E-08	2,46792E-05	7,61869E-09	0,877635039	2,35242E-06	0,000116903	3,60051E-21	0,770708523	9,76716E-15	0,008332624	0,386187928
3,33135E-08	2,79739E-07	1,8864E-19	0,00387271	2,90503E-12	4,75365E-09	2,25858E-10	0,255222058	8,63433E-13	0,407543876	6,25708E-10	2,43802E-11	9,9245E-21	5,44157E-16	1,42299E-05	4,52055E-19	5,86914E-31	1,37792E-28	5,31614E-10	1,1576E-19	6,22529E-08	6,85645E-11	1,5486E-09	1,46095E-07	8,94052E-07	6,37587E-13	5,26063E-28	0,000133247	3,94552E-16	3,13535E-41	9,95805E-07	6,10303E-06	7,13354E-11	0,007433953	1,92755E-06
9,88265E-19	3,76092E-14	1,1732E-15	9,62352E-14	0,000129353	1,31742E-22	0,25694873	1,07601E-11	0,739342351	0,187917263	1,04466E-07	1,38571E-05	3,32449E-12	1,78102E-46	0,004220811	2,72373E-64	1,38144E-07	9,83426E-34	6,97843E-21	5,65173E-09	2,13554E-12	0,000551262	4,07754E-24	2,03381E-23	0,000645828	0,073743889	1,05555E-12	0,001401014	1,04165E-20	7,29506E-21	1,62636E-13	0,000388427	3,68215E-26	0,525012983	7,14753E-07
0,787991281	0,859593657	0,000114204	0,001353478	0,370784214	1,50796E-07	0,449367199	0,219646435	0,249467439	0,000560388	0,036729689	0,030632562	0,669368338	0,000670555	0,660407577	1,60023E-10	2,71122E-06	0,002200168	7,91738E-07	0,006403705	0,020108729	0,816926713	0,450875242	0,467883774	0,923537607	0,024569834	0,266056326	0,790762603	0,002822318	0,005838978	4,67959E-10	1,83373E-11	0,004427647	0,047917326	0,000398056
0,000854504	0,900520593	0,001274989	0,961908787	0,00029975	0,257041814	0,037886792	0,000368609	5,4577E-05	3,55348E-07	8,24697E-11	3,82477E-11	7,2336E-10	0,147228871	0,217830881	0,113302	7,79517E-11	0,017646188	0,116485279	3,07179E-11	0,067010448	0,039010575	6,50043E-06	0,065551632	0,001699492	0,25969423	0,000784285	3,67036E-10	0,001669265	2,7287E-06	2,09779E-07	8,71248E-16	0,221430513	0,001376412	5,95466E-08
0,048307573	2,463E-13	6,37043E-31	2,51794E-12	5,04219E-06	2,32971E-22	0,000238408	6,38846E-05	0,506093109	7,20176E-06	0,164817684	0,10100994	0,007277893	3,23845E-42	6,68525E-06	3,93773E-30	0,06402093	2,86549E-26	4,30006E-22	2,57405E-08	3,63434E-18	0,003525673	4,394E-10	3,13934E-22	6,69057E-06	3,08278E-05	2,34212E-17	0,664961576	5,99427E-12	1,26236E-06	3,10557E-45	0,875631782	3,28523E-19	0,000783247	0,004856931
2,85286E-30	1,17727E-19	5,13194E-45	8,83462E-15	1,19948E-22	1,11152E-22	0,000106574	7,46058E-07	2,39268E-07	0,000955897	1,4453E-08	5,68573E-15	6,67924E-30	8,29481E-55	1,02708E-12	6,1623E-38	9,4256E-14	8,4956E-64	1,78012E-32	2,61785E-35	1,05E-29	5,21966E-10	1,66274E-18	1,92129E-38	8,60127E-10	9,34791E-12	2,75384E-46	1,34102E-06	6,67807E-53	1,85298E-30	3,83176E-17	2,99045E-12	2,52915E-29	0,24310814	6,80561E-21
0,039189677	0,056391698	0,760065498	0,000616914	0,832891062	0,748050888	0,065790532	0,613300565	0,004435745	3,33133E-08	1,015E-13	4,60993E-12	1,09251E-07	0,245430483	0,825189759	0,253348107	0,003804263	0,07787787	0,104703011	0,679229727	0,227927464	0,476008764	0,149443212	0,000176648	0,753810025	0,081763669	0,114578892	0,566915153	0,582976192	4,12581E-05	0,298851531	0,077468223	0,75216966	0,244514866	0,012751537

Supplementary Table S4B. Matrix of p-values for each comparison

PPARA	RAF1.pS338	RHEB	SCD	SGK1	SMARCB1	SOX17	SOX2	SPARC	STAT3.pS727	STAT3.pY705	TNFRSF4	TSC2	TYRO3	UGT1A1	WEE1	WIPI2	ASNS	BABAM1.pS29	BCL2	BMI1	CBX7	CDH1	FASN	FOS	FOXM1	G6PD	GATA1	GSKA_B	H3K27Ac	HSP90AA1_B1	IGFBP2	IRS1	ITGAL	JAG1	MECOM
1,94508E-08	1,4551E-13	3,78223E-09	9,58959E-08	3,87431E-12	2,7535E-06	9,61522E-10	8,73503E-18	1,38482E-09	0,144774628	0,440121501	2,37806E-22	6,01614E-36	9,72902E-06	4,63874E-25	1,09071E-05	9,04423E-10	0,136824462	1,94973E-15	0,019525317	0,109635732	0,000280376	9,03001E-08	1,58765E-09	0,108145279	4,90249E-06	0,0001538	1,09116E-08	1,629E-06	0,001874432	0,284533576	0,087707479	1,24034E-06	4,1092E-10	0,007045377	0,029454795
1,88625E-21	0,075453808	4,83587E-12	1,19853E-11	1,22733E-29	8,34438E-23	1,10933E-24	9,98715E-22	1,37137E-25	0,001421334	0,44730023	3,72264E-33	1,13737E-22	0,000714011	3,57443E-23	1,65253E-07	0,018736354	0,040905751	2,82794E-14	0,001195027	0,051031638	0,000128887	3,93777E-07	2,88182E-06	0,236573601	2,97942E-14	1,2627E-11	0,114755038	0,117838707	0,000277115	0,017139443	7,75073E-10	0,762884712	9,13914E-05	2,6756E-05	0,678869576
1,70422E-09	0,628604084	1,48593E-12	4,27496E-09	1,56588E-36	7,50203E-62	1,60686E-26	3,27471E-32	4,38719E-31	1,67818E-07	0,632044378	3,67478E-30	5,75289E-23	5,86396E-05	5,28081E-27	2,10182E-19	5,56635E-12	0,224977195	1,08214E-10	7,27756E-05	0,413039559	9,53014E-14	4,99646E-14	1,58286E-20	0,038314015	7,40651E-15	6,7647E-26	0,526210871	0,004513448	5,54276E-18	0,950899791	2,15269E-12	0,255666484	0,099875536	9,4768E-09	0,338359477
1,01993E-10	0,165567486	3,86592E-18	6,71489E-24	1,05633E-11	8,44787E-19	1,69098E-14	2,02166E-26	1,02485E-15	2,12423E-06	0,950574817	4,73221E-14	6,61706E-11	0,002540766	4,91469E-28	0,005022579	0,131117675	0,000172407	2,74394E-11	0,000224932	0,022663533	0,164133541	5,66436E-14	6,34528E-10	0,016982863	8,38538E-11	5,4557E-07	0,164193468	0,500895667	1,95162E-07	0,000229455	3,76515E-06	0,294952622	0,002332503	2,55888E-05	0,9974704
4,41206E-31	0,013109653	3,00104E-05	0,000830878	1,75552E-27	4,56911E-10	5,33972E-15	1,62833E-15	6,4517E-07	4,0914E-09	0,331490295	4,00291E-24	3,79036E-18	0,347223815	1,61601E-07	0,000272442	0,003267499	0,130779765	3,4933E-13	0,132673752	0,09575806	1,52414E-07	3,37557E-06	0,126650261	0,013211747	8,55103E-24	0,07229311	0,533990489	0,928696224	0,031202733	0,05354963	6,60866E-06	0,044168467	0,285255302	0,000971724	0,473336186
4,35489E-13	0,03755205	1,75621E-13	1,50275E-14	1,24272E-14	5,56321E-51	1,59701E-19	1,14526E-26	4,81221E-43	2,83444E-06	0,602532734	7,95607E-15	1,10971E-14	0,000385823	5,61397E-34	2,94035E-14	5,47904E-05	7,12749E-05	2,08837E-18	2,38919E-23	0,047642846	0,008010222	1,37495E-10	3,75524E-15	0,040522691	3,43241E-10	3,2126E-29	0,352915875	0,471753299	1,48597E-08	0,859798056	2,23556E-10	0,000519141	0,025293007	6,95016E-12	0,178074801
0,018635935	0,072954908	0,003958754	0,009272328	4,27688E-05	6,7936E-12	0,000711311	0,004380745	0,096459394	0,000673897	0,567147624	0,001208009	0,000680414	0,000918055	6,9661E-06	0,122452391	0,101061397	0,034144205	0,198885855	0,273999795	0,497820576	1,67028E-05	0,036562416	0,0838069	0,661847078	4,11085E-08	0,05412879	3,15102E-06	0,225686086	0,48758354	0,007751482	0,288422465	0,14903117	1,87997E-07	0,010407502	0,195542266
1,23804E-14	0,382478479	2,79654E-06	2,3311E-06	0,000926666	0,003077954	3,65831E-05	1,25493E-19	2,84335E-08	0,000613229	0,789514927	7,96416E-05	4,17447E-08	0,038526735	8,45929E-16	0,006737306	0,087286354	0,886333001	4,78611E-06	0,031810961	0,004027145	0,102048734	0,033292371	0,00330184	0,130421578	0,001525304	9,4569E-07	0,150950973	0,00351481	0,293747915	0,038455999	0,157485842	0,398638169	7,7094E-06	4,56082E-07	0,671053401
0,016552226	0,623484585	0,104802095	0,707595881	3,55324E-06	4,98988E-14	0,002552424	0,956036259	0,016933781	2,21963E-10	0,036947325	0,001088348	0,00500198	0,898643927	0,303850813	7,74193E-15	0,134573267	0,002515238	0,060306164	0,123713505	0,03609148	1,31439E-16	0,119176881	0,056103785	0,995896198	0,003932352	0,92481886	1,55785E-05	0,03810222	0,810710004	4,01071E-08	0,673241777	0,075756729	3,11332E-28	0,567136661	0,042076827
2,06218E-05	0,435753997	4,18817E-05	0,683655703	2,27249E-05	0,000256697	4,23118E-05	0,010017422	0,004085491	0,481326483	0,037147499	1,72394E-05	0,932500982	0,00050503	0,001839261	0,173692478	0,66783463	0,187790839	0,134517472	0,000309037	0,919501403	0,077029359	0,000147443	3,93069E-11	0,392531064	0,003550884	0,01797287	0,052427842	0,469879878	2,62343E-09	6,64413E-05	6,21627E-06	0,090050267	0,000273989	0,001815658	0,020704137
1,14201E-12	2,78491E-05	0,712614141	0,892514652	0,058385479	0,000579592	0,591803708	9,52856E-07	3,08738E-07	0,778537763	0,003216123	0,004931925	1,14171E-09	0,135021013	0,000157613	1,00869E-18	1,59206E-06	4,74662E-12	0,000268005	0,01969155	0,367720464	3,3421E-05	0,000495351	0,919577111	0,006344197	0,353963311	0,00022218	3,03123E-08	0,635680631	0,756991769	0,025599625	0,466911733	0,171421904	0,016855239	0,03206706	0,065231253
1,38128E-05	0,001383291	0,007962016	0,182627582	0,025019285	8,41036E-08	0,334100275	0,383586886	0,029346218	1,72405E-08	0,040982988	0,005228067	9,52034E-07	0,086304741	0,049049926	4,74662E-20	4,98614E-17	0,001410432	2,52235E-08	0,174447326	0,409668983	4,74255E-07	3,66227E-06	0,35927671	0,116711026	0,216979173	0,46789203	0,073111096	0,000495552	0,162735752	0,003395243	0,007931748	0,099517314	5,68921E-06	0,320966077	2,35748E-07
1,26234E-10	2,54935E-05	0,00022099	0,001028359	2,62331E-08	3,30753E-15	0,000418436	3,41902E-05	1,01024E-05	3,51351E-09	0,262387228	4,07382E-12	3,36417E-15	0,707980342	3,92873E-07	3,91125E-18	3,50505E-13	0,004184652	5,00405E-16	0,23628192	0,929368064	1,62264E-12	1,10501E-07	0,015186917	0,002288067	0,00020077	0,01281642	0,040727413	8,83418E-05	0,298342661	0,002423021	0,622309996	0,779393321	0,000536158	0,050300448	0,002831094

Supplementary Table S4B. Matrix of p-values for each comparison

NLN	NOTCH1	NOTCH1.cle	PAX6	PDCD4	SGK3	SMAD2	STK4	SUZ12	AURORA_A_B_C.pT288_232_198	BRAF.pS445	EEF2K	EIF4E	GAB2	HK2	HSPB1.pS82	INPP4B	MAP1LC3A_B	MTOR	PDL1	PEA15	PIM2	PTEN	RAB11	SMARCA2	STAT1	TP53BP1	TUBA1A	XPF	ARID1A	EIF2AK2	HSF1.pS326	ADM	EIF4G1
1,76289E-06	0,000761067	1,25296E-23	1,67484E-06	2,10491E-11	1,4992E-23	0,460379971	0,656439553	0,624840518	3,38364E-11	8,15723E-25	1,29606E-13	0,144749939	0,000565674	0,024751971	0,000832665	2,4886E-05	0,004274914	0,043574051	5,62951E-07	9,92945E-07	0,000328065	1,41503E-17	0,088881055	7,15934E-07	0,753162629	0,010217709	0,160203518	9,40462E-12	8,47537E-08	0,432815062	3,33135E-08	9,88265E-19	0,787991281
5,21081E-13	1,80888E-08	2,28111E-23	2,09677E-21	4,63766E-22	3,12039E-14	5,0301E-06	0,338179705	0,71775776	0,000199609	9,64943E-13	1,5081E-16	0,940746443	0,508273987	0,782001582	0,542779788	5,35527E-09	0,81243407	2,73973E-14	6,64583E-32	4,59913E-13	2,83722E-15	2,41308E-14	0,000710136	5,12361E-18	0,234484338	2,82821E-14	0,81172854	6,38704E-13	1,25013E-22	1,7743E-09	2,79739E-07	3,76092E-14	0,859593657
1,34548E-25	1,65126E-13	4,13745E-41	2,21206E-18	1,08199E-31	3,19799E-21	8,79181E-05	0,082412192	0,02140419	4,07657E-07	1,29217E-13	5,5349E-36	0,020883443	0,011357227	0,00036019	0,037152381	7,14652E-10	0,001916518	6,93614E-14	2,80324E-23	4,71345E-52	3,59866E-18	4,48123E-30	4,1958E-05	5,08003E-62	0,015142279	2,07356E-46	0,289902421	9,50871E-08	3,47108E-54	3,46022E-16	1,8864E-19	1,1732E-15	0,000114204
1,19256E-06	3,14471E-06	1,29423E-21	1,60705E-22	2,37337E-18	2,78286E-13	1,80568E-06	9,20463E-05	0,915362107	7,37417E-10	6,87944E-06	7,21303E-13	0,079123299	0,451237848	0,04079057	0,364865193	0,551741823	0,149144593	2,1682E-10	1,42461E-18	5,41061E-13	9,93679E-14	1,37983E-12	0,498660197	2,33228E-12	0,555747803	4,41001E-10	0,64488347	0,02306926	3,58059E-15	4,50499E-15	0,00387271	9,62352E-14	0,001353478
7,06944E-18	4,38121E-09	1,46963E-10	1,02772E-18	1,70299E-10	2,63694E-14	4,08481E-14	0,304438219	0,127042971	0,085714641	3,11088E-11	4,09061E-09	0,188376462	3,41544E-05	0,001065705	0,000480482	0,027356798	0,017041241	5,49373E-10	2,33063E-18	1,69626E-07	3,56175E-16	4,60873E-13	0,84198656	3,26928E-07	0,21105927	5,72725E-06	0,007601093	1,05438E-13	1,42263E-10	7,05397E-06	2,90503E-12	0,000129353	0,370784214
4,63046E-09	0,038906175	3,16224E-42	1,33142E-23	9,6622E-17	2,06493E-13	4,40557E-05	0,414442048	8,8112E-12	9,79693E-10	0,000131678	7,16336E-34	0,030704989	0,843576669	0,560709728	0,017123097	0,000245552	9,6697E-05	7,55574E-07	2,68251E-19	3,45931E-33	1,13284E-08	4,48527E-24	0,005213735	3,04097E-45	0,003965901	1,53508E-46	0,876625422	0,01364802	1,1671E-31	6,26345E-31	4,75365E-09	1,31742E-22	1,50796E-07
2,70299E-06	0,00441978	6,86054E-05	0,000812015	1,50243E-05	0,967618915	4,45638E-09	0,005092429	0,873117514	0,020130009	4,68411E-06	0,001128336	2,29727E-05	6,22773E-06	2,59662E-08	0,847588846	0,025182898	0,437590544	1,64517E-10	0,000645655	1,36296E-05	9,47571E-05	3,83212E-09	0,293409134	4,84405E-09	0,005565811	1,40974E-09	0,032051992	0,000824145	1,79943E-08	0,028415177	2,25858E-10	0,25694873	0,449367199
0,002983123	0,000432404	5,69846E-15	3,39436E-14	1,03291E-05	1,98822E-09	0,007457518	0,006039284	0,700674395	0,005195151	0,179142936	2,79902E-11	0,049675515	0,000121328	0,105448552	6,52893E-05	0,036997143	0,37687517	0,000268777	2,41081E-05	0,000151695	3,43393E-05	8,9792E-05	0,288797114	0,120647849	0,108740009	0,004429225	7,88336E-05	0,054583259	0,000587492	1,25707E-06	0,255222058	1,07601E-11	0,219646435
1,44505E-13	0,001499668	0,078290665	0,487392549	0,071461197	0,388891116	2,66712E-13	1,0133E-15	0,551705652	0,792406897	0,004621232	1,35959E-06	9,47729E-08	2,75948E-09	0,00033402	0,324776126	9,27175E-07	0,041800734	8,44423E-06	0,001361596	2,0407E-05	0,116562899	1,01975E-06	0,191931883	1,1063E-14	2,24922E-17	2,52893E-14	0,695461582	0,022640253	2,81874E-05	8,64804E-06	8,63433E-13	0,739342351	0,249467439
0,294773954	1,34972E-08	7,7832E-06	0,099522057	0,024914722	0,060315854	0,074603122	1,34281E-06	0,000559169	5,45738E-06	0,048999105	0,000233501	5,43274E-06	0,967286701	0,58034484	1,33452E-10	0,102123257	2,22311E-09	0,801662167	2,25528E-05	2,77278E-06	0,000189988	4,18527E-08	0,020473123	0,00168973	0,870778108	0,003399432	0,775577877	0,952601376	2,97672E-08	0,692269574	0,407543876	0,187917263	0,000560388
7,86715E-09	0,037790503	1,28294E-08	0,047457477	0,018936751	0,00145975	0,042231488	3,22111E-09	5,1898E-10	1,93468E-08	0,000636753	0,000161883	0,387587129	0,444595658	0,176156005	0,000679455	5,62313E-06	3,82815E-09	0,852840759	0,147761564	3,23682E-07	0,008161637	0,024108666	0,010531881	0,010486647	0,001285931	0,000585318	0,767858355	0,006553972	0,000190546	0,148775261	6,25708E-10	1,04466E-07	0,036729689
5,41771E-15	0,000294557	0,000688458	0,265564989	0,026160403	6,01257E-08	1,60218E-06	1,25399E-10	0,000102099	0,535773397	0,006578521	2,36935E-07	0,219501263	9,29004E-18	2,54946E-05	0,031516086	0,001533447	0,794573449	0,27148776	0,004454641	0,003351182	0,043056033	3,3305E-07	0,73032639	6,72237E-10	2,5582E-08	2,66463E-09	0,121620867	0,010314405	6,45835E-07	0,019691484	2,43802E-11	1,38571E-05	0,030632562
8,8196E-23	0,947032946	6,34357E-12	0,010301809	1,99532E-07	1,16154E-14	1,30561E-09	1,52855E-08	0,020615224	0,002413951	3,43829E-07	3,68865E-14	0,062854704	1,90112E-15	9,34013E-09	0,340680342	2,51656E-07	0,418558805	0,000265732	9,37518E-08	2,30102E-08	3,49198E-05	3,38427E-17	0,527290838	9,19413E-18	5,41857E-06	2,54981E-11	0,205700312	4,31234E-08	6,46877E-14	0,001593841	9,9245E-21	3,32449E-12	0,669368338
2,01577E-39	5,85211E-09	1,75378E-91	1,02488E-47	7,4557E-46	1,48547E-37	5,00895E-12	0,49260632	0,000197084	3,32613E-12	4,46073E-24	1,88621E-74	0,001472075	0,023886532	0,03462885	0,287081064	1,5789E-10	0,006046073	1,38342E-18	1,36602E-37	6,81528E-54	9,5634E-30	3,05595E-70	0,059071727	4,91824E-59	0,040726156	7,97195E-53	0,566017619	0,002539149	1,1213E-54	5,51046E-21	5,44157E-16	1,78102E-46	0,000670555
5,46305E-14	0,171866069	8,90272E-08	4,62596E-06	3,3913E-08	2,23563E-07	0,0808674	0,006379562	1,25195E-05	0,000912583	3,44986E-08	4,09297E-31	0,005240541	1,18538E-05	0,934911208	0,003002863	4,5025E-09	0,050277348	0,000433825	9,45342E-16	5,81587E-12	0,001485145	5,02711E-11	8,65253E-08	1,0571E-29	2,75648E-07	1,53947E-30	0,084919914	0,011527104	2,10781E-18	1,87895E-08	1,42299E-05	0,004220811	0,660407577
2,09498E-19	4,93917E-11	3,5668E-100	9,43444E-54	5,52695E-38	5,54318E-40	1,23377E-07	0,035548205	0,001815434	3,36838E-12	3,24634E-17	4,70809E-36	7,33588E-05	0,10667029	0,001064618	0,014969466	0,257677147	0,042945322	1,5357E-13	2,07187E-21	2,08677E-39	5,47006E-24	5,81605E-36	0,068133334	4,56992E-27	0,650796492	3,78859E-26	6,96484E-06	0,0031006	4,98108E-33	1,32856E-21	4,52055E-19	2,72373E-64	1,60023E-10
2,52972E-10	2,49887E-09	2,46191E-17	5,37974E-20	2,71313E-18	1,48189E-09	2,35701E-15	0,00443046	0,032933173	0,079224077	2,43497E-09	7,00447E-07	6,87103E-15	0,514073195	0,002306558	2,32832E-15	0,087731973	0,000186711	2,48366E-17	1,12029E-07	1,48081E-05	5,01705E-13	7,02212E-06	0,164038924	0,001796103	0,937465895	0,00080804	0,000143091	4,75212E-11	7,27858E-08	1,47058E-05	5,86914E-31	1,38144E-07	2,71122E-06
1,15601E-34	9,50671E-15	9,22711E-76	3,42982E-38	5,77469E-52	1,23366E-35	4,54382E-07	0,224235286	0,11410502	0,003280951	3,99019E-19	1,49656E-30	0,001470437	0,073664067	0,000159541	0,016401512	1,44898E-09	0,894068872	9,84988E-14	9,7743E-22	2,79998E-41	5,75318E-29	5,0458E-39	0,938827102	1,87015E-21	0,399415639	1,1919E-20	0,146374781	0,001603238	4,31939E-39	1,47919E-17	1,37792E-28	9,83426E-34	0,002200168
1,43528E-11	0,007286585	6,27346E-33	7,21236E-31	1,02971E-21	2,16793E-15	2,12854E-05	0,00618197	0,097330142	6,67322E-10	2,39279E-05	3,39261E-17	0,000149964	0,002071729	0,148481835	0,19440593	1,3544E-07	0,001256723	0,10616535	1,91482E-27	1,36899E-21	6,20223E-19	9,71779E-17	0,000141157	6,13368E-36	0,008185993	3,26931E-15	0,15141838	4,48295E-08	4,8782E-30	3,06037E-21	5,31614E-10	6,97843E-21	7,91738E-07
8,13326E-34	0,055359067	1,59319E-09	6,25807E-08	2,66881E-08	1,20284E-18	5,01381E-28	2,4963E-12	1,4293E-05	0,492845792	1,09498E-10	6,96352E-15	2,29128E-07	4,02813E-22	3,78412E-06	0,360675564	8,35608E-17	7,3463E-06	1,11922E-14	1,44308E-15	5,76271E-07	3,22279E-10	4,07318E-17	0,020729424	9,65709E-24	1,58373E-20	1,4648E-23	0,843634819	1,3711E-06	6,2268E-15	1,33535E-05	1,1576E-19	5,65173E-09	0,006403705
2,14308E-22	2,95367E-08	2,52026E-19	1,75958E-11	8,51884E-15	1,15423E-12	3,10147E-08	8,60893E-08	1,10395E-13	0,036784772	1,01422E-16	2,1281E-37	0,027919631	8,82748E-07	4,32635E-05	0,003083642	4,42411E-19	0,290156817	3,82512E-12	7,40266E-23	8,7818E-16	5,73586E-12	2,03045E-22	3,25205E-12	5,94778E-32	0,000361465	2,41396E-45	0,134236477	2,33719E-05	1,22124E-33	4,15326E-09	6,22529E-08	2,13554E-12	0,020108729
6,51945E-09	0,032398223	0,003419078	0,125256982	0,001041371	2,44907E-07	7,73875E-09	0,046979983	0,00019826	0,269765987	2,42792E-08	6,88764E-14	3,0515E-05	4,76656E-09	0,026755634	0,027468621	0,001132424	0,524177427	0,002848791	5,88322E-05	1,20539E-06	0,377713177	3,85444E-09	0,01637895	4,81645E-15	3,16232E-16	7,36212E-20	0,26579525	7,69008E-06	1,94772E-05	0,006262141	6,85645E-11	0,000551262	0,816926713
1,32504E-11	8,54346E-12	1,55188E-32	5,19135E-29	1,44363E-17	9,95337E-24	4,74925E-07	0,002692081	0,661954463	3,74728E-08	1,05231E-08	2,32417E-12	0,161700478	0,138444813	0,000454399	1,4338E-06	0,605897611	0,674792968	2,85965E-09	1,80602E-08	1,32075E-10	7,13398E-33	1,24961E-15	0,000292353	0,011918074	0,058296752	0,013713968	2,58134E-06	0,012684758	1,00424E-07	4,84468E-06	1,5486E-09	4,07754E-24	0,450875242
1,92193E-28	6,59532E-06	1,22476E-40	9,59177E-27	2,34712E-28	2,38117E-23	9,41384E-05	0,761837678	0,009594417	1,96271E-13	1,00314E-17	4,22721E-77	0,938134777	0,004122334	0,993493094	0,033125573	1,85862E-16	3,53234E-05	2,50221E-12	6,2078E-33	3,55273E-33	1,53006E-16	3,7938E-47	5,10489E-09	2,27545E-50	0,011283969	2,66408E-41	0,004950198	1,67163E-06	2,29382E-37	4,11078E-17	1,46095E-07	2,03381E-23	0,467883774
1,81137E-09	0,255039289	1,41609E-05	6,3938E-10	7,72013E-07	2,07523E-07	0,000144169	3,21567E-12	0,000272685	0,647755418	1,8045E-07	5,22231E-05	0,232046352	0,007860089	0,112368427	0,268190581	0,003527958	0,000559267	6,88915E-05	7,81099E-11	8,58332E-09	0,013552486	0,000908668	0,000124377	5,52167E-08	0,010786286	2,79986E-10	0,77982007	0,225398088	2,73164E-10	7,67484E-08	8,94052E-07	0,000645828	0,923537607
2,25477E-13	0,778444236	1,56676E-05	0,000176919	1,53184E-08	2,44837E-06	1,76405E-05	9,51676E-10	0,002194607	0,909303406	7,03948E-06	1,75657E-12	0,184431996	0,000453091	0,002781907	0,135881105	0,001570463	0,033552233	5,12371E-08	0,000156831	3,76313E-15	0,000283473	0,000223718	1,30792E-05	2,13235E-25	5,37118E-08	1,22912E-20	0,309766599	0,009285364	5,0642E-17	2,46792E-05	6,37587E-13	0,073743889	0,024569834
8,68809E-42	2,69567E-05	2,37752E-23	3,12178E-14	8,93188E-20	1,98729E-19	4,5721E-17	5,10737E-24	1,00248E-09	0,293957682	1,78925E-22	1,28047E-23	0,002514664	1,44099E-13	0,02172964	0,529996494	8,09833E-24	0,001188035	1,60459E-17	1,03449E-20	2,01448E-28	1,56623E-15	7,0532E-23	1,52032E-06	4,29166E-40	9,45388E-15	4,69398E-37	0,372484234	0,001527334	3,9752E-31	7,61869E-09	5,26063E-28	1,05555E-12	0,266056326
0,046296597	0,015909653	0,004768647	0,003026411	0,444429718	2,03317E-08	5,8645E-06	0,003600454	0,031399138	4,80855E-08	0,004466477	0,189166521	0,139633704	0,000188214	1,44672E-07	1,4069E-14	0,000315066	0,17608103	0,187099175	0,895311414	0,000340856	0,010203766	1,38318E-05	6,9601E-10	0,266344774	0,739118294	0,004618036	1,86188E-06	0,020644709	0,221216558	0,877635039	0,000133247	0,001401014	0,790762603
1,38838E-36	0,000120075	1,28299E-35	2,58041E-30	4,92671E-24	3,99238E-40	1,04663E-07	0,458856135	0,833020538	1,23859E-09	2,27182E-35	1,29421E-18	0,001988419	3,45079E-05	1,78925E-06	0,040957349	2,09119E-05	0,964028082	3,25251E-10	4,70104E-15	3,70093E-24	1,47214E-19	1,94018E-35	0,127413819	1,88261E-13	0,192278726	1,99389E-08	0,085325164	0,006500082	3,99334E-22	2,35242E-06	3,94552E-16	1,04165E-20	0,002822318
1,15797E-16	5,79671E-05	3,10914E-28	2,12942E-16	1,19035E-30	1,22048E-21	0,019362501	0,05540393	0,579884266	0,00609031	6,04243E-14	6,22151E-10	0,011683578	0,041047767	0,000316549	6,58368E-12	0,005323757	0,701419553	0,017487725	2,31069E-05	1,24183E-15	5,65995E-13	1,80149E-15	0,284552525	1,68209E-05	0,349745824	0,012332851	0,180043738	0,001581408	6,17285E-14	0,000116903	3,13535E-41	7,29506E-21	0,005838978
7,41081E-11	0,001671102	1,30007E-31	3,93373E-21	1,52973E-13	2,07857E-12	0,010696041	0,025666955	6,27466E-07	0,047808111	1,33861E-06	2,20023E-25	8,87393E-05	0,537141064	0,13435726	0,147245586	3,84706E-05	0,013059027	1,10479E-07	9,82538E-24	4,0546E-42	1,08334E-12	3,97178E-14	5,3255E-06	7,20032E-46	0,014515406	6,31533E-48	0,400810637	0,502972112	6,01305E-41	3,60051E-21	9,95805E-07	1,62636E-13	4,67959E-10
1,5687E-06	0,052383224	0,001018379	5,06788E-05	0,005305032	7,14743E-09	0,000887581	0,002982149	0,776488809	0,552370187	8,1049E-08	0,000138315	2,87123E-09	2,09364E-13	0,007294314	2,47926E-10	4,57034E-05	8,82365E-11	0,000786793	2,13225E-05	0,463051366	0,000405347	0,002693881	0,510173751	0,785456716	0,245047111	0,322004015	0,076472293	9,31769E-09	0,082201112	0,770708523	6,10303E-06	0,000388427	1,83373E-11
2,11847E-13	2,06773E-10	1,42345E-47	5,63034E-45	2,25728E-33	6,59776E-27	3,86888E-09	0,100738129	0,55221698	1,31908E-09	8,07466E-13	1,5193E-22	0,044167777	0,560656812	0,013940495	0,019009841	0,189379263	0,112137067	9,57585E-11	4,25353E-28	1,52214E-26	5,07021E-32	4,05175E-20	0,569645928	3,69845E-19	0,315673846	1,43775E-11	0,072772253	0,00070934	1,60812E-30	9,76716E-15	7,13354E-11	3,68215E-26	0,004427647
0,058155184	0,103820457	0,356915432	2,06369E-05	0,009110635	0,095248374	0,126054024	0,003749183	0,027641986	0,003847324	0,009700508	0,132386179	0,014901916	0,58382842	0,738894637	0,000537734	0,035486503	0,571269466	0,696419907	0,374057171	0,453158479	1,3399E-05	0,849006185	0,629218996	0,634478594	0,062789973	0,188311783	0,508349674	0,022037931	0,699475626	0,008332624	0,007433953	0,525012983	0,047917326
2,04397E-08	0,000992639	6,18895E-09	8,31906E-12	2,40852E-05	2,17667E-22	2,89252E-05	0,099077662	3,65889E-05	0,135618279	1,55814E-07	0,656251584	2,52571E-05	0,035072045	0,00145666	2,79943E-09	0,521071463	0,070341392	0,868922529	0,020319337	0,004304123	2,59378E-12	0,004431842	3,50971E-07	0,639161537	0,820941441	0,001165113	5,26021E-05	0,039208942	0,013764601	0,386187928	1,92755E-06	7,14753E-07	0,000398056
2,5129E-19	0,001339234	1,27918E-20	6,85888E-33	2,83836E-17	5,61048E-21	1,58608E-21	0,002879076	0,068521236	0,022439647	8,74043E-12	5,56411E-17	0,01620977	0,022857823	0,003933632	1,82806E-05	0,050378047	0,000210659	2,34842E-21	5,43854E-25	2,16361E-09	3,47243E-20	2,88316E-14	0,703867458	1,81451E-13	0,672030528	2,12115E-14	0,001436602	1,35742E-09	3,2413E-19	5,49134E-15	2,19586E-16	1,43117E-14	0,023505917
0,000266244	0,488162331	0,000128203	0,87259572	4,19972E-05	5,57693E-06	0,000374381	0,232707836	0,784439991	0,516104569	1,04895E-05	0,250396344	0,002427983	0,000752095	0,049207075	0,334946222	5,25695E-05	0,196352849	4,49344E-05	0,169804819	0,016678935	0,786476747	0,008331843	0,140903599	0,591169747	0,048856299	0,005336851	0,517199789	0,186359698	0,45983062	0,000430501	0,002725844	2,12087E-06	0,262814062
5,85989E-06	8,36555E-06	3,09447E-16	1,24737E-17	1,27293E-10	9,85788E-16	6,31391E-07	0,06982528	0,283969768	1,0204E-11	1,26127E-09	6,13015E-18	0,108635287	0,003854817	0,242159553	0,786208591	0,001278719	0,006817878	1,42984E-08	6,95699E-27	6,18694E-09	1,35086E-14	2,38673E-12	1,90438E-09	6,24363E-20	0,004805801	1,71414E-11	0,992367841	0,00010159	3,40497E-16	9,08538E-06	0,001016974	1,10406E-09	0,061286987
2,64115E-05	0,011241623	1,81957E-18	3,85415E-17	2,1313E-12	6,75659E-14	0,000378148	0,000147854	0,698622978	9,53148E-16	1,54562E-07	8,73814E-16	0,055754595	0,297825174	0,147890938	0,16364942	0,040802852	0,015706839	1,82585E-10	2,68017E-10	2,4448E-07	2,5636E-17	7,48778E-12	0,13008747	4,82598E-13	0,818886209	3,74674E-07	0,559042489	0,000899648	1,71847E-11	8,49897E-06	5,63945E-05	2,30027E-13	0,980936332
7,35389E-13	6,55555E-13	4,73297E-18	7,2552E-24	3,77378E-18	8,51647E-13	5,9442E-11	0,927679245	0,971655095	3,00891E-05	2,21863E-20	1,21534E-24	0,177725231	0,000397425	0,045490014	0,053657014	5,91934E-12	0,594663638	2,70664E-15	1,39888E-58	1,48327E-20	1,03635E-17	7,97725E-19	2,44699E-11	1,21985E-35	0,00017411	6,99336E-23	0,179696335	2,30748E-19	1,37171E-38	1,90216E-11	4,87743E-11	4,37102E-05	0,347918127
1,18194E-35	7,10834E-09	3,79658E-50	1,52779E-32	4,5656E-44	6,16471E-26	7,4211E-13	0,056992827	6,375E-05	5,42476E-11	4,07456E-17	5,02934E-52	0,047762989	0,00316216	0,145675043	9,88924E-07	6,04545E-14	3,10557E-05	2,61645E-16	1,67445E-42	5,61026E-58	1,96159E-24	1,28781E-44	2,01982E-08	2,2673E-158	6,05266E-06	5,76173E-79	0,231090403	3,54954E-07	8,9321E-106	3,65179E-20	1,02774E-21	4,76089E-23	1,0009E-05
7,49798E-08	1,72411E-06	4,43817E-22	2,09002E-28	2,41108E-15	3,1357E-12	9,21617E-10	0,203633504	0,441991503	3,90885E-07	3,53928E-15	4,32045E-24	0,593014791	0,023684432	0,124853004	0,069430548	6,98216E-09	0,028589153	1,29996E-11	2,9633E-50	1,44663E-17	3,37429E-17	5,84245E-19	1,38726E-12	3,39131E-32	5,10212E-05	2,87912E-22	0,061717363	4,74866E-13	7,38881E-30	1,92901E-18	7,93308E-07	1,22922E-08	0,888934956
4,72385E-23	3,5567E-19	6,6967E-105	3,32237E-55	2,33181E-49	6,15362E-42	4,45988E-06	0,001076652	0,145742984	1,34987E-15	3,74841E-21	1,4897E-30	2,01507E-10	0,005095886	8,75694E-07	0,135999326	0,007051618	0,00492509	1,17335E-09	2,62454E-23	2,14756E-62	2,38354E-36	3,10806E-34	0,657701437	1,28581E-27	0,247237744	2,29835E-17	6,95234E-06	6,7924E-10	4,19696E-42	1,0907E-18	1,68598E-23	1,97528E-48	8,87524E-10
3,19858E-19	1,71063E-07	7,62587E-49	3,23516E-40	8,06818E-33	4,16481E-23	0,000196741	0,49411301	0,048926415	2,83782E-07	1,07455E-16	3,43645E-37	0,060282806	0,061035375	0,937109733	0,000595727	7,10002E-18	0,117796795	3,77969E-10	3,66243E-40	2,60971E-38	3,63569E-26	4,65462E-32	2,04591E-17	8,37799E-45	0,006799911	6,61279E-32	0,002398674	1,91266E-05	1,98872E-45	3,61375E-19	1,46815E-10	4,59588E-30	0,132314924
1,00514E-05	0,87193303	5,1389E-07	2,11506E-13	2,08801E-10	1,39255E-06	3,96981E-07	0,032287069	6,77177E-05	0,106042544	0,000573973	0,003009647	0,857973804	0,013450242	0,020009159	0,000262295	0,341825028	0,096335997	0,333487691	2,36592E-05	6,00477E-05	0,000377781	0,000574843	0,009777093	1,71691E-06	0,165501707	0,004023153	0,001642672	7,41018E-05	8,29405E-05	4,86484E-14	4,43184E-24	0,000486156	6,17561E-05
0,270818683	0,00702919	0,021953708	0,387548531	0,261848707	0,647329384	0,608501107	0,302169413	0,007755636	0,88427697	0,672312729	0,124532637	0,161791884	0,23148189	0,723418295	0,014592035	0,118999771	0,328449957	0,236136392	0,462971298	0,043010288	0,001244998	0,072684126	0,001759312	0,906979825	0,524630946	0,100676345	0,275697511	0,836063113	0,104199509	0,06344872	0,036165187	0,03028708	0,340444846
1,18297E-16	1,34984E-15	1,23197E-30	5,17252E-27	1,34512E-22	7,24173E-23	2,51213E-10	0,714587694	0,817839055	2,34157E-12	6,43101E-20	1,49363E-26	0,21087974	0,006438665	0,007198792	0,521257024	1,43806E-09	0,00672627	3,94287E-12	9,76183E-52	2,97024E-25	5,5417E-22	2,63482E-19	1,89352E-08	2,8684E-36	0,455081333	6,14479E-15	0,833646237	7,46642E-31	1,07915E-40	5,09938E-12	1,28796E-15	4,32689E-11	0,793003956
2,99617E-28	9,11705E-08	4,4233E-51	2,28681E-32	7,0305E-27	7,76754E-60	3,40016E-16	0,267257419	0,000122788	1,09942E-08	2,0029E-33	1,83023E-30	0,458591186	0,000126816	0,034500573	0,066398632	4,75934E-05	0,793019048	6,95785E-16	3,15746E-26	7,81305E-23	3,40397E-21	4,70857E-28	0,262973732	5,19501E-32	0,027374707	1,67173E-22	0,005044591	5,80445E-08	2,44126E-33	1,89096E-07	7,21492E-21	2,48025E-35	0,221370442
0,011191741	0,001340765	1,3855E-14	1,42289E-12	3,09157E-12	3,11207E-05	0,49534754	0,00058922	0,340279251	7,77173E-18	0,000254005	2,02129E-05	0,025285188	0,220323112	2,93914E-06	0,470364351	0,905237136	1,07641E-07	0,173078721	0,000271214	3,23421E-08	1,01927E-08	4,7393E-08	0,934551303	1,14909E-06	0,032270487	0,033330789	0,048133171	0,000134093	4,17806E-08	0,000272815	0,000665224	2,28162E-05	0,029714989
5,70806E-18	3,61295E-10	2,7738E-102	1,90801E-48	1,64422E-41	4,93757E-44	2,32394E-08	0,143031803	0,000288601	2,11054E-23	1,92828E-32	2,01909E-44	0,015121504	0,471546278	0,001350796	0,193161147	5,09848E-06	0,003773391	6,0354E-18	1,05226E-29	4,96959E-47	6,39007E-28	8,03571E-38	0,243642023	1,23468E-36	0,283806318	7,53209E-27	0,006247111	0,00018999	1,45038E-41	3,68768E-15	3,1039E-18	1,20559E-52	0,00017658
2,3283E-12	0,087912452	1,12922E-10	3,10378E-05	2,2951E-08	5,36361E-05	0,03566503	1,54213E-12	0,002023892	0,623560487	3,46568E-11	2,78885E-21	0,025692777	0,002490001	0,675267363	0,829392281	1,25562E-12	0,936979521	0,00066892	8,16766E-11	5,59335E-20	0,000547748	1,03072E-08	1,18525E-11	1,09116E-21	0,000101971	7,07744E-16	0,318719761	0,00015001	1,76861E-22	0,000269462	7,36951E-09	0,000513112	0,273375361
1,73298E-08	0,98250472	2,72672E-18	1,39604E-05	4,89264E-14	2,56329E-14	0,199127425	0,120558475	0,057742205	0,098780671	8,42329E-05	1,69878E-05	1,03774E-22	0,010782249	0,000941285	0,321897135	0,228877099	0,039986865	3,01837E-07	0,159449469	2,12541E-16	9,90644E-05	3,97126E-06	0,870076329	2,69085E-15	0,879792715	3,08751E-07	0,256453558	0,303644561	1,91967E-20	0,018925449	2,6573E-17	3,26639E-19	4,34473E-29
0,000377957	0,779449856	0,002603775	9,04246E-11	3,50026E-05	0,000119063	0,000366907	0,269402142	0,241268621	1,01222E-10	0,064263589	1,91757E-05	0,025943149	0,000189115	0,20852672	0,008260238	0,479049305	7,0927E-06	0,010459305	8,52272E-05	0,012782471	1,3328E-05	2,32489E-06	0,740672556	4,90056E-12	0,017220319	6,05359E-05	0,401488547	0,147063214	2,92989E-06	0,000119337	0,303591791	0,004567064	1,12043E-07
2,22167E-25	1,40983E-08	1,82299E-34	2,62867E-27	1,50576E-29	2,0214E-41	2,85535E-06	0,061305337	0,002158031	9,28848E-07	8,78644E-19	3,5857E-26	0,018265913	8,34272E-06	0,007065205	0,88788674	6,79892E-05	0,508141821	8,44656E-09	3,80424E-21	2,02526E-12	9,99558E-19	4,63254E-29	0,183069295	5,27918E-21	0,007108342	6,30165E-15	0,070029111	0,006981254	2,79305E-19	4,66391E-08	2,33857E-18	1,55176E-35	0,001133872
0,492637081	0,22303214	1,70224E-05	6,57911E-06	0,007658607	0,045745126	0,506266175	1,07058E-06	0,000115207	1,45189E-05	0,386698949	1,23593E-05	1,71498E-05	0,004258683	0,215067435	5,83221E-13	0,774300819	1,91548E-11	0,010956861	3,89961E-08	3,01841E-09	0,026723183	0,001351934	0,021600058	8,82001E-17	0,098888874	6,00536E-19	0,930696546	0,528038331	1,3123E-08	4,33155E-07	0,196219681	0,001764831	7,59691E-12
0,980980985	0,750743483	0,015514511	0,00022633	0,095920183	0,023469857	0,277827801	0,050454532	0,002748074	0,138854076	0,052479936	0,000561112	0,009935997	0,178412036	0,909513013	0,175107805	0,16746126	0,791739942	0,005902244	0,032908219	0,98802286	0,013374237	0,010910905	0,588249917	0,437082219	0,179335546	0,599880182	0,233775636	0,299467934	0,323543769	0,117499403	0,398497635	0,014349845	0,176417074
2,96675E-16	0,000687295	0,026166634	0,047713488	3,27788E-05	0,002053969	4,64586E-09	2,32825E-11	0,316286468	0,060049002	1,71484E-07	2,07531E-07	2,36312E-06	4,33439E-11	0,137850961	0,00914715	6,21865E-19	0,360179701	4,48129E-07	1,53162E-13	7,67355E-07	0,007359199	6,94623E-06	8,39973E-16	4,16622E-12	0,000196353	1,03738E-06	3,70939E-05	9,25488E-12	1,27464E-12	0,086642537	2,02155E-07	0,641203066	3,60244E-08
8,6983E-09	0,00092338	3,69922E-17	3,6294E-20	8,44279E-13	5,81766E-26	5,97576E-05	0,000604868	0,009748745	2,40972E-18	2,30009E-07	1,15222E-16	8,42165E-05	0,002311906	0,432513414	0,003124625	0,184294676	6,94037E-07	3,51677E-05	5,18511E-19	1,09725E-11	2,4144E-11	1,11359E-14	0,858836519	4,177E-26	0,191660503	1,00985E-18	0,092889922	0,707306953	1,90102E-18	4,93376E-08	0,000429895	8,61609E-19	1,90039E-12
0,000251866	2,84633E-08	5,55829E-38	1,53286E-19	2,57329E-19	4,00346E-27	0,758967041	0,000116943	8,26237E-05	3,20209E-16	3,69925E-10	1,90635E-10	8,48251E-19	0,727175922	0,086316987	0,004784395	0,038110216	6,10803E-09	0,132876509	1,10792E-12	8,34244E-33	8,29391E-18	3,06384E-13	0,731957708	2,13076E-21	0,909534358	5,83546E-12	0,018559139	0,404981953	7,33804E-29	0,000756961	1,02078E-06	9,69125E-23	6,56142E-26
2,33291E-06	0,020226155	0,0013469	0,000620181	0,000300979	1,13284E-05	0,578334438	0,210027029	0,013661272	0,177088264	0,060422986	0,184099596	0,102994577	3,86383E-11	0,086338892	0,872839365	4,12848E-05	0,042333544	0,288150076	0,000104739	0,195050102	0,001111577	0,003475333	0,179390512	0,026123667	0,068295549	0,252806597	0,563111787	0,997513414	0,000621247	0,236903773	0,058171641	0,0122825	0,306474807
6,41523E-13	2,51481E-17	6,78363E-17	1,15868E-39	1,97702E-15	1,0885E-14	4,36959E-25	0,207838574	0,210858484	9,02298E-09	1,74109E-13	3,36383E-15	0,365874908	0,00117755	0,000411963	0,585553212	0,009721914	0,881065686	1,71136E-16	4,59113E-45	1,35848E-11	1,44132E-22	6,29536E-21	0,188463925	1,20306E-23	0,047784755	1,24639E-18	0,020320479	2,22928E-10	3,13704E-23	5,69754E-13	7,88971E-08	3,29819E-07	0,521487709
5,72015E-16	5,15168E-09	7,72813E-46	2,29299E-14	1,23942E-28	2,96277E-16	0,403096764	0,871931664	0,000524066	7,48542E-06	0,002882153	9,03147E-30	5,10294E-10	0,029535152	0,449770746	0,520226014	0,031793296	3,44373E-07	2,65922E-05	1,39138E-07	1,15168E-36	1,89707E-10	2,28021E-21	0,073496752	4,11044E-32	0,415864072	1,80562E-29	0,444431508	0,114371116	4,90652E-31	8,4779E-13	5,21942E-09	7,987E-31	3,01559E-11
0,050510675	0,606867891	0,000182853	0,038700958	0,855310934	1,47375E-09	0,259710862	0,000819432	0,436105155	1,92754E-08	0,372217227	0,006380635	3,18064E-09	1,08388E-05	0,453055428	0,924739865	0,279490797	7,18598E-08	0,433324383	0,272391646	0,276974064	0,005302576	2,49687E-06	0,022183605	0,050748563	0,808121142	0,653017613	0,141908032	0,022924736	0,735568158	0,855569924	0,223897686	8,33048E-11	8,65424E-08
0,007949389	0,144682563	0,087036849	0,012391513	5,07494E-05	0,521779828	1,45865E-06	0,023123433	0,15094618	1,04612E-05	0,024534784	0,016350077	6,91549E-07	0,967916987	0,842333238	0,000553263	0,021462073	5,36191E-08	1,81429E-07	0,672786171	0,000228924	0,009150165	0,245394948	0,007359318	1,00716E-05	0,047181064	0,234514135	0,001429659	0,000169747	1,98962E-06	0,150403182	0,008365213	0,286087933	6,9507E-05
0,000887058	1,8921E-08	2,58882E-18	1,47871E-13	1,0538E-09	4,90699E-10	0,233131119	0,157259311	0,035628425	8,21911E-06	0,005243676	0,000335516	5,53601E-17	0,944110055	0,887089211	0,00041303	6,15901E-05	6,04742E-06	0,939116288	1,85675E-15	6,01363E-32	9,81064E-17	2,28461E-06	2,37822E-06	2,63663E-19	0,675852488	0,006921809	0,875547258	0,119854653	7,5601E-31	1,61905E-05	8,9925E-05	5,18676E-06	4,98449E-12
0,555004729	0,001811111	0,004235439	0,168771464	0,03708648	0,000189276	0,04910978	9,86939E-14	0,892858571	0,087141291	0,125618649	0,603525083	1,88341E-20	0,008897402	0,022935867	0,006575179	0,000652207	7,35166E-08	0,001011589	0,827668668	0,000745985	0,023123684	0,016685204	1,75718E-06	0,025384252	0,02847475	0,116328197	0,008751666	0,00221323	0,940864199	0,164832101	0,007853433	1,60059E-05	1,01274E-15
2,52703E-06	2,36287E-15	8,22111E-08	9,2931E-11	4,64561E-10	1,30085E-06	0,037728785	0,117824514	0,480663779	0,001216579	0,000371162	1,22478E-08	0,50917621	0,404695502	0,55660669	1,08261E-07	6,06811E-10	0,054633796	8,79319E-10	1,10994E-26	2,8122E-10	1,00624E-09	2,25675E-10	2,02425E-06	9,68451E-18	0,457243432	2,83506E-10	0,017668241	0,00156359	7,94494E-17	1,52031E-05	0,507088536	0,000858732	0,646885183
0,550991997	0,065516707	0,016216228	0,524031991	0,661276916	0,928873922	0,653297523	0,001331755	0,000231897	0,025875377	0,001059679	0,315108191	2,78659E-07	0,294892743	0,513014039	0,000930442	0,495803567	0,657206461	0,88940261	0,797558874	0,011391508	0,359454389	0,008692858	1,1238E-07	0,145433071	0,010025003	3,30509E-08	0,002779719	1,0774E-12	0,532382932	0,043845954	0,331997886	0,01558311	5,67853E-16
0,369643456	0,001504437	3,99063E-06	5,75892E-07	0,000772615	2,67834E-15	0,01153268	9,1914E-13	0,051080835	1,67568E-07	0,10565516	0,085125078	9,23208E-09	0,226075013	0,893250288	0,47796073	0,921471188	0,033530251	0,956503845	9,59389E-05	0,266064234	3,56506E-05	2,55499E-05	0,405242465	0,438789224	0,000384473	0,024990804	0,37861156	0,353980343	0,053275222	0,715922561	0,000389975	7,53495E-08	0,062116152
1,00062E-09	0,040829985	2,18267E-19	5,64819E-24	1,25444E-14	2,36439E-10	6,2318E-05	0,000393181	0,697658838	0,005757473	2,53291E-13	5,7126E-09	0,03267523	0,971985381	0,879938249	0,000102972	0,020421119	0,18783441	0,000139358	1,49246E-08	3,18969E-17	6,00943E-10	7,3506E-08	0,009166731	2,48481E-13	0,575122012	3,70748E-10	0,537218691	0,141406157	8,37588E-14	7,38841E-09	1,69421E-09	1,14741E-14	0,007484967
0,449967889	0,025054311	0,199009863	0,554315369	0,348418302	0,183598407	1,55042E-05	0,420480784	0,006075878	0,129135969	0,002255174	0,823296666	0,004720947	0,011619235	0,028304418	0,050044184	3,00032E-05	0,002005685	0,184849505	0,110170832	0,043907089	0,129852659	0,002050312	0,045199892	0,487717641	0,987650313	0,051090486	0,207899194	0,144007528	0,491597919	0,025287255	0,058709568	0,489072466	0,628864692
0	1,13169E-07	3,74373E-30	2,98923E-19	1,50583E-29	9,92399E-33	3,50528E-17	4,51464E-07	0,039123934	0,001829442	2,12489E-18	8,06625E-34	0,583535896	3,80267E-10	0,00084112	0,626608417	9,04679E-11	0,564980069	7,90888E-17	8,19399E-19	3,32172E-22	1,08181E-23	3,32284E-30	0,012045111	3,44068E-32	0,001774637	1,75711E-24	0,356602315	0,000413059	3,01418E-29	1,77591E-10	2,64475E-20	3,39299E-17	0,806887835
1,13169E-07	0	1,33696E-17	7,78224E-13	4,38123E-16	2,68128E-10	0,001184759	0,001411435	0,477872906	1,10856E-05	0,00504631	2,77241E-07	0,001519533	0,031378162	4,2074E-06	0,282937464	0,009573857	0,524464481	1,4757E-11	1,58938E-13	6,70708E-13	5,09602E-23	1,08501E-08	0,473079804	3,8976E-05	0,032709143	0,000384721	0,140511025	0,011652679	7,63163E-14	0,096661701	0,050578137	5,48214E-06	0,357174258
3,74373E-30	1,33696E-17	0	7,64703E-48	6,90211E-52	1,75747E-53	1,05857E-07	0,026286486	0,001541438	2,38727E-22	6,67983E-24	1,47214E-60	7,2941E-09	0,881467516	1,23451E-08	0,977717301	9,42162E-05	1,41812E-06	3,37866E-13	7,6393E-25	1,98507E-71	3,1611E-35	8,73834E-72	0,839486134	6,01199E-45	0,658295448	7,85291E-34	0,000167935	1,53311E-05	2,88234E-54	1,53474E-17	4,05613E-24	1,05813E-75	3,9538E-09
2,98923E-19	7,78224E-13	7,64703E-48	0	9,2887E-38	9,00578E-30	1,08654E-13	0,042720656	0,819289132	4,65359E-13	6,24081E-19	1,52437E-26	0,052097672	0,793789635	0,045335256	0,076930026	0,021551772	0,73458372	3,3825E-14	2,03174E-34	1,34072E-27	3,73481E-53	1,59388E-27	0,273959528	2,65681E-24	0,386444866	1,00516E-17	0,002089017	0,002423545	4,27675E-34	1,11371E-22	5,18039E-13	4,75321E-20	0,000121208
1,50583E-29	4,38123E-16	6,90211E-52	9,2887E-38	0	2,55765E-30	4,14492E-06	0,630712914	0,806674658	1,85165E-08	1,92516E-20	5,06188E-33	1,6714E-05	0,876332031	0,004658059	0,779370645	5,58916E-08	0,006215226	7,34173E-10	5,53039E-24	8,35787E-38	8,83458E-25	1,70528E-27	0,026611766	3,17997E-31	0,850704529	2,65699E-25	0,933568953	0,000191932	3,72729E-49	1,12E-11	7,22276E-29	2,40243E-29	3,62429E-05
9,92399E-33	2,68128E-10	1,75747E-53	9,00578E-30	2,55765E-30	0	3,20599E-10	0,03015743	0,005887523	1,77744E-13	8,83192E-24	3,21887E-29	2,1703E-05	9,3711E-06	6,1125E-05	0,652233233	9,21473E-06	0,108802935	1,79465E-11	1,89015E-22	6,74365E-25	6,79533E-27	4,99508E-33	0,543324246	1,18092E-28	0,045277037	2,55037E-16	0,119798932	0,00755819	5,08709E-32	2,81789E-07	4,87156E-13	2,38337E-47	8,40783E-05
3,50528E-17	0,001184759	1,05857E-07	1,08654E-13	4,14492E-06	3,20599E-10	0	0,007729895	0,892529549	0,003249885	9,08467E-05	1,94245E-08	1,01117E-09	0,009961092	0,017163633	0,005480403	0,396796393	0,019538537	1,45205E-28	9,94888E-17	0,001761247	9,65795E-13	4,95243E-09	0,093830049	1,1249E-11	1,87726E-05	3,47074E-13	0,000869205	1,36537E-07	4,72656E-08	3,44934E-07	1,39177E-10	7,22475E-05	0,004656311
4,51464E-07	0,001411435	0,026286486	0,042720656	0,630712914	0,03015743	0,007729895	0	0,002199573	3,88398E-17	0,008639958	0,896406415	7,4085E-09	9,41103E-09	0,001257318	8,28899E-05	1,2408E-08	1,5179E-09	0,023221624	0,611156652	0,22024313	0,093827259	0,061399489	1,03109E-11	0,041653465	6,05314E-05	0,009052894	7,71129E-06	0,643145634	0,045791974	0,394633281	1,04693E-05	0,004931535	3,8127E-08
0,039123934	0,477872906	0,001541438	0,819289132	0,806674658	0,005887523	0,892529549	0,002199573	0	0,605028811	0,012241753	1,12119E-06	0,653786762	0,314965385	0,306046435	0,004881396	0,001568452	0,956326711	0,10064776	0,142860068	1,92232E-05	0,731706392	0,000386023	0,01136992	4,42374E-05	0,00064603	6,74751E-18	0,390067129	0,013255241	6,57328E-06	0,69422958	0,789820447	0,000150695	0,22142131
0,001829442	1,10856E-05	2,38727E-22	4,65359E-13	1,85165E-08	1,77744E-13	0,003249885	3,88398E-17	0,605028811	0	2,27948E-05	6,51214E-17	0,046473715	0,730812035	1,20258E-07	0,004253247	0,878091904	7,76514E-24	3,22144E-06	3,36658E-07	1,22883E-07	1,11994E-11	1,23302E-25	0,124743818	1,49151E-12	0,094684204	1,15867E-05	0,002175153	2,90224E-06	4,88126E-10	0,001838523	0,040122712	9,29775E-13	0,007171145
2,12489E-18	0,00504631	6,67983E-24	6,24081E-19	1,92516E-20	8,83192E-24	9,08467E-05	0,008639958	0,012241753	2,27948E-05	0	2,75215E-25	0,311116866	0,000235817	0,10352422	0,269727268	7,09194E-13	0,653569239	1,2184E-11	3,15625E-14	1,92922E-19	2,69828E-13	7,95444E-25	2,55607E-05	3,56541E-14	6,67799E-06	1,74896E-14	0,826412023	2,90969E-05	1,2614E-15	0,064449606	3,4063E-14	2,86985E-15	0,22458292
8,06625E-34	2,77241E-07	1,47214E-60	1,52437E-26	5,06188E-33	3,21887E-29	1,94245E-08	0,896406415	1,12119E-06	6,51214E-17	2,75215E-25	0	0,815131927	0,013399664	2,6766E-07	0,028109554	4,33581E-09	4,19816E-05	5,64756E-18	8,9417E-29	2,06298E-38	4,41838E-21	1,98164E-78	0,00012441	9,17782E-51	0,000648088	2,22557E-56	0,769200246	1,90765E-06	1,38067E-37	9,59441E-13	2,67136E-09	2,87094E-36	0,802340079
0,583535896	0,001519533	7,2941E-09	0,052097672	1,6714E-05	2,1703E-05	1,01117E-09	7,4085E-09	0,653786762	0,046473715	0,311116866	0,815131927	0	0,000463341	0,009698248	8,42318E-06	0,161887308	1,06197E-06	6,17239E-18	0,69786127	4,49856E-12	0,032301128	0,003549246	0,004405581	0,060568397	4,68017E-05	0,247121174	0,005367966	3,06997E-06	6,20588E-07	0,052828827	0,395891924	9,36681E-11	9,37626E-60
3,80267E-10	0,031378162	0,881467516	0,793789635	0,876332031	9,3711E-06	0,009961092	9,41103E-09	0,314965385	0,730812035	0,000235817	0,013399664	0,000463341	0	0,056171341	0,838988175	2,37314E-14	0,345641364	0,137571626	0,000655109	0,093486603	0,00022436	4,53523E-07	2,57403E-05	3,32044E-06	4,53005E-10	0,062510334	0,090451432	0,112121429	0,007205809	0,625680054	0,027872706	0,329320627	0,002172995
0,00084112	4,2074E-06	1,23451E-08	0,045335256	0,004658059	6,1125E-05	0,017163633	0,001257318	0,306046435	1,20258E-07	0,10352422	2,6766E-07	0,009698248	0,056171341	0	0,530476704	0,962559369	0,000985424	0,066507132	0,289824705	0,000726442	3,05985E-06	3,26094E-12	4,26031E-05	0,267848006	0,762992478	0,01906668	2,54767E-07	0,000607221	0,002091658	0,28129537	0,00130097	1,75242E-05	0,2410154
0,626608417	0,282937464	0,977717301	0,076930026	0,779370645	0,652233233	0,005480403	8,28899E-05	0,004881396	0,004253247	0,269727268	0,028109554	8,42318E-06	0,838988175	0,530476704	0	4,41496E-05	3,16333E-06	0,596739854	0,005979213	0,000107252	0,859432191	0,003700555	2,36369E-05	2,74939E-07	0,187894505	2,6847E-06	5,28613E-05	0,03186535	0,000299416	0,866130885	2,07705E-08	0,261551804	0,002448351
9,04679E-11	0,009573857	9,42162E-05	0,021551772	5,58916E-08	9,21473E-06	0,396796393	1,2408E-08	0,001568452	0,878091904	7,09194E-13	4,33581E-09	0,161887308	2,37314E-14	0,962559369	4,41496E-05	0	0,068926599	1,83139E-05	3,33455E-11	1,0009E-09	1,69747E-06	4,09137E-10	4,28428E-21	6,8968E-12	1,06914E-08	5,26258E-10	2,19894E-07	0,250280109	3,38885E-13	0,843919333	0,011059497	0,000652377	1,617E-05
0,564980069	0,524464481	1,41812E-06	0,73458372	0,006215226	0,108802935	0,019538537	1,5179E-09	0,956326711	7,76514E-24	0,653569239	4,19816E-05	1,06197E-06	0,345641364	0,000985424	3,16333E-06	0,068926599	0	0,13062318	0,300116771	0,000103306	0,007242459	5,58878E-09	0,93542412	1,08273E-06	0,094147859	0,177739557	0,698668303	0,076115614	3,04089E-05	0,498826709	0,766437855	0,00034551	1,07111E-12
7,90888E-17	1,4757E-11	3,37866E-13	3,3825E-14	7,34173E-10	1,79465E-11	1,45205E-28	0,023221624	0,10064776	3,22144E-06	1,2184E-11	5,64756E-18	6,17239E-18	0,137571626	0,066507132	0,596739854	1,83139E-05	0,13062318	0	3,62369E-19	1,69177E-07	2,18361E-14	2,95196E-20	0,193387175	2,08148E-14	0,002007193	2,0563E-17	0,394063638	0,001338591	2,47534E-10	5,02084E-06	0,000611817	3,84299E-05	2,99106E-09
8,19399E-19	1,58938E-13	7,6393E-25	2,03174E-34	5,53039E-24	1,89015E-22	9,94888E-17	0,611156652	0,142860068	3,36658E-07	3,15625E-14	8,9417E-29	0,69786127	0,000655109	0,289824705	0,005979213	3,33455E-11	0,300116771	3,62369E-19	0	1,31685E-21	7,61656E-27	3,3904E-22	6,06702E-07	7,17337E-35	0,053088318	7,92131E-26	0,821708554	3,77222E-09	3,20264E-43	3,97274E-16	2,2987E-05	4,04049E-13	0,987544071
3,32172E-22	6,70708E-13	1,98507E-71	1,34072E-27	8,35787E-38	6,74365E-25	0,001761247	0,22024313	1,92232E-05	1,22883E-07	1,92922E-19	2,06298E-38	4,49856E-12	0,093486603	0,000726442	0,000107252	1,0009E-09	0,000103306	1,69177E-07	1,31685E-21	0	2,50551E-22	5,20133E-39	0,000292531	3,24129E-46	0,04071591	9,12781E-40	0,562913508	0,014968033	3,9745E-57	1,15876E-14	1,19779E-20	3,19296E-31	4,79287E-13
1,08181E-23	5,09602E-23	3,1611E-35	3,73481E-53	8,83458E-25	6,79533E-27	9,65795E-13	0,093827259	0,731706392	1,11994E-11	2,69828E-13	4,41838E-21	0,032301128	0,00022436	3,05985E-06	0,859432191	1,69747E-06	0,007242459	2,18361E-14	7,61656E-27	2,50551E-22	0	1,24557E-27	0,001718641	4,39291E-23	0,000850564	1,18034E-11	0,049543722	0,00285268	1,6326E-33	1,74756E-05	9,09062E-09	5,76176E-16	0,040798804
3,32284E-30	1,08501E-08	8,73834E-72	1,59388E-27	1,70528E-27	4,99508E-33	4,95243E-09	0,061399489	0,000386023	1,23302E-25	7,95444E-25	1,98164E-78	0,003549246	4,53523E-07	3,26094E-12	0,003700555	4,09137E-10	5,58878E-09	2,95196E-20	3,3904E-22	5,20133E-39	1,24557E-27	0	0,082114147	3,96037E-39	0,001217498	9,92409E-33	0,081586769	6,12236E-05	1,93958E-34	1,30998E-09	1,79422E-08	5,68202E-41	0,0582754
0,012045111	0,473079804	0,839486134	0,273959528	0,026611766	0,543324246	0,093830049	1,03109E-11	0,01136992	0,124743818	2,55607E-05	0,00012441	0,004405581	2,57403E-05	4,26031E-05	2,36369E-05	4,28428E-21	0,93542412	0,193387175	6,06702E-07	0,000292531	0,001718641	0,082114147	0	1,2002E-08	0,004850679	0,000108602	1,62318E-18	0,10226399	5,12025E-07	0,333520808	0,812216799	0,665250017	2,33787E-05
3,44068E-32	3,8976E-05	6,01199E-45	2,65681E-24	3,17997E-31	1,18092E-28	1,1249E-11	0,041653465	4,42374E-05	1,49151E-12	3,56541E-14	9,17782E-51	0,060568397	3,32044E-06	0,267848006	2,74939E-07	6,8968E-12	1,08273E-06	2,08148E-14	7,17337E-35	3,24129E-46	4,39291E-23	3,96037E-39	1,2002E-08	0	2,79886E-08	2,49255E-74	0,180935501	2,69027E-05	1,30196E-80	4,19591E-14	1,11162E-17	1,70997E-24	2,04772E-06
0,001774637	0,032709143	0,658295448	0,386444866	0,850704529	0,045277037	1,87726E-05	6,05314E-05	0,00064603	0,094684204	6,67799E-06	0,000648088	4,68017E-05	4,53005E-10	0,762992478	0,187894505	1,06914E-08	0,094147859	0,002007193	0,053088318	0,04071591	0,000850564	0,001217498	0,004850679	2,79886E-08	0	1,17443E-07	0,350228694	0,549214966	0,081625281	5,68194E-11	0,064819766	0,588557933	0,415167582
1,75711E-24	0,000384721	7,85291E-34	1,00516E-17	2,65699E-25	2,55037E-16	3,47074E-13	0,009052894	6,74751E-18	1,15867E-05	1,74896E-14	2,22557E-56	0,247121174	0,062510334	0,01906668	2,6847E-06	5,26258E-10	0,177739557	2,0563E-17	7,92131E-26	9,12781E-40	1,18034E-11	9,92409E-33	0,000108602	2,49255E-74	1,17443E-07	0	0,3070341	0,079520723	2,29433E-48	6,10003E-19	3,91139E-12	3,14505E-20	8,83023E-06
0,356602315	0,140511025	0,000167935	0,002089017	0,933568953	0,119798932	0,000869205	7,71129E-06	0,390067129	0,002175153	0,826412023	0,769200246	0,005367966	0,090451432	2,54767E-07	5,28613E-05	2,19894E-07	0,698668303	0,394063638	0,821708554	0,562913508	0,049543722	0,081586769	1,62318E-18	0,180935501	0,350228694	0,3070341	0	0,207143753	0,513049612	0,176339949	0,040907684	0,003343761	0,000191608
0,000413059	0,011652679	1,53311E-05	0,002423545	0,000191932	0,00755819	1,36537E-07	0,643145634	0,013255241	2,90224E-06	2,90969E-05	1,90765E-06	3,06997E-06	0,112121429	0,000607221	0,03186535	0,250280109	0,076115614	0,001338591	3,77222E-09	0,014968033	0,00285268	6,12236E-05	0,10226399	2,69027E-05	0,549214966	0,079520723	0,207143753	0	5,53172E-06	0,003977687	3,60792E-09	0,021284721	1,9981E-05
3,01418E-29	7,63163E-14	2,88234E-54	4,27675E-34	3,72729E-49	5,08709E-32	4,72656E-08	0,045791974	6,57328E-06	4,88126E-10	1,2614E-15	1,38067E-37	6,20588E-07	0,007205809	0,002091658	0,000299416	3,38885E-13	3,04089E-05	2,47534E-10	3,20264E-43	3,9745E-57	1,6326E-33	1,93958E-34	5,12025E-07	1,30196E-80	0,081625281	2,29433E-48	0,513049612	5,53172E-06	0	2,4875E-16	1,20863E-18	3,33805E-21	1,84519E-07
1,77591E-10	0,096661701	1,53474E-17	1,11371E-22	1,12E-11	2,81789E-07	3,44934E-07	0,394633281	0,69422958	0,001838523	0,064449606	9,59441E-13	0,052828827	0,625680054	0,28129537	0,866130885	0,843919333	0,498826709	5,02084E-06	3,97274E-16	1,15876E-14	1,74756E-05	1,30998E-09	0,333520808	4,19591E-14	5,68194E-11	6,10003E-19	0,176339949	0,003977687	2,4875E-16	0	7,59119E-08	4,57156E-07	5,69265E-05
2,64475E-20	0,050578137	4,05613E-24	5,18039E-13	7,22276E-29	4,87156E-13	1,39177E-10	1,04693E-05	0,789820447	0,040122712	3,4063E-14	2,67136E-09	0,395891924	0,027872706	0,00130097	2,07705E-08	0,011059497	0,766437855	0,000611817	2,2987E-05	1,19779E-20	9,09062E-09	1,79422E-08	0,812216799	1,11162E-17	0,064819766	3,91139E-12	0,040907684	3,60792E-09	1,20863E-18	7,59119E-08	0	1,03386E-13	0,003724912
3,39299E-17	5,48214E-06	1,05813E-75	4,75321E-20	2,40243E-29	2,38337E-47	7,22475E-05	0,004931535	0,000150695	9,29775E-13	2,86985E-15	2,87094E-36	9,36681E-11	0,329320627	1,75242E-05	0,261551804	0,000652377	0,00034551	3,84299E-05	4,04049E-13	3,19296E-31	5,76176E-16	5,68202E-41	0,665250017	1,70997E-24	0,588557933	3,14505E-20	0,003343761	0,021284721	3,33805E-21	4,57156E-07	1,03386E-13	0	5,14534E-10
0,806887835	0,357174258	3,9538E-09	0,000121208	3,62429E-05	8,40783E-05	0,004656311	3,8127E-08	0,22142131	0,007171145	0,22458292	0,802340079	9,37626E-60	0,002172995	0,2410154	0,002448351	1,617E-05	1,07111E-12	2,99106E-09	0,987544071	4,79287E-13	0,040798804	0,0582754	2,33787E-05	2,04772E-06	0,415167582	8,83023E-06	0,000191608	1,9981E-05	1,84519E-07	5,69265E-05	0,003724912	5,14534E-10	0
0,009677573	0,003398803	0,595968989	0,633635303	0,019402711	0,149182575	5,0571E-05	1,03834E-06	0,459624962	0,01498467	0,543216276	0,280305076	1,75144E-12	0,041228966	0,230103126	1,25556E-17	0,131616906	4,77025E-09	0,196365754	0,077649704	0,000507701	0,025967186	0,919676985	5,87823E-05	1,95905E-06	0,46204628	6,74652E-06	0,012388047	0,002836903	0,000730364	0,285688285	5,39271E-05	0,927726285	3,70772E-10
2,02337E-17	6,47501E-15	1,6018E-38	3,29208E-35	8,81862E-26	3,07596E-28	1,60916E-06	0,132718656	1,31643E-05	0,000101304	1,64298E-08	2,29685E-23	3,43418E-12	0,000620927	0,010860323	7,35747E-06	1,8301E-09	0,046220841	1,08198E-08	3,95599E-31	1,99595E-31	1,49209E-34	2,16142E-29	0,000347471	4,05745E-44	0,00122929	1,77611E-44	0,757023568	0,109506976	1,31657E-47	5,81708E-13	0,000144638	1,12736E-28	6,18172E-13
6,48492E-45	1,00828E-25	7,42381E-75	1,23048E-38	6,4171E-41	8,88962E-68	8,21036E-11	0,138200601	0,000496148	5,3391E-13	5,02722E-23	3,41475E-47	0,000107969	1,78326E-09	0,008026495	9,71638E-14	[illegible]	[illegible]	[illegible]	[illegible]	[illegible]	[illegible]	[illegible]	[illegible]	[illegible]	[illegible]	[illegible]	[illegible]	[illegible]	[illegible]	[illegible]	[illegible]	[illegible]	[illegible]
0,84224764	0,000620584	0,457383639	0,00250992	0,20522523	0,650495864	0,025757606	3,85863E-05	0,678087358	0,000101073	0,060962526	0,073979058	0,870708078	0,633685825	0,660336327	8,34174E-09	7,60235E-05	0,541563606	1,88553E-05	2,28923E-08	0,745811335	0,002570079	0,000934796	6,74199E-06	0,10721894	0,006429021	0,013491726	0,000121692	0,604011986	0,218641335	0,193191639	1,24713E-08	0,39291179	0,043714323

Supplementary Table S4B. Matrix of p-values for each comparison

H3K27Me3	LMNB1	SPI1	WEE1.pS642
0,000854504	0,048307573	2,85286E-30	0,039189677
0,900520593	2,463E-13	1,17727E-19	0,056391698
0,001274989	6,37043E-31	5,13194E-45	0,760065498
0,961908787	2,51794E-12	8,83462E-15	0,000616914
0,00029975	5,04219E-06	1,19948E-22	0,832891062
0,257041814	2,32971E-22	1,11152E-22	0,748050888
0,037886792	0,000238408	0,000106574	0,065790532
0,000368609	6,38846E-05	7,46058E-07	0,613300565
5,4577E-05	0,506093109	2,39268E-07	0,004435745
3,55348E-07	7,20176E-06	0,000955897	3,33133E-08
8,24697E-11	0,164817684	1,4453E-08	1,015E-13
3,82477E-11	0,10100994	5,68573E-15	4,60993E-12
7,2336E-10	0,007277893	6,67924E-30	1,09251E-07
0,147228871	3,23845E-42	8,29481E-55	0,245430483
0,217830881	6,68525E-06	1,02708E-12	0,825189759
0,113302	3,93773E-30	6,1623E-38	0,253348107
7,79517E-11	0,06402093	9,4256E-14	0,003804263
0,017646188	2,86549E-26	8,4956E-64	0,07787787
0,116485279	4,30006E-22	1,78012E-32	0,104703011
3,07179E-11	2,57405E-08	2,61785E-35	0,679229727
0,067010448	3,63434E-18	1,05E-29	0,227927464
0,039010575	0,003525673	5,21966E-10	0,476008764
6,50043E-06	4,394E-10	1,66274E-18	0,149443212
0,065551632	3,13934E-22	1,92129E-38	0,000176648
0,001699492	6,69057E-06	8,60127E-10	0,753810025
0,25969423	3,08278E-05	9,34791E-12	0,081763669
0,000784285	2,34212E-17	2,75384E-46	0,114578892
3,67036E-10	0,664961576	1,34102E-06	0,566915153
0,001669265	5,99427E-12	6,67807E-53	0,582976192
2,7287E-06	1,26236E-06	1,85298E-30	4,12581E-05
2,09779E-07	3,10557E-45	3,83176E-17	0,298851531
8,71248E-16	0,875631782	2,99045E-12	0,077468223
0,221430513	3,28523E-19	2,52915E-29	0,75216966
0,001376412	0,000783247	0,24310814	0,244514866
5,95466E-08	0,004856931	6,80561E-21	0,012751537
4,24558E-08	9,63527E-11	9,95412E-24	0,113135545
0,000213509	0,28252491	2,19281E-09	0,009016532
0,194195028	2,46615E-12	4,30754E-15	8,30775E-06
0,535388788	2,79137E-07	5,99105E-11	0,021489854
0,235870499	8,29999E-21	3,37726E-35	8,98111E-09
0,000202448	1,19647E-43	9,90033E-52	0,006049575
0,00568238	3,16765E-24	2,4988E-24	2,23305E-13
0,723312809	2,23973E-27	6,35466E-57	0,745547915
0,045575371	1,25999E-34	1,10814E-43	0,000138083
0,00265889	0,014685449	1,40315E-08	0,529498617
0,014059521	0,020052467	0,341273594	0,575180069
0,488217918	1,2798E-11	7,49709E-43	0,000325123
0,004973117	5,32068E-19	2,30636E-57	0,119843141
0,00806158	0,013925106	9,66176E-08	0,003800799
0,86970787	8,45008E-28	9,52605E-50	0,042627681
0,101377425	0,001528759	1,89365E-16	3,66864E-12
0,338742263	5,17032E-09	2,64787E-21	5,66417E-08
6,427E-05	5,02509E-06	0,000333079	0,000671685
0,02028971	5,56882E-21	1,21867E-40	0,84739797
2,80185E-15	7,97404E-18	0,440142587	0,000279316
0,003611846	0,166706591	0,4192598	0,182229882
0,052970455	0,070711202	2,02022E-16	0,03410272
2,15343E-05	3,44999E-26	4,0063E-19	0,04898707
5,84685E-08	8,49985E-25	1,95967E-26	0,600319336
0,126821693	1,90312E-07	9,85961E-11	0,034577031
0,388649757	5,69466E-25	5,05039E-26	1,21696E-13
2,28942E-06	3,08769E-24	2,00502E-16	0,019035254
0,345842632	3,21733E-07	0,000160346	0,768743898
0,001802174	1,48439E-05	0,002238567	0,004789939
3,85931E-05	5,17506E-19	2,51443E-21	0,023109375
0,015130056	2,39582E-05	0,164965424	0,033627439
1,57612E-05	1,17845E-15	2,74196E-11	1,19198E-09
0,112442724	1,19253E-14	0,486462245	0,198629844
0,187366086	1,2008E-07	6,3369E-05	5,58309E-07
0,713723211	1,34252E-14	1,11664E-12	0,388686822
8,28236E-05	0,097731253	0,101237111	0,001480655
0,009677573	2,02337E-17	6,48492E-45	0,84224764
0,003398803	6,47501E-15	1,00828E-13	0,006205846
0,595968989	1,6018E-38	7,42381E-75	0,457383639
0,633635303	3,29208E-35	1,23048E-38	0,00250992
0,019402711	8,81862E-26	6,4171E-41	0,20522523
0,149182575	3,07596E-28	8,88962E-68	0,650495864
5,0571E-05	1,60916E-06	8,21036E-11	0,025757606
1,03834E-06	0,132718656	0,138200601	3,85863E-05
0,459624962	1,31643E-05	0,000496148	0,678087358
0,01498467	0,000101304	5,3391E-13	0,001010373
0,543216276	1,64298E-08	5,02722E-33	0,060962526
0,280305076	2,29685E-23	3,41475E-47	0,073979058
1,75144E-12	3,43418E-12	0,000107969	0,870708078
0,041228966	0,000620927	1,83696E-13	0,633685825
0,230103126	0,010860323	3,65611E-10	0,660336327
1,25556E-17	7,35747E-06	0,598271911	8,34174E-09
0,131616906	1,8301E-09	1,36778E-19	7,60235E-05
4,77025E-09	0,046220841	0,008266495	0,541563606
0,196365754	1,08198E-08	9,19614E-12	1,88553E-05
0,077649704	3,95599E-31	2,9344E-34	2,28923E-08
0,000507701	1,99595E-31	2,67502E-49	0,745811335
0,025967186	1,49209E-34	5,68005E-40	0,002570079
0,919676985	2,16142E-29	5,24854E-71	0,000934796
5,87823E-05	0,000347471	0,000900627	6,74199E-06
1,95905E-06	4,05745E-44	3,81125E-48	0,10721894
0,46204628	0,00122929	3,85567E-05	0,064429021
6,74652E-06	1,77611E-44	1,38167E-25	0,013491726
0,012388047	0,757023568	0,299156651	0,000121692
0,002836903	0,109506976	7,41412E-09	0,604011986
0,000730364	1,31657E-47	2,59664E-62	0,218641335
0,285688285	5,81708E-13	1,49118E-10	0,193191639
5,39271E-05	0,000144638	7,9666E-21	1,24713E-08
0,927726285	1,12736E-28	1,5798E-44	0,39291179
3,70772E-10	6,18172E-13	0,00163216	0,043714323
0	1,63476E-09	0,042539941	1,21066E-07
1,63476E-09	0	1,53791E-37	7,33173E-08
0,042539941	1,53791E-37	0	0,527482272
1,21066E-07	7,33173E-08	0,527482272	0

Supplementary table S5. 109 Prognostic proteins and their respective Protein Selector Set

Histone Modifiers	Cell cycle and DNA damage response	Ribosomal and transcriptional activity	Cell Metabolism	Proliferative pathways	Cell adhesion and cytoskeleton regulation	Apoptosis	Signaling regulation	T-Cell	Heatshock proteins	Cell differentiation	STAT	SRC family	Immunomodulation	SMAD family	Hippo signaling	G-protein coupled receptor
ASH2L	CDK1_2_3.pT14	EIF4E.pS209	AKR1C3	AKT3	EZR	BIRC2	KIT	CD4	HSF1	SOX2	STAT3.pS727	LCK	PEA15	SMAD2	STK4	NLN
BRD4	CDKN1B	EIF4EBP1.pS65	ASS1	MAPK14	CDH1	CASP3	SGK1	CD74	HSP90AA1_B1	GATA1	STAT3.pY705	LYN	PDL1			
EP300	CDKN1B.pS10	ETS1	DLST	MAPK14.pT180_Y182	ITGAL	CASP3.cle	IGFBP2	NOTCH3	HSPB1.pS82	PIM2	STAT1					
EZH2	CDKN1B.pT198	SOX17	EP300	MKNK1	AURORA_A_B_C.pT288_232_198	TNFRSF4	IRS1	JAG1	HSF1.pS326							
HDAC3	CHEK2	WIPI2	NDUFB4	RAF1.pS338	MAP1LC3A_B	BCL2	SGK3	NOTCH1								
JMJD6	SPARC	FOS	PKM2	RHEB	RAB11	PDL1	GAB2	NOTCH1.cle								
MEN1	UGT1A1	PAX6	PPARA	TSC2	TUBA1A	PEA15										
SMARCB1	WEE1	PDCD4	SCD	TYRO3	LMNB1	LMNB1										
BMI1	BABAM1.pS29	EEF2K	ASNS	SMAD2												
CBX7	FOXM1	EIF4E	FASN	BRAF.pS445												
H3K27Ac	MECOM	ARID1A	G6PD	INPP4B												
MECOM	AURORA_A_B_C.pT288_232_198	EIF2AK2	GSKA_B	MTOR												
SUZ12	MAP1LC3A_B	EIF4G1	HK2	PTEN												
SMARCA2	TP53BP1	SPI1	ADM													
ARID1A	XPF															
H3K27Me3	WEE1.pS642															

Color Legend	
Protein Selector	Color
PS1	
PS2	
PS3	
PS1 and PS3	
PS2 and PS3	

Supplementary Table S6. Expanded Demographic, Clinical and Molecular Features

Variable	Patient Cluster						Overall
	**C1, N = 91[1]**	**C2, N = 69[1]**	**C3, N = 113[1]**	**C4, N = 85[1]**	**C5, N = 61[1]**	**p-value[2]**	**N = 419[1]**
**Age (years)**	61.1(13.5)	60.7(16.4)	58.3(13.7)	53.8(15.8)	56.5(15.3)	**0.004**	58.1(15.0)
**Age group (years)**						**<0.001**	
≤ 40	9/91(9.9%)	12/69(17%)	10/113(8.8%)	18/85(21%)	14/61(23%)		63/419(15%)
41-55	14/91(15%)	7/69(10%)	33/113(29%)	25/85(29%)	14/61(23%)		93/419(22%)
56-70	47/91(52%)	28/69(41%)	46/113(41%)	31/85(36%)	19/61(31%)		171/419(41%)
> 70	21/91(23%)	22/69(32%)	24/113(21%)	11/85(13%)	14/61(23%)		92/419(22%)
**Gender**						0.22	
female	39/91(43%)	37/69(54%)	42/113(37%)	42/85(49%)	27/61(44%)		187/419(45%)
male	52/91(57%)	32/69(46%)	71/113(63%)	43/85(51%)	34/61(56%)		232/419(55%)
**Race**						0.25	
white	72/91(79%)	57/69(83%)	87/113(77%)	59/85(69%)	50/60(83%)		325/418(78%)
black	3/91(3.3%)	2/69(2.9%)	6/113(5.3%)	8/85(9.4%)	3/60(5.0%)		22/418(5.3%)
hispanic	9/91(9.9%)	4/69(5.8%)	12/113(11%)	7/85(8.2%)	4/60(6.7%)		36/418(8.6%)
asian	5/91(5.5%)	4/69(5.8%)	6/113(5.3%)	1/85(1.2%)	1/60(1.7%)		17/418(4.1%)
other	1/91(1.1%)	0/69(0%)	0/113(0%)	3/85(3.5%)	1/60(1.7%)		5/418(1.2%)
unknown	1/91(1.1%)	2/69(2.9%)	2/113(1.8%)	7/85(8.2%)	1/60(1.7%)		13/418(3.1%)
N/A	0	0	0	0	1		1
**White Blood Cell count (K/uL)**	3.8(7.0)	25.3(34.8)	18.4(40.4)	44.1(57.2)	26.7(43.8)	**<0.001**	22.7(41.9)
N/A	4	4	4	4	2		18
**Blasts (%)**	10.4(20.0)	26.7(28.7)	26.8(25.7)	58.7(28.2)	41.5(31.1)	**<0.001**	32.0(31.1)
N/A	6	3	5	3	1		18
**Hemoglobin (g/dl)**	9.4(1.6)	9.4(1.8)	9.5(1.8)	9.0(1.7)	8.8(1.5)	0.056	9.3(1.7)
N/A	5	4	4	4	2		19
**Platelets (K/uL)**	69.9(118.5)	101.6(153.0)	66.8(69.8)	40.9(40.3)	77.4(86.4)	**<0.001**	69.5(99.2)
N/A	3	3	4	3	0		13
**Serum B2M (ug/mL)**	2.6(1.1)	3.5(2.9)	3.1(1.8)	3.0(2.8)	3.1(1.3)	0.55	3.0(2.1)
N/A	59	49	72	42	36		258
**AML group**						**0.001**	
primary	43/91(47%)	34/69(49%)	52/113(46%)	62/85(73%)	35/61(57%)		226/419(54%)
secondary	48/91(53%)	35/69(51%)	61/113(54%)	23/85(27%)	26/61(43%)		193/419(46%)
**Cytogenetic Risk**						**<0.001**	
favorable	3/83(3.6%)	7/66(11%)	8/110(7.3%)	10/80(12%)	5/60(8.3%)		33/399(8.3%)
intermediary	53/83(64%)	34/66(52%)	63/110(57%)	51/80(64%)	17/60(28%)		218/399(55%)
unfavorable	27/83(33%)	25/66(38%)	39/110(35%)	19/80(24%)	38/60(63%)		148/399(37%)
N/A	8	3	3	5	1		20
**Cytogenetic Prognosis**						**<0.001**	
favorable	56/83(67%)	41/66(62%)	71/110(65%)	61/80(76%)	22/60(37%)		251/399(63%)
unfavorable	27/83(33%)	25/66(38%)	39/110(35%)	19/80(24%)	38/60(63%)		148/399(37%)
N/A	8	3	3	5	1		20
**Complex Karyotype**						**0.002**	
no	63/83(76%)	49/66(74%)	78/110(71%)	67/80(84%)	32/60(53%)		289/399(72%)
yes	20/83(24%)	17/66(26%)	32/110(29%)	13/80(16%)	28/60(47%)		110/399(28%)
N/A	8	3	3	5	1		20
**Diploid Karyotype**						0.17	
no	31/86(36%)	17/66(26%)	39/111(35%)	26/82(32%)	28/60(47%)		141/405(35%)
yes	55/86(64%)	49/66(74%)	72/111(65%)	56/82(68%)	32/60(53%)		264/405(65%)
N/A	5	3	2	3	1		14
**-5/5q-**						**0.002**	
no	70/78(90%)	58/66(88%)	95/108(88%)	73/79(92%)	40/59(68%)		336/390(86%)
yes	8/78(10%)	8/66(12%)	13/108(12%)	6/79(7.6%)	19/59(32%)		54/390(14%)
N/A	13	3	5	6	2		29

**-7/7q-**						**<0.001**	
no	64/78(82%)	58/66(88%)	95/108(88%)	77/79(97%)	38/59(64%)		332/390(85%)
yes	14/78(18%)	8/66(12%)	13/108(12%)	2/79(2.5%)	21/59(36%)		58/390(15%)
N/A	13	3	5	6	2		29
**t(8;21)**						0.32	
no	76/78(97%)	65/66(98%)	100/107(93%)	73/79(92%)	55/59(93%)		369/389(95%)
yes	2/78(2.6%)	1/66(1.5%)	7/107(6.5%)	6/79(7.6%)	4/59(6.8%)		20/389(5.1%)
N/A	13	3	6	6	2		30
**t(9;11)**						0.33	
no	76/78(97%)	66/66(100%)	104/108(96%)	76/79(96%)	59/59(100%)		381/390(98%)
yes	2/78(2.6%)	0/66(0%)	4/108(3.7%)	3/79(3.8%)	0/59(0%)		9/390(2.3%)
N/A	13	3	5	6	2		29
**t(6;9)**						0.14	
no	78/78(100%)	66/66(100%)	108/108(100%)	77/79(97%)	58/59(98%)		387/390(99%)
yes	0/78(0%)	0/66(0%)	0/108(0%)	2/79(2.5%)	1/59(1.7%)		3/390(0.8%)
N/A	13	3	5	6	2		29
**t(15;17)**							
no	78/78(100%)	66/66(100%)	107/107(100%)	79/79(100%)	59/59(100%)		389/389(100%)
yes	0/78(0%)	0/66(0%)	0/107(0%)	0/79(0%)	0/59(0%)		0/389(0%)
N/A	13	3	6	6	2		30
**t(11q23)**						0.98	
no	76/78(97%)	64/66(97%)	104/108(96%)	76/79(96%)	58/59(98%)		378/390(97%)
yes	2/78(2.6%)	2/66(3.0%)	4/108(3.7%)	3/79(3.8%)	1/59(1.7%)		12/390(3.1%)
N/A	13	3	5	6	2		29
**Inv 16**						**0.001**	
no	77/78(99%)	57/66(86%)	105/107(98%)	71/79(90%)	58/59(98%)		368/389(95%)
yes	1/78(1.3%)	9/66(14%)	2/107(1.9%)	8/79(10%)	1/59(1.7%)		21/389(5.4%)
N/A	13	3	6	6	2		30
**Del 12**						0.15	
no	73/78(94%)	63/66(95%)	107/108(99%)	78/79(99%)	58/59(98%)		379/390(97%)
yes	5/78(6.4%)	3/66(4.5%)	1/108(0.9%)	1/79(1.3%)	1/59(1.7%)		11/390(2.8%)
N/A	13	3	5	6	2		29
**Trisomy 6**						0.54	
no	76/78(97%)	66/66(100%)	106/108(98%)	79/79(100%)	58/59(98%)		385/390(99%)
yes	2/78(2.6%)	0/66(0%)	2/108(1.9%)	0/79(0%)	1/59(1.7%)		5/390(1.3%)
N/A	13	3	5	6	2		29
**Trisomy 8**						0.16	
no	66/78(85%)	55/66(83%)	96/108(89%)	75/79(95%)	51/59(86%)		343/390(88%)
yes	12/78(15%)	11/66(17%)	12/108(11%)	4/79(5.1%)	8/59(14%)		47/390(12%)
N/A	13	3	5	6	2		29
**non-t(9;11)/t(11q23)**						0.54	
no	78/78(100%)	64/66(97%)	107/108(99%)	77/79(97%)	58/59(98%)		384/390(98%)
yes	0/78(0%)	2/66(3.0%)	1/108(0.9%)	2/79(2.5%)	1/59(1.7%)		6/390(1.5%)
N/A	13	3	5	6	2		29
**t(9;22)Ph+**						>0.99	
no	78/78(100%)	66/66(100%)	107/108(99%)	79/79(100%)	59/59(100%)		389/390(100%)
yes	0/78(0%)	0/66(0%)	1/108(0.9%)	0/79(0%)	0/59(0%)		1/390(0.3%)
N/A	13	3	5	6	2		29
**ASLX1 Mut**						**0.012**	
no	38/53(72%)	35/48(73%)	61/74(82%)	60/67(90%)	33/35(94%)		227/277(82%)
yes	15/53(28%)	13/48(27%)	13/74(18%)	7/67(10%)	2/35(5.7%)		50/277(18%)
N/A	38	21	39	18	26		142
**CEBPA Mut**						**0.003**	
no	64/73(88%)	50/52(96%)	72/81(89%)	50/68(74%)	46/49(94%)		282/323(87%)

yes	9/73(12%)	2/52(3.8%)	9/81(11%)	18/68(26%)	3/49(6.1%)		41/323(13%)
N/A	18	17	32	17	12		96
**DNMT3 Mut**						**0.014**	
no	59/73(81%)	33/56(59%)	73/90(81%)	50/71(70%)	40/49(82%)		255/339(75%)
yes	14/73(19%)	23/56(41%)	17/90(19%)	21/71(30%)	9/49(18%)		84/339(25%)
N/A	18	13	23	14	12		80
**EZH2 Mut**						**0.031**	
no	63/67(94%)	51/51(100%)	80/86(93%)	70/70(100%)	39/42(93%)		303/316(96%)
yes	4/67(6.0%)	0/51(0%)	6/86(7.0%)	0/70(0%)	3/42(7.1%)		13/316(4.1%)
N/A	24	18	27	15	19		103
**FLT3 Mut**						**<0.001**	
no	73/77(95%)	43/56(77%)	79/97(81%)	44/77(57%)	44/54(81%)		283/361(78%)
yes	4/77(5.2%)	13/56(23%)	18/97(19%)	33/77(43%)	10/54(19%)		78/361(22%)
N/A	14	13	16	8	7		58
**FLT3_D835 Mut**						**<0.001**	
no	76/76(100%)	52/56(93%)	95/97(98%)	64/77(83%)	52/54(96%)		339/360(94%)
yes	0/76(0%)	4/56(7.1%)	2/97(2.1%)	13/77(17%)	2/54(3.7%)		21/360(5.8%)
N/A	15	13	16	8	7		59
**FLT3_ITD Mut**						**0.002**	
no	73/77(95%)	46/56(82%)	81/97(84%)	53/76(70%)	44/54(81%)		297/360(82%)
yes	4/77(5.2%)	10/56(18%)	16/97(16%)	23/76(30%)	10/54(19%)		63/360(18%)
N/A	14	13	16	9	7		59
**GATA2 Mut**						0.20	
no	52/53(98%)	46/48(96%)	70/74(95%)	58/66(88%)	34/35(97%)		260/276(94%)
yes	1/53(1.9%)	2/48(4.2%)	4/74(5.4%)	8/66(12%)	1/35(2.9%)		16/276(5.8%)
N/A	38	21	39	19	26		143
**IDH Mut**						0.66	
no	56/77(73%)	46/58(79%)	80/97(82%)	60/77(78%)	41/52(79%)		283/361(78%)
yes	21/77(27%)	12/58(21%)	17/97(18%)	17/77(22%)	11/52(21%)		78/361(22%)
N/A	14	11	16	8	9		58
**IDH1 Mut**						0.86	
no	65/74(88%)	51/56(91%)	89/97(92%)	70/76(92%)	46/52(88%)		321/355(90%)
yes	9/74(12%)	5/56(8.9%)	8/97(8.2%)	6/76(7.9%)	6/52(12%)		34/355(9.6%)
N/A	17	13	16	9	9		64
**IDH2 Mut**						0.84	
no	63/76(83%)	49/57(86%)	86/97(89%)	64/75(85%)	46/52(88%)		308/357(86%)
yes	13/76(17%)	8/57(14%)	11/97(11%)	11/75(15%)	6/52(12%)		49/357(14%)
N/A	15	12	16	10	9		62
**JAK2 Mut**						0.80	
no	69/72(96%)	51/52(98%)	88/91(97%)	72/74(97%)	44/47(94%)		324/336(96%)
yes	3/72(4.2%)	1/52(1.9%)	3/91(3.3%)	2/74(2.7%)	3/47(6.4%)		12/336(3.6%)
N/A	19	17	22	11	14		83
**KIT Mut**						0.57	
no	73/74(99%)	52/54(96%)	90/96(94%)	72/76(95%)	49/52(94%)		336/352(95%)
yes	1/74(1.4%)	2/54(3.7%)	6/96(6.2%)	4/76(5.3%)	3/52(5.8%)		16/352(4.5%)
N/A	17	15	17	9	9		67
**KMT2A Mut**						0.52	
no	15/15(100%)	22/22(100%)	23/23(100%)	17/18(94%)	14/14(100%)		91/92(99%)
yes	0/15(0%)	0/22(0%)	0/23(0%)	1/18(5.6%)	0/14(0%)		1/92(1.1%)
N/A	76	47	90	67	47		327
**MLL Mut**						0.31	
no	30/34(88%)	22/23(96%)	38/46(83%)	41/44(93%)	16/20(80%)		147/167(88%)
yes	4/34(12%)	1/23(4.3%)	8/46(17%)	3/44(6.8%)	4/20(20%)		20/167(12%)
N/A	57	46	67	41	41		252

**NPM1 Mut**						**<0.001**	
no	69/73(95%)	40/55(73%)	83/95(87%)	46/74(62%)	44/52(85%)		282/349(81%)
yes	4/73(5.5%)	15/55(27%)	12/95(13%)	28/74(38%)	8/52(15%)		67/349(19%)
N/A	18	14	18	11	9		70
**PTPN11 Mut**						0.19	
no	59/62(95%)	41/49(84%)	76/87(87%)	65/70(93%)	35/41(85%)		276/309(89%)
yes	3/62(4.8%)	8/49(16%)	11/87(13%)	5/70(7.1%)	6/41(15%)		33/309(11%)
N/A	29	20	26	15	20		110
**RAS Mut**						0.13	
no	66/76(87%)	42/56(75%)	72/99(73%)	56/78(72%)	39/53(74%)		275/362(76%)
yes	10/76(13%)	14/56(25%)	27/99(27%)	22/78(28%)	14/53(26%)		87/362(24%)
N/A	15	13	14	7	8		57
**RUNX1 Mut**						0.37	
no	42/54(78%)	42/48(88%)	59/73(81%)	58/65(89%)	31/35(89%)		232/275(84%)
yes	12/54(22%)	6/48(12%)	14/73(19%)	7/65(11%)	4/35(11%)		43/275(16%)
N/A	37	21	40	20	26		144
**SRSF2 Mut**						0.28	
no	11/17(65%)	18/23(78%)	18/21(86%)	17/19(89%)	13/14(93%)		77/94(82%)
yes	6/17(35%)	5/23(22%)	3/21(14%)	2/19(11%)	1/14(7.1%)		17/94(18%)
N/A	74	46	92	66	47		325
**TET2 Mut**						0.65	
no	44/54(81%)	44/49(90%)	61/75(81%)	55/66(83%)	32/36(89%)		236/280(84%)
yes	10/54(19%)	5/49(10%)	14/75(19%)	11/66(17%)	4/36(11%)		44/280(16%)
N/A	37	20	38	19	25		139
**TP53 Mut**						**0.004**	
no	61/75(81%)	47/57(82%)	86/101(85%)	69/76(91%)	34/53(64%)		297/362(82%)
yes	14/75(19%)	10/57(18%)	15/101(15%)	7/76(9.2%)	19/53(36%)		65/362(18%)
N/A	16	12	12	9	8		57
**U2AF Mut**						0.38	
no	15/15(100%)	21/22(95%)	17/21(81%)	17/19(89%)	13/14(93%)		83/91(91%)
yes	0/15(0%)	1/22(4.5%)	4/21(19%)	2/19(11%)	1/14(7.1%)		8/91(8.8%)
N/A	76	47	92	66	47		328
**WT1 Mut**						0.29	
no	51/52(98%)	43/46(93%)	71/74(96%)	57/64(89%)	33/34(97%)		255/270(94%)
yes	1/52(1.9%)	3/46(6.5%)	3/74(4.1%)	7/64(11%)	1/34(2.9%)		15/270(5.6%)
N/A	39	23	39	21	27		149

[1] Mean(SD); n/N(%)

[2] Kruskal-Wallis rank sum test; Fisher's Exact Test for Count Data with simulated p-value (based on 10000 replicates)

Supplementary Table S7. Expanded Univariate Cox Proportional Hazards of Overall Survival (OS) and Complete Remission Duration (CRD)

Variable	Univariate OS (N=419)			Univariate CRD (N=274)		
	HR*1*	95% CI*1*	p-value	HR*1*	95% CI*1*	p-value
**Cluster**						
Group1	1.00	—		1.00	—	
Group2	2.49	1.85, 3.37	**<0.001**	2.22	1.44, 3.43	**<0.001**
Group3	3.99	2.81, 5.68	**<0.001**	3.88	2.27, 6.64	**<0.001**
**Age (years)**	1.04	1.03, 1.05	**<0.001**	1.04	1.02, 1.05	**<0.001**
**Gender**						
female	1.00	—		1.00	—	
male	1.07	0.85, 1.35	0.58	1.29	0.90, 1.86	0.17
**White (race)**						
no	1.00	—		1.00	—	
yes	1.49	1.11, 2.01	**0.008**	1.09	0.72, 1.66	0.69
**Black (race)**						
no	1.00	—		1.00	—	
yes	1.03	0.62, 1.71	0.91	1.88	1.01, 3.50	**0.046**
**Hispanic (race)**						
no	1.00	—		1.00	—	
yes	0.64	0.41, 1.01	0.055	0.74	0.40, 1.37	0.34
**Asian (race)**						
no	1.00	—		1.00	—	
yes	0.42	0.20, 0.90	**0.025**	1.03	0.45, 2.34	0.95
**Other (race)**						
no	1.00	—		1.00	—	
yes	0.82	0.26, 2.56	0.73	0.72	0.10, 5.19	0.75
**White Blood Cell count (K/uL)**	1.00	1.00, 1.00	0.74	1.00	1.00, 1.01	0.63
**Blasts (%)**	1.00	0.99, 1.00	**0.014**	1.00	0.99, 1.01	0.82
**Hemoglobin (g/dl)**	0.92	0.85, 0.98	**0.016**	0.93	0.83, 1.04	0.22
**Platelets (K/uL)**	1.00	1.00, 1.00	0.083	1.00	1.0, 1.00	0.10
**Serum B2M (ug/mL)**	1.13	1.05, 1.21	**0.001**	1.07	0.95, 1.21	0.25
**AML group**						
primary	1.00	—		1.00	—	
secondary	2.84	2.24, 3.60	**<0.001**	2.24	1.55, 3.23	**<0.001**
**Complex Karyotype**						
no	1.00	—		1.00	—	
yes	2.51	1.95, 3.22	**<0.001**	2.29	1.53, 3.44	**<0.001**
**Diploid Karyotype**						
no	1.00	—		1.00	—	
yes	0.80	0.63, 1.03	0.080	0.85	0.58, 1.24	0.40
**-5/5q-**						
no	1.00	—		1.00	—	
yes	2.55	1.86, 3.49	**<0.001**	2.37	1.39, 4.04	**0.001**
**-7/7q-**						
no	1.00	—		1.00	—	
yes	2.04	1.50, 2.78	**<0.001**	1.36	0.71, 2.61	0.35
**t(8;21)**						
no	1.00	—		1.00	—	
yes	0.45	0.23, 0.87	**0.017**	0.82	0.40, 1.69	0.59
**t(9;11)**						
no	1.00	—		1.00	—	
yes	0.99	0.47, 2.10	0.98	2.26	0.92, 5.55	0.076
**t(6;9)**						
no	1.00	—		1.00	—	
yes	0.34	0.05, 2.39	0.28	0.00	0.00, Inf	>0.99

**t(11q23)**						
no	1.00	—		1.00	—	
yes	1.25	0.66, 2.35	0.49	1.99	0.81, 4.88	0.14
**Inv 16**						
no	1.00	—		1.00	—	
yes	0.16	0.06, 0.44	**<0.001**	0.29	0.11, 0.80	**0.016**
**Del 12**						
no	1.00	—		1.00	—	
yes	2.43	1.29, 4.59	**0.006**	2.01	0.64, 6.33	0.23
**Trisomy 8**						
no	1.00	—		1.00	—	
yes	0.83	0.57, 1.22	0.35	0.80	0.43, 1.48	0.47
**Trisomy 6**						
no	1.00	—		1.00	—	
yes	1.04	0.33, 3.25	0.94	0.73	0.10, 5.24	0.76
**non-t(9;11)/t(11q23)**						
no	1.00	—		1.00	—	
yes	1.35	0.55, 3.26	0.51	1.13	0.28, 4.56	0.87
**t(9;22)Ph+**						
no	1.00	—		1.00	—	
yes	1.59	0.22, 11.4	0.64	0.00	0.00, Inf	>0.99
**ASLX1 Mut**						
no	1.00	—		1.00	—	
yes	1.43	1.01, 2.02	**0.042**	1.30	0.74, 2.28	0.37
**CEBPA Mut**						
no	1.00	—		1.00	—	
yes	0.56	0.35, 0.87	**0.011**	0.60	0.31, 1.15	0.12
**DNMT3 Mut**						
no	1.00	—		1.00	—	
yes	0.78	0.57, 1.06	0.11	1.07	0.69, 1.64	0.77
**EZH2 Mut**						
no	1.00	—		1.00	—	
yes	1.24	0.64, 2.42	0.52	1.16	0.37, 3.67	0.80
**FLT3 Mut**						
no	1.00	—		1.00	—	
yes	0.59	0.42, 0.83	**0.002**	0.60	0.36, 0.98	**0.042**
**FLT3_D835 Mut**						
no	1.00	—		1.00	—	
yes	0.51	0.27, 0.97	**0.039**	0.61	0.27, 1.39	0.24
**FLT3_ITD Mut**						
no	1.00	—		1.00	—	
yes	0.62	0.43, 0.90	**0.012**	0.65	0.37, 1.12	0.12
**GATA2 Mut**						
no	1.00	—		1.00	—	
yes	0.78	0.40, 1.53	0.48	1.56	0.68, 3.59	0.29
**IDH Mut**						
no	1.00	—		1.00	—	
yes	0.82	0.60, 1.11	0.20	0.91	0.58, 1.43	0.68
**IDH1 Mut**						
no	1.00	—		1.00	—	
yes	1.32	0.88, 1.96	0.18	1.23	0.66, 2.31	0.51
**IDH2 Mut**						
no	1.00	—		1.00	—	
yes	0.61	0.41, 0.90	**0.014**	0.73	0.41, 1.28	0.27
**JAK2 Mut**						
no	1.00	—		1.00	—	

yes	1.90	1.03, 3.48	**0.039**	1.26	0.40, 3.98	0.69
**KIT Mut**						
no	1.00	—		1.00	—	
yes	0.53	0.26, 1.07	0.079	0.96	0.42, 2.18	0.92
**KMT2A Mut**						
no	1.00	—		1.00	—	
yes	0.88	0.12, 6.38	0.90	0.00	0.00, Inf	>0.99
**MLL Mut**						
no	1.00	—		1.00	—	
yes	1.96	1.16, 3.30	**0.012**	2.30	0.90, 5.90	0.083
**NPM1 Mut**						
no	1.00	—		1.00	—	
yes	0.54	0.37, 0.78	**0.001**	0.61	0.37, 1.01	0.054
**PTPN11 Mut**						
no	1.00	—		1.00	—	
yes	1.82	1.20, 2.75	**0.004**	1.74	0.90, 3.37	0.10
**RAS Mut**						
no	1.00	—		1.00	—	
yes	1.09	0.81, 1.46	0.58	1.17	0.75, 1.82	0.49
**RUNX1 Mut**						
no	1.00	—		1.00	—	
yes	1.21	0.83, 1.76	0.32	2.19	1.24, 3.84	**0.006**
**SRSF2 Mut**						
no	1.00	—		1.00	—	
yes	0.52	0.26, 1.05	0.069	0.59	0.21, 1.71	0.33
**TET2 Mut**						
no	1.00	—		1.00	—	
yes	0.97	0.66, 1.43	0.88	1.07	0.60, 1.90	0.82
**TP53 Mut**						
no	1.00	—		1.00	—	
yes	3.40	2.53, 4.56	**<0.001**	2.82	1.74, 4.57	**<0.001**
**U2AF Mut**						
no	1.00	—		1.00	—	
yes	1.81	0.86, 3.80	0.12	1.11	0.15, 8.26	0.92
**WT1 Mut**						
no	1.00	—		1.00	—	
yes	1.36	0.74, 2.51	0.32	2.37	0.86, 6.50	0.10

1 HR = Hazard Ratio, CI = Confidence Interval

Supplementary Table S8. Calculations for determining the accuracy of the final Protein Classifier (PC) model

		Predicted					
		C1	C2+C4	C3	C5	**True positive**	**False Negative**
Actual	C1 =91	79	3	9	0	79	12
	C2+C4 =154	1	124	26	3	124	30
	C3 =113	10	11	86	6	86	27
	C5 =61		13	8	40	40	21
**True positive**		79	124	86	40	329	
**False postive**		11	27	43	9		

Summary		
Correct	329	0,785203
Error	77	0,214797

Consequence of Error	Number affected	Assessment of severity of Error
Get Wrong TX CC> VH, VH>CC	23	Major Error - Therapy rec changed to incorrect
Wrong Group, Still Get CC	37	No consequence for induction, changes maintenance rec
C5> CC, No Rec > CC	21	Move from No Rec to CC, not harmed.
C2,3,4 >C5 Rec CC> no Rec	9	Move from Chemo to no rec, likely get CC. May be harmed

	Model 1	Model 2	Model 3	Overall C1,2,3,4 vs. 5	Overall C1,2+4, vs. 3
True Positive	79	124	86	326	203
False Negative	12	29	6	23	38
False Postiive	11	24	8	32	22
True Negative	317	140	40	40	86
Sensitivity	86,81	81,05	91,49	86,01	84,23
Specificity	96,65	85,37	86,97	55,56	79,63
PPV	87	85	90	91,06	90,22
NPV	96,35	81,05	86,97	63,49	69,35
Accuracy	94,51	83,28	90	86,94	82,8

Supplementary Table S9. p-values, adjusted p-values by the False Discovery Rate (FDR) and Log2-Fold-change (LFC) values for each comparison

Protein	p-value C1 vs rest	p-value C2 vs rest	p-value C3 vs rest	p-value C4 vs rest	p-value C5 vs rest	adj. p-value C1 vs rest	adj. p-value C2 vs rest	adj. p-value C3 vs rest	adj. p-value C4 vs rest	adj. p-value C5 vs rest	Log2-Fold-change C1 vs rest	Log2-Fold-change C2 vs rest	Log2-Fold-change C3 vs rest	Log2-Fold-change C4 vs rest	Log2-Fold-change C5 vs rest
ABL1	0,355	0,645	0,0803	0,424	0,00431	0,394337838	0,728282967	0,181693443	0,469556452	0,013219478	0,043041422	0,021380419	0,060381049	-0,042217646	-0,123195092
ABL1.pY412	2,76E-25	0,207	0,0442	4,15E-13	0,000000368	3,06584E-24	0,303846429	0,12030596	1,98331E-12	5,60178E-06	0,542361155	-0,069075695	0,050984567	-0,355609275	-0,283332483
ACACA	7,42E-14	0,415	0,95	0,00000652	0,00472	2,56271E-13	0,529704969	0,959336609	1,30718E-05	0,013787447	-0,530749293	0,041118815	-0,011087078	0,307895677	0,297234006
ACACA.pS79	0,672	0,0193	0,858	0,0466	0,939	0,702778626	0,045851445	0,908860825	0,063842	0,950248157	-0,0236124	-0,085457656	-0,001491411	0,094654641	0,006086184
ADAR	0,000359	0,385	0,0002	0,384	0,162	0,0005385	0,499164038	0,002348571	0,430038147	0,258069767	0,176961991	0,031654811	-0,151660287	0,030429041	-0,076298024
ADM	7,12E-20	1,75E-08	0,0000204	1,71E-14	0,175	4,18046E-19	5,65915E-07	0,000519794	1,01857E-13	0,274522901	0,84244005	-0,512126742	0,257059188	-0,62657477	-0,17755402
AIFM1	0,000000362	0,685	0,0655	5,26E-11	0,722	6,67184E-07	0,75276738	0,156514535	2,00172E-10	0,811893443	-0,247041675	0,003981558	-0,091290906	0,341442277	0,033915926
AKR1C3	2,94E-13	0,00729	0,722	0,013	0,000882	9,74468E-13	0,02109993	0,819729282	0,019429091	0,00351326	0,373106368	-0,144337791	0,011558949	-0,124202035	-0,207176059
AKT1	2,02E-08	0,00456	0,177	0,00000179	0,00036	4,06971E-08	0,014306565	0,333701835	3,89254E-06	0,001662472	-0,262302809	0,12845738	0,075348299	0,197931998	-0,160166424
AKT1_2_3	1,59E-11	0,000281	0,0861	1,51E-13	0,0277	4,27118E-11	0,001332207	0,190253226	7,75763E-13	0,060236508	-0,344036136	0,197846924	-0,07326348	0,378766138	-0,12496926
AKT1_2_3.pS473	0,0668	0,954	0,0316	0,0317	0,0195	0,0822	0,973581683	0,092768571	0,044772165	0,044525	0,062601528	0,040269264	0,114092307	-0,112988987	-0,163859309
AKT1_2_3.pT308	0,00155	0,0227	2,08E-08	0,00000456	0,602	0,002211979	0,053009659	4,2744E-06	9,3708E-06	0,714525862	-0,57484788	0,452204931	0,914402849	-0,807209235	-0,112841886
AKT1S1	0,001	0,701	0,0748	4,82E-11	0,315	0,001457447	0,762198413	0,174675	1,85142E-10	0,441755102	0,264604465	0,162239214	0,149659646	-0,69572918	0,126423164
AKT1S1.pT246	5,03E-10	0,2	0,535	0,00000634	0,12	1,18812E-09	0,297826087	0,671634146	1,27732E-05	0,202962963	0,228660536	-0,046793202	0,0183656	-0,15705615	-0,085686669
AKT2	5,97E-10	0,99	0,734	0,237	4,38E-08	1,4021E-09	0,992414634	0,828774725	0,280711816	1,20012E-06	0,21006976	0,008857546	-0,00930354	-0,046131043	-0,222214207
AKT3	7,81E-19	0,0598	0,00111	1,36E-12	0,0000107	4,33772E-18	0,109722321	0,007732373	6,21067E-12	8,14389E-05	0,338551191	-0,082060457	0,159066459	-0,287077546	-0,25063579
AMBRA1.pS52	0,00773	0,431	0,0268	2,15E-08	0,73	0,010315032	0,540064024	0,079817391	5,73799E-08	0,817520436	-0,13844416	-0,048179264	-0,086518687	0,26190586	0,039012103
ANXA1	2,12E-08	0,234	0,592	0,000000299	0,223	4,25034E-08	0,328238908	0,715623529	7,22876E-07	0,336959559	-0,632767189	0,094076408	0,106476416	0,505717819	-0,065215706
ANXA7	2,01E-09	0,000632	0,147	0,359	0,00839	4,46546E-09	0,002677856	0,284985849	0,405354396	0,022391494	0,536596092	-0,211823842	-0,152791297	0,013123217	-0,274298245
AR	0,000377	0,229	0,226	0,00462	0,237	0,000563444	0,32567128	0,393584746	0,007446353	0,352923913	0,043960258	-0,025307975	0,069611547	-0,087098613	-0,029088592
ARAF	9,48E-14	0,000124	0,0133	7,5E-11	0,716	3,2469E-13	0,00067952	0,045176033	2,80227E-10	0,808450549	-0,332767655	0,183908038	-0,10089706	0,306967617	0,01213837
ARID1A	1,52E-28	0,762	0,116	3,41E-25	0,000609	3,1236E-27	0,819848168	0,240787879	1,00108E-23	0,002554071	-0,848076789	0,041623679	-0,041304673	0,676998863	0,298398177
ASH2L	3,34E-36	0,641	0,208	2,85E-24	0,000000228	4,5758E-34	0,727765193	0,368482759	6,5075E-23	4,07426E-06	-1,199843889	0,010610282	0,034735139	0,833635434	0,489455101
ASNS	0,000236	0,00843	0,00179	0,00000262	0,919	0,000359244	0,023731027	0,010217917	5,52215E-06	0,941917706	0,21118864	-0,22101673	0,250260433	-0,328190049	-0,013838001
ASS1	5,84E-11	0,00793	0,000626	3,19E-08	0,00423	1,50015E-10	0,022477448	0,005044824	8,29804E-08	0,013170682	0,243185236	-0,110508545	0,117527927	-0,210812451	-0,122205639
ATF3	3,77E-21	0,000632	0,102	0,000000419	0,000191	2,49915E-20	0,002677856	0,214984615	1,00122E-06	0,000957329	0,344177397	-0,124015503	0,054512963	-0,189270587	-0,173531908
ATG3	0,00428	0,024	0,0259	7,72E-08	0,0168	0,00584412	0,055728814	0,078271324	1,94658E-07	0,039456	0,229878243	0,172097347	0,066294699	-0,326601063	-0,184892877
ATG4B	2,64E-08	0,102	0,864	2,52E-08	0,0741	5,26718E-08	0,170414634	0,912863753	6,68206E-08	0,135960268	0,79985301	-0,23032485	-0,135048372	-0,535529167	0,071513331
ATG7	0,643	0,0378	0,585	0,341	0,214	0,679365039	0,080293299	0,714676558	0,388229917	0,326966543	-0,028836304	0,264451067	-0,005911338	-0,060939455	-0,164452508
AURKA	0,00000248	0,000751	0,0000123	0,285	0,00000184	4,37459E-06	0,003026088	0,000388869	0,331827195	2,04389E-05	-0,307197303	-0,231513085	0,281894905	-0,091122764	0,348011694
AURORA_A_B_C.pT288_232_198	0,0000265	0,000289	0,00735	0,0000038	0,426	4,32202E-05	0,001349761	0,031142784	7,88788E-06	0,550584906	0,186327424	-0,169916088	0,114182598	-0,238538957	0,062546372
AXL	4,47E-09	0,0546	0,00153	4,44E-13	0,571	9,46995E-09	0,102468493	0,009385522	2,09752E-12	0,688214076	0,259475847	-0,069556578	0,186255718	-0,415180159	-0,032898125
BABAM1.pS29	7E-23	0,000000694	0,000853	1,16E-09	0,000384	5,32778E-22	1,14094E-05	0,006374236	3,58466E-09	0,0017536	-0,546462674	0,353520187	-0,192553242	0,354149762	0,200435006
BAK1	0,0103	0,511	0,0103	0,782	0,435	0,01352492	0,614096491	0,039936792	0,803505	0,556962617	0,124394861	0,041195999	-0,118533554	0,015288715	-0,047770611
BAP1	7,34E-08	0,211	0,583	4,6E-09	0,815	1,42299E-07	0,306434629	0,714676558	1,3221E-08	0,867784974	-0,249608995	-0,023563392	0,038848961	0,224142346	0,014317457
BAX	7,6E-13	0,00896	0,945	0,00000267	0,715	2,36636E-12	0,025051429	0,959336609	5,59883E-06	0,808450549	-0,426231677	0,152713539	0,025168282	0,281720138	0,007593532
BBC3	0,00000597	0,699	0,000399	0,311	0,113	1,01812E-05	0,762198413	0,003999732	0,358042017	0,194322176	-0,16962256	-0,002513375	0,127941796	-0,023241861	0,062251908
BCL2	0,458	0,129	0,0000725	6,36E-09	0,368	0,49930504	0,206299611	0,001146058	1,79038E-08	0,491064935	0,07862358	-0,223135138	-0,441033369	0,78922504	-0,188391339
BCL2.pS70	2,85E-13	0,000000974	0,937	0,0357	0,275	9,52317E-13	1,48264E-05	0,957640199	0,050077474	0,403660714	0,297190416	-0,191951976	-0,012123446	-0,088339032	-0,059884134
BCL2A1	0,00122	0,535	0,9	0,134	0,0104	0,001753217	0,633674352	0,936455696	0,166890909	0,026385185	0,251295429	0,06019926	-0,008599486	-0,141056102	-0,213048005
BCL2L1	7,7E-09	0,126	0,925	0,000000958	0,746	1,58235E-08	0,20388189	0,9504375	2,1634E-06	0,829775676	0,500791006	-0,140129618	0,015452949	-0,4155693	-0,013728333
BCL2L11	0,381	0,367	0,756	0,171	0,273	0,422078167	0,488145631	0,84204878	0,210422156	0,40216129	-0,188037223	0,085874284	0,011043394	0,11529885	-0,005335734
BECN1	0,000857	0,000188	0,0000664	0,0000068	0,888	0,001257954	0,000942293	0,001112988	1,3567E-05	0,921135678	-0,124584756	0,18017951	-0,112930341	0,13534885	-0,026118594
BID	4,29E-17	0,000126	0,781	0,00028	0,194	2,02666E-16	0,000681395	0,858264706	0,00050696	0,300883019	-0,55243732	0,313574138	0,004384943	0,24313603	0,085267847
BIRC2	2,66E-15	0,0841	0,604	0,000487	0,0000411	1,03138E-14	0,145231513	0,725859649	0,000851732	0,000272453	0,314492076	-0,040051481	0,000820321	-0,148683917	-0,193559156
BIRC5	2,6E-12	0,0541	0,000192	0,856	0,000000112	7,8E-12	0,101995872	0,002345118	0,866541872	2,3016E-06	-0,652923302	-0,246418848	0,330573944	0,001324859	0,639768813
BMI1	0,511	0,0429	0,000194	0,026	0,429	0,55123622	0,085591748	0,002345118	0,037494737	0,552724138	-0,023332851	-0,092391979	0,232566304	-0,146607044	-0,043584507
BRAF	0,000000125	0,7	0,333	0,209	0,0000831	2,34589E-07	0,762198413	0,48705694	0,252644118	0,000509763	0,178335679	0,01579725	-0,031144558	-0,052451859	-0,143714724
BRAF.pS445	8,87E-18	0,303	0,348	2,97E-13	0,069	4,44582E-17	0,412360927	0,500097902	1,45318E-12	0,128904545	-0,402539157	0,033542399	-0,052917208	0,380581835	0,102115245
BRD4	8,01E-18	0,535	0,000511	7,82E-17	0,0000194	4,06433E-17	0,633674352	0,004375438	6,18081E-16	0,000139884	-0,624202804	0,019320724	-0,213240701	0,620838626	0,362324306
BTK	5,99E-28	0,105	0,0583	2,98E-20	0,00291	1,07039E-26	0,173313253	0,144345181	3,31022E-19	0,00956808	1,028478112	-0,204443438	0,045253144	-0,707250325	-0,33183589
CASP3	2,85E-09	0,0767	0,663	7,84E-10	0,212	6,19762E-09	0,133749153	0,766739496	2,5372E-09	0,325119403	-0,506086138	-0,218839544	-0,017445536	0,711408169	0,036495461
CASP3.cle	2,6E-09	0,0000266	0,00262	0,000163	0,0423	5,68404E-09	0,000198775	0,013630633	0,000300417	0,0869265	0,295778427	-0,283494983	0,035549217	-0,049548972	-0,082631934
CASP7.cle	0,00279	0,00048	0,0775	0,0703	0,985	0,003900306	0,002116213	0,178946629	0,093204194	0,987402439	0,101926132	-0,186643524	0,051272279	-0,081821072	0,092277558
CASP9	1,93E-30	0,000175	0,000161	1,43E-19	0,000101	7,21118E-29	0,000899063	0,002067844	1,46933E-18	0,000576542	0,728174951	-0,248428312	0,084164148	-0,455769355	-0,261285746
CAV1	3,83E-11	0,00327	0,33	0,000000003	0,368	9,90019E-11	0,010666429	0,48705694	8,74468E-09	0,491064935	0,451536219	-0,202426463	0,021132778	-0,340144633	0,015437448
CBL	3,65E-12	0,000000166	0,275	0,38	0,807	1,04906E-11	3,4113E-06	0,438081395	0,426721311	0,863633766	-0,582824482	0,516149971	0,100834768	0,052832512	-0,002539439
CBX7	3,32E-12	9,04E-08	0,000017	0,00000568	0,00149	9,73574E-12	2,06413E-06	0,0004658	1,15568E-05	0,00532513	0,780170723	0,326616269	-0,34805114	-0,477585665	-0,255552215
CCNB1	0,0000976	0,0456	0,242	0,234	0,0103	0,000152523	0,089672727	0,407631148	0,278765217	0,026293789	-0,570363533	0,223413808	-0,154227904	0,172015451	0,553079086
CCND1	0,0102	0,000484	0,011	0,0000261	0,0239	0,013479743	0,002116213	0,04072973	4,94336E-05	0,053096757	-0,246578807	0,304143876	-0,14615286	0,317671431	-0,180886927
CCND3	0,0186	0,0000112	0,705	0,0755	0,751	0,023814953	0,0000959	0,802645429	0,099138978	0,83197035	-0,123717107	0,263445415	0,021341782	-0,097243071	-0,029614613
CD276	0,00375	0,526	0,000677	0,000252	0,00473	0,005154682	0,628447674	0,005152722	0,00046032	0,013787447	0,10431	-0,028768917	0,08991773	-0,108986227	-0,111444812
CD4	6,27E-13	0,000000012	0,0957	8,54E-13	0,0000329	1,99765E-12	0,000000548	0,204857813	3,98857E-12	0,000229185	1,647358844	1,125106642	-0,366195102	-1,441779241	-1,041637755
CD44	0,779	0,0000566	0,000000708	0,00136	0,0483	0,796440299	0,000363478	0,000048498	0,002272195	0,095438942	-0,036188253	0,29463912	-0,225689242	0,168017224	-0,137444552
CD70	0,557	0,00264	0,0434	0,0114	0,000452	0,596164063	0,009117983	0,118916	0,017225735	0,001997548	-0,03313278	0,22431121	0,161820114	-0,18721948	-0,215606895
CD74	0,0857	0,412	0,221	0,0394	0,00612	0,10390177	0,527514019	0,386514894	0,054523232	0,016548158	-0,128141243	0,016232558	0,092737959	0,148756046	-0,183035706
CD86	0,0921	0,388	0,56	0,00618	0,00519	0,110038081	0,501471698	0,689101796	0,009806873	0,014916713	-0,06797132	0,034308765	0,024233579	0,129244015	-0,151430305
CDC25C	0,000000117	0,00000387	0,000453	0,0000105	0,469	2,20583E-07	4,81991E-05	0,0041374	2,06483E-05	0,585893617	0,266155254	-0,224382699	0,108326558	-0,187032804	-0,044015673
CDH1	1,08E-13	0,00755	0,00138	1,83E-12	0,0615	3,66843E-13	0,021548958	0,008862188	8,26516E-12	0,117020833	0,394012473	-0,175461821	0,179242786	-0,385105727	-0,127657604
CDK1	0,77	0,798	0,0346	0,908	0,0017	0,789201995	0,854109375	0,10014507	0,916923833	0,006023276	-0,02843864	0,075476518	0,093258212	-0,006119783	-0,184305486
CDK1_2_3.pT14	7,16E-15	0,308	0,241	0,00216	0,000000201	2,69978E-14	0,416407895	0,407617284	0,003579677	3,75505E-06	-0,560417083	-0,104475916	0,068303097	0,172462992	0,549153476
CDK2	0,00102	0,0861	0,339	0,127	0,0101	0,001481343	0,14744625	0,490315789	0,160606154	0,025944375	0,108866445	-0,078210905	-0,011542932	0,077546048	-0,144841704
CDK9	1,06E-23	0,871	0,00381	1,07E-23	0,00017	9,26936E-23	0,913216837	0,018208256	2,09414E-22	0,000862593	-0,824544245	0,03593463	-0,20196728	0,809293168	0,354930254
CDKN1A	1,1E-09	0,0116	0,00000133	1,52E-11	0,00419	2,5257E-09	0,03157351	6,93563E-05	6,18535E-11	0,013145725	0,22557367	-0,069063012	0,216174346	-0,32369413	-0,153413836
CDKN1B	4,13E-15	0,0399	0,49	0,00000233	0,000000368	1,58638E-14	0,0819945	0,633301887	4,98766E-06	5,60178E-06	0,733919672	0,145301556	-0,042252323	-0,428490864	-0,539848451
CDKN1B.pS10	1,27E-21	0,241	0,773	5,81E-09	6,64E-08	8,55689E-21	0,335766102	0,854040323	1,64683E-08	1,51613E-06	0,755602303	0,060311382	-0,033491293	-0,463934467	-0,443290409
CDKN1B.pT198	1,43E-28	0,0141	0,00000435	2,54E-28	0,000344	3,09332E-27	0,035772222	0,000178785	1,7399E-26	0,001625103	-0,847364236	0,194072414	-0,317072142	0,893200386	0,284414346
CDKN2A	5,84E-14	0,0505	0,0605	0,000000422	0,000904	2,0341E-13	0,096988318	0,148008929	1,00255E-06	0,003538514	1,335332277	-0,412524164	0,070039863	-0,72658532	-0,535242256
CDT1	0,209	0,0000145	0,218	0,00963	0,127	0,241289326	0,00011919	0,382897436	0,014713494	0,212182927	-0,03833533	-0,22782233	0,028095903	0,12253221	0,100556489
CDX2	3,51E-12	0,208	0,121	1,79E-08	0,0194	1,01592E-11	0,304227758	0,248655	4,9046E-08	0,044525	0,326606599	-0,09700233	0,067313049	-0,232048642	-0,144044195
CHEK1	7,67E-08	0,000184	0,912	0,000139	9,91E-09	1,47999E-07	0,00093363	0,944161209	0,000257338	5,09126E-07	-0,445371167	-0,348275748	-0,006650057	0,249599473	0,679920989
CHEK1.pS345	0,296	0,425	0,468	0,768	0,102	0,335140496	0,535431193	0,620477419	0,793085427	0,178391489	-0,051906613	0,006554082	-0,015842834	-0,001730731	0,091032901
CHEK2	6,03E-10	0,0748	0,00132	5,18E-16	0,0000927	1,40814E-09	0,131943348	0,008750323	3,67066E-15	0,00055217	-0,328108303	-0,13140712	-0,189023888	0,485060031	0,262481559
CHEK2.pT68	0,806	0,372	0,858	0,0155	0,0786	0,819965347	0,488472843	0,908860825	0,022998195	0,142940708	0,049631063	0,030279432	-0,034574614	-0,120517566	0,110100672

CLPP	0,00559	0,456	0,273	0,712	0,514	0,007532754	0,557785714	0,436587549	0,746510204	0,625011834	0,087621858	-0,02168115	-0,056053235	0,002319304	-0,010045503
COPS5	1,18E-16	0,315	0,127	0,00000703	5,73E-08	5,32945E-16	0,42447541	0,257882353	1,3891E-05	1,38531E-06	0,352941261	-0,05267033	0,075753172	-0,203125578	-0,280028406
CREB1	0,0476	0,0875	0,128	0,00506	0,445	0,059283636	0,149221992	0,257882353	0,008092062	0,56623839	-0,036439836	0,060500248	-0,047013718	0,073280732	-0,037925835
CREB1.pS133	0,0036	0,00172	0,000482	0,00123	0,416	0,004965101	0,006201053	0,004214936	0,002071844	0,541063291	-0,125999236	0,159744459	-0,133635071	0,140739493	0,024188835
CRM1	0,0132	0,164	0,00173	0,00153	0,27	0,017168354	0,251507463	0,010014507	0,00254587	0,399172662	0,300562623	-0,090617974	-0,227861399	0,229400653	-0,248029223
CSE1L	2,5E-20	0,954	0,269	3,68E-15	0,00103	1,51103E-19	0,973581683	0,433564706	2,40076E-14	0,003919722	-0,679575484	0,00913734	-0,103369608	0,60582698	0,294815853
CSK	0,00045	0,00000595	0,159	0,00381	0,000151	0,000670109	6,27038E-05	0,305369159	0,006213929	0,000795654	0,234524916	0,272226381	-0,071811007	-0,204931024	-0,241473303
CSNK2A1	0,0267	0,0108	0,34	0,000000571	0,442	0,033974303	0,029790604	0,490315789	1,34103E-06	0,564167702	-0,024232904	-0,112336335	-0,041158184	0,159746486	0,014845276
CTCF	4,55E-24	0,425	0,162	2,35E-27	0,0382	4,4525E-23	0,535431193	0,309683721	9,6585E-26	0,079696447	-1,155270171	0,077040038	-0,025446856	0,938549837	0,313969166
CTNNB1.pS33_37_T41	0,0000217	0,032	0,815	0,644	0,00327	3,55327E-05	0,071091892	0,883812665	0,683937984	0,010582441	0,113179177	-0,061056187	-0,007387608	-0,001964663	-0,072920584
CTNNB1.pT41_S45	4,39E-08	0,0217	0,0324	0,00118	0,00288	8,63297E-08	0,050964	0,094442553	0,00200405	0,009545806	0,128918563	-0,063511258	0,057262896	-0,083675228	-0,087858827
CXCR5	0,0162	0,204	0,00141	0,00486	0,504	0,020806875	0,301597122	0,008915538	0,007802578	0,6165	-0,15227921	0,127524683	0,214931174	-0,222887886	0,016549396
DAPK1.pS308	0,126	0,0168	0,00978	0,0115	0,723	0,147538462	0,040856805	0,038649808	0,017313187	0,811893443	-0,156142675	0,240786408	-0,205472258	0,199285793	0,013400993
DDB1	0,112	0,000544	0,45	0,414	0,917	0,132657061	0,002353516	0,600487013	0,461121951	0,941917706	0,204597989	-0,223695758	0,005640898	0,003188836	-0,045341619
DLST	4,17E-22	0,0000134	0,0000007	8,29E-17	0,000516	2,95495E-21	0,000112396	0,000048498	6,42866E-16	0,002209125	0,671832074	-0,309762164	0,198884502	-0,474997309	-0,273099286
DLX1	5,49E-12	0,0402	0,00417	1,6E-14	0,453	1,55613E-11	0,081997537	0,019699655	9,67059E-14	0,572870769	-0,442574523	0,108301057	-0,186654566	0,547556537	0,068848125
DNM1L	0,000000731	0,0000261	0,000482	0,0000355	0,296	1,31772E-06	0,00019865	0,004214936	6,66233E-05	0,422337931	-0,419156606	0,392233506	-0,198073152	0,290867772	0,074630672
DNMT1	4,92E-12	0,000338	0,0107	0,00109	0,000149	1,40425E-11	0,001526571	0,039979091	0,00185888	0,000795312	-0,648523466	0,338456743	-0,203906268	0,313089826	0,427944355
DPF2	3,72E-22	0,18	0,0106	4,89E-27	0,399	2,68232E-21	0,27298893	0,039979091	1,82708E-25	0,523926518	-0,553028073	0,073697542	-0,090362275	0,574208345	0,07096612
DUSP4	2,97E-10	9,98E-09	0,000042	2,72E-09	0,774	7,2229E-10	5,12723E-07	0,000822	7,98514E-09	0,841571429	0,23095286	-0,205833628	0,179180537	-0,254913273	-0,040356976
DUSP6	5,6E-22	0,00000668	0,894	0,000000304	0,601	3,836E-21	0,000068637	0,934659898	7,30667E-07	0,714525862	-0,787047201	0,517217324	0,005296212	0,390070546	-0,011734075
DVL3	3,11E-14	0,578	0,751	0,000000188	0,0000884	1,11149E-13	0,672968839	0,838752717	4,6547E-07	0,0005343	-0,325205009	-0,037214853	-0,033271755	0,259281518	0,201251317
E2F1	5,55E-09	0,483	0,43	0,00102	0,000889	1,1638E-08	0,585584071	0,581578431	0,00174675	0,00351326	0,276700989	-0,016080839	0,023515628	-0,177019337	-0,167513658
EEF2	3,34E-12	0,426	0,267	4,33E-11	0,00428	9,73574E-12	0,535431193	0,433564706	1,6789E-10	0,013219478	-0,797407604	-0,109598057	-0,040581955	0,741405627	0,311500701
EEF2K	1,34E-26	0,0308	0,00632	3,15E-28	0,279	1,66891E-25	0,06917377	0,028233913	1,8495E-26	0,40662766	-0,901620587	0,186034216	-0,302091495	1,109253492	0,062865557
EGFR	0,709	0,479	0,189	0,672	0,0188	0,734002519	0,582452663	0,349905405	0,706373402	0,043408989	-0,014331505	0,042348866	0,082991704	-0,028480675	-0,121626483
EIF2AK2	4,63E-13	0,47	0,452	0,000000612	0,00000745	1,49837E-12	0,573204748	0,601203883	1,42108E-06	6,00382E-05	-0,574317399	-0,101063054	-0,032798059	0,344206585	0,501188972
EIF2AK3	0,0765	0,00192	0,374	0,0909	0,704	0,093575893	0,006861913	0,521064407	0,117483962	0,801506925	-0,16294779	0,207020176	0,07530202	-0,099511608	0,003913807
EIF2S1	0,00000114	0,957	0,0116	0,317	0,153	2,03713E-06	0,973581683	0,04145431	0,362916435	0,247570866	0,374965601	-0,020070416	-0,155863741	-0,088089433	-0,128993374
EIF2S1.pS51	0,764	0,000655	0,616	0,05	0,00000132	0,78501	0,00274699	0,735976744	0,068272425	0,00001644	0,009678292	-0,210015205	0,013631631	-0,098262323	0,325180984
EIF4E	0,632	0,000154	0,0473	0,132	0,000000104	0,672932642	0,00080119	0,124617308	0,165402439	2,24968E-06	0,076972974	-0,208656467	-0,087318803	0,02539143	0,23079088
EIF4E.pS209	1,28E-16	0,278	0,195	0,0752	4,07E-16	5,65677E-16	0,38086	0,354623894	0,099061538	8,36385E-14	-0,901039561	0,129054044	-0,091013943	0,183181809	0,994785339
EIF4EBP1	6,59E-13	0,18	0,0667	4,49E-15	0,00146	2,08345E-12	0,27298893	0,158460694	2,88342E-14	0,005263684	-0,626559029	-0,081023014	-0,115577638	0,633466519	0,305456944
EIF4EBP1.pS65	5,35E-33	0,000712	0,0532	1,46E-13	0,0000092	4,3977E-31	0,002897347	0,137516981	7,5957E-13	7,27154E-05	-0,83011705	0,247000526	-0,082430881	0,49510486	0,348316652
EIF4G1	0,3	0,156	0,664	0,596	0,152	0,338736264	0,241674157	0,766739496	0,636249351	0,246924901	0,140910294	-0,094954262	-0,035726011	-0,055847715	0,041583735
EIF4G2	0,000222	0,0000283	0,0000401	0,000026	0,774	0,00033919	0,000207702	0,000822	4,94336E-05	0,841571429	-0,298806574	0,40669208	-0,372927295	0,39045212	0,041547267
ELK1.pS383	0,000000612	1,79E-08	0,858	0,647	0,401	1,11297E-06	5,65915E-07	0,908860825	0,685353093	0,524875796	0,247606403	-0,203383241	-0,03700164	-0,043508511	0,001639284
EP300	2,01E-20	0,00583	0,238	0,000000901	0,000138	1,233E-19	0,017749111	0,405883817	2,06878E-06	0,000751697	-0,413882657	0,12889663	-0,023223375	0,208851566	0,188407264
EPHA2	6,88E-13	0,0172	0,0639	0,000000477	0,0131	2,15853E-12	0,041583529	0,153584211	1,12671E-06	0,032048214	0,227984334	0,023147806	0,124883312	-0,25499551	-0,203443015
EPHA2.pS897	0,681	0,529	0,26	0,0292	0,941	0,70858481	0,6302	0,427374502	0,041526644	0,950248157	-0,018664219	0,061536126	0,113762899	-0,120439089	-0,066097427
EPHA2.pY588	1,71E-08	0,00319	0,332	0,000457	0,474	3,46212E-08	0,010573306	0,48705694	0,000802679	0,590345455	-0,295994212	0,181688088	-0,059208462	0,177542552	0,066578672
ERBB2	0,274	0,963	0,7	0,573	0,284	0,311088398	0,977266667	0,799166667	0,614890339	0,411	0,005702529	0,032897183	0,077588061	-0,058728533	-0,090677396
ERBB2.pY1248	0,347	0,0289	0,252	0,0343	0,0153	0,386495935	0,065623757	0,419319838	0,048278425	0,036348555	0,043097054	0,070382995	0,066143577	-0,120611131	-0,084672259
ERG	6,77E-09	0,0761	0,181	0,00000197	0,125	1,40529E-08	0,133662821	0,338140909	4,23911E-06	0,209693878	-0,394446917	-0,101275985	0,102556352	0,286267811	0,116582558
ERN1	6,94E-17	0,0338	0,886	3,23E-15	0,00473	3,2413E-16	0,074287701	0,931319693	2,14118E-14	0,013787447	1,31456573	0,289247286	-0,181194764	-1,003684245	-0,524889257
ESR1	0,000266	0,000296	0,77	0,966	0,891	0,000403417	0,001366921	0,853018868	0,966	0,921135678	0,091264006	-0,106094757	-0,015272092	0,002951839	0,012932321
ETS1	1,16E-23	0,267	0,227	4,64E-15	4,38E-08	9,9325E-23	0,367013378	0,393658228	2,93391E-14	1,20012E-06	1,045154262	0,016180378	-0,018731052	-0,667178948	-0,549392246
EZH2	1,25E-30	0,355	0,657	1,95E-21	0,00251	5,1375E-29	0,473717532	0,764949008	2,76362E-20	0,008525702	-1,539188455	-0,159480427	-0,00086267	1,414487862	0,442607762
EZR	2,2E-11	0,0338	0,639	9,68E-12	0,00398	5,79615E-11	0,074287701	0,750368571	3,97848E-11	0,012582923	0,360853227	0,087759502	-0,00072615	-0,348331567	-0,136271503
FASN	3,48E-10	0,0162	0,391	0,00000531	0,485	8,41341E-10	0,039632143	0,537461538	1,08578E-05	0,602220544	0,397372759	-0,147361731	0,011296602	-0,257143109	-0,063756634
FLI1	0,0000994	0,392	0,476	0,000000934	0,296	0,000154748	0,505053292	0,625035144	2,13263E-06	0,422337931	-0,242121786	0,068767604	-0,015283652	0,219551695	-0,006341288
FN1	0,0000381	0,0124	0,335	8,84E-10	0,0948	6,04598E-05	0,033309804	0,488244681	2,81647E-09	0,167222318	0,646739077	0,508244905	0,19022789	-1,153297728	-0,247896323
FOS	0,0000695	0,0612	0,666	0,234	0,0615	0,000109863	0,111661062	0,766739496	0,278765217	0,117020833	-0,477909646	0,346083847	-0,080557291	0,101112018	0,266584028
FOXM1	2,73E-12	0,0897	0,0827	1,97E-14	0,879	8,07216E-12	0,152341736	0,183728108	1,15667E-13	0,914605063	0,428509554	-0,064288913	0,024880719	-0,396010025	-0,039160033
FOXO3	0,0000156	0,00117	0,0383	0,0117	0,171	2,59579E-05	0,004411651	0,107817123	0,01755	0,269275862	0,499977414	-0,364751624	0,156655841	-0,275376843	-0,170046305
FOXO3.pS318_321	0,638	0,00112	0,0454	0,274	0,832	0,677565891	0,004300278	0,122759211	0,319926136	0,883596899	0,01907755	0,164425488	-0,056753557	-0,067718944	-0,030009019
FRS2A.pY196	6,09E-15	0,00000767	0,0115	0,000000948	0,0448	2,31758E-14	7,50564E-05	0,04145431	2,15264E-06	0,091108374	0,295266569	-0,158978311	0,068556736	-0,185040569	-0,095752514
FZR1	4,79E-27	0,00085	0,0143	0,000000012	1,47E-08	7,03104E-26	0,003391748	0,047397581	3,33243E-08	6,713E-07	1,216820147	-0,43171571	0,120477593	-0,610102471	-0,583330818
G6PD	1,73E-11	0,0941	0,0000377	1,19E-12	0,00144	4,61708E-11	0,158504508	0,000822	5,49539E-12	0,005263684	0,346249118	-0,088606688	0,154601109	-0,346142465	-0,170061576
GAB2	2,99E-09	0,00468	0,509	0,000324	0,488	6,42188E-09	0,014571818	0,649686335	0,000581502	0,602964072	-0,379778308	0,174987048	-0,049849358	0,267546632	0,056677715
GAB2.pY452	2,95E-09	0,00013	0,803	0,362	0,119	6,38132E-09	0,000693896	0,87774734	0,407621918	0,202103306	0,408315012	-0,285487431	-0,075071498	0,012874549	-0,140239545
GAPDH	0,492	0,0277	0,0133	0,0267	0,356	0,534952381	0,063601676	0,045176033	0,03836958	0,47972459	-0,146957333	0,322813483	-0,280151788	0,292452692	-0,092737731
GATA1	0,994	0,00000472	0,00000515	0,0283	0,113	0,994	5,54263E-05	0,000192423	0,040386458	0,194322176	-0,170939816	-0,637034073	0,480031717	-0,180641782	0,41287975
GATA3	2,47E-30	0,447	0,592	1,68E-15	0,00471	7,809E-29	0,548408955	0,715623529	1,1508E-14	0,013787447	0,557861832	-0,03182911	-0,043741665	-0,377013961	-0,16789364
GLS	0,816	0,814	0,0016	0,000236	0,805	0,828088889	0,866720207	0,009649565	0,000433018	0,863633766	-0,004396759	-0,000171982	-0,176530093	0,234675502	-0,019369544
GPX4	0,00855	0,0578	0,158	0,414	0,0181	0,011335645	0,106528251	0,304873239	0,461121951	0,042028814	0,135631949	0,120401538	-0,074491092	-0,027656291	-0,164627003
GRP75	1,87E-09	0,913	0,747	0,0000225	0,013	4,17701E-09	0,942821608	0,837678474	4,34155E-05	0,031994012	-0,547521669	-0,046114409	-0,065187435	0,442857995	0,326834069
GRP78	0,0000113	0,821	0,0461	9,55E-15	0,154	1,88793E-05	0,871914729	0,123837255	5,85828E-14	0,248211765	0,19533007	-0,028928214	0,053725706	-0,315718541	0,09038952
GSKA_B	0,27	0,00303	0,00774	0,812	0,434	0,307396122	0,010207623	0,0322224	0,830179104	0,556962617	0,193291941	0,241459634	-0,228477041	-0,0561854	-0,096402848
GSKA_B.pS21_9	0,00804	0,0136	0,745	5,58E-08	0,928	0,010693981	0,035154717	0,837678474	1,44238E-07	0,948776119	0,109894325	0,135665682	0,023177587	-0,254728805	-0,005775981
GYS1	0,004	0,0149	0,422	0,264	0,285	0,00548	0,037340854	0,576219269	0,309128205	0,411	-0,220264888	0,208614974	0,086759274	-0,128891787	0,100544482
GYS1.pS641	0,000316	0,0193	0,0000642	0,00585	0,892	0,000477485	0,045851445	0,001112988	0,009319186	0,921135678	-0,301777539	0,192588439	0,333576104	-0,233170202	-0,025563115
H2AX.pS139	0,0747	0,214	0,192	0,35	0,84	0,091646866	0,308610526	0,352285714	0,396280992	0,889793814	-0,197273501	0,158772685	0,052330485	-0,077784784	0,11231274
H2BFM	5,34E-24	0,000954	0,000925	4,59E-14	0,0000944	5,10405E-23	0,003770135	0,006788839	2,58423E-13	0,000554263	0,589856874	-0,23156147	0,133943482	-0,388458853	-0,257211712
H3K27Ac	0,00000385	0,988	0,00196	0,0523	0,489	6,7334E-06	0,992414634	0,010599474	0,070941584	0,602964072	-0,350863942	0,027832886	0,181059625	0,134241506	-0,012426656
H3K27Me3	0,0016	0,504	0,205	0,00662	0,0000072	0,002267586	0,607460411	0,367925764	0,010384809	5,97594E-05	0,212525744	-0,025402299	-0,072896361	0,210888621	-0,421124665
H3K36Me3	0,0122	0,141	0,125	0,000275	0,109	0,015918095	0,222034483	0,255597015	0,000500111	0,189025316	-0,150359803	-0,043891153	0,073335014	0,18566862	-0,103443515
H3K4Me1	0,00228	0,0313	0,0136	0,0217	0,0733	0,003198225	0,069914674	0,045816393	0,031739146	0,135464126	0,09867235	-0,075436054	0,096505438	-0,093756189	-0,082361865
H3K4Me2	9,47E-08	0,128	0,0689	0,00000593	0,0783	1,80193E-07	0,206299611	0,162746552	1,20061E-05	0,142940708	0,199247217	-0,053060751	0,065857343	-0,175713694	-0,089496163
H3K4Me3	0,821	0,0427	0,939	0,00455	0,458	0,831110837	0,085591748	0,957640199	0,007362402	0,573896341	-0,032224514	-0,072640944	0,003754221	0,114044759	-0,029830024
H3K9Me2	0,329	0,00154	0,483	0,0216	0,189	0,368444142	0,00565125	0,629506329	0,031705714	0,294238636	0,024334032	-0,133765993	0,04042698	0,117715951	-0,102377207
HDAC1	0,00306	0,562	0,315	0,834	0,000105	0,004263254	0,661122857	0,4795	0,846355556	0,000591164	0,130113733	-0,029810082	0,009314338	0,002035155	-0,162266254
HDAC2	0,000332	0,858	0,001	3,83E-13	0,806	0,000499824	0,9042	0,007086207	1,85192E-12	0,863633766	-0,150563783	0,01027897	-0,119858163	0,30633665	-0,014042093
HDAC3	9,21E-11	3,07E-12	0,00064	0,00777	0,133	2,33661E-10	1,26177E-09	0,005058462	0,012096477	0,220415323	0,241846003	-0,234237594	0,102649156	-0,113136131	-0,086969931
HDAC6	0,0000336	0,425	0,0229	0,00000252	0,132	5,39438E-05	0,535431193	0,070766165	5,33876E-06	0,219643725	-0,264437669	0,066875529	-0,155774974	0,326875982	0,109179845
HES1	0,0000381	0,0348	0,00711	0,0000194	0,266	6,04598E-05	0,075277895	0,030439688	3,76104E-05	0,3946787	0,215514965	-0,11661128	0,153577724	-0,262855899	-0,067060164

HEXIM1	8,51E-16	0,687	0,241	0,00000142	0,000000713	3,46298E-15	0,752952	0,407617284	3,10436E-06	0,000009453	-0,467571839	-0,00082484	-0,064936833	0,284829629	0,372519314
HIF1A	5,38E-20	0,0000356	0,00528	5,22E-10	0,00538	3,20461E-19	0,00024386	0,024382921	1,73018E-09	0,015145068	0,426426868	-0,176371599	0,090687442	-0,281419195	-0,165533221
HIST3H3	0,228	0,738	0,00784	0,536	0,022	0,26175419	0,798205263	0,0322224	0,578204724	0,049141304	-0,111176369	0,006519049	0,128772088	0,074138184	-0,155531966
HK2	2,41E-10	0,0112	0,0267	0,00262	0,138	6,00309E-10	0,030688	0,079817391	0,004324578	0,226872	-0,419374284	0,153948625	0,129137005	0,260686461	-0,140423281
HNRNPK	0,00000715	0,267	0,00099	1,98E-10	0,332	1,20932E-05	0,367013378	0,007086207	7,01534E-10	0,454694352	-0,274461362	0,039354942	-0,20683481	0,442682693	0,083609913
HSF1	7,58E-19	0,182	0,00188	6,54E-22	0,0571	4,26764E-18	0,275007353	0,010412	9,59979E-21	0,110698585	-0,478577372	0,073369926	-0,164373977	0,546692627	0,122530148
HSF1.pS326	7,18E-33	0,681	0,19	0,0171	5,34E-20	4,9183E-31	0,752395161	0,350179372	0,025190323	2,19474E-17	-1,02520751	0,027009507	0,112258454	0,227965821	0,897265854
HSP60	1,29E-11	0,371	0,0221	1,76E-10	0,00995	3,51119E-11	0,488472843	0,069336641	6,29009E-10	0,025882595	-0,717366688	0,011174873	-0,334377425	0,784828589	0,477283023
HSP90AA1_B1	0,114	0,0000327	0,187	0,311	0,000679	0,134637931	0,000227792	0,347769231	0,358042017	0,002763059	-0,048475182	-0,137585356	0,03722853	0,02445931	0,127662133
HSPB1	2,43E-10	0,129	0,0000677	5,78E-09	0,0000291	6,01645E-10	0,206299611	0,001112988	1,64683E-08	0,000206209	0,253068024	-0,066721561	0,153689395	-0,254485813	-0,184617105
HSPB1.pS82	0,303	0,073	0,867	6,97E-12	4,25E-12	0,341186301	0,129323276	0,913684615	2,92313E-11	5,8225E-10	-0,237106543	0,302240535	0,006007768	-1,072543542	1,374659846
IDO1	0,000000011	0,0387	0,0466	0,000859	0,00133	2,23812E-08	0,080739594	0,124095484	0,001477192	0,004969364	0,233461894	-0,095039653	0,046001503	-0,113034976	-0,139881857
IGF1R	0,0846	0,583	0,0549	0,0592	0,0886	0,102871598	0,674224719	0,140298148	0,079254723	0,157638961	0,127466395	-0,007478231	0,134050742	-0,178873482	-0,145663801
IGF1R.pY1135_1136	1,26E-16	0,978	0,055	6,24E-14	0,000452	5,62891E-16	0,987611794	0,140298148	3,37453E-13	0,001997548	0,37057316	-0,004593315	0,031395839	-0,297194927	-0,164766941
IGFBP2	0,00000156	0,0308	0,0468	1,74E-10	0,152	2,77558E-06	0,06917377	0,124095484	6,27316E-10	0,246924901	0,321567919	-0,057775919	0,071500559	-0,508267026	0,171924341
IKB	3,61E-09	0,0362	0,0104	1,93E-13	0,643	7,68762E-09	0,077490625	0,039947664	9,67354E-13	0,750775568	-0,549821359	0,194543525	-0,180690994	0,653439884	-0,027034677
IL23R	0,0222	0,924	0,793	0,0232	0,605	0,028336025	0,951789474	0,869128	0,033693286	0,714525862	-0,092518859	0,019852573	-0,010557694	0,077009816	0,021101683
INPP4B	2,22E-10	0,788	0,814	9,23E-11	0,583	5,56354E-10	0,845608355	0,883812665	3,41759E-10	0,698580175	0,425271141	-0,005674564	-0,017467215	-0,480529388	0,077376975
IRS1	0,851	0,142	0,00784	0,000068	0,0118	0,857257353	0,222755725	0,0322224	0,000127036	0,029215663	0,002893401	0,071031242	-0,113702315	0,170545148	-0,124184669
IRS2	0,0000879	0,112	0,48	0,0000157	0,0264	0,000137889	0,183394422	0,628280255	3,05815E-05	0,057714894	0,305928965	0,107510389	0,016408267	-0,343322783	-0,11668536
ITGA2	9,75E-27	0,444	0,000331	7,46E-24	0,0000021	1,29266E-25	0,548	0,003551462	1,61372E-22	2,25523E-05	0,60174543	-0,043035497	0,115862543	-0,512233472	-0,292406681
ITGAL	0,0414	3,07E-09	0,00378	0,194	0,000476	0,05187622	4,2059E-07	0,018208256	0,235899408	0,002059326	0,113919366	0,933086373	-0,345645025	-0,171346627	-0,417531674
ITGB1	2,71E-18	0,0000973	0,00187	3,91E-14	0,165	1,42796E-17	0,00057129	0,010412	2,23196E-13	0,261833977	0,329771544	-0,137770236	0,078386651	-0,259565136	-0,085047588
JAG1	3,09E-17	0,0614	0,000397	1,29E-16	0,00344	1,47673E-16	0,111661062	0,003999732	9,81833E-16	0,011045625	0,529409705	-0,234641154	0,321677846	-0,495915366	-0,328746969
JAK2	1,12E-19	8,72E-09	0,0904	1,52E-09	0,844	6,48338E-19	5,12723E-07	0,198686631	4,59353E-09	0,891732648	-0,835828132	0,585760681	-0,107731245	0,5038324	0,01021187
JMJD6	4,13E-08	0,0654	0,0759	0,000000155	0,00000416	8,16072E-08	0,118411454	0,176242373	3,86091E-07	3,97619E-05	0,220228348	0,080055098	0,10868914	-0,243995083	-0,244433512
JUN.pS73	0,0917	0,014	0,295	0,016	0,0415	0,110038081	0,03573913	0,461007605	0,023654676	0,085711055	0,098365113	-0,119795591	0,019217801	-0,096948261	0,093639969
JUNB	4,53E-10	0,486	0,00539	3,21E-21	0,457	1,0762E-09	0,587488235	0,024614333	4,3977E-20	0,573896341	0,390568919	0,05216586	0,114246582	-0,522456408	-0,093203934
KAT2A	0,708	0,0379	0,00228	0,00938	0,000468	0,734002519	0,080293299	0,01216987	0,014431007	0,002046255	-0,010681364	-0,099810685	-0,14735085	0,120315624	0,20187666
KDM1A	1,69E-15	0,0352	0,233	2,6E-12	0,000000451	6,67875E-15	0,075744503	0,3990125	1,13681E-11	6,39176E-06	-0,481318952	-0,084031681	-0,065760029	0,376973469	0,364838662
KDR	0,0103	3,74E-08	0,195	0,0000034	0,0633	0,01352492	0,000001096	0,354623894	7,0934E-06	0,119340826	0,233695438	0,447072952	-0,041688352	-0,41598761	-0,207013697
KEAP1	0,665	0,0383	0,484	0,507	0,295	0,697232143	0,080312755	0,629506293	0,550891821	0,422337931	-0,084878614	-0,213437784	0,159919269	-0,004408844	0,104563511
KIT	1,07E-16	0,0157	0,271	2,21E-10	0,000296	4,88633E-16	0,038871687	0,435082031	7,76333E-10	0,001431247	-1,077255343	-0,344259306	0,159558553	0,831913004	0,518937352
KMT2A	0,000000003	0,0535	1,67E-08	1,78E-17	0,0212	6,42188E-09	0,101798611	4,2744E-06	1,49302E-16	0,047874725	-0,405300075	0,141506062	-0,313984923	0,54200634	0,190009887
KMT2D	2,51E-26	0,888	0,00703	1,57E-23	0,00000529	3,03415E-25	0,92396962	0,030414	2,93305E-22	4,94113E-05	-0,560272586	-0,006981158	-0,11202108	0,513440458	0,283382243
LATS1	0,791	0,57	0,391	0,178	0,000424	0,806702233	0,667435897	0,537461538	0,217732143	0,001914989	0,003176357	-0,026737652	-0,03849725	-0,046069586	0,146074725
LATS2	0,00615	0,00296	0,196	0,118	0,938	0,008233388	0,010054215	0,354872247	0,149685185	0,950248157	0,093255783	-0,103569937	0,024994665	-0,050495265	0,013142706
LCK	2,45E-33	0,0768	0,298	9,82E-24	7,31E-09	2,51738E-31	0,133749153	0,463931818	2,01801E-22	4,29201E-07	0,948593797	0,079778582	-0,025396065	-0,688341118	-0,449680875
LEF1	5,3E-39	0,156	0,5	6,54E-12	0,00000034	2,1783E-36	0,241674157	0,64146729	2,79994E-11	5,5896E-06	1,584062363	-0,272710566	-0,230224688	-0,688438979	-0,603976616
LGALS3	3,78E-14	0,00289	0,0122	9,78E-19	0,00000042	1,32785E-13	0,00989825	0,04285641	9,34786E-18	0,000006165	0,708919691	0,344629095	0,08156528	-0,757939371	-0,493868168
LMNB1	1,8E-16	0,0000377	0,000619	2,92E-20	0,361	7,78737E-16	0,000254011	0,005044824	3,31022E-19	0,484872549	-0,863579349	0,489965954	-0,308943886	0,945008206	-0,100864669
LRP6.pS1490	0,000000667	0,000302	0,427	0,111	0,42	1,20765E-06	0,001379133	0,581115894	0,141680124	0,544542587	0,218447257	-0,131719989	-0,050238388	-0,068190461	0,015289594
LYN	0,00000421	0,000000339	0,248	0,262	0,872	7,33182E-06	6,63471E-06	0,414341463	0,308544413	0,912984733	-0,196811136	0,291952213	0,049287512	-0,088563805	-0,016767303
MAP1LC3A_B	0,404	0,0404	0,0582	0,000516	0,00589	0,445158177	0,081997537	0,144345181	0,00089657	0,016246913	0,043590418	-0,12125189	0,032444557	-0,16309974	0,235171722
MAP2K1	0,0000854	0,00105	0,0116	5,84E-15	0,0207	0,00013448	0,00411	0,04145431	3,63673E-14	0,047003867	0,229056977	0,315415519	0,132622678	-0,537548648	-0,173046905
MAP2K1.pS217_221	0,0000309	0,0347	0,89	0,508	0,0857	4,98035E-05	0,075277895	0,933137755	0,550891821	0,153810917	-0,199001837	0,111452048	0,015145714	0,048003048	0,062355747
MAPK1	0,68	0,0538	0,0386	0,757	0,795	0,70858481	0,101897696	0,107922449	0,785674242	0,859755906	-0,014501091	0,208306024	-0,145170485	0,025248567	-0,013502321
MAPK1_3.pT202_Y204	7,03E-12	0,0158	0,0918	0,118	0,115	1,96553E-11	0,03888503	0,200690426	0,149685185	0,1969375	-0,907317501	0,470779871	0,148275289	0,285339864	0,113765784
MAPK14	5,67E-24	0,00000807	0,0564	1,05E-09	0,0107	5,2963E-23	7,71342E-05	0,142211043	3,29427E-09	0,026652727	-0,781160573	0,367534121	-0,11672349	0,483840275	0,217051525
MAPK14.pT180_Y182	1,14E-25	0,00000551	0,81	0,182	4,15E-10	1,33869E-24	6,12057E-05	0,883050398	0,221964392	4,26413E-08	-1,606821654	0,717290399	-0,027358295	0,292848647	1,065567892
MAPK7	0,0134	0,804	0,289	0,00941	0,316	0,017373502	0,858296104	0,456842308	0,014431007	0,441755102	0,169967245	-0,015478617	0,024047701	-0,147673153	-0,061290151
MCL1	0,00507	0,0000528	0,975	0,0000249	0,00575	0,006877129	0,000344457	0,975	4,75995E-05	0,015967905	0,166555084	0,247807138	0,004879629	-0,273695058	-0,153603976
MDM2	2,06E-09	0,0135	0,128	0,0712	0,663	4,55194E-09	0,035117089	0,257882353	0,094093891	0,76658427	-0,467149546	0,208996276	0,144134721	0,127808862	0,012997327
MDM2.pS166	8,43E-29	0,00606	0,00144	3,95E-17	0,00000541	2,03808E-27	0,01818	0,008967273	3,2469E-16	4,94113E-05	-0,700595724	0,186143091	-0,149233146	0,496862032	0,342077852
MECOM	0,415	0,0514	0,322	0,0101	0,00538	0,45605615	0,098257674	0,483	0,015317712	0,015145068	-0,053244756	-0,141747285	0,050352626	-0,127382869	0,315402188
MEF2C	3,61E-10	0,0118	0,17	0,000000998	0,405	8,67667E-10	0,031906579	0,323472222	2,22923E-06	0,528428571	0,272938307	-0,099745531	0,013140798	-0,215942864	-0,002819519
MEN1	7,33E-12	0,949	0,0000215	2,52E-16	0,0000449	2,03556E-11	0,973581683	0,000519794	1,81705E-15	0,000292919	-0,563480677	0,010480184	-0,302771607	0,654433802	0,38719369
MET.pY1230_1234_1235	0,00604	0,994	0,261	0,0536	0,015	0,008112549	0,994	0,427374502	0,072465789	0,035843023	0,143221447	-0,005183138	0,029548371	-0,093221828	-0,115617586
MKNK1	2,31E-09	0,00234	0,679	0,462	0,0000409	5,07706E-09	0,00822	0,779522346	0,507705882	0,000272453	-0,338232635	0,215655076	-0,007939523	-0,042633621	0,291803231
MSH2	0,0277	6,01E-08	0,556	0,00982	0,575	0,035029846	1,54382E-06	0,686234234	0,014948222	0,691008772	0,075830989	-0,171554357	0,034993828	0,060066131	-0,047424658
MSH6	5,25E-25	0,404	0,000444	7,37E-30	0,00748	5,67829E-24	0,5188875	0,0041374	1,00969E-27	0,020093333	-0,773916826	0,026954586	-0,300973967	0,986274079	0,222349708
MSI2	0,00349	0,00525	0,0404	2,67E-09	0,0319	0,004829596	0,016223684	0,111438926	7,89475E-09	0,067235385	-0,247938444	-0,28202873	-0,212945727	0,600359963	0,20745849
MSLN	0,222	0,747	0,626	0,562	0,622	0,255579832	0,805818898	0,741458213	0,604664921	0,730405714	-0,385596403	0,577411738	-0,332713108	0,40771967	-0,114745839
MTOR	2,17E-12	0,577	0,00168	2,18E-18	0,109	6,55787E-12	0,672968839	0,009864	1,94778E-17	0,189025316	-0,381723284	0,047224371	-0,143608401	0,459061215	0,100107675
MTOR.pS2448	1,82E-17	0,0126	0,368	0,0000822	0,0904	8,905E-17	0,033410323	0,51444898	0,00015287	0,160148276	-0,661481896	0,22166175	0,039123449	0,339770407	0,155320446
MUC1	0,122	0,224	0,000131	0,74	0,179	0,143262857	0,321902098	0,001736806	0,769974684	0,279730038	-0,113070199	-0,060672032	0,164437364	-0,028903249	-0,001154093
MYC	0,000000185	0,00724	0,0215	0,00898	0,00145	3,45614E-07	0,02109993	0,068289231	0,013875113	0,005263684	0,224216075	-0,133855373	0,090948365	-0,114688715	-0,153337948
MYH11	0,508	0,0000058	0,0393	0,928	0,00445	0,549442105	6,27038E-05	0,109137162	0,932537897	0,013448162	-0,007990625	0,330670297	-0,105947157	0,041731167	-0,241253345
MYH9.pS1943	0,665	0,0000107	0,962	0,0616	0,0402	0,697232143	9,35681E-05	0,966704156	0,081933981	0,083445455	-0,045070734	0,493172938	0,053089828	-0,223037526	-0,277889911
NCL	3,96E-19	0,723	0,00232	2,1E-23	0,001	2,2605E-18	0,784044855	0,012224615	3,75261E-22	0,003841121	-0,559928348	-0,044148561	-0,214893862	0,708878044	0,232827245
NCSTN	0,178	0,288	0,896	0,785	0,757	0,206661017	0,393249169	0,934659898	0,80457606	0,834120643	-0,053374951	0,037574782	-0,009818883	0,006933348	0,03793315
NDRG1.pT346	2,34E-16	0,00022	0,363	0,00393	0,204	9,91485E-16	0,001089398	0,512342466	0,006384308	0,315203008	-0,837939483	0,530362042	0,016408756	0,221137317	0,245311109
NDUFB4	1,29E-14	0,000146	0,000000159	9,68E-12	0,000121	4,77649E-14	0,000769308	0,000021783	3,97848E-11	0,000672041	0,401893336	-0,169157911	0,179852571	-0,324118429	-0,225651101
NF2	2,79E-18	0,0554	0,357	2,84E-10	0,0318	1,45151E-17	0,103028959	0,507705882	9,727E-10	0,067235385	0,82554103	-0,208884224	0,037137791	-0,554746739	-0,234961318
NFE2L2	1,82E-27	0,193	0,000337	3,05E-23	0,00000639	3,11675E-26	0,2895	0,003551462	5,22313E-22	5,70933E-05	0,699213135	-0,092800912	0,076750821	-0,524132842	-0,293192149
NLN	1,32E-28	0,106	0,259	3,13E-14	0,0000546	3,014E-27	0,174264	0,427374502	1,81187E-13	0,00034524	-0,932235987	0,148325092	-0,061549296	0,636051819	0,380715686
NOL3	0,00139	0,0000312	0,0636	0,15	0,961	0,001990557	0,000224968	0,153584211	0,185135135	0,968066176	-0,317388274	0,416181089	-0,128523917	0,117496987	0,024315068
NOTCH1	9,12E-08	0,00605	0,824	0,00357	0,809	1,7434E-07	0,01818	0,891221053	0,00586908	0,863633766	0,204933092	-0,094789133	-0,032561305	-0,100195592	0,006537791
NOTCH1.cle	4,75E-30	0,000003	0,00000135	8,66E-25	0,00575	1,39446E-28	3,85313E-05	6,93563E-05	2,15866E-23	0,015967905	1,977831631	-0,822697162	0,357158533	-1,357670327	-0,593998957
NOTCH2	7,34E-11	0,384	0,145	8,52E-10	0,124	1,87375E-10	0,499164038	0,282440758	2,73572E-09	0,208868852	0,373111135	-0,046959275	0,029870018	-0,315910059	-0,094638408
NOTCH3	0,104	0,656	0,0357	0,0827	0,0882	0,123537572	0,732652174	0,101474483	0,107562342	0,157609565	0,102479544	0,021951498	-0,069455178	0,075150057	-0,152066448
NPM1	0,00000743	0,00000696	0,312	0,0584	0,71	1,25153E-05	6,97698E-05	0,478226766	0,078696393	0,806104972	0,184735794	-0,216530359	0,025725826	-0,048715156	0,009556271
NRAS	7,05E-18	0,204	0,946	0,00000117	0,00105	3,62194E-17	0,301597122	0,959336609	2,58532E-06	0,003959174	0,401783472	0,012826094	-0,055717565	-0,210601957	-0,201327581
NUMB	0,927	0,00417	0,0358	0,668	0,332	0,931533007	0,013389609	0,101474483	0,703969231	0,454694352	-0,02161784	0,400173439	-0,180201787	0,015561942	-0,147894743
NUP98	3,37E-24	0,584	0,0197	6,53E-23	0,0000349	3,37822E-23	0,674224719	0,063255469	1,03224E-21	0,000239065	-0,535877083	-0,011670614	-0,104912717	0,510160079	0,248233894
OMA1	8,5E-09	0,85	0,000443	7,2E-16	0,0792	1,73806E-08	0,900386598	0,0041374	5,01559E-15	0,143397357	-0,436694031	0,007926111	-0,296461216	0,694467059	0,154691089

P90RSK.pT573	0,961	0,233	0,0809	0,172	0,501	0,963343902	0,327955479	0,181693443	0,211020896	0,614659701	-0,005352167	-0,01883398	0,05106085	-0,050895741	0,01346003
PAK1	7,75E-08	5,61E-09	0,92	0,456	0,381	1,48843E-07	5,12723E-07	0,947669173	0,502455764	0,505132258	-0,41686246	0,495605757	-0,03070293	-0,026256674	0,104437428
PAK4	1,28E-09	0,0436	0,0479	2,15E-08	0,28	2,89055E-09	0,086568116	0,125381013	5,73799E-08	0,40664311	0,535308831	-0,1921923	0,026419233	-0,315022621	-0,151409743
PARP	0,00103	0,000282	0,302	0,00000137	0,000565	0,001490599	0,001332207	0,468384906	3,01107E-06	0,002393969	-0,201891786	-0,258656498	-0,112874329	0,296320593	0,355483439
PARP.cle214	0,0508	0,00963	0,31	0,132	0,0446	0,063077946	0,02674277	0,478226766	0,165402439	0,091108374	-0,217069426	-0,287953636	-0,008980143	0,194186969	0,376898794
PAX6	1,47E-16	0,00243	0,000063	1,98E-13	0,00181	6,42734E-16	0,008463814	0,001112988	9,80458E-13	0,006304322	0,349914782	-0,151955123	0,12095165	-0,27230657	-0,147725197
PCNA	1,28E-09	0,957	0,0316	1,48E-09	0,00437	2,89055E-09	0,973581683	0,092768571	4,50578E-09	0,013304222	-0,40959377	0,035296465	-0,109064402	0,360281886	0,225110879
PDCD1	2,67E-27	0,000709	0,138	3,55E-11	0,000649	4,22065E-26	0,002897347	0,27137799	1,38957E-10	0,002694333	0,771513673	-0,278429945	0,036967575	-0,423527662	-0,254527494
PDCD4	6,92E-24	0,00121	0,0553	2,2E-12	0,00536	6,32027E-23	0,004521	0,140298148	9,82826E-12	0,015145068	-1,363813528	0,483244677	-0,130441248	0,860931778	0,416947136
PDGFRB	2,72E-13	0,00736	0,324	4,12E-10	0,873	9,16328E-13	0,021153566	0,484232727	1,37668E-09	0,912984733	0,305698471	-0,184267597	0,022727714	-0,327678586	0,175954332
PDHK1	0,711	0,19	0,00702	0,218	0,298	0,734223618	0,286043956	0,030414	0,262750733	0,422337931	-0,022231668	0,060578808	-0,118496374	0,063181182	0,068882062
PDK1	0,316	0,00661	0,387	0,264	0,062	0,354852459	0,019544676	0,535545455	0,309128205	0,117428571	-0,043791854	0,165628024	-0,033436788	0,027041489	-0,105520108
PDK1.pS241	7,66E-09	0,00000296	0,511	0,03	0,85	1,58204E-08	3,85313E-05	0,650219814	0,042517241	0,895769231	-0,355846202	0,312200047	-0,031541941	0,14320528	0,004887153
PDL1	2E-14	0,446	0,00326	1,98E-19	0,117	7,21053E-14	0,548408955	0,016142892	1,98483E-18	0,19953112	0,419536254	-0,038369461	0,137914698	-0,479659241	-0,125789184
PEA15	4,63E-22	0,0393	0,0809	7,65E-28	0,00146	3,22531E-21	0,081167337	0,181693443	3,93019E-26	0,005263684	0,7503884	0,064826988	0,063953751	-0,698893485	-0,289997779
PECAM1	5,94E-08	0,0184	0,411	6,02E-11	0,0168	1,15703E-07	0,044224561	0,56307	2,26993E-10	0,039456	0,377007141	0,140265999	0,062900321	-0,440034506	-0,197944898
PHLPP	9,43E-12	0,0686	0,00349	9,24E-10	0,00602	2,58382E-11	0,123120524	0,017076071	2,92126E-09	0,016385563	-0,495046982	0,123673659	-0,104418928	0,375129345	0,217516212
PIK3CA	9,04E-17	0,0000327	0,0353	0,423	0,0735	4,17465E-16	0,000227792	0,101456643	0,469556452	0,135464126	0,412001261	-0,199515006	-0,114034671	-0,041471144	-0,108007585
PIK3CB	1,18E-09	0,524	0,000000556	3,33E-20	0,0251	2,69433E-09	0,627883382	0,000048498	3,60166E-19	0,055462903	0,321107063	-0,035885693	0,225067537	-0,465412637	-0,150521322
PIK3R1	0,138	0,652	0,536	0,133	0,774	0,161130682	0,732163934	0,671634146	0,166148936	0,841571429	-0,047972998	-0,041218015	0,043340448	0,03493339	-0,002888169
PIM2	2,76E-25	0,00000445	0,0253	1,97E-16	0,448	3,06584E-24	5,37926E-05	0,077024444	1,44584E-15	0,568296296	0,40752908	-0,200948687	0,097254733	-0,323935095	-0,067651525
PKM2	3,68E-10	0,0000219	0,00116	0,0503	0,313	8,79349E-10	0,000173094	0,007946	0,068454636	0,440558219	-0,5909782	0,419257896	0,324626819	-0,193333533	0,081428336
PLCG1.pS1248	0,00000578	0,157	0,363	0,00000422	0,689	9,89825E-06	0,241674157	0,512342466	8,71568E-06	0,791002793	0,445894496	0,092438491	-0,133327947	-0,37052051	-0,018873407
PLCG2.pY759	0,000583	0,44	0,52	0,06	0,0603	0,000861917	0,546344411	0,656843558	0,080064935	0,115809813	0,139556742	0,021396284	-0,025198247	-0,073092202	-0,079486093
PLK1	1,76E-29	0,000242	0,0125	1,49E-18	0,0147	4,8224E-28	0,001170141	0,043538136	1,36087E-17	0,035331579	0,705061479	-0,234468995	0,069544968	-0,472164032	-0,200670292
PPARA	1,18E-18	0,0701	0,0153	0,00000104	3,99E-09	6,35182E-18	0,124723377	0,0503064	2,31049E-06	3,27978E-07	0,278610226	-0,064667459	0,069787293	-0,164126434	-0,206419747
PPP2R2A_B_C_D	0,0118	0,241	0,293	0,000447	0,332	0,015445223	0,335766102	0,461007605	0,000790249	0,454694352	-0,137273459	0,072669439	-0,010048802	0,134789305	-0,052057407
PREX1	0,0000205	0,000000481	0,32	0,00645	0,0301	3,38373E-05	8,23713E-06	0,483	0,010156897	0,064770157	-0,264889654	0,360952736	-0,064761132	0,17427132	-0,161133553
PRKAA1_2	0,0626	0,664	0,556	0,923	0,045	0,077263063	0,739577236	0,686234234	0,929786765	0,091108374	-0,078771622	0,002931085	0,016245485	-0,047022571	0,139830756
PRKAA1_2.pT172	3,4E-11	0,884	0,00639	0,0924	0,0107	8,90064E-11	0,92396962	0,028239677	0,119048276	0,026652727	-0,523235077	0,005277687	0,165740203	0,17344303	0,221405445
PRKAA2.pS345	0,0000215	0,265	0,748	0,473	0,0101	0,000035346	0,366717172	0,837678474	0,517029255	0,025944375	0,15729046	-0,011738788	-0,007318849	-0,043590502	-0,133744318
PRKAR1A	0,537	0,00000961	0,0216	0,47	0,0966	0,577767016	8,69354E-05	0,068289231	0,51512	0,169669231	0,051355016	0,332078852	-0,113304274	-0,063600267	-0,175341242
PRKCA	0,000574	0,00000956	0,621	2,08E-09	0,0134	0,000851675	8,69354E-05	0,73766185	6,19478E-09	0,032588166	0,288219211	0,482566258	0,026732196	-0,534715589	-0,274773901
PRKCA.pS657	0,0882	0,000000405	0,295	0,000000968	0,00225	0,106305572	7,23717E-06	0,461007605	2,17403E-06	0,00770625	0,180504414	0,706739245	0,131785141	-0,596907408	-0,460944857
PRKCB	0,000153	9,28E-11	0,356	0,144	0,658	0,000236402	1,90704E-08	0,507705882	0,17826506	0,766113314	-0,305354275	0,485993162	0,018630249	-0,101676377	-0,017406579
PRKCB.pS660	4,64E-08	7,89E-09	0,181	0,347	0,772	9,08114E-08	5,12723E-07	0,338140909	0,393969613	0,841571429	-0,437136764	0,505449777	0,091556019	-0,068111402	-0,017908935
PRMT1	5,79E-31	0,593	0,114	4,82E-25	0,0000177	3,39956E-29	0,682697479	0,23905102	1,32068E-23	0,000129905	-1,604504609	0,017861438	-0,126934752	1,314137809	0,665842904
PRMT5	0,0000303	1,55E-08	0,948	0,222	0,0173	4,90287E-05	5,65915E-07	0,959336609	0,266789474	0,040399432	0,342526686	-0,348726844	-0,039406015	-0,099043384	0,108638136
PRMT7	1,19E-18	0,0678	0,665	0,00000021	0,00259	6,35182E-18	0,122218421	0,766739496	5,1375E-07	0,008725328	-0,643129294	0,164416051	-0,014066233	0,342477537	0,274243083
PTEN	7,64E-27	0,00000973	0,0019	9,02E-28	0,511	1,04668E-25	8,69354E-05	0,010412	4,11913E-26	0,623207715	-1,393547733	0,626350585	-0,423673122	1,567320158	-0,154693835
PTGS2	0,202	0,0455	0,432	0,0392	0,0254	0,233864789	0,089672727	0,581578431	0,05442973	0,055825668	0,101572875	0,063500076	0,046987178	-0,116871432	-0,131514639
PTK2	0,000000364	0,584	0,0621	1,47E-16	0,587	6,67875E-07	0,674224719	0,15102426	1,09849E-15	0,701328488	0,565364398	0,11407401	0,214695121	-0,931691386	-0,027607535
PTK2.pY397	0,00108	0,00107	0,592	0,00000241	0,0105	0,001557474	0,004148774	0,715623529	5,13218E-06	0,02647546	0,08106282	0,180471037	0,050412585	-0,211083299	-0,115782128
PTPN11	8,84E-10	9,92E-09	0,367	0,0429	0,972	2,04115E-09	5,12723E-07	0,51444898	0,05916745	0,976753056	0,275884153	-0,215632912	0,005058532	-0,093037085	-0,025672976
PTPN11.pY542	0,00341	0,00161	0,692	0,226	0,047	0,004734831	0,005855841	0,792233983	0,270804665	0,093318841	-0,151396934	0,168603302	-0,000561752	-0,081013325	0,126683694
PTPN12	0,000713	0,0000249	0,636	0,614	0,62	0,001050333	0,000193092	0,750368571	0,653766839	0,730143266	-0,289005525	0,413364423	0,089309351	-0,103673358	-0,068750162
PTPN6	0,662	0,000121	0,283	0,00368	3,92E-08	0,697232143	0,000672041	0,449084942	0,006025817	1,20012E-06	-0,067069785	0,256467984	-0,03383676	0,13561051	-0,314670655
PTPN6.pY564	0,232	0,000000389	0,446	0,000625	0,318	0,265604457	7,23717E-06	0,597087948	0,001079307	0,442935811	-0,07067004	0,412670996	0,05246149	-0,28211321	-0,076081353
PXN	0,000000318	0,00307	0,329	0,00000002	0,143	5,8873E-07	0,010258293	0,48705694	5,40789E-08	0,234155378	0,540242779	0,323965455	-0,062514509	-0,604040772	-0,212409011
PYK2.pY402	0,547	1,41E-08	0,265	0,00824	0,399	0,586989556	5,65915E-07	0,432202381	0,012779774	0,523926518	-0,122561876	0,705582577	-0,078196357	-0,329051837	-0,061174881
RAB11	0,143	0,0156	0,47	0,00127	0,747	0,166495751	0,038858182	0,621125402	0,00213049	0,829775676	0,063568466	0,1035632	0,010064756	-0,155737191	-0,024186122
RAD50	3,71E-11	0,443	0,854	1,08E-13	0,764	9,6507E-11	0,548	0,908860825	5,69077E-13	0,839582888	-0,455898893	-0,119353641	-0,022055895	0,57438017	0,043321563
RAD51	1,9E-11	0,0000584	0,000245	6,36E-10	0,234	5,03806E-11	0,000369268	0,002797083	2,09117E-09	0,349723636	0,398925835	-0,257704325	0,158488597	-0,327963323	-0,084848389
RAF1	0,64	0,000701	0,364	0,059	0,781	0,677938144	0,002897347	0,512342466	0,079245098	0,846941953	0,03143996	0,126479649	-0,045787386	-0,089377419	0,005850248
RAF1.pS338	0,000142	0,0422	0,12	0,762	0,996	0,000220234	0,085020588	0,247839196	0,788871537	0,996	-0,174348413	0,115319281	0,055332612	0,018762162	-0,001234455
RB1	9,4E-21	0,0463	0,0939	3,52E-10	0,000156	5,94369E-20	0,090186256	0,202057068	1,18584E-09	0,00080145	-0,602969708	0,127040081	-0,089070959	0,41994394	0,27873931
RB1.pS807_811	1,44E-11	0,0391	0,0482	0,0141	0,00000174	3,89368E-11	0,081162121	0,125381013	0,020996739	0,000019865	-0,650666894	-0,235819072	0,145248157	0,262818097	0,578456795
RELA_P100	1,86E-09	0,372	0,858	2,32E-10	0,336	4,17701E-09	0,488472843	0,908860825	8,08068E-10	0,455762376	0,640397227	0,079652517	-0,008665853	-0,630887581	-0,129482481
RELA_P65	8,93E-12	0,00128	0,233	0,000339	0,319	2,46324E-11	0,004739459	0,3990125	0,000605778	0,442935811	0,22564441	-0,106416961	-0,040591478	-0,126709118	0,038271784
RELA_P65.pS536	0,828	0,000101	0,000126	0,735	0,387	0,836137592	0,000584662	0,0017262	0,767756345	0,511437299	-0,019434032	0,322446563	-0,282989694	-0,004921606	0,124458492
RHEB	1,82E-12	0,2	0,132	1,52E-10	0,137	5,54089E-12	0,297826087	0,263359223	5,5285E-10	0,22613253	0,277584668	-0,027038787	0,071074473	-0,271655528	-0,108877789
RICTOR.pT1135	0,434	0,212	0,0107	0,0000113	0,664	0,475664	0,306802817	0,039979091	2,21157E-05	0,76658427	0,056979833	0,085907008	0,104104289	-0,222760704	-0,048123915
RIP3	4,97E-16	0,44	0,919	0,00000019	0,00000727	2,0633E-15	0,546344411	0,947669173	4,67605E-07	5,97594E-05	0,380721454	0,022791114	0,001973785	-0,256649114	-0,215045359
RIPK1	0,0000085	0,655	0,549	0,000517	0,336	1,42592E-05	0,732652174	0,681688822	0,00089657	0,455762376	0,234346056	0,012041171	-0,024079106	-0,157899813	-0,09021762
RPA32	0,00000229	0,384	0,00000828	2,56E-20	0,755	4,05685E-06	0,499164038	0,00028359	3,00617E-19	0,834120643	-0,36331491	0,09433838	-0,304044413	0,690463117	-0,023951307
RPA32.pS4_8	2,79E-10	0,376	0,542	2,89E-08	0,375	6,86641E-10	0,492152866	0,675036364	7,56554E-08	0,498786408	-0,396988515	0,094843669	-0,022184162	0,292434734	0,092668762
RPS6	9,83E-13	0,332	0,521	0,000388	0,0000066	3,03769E-12	0,444469055	0,656843558	0,000690338	5,77149E-05	-0,412018953	-0,04506944	0,065682348	0,181792337	0,272649512
RPS6.pS235_236	3,42E-08	0,227	0,497	0,771	0,00000321	6,79043E-08	0,325076655	0,640335423	0,79418797	3,21783E-05	-0,471479845	0,186085999	-0,007076062	-0,037172646	0,498157386
RPS6.pS240_244	3,98E-21	0,0139	0,0935	0,525	2,08E-08	2,59648E-20	0,035705625	0,202057068	0,567828947	7,77164E-07	-1,320263472	0,414338876	0,163731665	0,113983963	0,938887356
RP56KB1	0,121	0,324	0,54	0,000448	0,704	0,142495702	0,435176471	0,674589666	0,000790249	0,801506925	-0,122793041	-0,018345992	0,004962524	0,141846871	-0,004141609
RPS6KB1.pT389	0,00000357	0,00000195	0,339	0,303	0,699	6,27038E-06	2,76362E-05	0,490315789	0,350797183	0,800247911	-0,190005147	0,241222245	0,036661191	-0,0573784	0,00948157
RPTOR	1,56E-15	0,867	0,586	3,54E-09	0,0376	6,28588E-15	0,911347826	0,714676558	1,02461E-08	0,078844898	0,333138028	0,033029562	-0,034444287	-0,250419949	-0,111765313
RSKA1_2_3	7,5E-21	0,0209	0,0225	1,84E-13	0,0027	4,81641E-20	0,049367241	0,070056818	9,3363E-13	0,009021951	0,552884833	-0,160789973	0,021117682	-0,335122401	-0,175634715
S100A4	0,000000262	7,06E-08	0,433	6,63E-12	0,00000126	4,87249E-07	1,70686E-06	0,581578431	2,80921E-11	1,61831E-05	0,499698261	0,60775824	0,096135816	-0,742142271	-0,542500245
SCD	6,58E-09	0,0132	0,000293	1,85E-09	0,0603	1,37278E-08	0,034555414	0,003254676	5,55E-09	0,115809813	0,187499979	-0,070361496	0,126640779	-0,2154405	-0,0989055
SDHA	0,0447	0,978	0,517	0,736	0,006	0,055841033	0,987611794	0,655824074	0,767756345	0,016385563	-0,225109596	-0,079725649	-0,00622584	0,04794521	0,343372023
SETD1A	1,13E-14	0,00207	0,0117	6,21E-21	0,00000214	4,22209E-14	0,007334224	0,04145431	7,97597E-20	2,25523E-05	-0,404316638	-0,157719461	-0,120043009	0,485589245	0,285829128
SETD1B	0,0000048	0,0696	0,00276	7,18E-10	0,857	8,32405E-06	0,124372174	0,0141795	2,34205E-09	0,900836317	-0,121868226	0,035147371	-0,05907299	0,174206333	-0,005237574
SF3B1	2,46E-23	0,151	0,00000411	2,4E-26	0,000139	1,98247E-22	0,235973384	0,000178785	7,58769E-25	0,000751697	-0,753321031	0,120793405	-0,26841681	0,762955055	0,329196255
SF3B1.pT313	1,58E-20	0,652	0,00067	8,93E-25	0,0000981	9,83909E-20	0,732163934	0,005152722	2,15896E-23	0,000567875	-0,612585145	-0,053068299	-0,214514404	0,7245615	0,293672447
SFRP1	0,0402	0,0989	0,639	0,817	0,737	0,050681595	0,165909796	0,750368571	0,831155941	0,823116848	0,059541376	-0,053618437	0,012602887	-0,012026443	-0,026406156
SGK1	2,48E-16	0,137	0,767	3,36E-12	0,00458	1,04008E-15	0,216565385	0,851991892	1,45364E-11	0,01374	0,486438798	0,079081906	-0,038456633	-0,381987031	-0,194829564
SGK3	1,32E-26	0,000000927	0,101	2,74E-10	0,029	1,66891E-25	1,46537E-05	0,213974227	9,46336E-10	0,062731579	-0,792730862	0,435585708	-0,099224916	0,465367365	0,153917739
SHC1.pY317	2,69E-12	0,028	0,779	0,000000575	0,605	8,01152E-12	0,063933333	0,858264706	1,34276E-06	0,714525862	0,296244711	-0,078726445	-0,014207063	-0,210340785	-0,021899523
SIRT1	0,000000117	0,433	0,137	0,932	0,000679	2,20583E-07	0,540920973	0,27137799	0,934273171	0,002763059	0,214132814	-0,036975498	-0,033240455	-0,028364127	-0,162278228
SIRT6	0,0137	0,0146	0,313	0,00697	0,000268	0,017706604	0,036813497	0,478226766	0,010892281	0,001321071	-0,103084478	-0,097256831	-0,04855717	0,119470286	0,170018609

SMAD1	0,0000057	0,00672	0,213	7,06E-08	0,000349	9,80209E-06	0,019728	0,37572103	1,80227E-07	0,001629989	-0,308633477	-0,127157108	-0,090242171	0,341907251	0,260856448
SMAD2	1,68E-14	0,637	0,433	1,28E-08	0,00295	6,11044E-14	0,725227147	0,581578431	3,53074E-08	0,009622619	-0,453280423	0,026611142	-0,016264334	0,306506651	0,217397166
SMAD2.pS245_250_255	0,0571	0,000107	0,606	0,313	6,73E-09	0,070687048	0,000610792	0,726139942	0,359337989	4,29201E-07	-0,085010356	-0,200308048	-0,044179523	0,052301511	0,339666092
SMAD2.pS465_467	1,67E-14	0,000397	0,0138	1,5E-10	0,297	6,11044E-14	0,001773554	0,046112195	5,50446E-10	0,422337931	0,333435858	-0,166439085	0,077890107	-0,258548131	-0,058887822
SMAD5.pS463_465	1,81E-23	0,0129	0,00593	1,46E-20	0,0549	1,48782E-22	0,033986538	0,026782747	1,81836E-19	0,106937915	0,527004628	-0,133217633	0,113293171	-0,470536225	-0,140661055
SMARCA2	3,92E-27	0,678	0,01	1E-29	0,000729	5,96711E-26	0,75109973	0,039142857	1,0275E-27	0,002937441	-0,794981994	-0,02159294	-0,150947417	0,82369031	0,278641997
SMARCA4	2,07E-10	0,306	0,0571	2,84E-15	0,797	5,21945E-10	0,415069307	0,143098171	1,91351E-14	0,859755906	-0,502364448	0,09855016	-0,07684795	0,509942199	0,036385067
SMARCB1	9,56E-31	0,645	0,0246	2,83E-32	0,000946	4,36573E-29	0,728282967	0,075452239	5,81565E-30	0,003667981	-1,076674161	0,014100901	-0,126309151	1,01711218	0,333718135
SNAI1	0,0409	0,0551	0,208	0,244	0,529	0,051406422	0,102936818	0,368482759	0,288172414	0,641353982	0,0661511	-0,01239351	0,027270447	-0,051990513	-0,052299736
SOD1	1,02E-12	0,0268	0,206	0,00000182	0,0314	3,12851E-12	0,061880899	0,368113043	3,93695E-06	0,066867358	0,458625301	-0,157971366	0,06509051	-0,294557035	-0,172280655
SOD2	0,0272	0,229	0,32	0,321	0,232	0,034503704	0,32567128	0,483	0,366475	0,348	-0,245156115	0,16872881	0,12611433	-0,102560716	0,08212915
SOX17	3,76E-13	0,854	0,1	4,94E-14	0,0305	1,23629E-12	0,902298201	0,212953368	2,7437E-13	0,065289063	0,325054964	0,010976817	0,065174917	-0,340528246	-0,116922432
SOX2	2,11E-27	0,0000446	0,0112	2,09E-11	0,0000815	3,46884E-26	0,000295655	0,0411	8,33971E-11	0,000507523	0,74075811	-0,260772459	0,006173074	-0,355394429	-0,271833404
SPARC	2,74E-23	0,0849	0,00825	1,45E-22	0,0466	2,16565E-22	0,145999582	0,033242647	2,20722E-21	0,093318841	0,659808889	-0,11592952	0,125857078	-0,604821888	-0,186613721
SPI1	2,98E-38	0,0000185	0,175	7,9E-20	0,0539	6,1239E-36	0,000149088	0,331451613	8,32538E-19	0,105994737	-2,186288244	0,739439753	0,013640269	1,289751427	0,472166191
SPP1	6,01E-12	0,685	0,000113	2,1E-20	0,0549	1,69186E-11	0,75276738	0,001615655	2,53853E-19	0,106937915	0,406701855	-0,057457171	0,207827142	-0,528281178	-0,134627282
SRC	0,000157	0,00583	0,829	2,82E-08	0,207	0,000241674	0,017749111	0,894275591	7,42962E-08	0,318640449	0,59350311	0,445163877	0,03380746	-0,814090299	-0,298679332
SRC.pY527	8,57E-10	0,00000159	0,912	1,08E-13	0,00031	1,98998E-09	2,33389E-05	0,944161209	5,69077E-13	0,001481512	0,705364564	0,598878917	0,004589214	-0,879538572	-0,490211243
SRSF1	0,449	0,105	0,473	0,425	0,00975	0,490795213	0,173313253	0,623086538	0,469556452	0,025523885	0,022066871	0,085844276	0,045169974	-0,064898081	-0,112245721
SSBP2	5,71E-13	0,137	0,129	3,49E-18	0,047	1,83345E-12	0,216565385	0,258629268	3,04825E-17	0,093318841	-0,806417452	-0,220823295	-0,162610707	1,041344803	0,250105539
STAT1	0,00159	0,425	0,65	1,07E-08	0,156	0,002261211	0,535431193	0,760116477	2,99163E-08	0,250453125	0,315691976	0,037214985	0,047569958	-0,506101284	0,109983765
STAT1.pY701	1,03E-18	0,205	0,967	5,63E-14	0,635	5,6444E-18	0,301989247	0,969358537	3,08524E-13	0,743547009	0,375990179	-0,068115813	-0,004472358	-0,373975696	0,054687116
STAT3	0,000000566	0,563	0,00162	0,298	0,931	1,03389E-06	0,661122857	0,009649565	0,345983051	0,94948139	-0,319292888	0,065181927	0,173577495	0,037296164	0,040997795
STAT3.pS727	2,41E-17	0,265	0,115	0,0377	0,0000119	1,16531E-16	0,366717172	0,239923858	0,052703061	8,89255E-05	-0,415848923	0,055808115	0,059654329	0,090565135	0,294469325
STAT3.pY705	0,497	0,0477	0,138	0,0238	0,0459	0,538963061	0,092040845	0,27137799	0,034442958	0,092475	-0,020534711	-0,079873108	0,049906891	-0,044203204	0,094839459
STAT5A	3,67E-14	0,886	0,00312	3,56E-18	0,0049	1,30032E-13	0,92396962	0,015831111	3,04825E-17	0,014182394	-0,53924665	-0,029477054	-0,208520949	0,666218884	0,233702177
STK11	1,4E-23	0,135	0,728	6,06E-17	0,879	1,17429E-22	0,21505814	0,824264463	4,88365E-16	0,914605063	0,689061253	-0,053862034	-0,090893159	-0,501541023	-0,086302721
STK4	0,0000187	0,00000499	0,0132	0,0822	0,00000255	3,09907E-05	5,69692E-05	0,045176033	0,107251429	2,62013E-05	0,308520623	0,298723624	-0,120288195	-0,138069351	-0,382076427
STMN1	4,78E-28	0,913	0,233	3,26E-10	0,0000508	9,35514E-27	0,942821608	0,3990125	1,10732E-09	0,000326231	-0,88248901	0,033213112	0,122090285	0,475025736	0,35858672
SUZ12	0,0147	0,125	0,0819	0,000000218	0,456	0,018939498	0,203063241	0,182939674	5,30166E-07	0,573896341	-0,0836598	-0,060874211	-0,06242662	0,208022779	0,01007468
SYK	6,27E-29	0,000119	0,00963	0,0221	0,00173	1,61061E-27	0,000669986	0,038426505	0,032209574	0,006077179	-1,57107585	0,585550177	0,360549307	0,361751199	0,458615474
TAPBP	0,335	0,0063	0,596	0,0928	0,157	0,374144022	0,018763043	0,718346041	0,11919	0,251077821	0,061124933	0,187904868	-0,017330415	-0,110704156	-0,119984315
TFRC	0,000029	0,00325	0,00803	7,22E-08	0,312	4,71107E-05	0,010666429	0,032676535	1,83174E-07	0,440558219	1,028895605	-0,79165113	0,537106738	-1,21599722	0,199559624
TGM2	3,57E-23	0,00427	0,0000398	1,04E-28	0,279	2,76843E-22	0,013604419	0,000822	8,5488E-27	0,40662766	2,188779589	-0,707001551	0,720810869	-2,340673121	-0,30813692
TIGAR	0,401	0,000000123	0,501	0,0968	0,0661	0,443040323	2,66068E-06	0,64146729	0,123940187	0,124050685	-0,021710497	0,247174792	-0,008660871	-0,087834402	-0,115768937
TNFRSF4	1,05E-24	0,552	0,35	1,84E-08	0,000000491	1,10654E-23	0,651931034	0,501219512	5,00821E-08	6,7267E-06	0,530742235	0,017620487	-0,058763775	-0,297880555	-0,26460144
TNK1	2,33E-22	0,0125	0,000411	1,58E-11	0,000000037	1,71005E-21	0,03336039	0,004021929	6,36647E-11	1,20012E-06	0,533979632	-0,146551023	0,081514458	-0,304673214	-0,300757909
TP53	2,31E-15	0,209	0,49	9,17E-08	0,327	9,042E-15	0,304606383	0,633301887	2,29809E-07	0,452515152	0,492371562	-0,082779185	-0,051947455	-0,417479658	0,043561567
TP53BP1	8,88E-24	0,232	0,00479	5,72E-33	0,00347	7,93409E-23	0,327955479	0,022371477	2,35092E-30	0,011055581	-1,048679748	-0,192278141	-0,359788488	1,448499589	0,332556702
TRAP1	0,00022	0,675	0,838	0,208	0,00216	0,000337388	0,749797297	0,901617801	0,252176991	0,007460168	-0,272441972	-0,053532381	-0,021892635	0,11042881	0,322675364
TRIM28	8,51E-16	0,595	0,0604	1,35E-18	0,0217	3,46298E-15	0,683086592	0,148008929	1,26102E-17	0,048736066	-0,719991997	0,051343961	-0,098304902	0,642918512	0,247142358
TSC1	0,57	0,0929	0,202	0,0466	0,661	0,608493506	0,15712716	0,364131579	0,063842	0,76658427	-0,031481534	0,149338587	0,10634123	-0,165390043	-0,075488131
TSC2	7,36E-31	0,0405	0,144	3,28E-11	1,86E-08	3,7812E-29	0,081997537	0,281828571	1,29623E-10	7,6446E-07	-0,639352859	0,117823143	-0,07205707	0,379147848	0,364756717
TSC2.pT1462	0,0873	0,628	0,333	0,0269	0,229	0,105530294	0,718963788	0,48705694	0,0385223	0,344758242	0,057706065	0,018619788	0,02028745	-0,079685112	-0,027993298
TUBA1A	0,00513	0,103	0,376	0,664	0,347	0,006935625	0,171388664	0,522081081	0,701552699	0,469134868	-0,205503087	0,132981278	0,111194249	-0,040881652	0,010903638
TXNRD1	0,00477	0,0000662	0,939	1,42E-09	0,49	0,006491623	0,000400121	0,957640199	4,35537E-09	0,602964072	-0,111563429	-0,208976631	-0,028822389	0,322131372	0,010442431
TYRO3	0,000000808	0,0795	0,333	0,128	0,333	1,45017E-06	0,137867089	0,48705694	0,161374233	0,454694352	0,248376636	-0,047426056	-0,076409046	-0,094136053	-0,043667554
UGT1A1	2,82E-24	0,00454	0,0000138	4,99E-21	0,00027	2,89755E-23	0,014306565	0,000405129	6,61577E-20	0,001321071	1,126380277	-0,337453761	0,22574978	-0,832854427	-0,441062775
ULK1.pS757	0,00201	0,233	0,322	0,00123	0,935	0,002829144	0,327955479	0,483	0,002071844	0,950248157	0,132436907	-0,077346066	0,04746708	-0,115795411	-0,020100481
VASP	0,000978	0,0000783	0,00135	0,0000321	0,683	0,001430456	0,000466396	0,008807143	6,05188E-05	0,786310924	-0,273269134	0,398160998	0,266381808	-0,387185524	0,014753909
VAV1	0,00000507	0,00000207	0,0787	0,0836	0,562	8,75534E-06	0,000028359	0,180702235	0,108389905	0,679358824	-0,454462473	0,450061769	-0,068467975	0,147946113	0,039522836
VCP	0,00000637	0,0462	0,00316	0,000000641	0,223	1,08185E-05	0,090186256	0,015838537	1,48006E-06	0,336959559	-0,399597973	0,223217653	-0,241426968	0,444049014	0,104313773
VDAC1_VDAC3	0,758	0,157	0,847	0,136	0,00966	0,780796992	0,241674157	0,908860825	0,168870091	0,025450385	-0,022784193	0,069365088	0,026053642	0,037960997	-0,136170887
VHL	2,93E-10	0,00113	0,255	0,000287	0,215	7,16804E-10	0,004300278	0,422600806	0,000517355	0,327277778	0,258867689	-0,127412954	0,013504961	-0,133421585	-0,060847864
VIM	0,247	0,00000004	0,0000945	0,00000693	0,0000036	0,281991667	0,000001096	0,0014385	1,37596E-05	3,52286E-05	-0,215093341	0,835940744	-0,494135994	0,65102074	-0,694390848
VTCN1	5,56E-27	0,0468	0,000114	3,4E-19	0,000000292	7,87986E-26	0,090730189	0,001615655	3,32714E-18	5,0005E-06	0,527854279	-0,090794605	0,101046279	-0,394449566	-0,268259909
WDR5	8,67E-23	0,123	0,000119	3,21E-23	0,00868	6,47885E-22	0,200607143	0,008017869	5,27724E-22	0,023016	-0,763197634	0,131197463	-0,243894356	0,826190191	0,210154032
WDR77	0,0919	0,00385	0,0157	0,0379	0,0818	0,110038081	0,012459449	0,051211905	0,052803051	0,147455263	0,324040401	-0,22595026	-0,215390391	0,062217412	0,067130901
WDR82	3,98E-13	0,000227	0,0798	0,595	0,073	1,29824E-12	0,001110679	0,181693443	0,636249351	0,135464126	0,473258779	-0,183856206	-0,099506542	-0,103254835	-0,151733614
WEE1	2,16E-16	0,0000626	0,618	2,55E-12	0,000000199	9,2475E-16	0,000384009	0,736226087	1,12694E-11	3,75505E-06	-0,481940332	-0,303716585	-0,004075977	0,449115807	0,417174279
WEE1.pS642	0,00173	0,371	0,0724	0,00621	0,000000047	0,002443402	0,488472843	0,170036571	0,009816577	1,20731E-06	-0,122392181	-0,056654509	0,072127086	-0,147214738	0,307115314
WIPI2	1,65E-15	0,057	0,247	0,236	0,00000678	6,58398E-15	0,105527027	0,414341463	0,28033526	5,80538E-05	0,593904385	-0,167069383	-0,070618035	-0,142760025	-0,329578944
WTAP	2,39E-30	0,894	0,0168	1,98E-26	0,0000106	7,809E-29	0,927863636	0,054368504	6,7815E-25	8,14389E-05	-0,732607121	-0,004827281	-0,126986197	0,689262397	0,311667985
XPA	0,0000349	0,034	0,000552	0,817	0,014	5,58128E-05	0,074329787	0,004630041	0,831155941	0,033847059	0,507091059	0,235327852	-0,263695792	-0,193904431	-0,283716534
XPF	4,82E-09	0,981	0,312	0,497	0,00000144	1,01591E-08	0,988213235	0,478226766	0,541822281	1,74071E-05	0,250743963	-0,00177156	-0,040948067	-0,030664137	-0,236054838
XRCC1	0,0808	0,0000609	0,957	0,0000238	0,17	0,098542433	0,000379241	0,964036765	4,57093E-05	0,268730769	-0,048980254	-0,149176312	0,00664018	0,151103377	0,024962979
YAP1.pS127	0,0968	0,17	0,269	0,077	0,899	0,115318261	0,259739777	0,433564706	0,100786624	0,926037594	-0,002090588	-0,069977654	0,131057489	-0,082506036	-0,020010956
YWHAE	1,13E-17	0,0383	0,0937	6,83E-08	0,000154	5,59554E-17	0,080312755	0,202057068	1,75446E-07	0,00080119	0,403569932	-0,081404904	0,035709142	-0,240380664	-0,20561272
ZAP70	5,74E-28	0,635	0,651	1,07E-09	0,00000165	1,07039E-26	0,724958333	0,760116477	3,33159E-09	1,93757E-05	1,950240857	-0,19093394	-0,188195698	-0,963976538	-0,903242498

Supplementary Table S10. Differential expression proteins and their directionality (up- or down-regulated) for each cluster

DE.proteins.C1	up.regulated.C1	down.regulated.C1	DE.proteins.C2	up.regulated.C2	down.regulated.C2	DE.proteins.C3	up.regulated.C3	down.regulated.C3	DE.proteins.C4	up.regulated.C4	down.regulated.C4	DE.proteins.C5	up.regulated.C5	down.regulated.C5
ABL1.pY412	ABL1.pY412	ACACA	CBL	CBL	ADM	AKT1_2_3.pT308	AKT1_2_3.pT308		ANXA1	ANXA1	ADM	BIRC5	BIRC5	CD4
ADM	ADM	AKT1_2_3.pT308	CD4	CD4	GATA1	TFRC	TFRC		ARID1A	ARID1A	AKT1_2_3.pT308	CCNB1	CCNB1	CDKN1B
ANXA7	ANXA7	ANXA1	DUSP6	DUSP6	NOTCH1.cle	TGM2	TGM2		ASH2L	ASH2L	AKT1S1	CDK1_2_3.pT14	CDK1_2_3.pT14	CDKN2A
ATG4B	ATG4B	ARID1A	FN1	FN1	TFRC				BCL2	BCL2	ATG4B	CHEK1	CHEK1	ERN1
BCL2L1	BCL2L1	ASH2L	ITGAL	ITGAL	TGM2				BRD4	BRD4	BTK	EIF2AK2	EIF2AK2	ETS1
BTK	BTK	BABAM1.pS29	JAK2	JAK2					CASP3	CASP3	CD4	EIF4E.pS209	EIF4E.pS209	FZR1
CASP9	CASP9	BID	MAPK14.pT180_Y182	MAPK14.pT180_Y182					CDK9	CDK9	CDKN2A	HSF1.pS326	HSF1.pS326	LEF1
CBX7	CBX7	BIRC5	NDRG1.pT346	NDRG1.pT346					CDKN1B.pT198	CDKN1B.pT198	ERN1	HSPB1.pS82	HSPB1.pS82	NOTCH1.cle
CD4	CD4	BRD4	PRKCA.pS657	PRKCA.pS657					CSE1L	CSE1L	ETS1	KIT	KIT	S100A4
CDKN1B	CDKN1B	CASP3	PRKCB.pS660	PRKCB.pS660					CTCF	CTCF	FN1	MAPK14.pT180_Y182	MAPK14.pT180_Y182	VIM
CDKN1B.pS10	CDKN1B.pS10	CBL	PTEN	PTEN					DLX1	DLX1	FZR1	PRMT1	PRMT1	ZAP70
CDKN2A	CDKN2A	CCNB1	PYK2.pY402	PYK2.pY402					DPF2	DPF2	HSPB1.pS82	RB1.pS807_811	RB1.pS807_811	
DLST	DLST	CDK1_2_3.pT14	S100A4	S100A4					EEF2	EEF2	IGFBP2	RPS6.pS240_244	RPS6.pS240_244	
ERN1	ERN1	CDK9	SPI1	SPI1					EEF2K	EEF2K	ITGA2	CD4		
ETS1	ETS1	CDKN1B.pT198	SRC.pY527	SRC.pY527					EIF4EBP1	EIF4EBP1	JUNB	CDKN1B		
FN1	FN1	CSE1L	SYK	SYK					EZH2	EZH2	LCK	CDKN2A		
FZR1	FZR1	CTCF	VIM	VIM					HSF1	HSF1	LEF1	ERN1		
GATA3	GATA3	DNMT1	ADM						HSP60	HSP60	LGALS3	ETS1		
H2BFM	H2BFM	DPF2	GATA1						IKB	IKB	MAP2K1	FZR1		
ITGA2	ITGA2	DUSP6	NOTCH1.cle						JAK2	JAK2	NF2	LEF1		
JAG1	JAG1	EEF2	TFRC						KIT	KIT	NFE2L2	NOTCH1.cle		
LCK	LCK	EEF2K	TGM2						KMT2A	KMT2A	NOTCH1.cle	S100A4		
LEF1	LEF1	EIF2AK2							KMT2D	KMT2D	PEA15	VIM		
LGALS3	LGALS3	EIF4E.pS209							LMNB1	LMNB1	PRKCA	ZAP70		
NF2	NF2	EIF4EBP1							MEN1	MEN1	PRKCA.pS657			
NFE2L2	NFE2L2	EIF4EBP1.pS65							MSH6	MSH6	PTK2			
NOTCH1.cle	NOTCH1.cle	EZH2							MSI2	MSI2	PXN			
PAK4	PAK4	GRP75							NCL	NCL	RELA_P100			
PDCD1	PDCD1	HSF1.pS326							NLN	NLN	S100A4			
PEA15	PEA15	HSP60							NUP98	NUP98	SPARC			
PLK1	PLK1	IKB							OMA1	OMA1	SPP1			
PTK2	PTK2	JAK2							PDCD4	PDCD4	SRC			
PXN	PXN	KIT							PRMT1	PRMT1	SRC.pY527			
RELA_P100	RELA_P100	KMT2D							PTEN	PTEN	STAT1			
RSKA1_2_3	RSKA1_2_3	LMNB1							RAD50	RAD50	STK11			
SMAD5.pS463_465	SMAD5.pS463_465	MAPK1_3.pT202_Y204							RPA32	RPA32	TFRC			
SOX2	SOX2	MAPK14							SF3B1	SF3B1	TGM2			
SPARC	SPARC	MAPK14.pT180_Y182							SF3B1.pT313	SF3B1.pT313	UGT1A1			
SRC	SRC	MDM2.pS166							SMARCA2	SMARCA2	ZAP70			
SRC.pY527	SRC.pY527	MEN1							SMARCA4	SMARCA4				
STK11	STK11	MSH6							SMARCB1	SMARCB1				
TFRC	TFRC	MTOR.pS2448							SPI1	SPI1				
TGM2	TGM2	NCL							SSBP2	SSBP2				
TNFRSF4	TNFRSF4	NDRG1.pT346							STAT5A	STAT5A				
TNK1	TNK1	NLN							TP53BP1	TP53BP1				
UGT1A1	UGT1A1	NUP98							TRIM28	TRIM28				
VTCN1	VTCN1	PDCD4							VIM	VIM				
WIPI2	WIPI2	PKM2							WDR5	WDR5				
XPA	XPA	PRKAA1_2.pT172							WTAP	WTAP				
ZAP70	ZAP70	PRMT1							ADM					
ACACA		PRMT7							AKT1_2_3.pT308					
AKT1_2_3.pT308		PTEN							AKT1S1					
ANXA1		RB1							ATG4B					
ARID1A		RB1.pS807_811							BTK					
ASH2L		RPS6.pS240_244							CD4					
BABAM1.pS29		SF3B1							CDKN2A					
BID		SF3B1.pT313							ERN1					
BIRC5		SGK3							ETS1					
BRD4		SMARCA2							FN1					
CASP3		SMARCA4							FZR1					
CBL		SMARCB1							HSPB1.pS82					
CCNB1		SPI1							IGFBP2					
CDK1_2_3.pT14		SSBP2							ITGA2					
CDK9		STAT5A							JUNB					
CDKN1B.pT198		STMN1							LCK					
CSE1L		SYK							LEF1					
CTCF		TP53BP1							LGALS3					
DNMT1		TRIM28							MAP2K1					
DPF2		TSC2							NF2					
DUSP6		WDR5							NFE2L2					
EEF2		WTAP							NOTCH1.cle					
EEF2K									PEA15					
EIF2AK2									PRKCA					
EIF4E.pS209									PRKCA.pS657					
EIF4EBP1									PTK2					
EIF4EBP1.pS65									PXN					
EZH2									RELA_P100					
GRP75									S100A4					
HSF1.pS326									SPARC					
HSP60									SPP1					
IKB									SRC					
JAK2									SRC.pY527					
KIT									STAT1					
KMT2D									STK11					
LMNB1									TFRC					
MAPK1_3.pT202_Y204									TGM2					
MAPK14									UGT1A1					
MAPK14.pT180_Y182									ZAP70					
MDM2.pS166														
MEN1														
MSH6														
MTOR.pS2448														
NCL														
NDRG1.pT346														
NLN														
NUP98														
PDCD4														
PKM2														
PRKAA1_2.pT172														
PRMT1														
PRMT7														
PTEN														
RB1														
RB1.pS807_811														
RPS6.pS240_244														
SF3B1														
SF3B1.pT313														
SGK3														
SMARCA2														
SMARCA4														
SMARCB1														
SPI1														
SSBP2														
STAT5A														
STMN1														
SYK														
TP53BP1														
TRIM28														
TSC2														
WDR5														
WTAP														

Supplementary Table S11. Ontology analysis of cluster C5 differentially expressed proteins with WikiPathways 2023

Term	Overlap	P.value	Adjusted.P.value	Odds.Ratio	Combined.Score	Genes
TROP2 Regulatory Signaling WP5300	7/45	1,13949E-13	1,41297E-11	193,2853186	5760,487358	RB1;CDKN1B;NOTCH1;CDKN2A;CHEK1;CDK2;VIM
IL 24 Signaling Pathway WP5413	14/232	4,58909E-21	1,1381E-18	105,7278287	4951,298106	RB1;CDKN1B;CDKN2A;RPS6;HSPB1;EIF2AK2;MAPK14;ERN1;CCNB1;CHEK1;CDK2;CDK1;BIRC5;EIF4E
Apoptosis Related Network Due To Altered Notch3 In Ovarian Cancer WP2864	6/53	5,71386E-11	3,51883E-09	127,193617	2999,930254	ERN1;CDKN1B;HSPB1;BIRC5;VIM;ETS1
Integrated Cancer Pathway WP1971	5/44	2,58975E-09	6,42259E-08	121,7032967	2406,281435	RB1;CDKN1B;CHEK1;CDK2;CDK1
G1 To S Cell Cycle Control WP45	6/64	1,84853E-10	7,64059E-09	103,0137931	2308,689595	RB1;CCNB1;CDKN1B;CDKN2A;CDK2;CDK1
Cell Cycle WP179	8/120	1,89186E-12	1,56394E-10	78,81746032	2127,555919	RB1;CCNB1;FZR1;CDKN1B;CDKN2A;CHEK1;CDK2;CDK1
miRNA Regulation Of DNA Damage Response WP1530	6/69	2,943E-10	9,12329E-09	94,81428571	2080,834395	RB1;CCNB1;CDKN1B;CHEK1;CDK2;CDK1
DNA Damage Response WP707	6/69	2,943E-10	9,12329E-09	94,81428571	2080,834395	RB1;CCNB1;CDKN1B;CHEK1;CDK2;CDK1
Interferon Type I Signaling Pathways WP585	5/54	7,47584E-09	1,54501E-07	96,81729835	1811,605494	ZAP70;PRMT1;RPS6;MAPK14;EIF4E
CKAP4 Signaling Pathway Map WP5322	7/109	7,09442E-11	3,51883E-09	71,77708978	1677,367965	CCNB1;CDKN1B;LEF1;CDK1;BIRC5;VIM;MAPK14
Thyroid Stimulating Hormone TSH Signaling Pathway WP2032	5/58	1,07938E-08	1,91205E-07	89,49236298	1641,674146	RB1;CDKN1B;RPS6;CDK2;MAPK14
ATM Signaling Pathway WP2516	4/40	1,98584E-07	3,07805E-06	100,6969697	1553,96125	CCNB1;CHEK1;CDK2;CDK1
Retinoblastoma Gene In Cancer WP2446	6/87	1,22074E-09	3,36381E-08	73,67777778	1512,148784	RB1;CCNB1;CDKN1B;CHEK1;CDK2;CDK1
Head And Neck Squamous Cell Carcinoma WP4674	5/73	3,4921E-08	5,77361E-07	69,69887955	1196,742129	RB1;NOTCH1;CDKN2A;RPS6;EIF4E
Gastrin Signaling Pathway WP4659	6/115	6,64029E-09	1,49708E-07	54,67431193	1029,523354	CDKN1B;CDKN2A;RPS6;KIT;BIRC5;MAPK14
ID Signaling Pathway WP53	2/16	0,000192831	0,001062712	118,8095238	1016,2607	RB1;CDK2
Oncostatin M Signaling Pathway WP2374	4/56	7,86941E-07	1,02716E-05	69,65734266	979,0418097	CDKN1B;RPS6;CDK2;MAPK14
Amplification And Expansion Of Oncogenic Pathways As Metastatic Traits WP3678	2/17	0,000218367	0,001152235	110,8833333	934,6725228	NOTCH1;LEF1
Extracellular Vesicles In The Crosstalk Of Cardiac Cells WP4300	2/19	0,000274126	0,001359666	97,82843137	802,3811277	HSPB1;BIRC5
Melanoma WP4685	4/68	1,72654E-06	2,03896E-05	56,5625	750,5499161	RB1;CDKN2A;KIT;ETS1
Imatinib And Chronic Myeloid Leukemia WP3640	2/20	0,000304342	0,001466333	92,38888889	748,1060809	CDKN1B;KIT
Pathogenesis Of SARS CoV 2 Mediated By Nsp9 Nsp10 Complex WP4884	2/21	0,000336109	0,001572736	87,52192982	700,0069722	ZAP70;CD4
Androgen Receptor Network In Prostate Cancer WP2263	5/109	2,63183E-07	3,83938E-06	45,48992674	689,1912878	RB1;CHEK1;CDK2;CDK1;MAPK14
ATM Signaling In Development And Disease WP3878	3/45	2,66884E-05	0,000262015	61,90062112	651,8927841	CHEK1;CDK1;HSPB1
Network Map Of SARS CoV 2 Signaling Pathway WP5115	7/218	9,15641E-09	1,74676E-07	34,50760788	638,6948174	ZAP70;CCNB1;CD4;RPS6;CDK1;BIRC5;EIF4E
Aryl Hydrocarbon Receptor Pathway WP2586	3/46	2,85258E-05	0,000262015	60,45803842	632,6752703	RB1;CDKN1B;CDK2
Translation Inhibitors In Chronically Activated PDGFRA Cells WP4566	3/46	2,85258E-05	0,000262015	60,45803842	632,6752703	RPS6;MAPK14;EIF4E
Roles Of Ceramides In Development Of Insulin Resistance WP5181	2/23	0,000404285	0,001822957	79,17857143	618,6531109	ERN1;EIF2AK2
Spinal Cord Injury WP2431	5/119	4,07842E-07	5,61916E-06	41,47869674	610,2505524	RB1;CDKN1B;CDK2;CDK1;VIM
Follicle Stimulating Hormone FSH Signaling Pathway WP2035	2/24	0,000440686	0,001951609	75,57575758	583,9873316	RPS6;MAPK14
Hepatitis C And Hepatocellular Carcinoma WP3646	3/49	3,45325E-05	0,000305859	56,50661626	580,5269545	LEF1;BIRC5;MAPK14
Glioblastoma Signaling Pathways WP2261	4/82	3,66269E-06	3,94933E-05	46,37762238	580,5232137	RB1;CDKN1B;CDKN2A;CDK2
A Network Map Of Macrophage Stimulating Protein MSP Signaling WP5353	4/82	3,66269E-06	3,94933E-05	46,37762238	580,5232137	RPS6;HSPB1;VIM;MAPK14
Photodynamic Therapy Induced AP 1 Survival Signaling WP3611	3/50	3,6705E-05	0,000313891	55,30157262	564,7726521	RB1;CDKN2A;MAPK14
Cardiac Progenitor Differentiation WP2406	3/53	4,37567E-05	0,000343227	51,97565217	521,6725906	NOTCH1;KIT;MAPK14
Kit Receptor Signaling Pathway WP304	3/53	4,37567E-05	0,000343227	51,97565217	521,6725906	RPS6;KIT;MAPK14
Canonical And Non Canonical Notch Signaling WP3845	2/27	0,000559096	0,00243256	66,49666667	498,0060833	NOTCH1;LEF1
Overlap Between Signal Transduction Pathways Contributing To LMNA Laminopathies WP4879	3/55	4,89192E-05	0,000356823	49,97157191	495,9848538	RB1;NOTCH1;LEF1
DNA IR Double Strand Breaks And Cellular Response Via ATM WP3959	3/55	4,89192E-05	0,000356823	49,97157191	495,9848538	CDKN2A;CHEK1;HSF1
Sphingolipid Metabolism In Senescence WP5121	2/28	0,000601623	0,002528857	63,93589744	474,1409008	RB1;CDK2
Tumor Suppressor Activity Of SMARCB1 WP4204	2/31	0,000738321	0,002906407	57,31321839	413,2931745	RB1;CDKN2A
Host Pathogen Interaction Of Human Coronaviruses Interferon Induction WP4880	2/32	0,000786914	0,003049291	55,4	395,9655078	EIF2AK2;MAPK14
Th17 Cell Differentiation Pathway WP5130	3/69	9,65253E-05	0,000629954	39,34387352	363,7618747	ZAP70;CD4;MAPK14
Urotensin II Mediated Signaling Pathway WP5158	3/69	9,65253E-05	0,000629954	39,34387352	363,7618747	CDK2;CDK1;MAPK14
P38 MAPK Signaling Pathway WP400	2/35	0,000941721	0,003593027	50,35606061	350,871044	HSPB1;MAPK14
Leptin Signaling Pathway WP2034	3/71	0,000105115	0,000668425	38,18286445	349,7723757	RPS6;MAPK14;EIF4E
Primary Focal Segmental Glomerulosclerosis FSGS WP2572	3/72	0,000109591	0,000679464	37,62759924	343,1168973	CDKN1B;NOTCH1;VIM
Host Pathogen Interaction Of Human Coronaviruses MAPK Signaling WP4877	2/36	0,00099632	0,003743749	48,87254902	337,7797704	MAPK14;EIF4E
Prolactin Signaling Pathway WP2037	3/74	0,000118912	0,000719271	36,56399265	330,4335451	ZAP70;RPS6;MAPK14
Effect Of Progerin On Genes Involved In Hutchinson Gilford Progeria Syndrome WP4320	2/37	0,001052413	0,00383821	47,47380952	325,5122514	RB1;LEF1
Neovascularisation Processes WP4331	2/37	0,001052413	0,00383821	47,47380952	325,5122514	NOTCH1;KIT
T Cell Receptor Signaling Pathway WP69	3/75	0,00012376	0,000730771	36,05434783	324,3870709	ZAP70;CD4;MAPK14
Thymic Stromal Lymphopoietin TSLP Signaling Pathway WP2203	2/40	0,001229607	0,004356362	43,71929825	292,9656685	RPS6;MAPK14
Bladder Cancer WP2828	2/40	0,001229607	0,004356362	43,71929825	292,9656685	RB1;CDKN2A
DNA IR Damage And Cellular Response Via ATR WP4016	3/81	0,000155568	0,00089723	33,27090301	291,733496	CHEK1;CDK2;CDK1
Fas Ligand Pathway And Stress Induction Of Heat Shock Proteins WP314	2/43	0,001420102	0,004960357	40,51422764	265,6528602	RB1;HSPB1
Hair Follicle Development Cytodifferentiation Part 3 Of 3 WP2840	3/87	0,000192269	0,001062712	30,88509317	264,2717891	NOTCH1;LEF1;S100A4
T Cell Activation SARS CoV 2 WP5098	3/88	0,000198884	0,001072244	30,5202046	260,1172586	ZAP70;CD4;CDKN2A
Interleukin 11 Signaling Pathway WP2332	2/44	0,001486539	0,005120302	39,54761905	257,5065872	RPS6;BIRC5
Endoplasmic Reticulum Stress Response In Coronavirus Infection WP4861	2/46	0,0016238	0,005516471	37,74621212	242,4434019	ERN1;EIF2AK2
Thyroid Hormones Production And Peripheral Downstream Signaling Effects WP4746	3/93	0,000234175	0,001209906	28,8173913	240,8972619	NOTCH1;RPS6;MAPK14
Breast Cancer Pathway WP4262	4/154	4,42874E-05	0,000343227	24,02909091	240,8870741	RB1;NOTCH1;LEF1;KIT
VEGFA VEGFR2 Signaling WP3888	7/430	9,34189E-07	1,15839E-05	17,02836879	236,4148374	ERN1;RPS6;HSPB1;BIRC5;MAPK14;ETS1;EIF4E
Interleukin 1 IL 1 Structural Pathway WP2637	2/47	0,001694616	0,005635679	36,90555556	235,4684818	MAPK14;EIF4E
Small Cell Lung Cancer WP4658	3/95	0,000249353	0,001262034	28,18809074	233,8664224	RB1;CDKN1B;CDK2
Energy Metabolism WP1541	2/48	0,001766885	0,005690746	36,10144928	228,8303839	PRMT1;MAPK14
Pluripotent Stem Cell Differentiation Pathway WP2848	2/48	0,001766885	0,005690746	36,10144928	228,8303839	NOTCH1;KIT
Translation Factors WP107	2/50	0,001915765	0,00609115	34,59375	216,4751734	EIF2AK2;EIF4E
Prader Willi And Angelman Syndrome WP3998	3/102	0,000307457	0,001466333	26,18577075	211,7689314	RB1;CDKN2A;EIF4E
NRP1 Triggered Signaling Pathways In Pancreatic Cancer WP5144	2/53	0,002149889	0,006749019	32,55392157	199,9572225	CDKN1B;CDK2
Hematopoietic Stem Cell Differentiation WP2849	2/54	0,002230797	0,006915471	31,92628205	194,9226039	NOTCH1;LEF1
DNA Damage Response Only ATM Dependent WP710	3/110	0,000383808	0,001762672	24,21820398	190,4851127	CDKN1B;CDKN2A;LEF1
11P11 2 Copy Number Variation Syndrome WP5348	2/56	0,002396892	0,007338632	30,74074074	185,4767936	CD4;LEF1
Vitamin D Receptor Pathway WP2877	4/186	9,22483E-05	0,000629954	19,77222777	183,7042972	CDKN1B;CDKN2A;CDK2;S100A4
Markers Of Kidney Cell Lineage WP5236	2/59	0,002656677	0,008034829	29,11842105	172,6920116	NOTCH1;KIT
Modulators Of TCR Signaling And T Cell Activation WP5072	2/61	0,002836922	0,008476585	28,12853107	164,9748402	ZAP70;CDKN1B
DYRK1A Involvement Regarding Cell Proliferation In Brain Development WP5180	2/62	0,00292915	0,008546225	27,65833333	161,3322518	CDKN1B;NOTCH1
T Cell Antigen Receptor TCR Pathway During Staphylococcus Aureus Infection WP3863	2/62	0,00292915	0,008546225	27,65833333	161,3322518	ZAP70;CD4
Pathways Affected In Adenoid Cystic Carcinoma WP3651	2/65	0,003214214	0,009268896	26,33730159	151,1806528	NOTCH1;CHEK1
Pleural Mesothelioma WP5087	6/437	1,67019E-05	0,000172586	13,60301624	149,633046	CDKN2A;LEF1;RPS6;CDK2;KIT;MAPK14
TGF Beta Signaling Pathway WP366	3/132	0,000653783	0,002702302	20,06572295	147,1366372	CDK1;MAPK14;ETS1
Measles Virus Infection WP4630	3/136	0,000713062	0,002899005	19,45831971	140,9938673	CDKN1B;CDK2;EIF2AK2
Brain Derived Neurotrophic Factor BDNF Signaling Pathway WP2380	3/137	0,000728399	0,002906407	19,31213498	139,5236505	RPS6;MAPK14;EIF4E
PI3K Akt Signaling Pathway WP4172	5/338	6,57398E-05	0,000465814	14,04332904	135,2345328	CDKN1B;RPS6;CDK2;KIT;EIF4E
Ras Signaling WP4223	3/184	0,001704338	0,005635679	14,26351189	90,92387224	ZAP70;KIT;ETS1
Focal Adhesion PI3K Akt mTOR Signaling Pathway WP3932	4/302	0,000586108	0,002506119	12,00488103	89,34039288	CDKN1B;RPS6;KIT;EIF4E

Supplementary Table S12. Calculations for determining the benefit of the Protein Selector (PS) system for OS and CRD

**Overall Survival**									
Cluster	Color	N	Correct Treatment	Best 5yr OS	Worst 5yr	Delta 5yr OS	Alive at 5yr	% Total Pop Alive at 5yr
C1	Red	91	VH	68%	21%	47%	61,88	15%
C2	Blue	69	CC	58%	0%	58%	40,02	10%
C3	Purple	113	CC	18%	0%	18%	20,34	5%
C4	Green	85	CC	53%	0%	53%	45,05	11%
C5	Orange	61	Undefined	22%	0%	22%	13,42	3%
Total	Black	419	N/A	30%	N/A	N/A	125,70	30%
Alive at 5yr with therapy allocation							180,71	43%
Cure fraction increase							43,76%	43,76%

**Complete Remission Duration**								
Cluster	Color	N	Correct Treatment	best 5yr CRD	worst 5yr	Delta 5yr CRD	Remission at 5yr	% of CR achieving Pop in Remission at 5yr
C1	Red	63	VH	88%	59%	29%	55,13	20%
C2	Blue	53	CC	71%	0%	71%	37,79	14%
C3	Purple	64	CC	35%	0%	35%	22,66	8%
C4	Green	66	CC	68%	0%	68%	45,14	16%
C5	Orange	28	Undefined	46%	0%	46%	12,74	5%
Total	Black	274	N/A	52%	N/A	N/A	143,30	52%
Remisison at 5yr with therapy allocation							173,45	63%
Remission fraction increase							21,04%	21,04%
